# Decreasing rates of disorganised attachment in infants and young children, who are at risk of developing, or who already have disorganised attachment. A systematic review and meta-analysis of early parenting interventions

**DOI:** 10.1371/journal.pone.0180858

**Published:** 2017-07-14

**Authors:** Barry Wright, Lisa Hackney, Ellen Hughes, Melissa Barry, Danya Glaser, Vivien Prior, Victoria Allgar, David Marshall, Jamie Barrow, Natalie Kirby, Megan Garside, Pulkit Kaushal, Amanda Perry, Dean McMillan

**Affiliations:** 1 Hull York Medical School, University of York, York, United Kingdom; 2 Leeds and York Partnership NHS Foundation Trust, Leeds, United Kingdom; 3 Great Ormond Street Hospital and University College, London, United Kingdom; 4 Institute of Child Health, University College London, London, United Kingdom; 5 Tees, Esk and Wear Valley NHS Foundation Trust, York, United Kingdom; 6 Department Health Sciences, University of York, York, United Kingdom; TNO, NETHERLANDS

## Abstract

**Background:**

Disorganised attachment patterns in infants have been linked to later psychopathology. Services have variable practices for identifying and providing interventions for families of children with disorganised attachment patterns, which is the attachment pattern leading to most future psychopathology. Several recent government reports have highlighted the need for better parenting interventions in at risk groups.

**Objectives:**

The objective of this review and meta-analysis was to evaluate the clinical effectiveness of available parenting interventions for families of children at high risk of developing, or already showing, a disorganised pattern of attachment.

**Methods:**

Population: Studies were included if they involved parents or caregivers of young children with a mean age under 13 years who had a disorganised classification of attachment or were identified as at high risk of developing such problems.

Included interventions were aimed at parents or caregivers (e.g. foster carers) seeking to improve attachment.

Comparators included an alternative intervention, an attention control, treatment as usual or no intervention.

The primary outcome was a disorganised pattern in childhood measured using a validated attachment instrument.

Studies that did not use a true Randomised Controlled Trial (RCT) design were excluded from the review. Both published and unpublished papers were included, there were no restrictions on years since publication and foreign language papers were included where translation services could be accessed within necessary timescales.

**Results:**

A comprehensive search of relevant databases yielded 15,298 papers. This paper reports a systematic review as part of an NIHR HTA study identifying studies pre-2012, updated to include all papers to October 2016. Two independent reviewers undertook two stage screening and data extraction of the included studies at all stages. A Cochrane quality assessment was carried out to assess the risk of bias. In total, fourteen studies were included in the review. In a meta-analysis of these fourteen studies the interventions saw less disorganised attachment at outcome compared to the control (OR = 0.50, (0.32, 0.77), p = 0.008). The majority of the interventions targeted maternal sensitivity. We carried out exploratory analyses to examine factors that may influence treatment outcome but these should be treated with caution given that we were limited by small numbers of studies.

**Conclusions:**

Parenting interventions that target parental sensitivity show promise in reducing disorganised attachment. This is limited by few high quality studies and the fact that most studies are with mothers. More high quality randomised controlled trials are required to elucidate this further.

## Introduction

Attachment patterns are measured in infancy using a standardised assessment called the Strange Situation Procedure [1]. This is described extensively elsewhere [1,2] and involves a set procedure assessing infant responses under stress in the presence of an adult not known to them and including the departure and return of the caregiver. Trained observer classifications include secure and insecure (organised) classifications of attachment [1]. Disorganised attachment [2] has been identified as a lack of a co-ordinated response by the child. Research to date suggests that a disorganised attachment pattern in infancy is related to the highest risk of poor outcomes across the lifespan. In childhood, a disorganised attachment pattern has been suggested to be predictive of poorer social functioning [3], poor peer relationships [4], poor school attendance, conduct disorder and academic under achievement [5]. A systematic review and meta-analysis conducted by Van IJzendoorn and Bakermans-Kranenburg (1999) [6] showed an association between disorganised infant attachment and childhood behaviour problems from 12 studies with an overall effect size of r = 0.29. A selective review by Green and Goldwyn (2002)[7] found disorganised attachment to be linked with externalising and internalising problems in early school years [8–10] and aggression and oppositional defiant disorder [11]. In middle childhood, a shift has been found from an earlier disorganised pattern of attachment to controlling, punitive or caregiving behaviour [12].

The Minnesota study of Risk and Adaptation from Birth to Adulthood [13] examined the relationship between disorganised infant attachment and long term mental health outcomes. From this study Carlson (1998) [14] suggested that disorganised attachment was significantly correlated with overall history of psychopathology at age 17 and dissociative episodes at age 19. In a later study within the same sample Carlson and colleagues [15] showed that disorganised attachment was significantly correlated with borderline personality symptoms at age 28.

Much has been written about the antecedents of disorganised attachment, summarised by Lyons-Ruth & Jacobvitz (2008) [12]. In particular two aspects of maternal behaviour have been found to be associated with disorganised attachment. They are maternal frightened or frightening behaviour and maternal disrupted communication during times of infant distress and fearful arousal [16], which includes negative-intrusive behaviour, role confusion, withdrawal, affective contradictory communicative errors, and disorientation. Interestingly, there is less clarity about the role of insensitive parenting in the development of disorganised attachment.

In practice, attachment interventions typically take the form of enhancing parental skills. The majority of parental interventions focus on improving parental sensitivity; developing skills that enable parents to understand their baby’s signals and tune to their infant’s cues consistently, especially when the infant is distressed. This in turn, is intended to enhance infant attachment security. There have been two important meta-analyses that have examined the effectiveness of parental interventions in improving infant attachment. Bakermans-Kranenburg, Van IJzendoorn and Juffer (2003) [17] found that the included interventions significantly improved attachment security, but the effect size was small (d = 0.20). They did not examine the effect of the interventions on disorganised attachment. In a later review, Bakermans-Kraneburg and colleagues [18] did assess the effect of parental interventions on disorganised attachment. Fifteen interventions (from 10 studies) were included in the meta-analysis, which showed no improvement in disorganised patterns as a result of the interventions. This important review and meta-analysis included studies that were not true RCTs as they did not evidence that group allocation of the full sample was made purely by chance [19–23] or did not analyse the effect of interventions on disorganised attachment [24] or they merged the disorganised classification with another classification group [25]. Bakermans-Kranenburg, Van IJzendoorn and Juffer (2003) [17] reported that none of the included studies in their meta-analysis had conducted an intervention specifically aimed at preventing or reducing a disorganised attachment pattern.

Given the strong association between disorganised attachment and subsequent psychopathology, we wanted to systematically review parental interventions delivered to caregivers of children who had, or were at risk of developing, disorganised attachment, examining studies that measured disorganised attachment as an outcome to compare against those with an organised attachment pattern. We chose to examine the highest level of evidence and therefore only included papers that used a randomised controlled trial (RCT) study design. We sought to examine the clinical effectiveness of these interventions.

The present review comes from an NIHR funded systematic review taking the review to 2012, with an update to October 2016.

## Method

### Population, Intervention, Comparison, Outcome, Study Design (PICOS) criteria

The PICOS criteria were as follows:

- Studies were included if they involved parents or caregivers of young children under 13 years who had a disorganised classification of attachment or were identified as at high risk of developing such problems. Included interventions were aimed at parents or caregivers, including foster carers.
- Interventions were excluded if aimed at teachers or teaching assistants (without parents or caregivers) or those not focused at a parental level, but were included if involving parents and teachers.
- Comparators could include no intervention, an alternative intervention, an attention control or treatment as usual.
- The primary aim of the attachment tool had to focus on the measurement of the child’s attachment pattern to the caregiver with the ability to measure a disorganised pattern in childhood.
- Studies that did not use a true RCT design were excluded from the review. Both published and unpublished papers were included, there were no restrictions on years since publication and foreign language papers were included where translation services could be accessed within necessary timescales.

### Search strategy

A search strategy was developed to capture the patient group of children with ‘severe attachment problems’ and the interventions of interest, according to the guidelines for exhaustive searching prepared by the Centre for Reviews and Dissemination (CRD) and the Cochrane Collaboration [26]. The database searches were conducted in January 2012 (Supporting Information 1: Wright et al., 2015 [27] for full details of the databases searched and terms used). For the update of this review, the same search strategies were used. These database searches were conducted in October 2016 and identified papers published after 2011.

The following databases were searched:

- PsycINFO
- MEDLINE and MEDLINE In-Process
- EMBASE
- Social Policy & Practice
- Science Citation Index (SCI)
- Social Science Citation Index (SSCI)
- Conference Proceedings Citation Index- Science (CPCI-S)
- Conference Proceedings Citation Index- Social Science & Humanities (CPCI-SSH)
- ERIC (Educational Resources Information Center)
- Social Services Abstracts
- Applied Social Sciences Index and Abstracts (ASSIA)
- Cochrane Database of Systematic Reviews (CDSR)
- Database of Abstracts of reviews of Effects (DARE)
- Cochrane Central Register of Controlled Trials (CENTRAL)
- Health Technology Assessment (HTA) database
- NHS Economic Evaluation Database (NHS EED)
- Campbell Library
- Health Economic Evaluations Database (HEED)
- Social Care Online
- Research Register for Social Care
- Index to Theses
- OAIster
- OpenGrey
- Zetoc
- ClinicalTrials.gov
- metaRegister of Controlled Trials (mRCT)
- WHO International Clinical Trials Registry Platform (ICTRP)
- UK Clinical Research Network (UKCRN)
- HSRProj (Health Services Research Projects in Progress)

The following organisation websites were also searched:

- American Psychiatric Association (http://www.psych.org/)
- Association Child and Adolescent Mental Health (http://www.acamh.org.uk/)
- Mental Health Foundation (http://www.mentalhealth.org.uk/)
- MIND (http://www.mind.org.uk/)
- Royal College of Psychiatrists (http://www.rcpsych.ac.uk/)
- National Collaborating Centre for Mental Health (NCCMH) (http://www.nccmh.org.uk/)
- National Institute of Mental Health (NIMH) (http://www.nimh.nih.gov/index.shtml)
- Institute for Attachment & Child Development (http://www.instituteforattachment.org/)
- Association for Treatment and Training in the Attachment of Children (http://www.attach.org/)
- YoungMinds (http://www.youngminds.org.uk/)
- British Association for Adoption and Fostering (www.baaf.org.uk/)

### Data collection

In the initial screening phase, titles and abstracts were dual screened and included in the next screening phase if they met the criteria described in detail in Wright et al., 2015 [27]. The reference lists of papers that were included in the initial screen were manually checked, to ensure all studies were identified. Attachment systematic reviews and meta-analyses were checked for additional papers and authors were contacted to clarify details and provided additional studies that were unpublished or ongoing. In a second screening phase, full papers of the resulting identified literature were obtained and reviewed independently, according to the criteria for this review, with disagreements discussed and resolved between reviewers with the assistance of a third reviewer when required. For those papers meeting the criteria for the review, full data extraction was conducted by two independent reviewers, see the Health Technologies Assessment online report for full details [27].

The same process was followed as closely as possible for the update of the review. In the initial phase, titles and abstracts were split into four and screened individually by four reviewers. References from books, reviews and papers included from the first sift were manually checked, and any relevant studies were brought through. This includes one paper published prior to 2011 [28] that was discovered that met all inclusion criteria. For the second phase, papers were dual screened and those that met the PICOS criteria were included for data extraction, which was carried out by three reviewers. Disagreements at any stage of the process were discussed and resolved between all reviewers.

Child attachment classifications were extracted from intervention and control groups as the primary outcome for the meta-analysis. Information was collected on demography (including the age of parent and child), ethnicity and the risk (of severe attachment problems) characteristics of the sample. Information was extracted regarding who was involved in the intervention; whether it was delivered to the parent alone, parent and child dyads or a mixture of the two; whether the intervention was for foster carers, and whether a male caregiver was involved. Papers were recorded as having male caregiver involvement if they reported having at least one male caregiver participating. Intervention characteristics were also extracted, including the aim or focus of the intervention, the number of sessions and length of time it ran for, where it was delivered, by whom it was delivered, the care or alternative treatment received by the comparison group and the length of time participants were followed up for.

### Meta-analysis

For the meta-analysis, the research team explored disorganised attachment as measured by the Strange Situation Procedure [1–2]. Data were extracted on the numbers of patients experiencing the outcome for each group. The odds ratio (OR) and 95% confidence interval (CI) were calculated for each study outcome. The ORs were pooled using a fixed-effect model or random effects model [the Mantel–Haenszel (M–H) method] and the corresponding 95% CIs were calculated. Where the analysis indicated significant heterogeneity a random effects model was chosen, otherwise a fixed effects model was applied. Statistical heterogeneity was assessed using the Cochran’s Q test. The Cochran’s Q tests the presence versus the absence of heterogeneity and the p value is stated. The I^2^ index describes the percentage of variation across studies that is due to heterogeneity rather than chance. Interpretation is as follows: 0% to 40%: might not be important; 30% to 60%: may represent moderate heterogeneity*; 50% to 90%: may represent substantial heterogeneity*; 75% to 100%: considerable heterogeneity. (*The importance of the observed value of I^2^ depends on (i) magnitude and direction of effects and (ii) strength of evidence for heterogeneity (e.g. P value from the chi-squared test, or a confidence interval for I^2^)[29]. A funnel plot was used to test for publication bias. Cohen’s d effect sizes were calculated [30] to make comparisons to previous meta-analyses on attachment interventions.

### Exploratory analyses

Exploratory analyses were conducted to explore treatment factors. Due to the small number of included studies, these analyses are purely exploratory, intended to guide future research, rather than make claims about the effectiveness of intervention components. Features of the intervention were explored through three analyses including number of sessions (based on previous meta-analysis research for comparison [22]), whether video feedback was used, and whether maternal sensitivity was the main focus. Participant related factors were explored through four analyses including age of the child at the start of the intervention, whether partner (usually male) caregivers were involved, foster carer studies and whether the intervention was with caregiver alone or together with their child. The length of follow-up as a research design feature was also explored through an analysis. A sensitivity analysis was performed excluding the two studies [31, 32] which had active intervention in the control group. All analyses were undertaken on Review Manager 5.3.

### Quality assessment

The quality assessment was conducted with the ‘risk of bias’ assessment for RCTs using the criteria recommended by the Cochrane Handbook [26]. The recommended approach for assessing risk of bias in studies included in the Cochrane Review is a two-part tool, addressing six specific domains including:

- Sequence generation and allocation concealment (selection bias)
- Blinding of participants and providers (performance bias)
- Blinding of outcome assessor (detection bias)
- Incomplete outcome data (attrition bias)
- Selective outcome reporting (reporting bias)
- Other sources of bias.

Two independent reviewers assigned a rating of high or low bias for all included papers, under the six domains. A rating of unclear was assigned where there was insufficient information to judge the level of bias. Any disagreements were resolved by arbitration and a third party when required. The same process was followed for the update, where three independent reviewers conducted the quality assessment.

## Results

The initial database searches identified 15,621 records for the three reviews. After the records were de-duplicated, this left 10,167 records for the initial screening of title and abstract for the three reviews. Of these, 445 met first-sift inclusion criteria for this review, including 21 that were identified through other sources. These papers were fully screened and eight of these studies met the final inclusion criteria. An additional four were not included in the meta-analysis but discussed included interventions with the same sample so any additional information about trial design was included in the quality assessment.

The update of the main review identified a further 5,222 records. After these were de-duplicated, this left 5,131 records for initial screening. 230 of these met the criteria for the first sift, including five identified through other sources. After fully screening these papers for inclusion criteria, seven were included in the final meta-analysis. These were then combined with the studies included from the initial review and are reported in S1 Fig.

### Study characteristics

Table 1 shows the fourteen intervention studies that met the criteria. All the studies were conducted by authors affiliated with Western universities; five from USA [28 & 31–34], two from Canada [35, 36], four from UK [37–40], one from the Netherlands [41], one from France [42] and one from Australia [43]. Five studies had a majority white or Caucasian ethnicity [34, 35, 38, 39, 41]. The remainder had reported majority ethnicity of 61% African American [31], 43% African American [32], 45% Latino [33], 74% minority race [28] and 48.6% 1^st^ generation immigrants to France [42]. Two studies did not specify ethnicity [40, 43]. One intervention was delivered in South African shanty towns [37]. Sample sizes ranged from 43 [43] to 449 [37] and included 1816 children in total. The age of the child at the beginning of the intervention ranged from neonatal [33] to a mean age of 3.35 years [36]. The parents mean age ranged from 18 [35] to 35.5 years [43]. The majority of interventions were delivered to mothers and their infants or young children, with just three involving male caregivers as well [31, 33, 43]. We found no RCTs with gay couples.

**Table 1:** Characteristics of included intervention studies and disorganised attachment outcomes.

Study	Participants	Sample risk	Intervention focus	Duration/intensity/delivery	Control group	Time point disorganisation assessed	Disorganised attachment outcomes
Bernard (2012)	N = 120. Child: NR. Parent: 28 years. 61% African American. Male caregiver involved	Child protective services—children considered for fostering	Maternal sensitivity. Antecedents of disorganisation addressed	10 weekly homes visits with 6 video feedback sessions. Delivered by social workers or psychologists	Developmental Education for Families (DEF): A Manualised home visitation programme	Post intervention only	Significantly less disorganisation in intervention group
Cassidy (2011)	N = 220. Child: 6.5 months. Parent: 24years. 43% African American. Male caregiver not involved	Economically stressed mothers. Irritable infants	Maternal sensitivity. Antecedents of disorganisation not addressed	3, hour long home visits over 2.5 months and a later 4^th^ brief visit. Video feedback on all 4 sessions. Delivered by masters and doctoral level clinicians	3 hour long psychoeducational home visit sessions by the same interventionist	Post intervention only	Data available but no analysis comparing disorganised groups
Cooper (2009)	N = 449. Child: prenatal. Parent: 26 years. Ethnicity NR. Male caregiver not involved	Poverty in South Africa, predominantly shanty towns. Pregnant mothers	Maternal sensitivity. Antecedents of disorganisation not addressed	16 sessions over 5 months in the home. Delivered by trained lay community workers	Fortnightly visits by community health worker	Post intervention only	Group differences not significant
Heinicke (1999)	N = 70. Child: prenatal. Parent: 24 years. 45% Latino. Male caregiver included	Low SES, lacked support. Pregnant mothers	Maternal communication, adaptation to child and family support. Antecedents of disorganisation not addressed	Hour long, weekly home visits from pregnancy to 1^st^ year. Optional referral to community services for 12 months. Delivered by mental health professionals	Care as usual from paediatric clinic	Post intervention only	Significantly less disorganisation in intervention group
Moran (2005)	N = 100. Child: 6 months. Parent: 18 years. 81% Caucasian. Male caregiver not involved	Adolescent mothers	Maternal sensitivity. Antecedents of disorganisation not addressed	8 hour long home visits over 5 months with 8 video feedback sessions. Delivered by infant attachment professionals	1 home visit	Post intervention only	Data available but no analysis comparing disorganised groups
Moss (2011)	N = 89. Child: 3.years. Parent: 27 years. Ethnicity: NR. Male caregiver not involved	Child maltreated or being monitored for maltreatment	Maternal sensitivity. Antecedents of disorganisation not addressed	8 weekly 90 minute long home visits with 8 video feedback sessions. Delivered by specifically trained child welfare clinical workers	Standard agency services consisted of monthly visits by child welfare caseworker	Pre and post intervention	Significantly greater decrease in disorganisation from pre to post test in intervention group
Toth (2006)	N = 130. Child: 20 months. Parent: 31 years. 93% European/American. Male caregiver not involved	Depressed mothers	Maternal representation, unresolved conflict and relationship with child. Antecedents of disorganisation addressed	79 weeks of Toddler-Child Psychotherapy sessions. Mean number of sessions = 45. Delivered by a psychotherapist	NR	Pre and post intervention	Significant group differences in disorganisation
Van den Boom (1995)	N = 100. Child: 6 months. Parent: NR. 100% Caucasian. Male caregiver not involved	Low SES mothers. Irritable infants	Maternal sensitivity. Antecedents of disorganisation not addressed	One, 2 hour home visit every 3 weeks, over 3 months. Delivered by: NR	Care as usual	Post intervention only	Data available but no analysis comparing disorganised groups
Fonagy, P., et al. (2016)	N = 76. Child: 3.85 months. Parent: 31.1 years. 63% white ethnicity	Parental mental health problems and social adversity	Maternal sensitivity. Antecedents of disorganisation addressed.	Parent-Infant Psychotherapy sessions offered weekly, delivered by an experienced parent–infant psychotherapist. Mutually agreed ending over 12 month period.	Secondary or specialist primary care	Post intervention only.	Attachment disorganisation did not differ significantly between control and intervention groups.
Gradisar, M., et al. (2016)	N = 43. Child: 10.8 months. Parent: 34.4 years. Male caregiver involved	Parents identified their children as having a sleep problem	Infant’s sleep. Child-parent attachment included as a secondary outcome. Antecedents of disorganisation not addressed.	Two intervention groups; graduated extinction, bedtime fading. Implemented at home by the parents. Also received 24 hour phone support and an information booklet.	Sleep education control. Information leaflet and 24 hour phone support also available.	Post intervention only	No significant differences found between the groups
Stronach, E. P. et al (2013), Cicchetti et al (2006)	N = 189 (Inclusive of 52 nonmaltreated comparison). Child = 13.31 months. Mother = 26.98 years, 74.6% minority race.	Child maltreatment	Maternal sensitivity. Antecedents of disorganisation addressed.	Two intervention groups; CPP/IPP (Child/Infant-Parent Psychotherapy) and PPI (Psychoeducational Parenting Intervention). Weekly sessions with the dyad in the home for 12 months, delivered by Master’s level therapists. Average length CPP/IPP, 46.4 weeks. Average length PPI, 49.4 weeks.	(CS) Community Standard: included assistance in obtaining referrals to services and resources that may have been difficult to access outside the research trial. (NC) Sample of nonmaltreated comparison children.	Pre and post intervention (Cicchetti, 2006). Post intervention follow up (Stronach, 2013)	Post intervention (Cicchetti, 2006) intervention groupshad significantly lower rates ofdisorganised attachment than the CS group. Follow up (Stronach, 2013). CPP had significantly lower rates of disorganised attachment than CS and PPI.
Challacombe F.L., et al. (2017)	N = 71 (Inclusive of 37 healthy controls). Child: NR. Parent: 33.2 years. 85% white ethnicity.	Mothers with post-partum OCD	Improving OCD symptoms. Maternal sensitivity. Antecedents of disorganisation are addressed.	12 hours of iCBT, typically delivered in 4 sessions of 3 hours over a 2 week period. Up to 3 follow-up sessions of 1 hour offered at monthly intervals, delivered face to face by a qualified clinician or therapist.	TAU	Post intervention only	Data available but no analysis comparing disorganised outcomes.
Cooper, P. J., et al. (2015)	N = 301. Child: NR. Parents: 28.3 years.	Mothers at risk of postnatal depression (PND)	To prevent the development of PND. Maternal sensitivity. Antecedents of disorganisation are addressed.	(R-HV) Supportive counselling delivered by NHS employed Health Visitors. 11 home visits, 2 antenatally and then 9 in the first 16 weeks postnatally.	Routine primary care (TAU)	Post intervention only	No analysis for disorganised attachment available. After contacting author, raw figures show more children with disorganised attachment in the intervention than the control.
Tereno. S., et al (2016)	N = 117. Child: 14.2 months. Parents: 23.8 years. Ethnicity: 48.6% were first generation immigrants in France	Mothers with less than 12 years education and/or planning to raise child without a father and/or low income	Maternal sensitivity. Addresses antecedents of disorganisation.	Usual care, assessment home visits, and the CAPEDP program. Trained psychologists visited families at home for a total of 44 visits from the antenatal period up to the child’s second year. Phone calls could be made as often as needed.	TAU	Mid-intervention	Infant disorganisation was significantly reduced in the intervention group compared to the control group

Parent age refers to the mean age at recruitment. Child age is reported at start of the intervention. Only the majority ethnicity is presented. NR = Not reported

Interventions were delivered to families that were at higher risk of disorganised infant attachment for various reasons comprising risk of maltreatment or child protection issues [31, 36], parental mental health problems [34, 38–40] and adolescent mothers [35]. One sample were at risk due to infant sleep difficulties [43]. Six samples were at risk due to social and economic deprivation [32, 33, 37, 41, 42, 44] and two of these samples had irritable infants, in addition [32, 41].

The included studies shared some similarities in the focus of the intervention. With the exception of two studies [33, 43] all of the interventions targeted aspects of maternal sensitivity. Several studies were based wholly on maternal sensitivity at a behavioural level, understanding the baby’s signals and responding to the baby’s cues [35–37, 38, 41, 42]. Three interventions [32, 39, 40] approached maternal sensitivity in the same way but with an extra element which explored and sought to address psychological factors that interfered with the mother’s capacity to respond sensitively. The Attachment and Biobehavioural Catch-up (ABC) intervention [31] helped parents to reinterpret their infant’s behaviour, be aware of and override their own issues, and create an environment that enhanced the child’s capacities. The Toddler Parent Psychotherapy (TPP) [34] and Infant Parent Psychotherapy (IPP) [28] interventions approached maternal sensitivity at the representational level, addressing mothers’ unresolved pasts through dyadic conjoint therapy sessions. Heinicke and colleagues’ (1999) [33] intervention focused on enhancing maternal communication with their infant, rather than sensitivity. However they did engage with parents’ personal adaptation, which sometimes involved resolution of internal conflict, and alternative relationships with the child, using a family systems approach. Gradisar and colleagues (2016) [43] focused on improving infant sleep for those whose parents had identified as having a sleep problem. Child-parent attachment was a secondary outcome. Half of the studies addressed the antecedents of disorganised attachment [28, 31, 34, 38, 39, 40, 42].

Most of the studies used video recording to assist in the coding of attachment styles, however these were not always used to provide participants with feedback. Some of the studies used video feedback to facilitate maternal sensitivity. Three chose to utilise the technique in every session, which equalled eight sessions in two interventions [35, 36] but just four sessions in another intervention [32]. One intervention [31] used video feedback in six of their ten sessions, another [42] used it only once, while the remainder did not use it at all. The interventions were delivered in the participant’s home, or a combination of home and clinic visits [38, 28], the only exceptions being Toth and colleagues (2006) [34], where the location of the Toddler Child Psychotherapy was not specified. Heinicke and colleagues (1999) [33] offered referrals to community services after their main intervention in addition to the course of home visits. For most interventions, visits were weekly, lasting 60–90 minutes, although van den Boom (1995) [41] scheduled more intensive sessions (two hours) but less frequently (every three weeks). One intervention was delivered over a period of only 2 weeks [39], however several interventions lasted 2–3 months [31, 32, 36, 41] while others lasted slightly longer, approximately four to five months [35, 37, 40] up to eleven months [42]. Four of the interventions were much longer in length, lasting 12–24 months [28, 33, 34, 38]. Most of the interventions were delivered by professionals, with the exception of one study which was delivered by lay community workers in South Africa [37], and one study where the intervention was implemented by parents in the home [43].

Most of the interventions were compared against a control group that received care as usual but control group participants in two studies [31–32] received an alternative intervention that was similar in intensity and duration but differed in focus so that intervention effects could be attributed to the content of the intervention. Comparison interventions in these two studies were psychoeducational in focus; addressing topics typically of concern to new parents [32] or the child’s cognitive and linguistic development [31]. The control group in Cooper and colleagues study (2009) [37] and Cichetti and colleagues (2006) [28] study may also have received or had access to more care than usual but less information was provided.

The included studies all used the Strange Situation Procedure [1–2] to measure disorganised attachment. However, the sample in the study by Moss (2011) [36] had a large age range, so the infants aged 12–24 months in the post intervention follow-up were seen in the Strange Situation, but the older children (aged 2–6 years) were seen in the Preschool Separation-Reunion Procedure [45], chosen for its conceptual and technical similarities to the SSP, with the differences allowing for age-related developmental changes [35]. The three studies that started the intervention when the child was over 12 months of age assessed disorganised attachment pre and post intervention [28, 34–35].

### Meta-analysis

The funnel plot (S2 Fig) showed that distribution was roughly symmetrical, indicating that publication bias was not likely to be present.

The main meta-analysis from the 14 included studies (S3 Fig) showed a significant benefit from treatment (OR = 0.50, (0.32, 0.77), p = 0.008). All but two of the included studies showed a trend towards a decrease in the number of children with a disorganised attachment pattern, with the odds ratios ranged from 0.18 to 0.97 and the effect sizes varied from large effects (0.95) [28, 42] and (0.92) [35] to very small effect sizes (0.02) [33] and 0.07 [31] with the others having a medium or large effect [46]. The two studies which showed an increase in disorganised attachment had odds ratios of 2.50 [40] and 2.39 [38], as shown in Table 2.

**Table 2:** Odds ratios and effect sizes of included studies.

Sub groups	k	N	OR	d
**Total set**	15	1503	0.50	0.38
Cassidy 2011		169	0.35	0.58
Challacombe 2017		28	0.76	0.15
Toth 2006		100	0.31	0.65
Cicchetti 2006		104	0.18	0.95
Cooper 2009		318	0.63	0.25
Cooper 2015		142	2.50	-0.51
Bernard 2012		120	0.88	0.07
Fonagy 2016		53	2.39	-0.48
Gradisar 2016		40	0.40	0.51
Heinicke 1999		64	0.97	0.02
Moran 2005		99	0.19	0.92
Moss 2011		67	0.72	0.18
Tereno 2016		117	0.18	0.95
Van Den Boom 1995		82	0.66	0.23

### Exploratory analyses

Table 3 shows the meta-analysis findings with odds ratios and Cohen’s d effect sizes for the exploratory analyses. The p-values for comparing subgroups are displayed, along with the analysis within each subgroup. We have not reported statistical meta-analyses comparisons where there is only one study in a comparison group, for example location, foster carer studies, the main therapeutic intent such as maternal sensitivity, the people involved in the sessions such as caregiver alone or together with child and the length of follow-up.

**Table 3:** Results of the exploratory analyses.

Sub groups	k	N	OR	95% CI	d	Q	p-value	I^2^	p-value
**Number of sessions**						7.23			0.03
<5	4	219	0.79	(0.40, 1.55)	0.13	1.30	0.73	0%	0.49
5–15	5	749	0.63	(0.30, 1.31)	0.25	13.75	0.008	71%	0.22
16+	5	438	0.27	(0.16, 0.45)	0.72	4.29	0.37	7%	<0.001
**Video feedback provided**						0.01			0.97
Yes	5	572	0.49	(0.33, 0.71)	0.39	7.98	0.09	50%	<0.001
No	9	931	0.49	(0.34, 0.71)	0.39	20.27	0.009	61%	0.01
**Age of child**						4.11			0.13
Prenatal	4	641	0.67	(0.28, 1.60)	0.22	7.81	0.05	62%	0.37
<6 months	3	234	0.89	(0.47, 1.69)	0.06	0.19	0.91	0%	0.72
>6 months	6	508	0.32	(0.15, 0.68)	0.63	11.04	0.05	55%	0.003
**Male caregiver included**						0.05			0.83
Yes	3	224	0.45	(0.21, 0.97)	0.44	2.49	0.29	20%	0.04
No	11	1279	0.50	(0.29, 0.84)	0.38	25.51	0.004	61%	0.01

### Features of the intervention

Meta-analyses are reported for two subgroups comparing features of the intervention; number of sessions, and video feedback. Overall, there was statistical significance (p = 0.03) in disorganised attachment when the interventions were split by the number of sessions (<5, 5–15, >16), showing less disorganised attachment at outcome. For the four studies that had interventions lasting 1–4 sessions [32, 39, 41, 43], the analysis revealed a non-significant odds ratio of 0.79 (95% CI: 0.40, 1.55, p = 0.49). Similarly the five studies that had interventions of 5–15 sessions [31, 35–37, 40], the analysis revealed a non-significant odds ratio of 0.63 (95% CI: 0.30, 1.31, p = 0.22). The five studies that had more than 16 sessions [28, 33–34, 38, 42], there was a significant odds ratio of 0.27 (95% CI: 0.16, 0.45, p<0.001). There are no direct comparisons randomising between short and long interventions in terms of number of sessions and so limited conclusions can be drawn from this. Less than half of the interventions used video feedback [31, 32, 35, 36, 42] with no statistically significant difference between those that did use video feedback and those that did not p = 0.97. No study directly compared treatment with or without video feedback although in both groups of the exploratory analysis there were statistically significant odds ratios, with the intervention having less disorganised attachment at outcome compared to the control.

### Features of the participants

Meta-analyses are reported for two subgroups that compare features of the intervention participants; age of the child and male caregiver involvement. In the six studies that delivered the intervention after the child was at least six months of age [28, 32, 34, 36, 39, 43], the analysis shows some promising findings, with the intervention showing less disorganised attachment at outcome compared to the control (OR = 0.32 (95%CI: 0.15, 0.68, p = 0.003). In the analysis on the four papers that delivered or started the intervention prenatally [33, 37, 40, 42], and in the three studies that delivered the intervention before the child was six months of age [35, 38, 41], there was no statistically significant difference in disorganised attachment between the intervention and control (p = 0.37, p = 0.72). There was no statistically significant difference in disorganised attachment between the three studies that reported male caregiver involvement [31, 33, 43] and the eleven studies that did not involve male caregivers [28, 32, 34–42] (p = 0.83) but again no study made a direct comparison.

### Quality assessment

Table 4 displays the results of the Cochrane quality assessment for intervention studies that examined disorganised attachment. Where authors published more than one paper on the intervention with the same sample, additional papers were reviewed and accounted for in the quality assessment but not included in the meta-analysis [44, 47–50]. These papers are presented with the included studies in Table 4. The Cochrane assessment tool illustrated variability in bias between studies. Three domains were consistently rated as high bias across the included studies. These were incomplete outcome data, selective reporting and ‘other’ bias. Incomplete outcome reporting was often rated as high bias because attrition was over 10% across the course of the trial. The reason for the selective reporting item predominantly receiving a rating of high bias across the studies was poor reporting of the secondary outcomes within the studies and lack of an available study protocol. Many of the studies received a rating of high in ‘other bias concerns’. There were various reasons for this including unexplained attrition, unexplained missing data, small sample size/low power and inconsistencies within the data.

**Table 4:** Cochrane quality assessment for intervention studies that included children with a disorganised attachment pattern.

	Random sequence	Allocation concealment	Blinding performance	Incomplete outcome	Selective reporting	Other sources of bias
Cassidy et al (2011)	Unclear	Unclear	Low	High	Unclear	High
Cooper et al (2009)	Low	Low	Low	High	Low	Low
Bernard et al (2012)	Unclear	Unclear	Low	Low	Low	Low
Heinicke et al (1999;2000;2001)	Low	Low	Low	Unclear	Unclear	High
Moran et al (2005)	Unclear	Unclear	Unclear	Low	Low	Low
Moss et al (2011)	Low	Unclear	Low	High	Unclear	High
Toth at al. (2006) Cicchetti et al., (1999)	Low	Unclear	Low	High	High	High
Van den Boom (1994;1995)	Unclear	Unclear	Low	Unclear	High	High
Fonagy, P., et al. (2016)	Low	Low	Low	High	Low	High
Gradisar, M., et al. (2016)	High	Unclear	Low	Unclear	Low	High
Stronach, E. P. et al (2013), Cicchetti, D., et al. (2006)	Unclear	Unclear	Low	High	Unclear	High
Challacombe F.L., et al. (2017)	Low	Low	Low	High	Unclear	High
Cooper, P.J., et al (2015)	Low	Low	Low	High	High	High
Tereno, S., et al (2016)	Low	Low	Low	Low	High	High

A rating of unclear was given where the authors’ descriptions were not sufficient to rate the relevant information. This was most apparent in the random sequence and the method of allocation concealment categories. Blinding was conducted to some extent in thirteen of the studies [28, 31–34, 36–44, 47–50]. The mixed presentation of trial quality across the review suggests that any conclusions should be interpreted with caution.

#### Sensitivity analysis

A sensitivity analysis was performed excluding the two studies [31, 32] comparing with an active intervention, shown in S4 Fig. There remained to be a significant benefit from treatment (OR = 0.50, (0.29, 0.85), p = 0.01).

Whilst subgroup analysis should be treated with caution because data are combined and there is no direct comparison, we report them here for completion. When excluding studies compared with an active intervention, there remained to be no statistical significance in disorganised attachment when the interventions were split by the number of sessions (p = 0.06), between those that did use video feedback and those that did not (p = 0.65) or whether or not there was male caregiver involvement (p = 0.60). There was a statistically significant difference for age of child (p = 0.007). In the five studies that delivered the intervention after the child was at least six months of age [28, 34, 36, 39, 43], the analysis shows the intervention showing less disorganised attachment at outcome compared to the control OR = 0.22 (95%CI: 0.12, 0.42, p<0.001). As shown above, in the analysis that delivered or started the intervention prenatally and before the child was six months of age, there was no statistically significant difference in disorganised attachment between the intervention and control.

## Discussion

The main finding, based on fourteen studies, showed that parental interventions significantly decreased disorganised attachment with a medium effect size (d = 0.38), as shown in Table 2. The studies varied in the strength of their positive intervention effect. Some interventions had little effect on disorganised attachment [32, 33, 35, 37–39, 40, 41, 43]. Five of the studies made a significant difference to disorganised attachment, with a medium [31, 42] or large effect [28, 34, 36]. Two studies [40, 43] showed decreased levels of disorganised attachment in the control condition groups, although these findings were non-significant. The overall finding is interesting given that the previous published meta-analysis [18] produced an overall non-significant effect on disorganised attachment with an effect size of d = 0.05. In contrast to the earlier review, a higher proportion of included interventions in our review were effective in decreasing rates of disorganised attachment. Additionally there were no interventions that had a significantly negative effect on disorganised attachment. The disparity with the earlier review [18] is worthy of further discussion.

Despite some similarities in the inclusion criteria, only two studies were included in both reviews [33, 41]. The present review included *only* true RCT’s; where the full sample was randomly allocated to the intervention or control group. Several studies reported in the earlier review [18] did not meet this criterion. They may have, for instance, randomly assigned part of the sample but allocated a portion of the sample based on therapist time and availability [19] or not reported using random allocation [22]. Furthermore, several recent high quality studies have been included in the present review that were published after the 2005 review. The difference in findings could indicate that attachment intervention studies are using more rigorous trial design and becoming more effective in reducing rates of disorganised attachment.

The impact of this research is therefore important, both in identifying the need for robust high quality methodologies in the field, but also in showing that interventions can improve disorganized attachment in infants.

Previous meta-analyses on parental attachment interventions and attachment insecurity [17, 51] combined the A, C and D (insecure and disorganised classifications) to compare against the B (secure) classification and found only weak intervention effect sizes for improvement in attachment security. They suggested that the least intensive interventions, with fewer than five sessions, were significantly more effective at increasing attachment security than interventions with a higher number of sessions. They concluded that short, focused interventions seemed more effective in improving attachment insecurity and showed that in some cases highly intensive interventions appeared to have a detrimental effect. Interestingly, our current analysis suggests that the “less is more” case put forward by [17] for improving attachment security does not appear to extend to disorganised attachment. In our exploratory work we found that interventions with fewer than 15 sessions did not statistically improve disorganised attachment when compared to the control group, yet interventions with more than 16 sessions did. This is different from the findings of the review by Juffer and colleagues [22]. The groups were small in this sub-group analysis, and so the findings should be treated with caution, with more research necessary to elucidate this further.

Video feedback is commonly used in interventions with a primary focus on maternal sensitivity. It allows the parent to review their own infant’s cues and needs, and reflect on their response to them. Previous research showed mixed results on the value of video feedback in improving insecure attachment. The early review by Van IJzendoorn and Juffer (1995) [51] found strategies such as video feedback to be more successful than therapeutic work alone, at least in the short-term. The later review by Bakermans-Kranenburg, Van IJzendoorn and Juffer (2003) [17], however, found that interventions were more effective when they did not use video feedback. The present review showed that interventions were more effective than a control, whether or not they made use of this technique.

Our exploratory analysis found limited evidence of the benefit of interventions that started when the infant was under six months. The reasons for this are not clear and may be an artefact or may be related to the interventions included. Parents may engage less with pre-natal work because it is not in the “here and now” of mother-child interaction. Infants aged 6–12 months, where our study showed good effect sizes in an exploratory meta-analysis, are within the government guidelines for critical early intervention work within the first two years of life [52]. The finding is in line with outcomes for insecure attachment [17] and a previous review on disorganised attachment [18].

There are a number of studies of which we were aware that might have been expected to be included in a review of this nature. Some of these parental interventions that are intended to improve attachment security have not carried out yet published RCTs, but were mentioned by the research PPI or expert groups. For example, Theraplay [53], Dyadic Developmental Psychotherapy [54] and Watch, Wait and Wonder [19] were all therapies discussed in focus groups where no studies meeting PICO criteria with a RCT at the time of review were found. One finding from our review therefore is that further important research is needed using RCTs to test available interventions currently in clinical use as they may not have an evidence base. It should be noted that the absence of a known intervention in our meta-analysis is not a comment on the intervention itself, but on the presence of available evidence meeting the criteria for this systematic review.

Our criteria excluded any studies that were not focused on interventions at the caregiver/parental level. Interventions which involve a change in caregiver including, for example, adoption as an intervention [55] were excluded. Studies such as the Bucharest Early Intervention Project (BEIP) [56–57] and the English Romanian Adoptee (ERA) study [58] were therefore not included in this review.

The meta-analyses, particularly the exploratory subgroup analyses were limited by the relatively small number of studies included in the review. We considered it important to review only the highest quality evidence; however the tight inclusion criteria may have resulted in a smaller inclusion figure. The categories in the exploratory subgroup analyses therefore need to be interpreted with great caution. We present the results here for discussion and to encourage debate about which parameters to examine in future research. The most frequent reason for exclusion to this review was because studies lacked the robust methodology of an RCT design, highlighting the need for more high quality, rigorous research in this field. Some papers that were potentially relevant, based on titles and abstract, were not retrieved or processed within our timescales. Consideration should also be made of the quality assessment conducted and how this may influence the strength of the findings in this area of research. Attrition rates were high across most studies, and often went unexplained. Future studies should be clear about recruitment, randomisation and attrition and should explore mechanisms of change as well as treatment related factors carefully to inform future meta-analyses. They should also provide clearer methodological reporting such as sequence generation, allocation concealment and primary outcome measures. Overall, the meta-analysis illustrates that parenting interventions significantly decrease disorganised attachment in infants identified as at risk. Whilst variations in intervention design presents some limitations in the extent to which findings can be generalised to clinical practice, most studies focused on seeking to enhance parental attachment and sensitivity. This review presents interesting findings that have not been demonstrated by previous research.

## Supporting information

S1 Fig PRISMA diagram of included studies. (TIF)

S2 Fig Funnel plot of included studies. (TIF)

S3 Fig A meta-analysis of disorganised attachment at outcome comparing a parenting intervention and a control condition. (TIF)

S4 Fig Sensitivity analysis excluding two studies with active intervention for the meta-analysis of disorganised attachment at outcome comparing a parenting intervention and a control condition. (TIF)

S1 File Clinical and cost effectiveness of parenting interventions for children with ‘severe attachment problems’: A systematic review and meta-analysis. Wright, W., Barry, M., Hughes, E., Trepel, D., Ali, S., Allgar, V., Cottrill, L., Duffy, S., Fell, J., Gilbody, S., Glanville, J., Glaser, D., Hackney, L., Manea1, L., McMillan, D., Palmer, S., Prior, V., Whitton, C., Perry, A. Clinical and cost effectiveness of parenting interventions for children with ‘severe attachment problems’: A systematic review and meta-analysis. Health Technology Assessment. 2015; 19: 1–348.
(PDF)

S2 File PRISMA 2009 checklist. (DOC)

## References

[1] Ainsworth M., Blehar M., Waters E., & Wall S. Patterns of attachment: A psychological study of the strange situation. Hillsdale, NJ: Lawrence Erlbaum Associates; 1978.

[2] Main M., & Solomon J. Procedures for identifying infants as disorganized/disoriented during the Ainsworth Strange Situation In Greenberg M.T., Cicchetti D., & Cummings E.M. (Eds.), Attachment during the preschool years: Theory, research and intervention. Chicago: University of Chicago Press; 1990 pp. 121–160.

[3] Futh A., O'Connor T., Matias C., Green J., & Stephen S. Attachment narratives and behavior and emotional symptoms in an ethnically diverse, at risk sample. Journal of the American Academy of Child and Adolescent. 2008; 47: 709–718. 10.1097/CHI.0b013e31816bff65 18434917

[4] Groh A.M., Fearon R.P., Bakermans-Kranenburg M.J., Van IJzendoorn M.H., Steele R.D., Roisman G.I. The significance of attachment security for children’s social competence with peers: a meta-analytic study. Attachment & Human Development. 2014; 16: 103–136. 24547936 10.1080/14616734.2014.883636 PMC4021853

[5] Leschied A.W., Chiodo D., Whitehead P.C., & Hurley D. The relationship between maternal depression and child outcomes in a child welfare sample: Implications for treatment and policy. Child & Family Social Work. 2005; 10: 281–291.

[6] Van IJzendoorn M., & Bakermans-Kranenburg M. J. Disorganized attachment in early childhood: Meta-analysis of precursors, concomitants, and sequelae. Development and Psychopathology, 1999; 11: 225–249. 16506532 10.1017/s0954579499002035

[7] Green J., & Goldwyn R. Annotation: attachment disorganisation and psychopathology: new findings in attachment research and their potential implications for developmental psychopathology in childhood. Journal of Child Psychology and Psychiatry.2002; 43: 835–846. 12405473 10.1111/1469-7610.00102

[8] Fearon R. P., Bakermans-Kranenburg M. J., Van IJzendoorn M. H., Lapsley A.-M. & Roisman G. I. The significance of insecure attachment and disorganization in the development of children's externalizing behavior: A metaanalytic study. Child Development. 2010; 81: 435–456. doi: 10.1111/j.1467-8624.2009.01405.x 20438450 10.1111/j.1467-8624.2009.01405.x

[9] Groh A.M., Roisman G.I. Van IJzendoorn M.H., Bakermans-Kranenburg M.J., Fearon R.P. The Significance of Insecure and Disorganized Attachment for Children’s Internalizing Symptoms: A Meta-Analytic Study. Child Development. 2012; 83: 591–610. doi: 10.1111/j.1467-8624.2011.01711.x 22235928 10.1111/j.1467-8624.2011.01711.x

[10] Lyons-Ruth K. Attachment relationships among children with aggressive behavior problems: The role of disorganized early attachment patterns. Journal of Consulting and Clinical Psychology. 1996; 64: 64–73. 8907085 10.1037//0022-006x.64.1.64

[11] Greenberg M. Attachment and psychopathology in childhood In Cassidy J, & Shaver PR. (Eds.). Handbook of attachment: Theory, research and clinical applications. New York: Guilford Press; 1999 pp. 469–497.

[12] Lyons-Ruth K., Jacobvitz D. Attachment disorganisation: Genetic factors, parenting contexts, and development transformation from infancy to adulthood In Cassidy J. & Shaver P.R. Handbook of Attachment. Theory, Research and Clinical Applications, 2^nd^ Edition: New York: Guilford Press; 2008 pp 666–697.

[13] Sroufe L.A., Egeland B., Carlson E.A., Collins W.A. The development of the person The Minnesota Study of Risk and Adaptation. New York: Guildford Press; 2005.

[14] Carlson E.A. A prospective longitudinal study of attachment disorganization/disorientation. Child Development. 1998; 69: 1107–1128. 9768489

[15] Carlson E.A., Egeland B., & Sroufe L. A prospective investigation of the development of borderline personality symptoms. Development and Psychopathology. 2009; 21: 1311–1334. doi: 10.1017/S0954579409990174 19825270 10.1017/S0954579409990174 PMC11034778

[16] Lyons-Ruth K., Bronfman E., Parsons E. Maternal frightened, frightening, or atypical behavior and disorganised infant attachment patterns. Monographs of the Society for Research in Child Development. 1999; 64: 67–96. 10597543 10.1111/1540-5834.00034

[17] Bakermans-Kranenburg M., Van IJzendoorn M., & Juffer F. Less is more: meta-analyses of sensitivity and attachment interventions in early childhood. Psychological bulletin.2003; 129: 195–215. 12696839 10.1037/0033-2909.129.2.195

[18] Bakermans-Kranenburg M., Van IJzendoorn M., & Juffer F. Disorganized infant attachment and preventive interventions: a review and meta‐analysis. Infant Mental Health Journal. 2005; 28: 191–216. 10.1002/imhj.20046 28682504

[19] Cohen N., Muir E., Parker C., Brown M., Lojkasek M., Muir R., & Barwick M. Watch, wait, and wonder: Testing the effectiveness of a new approach to mother-infant psychotherapy. Infant Mental Health Journal. 1999; 20: 429–451.

[20] Egeland B., & Erickson M. Attachment theory and findings: Implications for prevention and interventioN In Kramer S.A. & Parens H. (Eds.), Prevention in mental health: Now, tomorrow, ever? Northvale, NJ: Jason Aronson, Inc; 1993 pp. 21–59.

[21] Gelfand D., Teti D., Seiner S., & Jameson P. Helping mothers fight depression: Evaluation of a home-based intervention program for depressed mothers and their infants. Journal of Clinical Child Psychology. 1996; 25: 406–422.

[22] Juffer F., Bakermans-Kranenburg M., & Van IJzendoorn M. The importance of parenting in the development of disorganized attachment: Evidence from a preventive intervention study in adoptive families. Journal of Child Psychology and Psychiatry. 2005; 46: 263–274. doi: 10.1111/j.1469-7610.2004.00353.x 15755303 10.1111/j.1469-7610.2004.00353.x

[23] Lyons-Ruth K., Connell D., Grunebaum H., & Botein S. Infants at social risk: Maternal depression and family support services as mediators of infant development and security of attachment. Child Development. 1990; 61: 85–98. 2307048 10.1111/j.1467-8624.1990.tb02762.x PMC7323915

[24] Cooper P., & Murray L. The impact of psychological treatments of postpartum depression on maternal mood and infant development—Postpartum depression and child development. New York: Guilford; 1997.

[25] Sajaniemi N., Makela J., Saolkorpi T., von Wendt L., Hamalainen T., & Hakamies-Blomqvist L. Cognitive performance and attachment patterns at four years of age in extremely low birth weight infants after early intervention. European Child & Adolescent Psychiatry. 2001; 10: 122–129. 11469284 10.1007/s007870170035

[26] Higgins J., & Green S. Cochrane Handbook for Systematic Reviews of Interventions 5.0.2 Chichester UK: John Wiley & Sons, Ltd; 2009.

[27] Wright W., Barry M., Hughes E., Trepel D., Ali S., Allgar V., Cottrill L., Duffy S., Fell J., Gilbody S., Glanville J., Glaser D., Hackney L., Manea L., McMillan D., Palmer S., Prior V., Whitton C., Perry A. Clinical and cost effectiveness of parenting interventions for children with ‘severe attachment problems’: A systematic review and meta-analysis. Health Technology Assessment. 2015; 19: 1–348. 10.3310/hta19520 PMC4780895 26177494

[28] Cicchetti D., Rogosch F. A., & Toth S. L. Fostering secure attachment in infants in maltreating families through preventive interventions. Development and psychopathology, 2006; 18(03), 623–649. 17152394 10.1017/s0954579406060329

[29] Higgins JPT, Green S (editors). Cochrane Handbook for Systematic Reviews of Interventions Version 5.1.0 [updated March 2011]. The Cochrane Collaboration, 2011.

[30] Borenstein M., Hedges L., Higgins J., & Rothstein H. Converting Among Effect Sizes, in Introduction to Meta-Analysis. West Sussex: John Wiley; 2009.

[31] Bernard K., Dozier M., Bick J., Lewis-Morrarty E., Lindhiem O., & Carlson E. Enhancing Attachment Organization Among Maltreated Children: Results of a Randomized Clinical Trial. Child Development. 2012; 83: 623–636. doi: 10.1111/j.1467-8624.2011.01712.x 22239483 10.1111/j.1467-8624.2011.01712.x PMC3305815

[32] Cassidy J., Woodhouse S.S., Sherman L.J., Stupica B., & Lejuez C. Enhancing infant attachment security: An examination of treatment efficacy and differential susceptibility. Development and Psychopathology. 2011; 23: 131–148. doi: 10.1017/S0954579410000696 21262044 10.1017/S0954579410000696

[33] Heinicke C.M., Fineman N.R., Ruth G., Recchia S.L., Guthrie D., & Rodning C. Relationship-based intervention with at-risk mothers: Outcome in the first year of life. Infant Mental Health Journal. 1999; 20: 349–374.

[34] Toth S. L., Rogosch F. A., Manly J. T., & Cicchetti D. The efficacy of toddler-parent psychotherapy to reorganize attachment in the young offspring of mothers with major depressive disorder: A randomized preventive trial. Journal of Consulting and Clinical Psychology. 2006; 74: 1006–1016. doi: 10.1037/0022-006X.74.6.1006 17154731 10.1037/0022-006X.74.6.1006

[35] Moran G., Pederson D.R., & Krupka A. Maternal unresolved attachment status impedes the effectiveness of interventions with adolescent mothers. Infant Mental Health Journal. 2005; 26: 231–249. doi: 10.1002/imhj.20045 28682506 10.1002/imhj.20045

[36] Moss E., Dubois-Comtois K., Cyr C., Tarabulsy G. M., St-Laurent D., & Bernier A. Efficacy of a home-visiting intervention aimed at improving maternal sensitivity, child attachment, and behavioral outcomes for maltreated children: A randomized control trial. Development and Psychopathology. 2011; 23: 195–210. doi: 10.1017/S0954579410000738 21262048 10.1017/S0954579410000738

[37] Cooper P.J., Tomlinson M., Swartz L., Landman M., Molteno C., Stein A., McPherson K., Murray L. Improving quality of mother-infant relationship and infant attachment in socioeconomically deprived community in South Africa: Randomised controlled trial. BMJ: British Medical Journal. 2009; 338: 1–11. 10.1136/bmj.b974 PMC2669116 19366752

[38] Fonagy P., Sleed M., & Baradon T. Randomized Controlled Trial of Parent-Infant Psychotherapy for parents with mental health problems and young infants. Infant mental health journal. 2016; 37(2), 97–114 doi: 10.1002/imhj.21553 26939716 10.1002/imhj.21553

[39] Challacombe F. L., Salkovskis P. M., Woolgar M., Wilkinson E. L., Read J., & Acheson R. A pilot randomized controlled trial of time-intensive cognitive–behaviour therapy for postpartum obsessive–compulsive disorder: effects on maternal symptoms, mother–infant interactions and attachment. Psychological Medicine, 2017; 1–11. 10.1017/S0033291716003573 28137316

[40] Cooper P. J., De Pascalis L., Woolgar M., Romaniuk H., & Murray L. Attempting to prevent postnatal depression by targeting the mother–infant relationship: a randomised controlled trial. Primary health care research & development, 2015; 16(04), 383–397. 25381790 10.1017/S1463423614000401

[41] van den Boom D. C. Do first-year intervention effects endure? Follow-up during toddlerhood of a sample of Dutch irritable infants. Child Development. 1995; 66: 1798–1816. 8556900

[42] Tereno S., Guedeney N., Dugravier R., Greacen T., Saïas T., Tubach F., Ulgen S., Matos I. & Guédeney A. Sécurité de l’attachement des jeunes enfants dans une population française vulnérable. L'Encéphale. 2016. 10.1016/j.encep.2015.11.006 27216594

[43] Gradisar M., Jackson K., Spurrier N. J., Gibson J., Whitham J., Williams A. S., Dolby R. & Kennaway D. J. (2016). Behavioral Interventions for Infant Sleep Problems: A Randomized Controlled Trial. Pediatrics. 2016; 137(6). 10.1542/peds.2015-1486 27221288

[44] Stronach E. P., Toth S. L., Rogosch F., & Cicchetti D. Preventive interventions and sustained attachment security in maltreated children. Development and psychopathology, 2013; 25: 919–930 doi: 10.1017/S0954579413000278 24229539 10.1017/S0954579413000278 PMC3857699

[45] Cassidy, J., Marvin, R.S., & the MacArthur Working Group on Attachment (1992). Attachment organisation in 2 ½ to 4 ½ year olds: Coding manual. Unpublished manuscript. University of Viginia.

[46] Cohen J. Statistical power analysis for the behavioral sciences. New Jersey: Lawrence Erlbaum Associates; 1988.

[47] Cicchetti D., Toth S. L., & Rogosch F. A. The efficacy of toddler-parent psychotherapy to increase attachment security in offspring of depressed mothers. Attachment & Human Development. 1999; 1: 34–66. 11707882 10.1080/14616739900134021

[48] Heinicke C. M., Goorsky M., Moscov S., Dudley K., Gordon J., Schneider C., & Guthrie D. Relationship-based intervention with at-risk mothers: Factors affecting variations in outcome. Infant Mental Health Journal. 2000; 21: 133–155.

[49] Heinicke C.M., Rineman N.R., Ponce V.A., & Guthrie D. Relation-based intervention with at-risk mothers: Outcome in the second year of life. Infant Mental Health Journal. 2001; 22: 431–462.

[50] van den Boom D. C. The influence of temperament and mothering on attachment and exploration: An experimental manipulation of sensitive responsiveness among lower-class mothers with irritable infants. Child Development. 1994; 65: 1457–1477. 7982362 10.1111/j.1467-8624.1994.tb00829.x

[51] Van IJzendoorn M. H., Juffer F., & Duyvesteyn M. G. C. Breaking the Intergenerational Cycle of Insecure Attachment: A Review of the Effects of Attachment-Based Interventions on Maternal Sensitivity and Infant Security. Journal of Child Psychology and Psychiatry. 1995; 36: 225–248. 7759588 10.1111/j.1469-7610.1995.tb01822.x

[52] WAVE Trust. Conception to age 2—the age of opportunity. Surrey: WAVE Trust; 2013.

[53] Booth P., & Jernberg A. Theraplay: Helping parents and children build better relationships through attachment-based play. San Fransisco: John Wiley & Sons; 2009.

[54] Becker-Weidman A. The Dyadic Developmental Psychotherapy Casebook. New York: Jason Aronson Inc. Publishers; 2011.

[55] Jaffari-Bimmel N., Juffer F., Van IJzendoorn M. H., Bakermans-Kranenburg M. J., & Mooijaart A. Social development from infancy to adolescence: Longitudinal and concurrent factors in an adoption sample. Developmental Psychology. 2006; 42: 1143–1153. doi: 10.1037/0012-1649.42.6.1143 17087548 10.1037/0012-1649.42.6.1143

[56] Boris N. W., Hinshaw-Fuselier S.S., Smyke A.T., Scheeringa M.S., Heller S.S., & Zeanah C.H. Comparing criteria for attachment disorders: Establishing reliability and validity in high-risk samples. Journal of the American Academy of Child & Adolescent Psychiatry. 2004; 43: 568–577. 15100563 10.1097/00004583-200405000-00010

[57] Zeanah C.H., Berlin L.J., & Boris N. Practitioner Review: Clinical applications of attachment theory and research for infants and young children. Journal of Child Psychology and Psychiatry. 2011; 52: 819–833. doi: 10.1111/j.1469-7610.2011.02399.x 21554304 10.1111/j.1469-7610.2011.02399.x PMC3670111

[58] O’Connor T., Bredenkamp D., & Rutter M. The English and Romanian Adoptees (ERA) Study Team. Attachment disturbances and disorders in children exposed to early severe deprivation. Infant Mental Health Journal. 1999; 20: 10–29.

