# Supplementary figures and images for: Decreasing rates of disorganised attachment in infants and young children, who are at risk of developing, or who already have disorganised attachment. A systematic review and meta-analysis of early parenting interventions
Source: PLoS One. 2017 Jul 14;12(7):e0180858. doi: 10.1371/journal.pone.0180858 (PMC5510823; doi:10.1371/journal.pone.0180858)

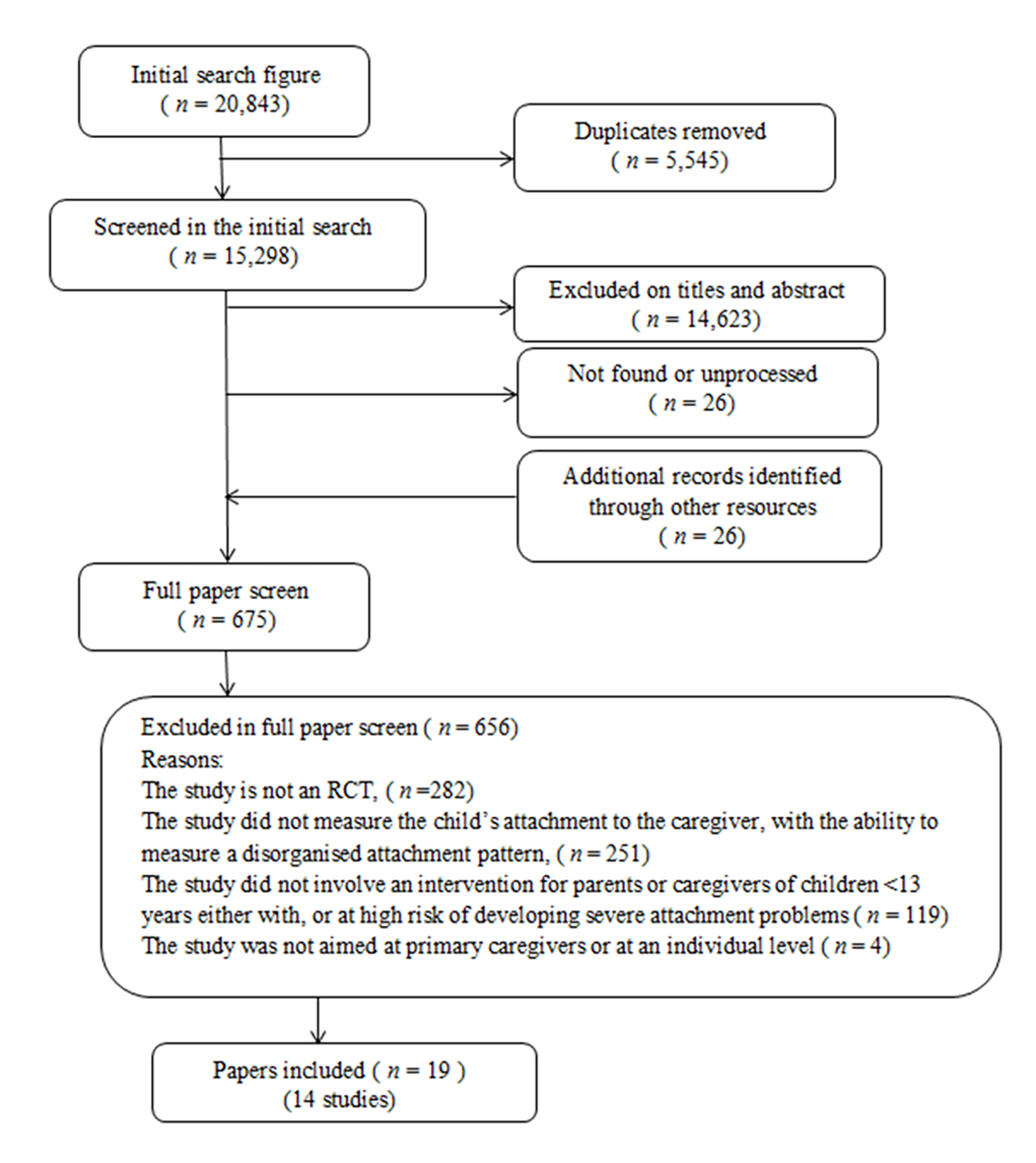

Supplement: S1 Fig — (TIF) [file pone.0180858.s001.tif]

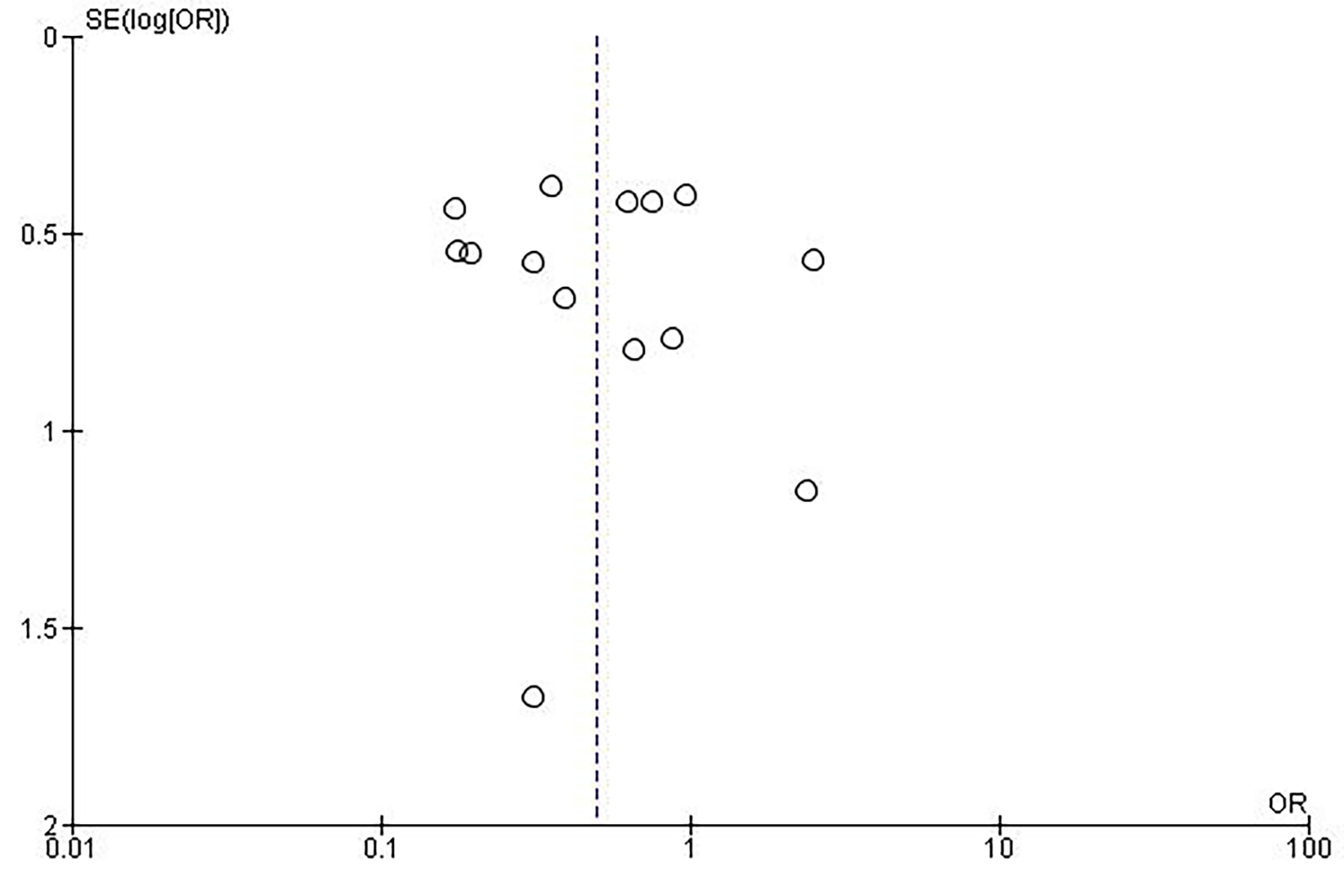

Supplement: S2 Fig — (TIF) [file pone.0180858.s002.TIF]
