# Supplementary material for: Decreasing rates of disorganised attachment in infants and young children, who are at risk of developing, or who already have disorganised attachment. A systematic review and meta-analysis of early parenting interventions
Source: PLoS One. 2017 Jul 14;12(7):e0180858. doi: 10.1371/journal.pone.0180858 (PMC5510823; doi:10.1371/journal.pone.0180858)
Supplement: S1 File — Wright, W., Barry, M., Hughes, E., Trepel, D., Ali, S., Allgar, V., Cottrill, L., Duffy, S., Fell, J., Gilbody, S., Glanville, J., Glaser, D., Hackney, L., Manea1, L., McMillan, D., Palmer, S., Prior, V., Whitton, C., Perry, A. Clinical and cost effectiveness of parenting interventions for children with ‘severe attachment problems’: A systematic review and meta-analysis. Health Technology Assessment. 2015; 19: 1–348. (PDF) [file pone.0180858.s005.pdf]

HEALTH TECHNOLOGY ASSESSMENT

VOLUME 19 ISSUE 52 JULY 2015
ISSN 1366-5278

Clinical effectiveness and cost-effectiveness of parenting interventions for children with severe attachment problems: a systematic review and meta-analysis

Barry Wright, Melissa Barry, Ellen Hughes, Dominic Trépel, Shehzad Ali, Victoria Allgar, Lucy Cottrill, Steven Duffy, Jenny Fell, Julie Glanville, Danya Glaser, Lisa Hackney, Laura Manea, Dean McMillan, Stephen Palmer, Vivien Prior, Clare Whitton, Amanda Perry and Simon Gilbody

NHS
National Institute for Health Research

DOI 10.3310/hta19520

Clinical effectiveness and cost-effectiveness of parenting interventions for children with severe attachment problems: a systematic review and meta-analysis

Barry Wright,[1,2*] Melissa Barry,[2] Ellen Hughes,[2] Dominic Trépel,[3] Shehzad Ali,[3] Victoria Allgar,[1] Lucy Cottrill,[2] Steven Duffy,[4] Jenny Fell,[2,3] Julie Glanville,[4] Danya Glaser,[5,6] Lisa Hackney,[2] Laura Manea,[1] Dean McMillan,[1,3] Stephen Palmer,[7] Vivien Prior,[8] Clare Whitton,[9] Amanda Perry[3] and Simon Gilbody[1,3]

[1]Hull York Medical School, University of York, York, UK
[2]Leeds and York Partnership NHS Foundation Trust, Leeds, UK
[3]Department of Health Sciences, University of York, York, UK
[4]York Health Economics Consortium, York, UK
[5]Great Ormond Street Hospital, London, UK
[6]Research Department of Clinical, Educational and Health Psychology, University College London, London, UK
[7]Centre for Health Economics, University of York, York, UK
[8]Faculty of Population Health Sciences, Institute of Child Health, University College London, London, UK
[9]Tees, Esk and Wear Valleys NHS Foundation Trust, Middlesbrough, UK

*Corresponding author

Declared competing interests of authors: none

Published July 2015
DOI: 10.3310/hta19520

This report should be referenced as follows:

Wright B, Barry M, Hughes E, Trépel D, Ali S, Allgar V, *et al*. Clinical effectiveness and cost-effectiveness of parenting interventions for children with severe attachment problems: a systematic review and meta-analysis. *Health Technol Assess* 2015;**19**(52).

Health Technology Assessment is indexed and abstracted in *Index Medicus*/MEDLINE, *Excerpta Medica*/EMBASE, *Science Citation Index Expanded* (SciSearch®) and *Current Contents*®/ Clinical Medicine.

Health Technology Assessment

HTA/HTA TAR

ISSN 1366-5278 (Print)

ISSN 2046-4924 (Online)

Impact factor: 5.116

Health Technology Assessment is indexed in MEDLINE, CINAHL, EMBASE, The Cochrane Library and the ISI Science Citation Index.

This journal is a member of and subscribes to the principles of the Committee on Publication Ethics (COPE) (www.publicationethics.org/).

Editorial contact: nihredit@southampton.ac.uk

The full HTA archive is freely available to view online at www.journalslibrary.nihr.ac.uk/hta. Print-on-demand copies can be purchased from the report pages of the NIHR Journals Library website: www.journalslibrary.nihr.ac.uk

Criteria for inclusion in the *Health Technology Assessment* journal

Reports are published in *Health Technology Assessment* (HTA) if (1) they have resulted from work for the HTA programme, and (2) they are of a sufficiently high scientific quality as assessed by the reviewers and editors.

Reviews in *Health Technology Assessment* are termed 'systematic' when the account of the search appraisal and synthesis methods (to minimise biases and random errors) would, in theory, permit the replication of the review by others.

HTA programme

The HTA programme, part of the National Institute for Health Research (NIHR), was set up in 1993. It produces high-quality research information on the effectiveness, costs and broader impact of health technologies for those who use, manage and provide care in the NHS. 'Health technologies' are broadly defined as all interventions used to promote health, prevent and treat disease, and improve rehabilitation and long-term care.

The journal is indexed in NHS Evidence via its abstracts included in MEDLINE and its Technology Assessment Reports inform National Institute for Health and Care Excellence (NICE) guidance. HTA research is also an important source of evidence for National Screening Committee (NSC) policy decisions.

For more information about the HTA programme please visit the website: http://www.nets.nihr.ac.uk/programmes/hta

This report

The research reported in this issue of the journal was funded by the HTA programme as project number 10/45/04. The contractual start date was in October 2011. The draft report began editorial review in October 2013 and was accepted for publication in May 2014. The authors have been wholly responsible for all data collection, analysis and interpretation, and for writing up their work. The HTA editors and publisher have tried to ensure the accuracy of the authors' report and would like to thank the reviewers for their constructive comments on the draft document. However, they do not accept liability for damages or losses arising from material published in this report.

This report presents independent research funded by the National Institute for Health Research (NIHR). The views and opinions expressed by authors in this publication are those of the authors and do not necessarily reflect those of the NHS, the NIHR, NETSCC, the HTA programme or the Department of Health. If there are verbatim quotations included in this publication the views and opinions expressed by the interviewees are those of the interviewees and do not necessarily reflect those of the authors, those of the NHS, the NIHR, NETSCC, the HTA programme or the Department of Health.

© Queen's Printer and Controller of HMSO 2015. This work was produced by Wright *et al.* under the terms of a commissioning contract issued by the Secretary of State for Health. This issue may be freely reproduced for the purposes of private research and study and extracts (or indeed, the full report) may be included in professional journals provided that suitable acknowledgement is made and the reproduction is not associated with any form of advertising. Applications for commercial reproduction should be addressed to: NIHR Journals Library, National Institute for Health Research, Evaluation, Trials and Studies Coordinating Centre, Alpha House, University of Southampton Science Park, Southampton SO16 7NS, UK.

Published by the NIHR Journals Library (www.journalslibrary.nihr.ac.uk), produced by Prepress Projects Ltd, Perth, Scotland (www.prepress-projects.co.uk).

Editor-in-Chief of *Health Technology Assessment* and NIHR Journals Library

Professor Tom Walley Director, NIHR Evaluation, Trials and Studies and Director of the HTA Programme, UK

NIHR Journals Library Editors

Professor Ken Stein Chair of HTA Editorial Board and Professor of Public Health, University of Exeter Medical School, UK

Professor Andree Le May Chair of NIHR Journals Library Editorial Group (EME, HS&DR, PGfAR, PHR journals)

Dr Martin Ashton-Key Consultant in Public Health Medicine/Consultant Advisor, NETSCC, UK

Professor Matthias Beck Chair in Public Sector Management and Subject Leader (Management Group), Queen's University Management School, Queen's University Belfast, UK

Professor Aileen Clarke Professor of Public Health and Health Services Research, Warwick Medical School, University of Warwick, UK

Dr Tessa Crilly Director, Crystal Blue Consulting Ltd, UK

Dr Peter Davidson Director of NETSCC, HTA, UK

Ms Tara Lamont Scientific Advisor, NETSCC, UK

Professor Elaine McColl Director, Newcastle Clinical Trials Unit, Institute of Health and Society, Newcastle University, UK

Professor William McGuire Professor of Child Health, Hull York Medical School, University of York, UK

Professor Geoffrey Meads Professor of Health Sciences Research, Faculty of Education, University of Winchester, UK

Professor John Powell Consultant Clinical Adviser, National Institute for Health and Care Excellence (NICE), UK

Professor James Raftery Professor of Health Technology Assessment, Wessex Institute, Faculty of Medicine, University of Southampton, UK

Dr Rob Riemsma Reviews Manager, Kleijnen Systematic Reviews Ltd, UK

Professor Helen Roberts Professor of Child Health Research, UCL Institute of Child Health, UK

Professor Helen Snooks Professor of Health Services Research, Institute of Life Science, College of Medicine, Swansea University, UK

Please visit the website for a list of members of the NIHR Journals Library Board: www.journalslibrary.nihr.ac.uk/about/editors

Editorial contact: nihredit@southampton.ac.uk

Abstract

Clinical effectiveness and cost-effectiveness of parenting interventions for children with severe attachment problems: a systematic review and meta-analysis

Barry Wright,[1,2*] Melissa Barry,[2] Ellen Hughes,[2] Dominic Trépel,[3] Shehzad Ali,[3] Victoria Allgar,[1] Lucy Cottrill,[2] Steven Duffy,[4] Jenny Fell,[2,3] Julie Glanville,[4] Danya Glaser,[5,6] Lisa Hackney,[2] Laura Manea,[1] Dean McMillan,[1,3] Stephen Palmer,[7] Vivien Prior,[8] Clare Whitton,[9] Amanda Perry[3] and Simon Gilbody[1,3]

[1]Hull York Medical School, University of York, York, UK
[2]Leeds and York Partnership NHS Foundation Trust, Leeds, UK
[3]Department of Health Sciences, University of York, York, UK
[4]York Health Economics Consortium, York, UK
[5]Great Ormond Street Hospital, London, UK
[6]Research Department of Clinical, Educational and Health Psychology, University College London, London, UK
[7]Centre for Health Economics, University of York, York, UK
[8]Faculty of Population Health Sciences, Institute of Child Health, University College London, London, UK
[9]Tees, Esk and Wear Valleys NHS Foundation Trust, Middlesbrough, UK

*Corresponding author barry.wright1@nhs.net

Background and objectives: Services have variable practices for identifying and providing interventions for 'severe attachment problems' (disorganised attachment patterns and attachment disorders). Several government reports have highlighted the need for better parenting interventions in at-risk groups. This report was commissioned to evaluate the clinical effectiveness and cost-effectiveness of parenting interventions for children with severe attachment problems (the main review). One supplementary review explored the evaluation of assessment tools and a second reviewed 10-year outcome data to better inform health economic aspects of the main review.

Data sources: A total of 29 electronic databases were searched with additional mechanisms for identifying a wide pool of references using the Cochrane methodology. Examples of databases searched include PsycINFO (1806 to January week 1, 2012), MEDLINE and MEDLINE In-Process & Other Non-Indexed Citations (1946 to December week 4, 2011) and EMBASE (1974 to week 1, 2012). Searches were carried out between 6 and 12 January 2012.

Review methods: Papers identified were screened and data were extracted by two independent reviewers, with disagreements arbitrated by a third independent reviewer. Quality assessment tools were used, including quality assessment of diagnostic accuracy studies – version 2 and the Cochrane risk of bias tool. Meta-analysis of randomised controlled trials (RCTs) of parenting interventions was undertaken. A health economics analysis was conducted.

© Queen's Printer and Controller of HMSO 2015. This work was produced by Wright *et al.* under the terms of a commissioning contract issued by the Secretary of State for Health. This issue may be freely reproduced for the purposes of private research and study and extracts (or indeed, the full report) may be included in professional journals provided that suitable acknowledgement is made and the reproduction is not associated with any form of advertising. Applications for commercial reproduction should be addressed to: NIHR Journals Library, National Institute for Health Research, Evaluation, Trials and Studies Coordinating Centre, Alpha House, University of Southampton Science Park, Southampton SO16 7NS, UK.

Results: The initial search returned 10,167 citations. This yielded 29 RCTs in the main review of parenting interventions to improve attachment patterns, and one involving children with reactive attachment disorder. A meta-analysis of eight studies seeking to improve outcome in at-risk populations showed statistically significant improvement in disorganised attachment. The interventions saw less disorganised attachment at outcome than the control (odds ratio 0.47, 95% confidence interval 0.34 to 0.65; $p < 0.00001$). Much of this focused around interventions improving maternal sensitivity, with or without video feedback. In our first supplementary review, 35 papers evaluated an attachment assessment tool demonstrating validity or psychometric data. Only five reported test–retest data. Twenty-six studies reported inter-rater reliability, with 24 reporting a level of 0.7 or above. Cronbach's alphas were reported in 12 studies for the comparative tests (11 with $\alpha > 0.7$) and four studies for the reference tests (four with $\alpha > 0.7$). Three carried out concurrent validity comparing the Strange Situation Procedure (SSP) with another assessment tool. These had good sensitivity but poor specificity. The Disturbances of Attachment Interview had good sensitivity and specificity with the research diagnostic criteria (RDC) for attachment disorders. In our supplementary review of 10-year outcomes in cohorts using a baseline reference standard, two studies were found with disorganised attachment at baseline, with one finding raised psychopathology in adolescence. Budget impact analysis of costs was estimated because a decision model could not be justifiably populated. This, alongside other findings, informed research priorities.

Limitations: There are relatively few UK-based clinical trials. A 10-year follow-up, while necessary for our health economists for long-term sequelae, yielded a limited number of papers.

Conclusions: Maternal sensitivity interventions show good outcomes in at-risk populations, but require further research with complex children. The SSP and RDC for attachment disorders remain the reference standards for identification until more concurrent and predictive validity research is conducted. A birth cohort with sequential attachment measures and outcomes across different domains is recommended with further, methodologically sound randomised controlled intervention trials. The main area identified for future work was a need for good-quality RCTs in at-risk groups such as those entering foster care or adoption.

Study registration: This study is registered as PROSPERO CRD42011001395.

Funding: The National Institute for Health Research Health Technology Assessment programme.

Contents

List of tables xiii

List of figures xv

List of boxes xix

List of abbreviations xxi

Plain English summary xxiii

Scientific summary xxv

Chapter 1 Background **1**
What is attachment? 1
Attachment patterns and their antecedents 1
Natural history 2
Stability 2
Evolution within disorganised pattern 2
Change of assessed manifestation of attachment 2
The significance of attachment and its relationship to psychopathology 3
Tools for assessing attachment patterns 4
Assessment of attachment behaviour 4
Representations of attachment 5
Coherence of accounts 5
Attachment disorders 5
Is there a gold standard for measuring attachment? 6
Interventions for attachment problems (disorganised attachment patterns and attachment disorders) 6
Policy and practice 7
Purpose of the present review 8

Chapter 2 Aims, objectives and scope **9**
Aims 9
Current review definition 9
Scope of the review 9
Objectives 11
Description of the decision problem for the purposes of health economic analysis 11
How do we identify those who will benefit from interventions? 11
Who is at risk and who is it that we should be treating? 12
Which parenting interventions work and are they cost-effective? 12
Overview of process 12

Chapter 3 Literature search **13**
Search strategy 13
Search terms 14
Screening of citations 15

© Queen's Printer and Controller of HMSO 2015. This work was produced by Wright *et al.* under the terms of a commissioning contract issued by the Secretary of State for Health. This issue may be freely reproduced for the purposes of private research and study and extracts (or indeed, the full report) may be included in professional journals provided that suitable acknowledgement is made and the reproduction is not associated with any form of advertising. Applications for commercial reproduction should be addressed to: NIHR Journals Library, National Institute for Health Research, Evaluation, Trials and Studies Coordinating Centre, Alpha House, University of Southampton Science Park, Southampton SO16 7NS, UK.

Inclusion and exclusion criteria 15
Additional search strategies 15
Stakeholder involvement 15

Chapter 4 Supplementary systematic review 1: validity of methods to identify attachment patterns and disorders **17**
Introduction 17
Methods 18
Inclusion criteria 18
Data extraction 18
Diagnostic accuracy 18
Quality assessment strategy 19
Data synthesis 19
Results 19
Study characteristics 20
Population characteristics 35
Quality assessment 36
Taxonomy of instruments assessing attachment patterns 41
Taxonomy of assessment for attachment disorder 45
Psychometric properties 45
Sensitivity and specificity analysis 61
Discussion and summary of findings 63

Chapter 5 Supplementary systematic review 2: studies of severe attachment problems with a follow-up of 10 years or longer **65**
Introduction 65
Methods 65
Inclusion criteria 66
Data extraction 66
Quality assessment 66
Data synthesis 67
Results 68
Study and sample characteristics and prevalence data 68
Population characteristics 72
Rates of disorganised attachment in the various papers reviewed 72
Quality assessment 73
Long-term outcomes 75
Conclusion 77

Chapter 6 Main systematic review: early parenting interventions for families of children with severe attachment problems **79**
Introduction 79
Methods 79
Inclusion criteria 79
Conducting the quality assessment 80
Data extraction 80
Demographic information 80
Data synthesis 80
Meta-analysis 81

Results 81
Characteristics of included intervention studies 83
Specific interventions 106
Interventions which were not included 110
Quality assessment 111
Meta-analyses findings 115

Chapter 7 Economic evaluation of parenting interventions for severe attachment problems **129**
Introduction 129
Section 1: systematic review of existing cost-effectiveness evidence 129
Methods 129
Results 130
Discussion 131
Section 2: economic evaluation of severe attachment problems 132
Natural history of severe attachment problems 133
Methods for developing a theoretical modelling framework 134
Conclusions 154

Chapter 8 Research priorities and value of information analysis **157**
Gaps and limitations identified 157
Consensus on meaning of severe attachment problems 157
Measures and identification of children with severe attachment problems 157
Information on severe attachment problems within specific subpopulations 158
Prevalence of severe attachment problems 158
Long-term health outcomes 159
Intervention effects 159
Resource and cost-effectiveness information 160
Value of information analysis and informing future research priorities 160
Description of future research priorities 160
Priority 1: recommendations for clarifying the meaning of 'severe attachment problems' and developing consensus on assessment tools to be used 160
Priority 2: recommendation for prevalence and long-term outcome studies 161
Priority 3: randomised controlled trials of interventions (clinical effectiveness and cost-effectiveness) 162
Summary 164

Chapter 9 Discussion and conclusion **165**
Statement of principal findings 165
Objective 1 165
Objective 2 165
Objective 3 165
Objective 4 165
Objective 5 (supplementary review 1) 165
Objective 6 (supplementary review 2) 166
Limitations 166
Implications for practice 168
Implications for research 169

Acknowledgements **171**

References **173**

© Queen's Printer and Controller of HMSO 2015. This work was produced by Wright *et al.* under the terms of a commissioning contract issued by the Secretary of State for Health. This issue may be freely reproduced for the purposes of private research and study and extracts (or indeed, the full report) may be included in professional journals provided that suitable acknowledgement is made and the reproduction is not associated with any form of advertising. Applications for commercial reproduction should be addressed to: NIHR Journals Library, National Institute for Health Research, Evaluation, Trials and Studies Coordinating Centre, Alpha House, University of Southampton Science Park, Southampton SO16 7NS, UK.

Appendix 1 Full search strategy **191**

Appendix 2 Stakeholder involvement **259**

Appendix 3 Quality assessment tool for cohort studies **261**

Appendix 4 Additional searches for 5- to 10-year outcomes for children with disorganised attachment at baseline **263**

Appendix 5 List of excluded studies, with reasons **265**

Appendix 6 Meta-analysis of studies seeking to establish secure attachment patterns **317**

Appendix 7 Cochrane risk of bias tool **341**

Appendix 8 The Quality assessment of diagnostic accuracy studies – version 2 **345**

List of tables

TABLE 1 Overview of screening and assessment studies and instrument characteristics 21

TABLE 2 Taxonomy of screening tools for attachment patterns 32

TABLE 3 Taxonomy of screening tools for attachment disorders 33

TABLE 4 Summary of quality assessment for accuracy tool/diagnostic accuracy studies 37

TABLE 5 Ratings on applicability questions of QUADAS-2 for studies included in supplementary review 1 40

TABLE 6 Psychometric properties of the index and reference test(s) 46

TABLE 7 Performance of attachment procedure measures: concurrent validity as compared with reference standard 61

TABLE 8 Performance of instruments assessing attachment disorders: concurrent validity as compared with reference standard 61

TABLE 9 Criteria and domains examined by the bespoke quality assessment tool 67

TABLE 10 Study, sample and prevalence characteristics of included studies 69

TABLE 11 Table illustrating the quality assessment of included studies 74

TABLE 12 Summary of findings of attachment stability as a long-term outcome 75

TABLE 13 Summary of findings for mental illness as a long-term outcome 76

TABLE 14 Study and intervention characteristics: participants without a disorganised classification or diagnosis of RAD 84

TABLE 15 Study and intervention characteristics: participants with a disorganised classification or diagnosis of RAD 100

TABLE 16 Cochrane quality assessment checklist for studies without a disorganised category or a diagnosis of RAD/DAD 112

TABLE 17 Cochrane quality assessment table for studies that contained a disorganised category or a diagnosis of RAD/DAD 114

TABLE 18 Identified studies of attachment containing cost–consequence analysis 130

TABLE 19 Type of severe attachment problem, sample population in which prevalence was measured and the measures of prevalence (mean, minimum and maximum) from the available literature 140

© Queen's Printer and Controller of HMSO 2015. This work was produced by Wright *et al.* under the terms of a commissioning contract issued by the Secretary of State for Health. This issue may be freely reproduced for the purposes of private research and study and extracts (or indeed, the full report) may be included in professional journals provided that suitable acknowledgement is made and the reproduction is not associated with any form of advertising. Applications for commercial reproduction should be addressed to: NIHR Journals Library, National Institute for Health Research, Evaluation, Trials and Studies Coordinating Centre, Alpha House, University of Southampton Science Park, Southampton SO16 7NS, UK.

TABLE 20 Identification strategy, resources required and the estimated cost to the NHS for identifying disorganised patterns of attachment 145

TABLE 21 Identification strategy, resources required and the estimated cost to the NHS for identifying an attachment disorder 145

TABLE 22 Intervention for improving or preventing disorganised attachment, required resource inputs (type of personnel performing the intervention, duration of each session and total number of sessions) and the estimated cost to the NHS for the treatment 146

TABLE 23 Treatment of RADs, required resource inputs (type of personnel performing the intervention, duration of each session and total number of sessions) and the estimated cost to the NHS for the treatment 147

TABLE 24 Required information to inform a decision model, the quality of evidence currently identified and specific details of each category 148

TABLE 25 Budget impact assessment of identification strategies to detect disorganised patterns of attachment 150

TABLE 26 Budget impact assessment of identification strategies to detect attachment disorders 152

TABLE 27 Budget impact assessment of the cost of treating disorganised patterns of attachment by target populations 153

TABLE 28 Budget impact assessment of the cost of treating RAD in general populations 154

TABLE 29 Summary of findings of attachment stability (or other attachment-related outcomes) between 5 and 10 years 263

TABLE 30 Summary of findings for psychosocial and developmental 5- to 10-year outcomes 264

List of figures

FIGURE 1 Severe attachment problems definition as defined by our review 10

FIGURE 2 A PRISMA diagram illustrating the results of the screening process in the supplementary review 20

FIGURE 3 Summary of study populations from studies included in supplementary review 1 36

FIGURE 4 Preferred Reporting Items for Systematic Reviews and Meta-Analyses flow chart of papers included in supplementary review 2 68

FIGURE 5 Preferred Reporting Items for Systematic Reviews and Meta-Analyses for the main systematic review 82

FIGURE 6 Study population characteristics for studies with and without a disorganised attachment pattern (D) or a diagnosis of RAD/DAD 106

FIGURE 7 Funnel plot of papers including a disorganised classification or a diagnosis of RAD 115

FIGURE 8 A meta-analysis of changes in disorganised outcomes, comparing parenting intervention and a control condition 117

FIGURE 9 Changes in disorganised outcomes, comparing duration of intervention 118

FIGURE 10 Changes to disorganised outcomes, comparing length of follow-up (< 12 months/≥ 12 months) 119

FIGURE 11 A meta-analysis of changes in disorganised outcomes, comparing number of sessions (< 5, 5–16, > 16) 120

FIGURE 12 A meta-analysis of changes to disorganised outcomes, comparing age of child at start (prenatal/≤ 6 months old/> 6 months old) 121

FIGURE 13 A meta-analysis of changes in disorganised outcomes, comparing caregivers with and without foster children 122

FIGURE 14 A meta-analysis of disorganised outcomes, comparing intervention locations (home, mixed, other) 123

FIGURE 15 A meta-analysis of changes in disorganised attachment patterns, comparing inclusion of male caregiver alongside female caregiver with exclusion of male caregiver 124

FIGURE 16 A meta-analysis of changes to disorganised outcomes, comparing interventions that provided video feedback with those that did not 125

© Queen's Printer and Controller of HMSO 2015. This work was produced by Wright *et al.* under the terms of a commissioning contract issued by the Secretary of State for Health. This issue may be freely reproduced for the purposes of private research and study and extracts (or indeed, the full report) may be included in professional journals provided that suitable acknowledgement is made and the reproduction is not associated with any form of advertising. Applications for commercial reproduction should be addressed to: NIHR Journals Library, National Institute for Health Research, Evaluation, Trials and Studies Coordinating Centre, Alpha House, University of Southampton Science Park, Southampton SO16 7NS, UK.

FIGURE 17 A meta-analysis of changes to disorganised outcomes, looking at whether or not the intervention attempts to enhance maternal sensitivity 126

FIGURE 18 A meta-analysis of changes to disorganised outcomes, comparing types of intervention involved (caregiver and child together, caregiver and child separately, caregiver alone or mixed) 127

FIGURE 19 How the health-care and non-health pathways to cost and outcomes indicate health-care and wider perspectives 132

FIGURE 20 Theoretical framework of an identification model for severe attachment problems in specific population of children [A = 1/0 (child with/without severe attachment problem); D = 1/0 (screen positive/screen negative)] 135

FIGURE 21 Theoretical framework of the decision model of interventions/treatments for severe attachment problems 136

FIGURE 22 Health mediated pathway from severe attachment problem post-treatment effects via longer-term mediators (such as child and adult levels of psychopathology) onto health and non-health outcomes 142

FIGURE 23 Mediating pathway of developmental sequelae from the post-treatment effects via long-term mediators (such as IQ, educational attainment and criminality) onto health and non-health outcomes 144

FIGURE 24 Forest plot for secure outcomes for all 17 interventions in the included studies 318

FIGURE 25 A meta-analysis of changes in secure outcomes comparing parental intervention to a control condition 319

FIGURE 26 A meta-analysis of changes in secure outcomes comparing duration of intervention (< 12 months, ≥ 12 months) 321

FIGURE 27 A meta-analysis of changes in secure outcomes comparing different lengths of follow-up (< 12 months, ≥ 12 months) 323

FIGURE 28 A meta-analysis of changes in secure outcomes comparing the number of sessions (< 5, 5–16, > 16) 325

FIGURE 29 A meta-analysis of changes in secure outcomes comparing age of child at start (≤ 6 months, > 6 months) 327

FIGURE 30 A meta-analysis of changes in secure outcomes comparing middle-class families and other family types 329

FIGURE 31 A meta-analysis of changes in secure outcomes comparing whether or not the male caregiver was included alongside the female caregiver in the intervention 331

FIGURE 32 A meta-analysis of changes in secure outcomes comparing whether or not video feedback was provided 333

FIGURE 33 A meta-analysis of changes in secure outcomes comparing whether or not the intervention attempts to enhance maternal sensitivity 335

FIGURE 34 A meta-analysis of changes to secure outcomes comparing whether or not the primary focus of the intervention was to modify child attachment 337

© Queen's Printer and Controller of HMSO 2015. This work was produced by Wright *et al.* under the terms of a commissioning contract issued by the Secretary of State for Health. This issue may be freely reproduced for the purposes of private research and study and extracts (or indeed, the full report) may be included in professional journals provided that suitable acknowledgement is made and the reproduction is not associated with any form of advertising. Applications for commercial reproduction should be addressed to: NIHR Journals Library, National Institute for Health Research, Evaluation, Trials and Studies Coordinating Centre, Alpha House, University of Southampton Science Park, Southampton SO16 7NS, UK.

List of boxes

BOX 1 Coding classification key for the SSP and modifications 34

© Queen's Printer and Controller of HMSO 2015. This work was produced by Wright *et al.* under the terms of a commissioning contract issued by the Secretary of State for Health. This issue may be freely reproduced for the purposes of private research and study and extracts (or indeed, the full report) may be included in professional journals provided that suitable acknowledgement is made and the reproduction is not associated with any form of advertising. Applications for commercial reproduction should be addressed to: NIHR Journals Library, National Institute for Health Research, Evaluation, Trials and Studies Coordinating Centre, Alpha House, University of Southampton Science Park, Southampton SO16 7NS, UK.

List of abbreviations

AAI	Adult Attachment Interview
ABC	Attachment and Biobehavioural Catch-up
ADI	Attachment Doll Interview
APA	American Psychiatric Association
AQS	attachment Q-set
BAT	Biopsychosocial Attachment Types
BERS	Behavioural and Emotional Rating Scale
CAI	Child Attachment Interview
CAMHS	Child and Adolescent Mental Health Service
CAP	California Attachment Procedure
CAPA	Child and Adolescent Psychiatric Assessment
CBRS	May-Nichols Child Behaviour Rating Scale
CDSR	Cochrane Database of Systematic Reviews
CENTRAL	Cochrane Central Register of Controlled Trials
CI	confidence interval
CMCAST	Computerised Manchester Child Attachment Story Task
CONSORT	Consolidated Standards of Reporting Trials
COS-4	Circle of Security – home visiting-4
CRD	Centre for Reviews and Dissemination
DAD	disinhibited attachment disorder
DAI	Disturbances of Attachment Interview
DARE	Database of Abstracts of Reviews of Effects
DC: 0–3R	Revised Classification of Mental Health and Developmental Disorders of Infancy and Early Childhood
DMM	Dynamic Maturational Model
DSM-IV	*Diagnostic and Statistical Manual of Mental Disorders*-Fourth Edition
DSM-V	*Diagnostic and Statistical Manual of Mental Disorders*-Fifth Edition
EHS	Early Head Start
HCP	Healthy Child Programme
HEED	Health Economic Evaluations Database
HTA	Health Technology Assessment
IAPT	Increasing Access to Psychological Therapies
ICD-10	*International Classification of Diseases*, Tenth Edition
ICD-11	*International Classification of Diseases*, Eleventh Edition
IMAS	Interview Measure of Attachment Security
IPT	interpersonal psychotherapy
IQ	intellectual quotient
LTS	Louisville Twin Study
M–H	Mantel–Haenszel
MCAST	Manchester Child Attachment Story Task
MCDC	Middle Childhood Disorganisation and Control
MeSH	medical subject headings
MIMRS	Marschak Interaction Method Rating System
MSSB	MacArthur Story Stem Battery

© Queen's Printer and Controller of HMSO 2015. This work was produced by Wright *et al.* under the terms of a commissioning contract issued by the Secretary of State for Health. This issue may be freely reproduced for the purposes of private research and study and extracts (or indeed, the full report) may be included in professional journals provided that suitable acknowledgement is made and the reproduction is not associated with any form of advertising. Applications for commercial reproduction should be addressed to: NIHR Journals Library, National Institute for Health Research, Evaluation, Trials and Studies Coordinating Centre, Alpha House, University of Southampton Science Park, Southampton SO16 7NS, UK.

MTFC-P	Multidimensional Treatment Foster Care Program for Pre-schoolers
NHS EED	NHS Economic Evaluation Database
NICE	National Institute for Health and Care Excellence
NIHR	National Institute for Health Research
OR	odds ratio
PAA	Preschool Assessment of Attachment
PAPA	Preschool Age Psychiatric Assessment
PICOS	participants, interventions, comparisons, outcomes, study design
PPI	patient and public involvement
PPIP	Preventative Psychotherapeutic Intervention Program
PRISMA	Preferred Reporting Items for Systematic Reviews and Meta-Analyses
PSSRU	Personal Social Services Research Unit
QALY	quality-adjusted life-year
QUADAS-2	quality assessment of diagnostic accuracy studies – version 2
RAD	reactive attachment disorder
RADQ	Randolph Attachment Disorder Questionnaire
RCT	randomised controlled trial
RDC	research diagnostic criteria
RPQ	Relationships Problems Questionnaire
SAT	Separation Anxiety Test
SD	standard deviation
SES	socioeconomic status
SSAP	Story Stem Assessment Profile
SSP	Strange Situation Procedure
TPP	Toddler–Parent Psychotherapy
UCLA FDP	University of California, Los Angeles Family Development Project
VIPP-R	Video-feedback Intervention to promote Positive Parenting with a Representational focus
WHO	World Health Organization
WRO	Waiting Room Observation

Plain English summary

This review was commissioned to find parental interventions shown in research to improve or prevent severe attachment problems. We found 10,167 research papers on attachment and three teams of reviewers gathered information for the following reviews.

The main systematic review identified 39 parental intervention papers meeting set criteria. Eight interventions for reducing disorganised attachment were meta-analysed and showed good outcomes overall. Most of these focused on improving parental sensitivity. Two papers included an economic evaluation of interventions with data of limited usefulness. A health economics analysis is provided.

Supplementary review 1 explored good ways of assessing attachment at baseline and showed a diverse literature. We identified 35 papers meeting set criteria that examined the development of an assessment tool for attachment patterns or attachment disorders. Scientific measures of their reliability and validity were varied. The two assessment tools compared with the Strange Situation Procedure at the same time had a tendency to identify high rates of false positives and would not be useful clinically. In supplementary review 2, we found eight studies that used a valid disorganised attachment measure or a valid measure of attachment disorder at baseline and followed the children up for at least 10 years. There was an association with emotional and behavioural problems by age 17 years and a weak correlation with personality disorder in young adults.

Helpful future research would include improved study designs and a well-planned large birth cohort study with long-term follow-up and a range of assessments and outcome measures. More intervention research would be productive.

© Queen's Printer and Controller of HMSO 2015. This work was produced by Wright *et al.* under the terms of a commissioning contract issued by the Secretary of State for Health. This issue may be freely reproduced for the purposes of private research and study and extracts (or indeed, the full report) may be included in professional journals provided that suitable acknowledgement is made and the reproduction is not associated with any form of advertising. Applications for commercial reproduction should be addressed to: NIHR Journals Library, National Institute for Health Research, Evaluation, Trials and Studies Coordinating Centre, Alpha House, University of Southampton Science Park, Southampton SO16 7NS, UK.

Scientific summary

Background

The concept of attachment describes the child's component of the caregiving bond between the infant and the primary caregiver, usually the mother. It allows the developing infant to explore the environment safely, to elicit care from a caregiver (seeking proximity during times of threat) and to learn how to cope with the challenges and anxieties presented in the environment. Various models and hypotheses suggest the importance of its influence on development.

Attachment is traditionally measured in two ways. The first involves identifying patterns of attachment (sometimes referred to as styles or attachment organisation). This involves coding the responses of an infant (usually aged between 9 and 18 months) to a series of encounters involving his or her primary caregiver and a stranger. The reference standard for this is the Strange Situation Procedure (SSP). This is not a diagnosis but a set of observed behaviours that present in one of a number of patterns. The second involves research diagnostic criteria (RDC) for attachment disorders, specified by both the American Psychatric Association (APA) and the World Health Organization (WHO). The relationship between attachment patterns and attachment disorders is unclear. There is a wide narrative literature describing the importance of attachment problems in the developmental course of children. This has led to numerous interventions being developed in an attempt to improve attachment and reduce negative outcomes. This review has been requested in order to elucidate the literature on the clinical effectiveness and cost-effectiveness of parenting interventions for children with severe attachment problems. This will inform best practice by those delivering these programmes. After extensive discussion with the expert/patient and public involvement group, we defined severe attachment problems [a term coined by the National Institute for Health Research (NIHR) call] as disorganised attachment patterns, or attachment disorders in children as diagnosed by the WHO or the APA classification system.

Objectives

The main objective specified in the Health Technology Assessment (HTA) programme call was to address the question 'What is the effectiveness and cost-effectiveness of early parenting interventions for parents whose children show signs of developing severe attachment problems?'.

To achieve this we specified our main review objectives as follows:

1. to identify the range of intervention programmes that are designed for parents of children with severe attachment problems
2. to examine the clinical effectiveness of intervention programmes designed for parents of children with severe attachment problems
3. to examine the cost-effectiveness of intervention programmes designed for parents of children with severe attachment problems
4. to identify research priorities for developing future intervention programmes for children with severe attachment disorders, from the perspective of the UK NHS.

These four objectives formed the basis of the main systematic review.

© Queen's Printer and Controller of HMSO 2015. This work was produced by Wright *et al.* under the terms of a commissioning contract issued by the Secretary of State for Health. This issue may be freely reproduced for the purposes of private research and study and extracts (or indeed, the full report) may be included in professional journals provided that suitable acknowledgement is made and the reproduction is not associated with any form of advertising. Applications for commercial reproduction should be addressed to: NIHR Journals Library, National Institute for Health Research, Evaluation, Trials and Studies Coordinating Centre, Alpha House, University of Southampton Science Park, Southampton SO16 7NS, UK.

We undertook supplementary reviews in order to support this work. These specifically focused on developing clarity around baseline measures of attachment and obtaining additional information about outcomes over 10 years, as most of the effectiveness studies reported outcomes that were short term. This was to provide additional information for the health economists. These objectives were as follows:

1. to review the methods of assessment and/or diagnosis of attachment patterns and/or disorders (supplementary systematic review 1)
2. to examine the 10-year or more outcomes among children with severe attachment problems and collect prevalence information from these studies (supplementary systematic review 2).

Methods

A literature search was undertaken across 29 electronic databases and 11 internet sites. Examples of databases searched include PsycINFO (1806 to January week 1, 2012), MEDLINE and MEDLINE In-Process & Other Non-Indexed Citations (1946 to December week 4, 2011) and EMBASE (1974 to week 1, 2012). Information was gathered by personal communication and authors' contact details, and by identifying additional references through bibliographic lists. The systematic review was divided into one main review and two supplementary reviews as described above, and utilised different screening criteria and data extraction information for each stage of the review. Methods outlined by the Centre for Reviews and Dissemination and Cochrane were followed. For the main systematic review of clinical effectiveness and cost-effectiveness, we evaluated randomised controlled trial (RCT) evidence to assess the effectiveness of interventions to improve attachment patterns or attachment disorders. The Cochrane risk of bias tool was used to carry out quality assessment. We set out to gather enough information to populate a health economics decision model and carry out a value of information analysis. The first supplementary review considered the diagnostic accuracy of screening and assessment tools used to identify attachment patterns and attachment disorder. Quality assessments were conducted using the quality assessment of diagnostic accuracy studies – version 2. The review of 10-year outcomes (supplementary review 2) investigated the long-term impact of severe attachment problems using prospective studies with a follow-up of 10 years or more.

Results

Supplementary review 1: validity of attachment assessment tools

A total of 35 publications met the inclusion criteria for this phase of the review. The majority sought to validate an attachment assessment procedure under investigation against the SSP.

Attachment pattern assessments

In terms of test performance, two studies reported data that allowed concurrent validity calculation of sensitivity and specificity. When compared with the SSP in detecting secure attachment, the California Attachment Procedure reported a sensitivity of 0.90 [95% confidence interval (CI) 0.76 to 0.97] and a specificity of 0.30 (95% CI 0.11 to 0.54). The sensitivity of the Louisville Twin Study attachment procedure to detect secure attachment was 0.82 (95% CI 0.61 to 0.95) and the specificity was 0.66 (95% CI 0.29 to 0.92). A number of other instruments were compared with the reference standard (SSP), with a range of validity and reliability data reported.

The nomenclature for the SSP was varied. In 14 papers using this tool, 12 used variations of nomenclature or classification subtypes.

Attachment disorder assessments

The Disturbances of Attachment Interview was compared with a semistructured interview to elicit RDC for attachment disorder. This found a sensitivity of 0.81 (95% CI 0.54 to 0.96) and a specificity of 0.86 (95% CI 0.78 to 0.92) for disinhibited attachment disorder, and a sensitivity of 0.80 (95% CI 0.28 to 0.99) and a specificity of 0.99 (95% CI 0.95 to 1.00) for inhibited attachment disorder.

When exploring the validity and reliability of all these assessments under consideration, only 5 of the 35 studies reported test–retest data. Inter-rater reliability was the most frequently reported type of reliability data. A total of 26 studies reported these data for the index tests and 23 studies for the reference test. Of these 26, 24 had good inter-rater reliability as defined by a level of 0.7 or above. Cronbach's alphas were reported in 12 studies for the index tests (in 11 studies $\alpha > 0.7$) and four studies for the reference tests (in four studies $\alpha > 0.7$).

The only study measuring attachment patterns and attachment disorders at the same time suggested that these are largely separate constructs.

Supplementary review 2: 10-year outcome studies with an assessment of severe attachment problems at baseline

When we reviewed studies of 10-year follow-up where attachment had been measured at inception using either the SSP or a diagnosis of attachment disorder (WHO or APA), we found eight studies that reported long-term data in relation to severe attachment problems at baseline. Two of these studies measured the stability of attachment over time and two examined the relationship between severe attachment problems in infancy and later mental health problems. The remaining four studies met the criteria in terms of reporting 10-year outcomes or more and measuring severe attachment problems at inception, but did not report the outcomes of those with disorganised attachment separately. We found an association between severe attachment problems, and borderline personality disorder in young adulthood and psychopathology in adolescence. This information was generated for potential use in a health economics model that included 10-year outcomes and demonstrated a limited number of papers for this purpose. It is important to note that this does not include shorter-term outcomes than 10 years. These have been included in previous reviews.

Main systematic review

In total, 30 studies were identified, 29 of which were delivering an intervention in a hypothesised 'at-risk' group to improve attachment patterns. The remaining study concerned treatment for children who already had a diagnosis of reactive attachment disorder.

Interventions to modify attachment patterns

Within the clinical effectiveness review, 18 studies were identified that presented data comparing a parenting intervention with a control in a RCT. Only eight of these examined interventions to reduce disorganised attachment patterns (the subject of our review). Other studies sought to establish secure attachment patterns and a meta-analysis of these is included in an appendix of the full report (see *Appendix 6*).

Studies seeking to improve disorganised patterns of attachment ($n = 8$) were combined and the pooled estimate gave a post-treatment effect of 0.47 (95% CI 0.34 to 0.65; $p < 0.00001$). Most of these interventions include elements that sought to improve maternal sensitivity as a way to improve the child's attachment security.

Interventions for children with attachment disorder

One study was found that met the criteria for this phase. This was an intervention for foster carers of children with an attachment disorder. Although this showed a modest improvement and reduced costs, the difference was non-significant.

© Queen's Printer and Controller of HMSO 2015. This work was produced by Wright *et al.* under the terms of a commissioning contract issued by the Secretary of State for Health. This issue may be freely reproduced for the purposes of private research and study and extracts (or indeed, the full report) may be included in professional journals provided that suitable acknowledgement is made and the reproduction is not associated with any form of advertising. Applications for commercial reproduction should be addressed to: NIHR Journals Library, National Institute for Health Research, Evaluation, Trials and Studies Coordinating Centre, Alpha House, University of Southampton Science Park, Southampton SO16 7NS, UK.

Main systematic review (cost-effectiveness)

Only two studies were found that ran an economic evaluation of a parenting intervention. Limited information was found to populate a decision model with any reasonable degree of certainty. We therefore performed provisional budget impact analysis based on the available evidence. A rating system that looked at the quantity and quality of evidence necessary to inform an economic model demonstrated that there were large gaps in the identified literature that would need to be filled to produce a robust economic model.

Implications for research

In light of the results of our evidence synthesis, we found some promising research and a number of significant gaps in the literature that would be important to fill, in order to inform clinical practice and decision-makers.

There is good evidence that a disorganised attachment pattern identified through the SSP is a useful early-life measure to predict which infants in high-risk groups may have later psychopathology and require intervention. Many assessment tools have limited reliability and validity data, and further work in this area would be useful. The current evidence around diagnosis of attachment disorder is less clear, and the diagnostic systems are currently changing. Further outcome work would be helpful for this group.

The health economics analysis suggests that there is a need for further research to improve consensus on the definitions and assessments of attachment patterns and attachment disorders, to improve our understanding of the relationship between different assessments at different times and to gather more information about the long-term sequelae for different subpopulations. A cohort study would be appropriate for this work. This would support the development of fully powered RCTs to generate more robust clinical effectiveness research with high-quality resource utilisation and cost-effectiveness. One way to carry out this task would be to begin a new inception cohort with sequential measures of attachment and robust collection of risk factors and outcomes, and to use this large cohort to embed RCTs to carry out improved clinical effectiveness and cost-effectiveness work. Some of this work could also be established within existing cohorts.

Implications for practice

With regard to parenting interventions, there is now good evidence to suggest that early parenting work focusing on maternal sensitivity is clinically effective, and that a range of programmes deliver this with or without video feedback. It is preferable in clinical practice to use an attachment assessment tool or diagnostic criteria that show good validity and reliability.

Study registration

This study is registered as PROSPERO CRD42011001395.

Funding

Funding for this study was provided by the HTA programme of the NIHR.

Chapter 1 Background

What is attachment?

The importance of the relationship between a child and his or her main caregivers has been recognised for some time and was captured most notably in the work of John Bowlby.[1] It is inherently linked to the promotion of survival by increasing the safety of the child. Attachment is a biological instinct whereby the child seeks proximity to the caregiver when feeling alarmed or sensing threat, in the expectation that the caregiver will provide protection for the child and reduce the child's arousal. The child's signals are designed to elicit the caregiver's protective response. This response was termed by Bowlby as caregiving.[1] Attachment is the child's bond to the caregiver and caregiving is the caregiver's bond to the child; together, these bonds form an important aspect of the parent–child relationship. Attachment and caregiving allow the developing child to explore the environment safely and learn how to cope with the challenges and anxieties presented in the environment.[2]

Attachment is thought to be important in social competence and emotion regulation.[3] It dynamically influences interactions as well as proactive and reactive responses to the environment. All of these influence brain development.[4] On the basis of repeated caregiving experiences, the infant develops internal working models which are representations of self and others that are used in the development of templates for relationships.[5] Such relationships are characterised by caregiving and care-seeking behaviours that have been experienced and rehearsed in infancy. Bowlby defined attachment as 'the lasting psychological connectedness between human beings'.[1]

Attachment patterns and their antecedents

There are different attachment patterns (sometimes referred to as attachment styles or classifications, or attachment organisation). Although each of these terms has its supporters and its merits, for the purposes of this review we will be using the term 'attachment patterns'. It is the quality or nature of the attachments, not their intensity, which is at issue.

Differences in the behaviour of children towards their caregivers when the children are stressed have been noted over time. Early studies of attachment behaviours by Ainsworth and Wittig[2] sought to operationalise and better understand these differences using the Strange Situation Procedure (SSP) (see *The Strange Situation Procedure*), which they pioneered and which has been further developed. The patterns refer to the children's strategies, when alarmed or feeling threatened, for gaining proximity to the caregiver in order to be protected. On the basis of earlier experiences, secure children (B pattern) are confident in the availability, and benign and consistent response of their caregivers to their display or distress, accept their caregiver's comfort, return to equilibrium and resume play or exploration. By contrast, an insecure avoidant child (A pattern) has experienced the caregiver's rejection, anger or unresponsiveness to his or her attachment needs. Consequently, while sensing distress, the child's organised strategy will be not to show his or her distress to the caregiver. An insecure ambivalent/resistant child (C pattern) has experienced his or her caregiver as inconsistent and unpredictable. Consequently, these children's organised strategy will be to show their distress or fear and cling to the caregiver, but resist the caregiver's attempts to soothe them. According to the 'mainstream' ABC + D classification,[6] infants and young children who have been emotionally and physically abused or neglected, and whose caregivers have been frightening or frightened, show a lack of organised strategy to gain their caregiver's response when alarmed (D pattern). An alternative, Dynamic Maturational Model (DMM) developed by Crittenden[7] regards those children termed disorganised as not, in fact, lacking a strategy, but using both an A strategy in which they maximise cognition and suppress genuine emotion, and a C strategy in which they express anger and coyness while minimising use of cognition.

© Queen's Printer and Controller of HMSO 2015. This work was produced by Wright *et al.* under the terms of a commissioning contract issued by the Secretary of State for Health. This issue may be freely reproduced for the purposes of private research and study and extracts (or indeed, the full report) may be included in professional journals provided that suitable acknowledgement is made and the reproduction is not associated with any form of advertising. Applications for commercial reproduction should be addressed to: NIHR Journals Library, National Institute for Health Research, Evaluation, Trials and Studies Coordinating Centre, Alpha House, University of Southampton Science Park, Southampton SO16 7NS, UK.

It is known that different types of parenting practice are related to infant attachment patterns. Ainsworth and colleagues[8] found that parental ways of carrying infants, responsiveness to crying, levels of interference and ignoring or rejecting behaviours all showed significant associations with different attachment patterns. A meta-analysis of over 4000 mother–infant dyads[9] found only a small association between infant attachment classification and maternal sensitivity. This 'transmission gap' might be explained by the maternal sensitivity and actual behaviour towards the child being conceived as global, rather than attachment-specific maternal sensitivity. What has been shown is that child attachment patterns are related to reflective functioning of the caregiver,[10] parental mental states[11] and the ability of the mother to make appropriate mind-related comments about the child's mental state.[12] Moreover, a significant correlation has been found between the attachment patterns of mother and father respectively, measured pre birth, and the attachment patterns of the infant to his or her parents, at age 1 year with the mother and 18 months with the father.[13]

Further influences on attachment patterns have been proposed, including genetic factors, which have thus far evaded attempts at replication.[14] Gene–environment interactions and differential susceptibility are theories that continue to be explored.[15] Temperamental reactivity between monozygotic twins shows higher levels of correlation ($r = 0.77$) than that between dizygotic twins ($r = 0.44$),[16] but no significant association has been shown between temperamental reactivity and infant attachment classification. Bakermans-Kranenburg and Van IJzendoorn[15] give a good account of the relationship between temperament and attachment and the thorny issues in trying to unravel these complex relationships. When considering these issues, other authors have reminded us of the importance of potential transgenerational factors.[17]

Natural history

Stability

The term natural history here refers to the progression, evolution and stability of early patterns of attachment. As a rule of thumb, providing there is no change in caregiving pattern (by either the same or different caregivers) and with secure attachment, there is evidence of overall stability of the pattern.[18] Insecure and, more so, disorganised attachment are associated with caregiver and caregiving difficulties, which are more likely to undergo change over a child's development, both because of their likely inherent instability and because they are more liable to interventions which may influence them.[19] These factors are likely to be associated with a change in the child's attachment pattern. However, Bowlby[20] referred to 'defensive exclusion', by which he meant the child excluding new information about relationships which did not accord with his or her existing internal working models. This would suggest that there would need to be a sustained and perceptible change in caregiving to exert a meaningful effect on the child's attachment pattern.

Evolution within disorganised pattern

There is some evidence[21,22] that the behavioural pattern described as disorganised in infancy and early childhood evolves into coercive controlling or compulsive caregiving patterns in preschool and middle childhood, even in low-risk settings.[23] However, there may be continuing disorganisation at the representational level, as shown in narrative stem completion tasks[19,24] and family drawings.[25]

Change of assessed manifestation of attachment

With development, presumed manifestations of attachment, and therefore ways of assessing attachment, change. Thus, in infancy and early childhood, attention is given to the distressed child's behaviour in relation to his or her caregiver, classically in separation and reunion procedures. In middle childhood, it becomes increasingly difficult to create sufficiently stressful situations in order to activate and then assess attachment behaviour. The solution has been to devise assessments of representation of attachment[26] using narrative completions and pictures. In adolescence, there has been a further progression using linguistic representation of state of mind with respect to attachment, that is, coherence of accounts, by 'surprising the unconscious' (Ammaniti M, Candelori C, Dazzi N, De Coro A, Muscetta S, Ortu F, *et al.* University of Rome, 1990, unpublished protocol). The question then arises regarding how closely related

the putative age-related manifestations or expressions of attachment are and how well they are measured by various proposed instruments used at different ages. This suggests that it might be preferable to refer to predictability rather than stability.

The significance of attachment and its relationship to psychopathology

In studying associations between attachment patterns and impaired functioning or psychopathology, the question arises about the nature of the association. If the impairment can be causally explained by prior or concurrent attachment difficulties, then the impairment can be properly considered as an aspect of the natural history. However, it is also possible that the antecedents of attachment difficulties – specifically, harmful parent–child interactions and their associated risk factors – could, independently of attachment, contribute to the functional impairment and psychopathology. In practice, it is difficult to disentangle these two mechanisms.[27] For this reason, discussion of the significance of attachment and its relationship to psychopathology is placed in its entirety under natural history.

There are various examples of studies that have attempted to link attachment patterns with subsequent disorders or outcomes. Studies have sought to show that behaviour problems in children can be predicted by attachment patterns.[28–30] These include both emotional and conduct problems.[30] For example, Speltz and colleagues[31] found that only 20% of a sample of clinic-referred children with early-onset conduct problems were securely attached to their parents, whereas 72% of children in the control group were securely attached. Futh and colleagues[32] examined how attachment representation related to social functioning and psychopathology in a sample of 113 children, 50% of whom were defined as high risk and 50% as low risk. Behaviour problems rated by teachers were linked to disorganised attachment patterns. Disorganised attachment was also predictive of poorer social functioning[32] and poor school attendance, conduct disorder and academic underachievement.[33] Offenders are also more likely to report disturbed or insecure attachments, and separation from attachment figures in childhood is suggested as being associated with personality disorder in offenders.[34] Insecure attachment is also purportedly linked to increased reactivity to stress,[35] notably in increased cortisol reactivity, which has itself been associated with a range of psychopathologies, including psychotic illness.[36] Longitudinal studies have linked disorganised attachment with hostility and hyperactivity, aggression and oppositional defiant disorder in children[37] and with dissociative symptoms in 17- to 19-year-olds.[27] Furthermore, attachment disorders, as distinct from insecure attachment patterns, are purported to have increased comorbidity with conduct disorders, developmental delay, attention deficit hyperactivity disorder and post-traumatic stress disorder.[38]

One of the problems, however, is that much of this often-quoted research uses a range of methodologies, often in small or selected samples and often using bespoke or unvalidated instruments for measuring attachment. For us to be confident in these associations, this research needs to be carefully scrutinised using high-quality standards. Although insecure attachment patterns may represent risk factors for some future problems, approximately one-third of infants in normal populations show some form of insecure attachment. Thus, insecure patterns of attachment should not be considered as indicators of pathology, but rather, may be considered as potential risk factors for the child's future functioning.[39] In this sense, although many people with psychopathology may be more likely to have had insecure attachments, many infants with attachment pattern difficulties may not go on to develop psychopathology. Indeed, some argue that measurements from the SSP are poor predictors of psychopathology in longitudinal studies.[40]

Work that has sought to quantify these issues suggests that genetic influences for prosocial behaviours are strong and independent of attachment pattern.[41,42] The interaction between environment and genetics is complex, with different children varying in susceptibility to environmental influences on their subsequent attachment pattern. However, once a particular attachment pattern has developed, genetic influences appear to take a significantly less part in the development of those behaviours for which attachment patterns are seen as risk factors.

© Queen's Printer and Controller of HMSO 2015. This work was produced by Wright *et al.* under the terms of a commissioning contract issued by the Secretary of State for Health. This issue may be freely reproduced for the purposes of private research and study and extracts (or indeed, the full report) may be included in professional journals provided that suitable acknowledgement is made and the reproduction is not associated with any form of advertising. Applications for commercial reproduction should be addressed to: NIHR Journals Library, National Institute for Health Research, Evaluation, Trials and Studies Coordinating Centre, Alpha House, University of Southampton Science Park, Southampton SO16 7NS, UK.

In summary, while, there is some evidence that disorganised attachment patterns are related to psychopathology, the link between insecure patterns and subsequent problems is not so clear.[39] This lends itself urgently for review, given that many clinicians use the paradigm of attachment in assessment and intervention, and there is a need to better understand the evidence that informs clinical practice. We have enough literature to consider that disorganised attachment is the most promising candidate. It is associated with poor outcomes and is a group to follow up, exploring systematically whether or not parental interventions are effective or cost-effective. Attachment disorders, to be discussed below (see *Attachment disorders*), could also be included in the overall term 'severe attachment problems'.

Tools for assessing attachment patterns

For developmental reasons, there cannot be a single gold standard for the measure of attachment that is usable across ages of development and akin to the measurement of haemoglobin. As described above (see *Change of assessed manifestation of attachment*), there are, by necessity, different ways of assessing attachment. Moreover, whereas some tools use observation, others use self-reports, either by questionnaires or by interview, Q-sorts and parental questionnaires.[43] There are numerous tools, some of which vary in their coding of the same observational procedure (e.g. ABC + D and DMM).

Assessment of attachment behaviour

The Strange Situation Procedure

The first procedure, developed by Ainsworth and Wittig,[2] was the SSP, also called the Strange Situation Test. This involved observing the child's reactions in a situation where the child's mother and a stranger (a safe adult unknown to the child) interact with the infant. In sequence, this involves the infant being with the mother, then a stranger entering; then the mother leaving and the infant being left with the stranger; then the mother returning and the stranger leaving; then the mother leaving the child alone; then the stranger returning; and finally, the mother returning and the stranger leaving. The stranger is included as a stressor, and the infant's interaction with the stranger is not part of the assessment of security of attachment. Mary Ainsworth proposed that an attachment pattern can be observed and characterised by the child's behaviour towards the mother at the two reunions.[2] She described three main attachment patterns within her work: secure attachment, ambivalent insecure attachment and avoidant insecure attachment. A fourth pattern of attachment, termed 'disorganised insecure attachment', was later added.[44] This addition was thought to be very significant in that, as described above, it was the greatest predictor of psychopathology.[45]

The SSP was the first procedure for assessing and defining childhood attachment behaviours and has come to be the bedrock that defines attachment patterns in infancy and early childhood. The SSP is known to be cross-culturally valid but to have some cross-cultural differences.[46]

For older children, there are modifications of the SSP to take account of the developmental changes relating to what is regarded as stressful. For preschool children, an adapted procedure extends the second separation to 5 minutes and the coding is modified to include controlling under disorganisation.[47] For 6-year-olds, the procedure extends the separation to 1 hour and there is no stranger.[22]

Attachment Q-set

The attachment Q-set (AQS) can be used to describe secure base behaviour in a number of environments, either at home or in a public place, inside or outside. It is designed to cover the spectrum of attachment-relevant behaviours, with items concerning a broad range of secure base and exploratory behaviour, affective response and social cognition. The observer spends a set amount of time observing the child.[48]

Representations of attachment

The two main procedures by which to assess the older child's representations of attachment are narrative stem completion and the use of pictures, commencing from the age of 4 years. Variants include the MacArthur Story Stem Battery (MSSB),[49] the Story Stem Assessment Profile (SSAP) (Hodges J, Steele M, Hillman S, Henderson K, 2002, unpublished data) and the Manchester Child Attachment Story Task (MCAST).[50] Drawings are used in the Separation Anxiety Test (SAT) and the School-age Assessment of Attachment.[51]

Coherence of accounts

These assessments are based on semistructured interviews with the child, and what is rated is the linguistic representation of the child's state of mind with respect to attachment. The two main tools are the Child Attachment Interview (CAI) for 7- to 11-year-olds, adapted from the Adult Attachment Interview (AAI),[52] and the Friends and Family Interview.[53]

Self-report attachment pattern questionnaires have also been used in 4- to 12-year-olds.[54]

Meta-analysis evidence[23] shows numerous subcatergorisations of attachment patterns, but does suggest that the measurement of disorganised attachment can be reliable.

Attachment disorders

Another group of attachment 'problems' has been defined in terms of psychopathology and these are 'attachment disorders'. The World Health Organization (WHO) classification system, the *International Classification of Diseases*, Tenth Edition (ICD-10),[55] defines two main attachment disorders: reactive attachment disorder (RAD) and disinhibited attachment disorder (DAD). According to the ICD-10,[55] RAD is

> *characterized by persistent abnormalities in the child's pattern of social relationships that are associated with emotional disturbance and are reactive to changes in environmental circumstances (e.g. fearfulness and hyper vigilance, poor social interaction with peers, aggression towards self and others, misery, and growth failure in some cases).*

Disinhibited attachment disorder is described as[55]

> *a particular pattern of abnormal social functioning that arises during the first five years of life e.g. diffuse, nonselectively focussed attachment behaviour, attention-seeking and indiscriminately friendly behaviour, poorly modulated peer interactions; sometimes with associated emotional or behavioural disturbances. It tends to persist despite marked changes in environmental circumstances.*

One issue with attachment disorders is that they extend beyond attachment relationships, and many of the difficulties included are not related to the central construct of attachment. There is a lack of clarity about the relationship between attachment disorganisation and attachment disorders, and the two may be conceptually different. There is widespread misconception about the meaning of the presumed diagnoses of attachment disorders. What is clear, however, is that children who acquire this 'diagnosis' are very troubled in terms of their behaviour and interpersonal relationships. Some very questionable interventions have been applied to them.

The American Psychiatric Association (APA) classification system, *Diagnostic and Statistical Manual of Mental Disorders*-Fourth Edition (DSM-IV), refers to an inhibited and a disinhibited subtype, both requiring 'pathogenic care'.[56] This attempts to integrate the literature on attachment patterns and disorders, although this has been criticised[57] and some suggest that research evidence no longer supports the currently described defining features of attachment disorder.

© Queen's Printer and Controller of HMSO 2015. This work was produced by Wright *et al.* under the terms of a commissioning contract issued by the Secretary of State for Health. This issue may be freely reproduced for the purposes of private research and study and extracts (or indeed, the full report) may be included in professional journals provided that suitable acknowledgement is made and the reproduction is not associated with any form of advertising. Applications for commercial reproduction should be addressed to: NIHR Journals Library, National Institute for Health Research, Evaluation, Trials and Studies Coordinating Centre, Alpha House, University of Southampton Science Park, Southampton SO16 7NS, UK.

The DSM-IV[56] has now been updated to the *Diagnostic and Statistical Manual of Mental Disorders*-Fifth Edition (DSM-V).[58] In DSM-IV, RAD included an inhibited and a disinhibited subtype. In DSM-V, RAD no longer has a disinhibited subtype. RAD (emotionally withdrawn) remains, and a new diagnosis is created, called disinhibited social engagement disorder.

The WHO ICD-10[55] system is being revised and is under consultation, with a new system being released in 2016. Other classification systems for developmental disorders have also been proposed.[59] It remains to be seen how these widespread changes in different classification systems will influence practice and research.

Is there a gold standard for measuring attachment?

As discussed, for developmental reasons there cannot be a single gold standard for the measure of attachment that can be used across ages of development and akin to the measurement of haemoglobin. Attachment is expressed by observable behaviour, providing there is an age-appropriate stressor. With development, it is possible to capture internal working models such as projective tests, as in the story stem procedures.[60] Later still, it is the coherence of the cognitive and emotional processing of childhood attachment experiences which appears to indicate security of attachment.[61] The research literature is peppered with instruments and tools that suggest they are measuring attachment with variable amounts of evidence. Although many of these may indeed be measuring attachment, there needs to be more caution and clarity on how they relate to each other. We cannot assume total stability in attachment patterns over time, and so concurrent administration of instruments will help us better understand concurrent validity. We have carried out a supplementary review to explore concurrent validity further.

The SSP will be our reference standard for this purpose, but we will also include other instruments compared concurrently with each other.

Alongside attachment patterns, research diagnostic criteria (RDC) for attachment disorders (such as RAD and DAD) have also been defined. They therefore also represent reference standards for systematic review.

The literature is ready, therefore, for a review that clarifies the current situation and subjects the vast literature on attachment to rigorous, high-quality standards. This will hopefully clarify our current knowledge, the quality of research that informs it and future potential research directions.

Interventions for attachment problems (disorganised attachment patterns and attachment disorders)

Juffer and colleagues[62] undertook a meta-analysis of interventions aimed at increasing parental sensitivity, improving attachment or both. Seventy studies, including 88 interventions, were included within the analysis. The authors report that typically developing infants from middle-class families formed the basis of some samples. The most effective interventions were found to be those with a focused, behavioural approach which were aimed at increasing parental sensitivity. They were particularly effective when video feedback was used. Twenty-nine of the interventions investigated were specifically intended to improve attachment security. These showed a significant, although small effect size ($d = 0.19$). Again, those interventions which targeted parental sensitivity were the most effective at improving attachment relationships. Although this meta-analysis resulted in the development of a promising intervention,[62] the interventions focused more widely than on children with severe attachment problems, including preventative interventions for children with no current attachment problems and at low risk for developing them. A more clinically based practitioner review highlights a range of current intervention options, and notes that many of these have maternal sensitivity and an improved understanding of the developmental needs of the child as central components of therapy.[63] More research systematically reviewing high-quality parental intervention studies in high risk groups will be a helpful addition to the literature.

Policy and practice

The introduction of the *Every Child Matters* agenda[64] and the Children Act (2004)[65] provided a framework for all services to work together holistically to support children's development. The government has recognised that the early years of a child's development are of vital importance.[66] This has been incorporated into the Children's Plan,[67] a 10-year strategy that aims to promote the development of social and emotional skills during the early years of a child's life and onwards, including the promotion of attachment and bonding in the first years of life. The Early Years Foundation Stage[68] was developed with a focus on learning, development and welfare standards, and looks at the whole range of a child's cognitive and non-cognitive development.

An early years commission report, *Breakthrough Britain: The Next Generation*,[69] published by The Centre for Social Justice, suggested that government policy was focusing on reducing economic poverty and improving educational achievement and not on the importance of relationships in young children's development. It called for greater recognition of the role of attachment and family relationships in contributing to the well-being of children. The report argues that children who experience 'relationship dysfunction' are at a higher risk of later life difficulties than children exposed to economic or educational disadvantage.

The early years commission report[68] highlights the importance of parent–child relationships during the earliest years of a child's life and the need for effective intervention strategies aimed at parents in order to enhance children's social and emotional health and well-being. The report acknowledges how emotional, environmental, physical, biological and social factors are all interrelated. It further concludes that parenting educational programmes are effective and recommends the use of parent management training. Such programmes include the Incredible Years programme[70] and parent–child interaction therapy.[69]

The Department of Health has now developed the Healthy Child Programme (HCP),[71] an early intervention and prevention public health strategy for children aged 0–5 years.[72] The HCP feeds directly into the Children's Plan[68] and contributes to the National Service Framework for Children, Young People and Maternity Services.[73] The HCP aims to improve the health and well-being of children by adopting an integrated approach to support for children and families. This was delivered by health professionals, particularly health visitors, and was a service provided within Sure Start Children's Centres.[74] The Department of Health advocates that effective implementation of the HCP should lead to 'strong parent–child attachment and positive parenting, resulting in better social and emotional well being among children'.[71]

The National Academy of Parenting Practitioners (NAPP)[75] was established in 2007 with the aim of training and supporting practitioners in evidence-based parenting skills, programmes and therapies. Building on the knowledge gained by HCP in 'what works', a key aim of NAPP is to evaluate high-quality evidence in order that commissioners can commission effective parenting programmes. A commissioning toolkit containing a database of parenting interventions, available for different situations, was developed by the Children's Workforce Development Council in 2008.[76] The Department for Education and Skills set up the Parenting Fund in 2004. This funds projects to provide direct support to parenting services and to support nurturing relationships. More recently, Child and Adolescent Mental Health Service (CAMHS) Increasing Access to Psychological Therapies (IAPT)[77] has been rolled out across the country, with robust monitoring of child outcomes and parenting programmes coming to the fore in a second wave of therapies being delivered.

Recent government policy on early years education proposes to improve access to nursery education for the most disadvantaged 2-year-olds.[78] Given that those children with severe attachment problems are likely to come from the most disadvantaged families in society,[79] this is likely to have an impact and change the relationships, responsibilities and tasks of those caring for infants. This is as yet unevaluated in terms of attachment and other future outcomes.

© Queen's Printer and Controller of HMSO 2015. This work was produced by Wright *et al.* under the terms of a commissioning contract issued by the Secretary of State for Health. This issue may be freely reproduced for the purposes of private research and study and extracts (or indeed, the full report) may be included in professional journals provided that suitable acknowledgement is made and the reproduction is not associated with any form of advertising. Applications for commercial reproduction should be addressed to: NIHR Journals Library, National Institute for Health Research, Evaluation, Trials and Studies Coordinating Centre, Alpha House, University of Southampton Science Park, Southampton SO16 7NS, UK.

In a strategic review of health inequalities in England, Professor Sir Michael Marmot[80] highlighted the importance of acting in the early years. In reviewing the child protection system, Professor Eileen Munro[81] also suggested that early intervention is important, with a need to understand the importance of preventative services and early support for children.

In written evidence submitted to Frank Field's review of poverty and life chances,[82] 'many highlighted the importance of strong parent and child relationships' (see sections 6.11 and 6.15 in Field[82]) including 'the forming of strong attachments' (see section 6.11) [© Crown copyright 2010, contains public sector information licensed under the Open Government Licence v3.0 (www.nationalarchives.gov.uk/doc/open-government-licence/version/3/)]. It is not, however, specifically listed in this report as a strong predictor of children's life chances (see section 6.36), suggesting that although attachment is widely accepted as being important, additional research would be helpful to strengthen the evidence base.

The UK government response to these various reviews of early intervention services,[83] the prevention of poverty and its impact on children,[82] health inequalities[80] (including those affecting children) and the child protection system[84] was published in 2011.[85] This included a number of commitments, including an intent to continue to build an effective evidence base (see *Supporting Families in the Foundation Years*,[85] pp. 76–8); to improve systems to measure school readiness, for example through a revised Early Years Foundation Stage Profile[84] (p. 81[85]); to continue a rolling review of effective and evidence-based early intervention programmes (p. 82[85]); to continue to develop a more highly qualified early-years workforce (p. 83[85]); to refocus local services, including children's centres, on work to support the most disadvantaged children (p. 84[85]); to give parents and local communities more influence over local services they receive (p. 85[85]); and to explore a new foundation to champion early intervention (p. 85[85]). These both directly and indirectly require an improved evidence base on which to draw. The review published here provides additional evidence on what works and describes future research that is necessary.

Purpose of the present review

As highlighted in the brief literature review above, there are many gaps and ambiguities in the literature, and this confirms that 'the area of attachment is ripe for greater synthesis of evidence-based practice that covers both intervention and assessment'.[43] A particular limitation is the need to investigate the effectiveness of interventions in a UK setting.[86] The National Institute for Health and Care Excellence (NICE) is currently considering this issue. The main focus of this review is to systematically review the clinical effectiveness and cost-effectiveness of parenting interventions for severe attachment problems (disorganised attachment patterns and attachment disorders). *Chapter 2* will describe the aims, objectives and scope of this work and the decision problem that faces decision-makers in the context of a UK setting.

Chapter 2 Aims, objectives and scope

Many proposed parenting interventions are time-consuming and costly, utilising the time of experienced clinicians and therapists. The availability of such interventions in services is, therefore, limited. At present, services face uncertainty about who to prioritise for treatment. What is on offer, when and to whom varies largely from service to service, whether this be local authority provision, voluntary provision or services provided by child health or child mental health teams. As described in the opening chapter, there has been an increasing focus on the importance of attachment, parenting and early-life relationships in government policy.

Aims

The National Institute for Health Research (NIHR) Health Technology Assessment (HTA) programme commissioned a systematic review to provide more evidence, specifically around parenting interventions for parents of children likely to develop severe attachment problems. The main aim of the HTA call was to study:

> *The effectiveness and cost effectiveness of an early parenting intervention for parents whose children show signs of developing severe attachment problems*

1. *Technology: Interventions to support parents in modifying child behaviour to prevent, reduce and treat severe attachment problems.*
2. *Patient Group: Parents of children who show evidence of developing severe attachment problems.*
3. *Setting: Community.*
4. *Control or comparative treatment: Treatment as usual.*

Current review definition

We need initially to define what is included within a definition of severe attachment problems. The extant literature discussed in *Chapter 1* describes the best evidence to date linking both attachment disorders and disorganised attachment patterns with subsequent psychopathology. The use of insecure attachment as a predictor is less promising because of very high prevalence rates of insecure attachment (approximately 35%).[87] For the purposes of this review, therefore, we will consider severe attachment problems to be either attachment disorders, as defined using RDC (including RAD and DAD and the subtypes defined), or disorganised attachment patterns, using the SSP with the classification system that includes disorganised attachment pattern (*Figure 1*).

Scope of the review

Resources for this review were focused around the specific NIHR HTA programme call to explore the clinical effectiveness and cost-effectiveness of parental interventions for severe attachment problems. This is our main review. As the commissioned research has a focus around parental interventions, we have excluded studies that do not include parental interventions, where the focus may, for example, have been organisational, administrative or systemic. We include parenting/caregiver interventions working with a consistently available caregiver (alone or with caregiver and child, but not child alone). This would not, for example, include institutionalisation or multiple staff/child interactions as a parenting intervention. We are specifically examining the change in the child's attachment patterns or disorder and any associated changes.

© Queen's Printer and Controller of HMSO 2015. This work was produced by Wright *et al.* under the terms of a commissioning contract issued by the Secretary of State for Health. This issue may be freely reproduced for the purposes of private research and study and extracts (or indeed, the full report) may be included in professional journals provided that suitable acknowledgement is made and the reproduction is not associated with any form of advertising. Applications for commercial reproduction should be addressed to: NIHR Journals Library, National Institute for Health Research, Evaluation, Trials and Studies Coordinating Centre, Alpha House, University of Southampton Science Park, Southampton SO16 7NS, UK.

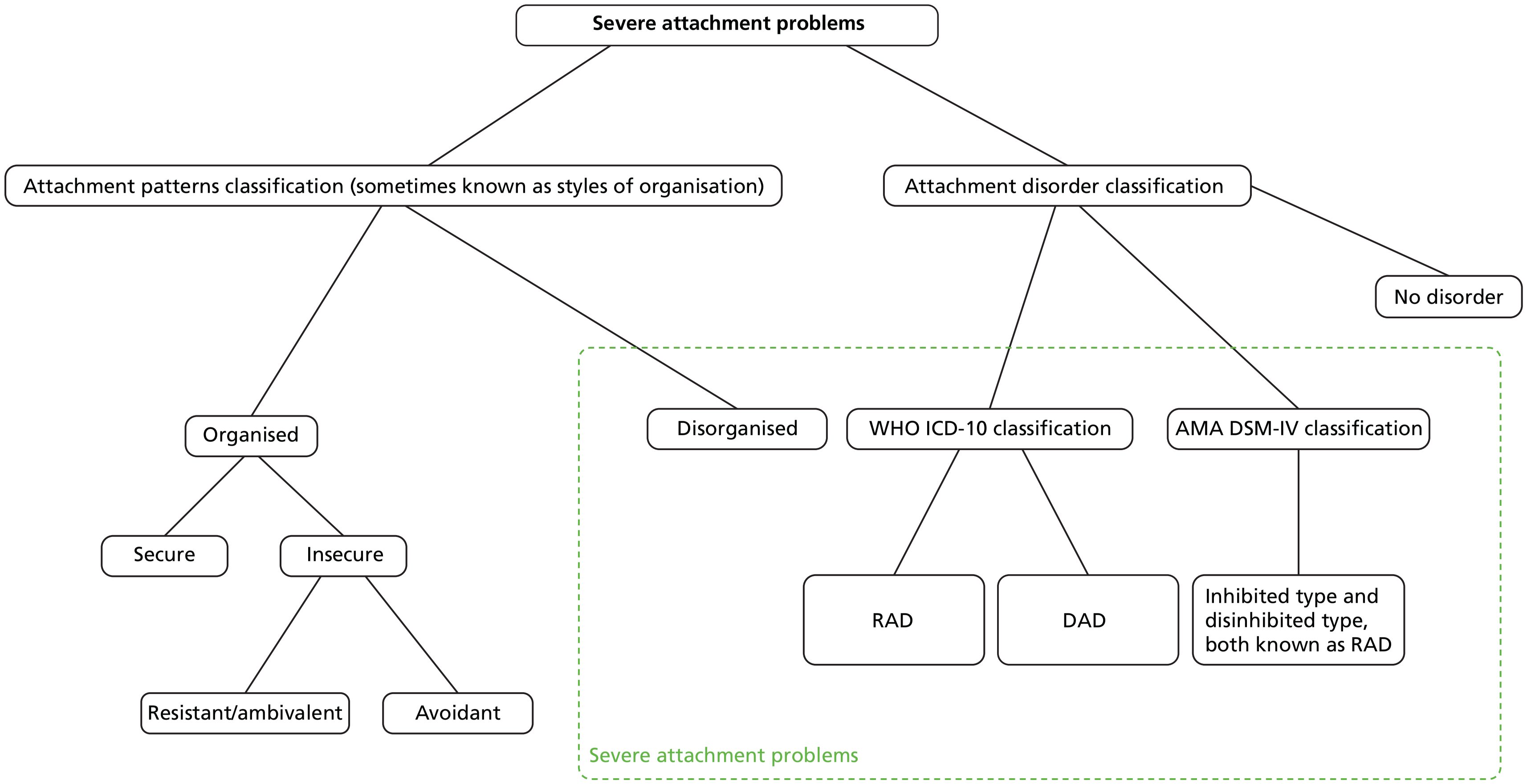

FIGURE 1 Severe attachment problems definition as defined by our review. AMA, American Medical Association.

In order to carry out this work, it was necessary to carry out two supplementary reviews. The first supplementary review assessed the mechanisms for identifying severe attachment problems (see *Chapter 4*). We also carried out a second supplementary review to bolster evidence to the health economists about long-term follow-ups (see *Chapter 5*). This restricted itself to a review of 10-year follow-up or more of infants/children with severe attachment problems at baseline to enable us to explore outcomes of children at primary school age and above. We recognise that there is a huge literature on shorter-term outcomes which has been covered extensively in other systematic review work and is not the central focus of our main review of parental interventions.

Objectives

To achieve the overall aim of assessing the clinical effectiveness and cost-effectiveness of parenting interventions, we specified a series of objectives as follows:

1. to identify the range of *intervention programmes* that are designed for parents of children with severe attachment problems (see *Chapter 6*)
2. to examine the *clinical effectiveness* of intervention programmes designed for parents of children with severe attachment problems (see *Chapter 6*)
3. to examine the *cost-effectiveness* of intervention programmes designed for parents of children with severe attachment problems (see *Chapter 7*)
4. to identify *research priorities* for developing future intervention programmes for children with severe attachment disorders, from the perspective of the UK NHS (see *Chapter 8*)
5. to review the methods of *assessment and/or diagnosis* of attachment patterns and/or disorders (supplementary systematic review 1; see *Chapter 4*)
6. to examine the 10-year and longer outcomes among children with severe attachment problems and collect prevalence information from these studies (supplementary systematic review 2; see *Chapter 5*).

Description of the decision problem for the purposes of health economic analysis

How do we identify those who will benefit from interventions?

The first step in providing clarity as to who should be prioritised for treatment is to clarify how we identify the children who will benefit from the available treatments in a valid and reliable way. As discussed in *Chapter 1*, a central problem facing this review on attachment is large differences between attachment patterns and attachment disorders. Furthermore, how do we identify severe attachment problems in infants or children when stability over time may vary? For example, a meta-analysis of 840 infants in nine samples, where assessments took place between 2 and 60 months apart, found a stability of $r = 0.34$ for disorganised attachment.[23] The concept of attachment may also be used by clinicians in many different ways, with some straying far from Bowlby's original construct[1] by using it to describe broad aspects of the quality of relationships between parent and child. These all lead to misunderstandings in interpreting the literature.

Many attachment instruments have not been well validated. There is currently no biological measure of attachment patterns. In regard to attachment disorders, the construct is under scrutiny and subject to revision, as is the case with both the APA (DSM-IV to DSM-V[56,58]) and WHO [ICD-10[55] to *International Classification of Diseases*, Eleventh Edition (ICD-11)[88]] definitions of attachment disorders. We can, however, review extant research diagnostic systems for attachment disorders from both groups.

Clinicians do not currently know which is the best assessment tool to use to identify severe attachment problems. Therefore, the aim of the first supplementary systematic review of the literature is to identify the valid and reliable assessments of attachment patterns and disorders. As this review has its focus on

© Queen's Printer and Controller of HMSO 2015. This work was produced by Wright *et al.* under the terms of a commissioning contract issued by the Secretary of State for Health. This issue may be freely reproduced for the purposes of private research and study and extracts (or indeed, the full report) may be included in professional journals provided that suitable acknowledgement is made and the reproduction is not associated with any form of advertising. Applications for commercial reproduction should be addressed to: NIHR Journals Library, National Institute for Health Research, Evaluation, Trials and Studies Coordinating Centre, Alpha House, University of Southampton Science Park, Southampton SO16 7NS, UK.

parenting interventions, we are interested in identifying severe attachment problems early in life. In order to better understand the relationship between attachment patterns and attachment disorders, we will also explore how they relate to one another by looking for any studies that have compared their use in the same children at the same time.

Who is at risk and who is it that we should be treating?

Once we have identified clear ways of measuring severe attachment problems that are reliable and valid, we need to know what this means in terms of outcomes for the child, whether they receive the intervention or not. We need to understand more clearly what it means to have different attachment patterns in infancy[1,6,8] or attachment disorders[57] in older infants and children. What happens to those children in the longer term? For health economic reasons, we are particularly interested in studies that look at follow-up that takes infants or children at least to the end of primary school education, and hopefully considerably beyond, to inform any health economic modelling work. Very short- or short-term studies, although important for many reasons, are less useful for this purpose.

Using the findings from the first systematic review on assessment and measurement, we seek to evaluate the longer-term outcomes for those identified that have been left untreated. This forms the second supplementary systematic review (see *Chapter 5*). This will look at the evidence from longitudinal studies that follow children up for 10 years or more. We will explore attachment outcomes and, where possible, whether or not other outcome information is of value in its current form. This is a small supplementary review to inform the main focus of this work, which centres on the clinical effectiveness and cost-effectiveness of parental interventions.

Which parenting interventions work and are they cost-effective?

Clinicians are often unsure about the best intervention or treatment options for the children (and their families) identified as having severe attachment problems. Resources for these interventions are limited. There are ambiguities surrounding the clinical effectiveness and cost-effectiveness of interventions provided to families, including whether or not any improvement in attachment would be associated with a change in other outcomes (educational attainment, psychological well-being, quality of life, future criminality, etc.), and how acceptable these interventions would be in terms of the practicalities of delivering them in busy services and their acceptability to service users.

The attachment literature investigating the efficacy of parenting interventions consists of a variety of research designs, from single case-study designs to randomised controlled trials (RCTs). We seek to systematically review this literature, selecting only RCT designs which illustrate the highest level of evidence for the clinical efficacy of treatment. Do the interventions work (see *Chapter 6*) and are they cost-effective (see *Chapter 7*) in a current environment where funding is tight?

By liaising with experts and service users in patient and public involvement (PPI) groups as we conduct our reviews, we hope to identify any gaps in the literature and the acceptability of interventions that are found to be clinically effective.

Overview of process

A single comprehensive literature search strategy was carried out to identify the evidence needed for the review (see *Chapter 3*). This was then passed to three teams of systematic reviewers. The first conducted the main review of clinical effectiveness and cost-effectiveness alongside the health economists. Two supplementary review teams carried out work on assessment tools and 10-year follow-up after baseline severe attachment problems. At each stage of the review and the production of the final report, we adhered to the relevant guidelines for the conduct and reporting of systematic reviews [Centre for Reviews and Dissemination (CRD),[89] Preferred Reporting Items for Systematic Reviews and Meta-Analyses (PRISMA)[90] and Cochrane[91] guidelines].

Chapter 3 Literature search

The main focus of the literature search was to identify studies about the clinical effectiveness and cost-effectiveness of parental intervention programmes for children with severe attachment problems. However, we also wanted more broadly to identify studies about methods of assessment and diagnosis. In order to provide additional information for the health economics aspect of cost-effectiveness, we systematically reviewed 10-year follow-up studies and extracted any outcome data and prevalence estimates from within these studies. It was decided, following an initial scoping exercise, that a single comprehensive search, as opposed to a separate search for each phase of the review, would be the most effective and efficient means of identifying the relevant literature for each phase. A large single search encompassing five search strategies was designed (see *Appendix 1*) to identify studies about attachment disorder/patterns/problems from the following perspectives:

1. assessment/diagnosis
2. epidemiology/natural history
3. named intervention programmes
4. controlled trials
5. economics/costs.

At all stages, the CRD guidelines were followed.

Search strategy

A range of databases and organisational websites, covering both databases of predominantly peer-reviewed citations and grey literature sources, were searched to identify relevant clinical effectiveness and cost-effectiveness literature:

- PsycINFO
- MEDLINE and MEDLINE In-Process & Other Non-Indexed Citations
- EMBASE
- Social Policy & Practice
- Science Citation Index (SCI)
- Social Sciences Citation Index (SSCI)
- Conference Proceedings Citation Index – Science (CPCI-S)
- Conference Proceedings Citation Index – Social Science & Humanities (CPCI-SSH)
- Education Resources Information Center (ERIC)
- Social Services Abstracts
- Applied Social Sciences Index and Abstracts (ASSIA)
- Cochrane Database of Systematic Reviews (CDSR)
- Database of Abstracts of Reviews of Effects (DARE)
- Cochrane Central Register of Controlled Trials (CENTRAL)
- HTA database
- NHS Economic Evaluation Database (NHS EED)
- The Campbell Library
- Health Economic Evaluations Database (HEED)
- Social Care Online
- Research Register for Social Care
- Index to THESES
- OAIster
- OpenGrey
- Zetoc

© Queen's Printer and Controller of HMSO 2015. This work was produced by Wright *et al.* under the terms of a commissioning contract issued by the Secretary of State for Health. This issue may be freely reproduced for the purposes of private research and study and extracts (or indeed, the full report) may be included in professional journals provided that suitable acknowledgement is made and the reproduction is not associated with any form of advertising. Applications for commercial reproduction should be addressed to: NIHR Journals Library, National Institute for Health Research, Evaluation, Trials and Studies Coordinating Centre, Alpha House, University of Southampton Science Park, Southampton SO16 7NS, UK.

- ClinicalTrials.gov
- *meta*Register of Current Controlled Trials (*m*RCT)
- WHO International Clinical Trials Registry Platform (ICTRP)
- UK Clinical Research Network (UKCRN)
- Health Services Research Projects in Progress (HSRProj).

The following organisation websites were also searched:

- APA (www.psych.org/)
- Association for Child and Adolescent Mental Health (www.acamh.org.uk/)
- Mental Health Foundation (www.mentalhealth.org.uk/)
- MIND (www.mind.org.uk/)
- Royal College of Psychiatrists (www.rcpsych.ac.uk/)
- National Collaborating Centre for Mental Health (NCCMH) (www.nccmh.org.uk/)
- National Institute of Mental Health (NIMH) (www.nimh.nih.gov/index.shtml)
- Institute for Attachment & Child Development (www.instituteforattachment.org/)
- Association for Treatment and Training in the Attachment of Children (www.attach.org/)
- YoungMinds (www.youngminds.org.uk/)
- British Association for Adoption and Fostering (www.baaf.org.uk/).

All searches were carried out between 6 and 12 January 2012.

Search terms

The literature searches involved searching a wide range of databases covering research in the fields of health, mental health, health economics, education and social care. The search strategies were devised using a combination of subject indexing terms (where available), such as medical subject headings (MeSH) in MEDLINE, and free-text search terms in the title and abstract. The search terms were identified through discussion in the research team, by scanning background literature and by browsing database thesauri. *Appendix 1* provides the full list of search terms for each of the included databases.

In a number of resources it was possible to conduct generic searches for 'attachment', rather than undertake five separate targeted searches. For the 'assessment', 'controlled trials' and 'economics' searches we included methodological search filters identified from the InterTASC Information Specialists' Sub-Group Search Filter Resource (www.york.ac.uk/inst/crd/intertasc/index.htm).

This approach still retrieved relatively large numbers of results, and so we introduced a further facet of search terms for 'children', 'parents', 'fostering', 'adoption', 'child neglect' and 'child abuse'. The introduction of this facet made the results more precise by removing much of the adult-oriented literature about romantic/couple attachment, God/religion attachment, friendship problems and other similar attachment-related items in which we had no interest. A further limit was introduced to the search strategy which removed selected publication types (letters, editorials and book reviews).

No limitations were made in terms of publication status, publication date or language.

Screening of citations

The titles and abstracts of bibliographic records were downloaded and imported into EndNote bibliographic management software (version 5; Thomson Reuters, CA, USA) and duplicate records were removed using several algorithms. Two reviewers screened the titles and abstracts to identify potentially eligible studies produced from the literature search. Full papers for potentially eligible studies were obtained and assessed for inclusion independently by two reviewers. Any disagreements were resolved by consensus, or by a third party when necessary, at both the abstract and full-paper sift.

Inclusion and exclusion criteria

Detailed separate participants, interventions, comparisons, outcomes, study design (PICOS) criteria were developed for the different phases of the review (for further details see each individual relevant chapter). Reviewers were instructed to be inclusive at the first sift (titles and abstracts) if there was any uncertainty about a reference, but to apply the PICOS criteria rigorously at the second sift (full paper).

Additional search strategies

A manual search of the reference lists of included studies was conducted to ensure that all studies had been identified. Authors were subsequently contacted to clarify information or gain additional studies that might be unpublished or ongoing. Systematic reviews and meta-analyses that contained potentially relevant references for inclusion in the review were flagged, to be searched and reference checked at the end of the screening.

Stakeholder involvement

A range of different stakeholders were contacted to help us frame some of our ideas and understanding about the associated problems of caring for and conducting research with young people with attachment patterns or disorders. This PPI was integral to the work and the stakeholders formed part of the wider research team. They were involved from the creation of the protocol, helping to identify what the issues were, how to contextualise the intervention findings and how to present information, and will be involved in determining how the findings should best be disseminated. Throughout the project we consulted with academics with methodological expertise in the conduct of systematic reviews and economic analysis and content expertise of attachment theory and disorders.

In addition to membership of a steering group, we held PPI/stakeholder workshops in February 2013 and September 2013. The workshops provided an outline of the research project and the group (consisting of parents and expert academics working in the field) were asked to take part in a series of focus groups. We were particularly interested in generating knowledge which might inform the economic decision modelling process, currently available parenting interventions and desirable treatment options and mechanisms to disseminate the research findings. *Appendix 2* provides a list of the stakeholder and advisory group members.

© Queen's Printer and Controller of HMSO 2015. This work was produced by Wright *et al.* under the terms of a commissioning contract issued by the Secretary of State for Health. This issue may be freely reproduced for the purposes of private research and study and extracts (or indeed, the full report) may be included in professional journals provided that suitable acknowledgement is made and the reproduction is not associated with any form of advertising. Applications for commercial reproduction should be addressed to: NIHR Journals Library, National Institute for Health Research, Evaluation, Trials and Studies Coordinating Centre, Alpha House, University of Southampton Science Park, Southampton SO16 7NS, UK.

Chapter 4 Supplementary systematic review 1: validity of methods to identify attachment patterns and disorders

Introduction

The research objective of our first supplementary review was to review the methods of assessment and/or diagnosis of attachment problems and/or disorders.

The literature referring to the concept of infant attachment is vast. Defined clinical and research paradigms, such as attachment patterns and disorders as discussed in *Chapter 1*, differ from each other in a number of significant ways. In order for research on potential parental interventions for severe attachment problems to progress, it is necessary to be clear about how we are defining and identifying severe attachment problems. For example, the attachment pattern literature seeks to identify risk factors and identifiable behaviours that give us important developmental information. By contrast, the attachment disorder literature sits within the context of diagnostic systems. They therefore come from very different traditions. This supplementary systematic review seeks to shine further light on the evidence base in this area to date.

We have set out to explore studies in which tools available to screen, assess and/or diagnose attachment problems (both attachment patterns and attachment disorders) are compared with each other, and we are particularly interested in concurrent validity. This is to complement the fundamentally different work of Van IJzendoorn and Bakermans-Kranenburg,[23] who empirically studied single measures of disorganised attachment but without comparison with other instruments. We provide information on the procedures surrounding each tool identified in the review, the psychometric properties and validity of the reported tools and the population studied. Where raw data are available in a comparison between a reference standard and another instrument concurrently used, we calculate sensitivity and specificity. We also carry out a quality assessment of each publication. Finally, for those instruments meeting the quality criteria and where comparison with a reference standard is available, we describe the instrument in more detail to form part of taxonomy.

By extracting this information, we can establish the variability in the assessment tools available and how they relate to the reference standards. This informed our choice of instruments to use in the second supplementary review, exploring outcomes of severe attachment problems at 10 years or more, and laid out the state of research in this field to inform future research directions.

© Queen's Printer and Controller of HMSO 2015. This work was produced by Wright *et al.* under the terms of a commissioning contract issued by the Secretary of State for Health. This issue may be freely reproduced for the purposes of private research and study and extracts (or indeed, the full report) may be included in professional journals provided that suitable acknowledgement is made and the reproduction is not associated with any form of advertising. Applications for commercial reproduction should be addressed to: NIHR Journals Library, National Institute for Health Research, Evaluation, Trials and Studies Coordinating Centre, Alpha House, University of Southampton Science Park, Southampton SO16 7NS, UK.

Methods

The identified literature was dual screened according to the screening criteria specified in *Inclusion criteria*. Initially, titles and abstracts were reviewed independently, with disagreements discussed and resolved between reviewers and a third party when required. Complete copies of all potential 'includes' (papers to be included) were then obtained. When required, disagreements were discussed and resolved by a third party. Where a foreign language paper was identified, translation then screening was performed as above.

Inclusion criteria

All study designs were eligible in this stage of the review. For inclusion, studies had to provide sufficient data for extraction. Sensitivity and specificity analysis data were not a requirement, although this analysis was undertaken where possible (only where complete raw data were available).

The PICOS criteria were as follows:

- *Population and setting* Children being assessed for attachment patterns or disorders where the research reports an average age of 13 years or below (we chose this in discussion with PPI and experts in the light of the overall aim of the review on early parental interventions). As discussed in *Chapter 1*, we refer to attachment patterns to mean any paper that explored attachment patterns, attachment styles or attachment organisation, recognising that different authors in the field use different terminology. We felt that it was important not to exclude any papers that were relevant but used different terminology.
- *Intervention* Screening, assessment and/or diagnostic tools evaluating attachment patterns or disorders. The instrument must have been under development or evaluation, and must have been a completed tool or subscale on attachment rather than an individual item. Attachment pattern requires a primary caregiver (NB a member of staff in a child care institution is not considered a fair test).
- *Reference* A comparison tool assessing attachment patterns or disorders identified by ICD-10[55] or DSM criteria.[56]
- *Outcomes* Studies reporting on the psychometric properties and validity of the tools.
- *Study design* Cross-sectional studies, case–control studies or prospective cohort studies incorporating any method of assessment (for example observation, semistructured interviews and questionnaires).

Data extraction

A data extraction form was developed, piloted and adapted on the basis of this piloting. Subsequently, all studies were dual extracted and reviewers met to agree and discuss discrepancies in data items. Where studies had multiple publications, data were extracted as a single study. The following items were extracted from each study: study characteristics, population details, index and reference tool details, data for sensitivity and specificity analysis, economic resource information and psychometric properties of index and reference tools.

Diagnostic accuracy

Where possible, a sensitivity and specificity analysis was calculated.

Quality assessment strategy

Each study was assessed for methodological quality by two reviewers using the quality assessment of diagnostic accuracy studies – version 2 (QUADAS-2).[92] Discrepancies in quality assessment were discussed and resolved between reviewers. QUADAS-2[92] is a validated quality assessment tool for diagnostic studies. It consists of four key domains: domain 1, patient selection; domain 2, index test(s); domain 3, reference standard(s); and domain 4, flow and timing [flow of patients through the study and the timing of the index and reference test(s)]. To help reach a judgement on the risk of bias, signalling questions were included. These flagged aspects of study design related to the potential for bias and aimed to help reviewers make risk-of-bias judgements. A further three questions in the tool consider the applicability of the patient selection, index tool and reference tool. Each item was rated 'yes', 'no' or 'unclear' according to the guidance provided.

Following quality assessment of the first few studies, it became apparent that the range of study designs made two questions irrelevant to some studies, as follows:

- Domain 2. Question 2: if a threshold was used, was it prespecified?
- Domain 3. Question 1: is the reference standard likely to correctly classify the target condition?

In order to avoid penalising studies where these aspects were not relevant, we agreed to enter a response of 'not applicable'. The following circumstances led to an opinion of 'not applicable': in cases where screening was performed using observational opinion, question 2 is not applicable; in studies where diagnosis is not the objective and, therefore, a 'cut-off' is not specified, question 2 is not applicable; and finally, in case–control studies where only one tool is assessed, question 1 is not applicable.

Data synthesis

A meta-analysis was not conducted because it was not appropriate. A wide range of instruments were compared with the reference standards, most of which were not repeated in further study to enable comparison between studies. A descriptive summary of results is presented.

Results

The initial literature search identified 10,167 publications after the removal of duplications. Following title/abstract screening and additional reference checks, 454 publications were full-paper screened. *Figure 2* (PRISMA diagram) details the flow of screened, included and excluded articles. A total of 35 publications[24,25,47,50,52,93–122] met the inclusion criteria for this phase of the review, of which two[109,112] duplicated data from other included reports.

Three studies were found that compared an attachment assessment procedure with the reference standard (SSP)[93–95] (see *Table 1* for a summary of the characteristics of these studies). Two of the studies were conducted in the USA[93,94] and the third study was conducted in Romania.[95] The ages of the samples ranged from 17 to 25 months. There was no significant difference in the proportions of boys and girls in any of the studies.

© Queen's Printer and Controller of HMSO 2015. This work was produced by Wright *et al.* under the terms of a commissioning contract issued by the Secretary of State for Health. This issue may be freely reproduced for the purposes of private research and study and extracts (or indeed, the full report) may be included in professional journals provided that suitable acknowledgement is made and the reproduction is not associated with any form of advertising. Applications for commercial reproduction should be addressed to: NIHR Journals Library, National Institute for Health Research, Evaluation, Trials and Studies Coordinating Centre, Alpha House, University of Southampton Science Park, Southampton SO16 7NS, UK.

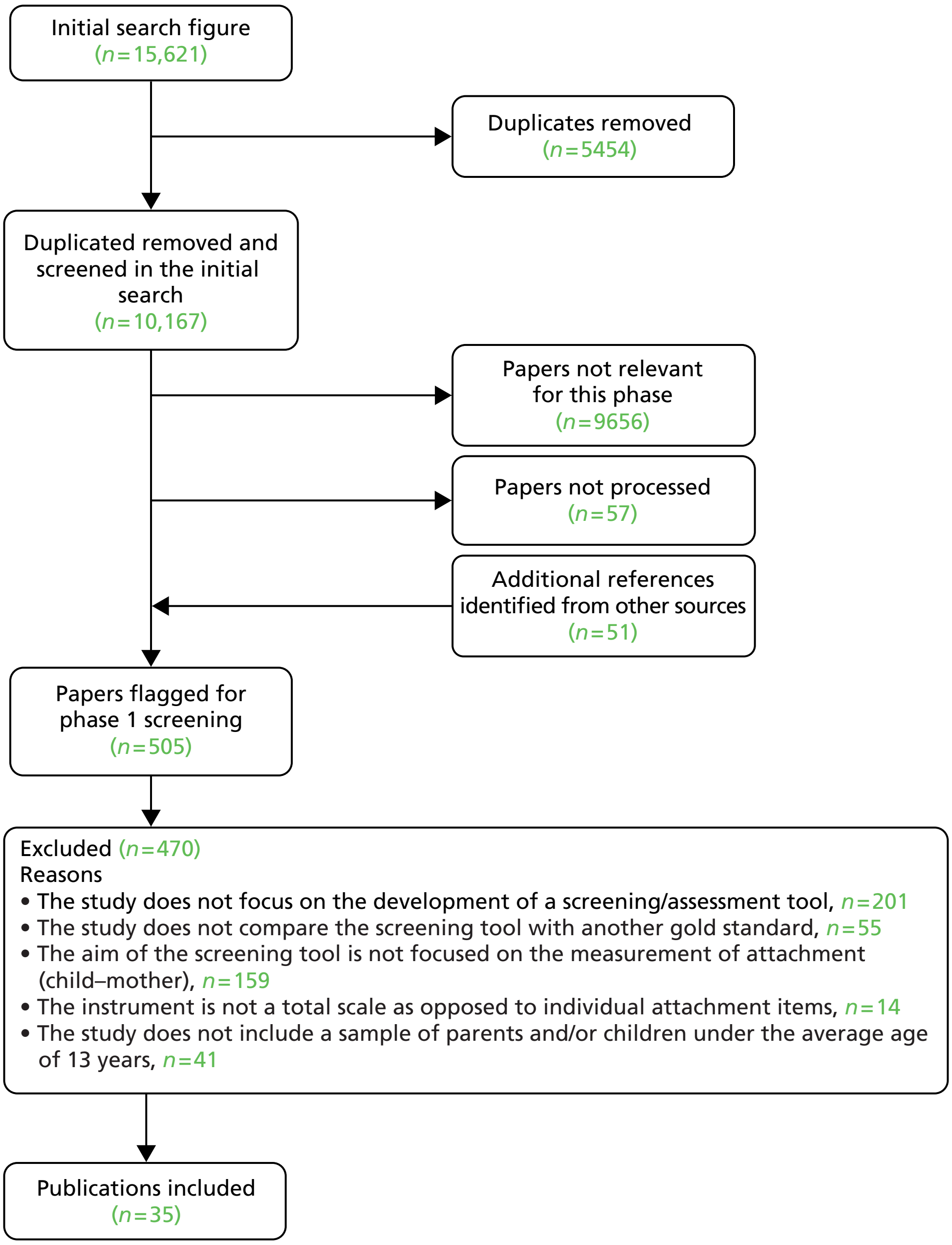

FIGURE 2 A PRISMA diagram illustrating the results of the screening process in the supplementary review.

Study characteristics

An overview of study characteristics is detailed in *Table 1* and a taxonomy of the tools identified is presented in *Tables 2* and *3*. Thirty-three studies were published between 1988 and 2011, of which the majority were undertaken in the USA (*n* = 18[24,47,93,94,96–100,102–104,106,111,113,115,116,118,121]), with the rest spread across the UK (*n* = 4[50,52,107–109]), Canada (*n* = 4[25,105,110,119]), Germany (*n* = 2[101,122]), the Netherlands (*n* = 2[117,120]), Romania (*n* = 1[94]) and Spain (*n* = 1[114]).

TABLE 1 Overview of screening and assessment studies and instrument characteristics

Author, year and country of publication	Participant details: children	Participant details: parents	Test instrument(s) (classification)/tool description and administration	Comparison test(s) (classification)/tool description and administration	Coding classification key (see *Box 1*)
Aber *et al.* (1990)[96]<br><br>USA	$n = 58$<br><br>Age range 19–24 months<br><br>24 males<br><br>Ethnicity unknown	Details unknown	Modified SSP (summary scores derived)<br><br>Scales of 0–3 and qualitative assessment on 18 behavioural variables. Time 30 minutes. Delivered by research assistant. Conducted in playgroup room	Teacher-sorted Toddler AQS<br><br>Adapted Waters and Deane (1985),[48] 92-item sort. Time 7–14 hours. Delivered by teacher. Conducted in playgroup room	
Backman (2003)[97]<br><br>USA	$N = 37$<br><br>Clinical: $n = 20$; ethnicity 10 mixed<br><br>Normative: $n = 17$; ethnicity 10 white<br><br>Age range 1–5 years<br><br>Gender unknown	Clinical group: mean age 28.30 years, age range 18–44 years; ethnicity seven white<br><br>Normative group: mean age 33.24 years, age range 24–44 years; ethnicity 10 white	MIMRS (summary scores derived)<br><br>7–10 task cards rated on 5-point scales. Time not reported. Delivered by mother and researcher. Location not reported	AQS<br><br>Waters (1987),[123] 90-item sort. Time 2–6 hours. Delivered by mother. Location not reported	
Boris *et al.* (2004)[98]<br><br>USA	$n = 69$<br><br>Age: mean/SD not reported, range 13–48 months<br><br>Gender: 45–54.5% male<br><br>Ethnicity unknown	Age: mean/SD not reported, range 17–35 years<br><br>Ethnicity: 9.1–55.0% white	Clinical assessment (DSM-IV criteria for presence/absence of attachment disorders)<br><br>Diagnostic manual. Time not reported. Delivered by experienced clinical assessor. Laboratory setting	SSP: standard Ainsworth laboratory procedure [Ainsworth (1978)[8]]<br><br>*and*<br><br>AQS: Waters and Deane (1985),[48] 90-item sort. Time: 2 hours. Delivered by trained observers. Conducted at home	(CC5)

continued

© Queen's Printer and Controller of HMSO 2015. This work was produced by Wright *et al.* under the terms of a commissioning contract issued by the Secretary of State for Health. This issue may be freely reproduced for the purposes of private research and study and extracts (or indeed, the full report) may be included in professional journals provided that suitable acknowledgement is made and the reproduction is not associated with any form of advertising. Applications for commercial reproduction should be addressed to: NIHR Journals Library, National Institute for Health Research, Evaluation, Trials and Studies Coordinating Centre, Alpha House, University of Southampton Science Park, Southampton SO16 7NS, UK.

TABLE 1 Overview of screening and assessment studies and instrument characteristics (*continued*)

Author, year and country of publication	Participant details: children	Participant details: parents	Test instrument(s) (classification)/tool description and administration	Comparison test(s) (classification)/tool description and administration	Coding classification key (see *Box 1*)
Bureau *et al.* (2009)[99]<br>USA	*n* = 43<br>Age range 7.3–9.6 years<br>Gender unknown<br>Ethnicity 81% Caucasian	Details unknown	MCDC scales (CC28)<br>Behavioural rating scales from 1 to 9. Time 1 hour 5 minutes. Delivered by interviewer. Laboratory setting	SAT (CC9)<br>Six story drawings. Time not reported. Unclear who administered. Location not reported	(CC9) (CC28)
Cassidy (1992)[47]<br>USA	*n* = 52<br>Mean age 6.2 years, range 5.7–6.8 years<br>26 males<br>Ethnicity unknown	Mean age 35.2 years, range 28–44 years<br>Ethnicity unknown	Incomplete stories with doll family (CC21)<br>Six stories rated on 5-point scales. Time not reported. Delivered by experimenter. Location not reported	Separation–reunion episode (CC24)<br>9-point scales. Time not reported. Delivered by experimenter. Location not reported	(CC24) (CC21)
Clarke-Stewart *et al.* (2001)[93]<br>USA	*n* = 60<br>Age range unknown<br>Mean age 16.6 months (SD 1.11 months)<br>Gender unknown<br>Ethnicity 79% white	Average age 32 years<br>Ethnicity unknown	CAP (CC6)<br>Three stressor stimuli under observation. Time 1 hour. Delivered by research assistant. Laboratory setting	SSP (CC6)<br>Standard Ainsworth laboratory procedure [Ainsworth (1969),[3] (1978)[8]]	(CC6)

Author, year and country of publication	Participant details: children	Participant details: parents	Test instrument(s) (classification)/tool description and administration	Comparison test(s) (classification)/tool description and administration	Coding classification key (see *Box 1*)
Crittenden *et al.* (2007)[100]<br><br>USA	*n* = 51<br><br>Age: mean 39 months, (SD 5.2 months) range 2.5–4 years<br><br>Gender: 57% males<br><br>Ethnicity Caucasian	Details not reported	SSP (CC11)<br><br>SSP; Ainsworth extended method [Crittenden (1985)[124]]. Time: 20 minutes for procedure. Delivered by undergraduate coders. Laboratory setting	SSP (CC26)<br><br>Cassidy–Marvin classification method.[47] Reclassification of Ainsworth extended method video. Delivered by trained graduate coders. Laboratory setting<br><br>*and*<br><br>SSP (CC22)<br><br>PAA classification method. Reclassification of Ainsworth extended method video.[8] Delivered by trained graduate coders. Laboratory setting	(CC26) (CC11) (CC22)
Equit *et al.* (2011)[101]<br><br>Germany	*n* = 299<br><br>Mean age 3.94 years, range 0–5 years<br><br>182 males<br><br>Ethnicity unknown	Details unknown	DC: 0–3R used to screen psychiatric referrals for any diagnosis<br><br>Diagnostic manual. Time not reported. Delivered by psychiatrist and clinical psychologist. Location not reported	ICD-10[55] used to screen psychiatric referrals for any diagnosis<br><br>Diagnostic manual. 3–4.5 hours. Delivered by child psychiatrist or clinical psychologist. Location not reported	
Fagot *et al.* (1996)[102]<br><br>USA	*n* = 175 (completed cases, *n* = 96)<br><br>Aged 18 and 30 months at first and second visits, respectively<br><br>Gender unknown<br><br>Ethnicity 95% European American	Details unknown	Modified SSP 30 months (CC12)<br><br>Time not reported. Shortened Ainsworth procedure [Ainsworth (1978)[8]] (only one reunion episode)	SSP 18 months (CC7)<br><br>Standard Ainsworth laboratory procedure [Ainsworth (1969)[3]]	(CC7) (CC12)

continued

© Queen's Printer and Controller of HMSO 2015. This work was produced by Wright *et al.* under the terms of a commissioning contract issued by the Secretary of State for Health. This issue may be freely reproduced for the purposes of private research and study and extracts (or indeed, the full report) may be included in professional journals provided that suitable acknowledgement is made and the reproduction is not associated with any form of advertising. Applications for commercial reproduction should be addressed to: NIHR Journals Library, National Institute for Health Research, Evaluation, Trials and Studies Coordinating Centre, Alpha House, University of Southampton Science Park, Southampton SO16 7NS, UK.

TABLE 1 Overview of screening and assessment studies and instrument characteristics (*continued*)

Author, year and country of publication	Participant details: children	Participant details: parents	Test instrument(s) (classification)/tool description and administration	Comparison test(s) (classification)/tool description and administration	Coding classification key (see *Box 1*)
Finkel *et al.* (1998)[94]<br>USA	$n = 16$<br>Age range 19–25 months<br>Gender unknown<br>Ethnicity unknown	Details unknown	LTS (CC7)<br>Similar to the SSP. Time 88 minutes. Delivered by researcher. Conducted at LTS facility	SSP (CC7)<br>Standard Ainsworth laboratory procedure [Ainsworth (1969)[2]]	(CC7)
Fury *et al.* (1997)[103]<br>USA	$n = 171$<br>Age range 8–8.9 years<br>Gender unknown<br>Ethnicity unknown	Age range 12–37 years at delivery<br>Ethnicity 80% Caucasian	Family drawing modified checklist (CC17)<br>*and*<br>Family Drawing Global Rating Scale (summary scores derived)<br>21-item checklist and eight 7-point scales. Time 20 minutes. Delivered by examiner. Conducted at home	SSP (CC13)<br>Standard Ainsworth laboratory procedure [Ainsworth (1978)[8]]	(CC13) (CC17)
Gleason *et al.* (2011)[95]<br>Romania	$n = 136$<br>Age range unknown<br>Mean age 22 months<br>Gender unknown<br>Ethnicity 53.9% Romanian	Details unknown	DAI (diagnostic interview: indiscriminately social/disinhibited RAD or emotionally withdrawn/inhibited RAD)<br>8-item interview. Time not reported. Delivered by trained interviewer. Location not reported	PAPA (diagnostic interview: RAD, ADHD, disruptive behaviour disorder, major depressive disorder and functional impairment)<br>Diagnostic interview, details not reported. Time not reported. Unclear who administered. Location not reported	
Goldwyn *et al.* (2000)[50]<br>UK	$n = 31$<br>Age unknown<br>Gender unknown<br>Ethnicity unknown	Details unknown	MCAST (CC9)<br>Details not reported. Time not reported. Unknown who administered. Location not reported	SAT (CC8)<br>Details not reported	(CC8) (CC9)

Author, year and country of publication	Participant details: children	Participant details: parents	Test instrument(s) (classification)/tool description and administration	Comparison test(s) (classification)/tool description and administration	Coding classification key (see *Box 1*)
Gurganus (2002)[104]<br><br>USA	*n* = 243<br><br>Age range 4–18 years<br><br>Mean weighted age 8.6 years<br><br>122 males<br><br>Ethnicity unknown	Details unknown	CBRS (CC1)<br><br>Fifty-two items rated on 4-point scales. Time not reported. Caregiver self-report questionnaire. Conducted at home	RADQ (summary score derived)<br><br>Questionnaire details not reported. Time not reported. Caregiver self-report questionnaire. Conducted at home	(CC1)
Head (1997)[105]<br><br>Canada	*n* = 42<br><br>Mean age 6 years, range 5–7 years<br><br>23 males<br><br>Ethnicity unclear	Details unknown	Revised PBAR (CC1)<br><br>One to five drawings. Time 2 hours. Delivered by research assistant. Laboratory setting	SSP (CC22)<br><br>SSP (unclear method reference). Time 21 minutes. Laboratory setting	(CC22) (CC1)
Madigan *et al.* (2003)[25]<br><br>Canada	*n* = 123<br><br>Mean age 7.2 years<br><br>50 males<br><br>Ethnicity unknown	Reported based on infant's previous attachment classification<br><br>Mean maternal age: avoidant = 28.1 years; secure = 29.4 years; resistant = 30.7 years<br><br>Ethnicity unknown	Family Drawing clinical scheme (summary scores derived)<br><br>*and*<br><br>Family Drawing checklist (markers of attachment styles)<br><br>*and*<br><br>Family Drawing Global Rating Scale (CC17)<br><br>*and*<br><br>Family Drawing clinician's opinion (CC3)	SSP (CC3)<br><br>Standard Ainsworth laboratory procedure [Ainsworth (1978)[8]]	(CC3) (CC17)

continued

© Queen's Printer and Controller of HMSO 2015. This work was produced by Wright *et al.* under the terms of a commissioning contract issued by the Secretary of State for Health. This issue may be freely reproduced for the purposes of private research and study and extracts (or indeed, the full report) may be included in professional journals provided that suitable acknowledgement is made and the reproduction is not associated with any form of advertising. Applications for commercial reproduction should be addressed to: NIHR Journals Library, National Institute for Health Research, Evaluation, Trials and Studies Coordinating Centre, Alpha House, University of Southampton Science Park, Southampton SO16 7NS, UK.

TABLE 1 Overview of screening and assessment studies and instrument characteristics (*continued*)

Author, year and country of publication	Participant details: children	Participant details: parents	Test instrument(s) (classification)/tool description and administration	Comparison test(s) (classification)/tool description and administration	Coding classification key (see *Box 1*)
			18-marker clinical scheme, 22-item checklist and 7 items rated on 7-point global rating scale. Time 30 minutes. Delivered by examiner. Location not reported		
Mangelsdorf *et al.* (1996)[106]<br><br>USA	$N = 100$ (complete data $n = 74$)<br><br>Clinical: $n = 34$, 54.1% male, ethnicity 89.2% Caucasian<br><br>Normative: $n = 40$, 40.5% male, ethnicity 95.1% Caucasian<br><br>Aged 14 and 19 months at first and second visits	Clinical group: mean maternal age 27.5 years<br><br>Normative group: mean maternal age 28.9 years<br><br>Ethnicity unknown	SSP (CC11)<br><br>Standard Ainsworth laboratory procedure [Ainsworth (1978)[8]]	AQS<br><br>Waters (1995),[125] 90-item sort. Time 3 hours. Delivered by observers. Conducted at home	(CC11)
Minnis *et al.* (2009);[108] McLaughlin *et al.* (2010)[109]<br><br>UK	$N = 77$<br><br>Age range unknown<br><br>Clinical: $n = 38$, mean age 6.57 years, 66% males<br><br>Normative: $n = 39$, mean age 6.44 years, 67% males<br><br>Ethnicity 100% white British	Details unknown	CAPA-RAD (screening tool for RAD and other diagnosis)<br><br>Twenty-eight items. Time 15–30 minutes. Delivered by interviewer. Location not reported<br><br>*and*<br><br>WRO (screening tool for RAD)<br><br>Yes/no rating on 20 items. Time 15 minutes. Delivered by observer. Location waiting room<br><br>*and*<br><br>RPQ (screening tool for RAD)<br><br>Fourteen items rated on a scale of 0–3. Time not reported. Delivered by teacher. Location not reported	MCAST (CC10)<br><br>Four vignettes rated on a scale of 1–9. Time not reported. Delivered by researcher. Location not reported<br><br>RAD children screened with ICD-10 vs. normative sample	(CC10)

Author, year and country of publication	Participant details: children	Participant details: parents	Test instrument(s) (classification)/tool description and administration	Comparison test(s) (classification)/tool description and administration	Coding classification key (see *Box 1*)
Minnis *et al.* (2010)[107]<br><br>UK	$N = 82$<br><br>Complete data $n = 55$ (33 male)<br><br>Clinical: $n = 28$; normative $n = 27$<br><br>Age range 5–8 years<br><br>Ethnicity unknown	Details unknown	CMCAST (CC10)<br><br>Four stories. Time 22 minutes. Delivered by research assistant. Location not reported	RAD children, screened with ICD-10 vs. normative sample and MCAST (CC10)<br><br>Four stories. Time 17 minutes. Delivered by research assistant. Location not reported	(CC10)
Ogilvie (2000)[110]<br><br>Canada	$N = 303$<br><br>Complete data $n = 285$<br><br>Mean age 12.17 years, range 6–20 years. 151 males<br><br>Ethnicity 53% Caucasian	Age range 20–70 years (two unspecified)<br><br>Ethnicity 59% Caucasian	BERS + BAT (summary scores derived)<br><br>Eighty-five items rated on scale of 0–3<br><br>Time estimated 15 minutes. Caregiver self-report questionnaire. Conducted at home	RADQ (summary score derived)<br><br>Thirty items rated on scale of 1–5. Time estimated 10 minutes. Caregiver self-report questionnaire. Conducted at home	
Oppenheim (1990);[111] Oppenheim (1997)[112]<br><br>USA	$n = 35$<br><br>Mean age 44 months, range 35–58 months<br><br>19 males<br><br>Ethnicity 100% Caucasian	Details unknown	ADI (summary scores derived)<br><br>Six vignettes rated on scales of 1–3 and 1–4. Time 20–40 minutes. Delivered by trained interviewer. Conducted at school	AQS version 3.0<br><br>Waters (1987),[123] 90-item sort. Time 72 hours. Delivered by mother. Conducted at home<br><br>*and*<br><br>Bespoke separation–reunion observation (summary scores derived)<br><br>Seven items rated on scales of 0–4 and 0–3. Time 25–48 minutes. Delivered by teachers and observers. Conducted at school	

continued

© Queen's Printer and Controller of HMSO 2015. This work was produced by Wright *et al.* under the terms of a commissioning contract issued by the Secretary of State for Health. This issue may be freely reproduced for the purposes of private research and study and extracts (or indeed, the full report) may be included in professional journals provided that suitable acknowledgement is made and the reproduction is not associated with any form of advertising. Applications for commercial reproduction should be addressed to: NIHR Journals Library, National Institute for Health Research, Evaluation, Trials and Studies Coordinating Centre, Alpha House, University of Southampton Science Park, Southampton SO16 7NS, UK.

TABLE 1 Overview of screening and assessment studies and instrument characteristics (*continued*)

Author, year and country of publication	Participant details: children	Participant details: parents	Test instrument(s) (classification)/tool description and administration	Comparison test(s) (classification)/tool description and administration	Coding classification key (see *Box 1*)
Posada (2006)[113]<br><br>USA	$n = 45$<br><br>Age range 36–43 months<br><br>25 males<br><br>Ethnicity 44 white	Average maternal age 33.04 years, paternal age 35 years<br><br>Ethnicity 44 white	AQS<br><br>Waters (1995),[125] 90-item sort and 4-scale scores. Time 5–6 hours. Delivered by researchers. Conducted at home	SSP (CC16)<br><br>Standard Ainsworth laboratory procedure [Ainsworth (1978)[8]]	(CC16)
Roman (2010)[114]<br><br>Spain	$N = 148$<br><br>Age range unknown<br><br>Adopted group: $n = 40$, average age 75.68 months, 72.5% male<br><br>Care centre children: $n = 50$, average age 77.60 months, 48% male<br><br>Normative: $n = 58$, average age 75.17 months, 50% male<br><br>Ethnicity unknown	Details unknown	SSAP (markers of attachment styles)<br><br>Thirteen narrative story stems. Time not reported. Delivered by interviewer. Location not reported	IMAS [shortened AQS, Chisholm *et al.* (1999)[126]]<br><br>Twenty-three items. Time not reported. Delivered by interviewer. Location not reported<br><br>*and*<br><br>RPQ (summary scores derived)<br><br>Ten items. Time not reported. Caregiver self-report questionnaire. Conducted at home or foster centre	
Shmueli *et al.* (2008)[52]<br><br>UK	$N = 227$<br><br>Age range unknown<br><br>Clinical: $n = 65$, mean age 10.4 years, 58.5% male, ethnicity 82% white<br><br>Normative: $n = 161$, mean age 10.9 years, 50.3% male, ethnicity 70% white	Details unknown	CAI (CC20)<br><br>Fifteen items rated on scales of 1–9. Time 20–80 minutes. Delivered by interviewer. Location not reported	SAT (CC2)<br><br>Nine pictures. Time not reported. Delivered by experienced interviewers. Location not reported	(CC2) (CC20)

Author, year and country of publication	Participant details: children	Participant details: parents	Test instrument(s) (classification)/tool description and administration	Comparison test(s) (classification)/tool description and administration	Coding classification key (see *Box 1*)
Silver (2005)[115]<br>USA	$N = 233$<br>Complete data $n = 140$<br>Age range unknown<br>Mean age 7 years<br>76 males<br>Ethnicity unknown	Details unknown	Family Drawing checklist (markers of attachment styles)<br>*and*<br>Family Drawing Global Rating Scales (summary scores derived)<br>*and*<br>Family Drawing principal investigator's opinion (CC4)<br>*and*<br>Family Drawing clinician's opinion (CC4)<br>*and*<br>Modified relatedness scales (CC29)<br>Twenty-three-item checklist and six 5-point global scales and 15 items rated on 4-point scales. Time not reported. Delivered by researcher. Conducted at home	SSP (CC4 and CC23)<br>Standard Ainsworth laboratory procedure [Ainsworth (1969)[2]]	(CC4) (CC23) (CC29)
Sirl (1999)[116]<br>USA	$N = 69$<br>Complete data $n = 56$<br>Mean age 6.57 years, range 5.77–7.25 years<br>25 males<br>Ethnicity 100% African American	Details unknown	Modified ASCT<br>Four story stems coding on scale of 0–1 for over 30 socioemotional codes (modified Rochester Narrative coding system). Time not reported. Delivered by examiner. Laboratory setting	SSP with separation–reunion procedure (CC26)<br>Items rated on 7- and 9-point scales. Standard Ainsworth laboratory procedure [Ainsworth (1978)[8]] with separation–reunion procedure [Cassidy and Marvin (1989)[47]]	(CC26)

continued

© Queen's Printer and Controller of HMSO 2015. This work was produced by Wright *et al.* under the terms of a commissioning contract issued by the Secretary of State for Health. This issue may be freely reproduced for the purposes of private research and study and extracts (or indeed, the full report) may be included in professional journals provided that suitable acknowledgement is made and the reproduction is not associated with any form of advertising. Applications for commercial reproduction should be addressed to: NIHR Journals Library, National Institute for Health Research, Evaluation, Trials and Studies Coordinating Centre, Alpha House, University of Southampton Science Park, Southampton SO16 7NS, UK.

TABLE 1 Overview of screening and assessment studies and instrument characteristics (*continued*)

Author, year and country of publication	Participant details: children	Participant details: parents	Test instrument(s) (classification)/tool description and administration	Comparison test(s) (classification)/tool description and administration	Coding classification key (see *Box 1*)
Smeekens *et al.* (2009)[117]<br><br>the Netherlands	*n* = 129<br><br>Complete data *n* = 111<br><br>Age range unknown<br><br>Mean age 63.6 months<br><br>59 males<br><br>Ethnicity unknown	Age range 22–47 years<br><br>Ethnicity unknown	SSSP (CC4)<br><br>Modified Ainsworth procedure [Ainsworth (1978)[7]] (only one separation lasting 4 minutes). Time 10 minutes	AQS version 3.0<br><br>Waters (1995),[125] 90-item sort. Time 2 hours. Delivered by trained observer. Conducted at home	(CC4)
Solomon *et al.* (1995)[24]<br><br>USA	*n* = 69<br><br>Mean age 70.5 months, range 57–94 months<br><br>Gender unknown<br><br>Ethnicity unknown	Reported in groups based on method of recruitment: by telephone (*n* = 17) 18% non-white; by letter (*n* = 52) 21% non-white<br><br>No further details	Adapted separation–reunion story completion task (CC30)<br><br>Five stories. Time 1 hour. Delivered by researcher. Laboratory setting	Separation–reunion episode (A15)<br><br>Details not reported. Time 65 minutes. Delivered by parent and researcher. Laboratory setting	(CC15) (CC30)
Spieker and Crittenden (2010)[118]<br><br>USA	*n* = 306<br><br>Aged 15 and 36 months at first and second visits<br><br>Gender unknown<br><br>Ethnicity unknown	Details unknown	Modified SSP (CC27 and CC19)<br><br>Modified Ainsworth procedure [Ainsworth (1978)[8]] (stranger reunion removed and second separation duration 5 minutes)	SSP (CC4)<br><br>Standard Ainsworth laboratory procedure [Ainsworth (1978)[8]]	(CC4) (CC27) (CC19)
Tarabulsy and Moran (1997)[119]<br><br>Canada	*n* = 79<br><br>Aged 15 and 36 months at first and second visits<br><br>Gender unknown<br><br>Ethnicity unknown	Pre-term mothers, mean age 29 years (SD 4.9). Full-term mothers, mean age 30 years (SD 4.9)<br><br>Ethnicity unknown	AQS<br><br>Waters and Deane (1985),[48] 90-item sort. Time 2–3 hours. Delivered by observers and caregiver. Conducted at home	SSP (CC8)<br><br>Standard Ainsworth laboratory procedure [Ainsworth (1978)[8]]	(CC8)

Author, year and country of publication	Participant details: children	Participant details: parents	Test instrument(s) (classification)/tool description and administration	Comparison test(s) (classification)/tool description and administration	Coding classification key (see *Box 1*)
van Dam and Van IJzendoorn (1988)[120]<br><br>the Netherlands	$n = 39$<br><br>Age range unknown<br><br>Mean age 18 months<br><br>19 males<br><br>Ethnicity unknown	Details unknown	Adapted Parental AQS<br><br>Waters and Deane (1985),[48] 75-item sort. Time not reported. Delivered by research assistants and parents. Location not reported	SSP (CC14)<br><br>Standard Ainsworth laboratory procedure [Ainsworth (1978)[8]]	(CC14)
Vaughn and Waters (1990)[121]<br><br>USA	$n = 58$<br><br>Aged 12 or 18 months at first visit<br><br>25 males<br><br>Ethnicity unknown	Three infants had fathers who were 'non white'<br><br>Further details unknown	SSP (CC3)<br><br>Standard Ainsworth laboratory procedure [Ainsworth (1978)[8]]	AQS<br><br>Waters and Deane (1985),[48] 100-item sort. Time 4–9 hours. Delivered by observer. Conducted at home	(CC3)
Ziegenhein and Jacobsen (1999)[122]<br><br>Germany	$n = 33$<br><br>Aged 12 months, 18 months and 6 years at first, second and third visits, respectively<br><br>17 males<br><br>Ethnicity unknown	$n = 33$<br><br>Ethnicity German	Parent–child separation story (CC9)<br><br>Nine story pictures. Time 1 hour. Unknown who administered. Laboratory setting	SSP (CC9)<br><br>Standard Ainsworth laboratory procedure [Ainsworth (1978)[8]]<br><br>*and*<br><br>Separation–reunion episode (CC9)<br><br>Details not reported. Time 65 minutes. Delivered by parent. Laboratory setting	(CC9)

ADHD, attention deficit hyperactivity disorder; ADI, Attachment Doll Interview; ASCT, Attachment Story Completion Task; BAT, Biopsychosocial Attachment Types; BERS, Behavioural and Emotional Rating Scale; CAP, California Attachment Procedure; CAPA-RAD, Child and Adolescent Psychiatric Assessment – reactive attachment disorder; CBRS, May-Nichols Child Behaviour Rating Scale; CMCAST, Computerised Manchester Child Attachment Story Task; DAI, Disturbances of Attachment Interview; DC: 0–3R, Revised Classification of Mental Health and Developmental Disorders of Infancy and Early Childhood; IMAS, Interview Measure of Attachment Security; LTS, Louisville Twin Study; MCDC, Middle Childhood Disorganisation and Control; MIMRS, Marschak Interaction Method Rating System; PAA, Preschool Assessment of Attachment; PAPA, Preschool Age Psychiatric Assessment; PBAR, Permitting Blocking Access Inventory; RADQ, Randolph Attachment Disorder Questionnaire; RPQ, Relationships Problems Questionnaire; SD, standard deviation; SSSP, Shortened Strange Situation Procedure; WRO, Waiting Room Observation.

© Queen's Printer and Controller of HMSO 2015. This work was produced by Wright *et al.* under the terms of a commissioning contract issued by the Secretary of State for Health. This issue may be freely reproduced for the purposes of private research and study and extracts (or indeed, the full report) may be included in professional journals provided that suitable acknowledgement is made and the reproduction is not associated with any form of advertising. Applications for commercial reproduction should be addressed to: NIHR Journals Library, National Institute for Health Research, Evaluation, Trials and Studies Coordinating Centre, Alpha House, University of Southampton Science Park, Southampton SO16 7NS, UK.

TABLE 2 Taxonomy of screening tools for attachment patterns

Author and year	Instrument
Observational tools	
Boris *et al.* (2004);[98] Clarke-Stewart *et al.* (2001);[93] Finkel *et al.* (1998);[94] Head (1997);[105] Fury *et al.* (1997);[103] Madigan (2003);[25] Posada (2006);[113] Silver (2005);[115] Sirl (1999);[116] Spieker and Crittenden (2010);[118] Tarabulsy and Moran (1997);[119] van Dam and Van IJzendoorn (1988);[120] Mangelsdorf *et al.* (1996);[106] Vaughn and Waters (1990);[121] Fagot and Pears (1996);[102] Ziegenhein and Jacobsen (1999)[122]	SSP
Smeekens *et al.* (2009);[117] Spieker and Crittenden (2010);[118] Fagot and Pears (1996);[102] Crittenden *et al.* (2007);[100] Aber and Baker (1990)[96]	Modified SSP
Clarke-Stewart *et al.* (2001)[93]	CAP
Ziegenhein and Jacobsen (1999);[122] Solomon *et al.* (1995);[24] Cassidy and Marvin (1988)[47]	Separation–reunion procedure
Finkel *et al.* (1998)[94]	LTS procedure
Oppenheim (1990);[111] Oppenheim (1997)[112]	Bespoke separation–reunion observation
Backman (2003)[97]	MIMRS
Boris *et al.* (2004);[98] Backman (2003);[97] Oppenheim (1990);[111] Oppenheim (1997);[112] Posada (2006);[113] Smeekens (2009);[117] Tarabulsy and Moran (1997);[119] Mangelsdorf *et al.* (1996);[106] Vaughn and Waters (1990)[121]	AQS
Aber and Baker (1990);[96] van Dam and Van IJzendoorn (1988)[120]	Modified AQS
Bureau *et al.* (2009)[99]	MCDC scales
Interview: researcher-/clinician-completed	
Shmueli *et al.* (2008)[52]	CAI
Roman (2010)[114]	IMAS
Questionnaire: caregiver-/child-/teacher-completed	
Silver (2005)[115]	Modified relatedness scales
Stories with child response procedure	
Bureau *et al.* (2009);[99] Goldwyn *et al.* (2000);[50] Shmueli (2008)[52]	SAT
Head (1997)[105]	Revised PBAR
Minnis *et al.* (2010)[107]	CMCAST
Minnis *et al.* (2010);[107] Goldwyn *et al.* (2000);[50] Minnis *et al.* (2009);[108] McLaughlin *et al.* (2010)[109]	MCAST
Oppenheim (1990);[111] Oppenheim (1997)[112]	ADI
Sirl (1999)[116]	Modified ASCT
Ziegenhein and Jacobsen (1999)[122]	Parent–child separation story
Solomon *et al.* (1995)[24]	Adapted separation–reunion story completion task
Cassidy and Marvin (1988)[47]	Incomplete stories with doll family
Roman (2010)[114]	SSAP
Family Drawing Procedure	
Fury *et al.* (1997);[103] Madigan *et al.* (2003);[25] Silver (2005)[115]	Family drawing

ADI, Attachment Doll Interview; ASCT, Attachment Story Completion Task; CAP, California Attachment Procedure; CMCAST, Computerised Manchester Child Attachment Story Task; IMAS, Interview Measure of Attachment Security; LTS, Louisville Twin Study; MCDC, Middle Childhood Disorganisation and Control; MIMRS, Marshak Interaction Method Rating System; PBAR, Permitting Blocking Access Inventory.

TABLE 3 Taxonomy of screening tools for attachment disorders

Author and year	Instrument
Observational tools	
Minnis *et al.* (2009);[108] McLaughlin *et al.* (2010)[109]	WRO
Interview: researcher-/clinician-completed	
Boris *et al.* (2004)[98]	DSM-IV criteria
Gleason *et al.* (2011)[95]	DAI
Gleason *et al.* (2011)[95]	PAPA
Equit *et al.* (2011)[101]	DC: 0–3R
Equit *et al.* (2011)[101]	ICD-10
Minnis *et al.* (2009);[108] McLaughlin *et al.* (2010)[109]	CAPA
Questionnaire: caregiver-/child-/teacher-completed	
Gurganus (2002)[104]	CBRS
Gurganus (2002);[104] Ogilvie (2000)[110]	RADQ
Ogilvie (2000)[110]	BERS and BAT
Minnis *et al.* (2009);[108] McLaughlin *et al.* (2010);[109] Roman (2010)[114]	RPQ

BAT, Biopsychosocial Attachment Types; BERS, Behavioural and Emotional Rating Scale; CAPA, Child and Adolescent Psychiatric Assessment; CBRS, May-Nichols Child Behaviour Rating Scale; DAI, Disturbances of Attachment Interview; DC: 0–3R, Revised Classification of Mental Health and Developmental Disorders of Infancy and Early Childhood; LTS, Louisville Twin Study; PAPA, Preschool Age Psychiatric Assessment; RADQ, Randolph Attachment Disorder Questionnaire; RPQ, Relationships Problems Questionnaire; WRO, Waiting Room Observation.

A range of screening and/or diagnostic tools were identified, and the variation in the classification systems used with procedures was large. In relation to the SSP alone, 16 studies reportedly used the original Ainsworth (1969,[2] 1978[8]) procedure,[25,93,94,98,102,103,105,106,113,115,116,118–122] using 12 different variations on the classification system. Confusingly, many different authors use a variety of nomenclature to describe various classifications of attachment patterns. This is summarised in *Table 1* and *Box 1*. Some authors describing subcategories of insecure attachment use different names. For example, anxious–avoidant attachment is sometimes simply called 'avoidant', and sometimes 'defended' or 'dismissing'. Similarly, anxious–ambivalent attachment pattern is sometimes simply called 'ambivalent', and sometimes 'anxious–resistant' or 'preoccupied' (see *Box 1*). In some papers it is not clear whether authors are creating new categories with subtle differences or simply renaming existing categories. Nonetheless, this practice makes the literature extremely confusing to new trainees coming into the field, or indeed any practitioners or researchers wishing to better understand the field of attachment work.

There are two main schools of hierarchicalisation for attachment patterns. *Table 1* and *Box 1* describe classifications used by Ainsworth[8] and extended by Main and Solomon[6] whereby organised attachments may be secure or insecure, with insecure attachments having subcategories. A separate disorganised category exists in this system. By contrast, another body of work led by Crittenden[127] suggests that the disorganised category is actually a subgroup of insecure attachment that is unpredictable or changing in nature. Crittenden's A/C category, sometimes called avoidant/resistant, might be regarded as mapping on to the disorganised attachment described above, but it is understood in different ways. Nevertheless, it is a category of interest in terms of long-term psychopathology and long-term outcomes.

© Queen's Printer and Controller of HMSO 2015. This work was produced by Wright *et al.* under the terms of a commissioning contract issued by the Secretary of State for Health. This issue may be freely reproduced for the purposes of private research and study and extracts (or indeed, the full report) may be included in professional journals provided that suitable acknowledgement is made and the reproduction is not associated with any form of advertising. Applications for commercial reproduction should be addressed to: NIHR Journals Library, National Institute for Health Research, Evaluation, Trials and Studies Coordinating Centre, Alpha House, University of Southampton Science Park, Southampton SO16 7NS, UK.

BOX 1 Coding classification key for the SSP and modifications

CC1: secure, insecure.

CC2: secure F1–5, insecure DS1–2, E1–2.

CC3: secure, avoidant, resistant.

CC4: secure, avoidant, resistant, disorganised.

CC5: secure, insecure, disorganised.

CC6: secure B1–4, avoidant A1–2, resistant C.

CC7: secure B1–4, avoidant A1–2, resistant C1–2.

CC8: secure, avoidant, ambivalent.

CC9: secure, avoidant, ambivalent, disorganised.

CC10: secure, avoidant, resistant/ambivalent, disorganised.

CC11: secure, avoidant, resistant, avoidant/resistant.

CC12: secure, avoidant, resistant, avoidant/resistant, disorganised.

CC13: secure, anxious–avoidant, anxious–resistant.

CC14: secure B1–4, anxious–avoidant A1–2, anxious–resistant C1–2.

CC15: secure, anxious–avoidant, anxious–ambivalent, anxious–controlling, unclassifiable.

CC16: secure, anxious–avoidant, anxious–resistant, anxious–disorganised–controlling, anxious–other.

CC17: anxious–avoidant, anxious–resistant, anxious–insecure.

CC18: secure B1–5, anxious–avoidant A1–6, anxious–resistant C1–6, anxious–avoidant/anxious–resistant A/C.

CC19: secure, resistant C1–4, insecure A1–4, insecure/resistant, anxious–depression, insecure–other R.

CC20: secure, dismissing, preoccupied, disorganised.

CC21: secure/confident, avoidant, hostile–negative (disorganised).

CC22: secure, defended, coercive, defended/coercive.

CC23: secure, defended, coercive, defended/coercive, atypical.

BOX 1 Coding classification key for the SSP and modifications (*continued*)

CC24: secure, avoidant, controlling, ambivalent.

CC25: secure, avoidant, controlling, ambivalent, insecure–other.

CC26: secure, avoidant, dependent, controlling/disorganised.

CC27: secure B1–6, insecure A1–2, resistant C1–2, controlling–punitive, controlling–caregiving, controlling–general, insecure–other D1–4.

CC28: controlling–punitive, controlling–caregiving, disorganised.

CC29: optimal, adequate, confused, disengaged, deprived.

CC30: confident, frightened, casual, busy.

The identified comparison tools employed various techniques to assess children, utilising observational techniques, questionnaires, interviews, stories with child responses and interpretation of a child's drawings. Procedures involved the child, caregiver, teacher, researchers and clinicians depending on the child's age and the tool used. Descriptions of these assessments in full are included in the taxonomy (see *Tables 2* and *3*).

A total of seven papers included tools assessing children with attachment disorders.[95,98,101,104,108,110,114] Tools included DSM-IV, Disturbances of Attachment Interview (DAI), Preschool Age Psychiatric Assessment (PAPA), May-Nichols Child Behaviour Rating Scale (CBRS), Randolph Attachment Disorder Questionnaire (RADQ), Revised Classification of Mental Health and Developmental Disorders of Infancy and Early Childhood (DC: 0–3R), RDC for ICD-10, Behavioural and Emotional Rating Scale (BERS) with Biopsychosocial Attachment Types (BAT), Relationships Problems Questionnaire (RPQ) used in combination with the Child and Adolescent Psychiatric Assessment (CAPA), Waiting Room Observation (WRO) and the RPQ. Relationships Problems Questionnaire (RPQ) used in combination with the Child and Adolescent Psychiatric Assessment (CAPA) and Waiting Room Observation, (WRO) and the RPQ alone.

Population characteristics

Figure 3 summarises the profile of the population studied. *Table 1* includes detail on the participant age, gender and ethnicity.

The age of the children assessed for attachment problems ranged from 12 months to 20 years. We included studies where the mean age was 13 years or below, and therefore in some cases the upper end of the age range exceeded 13 years, as the sample included children older than this but with the average age still below 13 years. Many studies did not report on the child's ethnicity. In studies where ethnicity was recorded, participants were predominantly white or Caucasian. Other ethnic groups were generally under-represented.

Parental/caregiver information was less likely to be described in detail, with 29 studies reporting incomplete data or no detail.[24,25,47,50,52,93–96,99–102,104–108,111–122] In those studies providing demographic detail, parental age ranged from 12 to 70 years and ethnicity was predominantly white or Caucasian.

© Queen's Printer and Controller of HMSO 2015. This work was produced by Wright *et al.* under the terms of a commissioning contract issued by the Secretary of State for Health. This issue may be freely reproduced for the purposes of private research and study and extracts (or indeed, the full report) may be included in professional journals provided that suitable acknowledgement is made and the reproduction is not associated with any form of advertising. Applications for commercial reproduction should be addressed to: NIHR Journals Library, National Institute for Health Research, Evaluation, Trials and Studies Coordinating Centre, Alpha House, University of Southampton Science Park, Southampton SO16 7NS, UK.

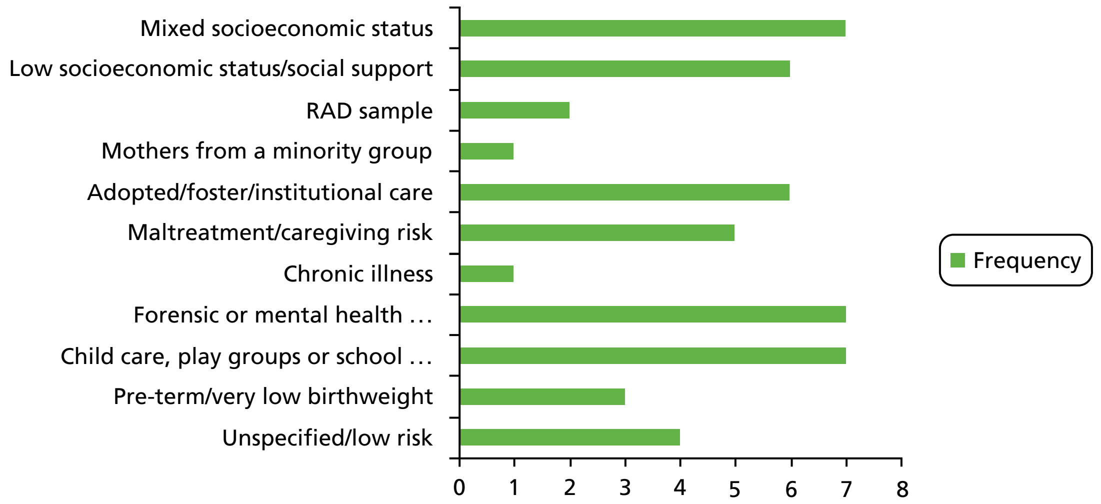

FIGURE 3 Summary of study populations from studies included in supplementary review 1.

Information on the population description was collected and classified into 11 categories (see *Figure 3*). These groupings can be further categorized according to four general dimensions:

- socioeconomic status (SES)
- risk populations (such as fostered, adopted or maltreated children or those in institutional care)
- mental health status of child (e.g. RAD or mental health user)
- groups deemed as low risk or population samples (e.g. playgroup children).

Four studies did not adequately describe the population,[50,117,118,120] for example classifying the population as 'low risk' without further clarification.

No papers reported on child literacy level; however, this is not surprising given that many studies were assessing infants with instruments completed by researchers or parents. A handful of studies did report on other child ability measures such as picture vocabulary and verbal ability.

Quality assessment

Table 4 summarises the results of the quality assessment. High risk of bias was the most frequently reported classification in domain 4 (flow and timing). The lowest risks were in domain 2 (index test) and domain 3 (reference standard). An unclear risk of bias was the most frequently reported classification in domain 1 (patient selection) and in domains 2 and 3.

All studies achieved a low risk of bias on all three applicability questions (*Table 5*) in QUADAS-2.[92] The screening criteria were such that this would inevitably be the outcome.

TABLE 4 Summary of quality assessment for accuracy tool/diagnostic accuracy studies

Study	Patient selection				Index test			Reference test			Flow/timing			
	Consecutive or random sample	Avoided case–control	Avoided inappropriate exclusions	Overall risk of bias	Interpreted blind to reference test	Threshold prespecified	Overall risk of bias	Reference test correctly classifies target condition	Reference test interpreted blind to index test	Overall risk of bias	Interval of 2 weeks or less	All participants receive same reference test	All participants included in analysis	Overall risk of bias
Aber and Baker (1990)[96]	✗	✓	✗	High	✓	N/A	Low	✓	✓	Low	✗	✗	✗	High
Backman (2003)[97]	?	✗	?	High	?	?	Unclear	✓	?	Unclear	✗	✗	✓	High
Boris *et al.* (2004)[98]	?	✗	?	High	✗	N/A	High	✓	✗	High	?	✓	✓	Unclear
Bureau *et al.* (2009)[99]	?	✗	?	High	?	✓	Unclear	✓	?	Unclear	?	✓	✓	Unclear
Cassidy and Marvin (1988)[47]	?	✓	?	Unclear	✓	N/A	Low	✓	✓	Low	✓	✓	✓	Low
Clarke-Stewart *et al.* (2001)[93]	?	✗	?	High	?	N/A	Unclear	✓	?	Unclear	✗	✓	✓	High
Crittenden *et al.* (2007)[100]	?	✓	?	Unclear	✓	N/A	Low	✓	✓	Low	?	✓	?	Unclear
Equit *et al.* (2011)[101]	✓	✓	✓	Low	?	N/A	Unclear	✓	?	Unclear	?	✓	✓	Unclear
Fagot and Pears (1996)[102]	?	✓	✓	Unclear	✓	N/A	Low	✓	✓	Low	✗	✓	?	High
Finkel *et al.* (1998)[94]	?	✓	✓	Unclear	✓	N/A	Low	✓	✓	Low	✓	✓	✓	Low
Fury *et al.* (1997)[103]	?	✓	?	Unclear	?	N/A	Unclear	✓	?	Unclear	✗	?	?	High
Gleason *et al.* (2011)[95]	✓	✓	✓	Low	?	✓	Unclear	?	?	Unclear	?	✓	✓	Unclear
Goldwyn *et al.* (2000)[50]	?	?	?	Unclear	?	N/A	Unclear	✓	✓	Low	?	?	?	Unclear

continued

© Queen's Printer and Controller of HMSO 2015. This work was produced by Wright *et al.* under the terms of a commissioning contract issued by the Secretary of State for Health. This issue may be freely reproduced for the purposes of private research and study and extracts (or indeed, the full report) may be included in professional journals provided that suitable acknowledgement is made and the reproduction is not associated with any form of advertising. Applications for commercial reproduction should be addressed to: NIHR Journals Library, National Institute for Health Research, Evaluation, Trials and Studies Coordinating Centre, Alpha House, University of Southampton Science Park, Southampton SO16 7NS, UK.

TABLE 4 Summary of quality assessment for accuracy tool/diagnostic accuracy studies (*continued*)

Study	Patient selection				Index test			Reference test			Flow/timing			
	Consecutive or random sample	Avoided case–control	Avoided inappropriate exclusions	Overall risk of bias	Interpreted blind to reference test	Threshold prespecified	Overall risk of bias	Reference test correctly classifies target condition	Reference test interpreted blind to index test	Overall risk of bias	Interval of 2 weeks or less	All participants receive same reference test	All participants included in analysis	Overall risk of bias
Gurganus (2002)[104]	?	✓	?	Unclear	?	✓	Unclear	✓	?	Unclear	?	?	?	Unclear
Head (1997)[105]	?	✓	?	Unclear	✓	✓	Low	✓	✓	Low	✓	✓	✓	Low
Madigan (2003)[25]	?	?	?	Unclear	✓	N/A	Low	✓	✓	Low	✗	?	?	Unclear
Mangelsdorf *et al.* (1996)[106]	?	✗	?	High	✓	N/A	Low	✓	✓	Low	✓	?	✗	High
Minnis *et al.* (2010)[107]	✓	✗	✓	High	✓	N/A	Low	✓	✓	Low	✗	✓	✗	High
Minnis *et al.* (2009);[108] McLaughlin *et al.* (2010)[109]	?	✗	?	High	?	N/A	Unclear	✓	?	Unclear	?	✗	✗	High
Ogilvie (2000)[110]	?	✓	?	Unclear	?	✓	Unclear	✓	?	Unclear	?	✓	✓	Unclear
Oppenheim (1990)[111] and Oppenheim (1997)[112]	?	✓	?	Unclear	?	N/A	Unclear	✓	?	Unclear	✗	?	?	Unclear
Posada (2006)[113]	?	✓	?	Unclear	?	N/A	Unclear	✓	✓	Low	?	✓	✓	Unclear
Roman (2010)[114]	?	✗	?	High	?	N/A	Unclear	✓	?	Unclear	?	✓	?	Unclear
Shmueli *et al.* (2008)[52]	?	✗	?	High	✓	✓	Low	✓	✓	Low	?	?	?	Unclear
Silver (2005)[115]	?	✓	?	Unclear	?	N/A	Unclear	✓	✓	Low	✗	✓	✗	High
Sirl (1999)[116]	?	✓	?	Unclear	?	N/A	Unclear	✓	?	Unclear	?	✓	✗	High
Smeekens *et al.* (2009)[117]	?	✓	?	Unclear	?	N/A	Unclear	✓	?	Unclear	?	?	?	Unclear

Study	Patient selection				Index test			Reference test			Flow/timing			
	Consecutive or random sample	Avoided case–control	Avoided inappropriate exclusions	Overall risk of bias	Interpreted blind to reference test	Threshold prespecified	Overall risk of bias	Reference test correctly classifies target condition	Reference test interpreted blind to index test	Overall risk of bias	Interval of 2 weeks or less	All participants receive same reference test	All participants included in analysis	Overall risk of bias
Solomon *et al.* (1995)[24]	?	✓	?	Unclear	✓	N/A	Low	✓	✓	Low	✓	✓	✓	Low
Spieker and Crittenden (2010)[118]	?	✓	?	Unclear	?	N/A	Unclear	✓	?	Unclear	✗	✓	?	High
Tarabulsy and Moran (1997)[119]	?	✗	?	High	?	N/A	Unclear	✓	?	Unclear	✗	✓	?	High
van Dam and Van IJzendoorn (1988)[120]	?	✓	?	Unclear	?	N/A	Unclear	✓	?	Unclear	✓	✓	✓	Low
Vaughn and Waters (1990)[121]	?	✓	?	Unclear	✗	N/A	High	✓	✗	High	✗	✓	?	High
Ziegenhein and Jacobsen (1999)[122]	✓	✓	?	Unclear	✓	N/A	Low	✓	✓	Low	✗	?	✗	High

✓, criterion met; ✗, criterion not met; ?, unclear whether or not criterion met; N/A, not applicable.

© Queen's Printer and Controller of HMSO 2015. This work was produced by Wright *et al.* under the terms of a commissioning contract issued by the Secretary of State for Health. This issue may be freely reproduced for the purposes of private research and study and extracts (or indeed, the full report) may be included in professional journals provided that suitable acknowledgement is made and the reproduction is not associated with any form of advertising. Applications for commercial reproduction should be addressed to: NIHR Journals Library, National Institute for Health Research, Evaluation, Trials and Studies Coordinating Centre, Alpha House, University of Southampton Science Park, Southampton SO16 7NS, UK.

TABLE 5 Ratings on applicability questions of QUADAS-2 for studies included in supplementary review 1

Study	Patient selection: applicability	Index test: applicability	Reference test: applicability
Aber and Baker (1990)[96]	Low	Low	Low
Backman (2003)[97]	Low	Low	Low
Boris *et al.* (2004)[98]	Low	Low	Low
Bureau *et al.* (2009)[99]	Low	Low	Low
Cassidy and Marvin (1988)[47]	Low	Low	Low
Clarke-Stewart (2001)[93]	Low	Low	Low
Crittenden *et al.* (2007)[100]	Low	Low	Low
Equit *et al.* (2011)[101]	Low	Low	Low
Fagot and Pears (1996)[102]	Low	Low	Low
Finkel *et al.* (1998)[94]	Low	Low	Low
Fury *et al.* (1997)[103]	Low	Low	Low
Gleason *et al.* (2011)[95]	Low	Low	Low
Goldwyn *et al.* (2000)[50]	Low	Low	Low
Gurganus (2002)[104]	Low	Low	Low
Head (1997)[105]	Low	Low	Low
Madigan (2003)[25]	Low	Low	Low
Mangelsdorf *et al.* (1996)[106]	Low	Low	Low
Minnis *et al.* (2009);[108] McLaughlin *et al.* (2010)[109]	Low	Low	Low
Minnis *et al.* (2010)[107]	Low	Low	Low
Ogilvie (2000)[110]	Low	Low	Low
Oppenheim (1990)[111] and Oppenheim (1997)[112]	Low	Low	Low
Posada (2006)[113]	Low	Low	Low
Roman (2010)[114]	Low	Low	Low
Shmueli (2008)[52]	Low	Low	Low
Silver (2005)[115]	Low	Low	Low
Sirl (1999)[116]	Low	Low	Low
Smeekens *et al.* (2009)[117]	Low	Low	Low
Solomon (1995)[24]	Low	Low	Low
Spieker and Crittenden (2010)[118]	Low	Low	Low
Tarabulsy and Moran (1997)[119]	Low	Low	Low
van Dam and Van IJzendoorn (1988)[120]	Low	Low	Low
Vaughn and Waters (1990)[121]	Low	Low	Low
Ziegenhein and Jacobsen (1999)[122]	Low	Low	Low

The three applicability questions in QUADAS-2 are:

- Is there concern that the included patients do not match the review question?
- Is there concern that the index test, its conduct or its interpretation differ from the review question?
- Is there concern that the target condition as defined by the reference standard does not match the review question?

Taxonomy of instruments assessing attachment patterns

The findings of the review are split into tools assessing (1) the construct of child attachment to the caregiver, described as an attachment pattern or an attachment security score, and (2) the symptomatology of attachment disorder as defined by the DSM-IV[56] or ICD-10.[55]

Observational tools

Instruments assessing attachment patterns seek to describe the attachment relationship between the child and primary caregiver. As described in *Chapter 1*, the original assessment method was the SSP developed by Ainsworth and Wittig,[2] with other additional methods of assessment being developed from this. The following describes the tools emerging from our review.

The Strange Situation Procedure

The 'Strange Situation'[2] identifies patterns of attachment that infants between the ages of 12 and 18 months form to their mothers. The procedure to elicit these consists of 3-minute intervals of separation and reunion of an infant with the mother/primary caregiver (not a staff member) and the introduction of a stranger. Interactions are coded according to behaviour at reunion in the context of the level of exploratory behaviour and distress on separation exhibited. Ainsworth described the types of attachment children had to their mothers, defining these as secure (type B) or insecure, including the subtypes anxious–ambivalent (type C) and anxious–avoidant (type A).[2] Securely attached infants tend to use the primary caregiver as a secure base for exploration. They clearly prefer the caregiver to the stranger, and demonstrate protest behaviours when separated from their primary caregiver. Anxious–avoidant infants explore without using the primary caregiver as a secure base. They appear unaffected at separation, and avoid or ignore the caregiver when reunited. They tend to treat the stranger in a similar way to the caregiver. Anxious–ambivalent infants refuse to explore their surroundings and become extremely anxious when separated. During reunion, they look for contact with their primary caregiver, but they may also pull away in anger. This group also resists both comfort and contact with the stranger. Classification is based on the infant's behaviour towards the caregiver during the two reunion episodes, viewed in the context of behaviour throughout the whole procedure. From this work, the researchers attempted to predict the impact which different types of attachment pattern had on the child's behaviour and psychological well-being.[8]

This procedure has been expanded by Main and Solomon,[44] who was involved in a variety of studies that assisted in the development of the ABC + D model. Main and Weston[128] identified that some of the children (12.5%) assessed between 12 and 18 months of age in Ainsworth's SSP were termed 'unclassifiable', in that they showed conflict and little positive responsiveness to the adult. This led them to assess and interpret the data collected on these 'unclassifiable' children. From this, they developed and validated a new attachment pattern, labelled disorganised/disoriented (type D).[6]

A total of 16 studies identified in this supplementary review used the SSP.[25,93,94,98,102,103,105,106,113,115,116,118–122] Of these, six used the original ABC classification system[25,93,94,103,105,106] and 10 included the disorganised category in some form.[98,102,113,115,116,118–122] This procedure has been very influential in the design of subsequent assessment tools, as evidenced by the development of many bespoke and modified versions of the procedure found in this review.

© Queen's Printer and Controller of HMSO 2015. This work was produced by Wright *et al.* under the terms of a commissioning contract issued by the Secretary of State for Health. This issue may be freely reproduced for the purposes of private research and study and extracts (or indeed, the full report) may be included in professional journals provided that suitable acknowledgement is made and the reproduction is not associated with any form of advertising. Applications for commercial reproduction should be addressed to: NIHR Journals Library, National Institute for Health Research, Evaluation, Trials and Studies Coordinating Centre, Alpha House, University of Southampton Science Park, Southampton SO16 7NS, UK.

Modified Strange Situation Procedure

The assessments classified as ‘modified SSP’ encompass adaptations to the original procedure by which it was shortened[117] or adapted to include one reunion episode,[102,118] or the separation time was extended.[118]

Often these modifications were to adapt the assessment for use with different age groups[100,102,118] and used different classification systems and terminology. Two studies used Crittenden *et al*'s Preschool Assessment of Attachment (PAA).[100,118] There are five subclassifications: the traditional secure category (B) and two insecure classifications (defended and coercive), as well as a D classification and an A–C classification. One study used the MacArthur Working Group on Attachment classification system.[47,118] There are six subclassifications: secure (type B); insecure–avoidant (type A); insecure–ambivalent (type C); and a D classification that consists of insecure–other, controlling–punitive and controlling–general classifications. The final classification system used an Ainsworth Extended Procedure.[100] There are four subclassifications for this procedure: secure (type B), insecure–avoidant (type A), insecure–resistant (type C) and insecure–avoidant/resistant (type A/C).

Separation Reunion Procedure

Assessments under this classification include only a separation and reunion episode conducted in various settings.[24,47,122] These were conducted in a classroom as part of a ‘preparation for preschool’ meeting[47] or in a laboratory setting.[122] The time period of the separation (before reunion) in the two studies,[24,122] where it was stated, was approximately 1 hour. Different classification systems were used, including the Main and Cassidy classification system.[129] This procedure uses the first 5 minutes of playroom reunion with the mother to allocate an attachment classification. The four main classifications are recognised (A, B, C, D), with the addition of a U classification for those that are ‘unclassifiable’ for some studies.[24] Typically, children in the U group show some elements of behavioural disorganisation and controlling behaviour. The time period of the separation (before reunion) varied between 2 minutes[96] and 1 hour.[24,122]

California Attachment Procedure

Designed to overcome the limitation that the SSP overdiagnoses children who routinely spend more than 20 hours per week in day care as insecure–avoidant,[93] the California Attachment Procedure (CAP)[93] is an alternative laboratory-based method. Instead of using separation from the caregiver, a series of anxiety-provoking situations followed by brief recovery periods (2–3 minutes) are experienced by the child. Anxiety-provoking situations for the child include a noisy hidden toy entering the room or a toy robot, and the presence of a costumed adult stranger. The coding draws heavily on Ainsworth’s original SSP, which produces ABC classifications.

Louisville Twin Study procedure

The Louisville Twin Study (LTS) procedure[94] is a modified version of the SSP, adapted to explore the attachment behaviour of twins at the age of 18–24 months. Similar to the SSP, each twin experiences two separations and reunions with the primary caregiver. During the first separation in the LTS procedure, the twins are together with two strangers, whereas during the second separation the twins are alone with a stranger. There are three distinct differences between the LTS procedure and the SSP. First, the twin is present during the first separation and during reunions. Second, during separations the play vignettes led by the strangers are scripted. Finally, the entire procedure is significantly longer (a 30-minute separation) in the LTS procedure. In our reviewed study,[94] only the second reunion for each twin – after being separated from both twin and caregiver – was coded for attachment behaviour which formed the primary basis for attachment classification.

Marshak Interaction Method Rating System

The Marchak Interaction Method Rating System (MIMRS)[97] is a clinical tool which uses structured observation of the interaction between the parent and child (parent–child dyad) to assess the quality of their relationship for purposes of parent guidance and treatment planning. Clinically, it is often used in conjunction with Theraplay® treatment. The MIMRS was developed by psychologists, using attachment theory and research by Ainsworth,[2] in an attempt to provide a reliable and valid measurement system for the Marshak Interaction Method.

Attachment Q-set

The AQS[48] utilises Q-sort methodology. It consists of 100 behavioural descriptions intended to cover the spectrum of attachment-related behaviours, including the secure base and exploratory behaviours, affective responses and social cognition of children between 12 and 48 months of age. The items are sorted into nine piles according to a predefined distribution to provide a summary of an infant's attachment-related behaviour as observed during 2- to 3-hour home visits. Q-sort observers thus describe the infant's behaviour in terms of an array of 100 scores. Items most characteristic of the child are placed at one end of the distribution, and those most uncharacteristic of the child are placed at the opposite end. Items that cannot be scored from the visit or are neither characteristic nor uncharacteristic of the child are placed in the central piles. An item's placement in the sort determines its score. The most characteristic items thus receive scores of 9. The items most unlike the child receive scores of 1. Attachment secure base behaviour is assessed on a continuum of security rather than categorically. Van IJzendoorn and colleagues[130] researched the reliability and validity of the AQS in a series of meta-analyses that included 13,835 children. The observer AQS security score showed convergent validity with SSP security ($r = 5.31$) and excellent predictive validity with parental sensitivity measures ($r = 5.39$). Its association with temperament was weaker ($r = 5.16$), which supports the discriminant validity of the observer AQS.

Modified Attachment Q-sort

Two studies included modified versions of the AQS. Aber and Baker[96] modified the measure to investigate the attachment security in naturally occurring separations when the child was entering a child care programme. The AQS was adapted to eliminate items that referenced behaviours only observable in the home, so that only items that were relevant to the behaviours displayed by the child at the child care centre were included. The authors also sought to make the tool usable by novices of attachment theory and child care work.

van Dam and Van IJzendoorn[120] translated a parental version of the AQS (containing 75 items) into Dutch. They altered the wording of items, removing double negatives to avoid confusing the parents. The criterion sorts were for 12-month-old children.

Middle Childhood Disorganisation and Control scales

Following a separation–reunion procedure in a laboratory setting, the Middle Childhood Disorganisation and Control (MCDC) scales can be used to rate the extent of three dimensions of children's behaviour towards their caregiver: controlling–caregiving, controlling–punitive and disorganised behaviour. The interactions in the reviewed study[99] were observed in a 5-minute reunion following a 1-hour separation, during which both child and caregiver were interviewed by examiners. The scales range from 1–9. The three dimensions of the child's behaviour are rated on separate scales. For the high range of the controlling–punitive scale, behaviour is marked by episodes of hostility towards the parent that include a challenging, humiliating, cruel or defying quality. Behaviour in the low range of the scale expresses annoyance, frustration or impatience towards the caregiver.

The high range of the controlling–caregiving scale is characterised by the child taking charge of the interaction. The low range of the scale includes minor indications of caregiving behaviour with the motivation of modifying affect, stimulating or distracting the parent. Evidence of the child subordinating his or her own desires and prioritising the parent's needs are also coded on this scale. The disorganised behaviour scale has eight categories that are rated as either high or low (such as manifestation of fear in the presence of the caregiver, lack of consistent strategy, preference for stranger and so on). The combination of high and low disorganised behaviour scores leads to an overall rating of disorganisation on a scale of 1–9. A score of 1 is assigned to a child who shows no signs of disorganisation.

© Queen's Printer and Controller of HMSO 2015. This work was produced by Wright *et al.* under the terms of a commissioning contract issued by the Secretary of State for Health. This issue may be freely reproduced for the purposes of private research and study and extracts (or indeed, the full report) may be included in professional journals provided that suitable acknowledgement is made and the reproduction is not associated with any form of advertising. Applications for commercial reproduction should be addressed to: NIHR Journals Library, National Institute for Health Research, Evaluation, Trials and Studies Coordinating Centre, Alpha House, University of Southampton Science Park, Southampton SO16 7NS, UK.

Interview: researcher-/clinician-completed

The Child Attachment Interview

The CAI[52] is a 19-question, semistructured interview that assesses children's mental representations of attachment figures. The CAI interview includes questions about children's experiences with memories and perceptions of their caregivers. These focus on situations in which the attachment system is presumed to be activated (e.g. emotional upset, illness, injury, separation).

In addition to reporting on what generally happens between the parent and child in response to these situations, the child is also asked to recall a specific occurrence. This enables detailed narratives about the relationships with attachment figures to emerge. The CAI is based on the AAI, and therefore, it assesses the affective nature of the relationship and the quality of the child's response. As with other interviews, it is videotaped for coding. Research suggests that the interview works with children aged 8–12 years.[131] Concurrent validity of the CAI is suggested by a significant association with other measures (SAT) and by a significant association with parental status according to the AAI.[132]

Interview Measure of Attachment Security

The Interview Measure of Attachment Security (IMAS) is an abbreviated version of the AQS[133] applied in an interview format developed by Chisholm and colleagues.[134,135] There are 23 items that evaluate the security of the attachment behaviours that the child presents to the caregiver.

Stories with a child response procedure

The Separation Anxiety Test

The SAT[136] is a semiprojective representational test in which children are shown a number of pictures depicting separations between a child and his or her parent(s). The child is asked a series of questions designed to elicit emotional narratives. Following this, the child's responses are coded according to criteria for securely attached, self-reliant and avoidant responses. The original SAT[136] was used with adolescents and has been adapted and revised over the years by authors including Klagsbrun and Bowlby,[137] for use with 4- to 7-year-olds, and Slough and Greenberg,[138] who scored the SAT based on four attachment scales.

Manchester Child Attachment Story Task

The MCAST is a doll-play story stem technique which seeks to measure attachment patterns in middle childhood.[50] Children between the ages of 5 and 7 years are given the beginnings of four stories ('story stems') using a doll's house, each containing an attachment-related theme: the child waking following a nightmare; the child injuring him/herself; the child becoming ill or lost while out shopping. The interviewer will play out the scenario initially until the child becomes interested and involved; at this point the interviewer asks the child 'what happens next?'. The assessment is recorded and how the child plays out the story thereafter is coded based on both Strange Situation and AAI codes, and the child is assigned an attachment classification.[50] The MCAST has good inter-rater reliability and stability of attachment patterns over time.[50]

Computerised Manchester Child Attachment Story Task

Minnis and colleagues[107] developed a computerised version of the MCAST, the Computerised Manchester Child Attachment Story Task (CMCAST). In this assessment tool, the narrated story stems are initially represented on the computer by the movement of simple two-dimensional screen 'dolls'. The child is then instructed to take over the controls of the computer, moving the dolls and providing a vocal narrative for each story. This is recorded, providing a downloadable audiovisual presentation of the child's story to be used for rating.

Attachment Doll Interview

The Attachment Doll Interview (ADI)[111,112] uses story stems in which children are portrayed as being in distress by an interviewer who begins a story using doll enactments and asks the child to complete the story. It was designed to measure three dimensions, which the authors consider to be markers of security of attachment. These are the quality of mother–child interaction presented in the story completions, the child's ability to talk openly about conflict and emotionally charged themes, and his or her ability to generate constructive resolutions for separations and stressful situations.[111,112]

Family Drawing Procedure

In the Family Drawing Procedure[25,103] children are asked to draw a picture of their family. Each drawing is coded in three ways, according to a scheme adapted from the Kinetic Family Drawings manual:[139] (1) each figure included in the drawing is scored on a variety of markers (e.g. presence/absence of family members, number of body parts, facial affect, location and size of figures); (2) the relations between figures are scored on three markers (e.g. presence/absence of barriers between figures, relative orientation, encapsulation of figures); and (3) the general context of the drawing is scored on five markers (e.g. use of colour, space and perspective).

Other known measures

Below are descriptions of some well-known assessments that were not included in the review as they did not meet the PICOS criteria for this phase of the review (for example they may not have had a comparison tool).

The MacArthur Story Stem Battery

The MSSB[140] is usually used with children aged 4–8 years and uses doll play to assess children's representations of relationships. The process of this includes telling a child the scripted stem of a story, using simple dolls as props.

The child is asked to 'show and tell' the clinician 'what happens next'. The child's completion of each scenario is recorded on video and analysed later by a trained evaluator using a scoring template. There are between 8 and 12 scenarios used, each stem depicting a range of moral and relationship dilemmas.

This tool has been used widely in both clinical work and research, including studies of the internal representations of children from normative samples,[141] maltreated children,[142] children exposed to parental conflict[143] and children with disruptive behavioural disorders.[144] It has been shown to predict behaviour problems[144] and anxiety in children.[140,145]

Taxonomy of assessment for attachment disorder

Observational tools

Preschool Age Psychiatric Assessment

The PAPA[95] is a parent-report-only assessment focused on children aged 2–5 years. Derived from the CAPA, it is tailored to feelings and behaviours pertinent to young children. Based on responses to the PAPA, an algorithm generates diagnoses, scale scores and scores reflecting the number of domains in which the child is impaired. For the study by Gleason and colleagues,[95] DSM-IV criteria were applied for all diagnoses except RAD, for which the RDC for preschool age were used. The test–retest reliability of the PAPA is similar to the reliability of structured psychiatric interviews focused on older children and adults.[95]

Psychometric properties

Table 6 summarises the psychometric properties of the index and reference tests and reports the outcome of screening for content and construct evidence.

With the exception of two studies,[94,119] all publications reported at least partial reliability data.

© Queen's Printer and Controller of HMSO 2015. This work was produced by Wright *et al.* under the terms of a commissioning contract issued by the Secretary of State for Health. This issue may be freely reproduced for the purposes of private research and study and extracts (or indeed, the full report) may be included in professional journals provided that suitable acknowledgement is made and the reproduction is not associated with any form of advertising. Applications for commercial reproduction should be addressed to: NIHR Journals Library, National Institute for Health Research, Evaluation, Trials and Studies Coordinating Centre, Alpha House, University of Southampton Science Park, Southampton SO16 7NS, UK.

TABLE 6 Psychometric properties of the index and reference test(s)

Author and year (index or reference test) for included study	Instrument	Internal consistency (Cronbach's alpha)	Inter-rater reliability measures	Stability (test–retest: Pearson product moment correlation)	Evidence of content validity (yes/no)	Evidence of construct validity (yes/no)
Aber and Baker (1990)[96]<br><br>(Index tool)	Modified SSP	Secure communication scale $\alpha = 0.75$<br><br>Flexible attention–deployment strategy scale $\alpha = 0.63$<br><br>Separation insecurity scale $\alpha = 0.61$<br><br>Reunion rejection scale $\alpha = 0.60$	Unclear number of coders<br><br>Agreement on scales between expert and coding team > 85%	N/R	No	Yes
Aber and Baker (1990)[96]<br><br>(Reference tool)	Teacher-sorted toddler AQS	N/R	Tri-coded<br><br>Coders' correlation on sort = 0.71<br><br>Reliability exceeded 0.60 for 87% of children	N/R	No	Yes
Backman (2003)[97]<br><br>(Index tool)	MIMRS	Total scale $\alpha = 0.96$<br><br>Parent scale $\alpha = 0.90$<br><br>Child scale $\alpha = 0.89$<br><br>Relational/emotional scale $\alpha = 0.89$<br><br>*(Alphas calculated with the addition of data from another study)*[146]	Clinical group dual coded; coders' correlation = 0.88 ($p < 0.001$)<br><br>Normative group dual coded; coders' correlation = 0.82 ($p < 0.001$)	N/R	No	Yes
Backman (2003)[97]<br><br>(Reference tool)	AQS	Prior research has shown an internal consistency score of 0.93	N/R	N/R	No	Yes

Author and year (index or reference test) for included study	Instrument	Internal consistency (Cronbach's alpha)	Inter-rater reliability measures	Stability (test–retest: Pearson product moment correlation)	Evidence of content validity (yes/no)	Evidence of construct validity (yes/no)
Boris *et al.* (2004)[98]<br><br>(Index tool)	Clinical assessment using the DSM-IV criteria screening for presence/absence of attachment disorders	N/R	Tri-coded<br><br>Agreement on presence/absence of attachment disorder κ range 0.62–0.74<br><br>Agreement on no disorder, attachment disorder with role reversal or other attachment disorder 54–73%, κ range 0.44–0.61<br><br>Agreement on no disorder (including attachment disorder with RR) or other attachment disorder κ range 0.57–0.76	N/R	No	Yes
Boris *et al.* (2004)[98]<br><br>(Reference tool)	SSP (A5)	N/R	Dual coded<br><br>Agreement on three-way classification 71%	N/R	No	Yes
Boris *et al.* (2004)[98]<br><br>(Reference tool)	AQS	N/R	Dual coded<br><br>κ average 0.77, range 0.48–0.92	N/R	No	Yes
Bureau *et al.* (2009)[99]<br><br>(Index tool)	MCDC (A28)	N/R	Unclear number of coders<br><br>Coders' correlation on punitive scale = 0.97<br><br>Coders' correlation on caregiving scale = 0.93<br><br>Coders' correlation on disorganisation scale = 0.83	N/R	No	Yes

continued

© Queen's Printer and Controller of HMSO 2015. This work was produced by Wright *et al.* under the terms of a commissioning contract issued by the Secretary of State for Health. This issue may be freely reproduced for the purposes of private research and study and extracts (or indeed, the full report) may be included in professional journals provided that suitable acknowledgement is made and the reproduction is not associated with any form of advertising. Applications for commercial reproduction should be addressed to: NIHR Journals Library, National Institute for Health Research, Evaluation, Trials and Studies Coordinating Centre, Alpha House, University of Southampton Science Park, Southampton SO16 7NS, UK.

TABLE 6 Psychometric properties of the index and reference test(s) (*continued*)

Author and year (index or reference test) for included study	Instrument	Internal consistency (Cronbach's alpha)	Inter-rater reliability measures	Stability (test–retest: Pearson product moment correlation)	Evidence of content validity (yes/no)	Evidence of construct validity (yes/no)
Bureau *et al.* (2009)[99]<br><br>(Reference tool)	SAT (A9)	N/R	Dual coded<br><br>Agreement on four-way classification $\kappa = 0.92$	N/R	No	Yes
Cassidy and Marvin (1988)[47]<br><br>(Index tool)	Incomplete stories with doll family (A21)	Summary score $\alpha = 0.78$	Dual coded<br><br>Agreement on five-point scale 92% (range 76–100%)<br><br>Agreement on three-way classification 86% (range 76–94%)	Retest 1 month apart (1 story)<br><br>Rating correlation = 0.63 ($p < 0.001$)	No	Yes
Cassidy and Marvin (1988)[47]<br><br>(Reference tool)	Separation–reunion episode (A24)	N/R	Dual coded<br><br>Agreement on four-way classification 76%, $\kappa = 0.59$<br><br>Coders' correlation on security scale = 0.80	N/R	No	Yes
Clarke-Stewart *et al.* (2001)[93]<br><br>(Index tool)	CAP (A6)	N/R	Dual coded<br><br>Agreement on three-way classification 75%	N/R	No	Yes
Clarke-Stewart *et al.* (2001)[93]<br><br>(Reference tool)	SSP (A6)	N/R	Dual coded<br><br>Agreement on three-way classification 83%, $\kappa = 0.69$	N/R	No	Yes
Crittenden *et al.* (2007)[100]<br><br>(Index tool)	Modified SSP – Ainsworth extended method (A11)	N/R	Dual coded<br><br>91% agreement, $\kappa = 0.83$ ($p < 0.000$)	N/R	No	Yes

Author and year (index or reference test) for included study	Instrument	Internal consistency (Cronbach's alpha)	Inter-rater reliability measures	Stability (test–retest: Pearson product moment correlation)	Evidence of content validity (yes/no)	Evidence of construct validity (yes/no)
Crittenden *et al.* (2007)[100] (Reference tool)	Modified SSP-CM method (A26)	N/R	Quad-coded<br>78% agreement, $\kappa = 0.72$ ($p < 0.01$)	N/R	No	Yes
	Modified SSP-PAA method (A22)	N/R	Tri-coded<br>86% agreement, $\kappa = 0.82$ ($p < 0.000$)	N/R	No	Yes
Equit *et al.* (2011)[101] (Index tool)	DC: 0–3R	N/R	Dual coded<br>Coders' correlation on diagnosis = 0.75–0.90	N/R	No	Yes
Equit *et al.* (2011)[101] (Reference tool)	ICD-10	N/R	N/R	N/R	No	Yes
Fagot *et al.* (1996)[102] (Index tool)	Modified SSP (A12)	N/R	Dual coded<br>Agreement on five-way classification 84%	N/R	No	Yes
Fagot and Pear (1996)[102] (Reference tool)	SSP (A7)	N/R	Dual coded<br>Approximately 12% disagreement on eight-way classification	N/R	No	Yes
Finkel *et al.* (1998)[94] (Index tool)	LTS (A7)	N/R	N/R	N/R	No	No
Finkel *et al.* (1998)[94] (Reference tool)	SSP (A7)	N/R	N/R	N/R	No	Yes

continued

© Queen's Printer and Controller of HMSO 2015. This work was produced by Wright *et al.* under the terms of a commissioning contract issued by the Secretary of State for Health. This issue may be freely reproduced for the purposes of private research and study and extracts (or indeed, the full report) may be included in professional journals provided that suitable acknowledgement is made and the reproduction is not associated with any form of advertising. Applications for commercial reproduction should be addressed to: NIHR Journals Library, National Institute for Health Research, Evaluation, Trials and Studies Coordinating Centre, Alpha House, University of Southampton Science Park, Southampton SO16 7NS, UK.

TABLE 6 Psychometric properties of the index and reference test(s) (*continued*)

Author and year (index or reference test) for included study	Instrument	Internal consistency (Cronbach's alpha)	Inter-rater reliability measures	Stability (test–retest: Pearson product moment correlation)	Evidence of content validity (yes/no)	Evidence of construct validity (yes/no)
Fury *et al.* (1997)[103]<br><br>(Index tool)	Family Drawing Modified Checklist (A17)	N/R	Dual coded<br><br>Agreement on presence/absence of signs 44–100%	N/R	No	Yes
Fury *et al.* (1997)[103]<br><br>(Index tool)	Family Drawing Global Rating Scale	N/R	Dual coded<br><br>Coders' correlation on scale ratings = 0.57–0.90	N/R	Yes	Yes
Fury *et al.* (1997)[103]<br><br>(Reference tool)	SSP (A13)	N/R	N/R	N/R	No	Yes
Gleason *et al.* (2011)[95]<br><br>(Index tool)	DAI	At baseline, 30, 42, 54 months<br><br>Indiscriminately social/disinhibited RAD signs $\alpha = 0.68, 0.68, 0.72, 0.75$<br><br>Emotionally withdrawn/inhibited RAD signs $\alpha = 0.69, 0.70, 0.70, 0.84$	Unclear number of coders<br><br>Agreement on RAD signs $\kappa = 0.80$	N/R	No	Yes
Gleason *et al.* (2011)[95]<br><br>(Reference tool)	PAPA	N/R	N/R	N/R	No	Yes
Goldwyn *et al.* (2000)[50]<br><br>(Index tool)	MCAST (A9)	N/R	N/R	N/R	No	Yes
Goldwyn *et al.* (2000)[50]<br><br>(Reference tool)	SAT (A8)	N/R	N/R	N/R	No	Yes

Author and year (index or reference test) for included study	Instrument	Internal consistency (Cronbach's alpha)	Inter-rater reliability measures	Stability (test–retest: Pearson product moment correlation)	Evidence of content validity (yes/no)	Evidence of construct validity (yes/no)
Gurganus (2002)[104] (Index tool)	CBRS (A1)	Analysis of 'modified' CBRS only<br>Attachment scale $\alpha = 0.836$	N/R	N/R	No	Yes
Gurganus (2002)[104] (Reference tool)	RADQ	N/R	N/R	N/R	No	Yes
Head (1997)[105] (Index tool)	Revised PBAR (A1)	N/R	N/R	N/R	No	Yes
Head (1997)[105] (Reference tool)	SSP (A22)	N/R	Dual coded<br>Agreement on four-way classification 85%, $\kappa = 0.77$	N/R	No	Yes
Madigan (2003)[25] (Index tool)	Family drawing clinical scheme	N/R	Dual coded<br>Agreement on presence/absence of signs 79–100%	N/R	Yes	Yes
Madigan (2003)[25] (Index tool)	Family Drawing Checklist	N/R	Dual coded<br>Agreement on presence/absence of signs 80–100%	N/R	Yes	Yes
Madigan (2003)[25] (Index tool)	Family Drawing Global Rating Scale (A17)	N/R	Dual coded<br>Coders' correlation on scale ratings = 0.54–0.85	N/R	Yes	Yes
Madigan (2003)[25] (Index tool)	Family drawing clinician's opinion (A3)	N/R	Dual coded<br>Agreement on three-way classification 84%, $\kappa = 0.64$	N/R	Yes	Yes

continued

© Queen's Printer and Controller of HMSO 2015. This work was produced by Wright *et al.* under the terms of a commissioning contract issued by the Secretary of State for Health. This issue may be freely reproduced for the purposes of private research and study and extracts (or indeed, the full report) may be included in professional journals provided that suitable acknowledgement is made and the reproduction is not associated with any form of advertising. Applications for commercial reproduction should be addressed to: NIHR Journals Library, National Institute for Health Research, Evaluation, Trials and Studies Coordinating Centre, Alpha House, University of Southampton Science Park, Southampton SO16 7NS, UK.

TABLE 6 Psychometric properties of the index and reference test(s) (*continued*)

Author and year (index or reference test) for included study	Instrument	Internal consistency (Cronbach's alpha)	Inter-rater reliability measures	Stability (test–retest: Pearson product moment correlation)	Evidence of content validity (yes/no)	Evidence of construct validity (yes/no)
Madigan *et al.* (2003)[25]	SSP (A3)	N/R	Dual coded	N/R	No	Yes
(Reference tool)			Agreement on three-way classification 80%			
Mangelsdorf *et al.* (1996)[106]	SSP (A11)	N/R	Dual coded: agreement on three-way classifications 90%	N/R	No	Yes
(Index tool)			Tri-coded (further subset): agreement on three-way classification 100%			
Mangelsdorf *et al.* (1996)[106]	AQS	N/R	Dual coded	N/R	No	Yes
(Reference tool)			Coders' reliability coefficient for full-term infants = 0.76 and for VLBW infants = 0.85			
Minnis *et al.* (2010)[107]	CMCAST (A10)	N/R	Dual coded	N/R	No	No
(Index tool)			Agreement on four-way classification 94%, $\kappa = 0.91$			
Minnis *et al.* (2010)[107]	MCAST (A10)	N/R	Dual coded	N/R	Yes	Yes
(Reference tool)			Agreement on four-way classification 96%, $\kappa = 0.93$			
Minnis *et al.* (2009);[108] McLaughlin *et al.* (2010)[109]	CAPA-RAD	N/R	Combined CAPA-RAD, WRO and RPQ diagnosis had 97% agreement with expert panel	N/R	Yes	Yes
(Index tool)						

Author and year (index or reference test) for included study	Instrument	Internal consistency (Cronbach's alpha)	Inter-rater reliability measures	Stability (test–retest: Pearson product moment correlation)	Evidence of content validity (yes/no)	Evidence of construct validity (yes/no)
Minnis *et al.* (2009);[108] McLaughlin *et al.* (2010)[109]<br><br>(Index tool)	WRO	$\alpha = 0.75$	Dual coded<br><br>Agreement on most questions (15/20) $\kappa > 0.61$<br><br>Agreement on five questions poor	N/R	Yes	Yes
Minnis *et al.* (2009);[108] McLaughlin *et al.* (2010)[109]<br><br>(Index tool)	RPQ	$\alpha = 0.92$	N/R	N/R	Yes	Yes
Minnis *et al.* (2009);[108] McLaughlin *et al.* (2010)[109]<br><br>(Reference tool)	MCAST	N/R	Dual coded<br><br>Agreement on four-way classification $\kappa = 0.93$<br><br>Agreement on two-way classification $\kappa = 1$	N/R	No	Yes
Ogilvie (2000)[110]<br><br>(Index tool)	BERS	N/R	N/R	N/R	Yes	Yes
Ogilvie (2000)[110]<br><br>(Index tool)	BERS + BAT	18/23 items assessed; $\alpha = 0.9166$	N/R	N/R	Yes	Yes
Ogilvie (2000)[110]<br><br>(Reference tool)	RADQ	Prior research has shown $\alpha > 0.80$	N/R	Prior research has shown test–retest correlations = 0.82 in the attachment disorder group and 0.85 in the non-attachment disorder group	No	Yes

continued

© Queen's Printer and Controller of HMSO 2015. This work was produced by Wright *et al.* under the terms of a commissioning contract issued by the Secretary of State for Health. This issue may be freely reproduced for the purposes of private research and study and extracts (or indeed, the full report) may be included in professional journals provided that suitable acknowledgement is made and the reproduction is not associated with any form of advertising. Applications for commercial reproduction should be addressed to: NIHR Journals Library, National Institute for Health Research, Evaluation, Trials and Studies Coordinating Centre, Alpha House, University of Southampton Science Park, Southampton SO16 7NS, UK.

TABLE 6 Psychometric properties of the index and reference test(s) (*continued*)

Author and year (index or reference test) for included study	Instrument	Internal consistency (Cronbach's alpha)	Inter-rater reliability measures	Stability (test–retest: Pearson product moment correlation)	Evidence of content validity (yes/no)	Evidence of construct validity (yes/no)
Oppenheim (1990);[111] Oppenheim (1997)[112]<br><br>(Index tool)	ADI	Emotional tone scale $\alpha = 0.89$<br><br>Emotional openness scale $\alpha = 0.96$<br><br>Resolution of distress score $\alpha = 0.57$	Dual coded<br><br>Coders' correlation on emotional openness scale = 0.88<br><br>Coders' correlation on emotional tone scale = 0.80<br><br>Coders' correlation on resolution of distress scale = 0.83	N/R	No	Yes
Oppenheim (1990);[111] Oppenheim (1997)[112]<br><br>(Reference tool)	AQS	N/R	N/R	Prior research has shown test–retest maternal AQS reliability coefficient = 0.88	No	Yes
Oppenheim (1990);[111] Oppenheim (1997)[112]<br><br>(Reference tool)	Bespoke separation–reunion observation	Two visits<br><br>Visit 1: exploration pre separation $\alpha = 0.88$; post separation $\alpha = 0.73$<br><br>Visit 2: exploration pre separation $\alpha = 0.93$; post separation $\alpha = 0.86$	Dual coded<br><br>Coders' reliability coefficient on exploration = 0.86, contact maintenance = 0.87, distal interaction = 0.69	Retest after 1 week<br><br>Pre-separation exploration correlation = 0.34 ($p = 0.043$)<br><br>Post-reunion exploration correlation = 0.41 ($p = 0.018$)<br><br>Post-reunion contact maintenance correlation = 0.66 ($p < 0.001$)	No	Yes

Author and year (index or reference test) for included study	Instrument	Internal consistency (Cronbach's alpha)	Inter-rater reliability measures	Stability (test–retest: Pearson product moment correlation)	Evidence of content validity (yes/no)	Evidence of construct validity (yes/no)
Posada (2006)[113]<br><br>(Index tool)	AQS	Smooth interactions with mother scale $\alpha = 0.90$<br><br>Proximity to mother scale $\alpha = 0.83$<br><br>Physical contact with mother scale $\alpha = 0.81$<br><br>Interactions with other adults scale $\alpha = 0.85$	Dual coded<br><br>Coders' correlation on score mean = 0.78	N/R	No	Yes
Posada (2006)[113]<br><br>(Reference tool)	SSP (A16)	N/R	Prior research has shown inter-rater agreement ranging from 75% to 92%	N/R	No	Yes
Roman (2010)[114]<br><br>(Index tool)	SSAP	Security indicators $\alpha = 0.853$<br><br>Insecurity indicators $\alpha = 0.835$<br><br>Avoidance indicators $\alpha = 0.822$<br><br>Disorganisation indicators $\alpha = 0.887$	Dual coded<br><br>Security indicators $\kappa = 0.899$<br><br>Insecurity indicators $\kappa = 0.848$<br><br>Avoidance indicators $\kappa = 0.879$<br><br>Disorganisation indicators $\kappa = 0.861$	N/R	No	Yes
Roman (2010)[114]<br><br>(Reference tool)	IMAS	$\alpha = 0.668$	N/R	N/R	No	Yes
Roman (2010)[114]<br><br>(Reference tool)	RPQ	Past-tense assessment $\alpha = 0.714$<br><br>Present assessment $\alpha = 0.747$	N/R	N/R	No	Yes

continued

© Queen's Printer and Controller of HMSO 2015. This work was produced by Wright *et al.* under the terms of a commissioning contract issued by the Secretary of State for Health. This issue may be freely reproduced for the purposes of private research and study and extracts (or indeed, the full report) may be included in professional journals provided that suitable acknowledgement is made and the reproduction is not associated with any form of advertising. Applications for commercial reproduction should be addressed to: NIHR Journals Library, National Institute for Health Research, Evaluation, Trials and Studies Coordinating Centre, Alpha House, University of Southampton Science Park, Southampton SO16 7NS, UK.

TABLE 6 Psychometric properties of the index and reference test(s) (*continued*)

Author and year (index or reference test) for included study	Instrument	Internal consistency (Cronbach's alpha)	Inter-rater reliability measures	Stability (test–retest: Pearson product moment correlation)	Evidence of content validity (yes/no)	Evidence of construct validity (yes/no)
Shmueli-Goetz *et al.* (2008)[52]<br><br>(Index tool)	CAI (A20)	State of mind scales $\alpha = 0.87$<br><br>Avoidance scales $\alpha = 0.84$<br><br>Active conflict scales $\alpha = 0.43$	Three sets of coders<br><br>(κ range for two-, three- and four-way classifications)<br><br>Group 1: tri-coded; median correlation = 0.88, κ range 0.80–0.86<br><br>Group 2: dual coded; median correlation = 0.87, κ range 0.67–0.81<br><br>Group 3: unclear number of coders; median correlation = 0.81, κ range 0.78–0.87	Retest 3 months apart: median correlation of scales = 0.69<br><br>Retest 1 year apart: median correlation of scales = 0.54	No	Yes
Shmueli-Goetz *et al.* (2008)[52]<br><br>(Reference tool)	SAT (A2)	N/R	N/R	N/R	No	Yes
Silver (2005)[115]<br><br>(Index tool)	Family Drawing Checklist	N/R	Dual coded<br><br>Agreement on presence/absence of signs 86–100%<br><br>κ range 0.72–1.00	N/R	No	Yes
Silver (2005)[115]<br><br>(Index tool)	Family Drawing Global Rating Scale	N/R	Dual coded<br><br>Coders' correlation on scale ratings = 0.87–0.96	N/R	No	Yes
Silver (2005)[115]<br><br>(Index tool)	Family drawing investigator's opinion (A4)	N/R	N/R	N/R	No	Yes

Author and year (index or reference test) for included study	Instrument	Internal consistency (Cronbach's alpha)	Inter-rater reliability measures	Stability (test–retest: Pearson product moment correlation)	Evidence of content validity (yes/no)	Evidence of construct validity (yes/no)
Silver (2005)[115]<br>(Index tool)	Family drawing clinician's opinion (A4)	N/R	N/R	N/R	No	Yes
Silver (2005)[115]<br>(Index tool)	Modified relatedness scales (A29)	N/R	N/R	N/R	No	Yes
Silver (2005)[115]<br>(Reference tool)	SSP (A4)	N/R	Dual coded<br>Agreement on four-way classification 77%	N/R	No	Yes
	SSP (A23)	N/R	Dual coded<br>Agreement on five-way classification 62%	N/R	No	Yes
Sirl (1999)[116]<br>(Index tool)	Modified ASCT	Four attachment-themed stories $\alpha = 0.56$<br>Four alternatively themed stories $\alpha = 0.39$<br>Eight stories combined $\alpha = 0.66$	Unclear number of coders<br>Agreement on coding 67%<br>Coders' correlation $\alpha = 0.75$ (range −0.24 to 1.00)	N/R	Yes	Yes
Sirl (1999)[116]<br>(Reference tool)	SSP with separation–reunion procedure (A26)	N/R	Dual coded<br>Agreement on four-way classification 84%, $\kappa = 0.58$ ($p < 0.001$)	N/R	No	Yes
Smeekens *et al.* (2009)[117]<br>(Index tool)	Shortened SSP (A4)	N/R	Dual coded<br>Agreement on four-way classification 95%	N/R	No	Yes
Smeekens *et al.* (2009)[117]<br>(Reference tool)	AQS	N/R	Dual coded<br>Reliability > 0.75	N/R	No	Yes

continued

© Queen's Printer and Controller of HMSO 2015. This work was produced by Wright *et al.* under the terms of a commissioning contract issued by the Secretary of State for Health. This issue may be freely reproduced for the purposes of private research and study and extracts (or indeed, the full report) may be included in professional journals provided that suitable acknowledgement is made and the reproduction is not associated with any form of advertising. Applications for commercial reproduction should be addressed to: NIHR Journals Library, National Institute for Health Research, Evaluation, Trials and Studies Coordinating Centre, Alpha House, University of Southampton Science Park, Southampton SO16 7NS, UK.

TABLE 6 Psychometric properties of the index and reference test(s) (*continued*)

Author and year (index or reference test) for included study	Instrument	Internal consistency (Cronbach's alpha)	Inter-rater reliability measures	Stability (test–retest: Pearson product moment correlation)	Evidence of content validity (yes/no)	Evidence of construct validity (yes/no)
Solomon (1995)[24]<br>(Index tool)	Adapted separation–reunion story completion task (A30)	N/R	Tri-coded<br>Agreement on four-way classification 71%, $\kappa = 0.62$ ($p < 0.001$)	N/R	No	Yes
Solomon (1995)[24]<br>(Reference tool)	Separation–reunion episode (A15)	N/R	Dual coded<br>Agreement on four-way classification 71%, $\kappa = 0.62$ ($p < 0.001$)	N/R	No	Yes
Spieker and Crittenden (2010)[118]<br>(Index tool)	Modified SSP – MacArthur classification system (A27)	N/R	Tri-coded<br>Agreement on four-way classification 77%, $\kappa = 0.50$ ($p < 0.001$)	N/R	No	Yes
Spieker and Crittenden (2010)[118]<br>(Index tool)	Modified SSP-PAA (A19)	N/R	Dual coded<br>Agreement on six-way classification 59%, $\kappa = 0.45$ ($p < 0.001$)	N/R	No	Yes
Spieker and Crittenden (2010)[118]<br>(Reference tool)	SSP (A4)	N/R	Dual coded<br>Agreement on four-way classification 82%, $\kappa = 0.70$	N/R	No	Yes
Tarabulsy and Moran (1997)[119]<br>(Index tool)	AQS	N/R	N/R	N/R	No	Yes
Tarabulsy and Moran (1997)[119]<br>(Reference tool)	SSP (A8)	N/R	Dual coded<br>Agreement on three-way classification 91%	N/R	No	Yes

Author and year (index or reference test) for included study	Instrument	Internal consistency (Cronbach's alpha)	Inter-rater reliability measures	Stability (test–retest: Pearson product moment correlation)	Evidence of content validity (yes/no)	Evidence of construct validity (yes/no)
van Dam and Van IJzendoorn (1988)[120]<br><br>(Index tool)	Adapted AQS	N/R	N/R	Retest approximately 10 days apart<br><br>Security scores = 0.75<br><br>Dependency scores = 0.86<br><br>Sociability scores = 0.78<br><br>Desirability scores = 0.82	No	Yes
van Dam and Van IJzendoorn (1988)[120]<br><br>(Reference tool)	SSP (A14)	N/R	Dual coded<br><br>Agreement on three-way classification 100%<br><br>Agreement on eight-way classification 93%<br><br>Reliability for interactive scales = 0.78	N/R	No	Yes
Vaughn and Waters (1990)[121]<br><br>(Index tool)	SSP (A3)	N/R	Dual coded<br><br>Agreement on three-way classification 86%	N/R	No	Yes

continued

© Queen's Printer and Controller of HMSO 2015. This work was produced by Wright *et al.* under the terms of a commissioning contract issued by the Secretary of State for Health. This issue may be freely reproduced for the purposes of private research and study and extracts (or indeed, the full report) may be included in professional journals provided that suitable acknowledgement is made and the reproduction is not associated with any form of advertising. Applications for commercial reproduction should be addressed to: NIHR Journals Library, National Institute for Health Research, Evaluation, Trials and Studies Coordinating Centre, Alpha House, University of Southampton Science Park, Southampton SO16 7NS, UK.

TABLE 6 Psychometric properties of the index and reference test(s) (*continued*)

Author and year (index or reference test) for included study	Instrument	Internal consistency (Cronbach's alpha)	Inter-rater reliability measures	Stability (test–retest: Pearson product moment correlation)	Evidence of content validity (yes/no)	Evidence of construct validity (yes/no)
Vaughn and Waters (1990)[121]<br><br>(Reference tool)	AQS	N/R	Dual coded<br><br>Coders' correlation on security score = 0.58<br><br>Coders' correlation on dependency score = 0.72<br><br>Coders' correlation on sociability score = 0.53<br><br>All $p < 0.05$	N/R	No	Yes
Ziegenhein and Jacobsen (1999)[122]<br><br>(Index tool)	Parent–child separation story (A9)	N/R	Dual coded<br><br>Agreement on four-way classification $\kappa = 0.68$	N/R	No	Yes
Ziegenhein and Jacobsen (1999)[122]<br><br>(Reference tool)	SSP (A9)	N/R	Dual coded<br><br>Agreement on four-way classification $\kappa = 0.87$ at 12 months and 0.91 at 18 months	N/R	No	No
Ziegenhein and Jacobsen (1999)[122]<br><br>(Reference tool)	Separation–reunion episode (A9)	N/R	Dual coded<br><br>Agreement on four-way classification $\kappa = 0.80$	N/R	No	Yes

ASCT, Attachment Story Completion Task; CAP, California Attachment Procedure; CAPA-RAD, Child and Adolescent Psychiatric Assessment – reactive attachment disorder; N/R, not reported; PBAR, Permitting Blocking Access Inventory; RR, role reversal; SSP-CM, Strange Situation Procedure-Cassidy–Marvin; VLBW, very low birthweight.

Test–retest data were the least reported information in the included studies, with only five studies reporting these data for either the index or reference test.[47,52,110,111,120]

Inter-rater reliability data were the most frequently reported validity data. A total of 26 studies reported these data for the index test[24,25,47,52,93,95–108,111,113–118,121,122] and 23 studies for the reference test.[24,25,47,93,96,98–100,102,105–108,111,113,115–122] Of these 26, 24 had good inter-rater reliability as defined by a level of 0.7 or above.[25,47,52,93,95–103,106–108,111,113–117,121,122]

Cronbach's alphas were reported in 12 studies for the index tests (12 studies $\alpha > 0.7$)[47,52,95–97,104,108,110,111,113,114,116] and in four studies for the reference tests (four studies $\alpha > 0.7$).[97,110,110,114]

Sensitivity and specificity analysis

Nine studies were found that compared an attachment assessment procedure with a reference standard.[25,93–95,102,108,115,118,122] Only three of these reported data that enabled calculations of concurrent validity sensitivity and specificity scores: two for attachment patterns[93,94] (*Table 7*) and one for attachment disorders[95] (*Table 8*).

The characteristics of these studies were summarised in *Table 1*. The majority validated an attachment assessment procedure under evaluation against the SSP. Most studies (six) were conducted in the USA[25,93,94,102,115,118] and the remainder (three) were conducted in European countries (Germany,[122] Romania[95] and Scotland[108]). The size of the samples ranged from 33[122] to just below 300.[118] There was no significant discrepancy in the proportions of boys and girls in any of the studies. Two studies did not report sufficient data to calculate 2 × 2 tables.[108,122]

TABLE 7 Performance of attachment procedure measures: concurrent validity as compared with reference standard

Study	Sensitivity (95% CI)	Specificity (95% CI)	ROC (95% CI)	DOR (95% CI)	PPV (95% CI)	NPV (95% CI)
Clarke-Stewart *et al.* (2001)[93] CAP vs. SSP (secure attachment)	0.90 (0.76 to 0.97)	0.30 (0.11 to 0.54)	0.60 (0.48 to 0.71)	3.86 (1.00 to 14.80)	0.72 (0.57 to 0.83)	0.60 (0.26 to 0.87)
Finkel *et al.* (1998)[94] LTS vs. SSP (secure attachment)	0.83 (0.61 to 0.95)	0.67 (0.3 to 0.93)	0.73 (0.55 to 0.90)	9.5 (1.64 to 55)	0.86 (0.65 to 0.97)	0.6 (0.26 to 0.88)

CI, confidence interval; DOR, diagnostic odds ratio; NPV, negative predictive value; PPV, positive predictive value; ROC, receiver operating characteristic.

TABLE 8 Performance of instruments assessing attachment disorders: concurrent validity as compared with reference standard

Study	Sensitivity (95% CI)	Specificity (95% CI)	ROC (95% CI)	DOR (95% CI)	PPV (95% CI)	NPV (95% CI)
Gleason *et al.* (2011)[95] DAI vs. PAPA (DAD)	0.81 (0.54 to 0.96)	0.86 (0.78 to 0.92)	0.83 (0.73 to 0.94)	27.85 (7.04 to 110.27)	0.48 (0.28 to 0.68)	0.96 (0.90 to 0.99)
Gleason *et al.* (2011)[95] DAI vs. PAPA RAD (inhibited attachment disorder)	0.80 (0.28 to 0.99)	0.99 (0.95 to 1.00)	0.86 (0.69 to 1.00)	456.00 (23.97 to 8675.15)	0.80 (0.28 to 0.99)	0.99 (0.95 to 1.00)

CI, confidence interval; DOR, diagnostic odds ratio; NPV, negative predictive value; PPV, positive predictive value; ROC, receiver operating characteristic.

© Queen's Printer and Controller of HMSO 2015. This work was produced by Wright *et al.* under the terms of a commissioning contract issued by the Secretary of State for Health. This issue may be freely reproduced for the purposes of private research and study and extracts (or indeed, the full report) may be included in professional journals provided that suitable acknowledgement is made and the reproduction is not associated with any form of advertising. Applications for commercial reproduction should be addressed to: NIHR Journals Library, National Institute for Health Research, Evaluation, Trials and Studies Coordinating Centre, Alpha House, University of Southampton Science Park, Southampton SO16 7NS, UK.

Most studies attempting to compare two attachment instruments did not include raw data. Two studies reported data that allowed concurrent validity calculation of sensitivity and specificity of a new procedure for assessing infant–mother attachment pattern.[93,94] Both studies were conducted in the USA and the samples sizes were small (60 toddlers[93] and 16 twin pairs[94] respectively).

Sensitivity and specificity of attachment pattern measures

Clarke-Stewart and colleagues[93] developed the CAP to assess attachment patterns. The SSP was administered when the children were approximately 17 months of age, always before the CAP. The CAP was administered when the children were approximately 18 months old. When compared with the SSP in detecting secure attachment, the sensitivity of CAP was 0.90 [95% confidence interval (CI) 0.76 to 0.97] but the specificity was very low at 0.30 (95% CI 0.11 to 0.54). The performance characteristics of CAP are summarised in *Table 7*. Disorganised attachment was not compared. The CAP, although good at identifying true positives, also has a very high false-positive rate, and so would not be useful as a screening instrument.

Finkel and colleagues[94] validated a measure of attachment for twins. Sixteen twin pairs from the LTS participated in an attachment assessment procedure (the LTS procedure), and 1 month later in the SSP (nine pairs at age 19 months and seven pairs at age 25 months). The sensitivity of the LTS procedure to detect secure attachment was 0.82 (95% CI 0.61 to 0.95) and specificity was 0.66 (95% CI 0.29 to 0.92), which represents a high false-positive rate. *Table 7* summarises the performance characteristics of the LTS procedure compared with the SSP as the reference standard.

Sensitivity and specificity of attachment disorder diagnostic tools

One study[95] examined the validity of an instrument for detecting the two types of RAD against the RDC. The authors assessed the criterion validity of the two types of RAD, comparing adult-reported signs of RAD using the DAI with the diagnosis as determined by a diagnostic interview (PAPA). The DAI is a semistructured, examiner-based interview of a caregiver who reports on signs of RAD in very young children. The PAPA is a comprehensive, parent-reported psychiatric diagnosis interview for preschool children. PAPA was administered when children were 54 months of age and the DAI was administered at 22, 30, 42 and 54 months. The validities of the DAI for indiscriminately social/disinhibited reactive disorder and emotionally withdrawn/inhibited reactive disorder respectively at 54 months, compared with PAPA, are summarised in *Table 8* and show good sensitivity and specificity.

Interestingly, we found one study that met our criteria that concurrently compared disorganised attachment pattern with attachment disorder. Gleason and colleagues[95] carried out the SSP in 135 Romanian children at 42 months and the DAI at the same time. Although these are not purporting to measure the same thing, we carried out some illustrative correlational statistics which confirmed that whether disorganised attachment pattern classification or secure attachment classification pattern is used to screen for concurrent attachment disorder, neither are helpful. Neither had a positive predictive value of more than 30%. Using disorganised attachment there were large numbers of children with attachment disorders who had organised patterns of attachment (19 out of 22; very low sensitivity of 0.14). Using insecure *or* disorganised attachment together, there were large numbers of children *not* securely attached who did not have attachment disorders (62 out of 82; very poor specificity of 0.39). We did not calculate the statistics for this because attachment patterns and attachment disorders are not attempting to measure the same construct. This is therefore for illustrative purposes only.

Discussion and summary of findings

Our review found a very large number of instruments used in an attempt to classify attachment patterns. Many of these are unvalidated in that they make no comparison with other attachment instruments or the reference standard (the SSP). Furthermore, numerous groups have redesigned aspects of the Ainsworth SSP, added categories to the classification system (or changed the boundaries of named categories) or changed the names of subclassifications. This makes the literature very confusing for all but the most avid attachment researcher. Researchers' views of the classification systems will be shaped by which papers they happen to be reading or taught in training. For example, across the 16 studies identified as using the standard Ainsworth SSP, 12 different variations on the classification system were used.[25,93,94,98,102,103,105,106,113,115,116,118–122]

Beyond the SSP, clinicians and researchers use a variety of techniques to assess children at different ages for attachment patterns. These include questionnaires, interviews, observation, stories and drawings. Many of these are not validated.

The only piece of research that concurrently compared the RAD diagnosis with attachment pattern assessment using the reference standard (that elucidated secure attachment patterns and disorganised attachment patterns) showed little relationship between attachment patterns and attachment disorders.[95] This suggests that using attachment pattern assessments in screening for attachment disorders is not helpful. It also appears to confirm the view of some authors that the constructs of attachment patterns and attachment disorders are not closely related.

This element of the review demonstrated that against a backdrop of many interesting hypotheses and theories, there is a need for further, high-quality scientific research that validates available instruments for use, compares them across time and child development and leads to expert consensus in how they are used to identify children at risk. The narrative literature on attachment and development rightly focuses attention on a child's behaviour in the context of his or her main caregivers and early-life relationships. More research is needed to allow scientific agreement to develop around key mechanisms to measure maladaptive attachment patterns and attachment disorders.

This initial supplementary review sought to identify a range of different screening and assessment tools used to measure attachment patterns or provide diagnostic criteria to identify an attachment disorder. For the purpose of our supplementary review of outcomes at 10 years or more (supplementary review 2, see *Chapter 5*), we used this review (supplementary review 1) to gather reliable and valid baseline measures of attachment.

For attachment patterns, as discussed in *Chapter 2* (see *Figure 1*), we have used the SSP[3] with a disorganised category (e.g. the addition of Main and Solomon's category D coding[6]). Additional attachment pattern assessment tools would be needed to meet the criteria described below.

- studies identifying attachment patterns
- identifying studies which had tested the development of screening or assessment tools against the SSP, or a psychometrically sound reference test that has been concurrently validated against the SSP with good sensitivity and specificity
- AND demonstrated good validity and reliability by reporting satisfactory Cronbach's alpha (> 0.7); a good significant correlational relationship between the test instrument and the reference test; assessment of the content validity; and illustrating good test–retest reliability.

The only two studies that we found that concurrently validated a second instrument against the SSP had good sensitivity but poor specificity.[93,94] Several studies reported good Cronbach's alpha scores with some attempt to measure construct validity but did not conduct concurrent validity against the SSP.[47,52,95–97,104,108,110,111,113,114,116] It was not possible to assert that any instrument had good enough validity and reliability to be used alongside our reference standard, the SSP.

© Queen's Printer and Controller of HMSO 2015. This work was produced by Wright *et al.* under the terms of a commissioning contract issued by the Secretary of State for Health. This issue may be freely reproduced for the purposes of private research and study and extracts (or indeed, the full report) may be included in professional journals provided that suitable acknowledgement is made and the reproduction is not associated with any form of advertising. Applications for commercial reproduction should be addressed to: NIHR Journals Library, National Institute for Health Research, Evaluation, Trials and Studies Coordinating Centre, Alpha House, University of Southampton Science Park, Southampton SO16 7NS, UK.

For attachment disorder, the RDC (DSM-IV[56] and ICD-10[55]) would be used (as discussed in *Chapter 2*; see *Figure 1*). Additional assessment tools would need to meet the criteria described below identifying attachment disorders:

- studies measuring an attachment disorder in comparison to a clinical population of RAD/DAD that had been identified initially using some standardised diagnostic criteria (e.g. DSM-IV/ICD-10, DC: 0–3)
- or where instruments were compared with a validated diagnostic criterion measurement/interview and demonstrated good validity and reliability using the criteria specified above.

In addition to the RDC, we found that the DAI had good concurrent validity against RDC for attachment disorder. Therefore, the DAI was accepted as a validated instrument that was used in the supplementary review 2 screening process for 10-year outcomes.

The new diagnostic systems (DSM-V[58] and ICD-11[88]) need to become established and to be used in research, in order to move us from theoretical constructs and newly evolved diagnostic systems to a better understanding of the relationship between various measures of attachment problems and their meaning in terms of short-, mid- and long-term outcomes.

In order for the literature to be more helpful to clinicians and future researchers, there need to be high-quality methodological studies in this field. In particular, there needs to be clarity to the classification system and nomenclature, and the assessment procedures.

Chapter 5 Supplementary systematic review 2: studies of severe attachment problems with a follow-up of 10 years or longer

Introduction

The research objective of our second supplementary review was to examine the 10-year outcomes of developmental, psychological and behavioural disorders among children with severe attachment problems and to collect prevalence information from these studies. It was not within the scope of this funded review to conduct a separate systematic review of prevalence data.

Although the main review sought to assess the clinical effectiveness and cost-effectiveness of parental instruments for severe attachment problems, we outlined two supplementary reviews (see *Chapter 4* and the present chapter) to help us interpret this work. This chapter seeks to provide robust data for health economic analysis by exploring outcomes at 10 years or more in infants or children with severe attachment problems at baseline. When we scoped the work for our main review, we found that outcomes were measured but were largely short term. These longer-term data supplement this. The steering group spent considerable time discussing this and also believed that they would provide some additional prevalence and outcome estimations to be used in the health economics analysis. In normal populations, approximately 35% of infants show some form of insecure attachment pattern.[87] The organised insecure patterns of attachment are therefore unlikely to be helpful as indicators of pathology, but rather, may be considered as risk factors for associated difficulties in the child's functioning.[39] Although many people with psychopathology may have had earlier attachment problems, most infants with insecure attachment patterns do not go on to develop psychopathology.[87] By contrast, the disorganised attachment pattern, unlike the organised insecure classifications, has been associated with behaviour problems in childhood.[23] Our review has defined 'severe attachment problems' as either a diagnosed attachment disorder or a disorganised attachment pattern.

Longitudinal studies have suggested that disorganised attachment is linked to hostility and hyperactivity, aggression and oppositional defiant disorder in children,[37] and to dissociative symptoms in 17- and 19-year-olds.[27] Furthermore, attachment disorders, as distinct from attachment patterns, are known to have increased comorbidity with conduct disorders, developmental delay, attention deficit hyperactivity disorder and post-traumatic stress disorder.[38]

Methods

We initially screened titles and abstracts using two reviewers, independent of each other. Any disagreements were resolved by discussion or arbitration, and a third party when required. Where both reviewers agreed, a full copy of the paper was obtained and assessed in more detail for potential inclusion in the review. In instances where a foreign language paper was identified, the paper was translated and then screening was performed.

© Queen's Printer and Controller of HMSO 2015. This work was produced by Wright *et al.* under the terms of a commissioning contract issued by the Secretary of State for Health. This issue may be freely reproduced for the purposes of private research and study and extracts (or indeed, the full report) may be included in professional journals provided that suitable acknowledgement is made and the reproduction is not associated with any form of advertising. Applications for commercial reproduction should be addressed to: NIHR Journals Library, National Institute for Health Research, Evaluation, Trials and Studies Coordinating Centre, Alpha House, University of Southampton Science Park, Southampton SO16 7NS, UK.

Inclusion criteria

The PICOS criteria were as follows:

- *Population and exposure* Studies that measured attachment disorder or disorganised attachment pattern in children under the age of 13 years at inception. If infants were too young to measure attachment at inception, studies were included if attachment was measured at, or before, the age of 12 months.
- *Instruments* Studies were only included where attachment problems had been measured using the diagnostic criteria for attachment disorder or disorganised attachment patterns using the SSP[8] with the disorganised pattern described by Main and Solomon.[6] If any measures had been validated against the reference standard in supplementary review 1, studies including those measures would have been included here, but none was found.
- *Comparator* Those without a disorganised attachment pattern or attachment disorder, at baseline or earliest time point, served as a comparator against those with a severe attachment problem.
- *Outcomes* Studies had to contain *any* of the following:
  - Data on the prevalence of severe attachment problems (defined as disorganised attachment pattern[6] or the diagnosis of attachment disorder).[55,56]
  - Epidemiological data including outcome data. The long-term outcomes searched for were stability of severe attachment problems, measured by an adolescent or adult measure of attachment; rates of subsequent mental ill health; psychosocial development; educational attainment; entry into care; or involvement in the criminal justice system. Only validated outcome measures or objectively measurable full-population outcomes, such as school grades or criminal convictions, were examined in each of these domains.
- *Study design* Studies were prospective longitudinal cohorts with a follow-up period of 10 years or more.

There are many studies on short-term outcomes of severe attachment problems which have been the subject of systematic reviews[23] and selective reviews by other authors.[147] Our initial scoping demonstrated a vast literature, and as the main focus of this review was on parental interventions, the steering group decided after lengthy discussion to limit this supplementary review to long-term sequelae (10 years or more) of severe attachment problems to explore any important long-term outcomes that might provide useful information for health economics analysis.

Data extraction

Data were extracted by two independent reviewers, who met to discuss any discrepancies in order to reach an agreement. Studies with multiple papers were examined and extracted separately. The following items were extracted for each paper: study characteristics, population details, prevalence and incidence of RAD and attachment patterns, stability of attachment and the specified long-term outcomes of children with severe attachment problems. The data extracted on participant characteristics and prevalence of attachment classifications relate to the sample that was followed up longitudinally, rather than the full, original sample. Where data were not reported in full, calculations were made from the reported data included. If this was not possible, the paper was excluded.

Quality assessment

For each study reporting the prevalence of severe attachment problems and/or the long-term outcomes for this population, we applied a bespoke 13-item quality assessment tool (*Table 9*). The tool was developed using the CRD's suggested quality criteria for assessment and recommendations from a systematic review of tools to assess the quality of cohort studies,[148] and by reference to previously administered cohort quality scales, such as the Newcastle–Ottawa Quality Assessment Scale for cohort studies.[149] We specified an attrition rate of $\leq 20\%$ as adequate for long-term cohort studies to receive a rating of 'low bias', based on previous quality assessment guidelines for cohort studies.[150]

The questions were developed to cover the five key domains for tools assessing study quality based on Sanderson and colleagues'[148] systematic review, with studies being allocated high, low or unclear bias. *Table 9* summarises these quality assessment criteria; further details of scoring can be found in *Appendix 3*.

Data synthesis

Given wide variability in outcomes, it was not appropriate to use meta-analytic procedures for this phase of the review. Instead, a narrative overview of the studies is given.

TABLE 9 Criteria and domains examined by the bespoke quality assessment tool

Criteria question	Domain
Q1: Was the cohort drawn from the same community/source?	Method for selecting study participants
Q2: Are the groups assembled/recruited at the same age (i.e. the measurement period)?	
Q3: Ascertainment of exposure – was the same measurement of attachment patterns/disorders used across the sample?	Methods for measuring exposure and outcome variables
Q4: Were the coders of the exposure blind to risk factors/predictive variables related to the exposure status?	
Q5: Is there demonstration that outcome(s) of interest were not present at the start of the study?	
Q6: Is there a description of attachment classification across the entire sample at baseline?	
Q7: Were subsequent measures rated by blind coders who were not aware of the exposed/unexposed status?	
Q8: Were there any significant differences at baseline (i.e. demographic variables) between those lost at follow-up?	Design-specific sources of bias
Q9: If significant differences at baseline are found, was any analysis performed to compensate?	
Q10: Adequacy of follow-up: were the dropout rates/attrition adequately reported?	
Q11: Were dropout rates and reasons for dropout similar across the exposed/unexposed?	
Q12: Did the study declare conflicts of interest or identification of funding resources?	Conflicts of interest
Q13: Any other bias?	Any other bias

© Queen's Printer and Controller of HMSO 2015. This work was produced by Wright *et al.* under the terms of a commissioning contract issued by the Secretary of State for Health. This issue may be freely reproduced for the purposes of private research and study and extracts (or indeed, the full report) may be included in professional journals provided that suitable acknowledgement is made and the reproduction is not associated with any form of advertising. Applications for commercial reproduction should be addressed to: NIHR Journals Library, National Institute for Health Research, Evaluation, Trials and Studies Coordinating Centre, Alpha House, University of Southampton Science Park, Southampton SO16 7NS, UK.

Results

Figure 4 summarises the selection process for supplementary review 2 examining outcomes at 10 years or more. Although a large number of studies met first-sift criteria ($n = 222$), only a small number met final inclusion criteria ($n = 8$[45,151–157]).

Study and sample characteristics and prevalence data

Table 10 shows the participant and study characteristics of included studies.

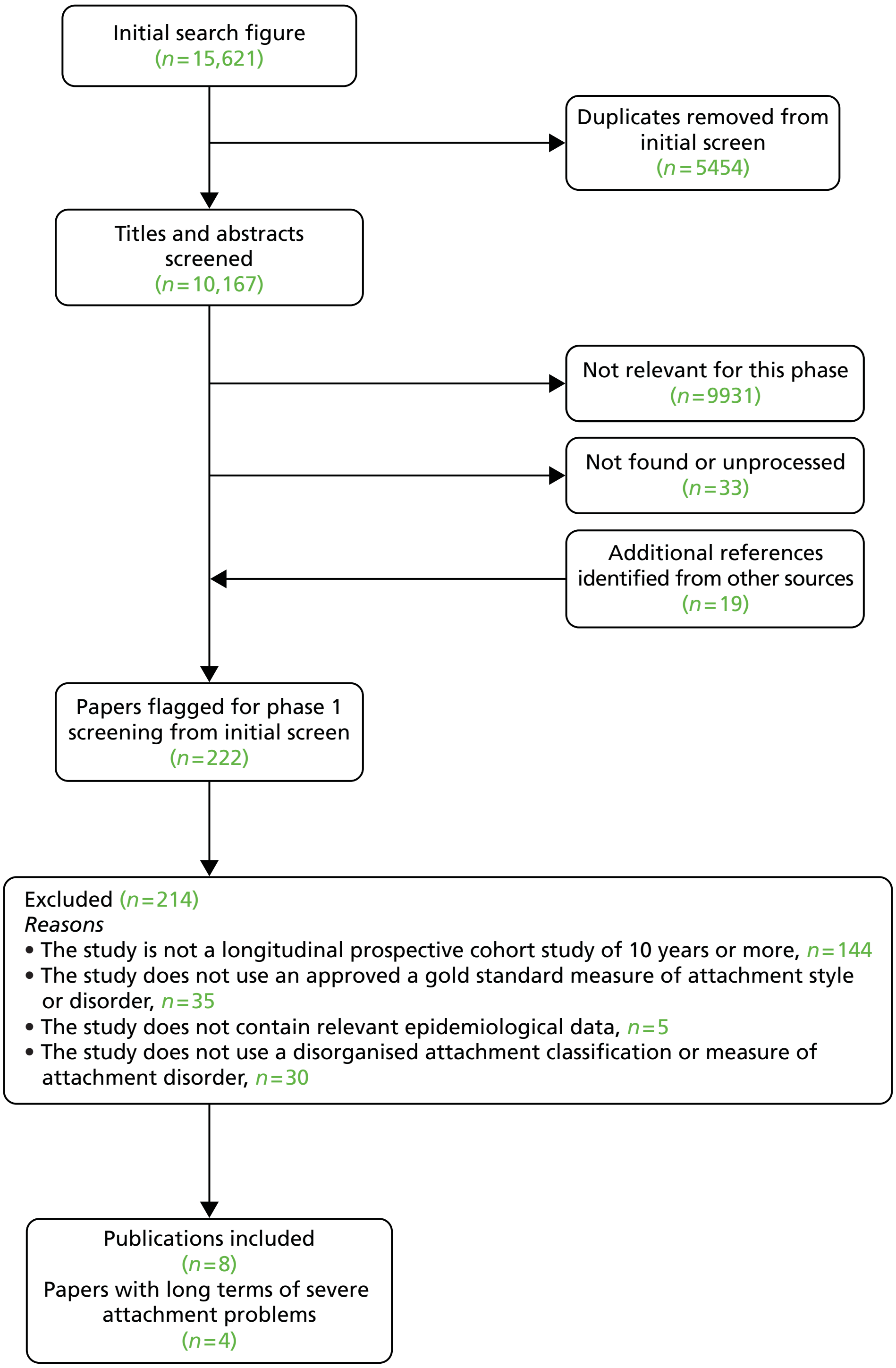

FIGURE 4 Preferred Reporting Items for Systematic Reviews and Meta-Analyses flow chart of papers included in supplementary review 2.

TABLE 10 Study, sample and prevalence characteristics of included studies

Author, year and country of publication	Study characteristics	Sample characteristics of the child at recruitment	Sample characteristics of the caregiver	Age of child at attachment measure	Attachment measure at baseline or earliest time point
Aikins *et al.* (2009)[151]<br><br>USA	At enrolment $n = 94$<br><br>At end of study $n = 47$<br><br>50% followed up<br><br>15-year follow-up	Age 12 months<br><br>Mean age not reported (SD not reported), range not reported<br><br>Sample defined as low risk<br><br>No further specification	Parents' age not reported<br><br>90% European American<br><br>100% middle class	12 months	SSP [Ainsworth (1978);[8] Main and Solomon (1990)[6]]<br><br>11% disorganised, 62% secure, 17% avoidant, 6% ambivalent
Ammaniti *et al.* (2005)[154]<br><br>Country not reported	At enrolment $n = 35$<br><br>At end of study $n = 21$<br><br>60% followed up<br><br>10- to 11-year follow-up	Mean age 12 months (SD not reported), range not reported	Mothers' mean age 29 years (SD not reported), range not reported<br><br>Ethnicity not reported<br><br>100% middle class	12–15 months	SSP [Main and Solomon (1990)[6]]<br><br>20% disorganised, 80% organised<br><br>SSP [Ainsworth (1978)[8]]<br><br>43% secure, 54% avoidant, 3% resistant
Carlson (1998)[45]<br><br>USA	Minnesota Longitudinal Study of Risk and Adaptation[158]<br><br>At enrolment $n = 267$<br><br>At end of study $n = 122$<br><br>46% followed up<br><br>19-year follow-up	Prenatal<br><br>High risk of poor developmental outcomes due to parents living in poverty	Mothers' mean age 20.66 years (SD 3.57 years), range 14–34 years<br><br>67% Caucasian<br><br>100% below poverty line	12 and 18 months<br><br>Some tapes were missing so attachment pattern was determined from data available	SSP [Main and Solomon (1990)[6]]<br><br>35% disorganised, 65% organised<br><br>SSP [Ainsworth (1978)[8]]<br><br>45% secure, 30% avoidant, 25% resistant

continued

© Queen's Printer and Controller of HMSO 2015. This work was produced by Wright *et al.* under the terms of a commissioning contract issued by the Secretary of State for Health. This issue may be freely reproduced for the purposes of private research and study and extracts (or indeed, the full report) may be included in professional journals provided that suitable acknowledgement is made and the reproduction is not associated with any form of advertising. Applications for commercial reproduction should be addressed to: NIHR Journals Library, National Institute for Health Research, Evaluation, Trials and Studies Coordinating Centre, Alpha House, University of Southampton Science Park, Southampton SO16 7NS, UK.

TABLE 10 Study, sample and prevalence characteristics of included studies (*continued*)

Author, year and country of publication	Study characteristics	Sample characteristics of the child at recruitment	Sample characteristics of the caregiver	Age of child at attachment measure	Attachment measure at baseline or earliest time point
Carlson *et al.* (2009)[153]<br><br>USA	Minnesota Longitudinal Study of Risk and Adaptation[158]<br><br>At enrolment $n = 267$<br><br>At end of study $n = 162$<br><br>61% followed up<br><br>28-year follow-up	Prenatal<br><br>High risk of poor developmental outcomes due to parents living in poverty	Mothers' mean age 20.66 years (SD 3.57 years), range 14–34 years<br><br>67% Caucasian<br><br>100% below poverty line	12 and 18 months	SSP<br><br>Prevalence not reported
Dan *et al.* (2011)[155]<br><br>Israel	Haifa Study of Early Child Care<br><br>At enrolment $n = 758$<br><br>At end of study $n = 136$<br><br>18% followed up<br><br>11-year follow-up	Mean gestational age 39.9 weeks (SD 1.22 weeks), range not reported	Mothers' mean age 29.2 years (SD 4.78 years) range not reported<br><br>100% Jewish<br><br>SES reflects whole population of Greater Haifa	12 months	SSP [Ainsworth (1978);[8] Main and Solomon (1990)[6]]<br><br>3% disorganised, 65% secure, 12% avoidant, 20% resistant
Jaffari-Bimmel *et al.* (2006)[156]<br><br>the Netherlands	At enrolment $n = 160$<br><br>At end of study $n = 143$<br><br>89% followed up<br><br>14-year follow-up	Mean age at adoption 10.76 weeks (SD 5.53 weeks) range not reported<br><br>Internationally adopted children, adopted before age 6 months<br><br>53.7% adopted from Sri Lanka	At birth, adoptive fathers' mean age was 34.62 years (SD 3.48 years) range not reported<br><br>Adoptive mothers' mean age was 32.52 years (SD 3.35 years) range not reported<br><br>Families 100% Caucasian<br><br>Predominantly middle or upper class	12 months	SSP [Main and Solomon (1990)[6]]<br><br>16% disorganised, 84% organised<br><br>SSP [Ainsworth (1978)[8]]<br><br>76% secure, 22% avoidant, 3% resistant

Author, year and country of publication	Study characteristics	Sample characteristics of the child at recruitment	Sample characteristics of the caregiver	Age of child at attachment measure	Attachment measure at baseline or earliest time point
Steele *et al.* (2002)[157]<br><br>UK	London Parent–Child Project<br><br>At enrolment $n = 100$<br><br>At end of study $n = 51$<br><br>51% followed up<br><br>11-year follow-up	Prenatal<br><br>Sample defined as low risk<br><br>No further specification	Mothers' median age 31 years (range 22–42 years)<br><br>100% white<br><br>Predominantly middle class	12 and 18 months (with mother and father respectively)	SSP [Ainsworth (1978);[8] Main and Solomon (1990)[6]]<br><br>8% and 2% disorganised (with mother and father respectively)<br><br>55% and 66% secure (with mother and father respectively)<br><br>29% and 32% avoidant (with mother and father respectively)<br><br>8% and 0% ambivalent (with mother and father respectively)
Weinfield *et al.* (2004)[152]<br><br>USA	Minnesota Longitudinal Study of Risk and Adaptation[158]<br><br>At enrolment $n = 267$<br><br>At end of study $n = 125$<br><br>47% followed up<br><br>19-year follow-up	Prenatal<br><br>High risk of poor developmental outcomes due to parents living in poverty	Mothers' median age 20 years (range 12–34 years)<br><br>66% European American<br><br>All mothers were living in poverty	12, 18 and 24 months<br><br>Some tapes were missing so attachment pattern was determined from data available	SSP [Ainsworth (1978);[8] Main and Solomon (1990)[6]]<br><br>40% disorganised, 36% secure, 12% avoidant, 12% resistant

SD, standard deviation.

© Queen's Printer and Controller of HMSO 2015. This work was produced by Wright *et al.* under the terms of a commissioning contract issued by the Secretary of State for Health. This issue may be freely reproduced for the purposes of private research and study and extracts (or indeed, the full report) may be included in professional journals provided that suitable acknowledgement is made and the reproduction is not associated with any form of advertising. Applications for commercial reproduction should be addressed to: NIHR Journals Library, National Institute for Health Research, Evaluation, Trials and Studies Coordinating Centre, Alpha House, University of Southampton Science Park, Southampton SO16 7NS, UK.

The eight studies were included as they met the following criteria:

- Four of the eight studies reported long-term data in relation to severe attachment problems at baseline.[45,151–153]
    - Two studies measured the stability of attachment over time.[151,152]
    - Two studies examined the relationship between severe attachment problems in infancy and later mental health problems.[45,155]
- Four studies met the criteria in terms of reporting long-term outcomes and measured severe attachment problems at inception, but did not report outcomes of disorganised attachment separately; therefore, the data have not been presented.[154–157]

The prevalence data for the study by Carlson and colleagues[153] are not included in *Table 10*, as although this study reports some data on prevalence at baseline, these are reported as a mean rating score across the sample and were not therefore available in a usable format.

Population characteristics

Three of the papers reviewed[45,152,153] were part of the Minnesota Longitudinal Study of Risk and Adaptation,[158] a large ongoing study in the USA. The other studies were conducted in various different countries but the predominant ethnicity was Caucasian, with the exception of an Israeli study with a 100% Jewish population.[155] The length of follow-up ranged from 10 to 28 years.

Table 10 shows the sample size at enrolment and follow-up, which also varied among studies. Sample size at follow-up ranged from 21[154] to 162[153] and attrition ranged from 11%[156] to 82%.[155]

Three studies successfully followed up more than 60%[153,154,156] and only one study managed to successfully follow up more than 70%.[156] The participant information is based on the sample of participants that was followed up longitudinally. This explains slight differences in participant details in the Minnesota papers, which all followed up infants recruited as part of the same original sample. All participants were recruited at 12 months of age or under. The Minnesota sample was defined as being at risk of poor developmental outcomes because the parents were living below the poverty line at the time of birth. Most other samples comprised participants from middle-class backgrounds. Two of these papers described their samples as low risk.[151,157] Although the authors were not explicit about what 'low risk' means, they appeared to be making reference to the SES of the parents. There was one population study that reflected all infants born during a 1-year period in a geographical region. Dan and colleagues[155] approached all parents who had given birth, within the same year, in Greater Haifa, Israel. Thus, their sample covered the full range of SES from that region.

Rates of disorganised attachment in the various papers reviewed

Table 10 shows the prevalence of disorganised attachment patterns in the various samples of the papers found for this phase, as well as the organised patterns of attachment. The papers that defined their samples as at risk owing to poverty reported considerably higher prevalence of disorganised attachment than the other studies (35%[45] and 40%[152]).

Similarly, the two papers in which the sample was described as low risk[151,157] had some of the lowest prevalence of disorganised attachment. Steele and colleagues[157] reported that 8% of participants showed a disorganised attachment pattern with their mother and just 2% with their father. Aikins and colleagues[151] reported that 11% of their sample had a disorganised pattern. Interestingly, one of the lowest reported prevalences of disorganised attachment was found in Dan and collagues'[155] sample. Dan and colleagues[155] found that only 3% of their sample, which included families from the whole population of Greater Haifa in Israel, had disorganised attachment patterns.

Ammaniti and colleagues[154] found that 20% of their middle-class sample had disorganised attachment patterns, and Jaffari-Bimmel and colleagues[156] found that 16% of their sample, who had been adopted internationally into middle-class families, had a disorganised attachment pattern. The variability in rates of disorganised attachment reflects the very different populations studied.

Quality assessment

Table 11 summarises the quality of the studies. Some items were rated as low bias fairly consistently across the studies. For example, all studies used the same measure of attachment across all participants.

Two items assessed the methods of selecting study participants. These items asked whether or not the cohorts were comparable by source and by age (items 1 and 2, respectively). Only one study[154] drew its cohort from different samples/sources (item 1). The majority recruited from the same sources. Two were unclear.[151,155] All the papers showed that the samples were recruited at the same age (item 2); however, it was unclear whether or not the groups in two papers from the Minnesota study[45,152] were comparable by age. Although the groups were recruited and assessed for severe attachment problems at the same age, data from some of the initial assessments were lost, and so the data analysed were, consequently, a mixture of two or three different assessment periods, conducted at different ages, from whatever data were available.

Items 3–7 assessed the methods of measuring exposure and outcome variables. Items relating to the measurement of exposure considered the ascertainment of exposure, blinding of coders measuring exposure and whether or not a description of exposure was provided for the entire sample at baseline (items 3, 4 and 6, respectively). The same validated measurement of attachment organisation or presence of RAD/DAD was used across the sample in all the papers (item 3). The majority of papers demonstrated that the coders of the initial attachment measure were blind to risk factors or predictive variables related to the exposure status (item 4). Two papers were unclear.[154,155] Two papers[156,157] provided attachment classifications for the full original sample (item 6). The remainder did not report these data.

Items relating to the measurement of outcomes considered whether or not outcomes of interest were present at the start of the study, and whether or not coders of outcome measures were blind to exposure status (items 5 and 7, respectively). The findings from item 5 have not been displayed in *Table 11* because this item was not considered to be applicable to any of the papers. It asked whether or not there was demonstration that outcome(s) of interest were present at the start of the study. Participants were recruited at an early age in all the papers and outcomes measured in late childhood, adolescence and adulthood were deemed not relevant or measurable in infancy, so item 5 could not be applied to any of the papers. Three papers[151,152,157] reported that subsequent outcome measures were rated by blind coders who were not aware of attachment status (item 7). The remainder did not report this information.

Items 8–11 assessed design-specific sources of bias. Items considered baseline differences between participants retained and those lost to follow-up, and whether or not analysis was conducted to compensate. Most of the papers tested for and reported no significant differences at baseline between participants followed up and those lost to attrition (item 8). Only one paper[154] provided insufficient information to judge whether or not baseline differences were present. Item 9 asked whether or not any analysis was conducted to compensate for significant differences at baseline. All papers, with the exception of Ammaniti and colleagues,[154] were rated lower on this item because they reported no significant baseline differences. Ammaniti and colleagues[154] reported insufficient information to permit judgement. Most of the studies had high attrition rates (item 10). Papers were rated as low bias on this item if they reported less than 20% attrition or provided a description of participants lost to follow-up; this applied to only one paper.[156] The study by Dan and colleagues[155] was unclear. Although several of the studies did report that there were no significant differences in attachment status between those retained and those lost to attrition, it could not be determined whether or not there were differences in the reasons for attrition between exposed and unexposed participants in any of the papers (item 11).

© Queen's Printer and Controller of HMSO 2015. This work was produced by Wright *et al.* under the terms of a commissioning contract issued by the Secretary of State for Health. This issue may be freely reproduced for the purposes of private research and study and extracts (or indeed, the full report) may be included in professional journals provided that suitable acknowledgement is made and the reproduction is not associated with any form of advertising. Applications for commercial reproduction should be addressed to: NIHR Journals Library, National Institute for Health Research, Evaluation, Trials and Studies Coordinating Centre, Alpha House, University of Southampton Science Park, Southampton SO16 7NS, UK.

TABLE 11 Table illustrating the quality assessment of included studies

	Method of selecting study participants		Methods of measuring exposure and outcome variables				Design-specific sources of bias				Conflict of interest	Any other bias
**Author and year**	**1**	**2**	**3**	**4**	**6**	**7**	**8**	**9**	**10**	**11**	**12**	**13**
Steele *et al.* (2002)[157]	Low	Low	Low	Low	Low	Low	Low	Low	High	Unclear	Unclear	Low
Jaffari-Bimmel *et al.* (2006)[156]	Low	Low	Low	Low	Low	Unclear	Low	Low	Low	Unclear	Unclear	High
Carlson *et al.* (2009)[153]	Low	Low	Low	Low	High	Unclear	Low	Low	High	Unclear	Unclear	Low
Aikins *et al.* (2009)[151]	Unclear	Low	Low	Low	High	Low	Low	Low	High	Unclear	Unclear	High
Weinfield *et al.* (2004)[152]	Low	Unclear	Low	Low	High	Low	Low	Low	High	Unclear	Unclear	High
Dan *et al.* (2011)[155]	Unclear	Low	Low	Unclear	High	Unclear	Low	Low	Unclear	Unclear	Unclear	Low
Carlson (1998)[45]	Low	Unclear	Low	Low	High	Unclear	Low	Low	High	Unclear	Unclear	High
Ammaniti *et al.* (2005)[154]	High	Low	Low	Unclear	High	Unclear	Unclear	Unclear	High	Unclear	Unclear	High

Note
Item 5 does not apply to studies of this nature and so it is not included.

Item 12 considered the declaration of conflicts of interest and item 13 checked for any other bias. It was unclear whether or not there were any conflicts of interest or funding sources that may cause bias in any of the papers. At least one source of important bias, not covered by the first 12, was found in five of the eight papers. Aikins and colleagues[151] received a rating of high bias on this item because prevalence figures were inconsistent with percentages calculated. Jaffari-Bimmel and colleagues[156] conducted an analysis to compensate for missing data but did not report the extent of missing data. In addition to this, part, but not all, of the sample received an attachment-based intervention at 5 months old. Ammaniti and colleagues[154] did not report results for the full sample on all outcome measures. Two papers received a rating of high bias for inconsistent reporting about the sample.[45,152]

Long-term outcomes

Two studies examined whether or not attachment problems continued from infancy to adolescence (*Table 12*). The two studies measure adolescent attachment at different ages, using different tools. Aikins and colleagues[151] reported that there was no continuity from disorganised infant attachment on the SSP to unresolved adolescent attachment on the Adult Attachment Projective Picture System.

Weinfield and colleagues[152] reported that infants with disorganised attachment were significantly more likely to be insecurely attached in late adolescence, as measured by the Berkeley AAI,[160] and less likely to be autonomous than participants who were organised in their attachment during infancy, with 86% of those who were disorganised in infancy classified as insecure on the AAI. However, of the 42 participants classified as disorganised in infancy, only nine were unresolved in adolescence. The unresolved classification was

TABLE 12 Summary of findings of attachment stability as a long-term outcome

Author and year	Age when assessed by SSP	Name of outcome and measurement tool	Age at follow-up	Summary of findings
Aikins *et al.* (2009)[151]	12 months	Adult attachment<br><br>AAP Picture System[159]	16 years	Conducted a chi-squared analysis to examine whether or not disorganised infant attachment predicted unresolved adolescent attachment<br><br>The finding was not significant: χ^2 (1, $n = 47$) = 0.33
Weinfield *et al.* (2004)[152]	12 and 18 months. Some tapes were missing so attachment pattern was determined from data available	Adult attachment<br><br>Berkeley AAI (George C, Kaplan N, Main M, 1985, unpublished data)	19 years	Conducted a chi-squared analysis to examine whether or not disorganised infant attachment predicted insecure adolescent attachment<br><br>The findings were significant: χ^2 (3) = 0.877 ($p = 0.032$)<br><br>A bivariate correlation were conducted to explore relations between disorganised attachment and unresolved loss<br><br>The finding was not significant: r (106) = 0.07<br><br>A bivariate correlation were conducted to explore relations between disorganised attachment and unresolved abuse<br><br>The finding was significant: r (32) = 0.48

AAP, Adult Attachment Projective.

© Queen's Printer and Controller of HMSO 2015. This work was produced by Wright *et al.* under the terms of a commissioning contract issued by the Secretary of State for Health. This issue may be freely reproduced for the purposes of private research and study and extracts (or indeed, the full report) may be included in professional journals provided that suitable acknowledgement is made and the reproduction is not associated with any form of advertising. Applications for commercial reproduction should be addressed to: NIHR Journals Library, National Institute for Health Research, Evaluation, Trials and Studies Coordinating Centre, Alpha House, University of Southampton Science Park, Southampton SO16 7NS, UK.

categorised further into unresolved 'loss' and unresolved 'abuse'. Weinfield and colleagues[152] explored the relationship between a disorganised attachment pattern on the SSP in infancy and unresolved loss and unresolved abuse in adolescence, only in those participants who discussed abuse and loss. They suggested that disorganised attachment was related to unresolved abuse but not unresolved loss.

The instruments used here for measuring attachment in later life (Adult Attachment Projective Picture System;[151] Berkeley AAI[152]) measure constructs suggested by their authors as being related to attachment. Validity testing is difficult because attachment is likely to change over a child's lifetime and be influenced by a range of factors. The tests used may also be assessing related or other constructs.

Table 13 shows the findings on long-term mental health outcomes of 'severe attachment problems'. Only two papers reported long-term outcomes of 10 years or more in relation to disorganised attachment. Of all outcomes searched for, three findings were found; all were in relation to mental health and all from the Minnesota sample. The findings were that disorganised attachment in infancy was significantly correlated with overall history of psychopathology at age 17 years,[45] dissociative experiences at age 19 years[45] and borderline personality symptoms at age 28 years.[153] This last association was weak, and lost its significance when included with maternal hostility and other factors in a regression analysis.

These findings were surprisingly limited, and so after submission of the draft report, additional scoping of the literature exploring 5- to 10-year follow-up was conducted. Although this was not comprehensive or systematic (because of very limited time scales), some findings are included in *Tables 29* and *30* in *Appendix 4* for reference.

TABLE 13 Summary of findings for mental illness as a long-term outcome

Author and year	Name of outcome and measurement tool	Age of participant (years)	Summary of findings
Carlson (1998)[45]	Overall history of psychopathology<br><br>K-SADS-E	17.5	Correlational analyses found disorganised attachment in infancy was related to overall history of psychopathology<br><br>$r\,(127) = 0.34$ ($p < 0.001$)
Carlson (1998)[45]	Dissociative experiences<br><br>Dissociative Experiences Scale[161]	19	Correlational analyses found disorganised attachment in infancy was related to concurrent self-report of dissociative episodes<br><br>$r\,(126) = 0.36$ ($p < 0.001$)
Carlson *et al.* (2009)[153]	Borderline personality disorder SCID[162]	28	Correlational analyses found disorganised attachment in infancy was significantly correlated with borderline personality symptoms at 28 years<br><br>$r\,(122) = 0.20$ ($p < 0.05$)

K-SADS-E, Kiddie Schedule for Affective Disorders and Schizophrenia Rating; SCID, Structured Clinical Interview for DSM Disorders.

Conclusion

Eight of the 10-year follow-up studies provided prevalence data for disorganised attachment.[45,151–157] In these papers, prevalence of disorganised infant attachment patterns was higher in populations where parents were living in poverty than in middle-class populations. Findings in terms of long-term outcomes of severe attachment problems are limited, owing to a lack of studies with long-term follow-ups. There are no longitudinal cohort studies that follow up children with a diagnosis of attachment disorders for 10 years or more. Four studies were found that measured long-term outcomes of disorganised attachment.[45,151–153] Two studies examined the continuity of disorganised attachment from infancy to adolescence. Aikins and colleagues[151] found no correlation between disorganised infant attachment and unresolved adolescent attachment on the Adult Attachment Projective Picture System. Using the Berkeley AAI, Weinfield and colleagues[152] found a correlation, in those who discussed abuse and loss, between disorganised infant attachment and unresolved abuse, but not unresolved loss, in adolescence.

Two papers, both from the Minnesota study, examined the relationship between disorganised infant attachment and long-term mental health outcomes. Carlson[45] found that disorganised attachment was significantly correlated with an overall history of psychopathology at age 17 years and dissociative episodes at age 19 years. Carlson and colleagues[153] found that disorganised attachment was significantly correlated with borderline personality symptoms at age 28 years. A separate review by Van IJzendoorn and Bakermans-Kranenburg,[23] using a meta-analysis, found an overall effect size of $r = 0.29$ across 12 studies for an association between disorganised attachment and childhood behaviour problems.[24,31,45,163–171]

As Green and Goldwyn[147] pointed out, the high base rates of attachment insecurity in the general population of up to 40% have made it difficult to use this as a predictor of psychopathology. Although disorganised attachment is less common and has associations with future psychopathology and other problems, it also encompasses a heterogeneous group in terms of the behaviours displayed in the SSP.[147] Furthermore, the stability of disorganised attachment assessed in a systematic review[23] is not high across a mean of 25 months over several included studies ($r = 0.34$), nor is there a strong association between attachment patterns with one caregiver and another.[23,147,172] There are also differences in inter-rater reliability (e.g. 0.76 in one study[173]). These do not invalidate the usefulness of the concept of attachment and the great diversity of insights that this literature affords us, but they do present challenges when we wish to use attachment patterns as markers of children who require intervention or as markers of outcome.

The large differences in categorisation make systematic review work extremely difficult, as variability in constructs and nomenclature may lead to the presentation of results in very different ways. For the purposes of this review, as severe attachment problems have been identified as attachment disorders or disorganised attachment patterns, we have only included children in these groups unless an alternative classification system very clearly maps on to disorganised attachment patterns. We have done this for the purposes of scientific clarity, although we recognise that this may exclude some papers that are considered by some authors to be relevant to this body of work.

© Queen's Printer and Controller of HMSO 2015. This work was produced by Wright *et al.* under the terms of a commissioning contract issued by the Secretary of State for Health. This issue may be freely reproduced for the purposes of private research and study and extracts (or indeed, the full report) may be included in professional journals provided that suitable acknowledgement is made and the reproduction is not associated with any form of advertising. Applications for commercial reproduction should be addressed to: NIHR Journals Library, National Institute for Health Research, Evaluation, Trials and Studies Coordinating Centre, Alpha House, University of Southampton Science Park, Southampton SO16 7NS, UK.

Chapter 6 Main systematic review: early parenting interventions for families of children with severe attachment problems

Introduction

The research objective of the main systematic review was to examine the clinical effectiveness of intervention programmes designed for parents of children with severe attachment problems.

This phase of the review is concerned with the identification and examination of intervention programmes designed for parents of children with severe attachment problems. We have examined interventions for children already showing severe attachment problems, defined as a diagnosis of an attachment disorder or a disorganised attachment pattern. We have also considered interventions aimed at preventing the development of such problems.

Methods

We developed a search strategy using a combination of two concepts to capture the patient group (children with 'severe attachment problems' as defined in *Chapter 1*) and the interventions of interest, according to the guidelines for exhaustive searching prepared by the CRD and Cochrane.[174] After an initial scoping exercise demonstrating very large numbers of papers with small, uncontrolled descriptions of interventions, the decision was made to limit this phase to RCTs only. PPI groups with experts and service users were held to ensure that any interventions that were predominant in the field that may not have been validated using a RCT were discussed, although they would not enter the systematic review.

Initially, titles and abstracts were reviewed independently, with disagreements discussed and resolved between reviewers and a third party when required. The identified literature was dual screened according to the screening criteria specified below (see *Inclusion criteria*). Papers were obtained when eligibility could not be ascertained and disagreements were discussed and resolved by a third party. In instances where a foreign language paper was identified, translation then screening was performed as above.

Inclusion criteria

The PICOS criteria were as follows:

- *Population* Parents or caregivers of young children (with a mean age under 13 years) who have severe attachment problems (as defined in *Chapter 1*) or are at high risk of developing such problems (e.g. children with a history of maltreatment).
- *Intervention* Interventions were aimed at parents or caregivers, including foster carers. Interventions were excluded if aimed at teachers or teaching assistants (without parents or caregivers) or not focused at an individual level (e.g. organisational structure change in a care setting).
- *Comparators* No intervention, an attention control or usual care.
- *Primary outcome* The child's attachment to the caregiver. Although we felt that it was important to examine the studies identified with a disorganised (D) pattern of attachment or a diagnosis of attachment disorder (e.g. RAD/DAD), we felt that it was also essential to examine the studies that look at the promotion of attachment security. Therefore, we did not specify that it was a necessary requirement for studies to contain a D classification or RAD/DAD diagnosis if attachment pattern was measured.

© Queen's Printer and Controller of HMSO 2015. This work was produced by Wright *et al.* under the terms of a commissioning contract issued by the Secretary of State for Health. This issue may be freely reproduced for the purposes of private research and study and extracts (or indeed, the full report) may be included in professional journals provided that suitable acknowledgement is made and the reproduction is not associated with any form of advertising. Applications for commercial reproduction should be addressed to: NIHR Journals Library, National Institute for Health Research, Evaluation, Trials and Studies Coordinating Centre, Alpha House, University of Southampton Science Park, Southampton SO16 7NS, UK.

Measures of attachment were relevant if the measure attempted to assess the nature of the child's attachment to the caregiver. Only whole attachment instruments or instruments that included a subscale measuring attachment were included. For inclusion in the review, the primary or principal aim of the tool must focus on the measurement of attachment pattern or attachment disorder.

- *Secondary outcomes* Quality of life; psychological well-being; rates of mental ill health at any age; psychosocial development; educational attainment; entry into care or the criminal justice system; acceptability of the intervention to parents. We sought validated outcome measures in any of these domains. [All secondary outcomes, with the exception of parenting, are related to the outcome for the child and not the parent (i.e. we are interested in the child's psychological morbidity and not that of the parents).]
- *Study design* RCTs.

Any paper that did not meet the above criteria but provided additional information to that given by a paper already included was automatically included in the review.

Conducting the quality assessment

The quality assessment was conducted with the risk-of-bias assessment for RCTs using the criteria recommended by the Cochrane Handbook.[174] The recommended approach for assessing risk of bias in studies included in the Cochrane Review is a two-part tool, addressing six specific domains:

- sequence generation and allocation concealment (selection bias)
- blinding of participants and providers (performance bias)
- blinding of outcome assessor (detection bias)
- incomplete outcome data (attrition bias)
- selective outcome reporting (reporting bias)
- other sources of bias.

The first part of the tool involves describing what was reported to have happened in the study. The second part of the tool involves assigning a judgement relating to the risk of bias for that entry, in terms of low, high or unclear risk. All quality assessments were conducted separately by two independent reviewers; any disagreements were resolved by arbitration and a third party when required.

Data extraction

Data were extracted by two independent reviewers and the information was standardised into an Excel® spreadsheet (2010; Microsoft Corporation, Redmond, WA, USA); reviewers subsequently met to agree the data extraction. This information formed the basis of the tables for the report. For non-English language studies, one reviewer conducted the translation using the specific template provided.

Where there were multiple publications for the same study, data were extracted primarily from the original complete publication. In cases in which the duplicate publications reported additional relevant data, these data were also extracted.

Demographic information

Demographic information was collected on a range of different variables. These included age of parent, age of child, ethnicity and SES.

Data synthesis

The results section is split into two main tables. The first looked at studies that have focused on promoting the security of attachment, without RAD or DAD or those with a disorganised pattern of attachment. The second focussed specifically on studies using a population with a disorganised pattern or a population with a diagnosis of RAD/DAD. This is also replicated in the meta-analysis.

Meta-analysis

For the meta-analysis we explored each dichotomous outcome (secure or disorganised attachment pattern as measured by the Ainsworth scale[6] or MacArthur Working Group scale[49]). We extracted data on the numbers of patients experiencing the outcome for each group. The odds ratio (OR) and 95% CI were calculated for each study outcome. The ORs were pooled using a fixed-effects model or random-effects model [the Mantel–Haenszel (M–H) method] and the corresponding 95% CIs were calculated. Statistical heterogeneity was assessed. This is the variability in the intervention effects being evaluated in the different studies. Statistical heterogeneity manifests itself in the observed intervention effects being more different from each other than one would expect from random error (chance) alone. Where the result indicated significant heterogeneity, a random-effects model was chosen; otherwise, a fixed-effects model was applied.

The data corresponding to the last reported follow-up were chosen. Subgroup analyses were undertaken for the following:

- duration of intervention
- length of follow-up
- number of sessions within the intervention (< 5, 5–16, > 16)
- age of child at the start of the intervention
- foster children involved
- middle class
- intervention location (home, mixed, other)
- male caregiver included
- video feedback provided
- intervention involving a component targeting maternal sensitivity
- primary focus to modify child attachment
- intervention involving caregiver and child together, caregiver and child separately, caregiver alone or mixed.

For continuous data, means, standard deviations (SDs) and sample sizes were extracted for each study. As a range of different outcome measures were used, Cohen's *d* effect sizes were calculated to allow comparison between studies, but no formal pooling of data was undertaken.

Funnel plot

A funnel plot is a method of investigating publication bias. It gives some idea of whether or not the study results are scattered symmetrically around a central, more precise effect. The vertical axis is a measure of the precision of the estimate of the treatment effect. Here, the measure of precision is the standard error of the log OR. The horizontal axis measures the treatment effect; here it is the OR on a log scale, so that the distance from 0.1 to 1 is the same as that from 1 to 10. The point estimate from each study is then plotted, and a vertical line added where the pooled estimate from the meta-analysis lies. We would expect less precise studies (with fewer participants and events) to be more affected by the play of chance, and so more widely scattered about the pooled estimate. As studies get bigger, with more events, we expect them to be closer to the pooled estimate. Overall, this should produce a triangular shape, or inverted funnel.

Results

Figure 5 summarises the results of the search. The database searches identified 15,621 records. After the records we deduplicated, this left 10,167 records for screening. Of these, 445 met first-sift inclusion criteria, including 21 which were identified through other sources. Of these 445 records, 39 met the final inclusion criteria.

© Queen's Printer and Controller of HMSO 2015. This work was produced by Wright *et al.* under the terms of a commissioning contract issued by the Secretary of State for Health. This issue may be freely reproduced for the purposes of private research and study and extracts (or indeed, the full report) may be included in professional journals provided that suitable acknowledgement is made and the reproduction is not associated with any form of advertising. Applications for commercial reproduction should be addressed to: NIHR Journals Library, National Institute for Health Research, Evaluation, Trials and Studies Coordinating Centre, Alpha House, University of Southampton Science Park, Southampton SO16 7NS, UK.

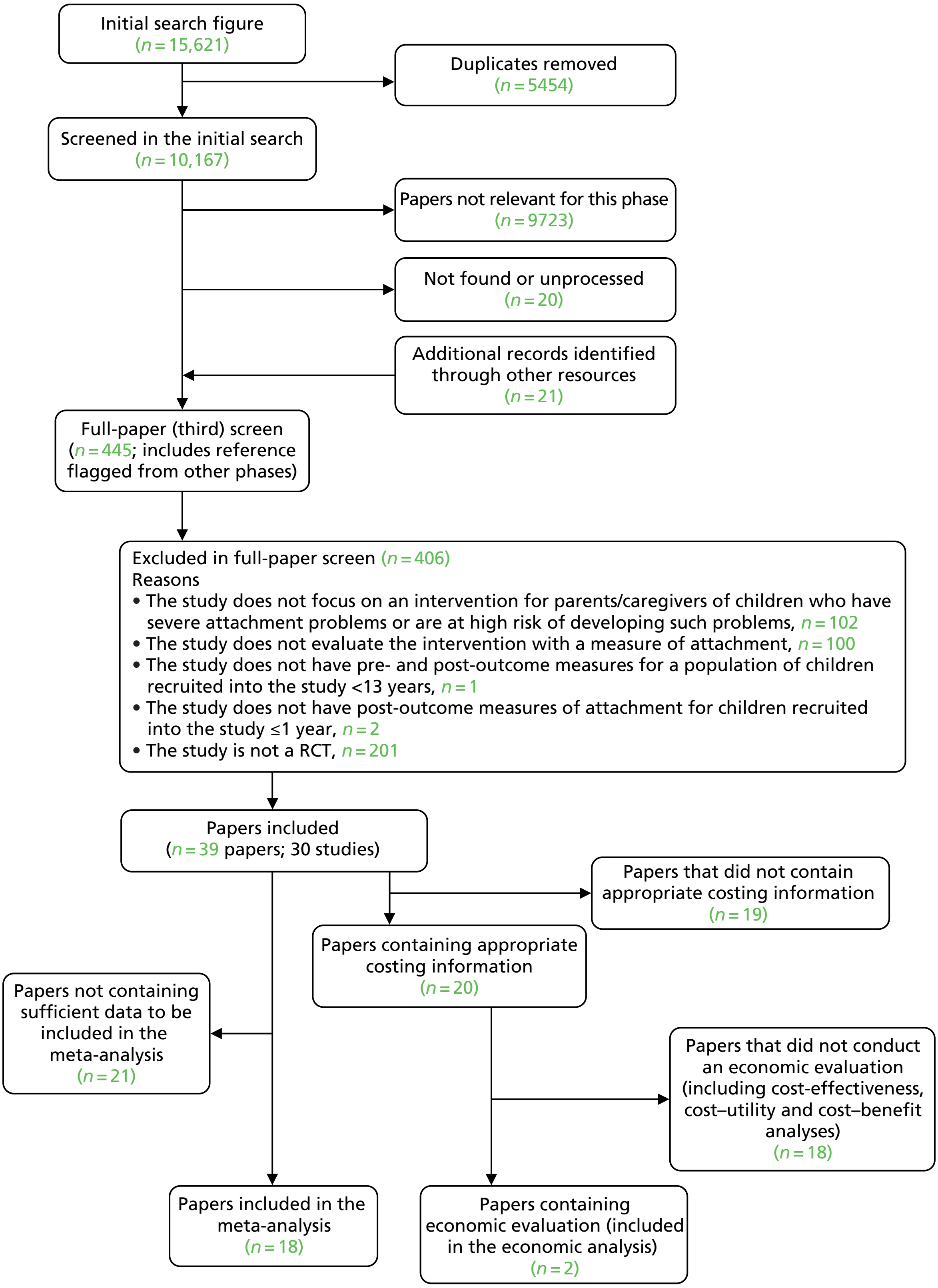

FIGURE 5 Preferred Reporting Items for Systematic Reviews and Meta-Analyses for the main systematic review.

Characteristics of included intervention studies

Table 14 shows the study, participant and intervention characteristics of included intervention studies without a disorganised classification or diagnosis of RAD. *Table 15* shows the study, participant and intervention characteristics of included intervention studies that contained a disorganised classification or diagnosis of RAD.

In total, 30 studies were identified,[175,178,180–184,188,189,191,194–196,198,200–203,205–208,211,212,218] 29 of which were delivering an intervention in a hypothesised 'at-risk' group to improve attachment security[129,175–178,180–186,189,191,194–196,198,200–203,205,206,208,211,212,218] and one of which provided treatment for children already with a diagnosis of RAD.[188] The studies were published between 1985 and 2012 and were undertaken in a variety of countries: five in the UK,[176,188,189,207,208] 14 in the USA,[177,180–182,184–186,191,194,196,200,201,205,211] three in the Netherlands,[178,198,203] three in Canada,[175,195,218] one in Germany,[202] one in Australia,[130] one in Italy,[206] one in Finland[212] and one in Lithuania.[183]

- Eight studies were identified in the main systematic review that evaluated the intervention using a measure that assessed a disorganised pattern.[189,190,194–196,198,200,218]
- Only one study was identified that was focused on a population of children with an attachment disorder diagnosis.[187]
- The remaining 21 studies evaluated the interventions with a measure that assessed only secure and insecure attachment patterns without a disorganised group.[129,175–178,180–186,201–209,211]

The characteristics of the populations in each study were described and classified into nine categories based on the reporting of authors' population descriptions and/or inclusion criteria. *Figure 6* shows the distribution of the population characteristics across the 30 included studies.

The nine population characteristic categories were:

- parent mental health
- low SES
- life events/homelessness
- child behavioural problems/disability/high irritability
- middle class
- poor parenting or parental sensitivity
- single/first-time/adolescent mothers
- low-birthweight/pre-term infants
- foster placement/child welfare/child's maltreatment history.

Figure 6 shows the study populations for the studies that did and did not contain a disorganised pattern of attachment or a diagnosis of attachment disorder, that is, those which were for children at high risk of severe attachment difficulties. Interestingly, the majority of the disorganised or disorder studies assessed interventions using a population where the parents were at higher risk of raising children with poor developmental outcomes, that is, where the parents either had a mental health problem or were single, first-time or adolescent mothers.

Ten conducted the intervention across a population with low SES, which was the largest category.[175,176,185,191,196,198,200,205,208,211] The other studies were distributed evenly between the other eight population categories.

© Queen's Printer and Controller of HMSO 2015. This work was produced by Wright *et al.* under the terms of a commissioning contract issued by the Secretary of State for Health. This issue may be freely reproduced for the purposes of private research and study and extracts (or indeed, the full report) may be included in professional journals provided that suitable acknowledgement is made and the reproduction is not associated with any form of advertising. Applications for commercial reproduction should be addressed to: NIHR Journals Library, National Institute for Health Research, Evaluation, Trials and Studies Coordinating Centre, Alpha House, University of Southampton Science Park, Southampton SO16 7NS, UK.

TABLE 14 Study and intervention characteristics: participants without a disorganised classification or diagnosis of RAD

Author, year and country of publication	Participants: parents/children	Sample characteristics: risk factor	Intervention name and description	Intervention duration/intensity/delivery (including age of child at delivery of intervention)	Control group description	Attachment measure
Ammaniti *et al.* (2006)[206]<br><br>Italy	$N = 110$ dyads<br><br>Depressive risk ($n = 36$), psychosocial risk ($n = 34$), low-risk group ($n = 40$)<br><br>Parent age: mean 32.5 years (SD 4.19 years), range 22–43 years<br><br>Child age: prenatal (2nd trimester)<br><br>Ethnicity not reported<br><br>SES not reported	Infants and their mothers divided into three risk groups:<br><br>1. depressive risk with one or no psychosocial risk factors<br>2. psychosocial risk and reported low level of depressive symptoms<br>3. low depressive and low psychosocial risk	Name: The Home Visiting Program<br><br>Aim: to enhance the mother's capacity to read and interpret the signals of the child<br><br>Description: home visits<br><br>Video feedback: no<br><br>Male caregiver involved: unclear<br><br>Location: home	Duration: not reported<br><br>Intensity: weekly in first semester then biweekly in second semester<br><br>Age of child at start: prenatal<br><br>Delivered by: psychologists	Received scheduled visits for data collection purposes only	SSP
Anisfeld *et al.* (1990)[180]<br><br>USA	$N = 60$ mothers<br><br>(I: $n = 23$; C: $n = 26$)<br><br>Parent age: I, mean 23.7 years (range and SD not reported); C, mean 24.5 years (range and SD not reported)<br><br>Child age: mean 2 days (range and SD not reported)<br><br>Ethnicity: predominantly Hispanic and black<br><br>SES: low income	Women from a low-income clinic population	Name: not reported<br><br>Aim: to create a secure and protective environment<br><br>Description (i): soft baby carriers provided<br><br>Video feedback: no<br><br>Male caregiver involved: no<br><br>Location: baby carrier can be used anywhere	Duration: not reported<br><br>Intensity: daily use<br><br>Age of child at start: 2 days<br><br>Delivered by: N/A	Plastic infant seats were provided instead of baby carriers	SSP

Author, year and country of publication	Participants: parents/children	Sample characteristics: risk factor	Intervention name and description	Intervention duration/intensity/delivery (including age of child at delivery of intervention)	Control group description	Attachment measure
Barnett *et al.* (1987);[129] Barnett and Parker (1985)[133]<br><br>Australia	$N = 90$ dyads (Ia, $n = 31$; Ib, $n = 30$; C, $n = 28$)<br><br>Parent age: Ia, mean 29.6 years (range 18–44 years) SD not reported; Ib, mean 28.7 years (range 18–44 years) SD not reported; C, mean 28.3 years (range 18–44 years) SD not reported<br><br>Child age: not reported<br><br>Ethnicity: Ia, 71% Australian; Ib, 77% Australian; C, 68% Australian<br><br>SES: middle class	Infants and their mothers with high trait anxiety	Name: Ia, professional intervention; Ib, non-professional intervention<br><br>Aim: Ia, to encourage maternal involvement; Ib, to provide support, help and advice<br><br>Description: Ia, (i) social worker home visits; Ib, (i) delivered by an experienced mother<br><br>Video feedback: no<br><br>Male caregiver involved: yes<br><br>Location: home	*Ia*<br><br>(i) duration 12 months<br><br>Intensity not reported<br><br>Age of child at start: not reported<br><br>Delivered by: social workers<br><br>*Ib*<br><br>(i) duration 12 months<br><br>Intensity not reported<br><br>Age of child at start: not reported<br><br>Delivered by: experienced mothers	Not reported	SSP
Beckwith (1988)[201]<br><br>USA	$N = 92$ families (I, $n = 37$; C, $n = 55$)<br><br>Parent age: mean 24 years (range and SD not reported)<br><br>Child age: newborn<br><br>Ethnicity: not reported<br><br>SES: not reported	Sick pre-term infants being reared by low-income parents	Name: UCLA Preterm Infant Study<br><br>Aim: to develop parent's observation skills<br><br>Description: an individualised and parent-directed home-visiting intervention<br><br>Video feedback: no<br><br>Male caregiver involved: yes<br><br>Location: hospital/home	Duration: 13 months<br><br>Intensity: not reported<br><br>Age of child at start: not reported<br><br>Delivered by: paediatric nurse and an early childhood educator	Not reported	Attachment security (measure not reported)

continued

© Queen's Printer and Controller of HMSO 2015. This work was produced by Wright *et al.* under the terms of a commissioning contract issued by the Secretary of State for Health. This issue may be freely reproduced for the purposes of private research and study and extracts (or indeed, the full report) may be included in professional journals provided that suitable acknowledgement is made and the reproduction is not associated with any form of advertising. Applications for commercial reproduction should be addressed to: NIHR Journals Library, National Institute for Health Research, Evaluation, Trials and Studies Coordinating Centre, Alpha House, University of Southampton Science Park, Southampton SO16 7NS, UK.

TABLE 14 Study and intervention characteristics: participants without a disorganised classification or diagnosis of RAD *(continued)*

Author, year and country of publication	Participants: parents/children	Sample characteristics: risk factor	Intervention name and description	Intervention duration/intensity/delivery (including age of child at delivery of intervention)	Control group description	Attachment measure
Brisch *et al.* (2003)[202]<br><br>Germany	$N = 87$ children (I, $n = 43$; C, $n = 44$)<br><br>Parent age: I, mean 30.9 years (SD 4.9 years), range 23–42 years; C, mean 30.9 years (SD 4.9 years), range 18–40 years<br><br>Child age: I, mean 27 weeks (SD 2.3 weeks), range 24–33 weeks' gestation; C: mean 27 weeks (SD 2.7 weeks), range 24–35 weeks' gestation<br><br>Ethnicity: 100% white<br><br>SES: 100% middle class	Middle-class white parents of pre-term babies	Name: PPIP<br><br>Aim: to improve parental coping and attachment<br><br>Description: (i) parent group, (ii) individual psychotherapy training sessions for both parents, (iii) sensitivity training, (iv) home visits<br><br>Video feedback: yes (one session)<br><br>Male caregiver involved: yes<br><br>Location: hospital	*Parent group*<br><br>Duration: fortnightly<br><br>Intensity: median = 5 sessions, range = 1–8 sessions, SD = 2.2 sessions<br><br>Age of child at start: preterm inpatient<br><br>*Individual psychotherapy training sessions for both parents*<br><br>Duration: fortnightly<br><br>Intensity: median = 5 sessions, range = 1–10 sessions, SD = 1.4 sessions<br><br>Age of child at start: preterm inpatient<br><br>*Sensitivity training*<br><br>Duration: 1-day video-feedback session<br><br>Intensity: 1 day<br><br>Age of child at start: 3 months	Preterm babies receiving usual hospital nursing care	SSP

Author, year and country of publication	Participants: parents/children	Sample characteristics: risk factor	Intervention name and description	Intervention duration/intensity/delivery (including age of child at delivery of intervention)	Control group description	Attachment measure
				Home visits<br><br>Duration: one home visit<br><br>Intensity: one extended home visit<br><br>Age of child at start: first week after discharge<br><br>*All delivered by a psychotherapist and a nurse from the NICU*		
Briskman and Scott (2012)[207]<br><br>UK	*N* = 77 carers (I, *n* = 42; C, *n* = 35)<br><br>Parent age: mean 50 years (SD 8 years), range 29–63 years<br><br>Child age: mean 7.9 years (SD 3.1 years), range 2–12 years<br><br>Ethnicity: 66.7% white British<br><br>SES: not reported	Foster carers and foster children	Name: Fostering Changes Programme<br><br>Aim: to enhance carer's relationship with his or her foster child<br><br>Description: structured group parenting skills course<br><br>Video feedback: no<br><br>Male caregiver involved: yes<br><br>Location: local authority venue	Duration: 12 weeks<br><br>Intensity: weekly, 180 minutes per session<br><br>Age of child at start: not reported<br><br>Delivered by: two experienced facilitators	Home visits, interviewed and asked to complete questionnaires	QUARQ (devised by an in-house research team)

continued

© Queen's Printer and Controller of HMSO 2015. This work was produced by Wright *et al.* under the terms of a commissioning contract issued by the Secretary of State for Health. This issue may be freely reproduced for the purposes of private research and study and extracts (or indeed, the full report) may be included in professional journals provided that suitable acknowledgement is made and the reproduction is not associated with any form of advertising. Applications for commercial reproduction should be addressed to: NIHR Journals Library, National Institute for Health Research, Evaluation, Trials and Studies Coordinating Centre, Alpha House, University of Southampton Science Park, Southampton SO16 7NS, UK.

TABLE 14 Study and intervention characteristics: participants without a disorganised classification or diagnosis of RAD (*continued*)

Author, year and country of publication	Participants: parents/children	Sample characteristics: risk factor	Intervention name and description	Intervention duration/intensity/delivery (including age of child at delivery of intervention)	Control group description	Attachment measure
Fisher and Kim (2007)[181]<br><br>USA	$N = 117$ children (I, $n = 57$; C, $n = 60$)<br><br>Parent age: not reported<br><br>Child age: I, mean 4.54 years (SD 0.86 years), range 3–5 years<br><br>Ethnicity: 89% European American<br><br>SES: not reported	3- to 5-year-old children new to foster care, re-entering foster care and moving between foster placements	Name: MTFC-P<br><br>Aim: to help foster parent maintain positive environment<br><br>Description: (i) intensive training for the foster parents, (ii) support through telephone contact, (iii) group meetings, (iv) therapeutic playgroup sessions for the infant, (v) behaviour specialist meetings, (vi) birth parent working with family therapist<br><br>Video feedback: no<br><br>Male caregiver involved: yes<br><br>Location: home and preschool day care	*Intensive training for the foster parents*<br><br>Duration: 12 hours<br><br>Intensity: not reported<br><br>Age of child at start: not reported<br><br>Delivered by: not reported<br><br>*Support through telephone contact*<br><br>Duration: not reported<br><br>Intensity: 24-hour on-call help<br><br>Age of child at start: not reported<br><br>Delivered by: foster parent consultant<br><br>*Group meetings*<br><br>Duration: not reported<br><br>Intensity: weekly<br><br>Age of child at start: not reported<br><br>Delivered by: not reported	Regular foster care	PAD[210]

Author, year and country of publication	Participants: parents/children	Sample characteristics: risk factor	Intervention name and description	Intervention duration/intensity/delivery (including age of child at delivery of intervention)	Control group description	Attachment measure
				Therapeutic playgroup sessions for the infant<br><br>Duration: 9–12 months<br><br>Intensity: weekly<br><br>Age of child at start: not reported<br><br>Delivered by: clinicians<br><br>*Behaviour specialist meetings*<br><br>Duration: 9–12 months<br><br>Intensity: not reported<br><br>Age of child at start: not reported<br><br>Delivered by: behavioural specialist<br><br>*Birth parent working with family therapist*<br><br>Duration: not reported<br><br>Intensity: not reported<br><br>Age of child at start: not reported<br><br>Delivered by: family therapist		

continued

© Queen's Printer and Controller of HMSO 2015. This work was produced by Wright *et al.* under the terms of a commissioning contract issued by the Secretary of State for Health. This issue may be freely reproduced for the purposes of private research and study and extracts (or indeed, the full report) may be included in professional journals provided that suitable acknowledgement is made and the reproduction is not associated with any form of advertising. Applications for commercial reproduction should be addressed to: NIHR Journals Library, National Institute for Health Research, Evaluation, Trials and Studies Coordinating Centre, Alpha House, University of Southampton Science Park, Southampton SO16 7NS, UK.

TABLE 14 Study and intervention characteristics: participants without a disorganised classification or diagnosis of RAD (*continued*)

Author, year and country of publication	Participants: parents/children	Sample characteristics: risk factor	Intervention name and description	Intervention duration/intensity/delivery (including age of child at delivery of intervention)	Control group description	Attachment measure
Forman *et al.* (2007)[182]<br><br>USA	$N = 120$ mothers (I, $n = 60$; C, $n = 60$)<br><br>Parent age: mean 30.6 years (SD 4.5 years) range not reported<br><br>Child age: mean 6.1 months (SD 0.7 months) range not reported<br><br>Ethnicity: European American<br><br>SES: not reported	Mothers with major depressive episode in post-partum period	Name: IPT<br><br>Aim: to address issues such as interpersonal conflict, social role transitions, loss and grief<br><br>Description: manualised IPT<br><br>Video feedback: no<br><br>Male caregiver involved: yes<br><br>Location: not reported	Duration: 12 weeks<br><br>Intensity: 12 sessions, 1 hour per session<br><br>Age of child at start: 6 months<br><br>Delivered by: experienced psychotherapist	Waitlist	AQS
Hansen and Ulrey (1988)[185]<br><br>USA	$N = 19$ children (I, $n = 10$; C, $n = 9$)<br><br>Parent age: not reported<br><br>Child age: mean and SD not reported, range 3–19 months<br><br>Ethnicity: 89% Caucasian<br><br>SES: 100% low-middle	Neuromotor-handicapped infants. All the families were classed as low and low-middle socioeconomic level	Name: not reported<br><br>Aim: to improve infant caregiver interaction<br><br>Description: massage technique training<br><br>Video feedback: no<br><br>Male caregiver involved: unclear<br><br>Location: infant development centre	Duration: not reported<br><br>Intensity: 3-hour sessions, twice a week<br><br>Age of child at start: not reported<br><br>Delivered by: not reported	Regular programme for early intervention	ASI profile (Foley and Hobin 1982)[213]

Author, year and country of publication	Participants: parents/children	Sample characteristics: risk factor	Intervention name and description	Intervention duration/intensity/delivery (including age of child at delivery of intervention)	Control group description	Attachment measure
Jacobson and Frye (1991)[186]<br><br>USA	*N* = 61 mothers (I, *n* = 31; C, *n* = 30)<br><br>Parent age: I, mean 21.5 years (SD 3.1 years), range 17–32 years; C, mean 22.2 years (SD 3.6 years), range 17–32 years<br><br>Child age: prenatal (third trimester)<br><br>Ethnicity: 93% white<br><br>SES: not reported	First-time mothers of at least 17 years of age participating in the federally funded WIC food supplementation programme	Name: not reported<br><br>Aim: to provide maternal support<br><br>Description: volunteer coach home visits<br><br>Video feedback: no<br><br>Male caregiver involved: no<br><br>Location: home	Duration: not reported<br><br>Intensity: monthly and then weekly, decreasing monthly until the baby was 1 year old<br><br>Age of child at start: prenatal<br><br>Delivered by: volunteer coaches	Not reported	AQS
Kalinauskiene *et al.* (2009)[183]<br><br>Lithuania	*N* = 54 mothers (I, *n* = 26; C, *n* = 28)<br><br>Parent age: mean 26.4 (SD 2.94) years, range not reported<br><br>Child age: I, mean 6.12 (SD 0.08) months, range not reported; C, mean 6.11 (SD 0.06) months, range not reported<br><br>Ethnicity: 77.8% Lithuanian<br><br>SES: 100% middle class	Non-clinical, middle-class mothers with low sensitivity as classified by observation and rating scale	Name: VIPP<br><br>Aim: to reinforce mother's sensitive responses<br><br>Description: (i) video-interactive feedback to promote positive parenting, (ii) baby's diary completed by parent to monitor crying, fussing, sleeping and satisfied behavioural states and caregiver's reactions, (iii) booster session with the father and mother together<br><br>Video feedback: yes (five sessions)<br><br>Male caregiver involved: yes<br><br>Location: home	VIPP<br><br>Duration: 5 months<br><br>Intensity: monthly, 90 minutes per session<br><br>Age of child at start: 7 months<br><br>Delivered by: psychologists with MA in clinical psychology<br><br>*Baby's diary completed by parent*<br><br>Duration: not reported<br><br>Intensity: 3 days before each session<br><br>Age of child at start: 7 months<br><br>Delivered by: N/A (completed by caregiver)	Monthly telephone contact for 5 months asking for information on the infant's development	AQS

continued

© Queen's Printer and Controller of HMSO 2015. This work was produced by Wright *et al.* under the terms of a commissioning contract issued by the Secretary of State for Health. This issue may be freely reproduced for the purposes of private research and study and extracts (or indeed, the full report) may be included in professional journals provided that suitable acknowledgement is made and the reproduction is not associated with any form of advertising. Applications for commercial reproduction should be addressed to: NIHR Journals Library, National Institute for Health Research, Evaluation, Trials and Studies Coordinating Centre, Alpha House, University of Southampton Science Park, Southampton SO16 7NS, UK.

TABLE 14 Study and intervention characteristics: participants without a disorganised classification or diagnosis of RAD (*continued*)

Author, year and country of publication	Participants: parents/children	Sample characteristics: risk factor	Intervention name and description	Intervention duration/intensity/delivery (including age of child at delivery of intervention)	Control group description	Attachment measure
				Booster session with the father and mother together<br><br>Duration: not reported<br><br>Intensity: not reported<br><br>Age of child at start: 7 months<br><br>Delivered by: psychologists with MA in clinical psychology		
Klein-Velderman *et al.* (2006),[203] Klein-Velderman *et al.* (2006)[204]<br><br>the Netherlands	$N = 81$ dyads [Ia (VIPP), $n = 28$; Ib (VIPP-R), $n = 26$; C, $n = 27$]<br><br>Parent age: mean 27.8 (SD 3.63) years, range not reported<br><br>Child age: unclear<br><br>Ethnicity: not reported<br><br>SES: not reported	Mothers who were tentatively classified as insecure or coded as dismissing or preoccupied on the AAI, with their firstborn infant	Name: Ia, VIPP; Ib, VIPP-R<br><br>Aim: to break the intergenerational cycle of insecure attachment<br><br>Description: Ia, (i) video feedback and brochures; Ib, (i) video feedback and brochures, (ii) additional discussions about the mother's attachment experiences<br><br>Video feedback: yes (three sessions)<br><br>Male caregiver involved: no<br><br>Location: home	*Ia*<br><br>(i) Duration: not reported<br><br>Intensity: four home visits lasting 90 minutes<br><br>Age of child at start: mean 6.83 months (SD 1.03 months), range not reported<br><br>Delivered by: female degree-educated home visitors<br><br>*Ib*<br><br>(i)(ii) Duration: not reported<br><br>Intensity: four visits lasting 180 minutes<br><br>Age of child at start: mean 6.83 months (SD 1.03 months), range not reported<br><br>Delivered by: female degree-educated home visitors	Filming in the home during mother–infant interactions	SSP<br><br>AQS

Author, year and country of publication	Participants: parents/children	Sample characteristics: risk factor	Intervention name and description	Intervention duration/intensity/delivery (including age of child at delivery of intervention)	Control group description	Attachment measure
Lieberman *et al.* (1991)[211]<br><br>USA	*N* = 59 dyads (I, *n* = 34; C, *n* = 25)<br><br>Parent age: mean 25.08 years (SD not reported), range 21–39 years<br><br>Child age: mean and SD not reported, range 11–14 months<br><br>Ethnicity: Latin American immigrants<br><br>SES: 100% low	Recent Latino immigrants of low SES	Name: not reported<br><br>Aim: to provide the mother with a corrective attachment experience<br><br>Description: mother–infant psychotherapy<br><br>Video feedback: no<br><br>Male caregiver involved: no<br><br>Location: home	Duration: 12 months<br><br>Intensity: weekly 90-minute sessions<br><br>Age of child at start: 12 months<br><br>Delivered by: women with master's degrees in psychology or social work and clinical experience	Monthly telephone contact	AQS<br><br>Avoidance–resistance (using Ainsworth interactive behaviour scale 1978[8])

continued

© Queen's Printer and Controller of HMSO 2015. This work was produced by Wright *et al.* under the terms of a commissioning contract issued by the Secretary of State for Health. This issue may be freely reproduced for the purposes of private research and study and extracts (or indeed, the full report) may be included in professional journals provided that suitable acknowledgement is made and the reproduction is not associated with any form of advertising. Applications for commercial reproduction should be addressed to: NIHR Journals Library, National Institute for Health Research, Evaluation, Trials and Studies Coordinating Centre, Alpha House, University of Southampton Science Park, Southampton SO16 7NS, UK.

TABLE 14 Study and intervention characteristics: participants without a disorganised classification or diagnosis of RAD (*continued*)

Author, year and country of publication	Participants: parents/children	Sample characteristics: risk factor	Intervention name and description	Intervention duration/intensity/delivery (including age of child at delivery of intervention)	Control group description	Attachment measure
Murray *et al.* (2003),[208] Cooper *et al.* (2003)[209]<br><br>UK	$N = 193$ mothers (Ia, counselling $n = 48$; Ib, CBT $n = 43$; Ic, psychodynamic $n = 50$; control, $n = 52$)<br><br>Parent age: Ia, mean 28.4 years (SD 5.3 years), range 20–38 years; Ib, mean 27.9 years (SD 5.4 years), range 17–39 years; Ic, mean 28.1 years (SD 5.6 years), range 17–42 years; C, mean 26.5 years (SD 5.1 years), range 18–36 years<br><br>Child age: not reported<br><br>Ethnicity: not reported<br><br>SES: 25% high social disadvantage	Mothers with major depressive episode in postpartum period	Name: Ia, counselling; Ib, CBT; Ic, brief psychodynamic psychotherapy<br><br>Aim: Ia, to encourage mothers to express concerns about their infant; Ib, directed at problems identified as associated with mother–child interaction; Ic, to explore mother's own attachment history<br><br>Description: Ia, non-directive counselling; Ib, CBT; Ic, brief psychodynamic psychotherapy<br><br>Video feedback: no<br><br>Male caregiver involved: no<br><br>Location: home	*Ia, Ib, Ic*<br><br>Duration: 10 weeks<br><br>Intensity: weekly<br><br>Age of child at start: 2 months<br><br>Delivered by six therapists for all three treatments, a specialist in each of the three treatments and three non-specialists	Routine primary care – usual care provided by the primary health care team	SSP
Niccols (2008)[175]<br><br>Canada	$N = 76$ dyads (I, $n = 48$; C, $n = 28$)<br><br>Parent age: mean 28.8 years (SD 6.2 years), range 18–40 years<br><br>Child age: mean 8.4 months (SD 5.4 months), range 1–24 months<br><br>Ethnicity: not reported<br><br>SES: 52.6% low	Mothers varied in age, education and SES (53% low SES); 30% single-parent status. They had slightly lower maternal sensitivity scores than a typical sample and the infants had slightly lower than typical security	Name: RFTS<br><br>Aim: to improve infant attachment security and maternal sensitivity<br><br>Description: parent group<br><br>Video feedback: no<br><br>Male caregiver involved: no<br><br>Location: convenient locations with free parking	Duration: 8 weeks<br><br>Intensity: eight sessions, 2 hours per session<br><br>Age of child at start: not reported<br><br>Delivered by: infant development specialists	Treatment as usual (home visiting)	AQS

Author, year and country of publication	Participants: parents/children	Sample characteristics: risk factor	Intervention name and description	Intervention duration/intensity/delivery (including age of child at delivery of intervention)	Control group description	Attachment measure
O'Connor *et al.* (2012)[176]<br><br>UK	$N = 174$ children (I, $n = 88$; C, $n = 86$)<br><br>Parent age: not reported<br><br>Child age: I, mean 66.4 months (SD 5.9 months) months, range not reported; C, mean 65.7 months (SD 5.5 months), range not reported<br><br>Ethnicity: I, 43% black African; C, 48% black African<br><br>SES: recruited from the most disadvantaged ward within a deprived inner-London borough	Recruited from the most disadvantaged ward within a deprived inner-London borough. Sample included a mixture of children at high risk of emotional and behavioural issues and a normative sample	Name: Incredible Years parent programme. Also used the SPOKES manual literacy programme<br><br>Aim: to improve parenting across diverse interaction settings<br><br>Description: (i) parents in a group format, videotapes, observations, group discussions and role plays; (ii) literacy programme – SPOKES taught the pause–prompt–praise technique<br><br>Video feedback: no<br><br>Male caregiver involved: yes<br><br>Location: home	*Parents in group format*<br><br>Duration: 12 weeks<br><br>Intensity: 18 sessions<br><br>Age of child at start: not reported<br><br>Delivered by: a leader and coleader. The leader had a psychology degree and master's degree in child development, coleaders were mental health professionals in training<br><br>*Literacy programme*<br><br>Duration: 6 weeks<br><br>Intensity: not reported<br><br>Age of child at start: not reported<br><br>Delivered by a leader and coleader. The leader had a psychology degree and master's degree in child development, coleaders were mental health professionals in training	General practitioner, school-based drop-in service and specialist mental health service available for both intervention and control	MCAST (Green *et al.* 2000)[214]

continued

© Queen's Printer and Controller of HMSO 2015. This work was produced by Wright *et al.* under the terms of a commissioning contract issued by the Secretary of State for Health. This issue may be freely reproduced for the purposes of private research and study and extracts (or indeed, the full report) may be included in professional journals provided that suitable acknowledgement is made and the reproduction is not associated with any form of advertising. Applications for commercial reproduction should be addressed to: NIHR Journals Library, National Institute for Health Research, Evaluation, Trials and Studies Coordinating Centre, Alpha House, University of Southampton Science Park, Southampton SO16 7NS, UK.

TABLE 14 Study and intervention characteristics: participants without a disorganised classification or diagnosis of RAD (*continued*)

Author, year and country of publication	Participants: parents/children	Sample characteristics: risk factor	Intervention name and description	Intervention duration/intensity/delivery (including age of child at delivery of intervention)	Control group description	Attachment measure
Roggman *et al.* (2009)[205]<br><br>USA	$N = 201$ children (I and C *n* not reported)<br><br>Parent age: mean 22.84 years (SD 5.27 years), range 14–44 years<br><br>Child age: not reported<br><br>Ethnicity: 82% European American<br><br>SES: low-income families	Mothers and toddlers in poverty with associated risk factors such as teen mothers (30%), low education (24%) and single parents (28%)	Name: EHS<br><br>Aim: to improve cognitive skills and attachment security<br><br>Description: (i) parent-focused home visiting, (ii) socialisation groups<br><br>Video feedback: no<br><br>Male caregiver involved: unclear<br><br>Location: home	*Parent-focused home visiting*<br><br>Duration: 3 years<br><br>Intensity: weekly<br><br>Age of child at start: not reported<br><br>Delivered by: family educator<br><br>*Socialisation groups*<br><br>Details not reported	Not reported	AQS
Sajaniemi *et al.* (2001)[212]<br><br>Finland	$N = 115$ children (I, $n = 52$; C, $n = 52$)<br><br>Parent age: not reported<br><br>Child age: not reported<br><br>Ethnicity: not reported<br><br>SES: not reported	Extremely low-birthweight infants (birthweight < 1000 g)	Name: not reported<br><br>Aim: to promote normal sensorimotor development<br><br>Description: home occupational therapy sessions<br><br>Video feedback: no<br><br>Male caregiver involved: no<br><br>Location: home	Duration: 6 months<br><br>Intensity: weekly sessions, 60 minutes per session<br><br>Age of child at start: 6 months<br><br>Delivered by: occupational therapist	Clinic visits at ages 3, 6, 9, 12, 18 and 24 months (like the intervention group) but no home treatment	PAA (Cassidy and Marvin 1992[47])

Author, year and country of publication	Participants: parents/children	Sample characteristics: risk factor	Intervention name and description	Intervention duration/intensity/delivery (including age of child at delivery of intervention)	Control group description	Attachment measure
Spieker *et al.* (2012)[177]<br><br>USA	N = 210 children (I, n = 105; C, n = 105)<br><br>Parent age: I, mean 36.5 years (SD 10.9 years), range not reported; C, mean 36.5 years (SD 10.9 years), range not reported<br><br>Child age: I, mean 17.96 months (SD 4.97 months), range 10–24 months; C, mean 18.06 months (SD 4.49 months), range 10–24 months<br><br>Ethnicity: I, 51% white; C, 65% white<br><br>SES: not reported	Toddlers in Child Welfare	Name: PFR<br><br>Aim: to increase caregiver's awareness of behavioural cues and miscues<br><br>Description: instructions and activities from the PFR manual including video feedback and handouts<br><br>Video feedback: yes (five sessions)<br><br>Male caregiver involved: yes<br><br>Location: home	Duration: 10 weeks<br><br>Intensity: 10 sessions, 60–75 minutes per session<br><br>Age of child at start: not reported<br><br>Delivered by master's degree-prepared providers	Early Education Support	TAS-45

continued

© Queen's Printer and Controller of HMSO 2015. This work was produced by Wright *et al.* under the terms of a commissioning contract issued by the Secretary of State for Health. This issue may be freely reproduced for the purposes of private research and study and extracts (or indeed, the full report) may be included in professional journals provided that suitable acknowledgement is made and the reproduction is not associated with any form of advertising. Applications for commercial reproduction should be addressed to: NIHR Journals Library, National Institute for Health Research, Evaluation, Trials and Studies Coordinating Centre, Alpha House, University of Southampton Science Park, Southampton SO16 7NS, UK.

TABLE 14 Study and intervention characteristics: participants without a disorganised classification or diagnosis of RAD (*continued*)

Author, year and country of publication	Participants: parents/children	Sample characteristics: risk factor	Intervention name and description	Intervention duration/intensity/delivery (including age of child at delivery of intervention)	Control group description	Attachment measure
Toth *et al.* (2002)[184]<br><br>USA	$N = 112$ dyads<br><br>[Ia (PPP), $n = 31$; Ib (PHV), $n = 48$; C, $n = 33$]<br><br>Parent age: not reported<br><br>Child age: mean 48.18 months (SD 6.88 months), range not reported<br><br>Ethnicity: not reported<br><br>SES: not reported	Families with a documented history of physical, sexual or emotional maltreatment or neglect	Name: Ia, PPP; Ib, PHV<br><br>Aim: Ia, to improve parent–child relationship; Ib, to address parent skills training<br><br>Description: Ia, corrective emotional experience in the context of the relationship with the therapist; Ib, home visits, social support and cognitive–behavioural techniques<br><br>Video feedback: no<br><br>Male caregiver involved: no<br><br>Location: home/centre	*Ia*<br><br>Duration: not reported<br><br>Intensity: weekly 60-minute sessions<br><br>Age of child at start: not reported<br><br>Delivered by: masters level clinical therapist<br><br>*Ib*<br><br>Duration: not reported<br><br>Intensity: Weekly 60 minute sessions<br><br>Age of child at start: not reported<br><br>Delivered by: master's degree-level clinical therapist, at home	Individual psychotherapy over the treatment period for a variety of mental health concerns	Narrative story-stem task (MSSB, Bretherton *et al.* 1990;[215] ASCT, Bretherton *et al.* 1990)[215]

Author, year and country of publication	Participants: parents/children	Sample characteristics: risk factor	Intervention name and description	Intervention duration/intensity/delivery (including age of child at delivery of intervention)	Control group description	Attachment measure
van Doesum *et al.* (2008),[178] Kersten-Alvarez *et al.* (2010)[179]<br><br>the Netherlands	*N* = 85 mothers (I, *n* = 43; C, *n* = 42)<br><br>Parent age: I, mean 29.6 years (SD 3.8 years), range not reported; C, mean 30.4 years (SD 3.9 years), range not reported<br><br>Child age: mean 5.5 months (SD 3.1 months), range 1–12 months<br><br>Ethnicity: 81% Dutch (Caucasian)<br><br>SES: not reported	Mothers meeting DSM-IV criteria for major depressive episodes or dysthymia receiving outpatient treatment for depression, with an infant up to 12 months	Name: not reported<br><br>Aim: to enhance the mother's sensitivity<br><br>Description: home visiting video-feedback-based intervention. Modelling, cognitive restructuring, practical pedagogical support and baby massage were also used as part of the intervention<br><br>Video feedback: yes (8–10 sessions)<br><br>Male caregiver involved: yes<br><br>Location: home	Duration: 3–4 months<br><br>Intensity: 8–10 sessions, 69–90 minutes per session<br><br>Age of child at start: 5.5 months<br><br>Delivered by: prevention specialist	Three telephone calls offering support and practical parenting advice	AQS<br><br>ASCT (Bretherton *et al.* 1990)[215]

ASCT, Attachment Story Completion Task; ASI, Attachment Separation Individualisation; C, control; CBT, cognitive–behavioural therapy; EHS, Early Head Start; I, intervention; IPT, interpersonal psychotherapy; MA, Master of Arts; MTFC-P, Multidimensional Treatment Foster Care Program for pre-schoolers; N/A, not applicable; NICU, Neonatal Intensive Care Unit; PAD, Parent Attachment Diary; PFR, Promoting First Relationships; PHV, Psychoeducational Home Visitation; PPIP, Preventative Psychotherapeutic Intervention Programme; PPP, preschool parent psychotherapy; QUARQ, Quality of Attachment Relationships Questionnaire; RFTS, Right from the Start; WIC, Women, Infants, and Children; SPOKES, Supporting Parents on Kids' Education; TAS-45, Toddler Attachment Sort-45; VIPP, Video-feedback Intervention to promote Positive Parenting; VIPP-R, Video-feedback Intervention to promote Positive Parenting with a Representational focus.

© Queen's Printer and Controller of HMSO 2015. This work was produced by Wright *et al.* under the terms of a commissioning contract issued by the Secretary of State for Health. This issue may be freely reproduced for the purposes of private research and study and extracts (or indeed, the full report) may be included in professional journals provided that suitable acknowledgement is made and the reproduction is not associated with any form of advertising. Applications for commercial reproduction should be addressed to: NIHR Journals Library, National Institute for Health Research, Evaluation, Trials and Studies Coordinating Centre, Alpha House, University of Southampton Science Park, Southampton SO16 7NS, UK.

TABLE 15 Study and intervention characteristics: participants with a disorganised classification or diagnosis of RAD

Author, year and country of publication	Participants: parents/children	Sample characteristics	Intervention name and description	Intervention duration/intensity/delivery (including age of child at delivery of intervention)	Control group description	Measure of attachment
Bernard *et al.* (2012),[193] Dozier *et al.* (2009)[194]<br><br>USA	$N = 120$ children (I, $n = 60$; C, $n = 60$)<br><br>Parent age: mean 28.4 years (SD 7.8 years), range 15.7–47.0 years<br><br>Child age: mean 10.1 months (SD 6.0 months), range 1.7–21.4 months<br><br>Ethnicity: 61% African American<br><br>SES: not reported	Parents referred by agencies working with Child Protective Services. Young children who have experienced early adversity	Name: ABC<br><br>Aim: to enhance parent's sensitive nurturing care<br><br>Description: individual semistructured parent coaching using video feedback<br><br>Video feedback: yes (six sessions)<br><br>Male caregiver involved: yes<br><br>Location: home/shelters	Duration: 10 weeks<br><br>Intensity: 10 sessions, once a week<br><br>Age of child at start: not reported<br><br>Delivered by: professional social workers or psychologists	DEF: manualised home visitation intervention programme	SSP, PAD (Stovall-McClough and Dozier 2004[210])

Author, year and country of publication	Participants: parents/children	Sample characteristics	Intervention name and description	Intervention duration/intensity/delivery (including age of child at delivery of intervention)	Control group description	Measure of attachment
Cassidy *et al.* (2011)[196]<br><br>USA	$N = 220$ children (I, $n = 86$; C, $n = 88$)<br><br>Parent age: mean 24.06 years (SD 5.23 years), range 18–39 years<br><br>Child age: newborn<br><br>Ethnicity: 43.2% African American<br><br>SES: economically stressed	Economically stressed mothers and highly irritable infants	Name: COS-4 intervention<br><br>Aim: to enhance maternal sensitivity<br><br>Description: (i) home visits, (ii) video feedback<br><br>Video feedback: yes (four sessions)<br><br>Male caregiver involved: no<br><br>Location: home	*Home visits*<br><br>Duration 2.5 months<br><br>Intensity: three 1-hour home visits<br><br>Age of child at start: 6.5 months<br><br>Delivered by: master's degree- and doctoral-level clinicians<br><br>*Video feedback*<br><br>Duration: not reported<br><br>Intensity: one visit<br><br>Age of child at start: not reported<br><br>Delivered by master's degree- and doctoral-level clinicians	Three 1-hour psychoeducational home visit sessions, following the same timeline as the intervention and delivered by the same visitor	SSP
Cooper (2009)[189]<br><br>UK	$N = 449$ mothers (I, $n = 220$; C, $n = 229$)<br><br>Parent age: I, mean 25.5 years (SD 5.23 years), range not reported; C, mean 26.2 years (SD 5.8 years), range not reported<br><br>Ethnicity: not reported<br><br>SES: not reported	Mothers living in poverty and in an area of high unemployment, typical housing being shanty town or shack accommodation	Name: not reported<br><br>Aim: to enhance maternal sensitivity<br><br>Description: home visits providing support and guidance in parenting<br><br>Video feedback: no<br><br>Male caregiver involved: no<br><br>Location: home	Duration: 5 months<br><br>Intensity: 16 sessions<br><br>Age of child at start: prenatal<br><br>Delivered by: lay community workers	Fortnightly visits by community health worker	SSP

continued

© Queen's Printer and Controller of HMSO 2015. This work was produced by Wright *et al.* under the terms of a commissioning contract issued by the Secretary of State for Health. This issue may be freely reproduced for the purposes of private research and study and extracts (or indeed, the full report) may be included in professional journals provided that suitable acknowledgement is made and the reproduction is not associated with any form of advertising. Applications for commercial reproduction should be addressed to: NIHR Journals Library, National Institute for Health Research, Evaluation, Trials and Studies Coordinating Centre, Alpha House, University of Southampton Science Park, Southampton SO16 7NS, UK.

TABLE 15 Study and intervention characteristics: participants with a disorganised classification or diagnosis of RAD (*continued*)

Author, year and country of publication	Participants: parents/children	Sample characteristics	Intervention name and description	Intervention duration/intensity/delivery (including age of child at delivery of intervention)	Control group description	Measure of attachment
Heinicke *et al.* (2001),[190] Heinicke *et al.* (2000),[192] Heinicke *et al.* (1999)[191]<br><br>USA	$N = 70$ families (I, $n = 31$; C, $n = 33$)<br><br>Parent age: mean 24 years, SD and range not reported<br><br>Child age: prenatal<br><br>Ethnicity: 45.3% Latino<br><br>SES: 62.5% working class	Socially high-risk mothers; all mothers were poor and lacked support	Name: UCLA FDP intervention<br><br>Aim: to promote mother's sense of self-efficacy<br><br>Description: (i) home visits, (ii) mother–infant group, (iii) possible referral to community resources<br><br>Video feedback: no<br><br>Male caregiver involved: yes<br><br>Location: (i) home, (ii) unclear for mother–infant group	*Home visits*<br><br>Duration: from late pregnancy to 1 year old<br><br>Intensity: weekly, 60 minutes per visit<br><br>Age of child at start: prenatal<br><br>Delivered by: mental health professionals<br><br>*Mother–infant group*<br><br>Duration: 12 months<br><br>Intensity: weekly (number of weeks attended range 0–43 weeks)<br><br>Age of child at start: 3 months<br><br>Delivered by: mental health professionals	Care as usual from the paediatric continuity clinic	Child expects care (Bayley's Scale of Infant Development);[216] child's secure response to separation; child's positive affect; SSP; AQS

Author, year and country of publication	Participants: parents/children	Sample characteristics	Intervention name and description	Intervention duration/intensity/delivery (including age of child at delivery of intervention)	Control group description	Measure of attachment
Minnis *et al.* (2001),[187] Minnis (1999)[188]<br><br>UK	*N* = 160 families (I, *n* = 80; C, *n* = 80)<br><br>Parent age:<br><br>I, mean 45 years (SD 8.8 years), range not reported (mother); mean 46 years (SD 10 years), range not reported (father)<br><br>C, mean 46 years (SD 7.8 years), range not reported (mother); mean 48 years (SD 7.3 years), range not reported (father)<br><br>Child age: I, mean 10.9 years (SD 3.1 years), range 5–16 years; C, mean 11.6 years (SD 3.27 years), range 5–16 years<br><br>Ethnicity: 99% white<br><br>SES: not reported	Children in foster care and their foster carers	Name: not reported<br><br>Aim: to increase understanding of emotional communication skills<br><br>Description: (i) extra training sessions for foster parents, (ii) training sessions for children<br><br>Video feedback: no<br><br>Male caregiver involved: yes<br><br>Location: unclear	*Extra training sessions for foster parents*<br><br>Duration: a week and 2 days<br><br>Intensity: 3 days, 6 hours per day<br><br>Age of child at start: not reported<br><br>Delivered by: experienced social worker/trainer<br><br>*Training sessions for children*<br><br>Details not reported	Standard services, optional council training	RAD scale (Minnis 1999[188])

continued

© Queen's Printer and Controller of HMSO 2015. This work was produced by Wright *et al.* under the terms of a commissioning contract issued by the Secretary of State for Health. This issue may be freely reproduced for the purposes of private research and study and extracts (or indeed, the full report) may be included in professional journals provided that suitable acknowledgement is made and the reproduction is not associated with any form of advertising. Applications for commercial reproduction should be addressed to: NIHR Journals Library, National Institute for Health Research, Evaluation, Trials and Studies Coordinating Centre, Alpha House, University of Southampton Science Park, Southampton SO16 7NS, UK.

TABLE 15 Study and intervention characteristics: participants with a disorganised classification or diagnosis of RAD (*continued*)

Author, year and country of publication	Participants: parents/children	Sample characteristics	Intervention name and description	Intervention duration/intensity/delivery (including age of child at delivery of intervention)	Control group description	Measure of attachment
Moran *et al.* (2005)[218]<br><br>Canada	$N = 100$ dyads (I, $n =$ not reported; C, $n =$ not reported)<br><br>Parent age: mean 18.42 (range 15.97–19.98) years (SD 1.01 years)<br><br>Child age: not reported<br><br>Ethnicity: 81% Caucasian<br><br>SES: not reported	Adolescent mothers and their infants	Name: not reported<br><br>Aim: to enhance maternal sensitivity<br><br>Description: video feedback home visits<br><br>Video feedback: yes (eight sessions)<br><br>Male caregiver involved: no<br><br>Location: home	Duration: 5 months<br><br>Intensity: eight home visits, 1 hour long<br><br>Age of child at start: 6 months<br><br>Delivered by: two professionals experienced in infant attachment and attachment theory	One home visit	SSP
Moss *et al.* (2011)[195]<br><br>Canada	$N = 89$ mothers (I, $n = 43$; C, $n = 46$)<br><br>Parent age: mean 27.82 years (SD 7.61 years), range 18–49 years<br><br>Child age: mean 3.35 years (SD 1.38 years), range 1–5.9 years<br><br>Ethnicity: not reported<br><br>SES: not reported	Very high-risk sample, comparable to other maltreating samples. Families being monitored for child maltreatment	Name: not reported<br><br>Aim: to enhance maternal sensitivity<br><br>Description: (i) home visits, (ii) video feedback<br><br>Video feedback: yes (eight sessions)<br><br>Male caregiver involved: no<br><br>Location: home	Duration: not reported<br><br>Intensity: eight weekly sessions, 90 minutes per session<br><br>Age of child at start: mean 3.35 years (SD 1.38 years)<br><br>Delivered by: child welfare clinical workers	Standard agency services consisted of monthly visits by a child welfare caseworker	SSP for 12- to 24-month-old children. Preschool separation–reunion procedure (Cassidy, Marvin and the MacArthur Working Group on Attachment 1992[47]) was used to assess attachment in children aged 2–6 years

Author, year and country of publication	Participants: parents/children	Sample characteristics	Intervention name and description	Intervention duration/intensity/delivery (including age of child at delivery of intervention)	Control group description	Measure of attachment
Toth *et al.* (2006),[199] Cicchetti *et al.* (1999)[200]<br><br>USA	*N* = 130 families (I, *n* = 66; C, *n* = 64)<br><br>Parent age: mean 31.68 years (SD 4.68 years), range 21–41 years<br><br>Child age: mean 20.34 months (SD 2.5 months), range not reported<br><br>Ethnicity: 92.9% European American<br><br>SES: 72.7% in two highest levels of social status	Mothers with a history of major depressive disorder since birth of their child	Name: TPP<br><br>Aim: to optimise the quality of the mother–child relationship<br><br>Description: TPP with an intervention manual<br><br>Video feedback: no<br><br>Male caregiver involved: no<br><br>Location: unclear	Duration: mean 58.19 weeks (range 42–79 weeks) (SD 10 weeks)<br><br>Intensity: mean number of sessions 45.24 (SD 11.16 mean number of sessions), range 30–75 mean number of sessions<br><br>Age of child at start: not reported<br><br>Delivered by: therapist	Not reported	SSP; AQS (Waters *et al.* 1995);[126] Attachment Q-scales (Howes and Richie, 1999)[217]
van den Boom (1995),[197] van den Boom (1994)[198]<br><br>the Netherlands	*N* = 100 dyads (I, *n* = 50; C, *n* = 50)<br><br>Parent age: mean and SD not reported, range 19–33 years<br><br>Child age: not reported<br><br>Ethnicity: 100% Caucasian<br><br>SES: low	Mothers meeting DSM-IV criteria for major depressive episodes or dysthymia receiving outpatient treatment for depression, with an infant up to 12 months	Name: not reported<br><br>Aim: to improve responsiveness to infant cues<br><br>Description: home visits observing the interaction between child and mother<br><br>Video feedback: no<br><br>Male caregiver involved: no<br><br>Location: home	Duration: 3 months<br><br>Intensity: one session every 3 weeks, 2 hours per session<br><br>Age of child at start: 6 months<br><br>Delivered by: not reported	Care as usual	AQS, SSP

ABC, Attachment and Biobehavioural Catch-up; C, control; COS-4, Circle of security – home visiting-4; DEF, Developmental Education for Families; I, intervention; PAD, Parent Attachment Diary; TTP, toddler–parent psychotherapy; UCLA FDP, University of California, Los Angeles Family Development Project.

© Queen's Printer and Controller of HMSO 2015. This work was produced by Wright *et al.* under the terms of a commissioning contract issued by the Secretary of State for Health. This issue may be freely reproduced for the purposes of private research and study and extracts (or indeed, the full report) may be included in professional journals provided that suitable acknowledgement is made and the reproduction is not associated with any form of advertising. Applications for commercial reproduction should be addressed to: NIHR Journals Library, National Institute for Health Research, Evaluation, Trials and Studies Coordinating Centre, Alpha House, University of Southampton Science Park, Southampton SO16 7NS, UK.

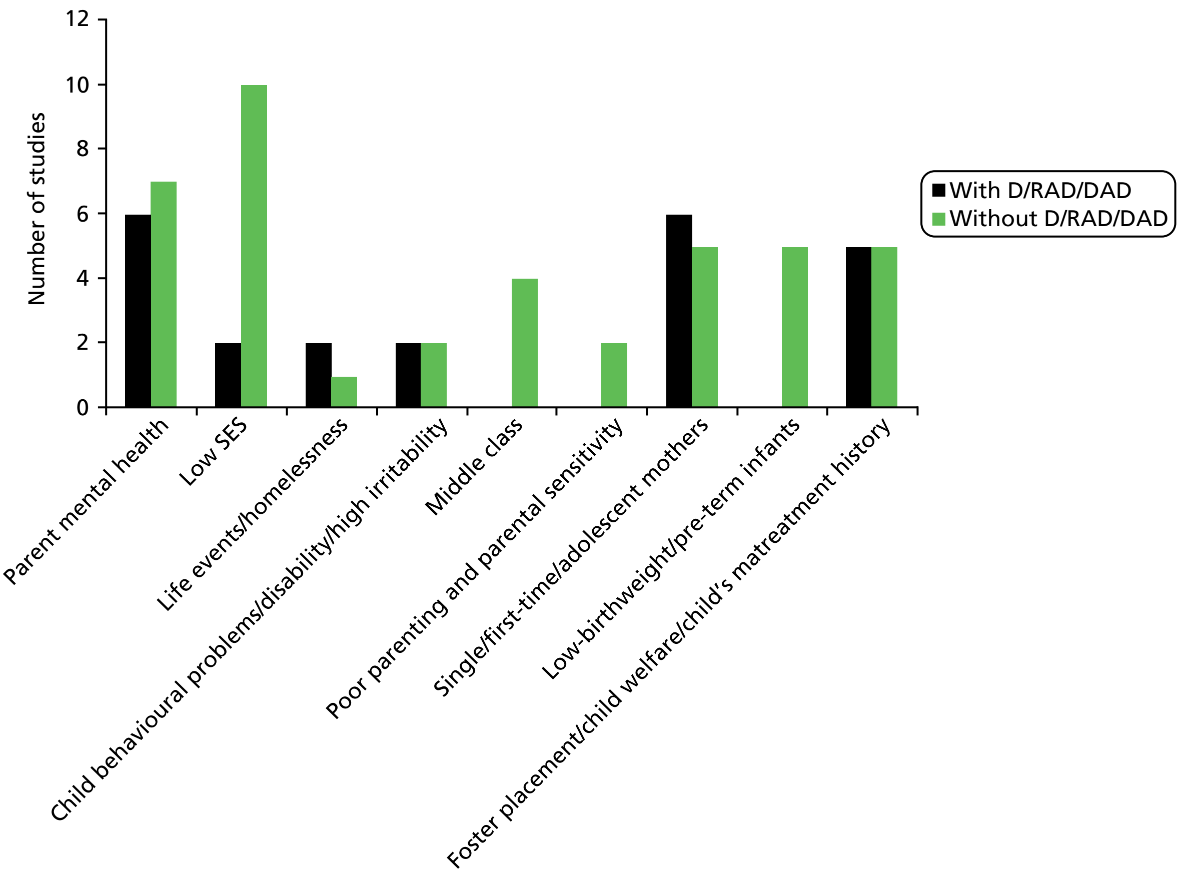

FIGURE 6 Study population characteristics for studies with and without a disorganised attachment pattern (D) or a diagnosis of RAD/DAD.

Specific interventions

The following are brief descriptions of some of the specific interventions included. Studies marked * concern participants with a disorganised pattern or a diagnosis of RAD/DAD.

Preventative Psychotherapeutic Intervention Program

Study Brisch and colleagues,[202] Germany.

Aim The Preventative Psychotherapeutic Intervention Program (PPIP) aims to improve parental sensitivity and enhance parents' capacity to recognise their infants' signalling with the aim of developing the child's secure attachment.

How PPIP includes a parent group on coping with premature birth; individual psychotherapy sessions for mothers and fathers separately that deals with experiences of loss and separation; a sensitivity training session; and, finally, a home visit.

University of California, Los Angeles Family Development Project intervention

Studies Heinicke and colleagues,[190] Heinicke and colleagues,[192] Heinicke and colleagues,[191] USA*; Beckwith,[201] USA.

Aim The aim of the University of California, Los Angeles Family Development Project (UCLA FDP) is to enhance the capacity of a family to support each other and to effectively recognise and meet the needs of their infant.

How Prenatal and postnatal health care is facilitated with weekly and biweekly home visits in the first 2 years, alongside developmental assessments and psychiatric services as required.

Circle of Security – home visiting-4 intervention

Study Cassidy and colleagues,[196] USA*.

Aim The Circle of Security – home visiting-4 (COS-4) is an early intervention programme designed to prevent insecure attachment and child mental health disorders.

How By instilling caregivers with awareness and understanding of the unconscious responses they have to their children. It seeks to teach caregivers to learn to regulate their cognitive, affective and behavioural responses to their infant.

Multidimensional Treatment Foster Care Program for Pre-schoolers

Study Fisher and Kim,[181] USA.

Aim The Multidimensional Treatment Foster Care Program for Pre-schoolers (MTFC-P) aims to encourage prosocial behaviour, non-abusive limit setting and close supervision of the child by the foster parent, with the intention of improving the attachment security of the child.

How MTFC-P provides foster parents with training and ongoing consultations and support from a team of staff. Children also received training and attend a therapeutic playgroup for approximately 9–12 months.

Promoting First Relationships programme

Study Spieker and colleagues,[177] USA*.

Aim Promoting First Relationships is a training programme dedicated to promoting children's social and emotional development through responsive, nurturing caregiver–child relationships.

How Providers work with caregivers to promote a healthy relationship between caregivers and their children. The programme of intervention includes videotaping interactions to provide insight into the relationship, and providing the caregiver with positive feedback to improve their competence and confidence with their child. The intervention also focuses on the deeper emotional feelings underlying caregivers' and children's behaviours.

Video-feedback Intervention to promote Positive Parenting

Studies Kalinauskiene and colleagues,[183] Lithuania; Klein-Velderman and colleagues,[203] Klein-Velderman, and colleagues[204] the Netherlands.

[See *Video-feedback Intervention to promote Positive Parenting with a Representational focus (VIPP-R)*.]

Video-feedback Intervention to promote Positive Parenting with a Representational focus

Studies Kalinauskiene and colleagues,[183] Lithuania; Klein-Velderman and colleagues,[203] Klein-Velderman and colleagues,[204] the Netherlands.

Aim The Video-feedback Intervention to promote Positive Parenting with a Representational focus (VIPP-R) intervention allows the caregiver to focus on the infant's signals and expression caught on tape, thereby improving observational skills with regards to the child. It also allows for positive reinforcement of sensitive behaviour shown by the caregiver on tape.

How Caregiver and infant are videotaped during daily situations at their home, for example playing together or at bath time. The tape is reviewed by the intervenor, who prepares comments for the next visit. During the next visit the videotape is reviewed with the parent, focusing on the positive interactions. VIPP-R adds a representational focus for the parent.

© Queen's Printer and Controller of HMSO 2015. This work was produced by Wright *et al.* under the terms of a commissioning contract issued by the Secretary of State for Health. This issue may be freely reproduced for the purposes of private research and study and extracts (or indeed, the full report) may be included in professional journals provided that suitable acknowledgement is made and the reproduction is not associated with any form of advertising. Applications for commercial reproduction should be addressed to: NIHR Journals Library, National Institute for Health Research, Evaluation, Trials and Studies Coordinating Centre, Alpha House, University of Southampton Science Park, Southampton SO16 7NS, UK.

Right from the Start

Study Niccols,[175] Canada.

Aim Right from the Start is a 'coping modelling problem solving approach' and was designed to enhance parental sensitivity, thereby improving the child's security.

How Large groups of parents (12–40) sit at tables in smaller groups and watch video clips of parents making exaggerated errors in common caregiver–child interaction situations. They discuss in their small groups the errors and the impact of the errors, as well as alternatives and the benefits of the alternatives. Large-group discussion follows each small-group discussion. The caregivers have opportunities to practise their skills at home and this is discussed in following sessions.

Toddler–Parent Psychotherapy

Studies Toth and colleagues,[199] Cicchetti and colleagues,[200] USA*.

Aim Toddler–Parent Psychotherapy (TPP) aims to explore how the parent perceives the child and help correct any distorted perceptions, supporting positive changes in behaviour towards the toddler.

How During TPP, mothers and infants are seen in dyadic conjoint therapy sessions. These sessions present an opportunity to observe the influence of the maternal representations on the interaction with the child. Through highlighting, clarifying and restructuring the dynamic balance between representational and interactional contributions to the quality of the mother–child relationship, improvement in the quality of maternal and child relationship capacities emerges.

Preschool parent psychotherapy

Study Toth and colleagues,[184] USA.

Aim Preschool parent psychotherapy is designed to provide the mother with a corrective emotional experience in the context of the relationship with the therapist.

How During the 60-minute dyadic sessions, the therapist uses empathy, respect, concern and positive regard to overcome the maltreating mother's negative expectations. The sessions seek to help the mother positively reconstruct representations of herself in relation to her child.

Psychoeducational Home Visitation

Study Toth and colleagues,[184] USA.

Aim The initial goal of the Psychoeducational Home Visitation (PHV) intervention is to conduct an assessment of the risk within the families and the circumstances of maltreatment, and then to focus on the provision of parent education regarding the development of the child, in addition to developing the parent's own self-care skills.

How Once risk and protective factors have been identified, the therapist attempts to implement change working with the mother–child dyad, using social support, psychoeducational strategies and cognitive behavioural techniques.

Early Head Start home-based programme

Study Roggman and colleagues,[205] USA.

Aim The aim of the Early Head Start (EHS) intervention is to foster positive parent–child interactions, to enhance parents' understanding of their children's development, to encourage parents to engage in activities with their children that promote development and to help families access needed services in the community.

How The programme was designed to provide child and family development services in weekly home visits and socialisation groups for parents and children. Parents in the EHS home-based programme are guided in reading their infants' cues, responding to their physical and emotional needs and enjoying playful interactions with them.

The Home Visiting Program

Study Ammaniti and colleagues,[206] Italy.

Aim The intervention aims to stimulate the mother–infant interaction and, in addition, works to support the marital interaction.

How During a home visit, the caregivers are encouraged to improve their sensitivity towards their child, observe their interactions with their baby and realise the importance of their influence on the child's development. Home visiting aims to enhance the parent's capacity to read and interpret the signals and behaviours of the child.

Attachment and Biobehavioural Catch-up (ABC)

Studies Bernard and colleagues,[193] Dozier and colleagues,[194] USA.

Aim The Attachment and Biobehavioural Catch-up (ABC) intervention is designed to assist families with children who have experienced early maltreatment or disruption in care.

How The programme is delivered in 10 manualised sessions with parents in the home. ABC helps caregivers to interpret their child's behavioural signals and provide more nurturing care where it does not come naturally. The intervention also helps caregivers to provide a responsive, predictable and appropriate environment that enhances the child's capabilities.

Fostering Changes Programme

Study Briskman and Scott,[207] UK.

Aim The intervention focuses on teaching foster carers new skills which can be used at home with their foster child. Understanding the antecedents of behaviour helps carers to know why specific patterns of behaviour arise in certain contexts, and helps them to recognise and avoid the psychological or environmental triggers.

How Each session begins with feedback from carers about using their newly acquired skills, and the introduction of a new topic, for example information about psychological and physiological influences on behaviour. At the end of each session carers are given the opportunity to feed back on their experience of the group, including any concerns they might have.

Interpersonal psychotherapy

Study Forman and colleagues, USA.[182]

Aim The main aim of interpersonal psychotherapy (IPT) in this context is to address problems that are interpersonal in nature, including interpersonal conflicts (particularly with the spouse and intimate others), and to understand social role transitions such as the transition to motherhood, as well as loss and grief.

How Although this can be used for any adult with depression, in the study by Forman[182] treatment began when children were approximately 6 months old and lasted for 12 weeks.

Counselling

Studies Murray and colleagues,[208] Cooper and colleagues,[209] UK.

Aim To offer support regarding concerns about being a new mother, focusing explicitly on the mother–infant relationship.

© Queen's Printer and Controller of HMSO 2015. This work was produced by Wright *et al.* under the terms of a commissioning contract issued by the Secretary of State for Health. This issue may be freely reproduced for the purposes of private research and study and extracts (or indeed, the full report) may be included in professional journals provided that suitable acknowledgement is made and the reproduction is not associated with any form of advertising. Applications for commercial reproduction should be addressed to: NIHR Journals Library, National Institute for Health Research, Evaluation, Trials and Studies Coordinating Centre, Alpha House, University of Southampton Science Park, Southampton SO16 7NS, UK.

How Non-directive, one-to-one counselling, in which women are provided with the opportunity to air their feelings about any current concerns, such as marital problems or financial difficulties, as well as concerns they might have about their infant. Therapy is conducted in the women's own homes on a weekly basis from 8 weeks to 18 weeks post-partum.

Cognitive–behavioural therapy

Studies Murray and colleagues,[208] Cooper and colleagues,[209] UK.

Aim As above (see *Counselling*), but in this instance the treatment is primarily directed not at the maternal depression itself, but at problems identified by the parent in the management of her infant (concerning, for example, feeding or sleeping), as well as observed problems in the quality of the mother–infant interaction.

How In the context of a supportive therapeutic relationship, the parent is provided with advice about managing particular infant problems, is encouraged to examine her patterns of thinking about her infant and herself as a mother (e.g. challenging negative thinking) and is helped through modelling and reinforcement to alter aspects of her interactions with her child.

Brief psychodynamic psychotherapy

Studies Murray and colleagues,[208] Cooper and colleagues,[209] UK.

Aim To explore the parent's representation of her infant and her relationship with her infant, to promote positive representations and coping.

How One-to-one psychodynamic therapy, using the treatment techniques to explore aspects of the mother's own early history to promote her representation of her infant.

Incredible Years parent programme

Study O'Connor and colleagues,[176] UK.

Aim The main aim of the Incredible Years programme is the treatment of child aggressive behaviour problems and attention deficit hyperactivity disorder, and the prevention of conduct problems, delinquency, violence and drug abuse.

How The intervention works towards improving parent–child interactions, building positive parent–child relationships and attachment, improving parental functioning and facilitating less harsh and more nurturing parenting. The intervention also attempts to increase parental social support and improve teacher classroom management skills and teacher–parent partnerships.

Interventions which were not included

There were a number of studies that we were aware of that we might have expected to see in a review of this nature. Some of these parental interventions for attachment disorders, or interventions intended to improve attachment security, have not been uncovered by this review but were mentioned by the PPI or expert groups, and are described below. It should be noted that this is not an exhaustive list.

Organisational or policy interventions

Our criteria excluded any studies that were not focused on interventions at the caregiver/parental level. Interventions at an organisational level, including, for example, adoption as an intervention,[156] and studies such as the Bucharest Early Intervention Project[98,219] and the English Romanian Adoptee study,[220] were therefore not included in this review.

Theraplay

Theraplay is an intervention used in several child mental health services around the UK.[221] We were unable to find any evidence that met the criteria of our systematic review.

Dyadic developmental psychotherapy

Dyadic developmental psychotherapy is a relationship-focused intervention that seeks to develop and sustain a contingent collaborative and affectively attuned relationship between therapist and child, between caregiver and child and between therapist and caregiver.[222] This intervention was not included in our review as no studies met the PICOS criteria for the main systematic review (see *Appendix 5*).

Watch, Wait and Wonder training

Watch, Wait and Wonder training is based on the notion of the infant negotiating the infant–parent relationship within the psychotherapy session. Most of the work in the intervention is between the mother and therapist. For half the session, the mother gets down on the floor with the infant, observes and interacts only on the infant's initiative. The idea is that this increases the mother's sensitivity and responsiveness as a result of her taking an observational viewpoint, while also being physically accessible. For the second half, the mother discusses her observations and experiences with the therapist.[223] This intervention did not make it into our review as no studies met the criteria for the main systematic review (see *Appendix 5*).

It should be noted that the non-inclusion of any intervention is not a comment on the intervention itself, but on the presence of available evidence for this systematic review.

Quality assessment

Table 16 shows the results of the Cochrane quality assessment for included intervention studies without a disorganised category or a diagnosis of RAD. *Table 17* shows the results of the Cochrane quality assessment for included intervention studies that contained a disorganised category or diagnosis of RAD. Three domains were consistently rated as high bias across the included studies. These were incomplete outcome data, selective reporting and 'other' bias. Incomplete outcome reporting was often rated as high bias because attrition was over 10% across the course of the trial. The reason for the selective reporting item predominantly receiving a rating of high bias across the studies was poor reporting of the secondary outcomes within the studies. Many of the studies received a rating of high in 'other bias concerns'. There were various reasons for this including unexplained attrition, unexplained missing data, small sample size/low power and inconsistencies within the data.

Unclear reporting, where the author's descriptions were not sufficient to rate the relevant information, was apparent, with a lack of detail about the random sequence and the method of allocation concealment. Blinding was conducted to some extent in approximately 60% of all trials. The mixed presentation of trial quality across the review suggests that any conclusions should be interpreted with caution.

Of the 34 interventions identified in this phase, 21 were established, named interventions. Many of these consisted of multifaceted treatment programmes with components such as home visits, video feedback, family therapy or sensitivity training for caregivers. In some cases, several studies assessed the same intervention programme, such as the UCLA FDP intervention, which was evaluated by both Heinicke and colleagues[190–192] and Beckwith.[201]

Of the included interventions, very few involved only the caregiver and not the child. The IPT intervention is one such example that used therapy sessions targeted at external problems regarding the caregiver.[182] The majority of studies included both caregiver and child in the intervention, particularly interventions that involved sensitivity training, video feedback or dyadic play sessions.[183,196,202–204]

Alongside these named interventions, several unnamed interventions are identified in this phase. Some of these therapies involved the caregiver's physical proximity to the child, such as massage therapy[185] or utilising baby carriers.[180] Others used similar techniques to the named interventions; for example, many involved home visits from experienced mothers,[129,133] volunteer coaches[186] or other professionals.

The named and unnamed interventions have several overlapping themes in terms of their content, using similar techniques within the intervention strategies. Some of the more common themes or foci of the

© Queen's Printer and Controller of HMSO 2015. This work was produced by Wright *et al.* under the terms of a commissioning contract issued by the Secretary of State for Health. This issue may be freely reproduced for the purposes of private research and study and extracts (or indeed, the full report) may be included in professional journals provided that suitable acknowledgement is made and the reproduction is not associated with any form of advertising. Applications for commercial reproduction should be addressed to: NIHR Journals Library, National Institute for Health Research, Evaluation, Trials and Studies Coordinating Centre, Alpha House, University of Southampton Science Park, Southampton SO16 7NS, UK.

TABLE 16 Cochrane quality assessment checklist for studies without a disorganised category or a diagnosis of RAD/DAD

Author, year and country of publication	Random sequence (rating high, low, unclear)	Allocation concealment (rating high, low, unclear)	Blinding performance (rating high, low, unclear)	Incomplete outcome (rating high, low, unclear)	Selective reporting (rating high, low, unclear)	Free of other bias (rating high, low, unclear)
O'Connor *et al.* (2012)[176]<br>UK	Low	Low	Low	Unclear	Low	High
Niccols (2008)[175]<br>Canada	Low	Unclear	Low	High	High	Low
Spieker *et al.* (2012)[177]<br>USA	Low	Unclear	Low	High	Low	High
Van Doesum *et al.* (2008);[178] Kersten-Alvarez *et al.* (2010)[179]<br>the Netherlands	Low	Unclear	Low	High	Unclear	Low
Anisfeld *et al.* (1990)[180]<br>USA	Unclear	Unclear	Low	High	High	High
Fisher and Kim (2007)[181]<br>USA	Unclear	Unclear	Low	High	Low	High
Forman *et al.* (2007)[182]<br>USA	Low	Unclear	Low	High	High	High
Kalinauskiene (2009)[183]<br>Lithuania	Unclear	Unclear	Unclear	Low	Unclear	Low
Toth *et al.* (2002)[184]<br>USA	Unclear	Unclear	Low	High	High	Low
Barnett *et al.* (1987);[129] Barnett and Parker (1985)[133]<br>Australia	Unclear	Unclear	Low	High	Unclear	High
Hansen and Ulrey (1988)[185]<br>USA	Unclear	Unclear	Low	High	High	High
Jacobson and Frye (1991)[186]<br>USA	Unclear	Unclear	Low	High	Unclear	High
Klein-Velderman *et al.* (2006);[203] Klein-Velderman *et al.* (2006)[204]<br>the Netherlands	Unclear	Unclear	Unclear	Low	High	High

TABLE 16 Cochrane quality assessment checklist for studies without a disorganised category or a diagnosis of RAD/DAD (*continued*)

Author, year and country of publication	Random sequence (rating high, low, unclear)	Allocation concealment (rating high, low, unclear)	Blinding performance (rating high, low, unclear)	Incomplete outcome (rating high, low, unclear)	Selective reporting (rating high, low, unclear)	Free of other bias (rating high, low, unclear)
Lieberman *et al.* (1991)[211]<br><br>USA	Unclear	Unclear	Low	High	Unclear	Unclear
Murray *et al.* (2003);[208] Cooper *et al.* (2003)[209]<br><br>UK	Low	Unclear	Unclear	High	Unclear	High
Sajaniemi *et al.* (2001)[212]<br><br>Finland	Low	Unclear	Unclear	High	Unclear	High
Ammaniti *et al.* (2006)[206]<br><br>Italy	Unclear	Unclear	Low	High	High	High
Beckwith (1988)[201]<br><br>USA	Unclear	Unclear	Unclear	High	High	High
Brisch *et al.* (2003)[202]<br><br>Germany	Unclear	Unclear	High	High	High	High
Briskman and Scott (2012)[207]<br><br>UK	Unclear	Unclear	Unclear	High	High	High
Roggman *et al.* (2009)[205]<br><br>USA	Unclear	Unclear	Unclear	High	High	High

© Queen's Printer and Controller of HMSO 2015. This work was produced by Wright *et al.* under the terms of a commissioning contract issued by the Secretary of State for Health. This issue may be freely reproduced for the purposes of private research and study and extracts (or indeed, the full report) may be included in professional journals provided that suitable acknowledgement is made and the reproduction is not associated with any form of advertising. Applications for commercial reproduction should be addressed to: NIHR Journals Library, National Institute for Health Research, Evaluation, Trials and Studies Coordinating Centre, Alpha House, University of Southampton Science Park, Southampton SO16 7NS, UK.

TABLE 17 Cochrane quality assessment table for studies that contained a disorganised category or a diagnosis of RAD/DAD

Author, year and country of publication	Random sequence (rating high, low, unclear)	Allocation concealment (rating high, low, unclear)	Blinding performance (rating high, low, unclear)	Incomplete outcome (rating high, low, unclear)	Selective reporting (rating high, low, unclear)	Free of other bias (rating high, low, unclear)
Minnis *et al.* (2001);[187] Minnis (1999)[188]<br><br>UK	Low	Low	Low	High	Low	Low
Cooper *et al.* (2009)[189]<br><br>UK	Low	Low	Low	High	Low	Low
Bernard *et al.* (2012);[193] Dozier *et al.* (2009)[194]<br><br>USA	Unclear	Unclear	Low	Low	Low	Low
Heinicke *et al.* (2001);[190] Heinicke *et al.* (1999);[191] Heinicke *et al.* (2000)[192]<br><br>USA	Low	Low	Low	Unclear	Unclear	High
Moran *et al.* (2005)[218]<br><br>Canada	Unclear	Unclear	Unclear	Low	Low	Low
Moss *et al.* (2011)[195]<br><br>Canada	Low	Unclear	Low	High	Unclear	High
Toth *et al.* (2006);[199] Cicchetti *et al.* (1999)[200]<br><br>USA	Low	Unclear	Low	High	High	High
Cassidy *et al.* (2011)[196]<br><br>USA	Unclear	Unclear	Low	High	Unclear	High
van den Boom (1995);[197] van den Boom (1994)[198]<br><br>the Netherlands	Unclear	Unclear	Low	Unclear	High	High

interventions included using positive feedback for the caregiver, exploring and changing the parent's perception of the child, improving parental attachment to the infant and promoting sensitive caregiving.

Meta-analyses findings

Of the 39 papers (30 studies[129,175–178,180–186,188,189,191,194–196,198,200–203,205–208,211,212,218]) that were included in the main systematic review (see *Figure 5*), 21 papers had data that could be included in the meta-analysis.[129,133,180,189–200,202–204,208,209,218] The remaining 18 papers could not be included because they did not contain sufficient raw data to be analysed. We initially set out to do three meta-analyses.

i. Only one RCT identified had investigated the effectiveness of an intervention on RAD symptoms;[190,191] therefore, we were unable to conduct a meta-analysis for the effectiveness of interventions on attachment disorders.
ii. We investigated the effectiveness of the intervention in reducing disorganised patterns of attachment.
iii. We investigated the effectiveness of the interventions in promoting a secure attachment as an outcome. This has been included in *Appendix 6* to provide additional information.

Disorganised attachment

Eight studies reported on interventions that attempted to reduce disorganised attachment in at-risk groups and, by corollary, increase organised attachments.[189,191,194–196,198,200,218] Disorganised attachment is the attachment pattern most associated with subsequent child psychopathology and subsequent adult psychopathology, and is therefore of great importance to this review. All of these studies were also included in the studies promoting a secure attachment (see *Appendix 6*).

Figure 7 presents the included study on a funnel plot. The distribution of the studies on the plot is roughly symmetrical, indicating that publication bias is not likely to be present.

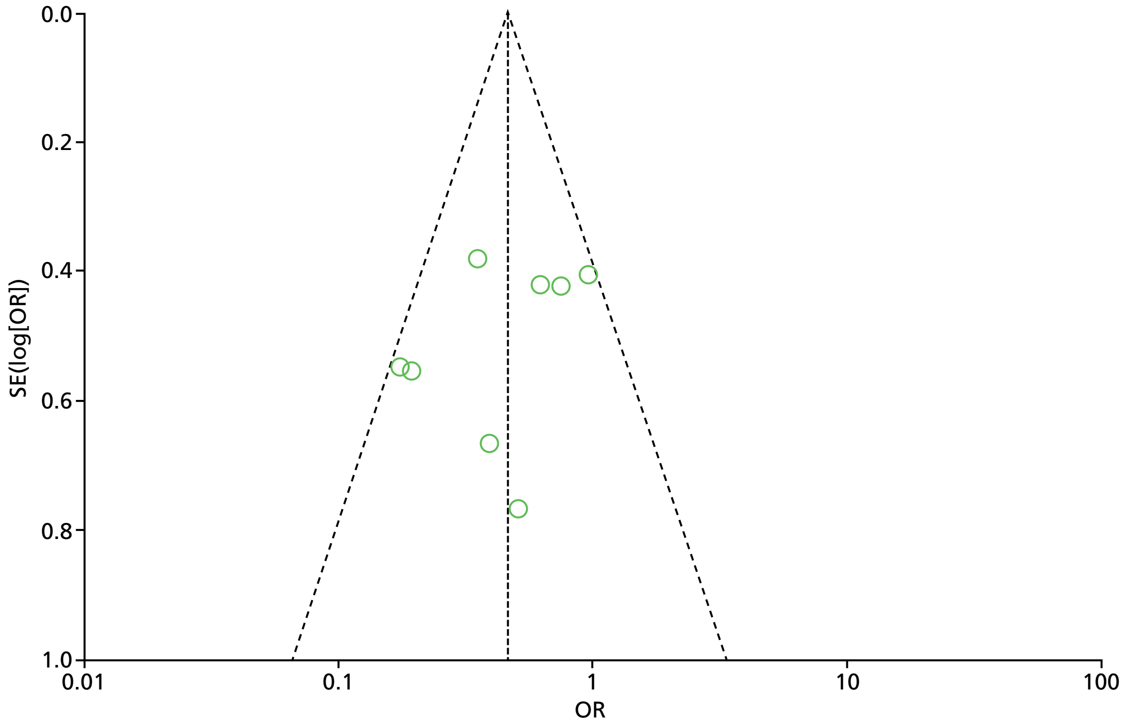

FIGURE 7 Funnel plot of papers including a disorganised classification or a diagnosis of RAD. SE, standard error.

© Queen's Printer and Controller of HMSO 2015. This work was produced by Wright *et al.* under the terms of a commissioning contract issued by the Secretary of State for Health. This issue may be freely reproduced for the purposes of private research and study and extracts (or indeed, the full report) may be included in professional journals provided that suitable acknowledgement is made and the reproduction is not associated with any form of advertising. Applications for commercial reproduction should be addressed to: NIHR Journals Library, National Institute for Health Research, Evaluation, Trials and Studies Coordinating Centre, Alpha House, University of Southampton Science Park, Southampton SO16 7NS, UK.

Overall, a meta-analysis of the included eight studies shows a very significant benefit from treatment (*Figure 8*). The intervention saw less disorganised attachment at outcome than the control (OR 0.47, 95% CI 0.34 to 0.65; $p < 0.00001$).

We conducted a number of subgroup analyses to compare the mean effect for different subgroups of studies. *Figure 9* displays the results of the analysis comparing intervention duration as measure in months. Only two studies[191,200] had an intervention lasting more than 12 months (13 months).

Figure 10 displays the results of the meta-analysis comparing the length of time taken to conduct follow-up assessments. Only one study had data on follow-up > 12 months.[189]

There is some evidence for a differential effect when examining the duration of the intervention as measured by the number of sessions (*Figure 11*). There is no evidence for a statistical significance for interventions of fewer than five sessions, but only two studies were included.[196–198] By contrast, the groups including 5–16 sessions[189,193–195,218] and more than 16 sessions[190,191,199,200] are both statistically significant. As there are no direct comparisons randomising between short and long interventions in terms of number of sessions, no definitive conclusions can be drawn from this.

Figure 12 displays the results of the meta-analysis comparing the age of the child at the start of the intervention. There are only a small number of studies in each of the three subgroups (two to three author groups in each). Interventions in children identified as at risk who receive interventions after 6 months of age show some promising findings. However, there are only two studies each in the prenatal[189–191] and 0- to 6-month[197,198,218] intervention groups.

Figure 13 displays the results of the meta-analysis comparing caregivers with and without foster children. Only two papers (from one study)[193,194] involved children who were in foster care.

Studies delivered at home show statistically significant improvement on meta-analysis (*Figure 14*). Only one study is included in which the intervention was not carried out exclusively at home, and therefore no comparison can be made.[190,191]

Figure 15 displays the results of the meta-analysis comparing studies that included a male caregiver in the intervention and those that did not. Two trials involved a male carer,[190,191,193,194] but the other six studies did not include a male carer in the intervention alongside the female caregiver.[189,195,196,199,200,218] Effect sizes were statistically significant in both groups.

Figure 16 displays the results of the meta-analysis comparing studies that provided video feedback in the intervention and those that did not. There are several studies in each of these groups. Both achieved statistical significance.

Most studies used an intervention that had, as one of its main elements, the enhancement of maternal sensitivity (*Figure 17*). Only one study involving 64 children was not focused around this. The Heinicke and colleagues[190,191] intervention intention was focused around improving adult self-esteem and self-efficacy as opposed to improving maternal sensitivity, but only had a small study group. The effect size for studies enhancing maternal sensitivity was highly statistically significant.

Some interventions involved the caregiver and child together, whereas others involved some sessions, in addition to the dyadic ones, that were just for the caregiver[193,194] (*Figure 18*). Most studies focus on therapy with the caregiver and child together.

Study or subgroup	Intervention Events	Intervention Total	Control Events	Control Total	Weight	OR M–H, fixed, 95% CI
Cassidy 2011[196]	12	85	15	84	11.9%	0.76 (0.33 to 1.73)
Cicchetti 1999,[200] Toth 2006[199]	5	46	22	54	16.6%	0.18 (0.06 to 0.52)
Cooper 2009[189]	10	156	16	162	13.5%	0.63 (0.27 to 1.42)
Dozier 2009,[194] Bernard 2012[193]	19	60	34	60	21.4%	0.35 (0.17 to 0.75)
Heinicke 1999,[191] 2001[190]	4	31	9	33	7.0%	0.40 (0.11 to 1.45)
Moran 2005[218]	28	49	29	50	11.3%	0.97 (0.44 to 2.14)
Moss 2011[195]	7	35	18	32	13.8%	0.19 (0.07 to 0.57)
van den Boom 1994,[198] 1995[197]	3	43	4	39	3.6%	0.66 (0.14 to 3.14)
**Total (95% CI)**		**505**		**514**	**100.0%**	**0.47 (0.34 to 0.65)**
Total events	88		148			

Heterogeneity: $\chi^2 = 11.36$, df = 7 ($p = 0.12$); $I^2 = 38\%$
Test for overall effect: $z = 4.53$ ($p < 0.00001$)

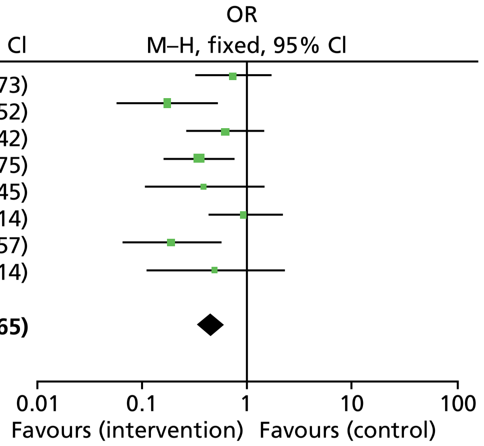

FIGURE 8 A meta-analysis of changes in disorganised outcomes, comparing parenting intervention and a control condition. df, degrees of freedom.

© Queen's Printer and Controller of HMSO 2015. This work was produced by Wright *et al.* under the terms of a commissioning contract issued by the Secretary of State for Health. This issue may be freely reproduced for the purposes of private research and study and extracts (or indeed, the full report) may be included in professional journals provided that suitable acknowledgement is made and the reproduction is not associated with any form of advertising. Applications for commercial reproduction should be addressed to: NIHR Journals Library, National Institute for Health Research, Evaluation, Trials and Studies Coordinating Centre, Alpha House, University of Southampton Science Park, Southampton SO16 7NS, UK.

Study or subgroup	Intervention Events	Intervention Total	Control Events	Control Total	Weight	OR M–H, fixed, 95% CI
**<6 months**						
Cassidy 2011[196]	12	85	15	84	12.0%	0.76 (0.33 to 1.73)
Cooper 2009[189]	10	156	16	162	13.6%	0.63 (0.27 to 1.42)
Dozier 2009,[194] Bernard 2012[193]	19	60	34	60	21.6%	0.35 (0.17 to 0.75)
Moran 2005[218]	28	49	29	50	11.4%	0.97 (0.44 to 2.14)
Moss 2011[195]	7	35	18	32	14.0%	0.19 (0.07 to 0.57)
van den Boom 1994[198]	3	43	4	39	3.6%	0.66 (0.14 to 3.14)
**Subtotal (95% CI)**		**428**		**427**	**76.2%**	**0.54 (0.38 to 0.78)**
Total events	79		116			
Heterogeneity: $\chi^2=7.49$, df=5 ($p=0.19$); $I^2=33\%$						
Test for overall effect: $z=3.35$ ($p=0.0008$)						
**>12 months**						
Cicchetti 1999,[200] Toth 2006[199]	5	46	22	54	16.7%	0.18 (0.06 to 0.52)
Heinicke 1999,[191] 2001[190]	4	31	9	33	7.0%	0.40 (0.11 to 1.45)
**Subtotal (95% CI)**		**77**		**87**	**23.8%**	**0.24 (0.11 to 0.55)**
Total events	9		31			
Heterogeneity: $\chi^2=0.87$, df=1 ($p=0.35$); $I^2=0\%$						
Test for overall effect: $z=3.39$ ($p=0.0007$)						
**Total (95% CI)**		**505**		**514**	**100.0%**	**0.47 (0.34 to 0.65)**
Total events	88		147			
Heterogeneity: $\chi^2=11.36$, df=7 ($p=0.12$); $I^2=38\%$						
Test for overall effect: $z=4.53$ ($p<0.00001$)						
Test for subgroup differences: $\chi^2=3.14$, df=1 ($p=0.08$); $I^2=68.1\%$						

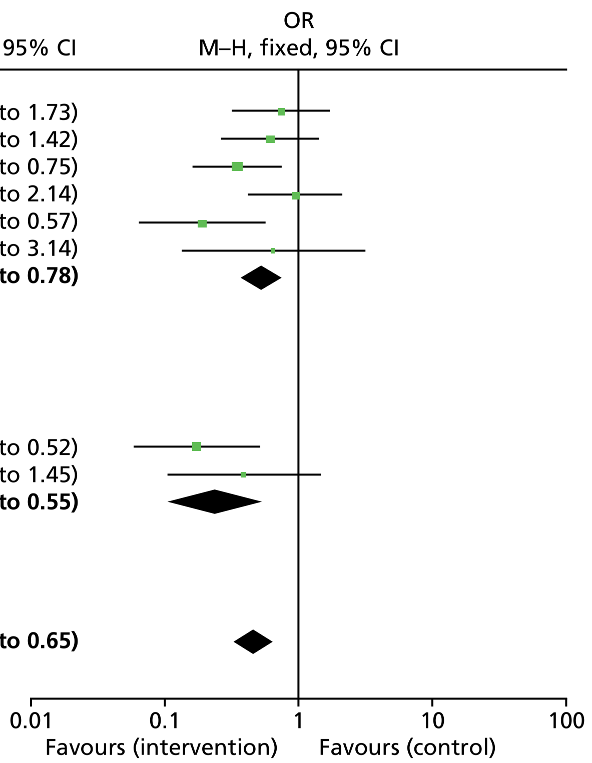

FIGURE 9 Changes in disorganised outcomes, comparing duration of intervention. df, degrees of freedom.

Study or subgroup	Intervention Events	Intervention Total	Control Events	Control Total	Weight	OR M–H, fixed, 95% CI
**<12 months**						
Dozier 2009,[194] Bernard 2012[193]	19	60	34	60	42.9%	0.35 (0.17 to 0.75)
Moran 2005[218]	28	49	29	50	22.7%	0.97 (0.44 to 2.14)
van den Boom 1994[198]	3	43	4	39	7.2%	0.66 (0.14 to 3.14)
**Subtotal (95% CI)**		**152**		**149**	**72.9%**	**0.57 (0.35 to 0.95)**
Total events	50		67			
Heterogeneity: $\chi^2=3.27$, df=2 ($p=0.20$); $I^2=39\%$						
Test for overall effect: $z=2.14$ ($p=0.03$)						
**>12 months**						
Cooper 2009[189]	10	156	16	162	27.1%	0.63 (0.27 to 1.42)
**Subtotal (95% CI)**		**156**		**162**	**27.1%**	**0.63 (0.27 to 1.42)**
Total events	10		16			
Heterogeneity: not applicable						
Test for overall effect: $z=1.12$ ($p=0.26$)						
**Total (95% CI)**		**308**		**311**	**100.0%**	**0.59 (0.38 to 0.91)**
Total events	60		83			
Heterogeneity: $\chi^2=3.30$, df=3 ($p=0.35$); $I^2=9\%$						
Test for overall effect: $z=2.41$ ($p=0.02$)						
Test for subgroup differences: $\chi^2=0.03$, df=1 ($p=0.87$); $I^2=0\%$						

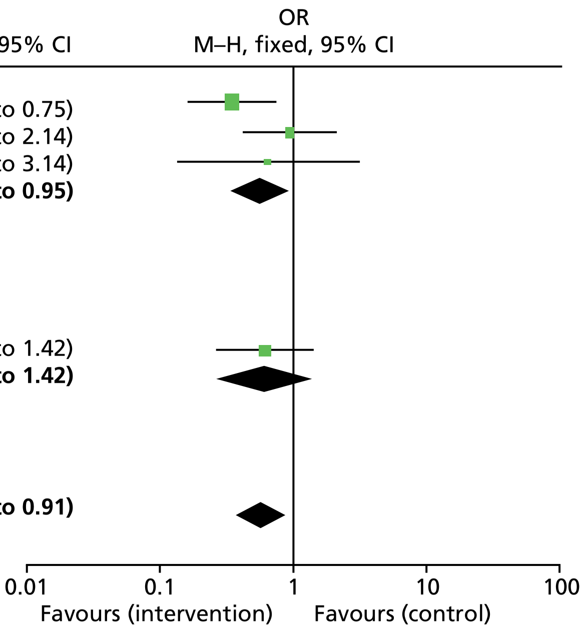

FIGURE 10 Changes to disorganised outcomes, comparing length of follow-up (< 12 months/≥ 12 months). df, degrees of freedom.

© Queen's Printer and Controller of HMSO 2015. This work was produced by Wright *et al.* under the terms of a commissioning contract issued by the Secretary of State for Health. This issue may be freely reproduced for the purposes of private research and study and extracts (or indeed, the full report) may be included in professional journals provided that suitable acknowledgement is made and the reproduction is not associated with any form of advertising. Applications for commercial reproduction should be addressed to: NIHR Journals Library, National Institute for Health Research, Evaluation, Trials and Studies Coordinating Centre, Alpha House, University of Southampton Science Park, Southampton SO16 7NS, UK.

Study or subgroup	Intervention Events	Intervention Total	Control Events	Control Total	Weight	OR M–H, fixed, 95% CI
**<5 sessions**						
Cassidy 2011[196]	12	85	15	84	12.0%	0.76 (0.33 to 1.73)
van den Boom 1994[198]	3	43	4	39	3.6%	0.66 (0.14 to 3.14)
**Subtotal (95% CI)**		**128**		**123**	**15.6%**	**0.73 (0.35 to 1.52)**
Total events	15		19			
Heterogeneity: $\chi^2=0.02$, df=1 ($p=0.88$); $I^2=0\%$						
Test for overall effect: $z=0.83$ ($p=0.41$)						
**5–15 sessions**						
Cooper 2009[189]	10	156	16	162	13.6%	0.63 (0.27 to 1.42)
Dozier 2009,[194] Bernard 2012[193]	19	60	34	60	21.6%	0.35 (0.17 to 0.75)
Moran 2005[218]	28	49	29	50	11.4%	0.97 (0.44 to 2.14)
Moss 2011[195]	7	35	18	32	14.0%	0.19 (0.07 to 0.57)
**Subtotal (95% CI)**		**300**		**304**	**60.6%**	**0.49 (0.33 to 0.74)**
Total events	64		97			
Heterogeneity: $\chi^2=6.64$, df=3 ($p=0.08$); $I^2=55\%$						
Test for overall effect: $z=3.36$ ($p=0.0008$)						
**>15 sessions**						
Cicchetti 1999,[200] Toth 2006[199]	5	46	22	54	16.7%	0.18 (0.06 to 0.52)
Heinicke 1999,[191] 2001[190]	4	31	9	33	7.0%	0.40 (0.11 to 1.45)
**Subtotal (95% CI)**		**77**		**87**	**23.8%**	**0.24 (0.11 to 0.55)**
Total events	9		31			
Heterogeneity: $\chi^2=0.87$, df=1 ($p=0.35$); $I^2=0\%$						
Test for overall effect: $z=3.39$ ($p=0.0007$)						
**Total (95% CI)**		**505**		**514**	**100.0%**	**0.47 (0.34 to 0.65)**
Total events	88		147			
Heterogeneity: $\chi^2=11.36$, df=7 ($p=0.12$); $I^2=38\%$						
Test for overall effect: $z=4.53$ ($p<0.00001$)						
Test for subgroup differences: $\chi^2=3.99$, df=2 ($p=0.14$); $I^2=49.9\%$						

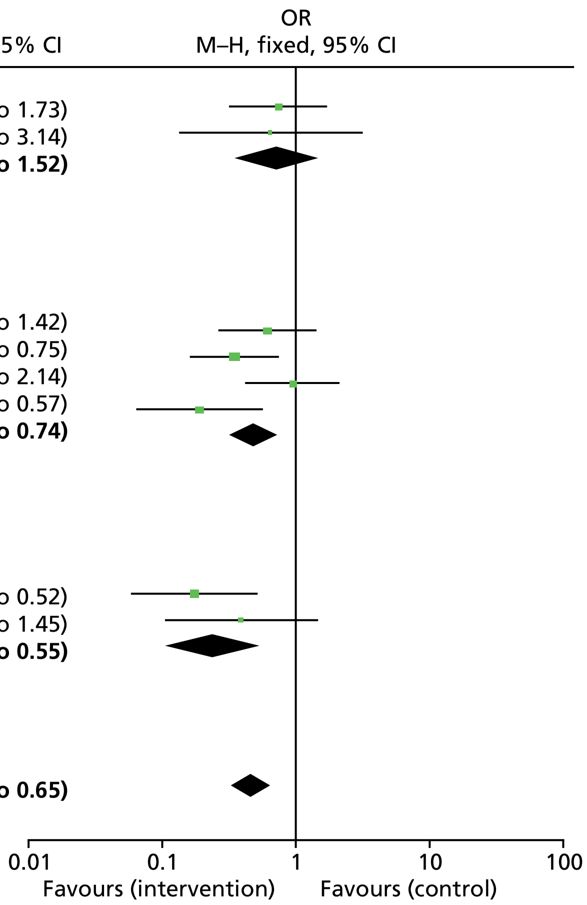

FIGURE 11 A meta-analysis of changes in disorganised outcomes, comparing number of sessions (< 5, 5–16, > 16). df, degrees of freedom.

Study or subgroup	Intervention Events	Intervention Total	Control Events	Control Total	Weight	OR M–H, fixed, 95% CI
**Prenatal**						
Cooper 2009[189]	10	156	16	162	17.4%	0.63 (0.27 to 1.42)
Heinicke 1999,[191] 2001[190]	4	31	9	33	9.0%	0.40 (0.11 to 1.45)
**Subtotal (95% CI)**		**187**		**195**	**26.4%**	**0.55 (0.27 to 1.09)**
Total events	14		25			
Heterogeneity: $\chi^2=0.34$, df $=1$ ($p=0.56$); $I^2=0\%$						
Test for overall effect: $z=1.71$ ($p=0.09$)						
**≤6 months**						
Moran 2005[218]	28	49	29	50	14.6%	0.97 (0.44 to 2.14)
van den Boom 1994[198]	3	43	4	39	4.6%	0.66 (0.14 to 3.14)
**Subtotal (95% CI)**		**92**		**89**	**19.2%**	**0.89 (0.44 to 1.81)**
Total events	31		33			
Heterogeneity: $\chi^2=0.19$, df $=1$ ($p=0.67$); $I^2=0\%$						
Test for overall effect: $z=0.32$ ($p=0.75$)						
**>6 months**						
Cassidy 2011[196]	12	85	15	84	15.3%	0.76 (0.33 to 1.73)
Cicchetti 1999,[200] Toth 2006[199]	5	46	22	54	21.3%	0.18 (0.06 to 0.52)
Moss 2011[195]	7	35	18	32	17.8%	0.19 (0.07 to 0.57)
**Subtotal (95% CI)**		**166**		**170**	**54.5%**	**0.35 (0.20 to 0.60)**
Total events	24		55			
Heterogeneity: $\chi^2=6.00$, df $=2$ ($p=0.05$); $I^2=67\%$						
Test for overall effect: $z=3.83$ ($p=0.0001$)						
**Total (95% CI)**		**445**		**454**	**100.0%**	**0.50 (0.35 to 0.72)**
Total events	69		113			
Heterogeneity: $\chi^2=10.58$, df $=6$ ($p=0.10$); $I^2=43\%$						
Test for overall effect: $z=3.72$ ($p<0.0002$)						
Test for subgroup differences: $\chi^2=4.38$, df $=2$ ($p=0.11$); $I^2=54.4\%$						

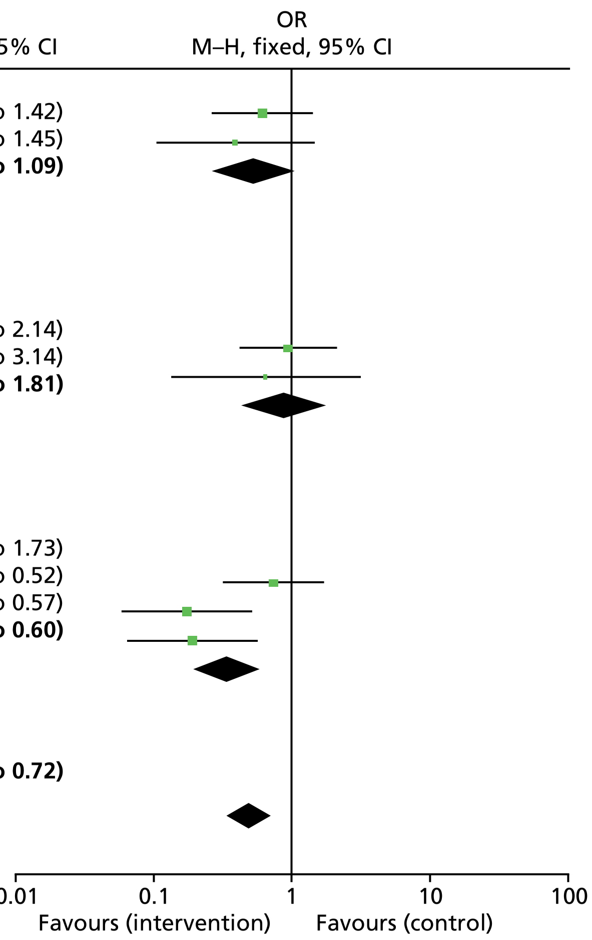

FIGURE 12 A meta-analysis of changes to disorganised outcomes, comparing age of child at start (prenatal/≤6 months old/>6 months old). df, degrees of freedom.

© Queen's Printer and Controller of HMSO 2015. This work was produced by Wright *et al.* under the terms of a commissioning contract issued by the Secretary of State for Health. This issue may be freely reproduced for the purposes of private research and study and extracts (or indeed, the full report) may be included in professional journals provided that suitable acknowledgement is made and the reproduction is not associated with any form of advertising. Applications for commercial reproduction should be addressed to: NIHR Journals Library, National Institute for Health Research, Evaluation, Trials and Studies Coordinating Centre, Alpha House, University of Southampton Science Park, Southampton SO16 7NS, UK.

Study or subgroup	Intervention Events	Intervention Total	Control Events	Control Total	Weight	OR M–H, fixed, 95% CI
**With foster**						
Dozier 2009,[194] Bernard 2012[193]	19	60	34	60	21.6%	0.35 (0.17 to 0.75)
**Subtotal (95% CI)**		**60**		**60**	**21.6%**	**0.35 (0.17 to 0.75)**
Total events	19		34			
Heterogeneity: not applicable						
Test for overall effect: $z=2.73$ ($p=0.006$)						
**Without foster**						
Cassidy 2011[196]	12	85	15	84	12.0%	0.76 (0.33 to 1.73)
Cicchetti 1999,[200] Toth 2006[199]	5	46	22	54	16.7%	0.18 (0.06 to 0.52)
Cooper 2009[189]	10	156	16	162	13.6%	0.63 (0.27 to 1.42)
Heinicke 1999,[191] 2001[190]	4	31	9	33	7.0%	0.40 (0.11 to 1.45)
Moran 2005[218]	28	49	29	50	11.4%	0.97 (0.44 to 2.14)
Moss 2011[195]	7	35	18	32	14.0%	0.19 (0.07 to 0.57)
van den Boom 1994,[198] 1995[197]	3	43	4	39	3.6%	0.66 (0.14 to 3.14)
**Subtotal (95% CI)**		**445**		**454**	**78.4%**	**0.50 (0.35 to 0.72)**
Total events	69		113			
Heterogeneity: $\chi^2=10.58$, df=6 ($p=0.10$); $I^2=43\%$						
Test for overall effect: $z=3.72$ ($p=0.0002$)						
**Total (95% CI)**		**505**		**514**	**100.0%**	**0.47 (0.34 to 0.65)**
Total events	88		147			
Heterogeneity: $\chi^2=11.36$, df=7 ($p=0.12$); $I^2=38\%$						
Test for overall effect: $z=4.53$ ($p<0.00001$)						
Test for subgroup differences: $\chi^2=0.69$, df=1 ($p=0.41$); $I^2=0\%$						

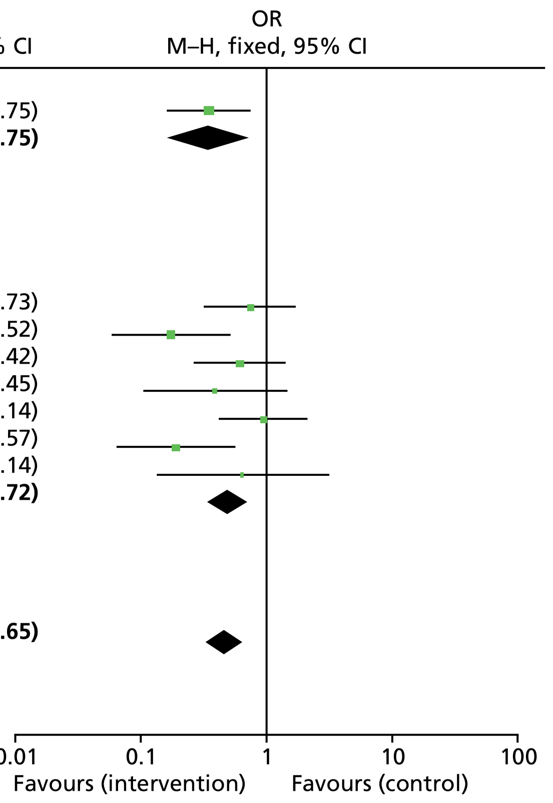

FIGURE 13 A meta-analysis of changes in disorganised outcomes, comparing caregivers with and without foster children. df, degrees of freedom.

Study or subgroup	Intervention Events	Intervention Total	Control Events	Control Total	Weight	OR M–H, fixed, 95% CI
**Home**						
Cassidy 2011[196]	12	85	15	84	14.4%	0.76 (0.33 to 1.73)
Cooper 2009[189]	10	156	16	162	16.4%	0.63 (0.27 to 1.42)
Dozier 2009,[194] Bernard 2012[193]	19	60	34	60	25.9%	0.35 (0.17 to 0.75)
Moran 2005[218]	28	49	29	50	13.7%	0.97 (0.44 to 2.14)
Moss 2011[195]	7	35	18	32	16.8%	0.19 (0.07 to 0.57)
van den Boom 1994,[198] 1995[197]	3	43	4	39	4.3%	0.66 (0.14 to 3.14)
**Subtotal (95% CI)**		**428**		**427**	**91.5%**	**0.54 (0.38 to 0.78)**
Total events	79		116			
Heterogeneity: $\chi^2=7.49$, df=5 ($p=0.19$); $I^2=33\%$						
Test for overall effect: $z=3.35$ ($p=0.0008$)						
**Mixed**						
Heinicke 1999,[191] 2001[190]	4	31	9	33	8.5%	0.40 (0.11 to 1.45)
**Subtotal (95% CI)**		**31**		**33**	**8.5%**	**0.40 (0.11 to 1.45)**
Total events	4		9			
Heterogeneity: not applicable						
Test for overall effect: $z=1.40$ ($p=0.16$)						
**Total (95% CI)**		**459**		**460**	**100.0%**	**0.53 (0.38 to 0.75)**
Total events	83		125			
Heterogeneity: $\chi^2=7.71$, df=6 ($p=0.26$); $I^2=22\%$						
Test for overall effect: $z=3.61$ ($p=0.0003$)						
Test for subgroup differences: $\chi^2=0.21$, df=1 ($p=0.64$); $I^2=0\%$						

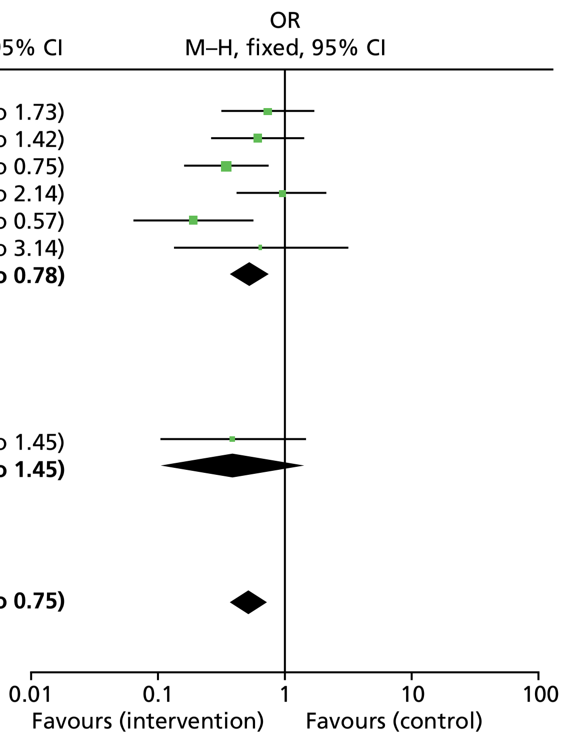

FIGURE 14 A meta-analysis of disorganised outcomes, comparing intervention locations (home, mixed, other). df, degrees of freedom.

© Queen's Printer and Controller of HMSO 2015. This work was produced by Wright *et al.* under the terms of a commissioning contract issued by the Secretary of State for Health. This issue may be freely reproduced for the purposes of private research and study and extracts (or indeed, the full report) may be included in professional journals provided that suitable acknowledgement is made and the reproduction is not associated with any form of advertising. Applications for commercial reproduction should be addressed to: NIHR Journals Library, National Institute for Health Research, Evaluation, Trials and Studies Coordinating Centre, Alpha House, University of Southampton Science Park, Southampton SO16 7NS, UK.

Study or subgroup	Intervention Events	Intervention Total	Control Events	Control Total	Weight	OR M–H, fixed, 95% CI	OR M–H, fixed, 95% CI
**Included male caregiver**							
Dozier 2009,[194] Bernard 2012[193]	19	60	34	60	21.6%	0.35 (0.17 to 0.75)	
Heinicke 1999,[191] 2001[190]	4	31	9	33	7.0%	0.40 (0.11 to 1.45)	
**Subtotal (95% CI)**		**91**		**93**	**28.6%**	**0.36 (0.19 to 0.70)**	
Total events	23		43				
Heterogeneity: $\chi^2=0.02$, df = 1 ($p=0.89$); $I^2=0\%$							
Test for overall effect: $z=3.06$ ($p=0.002$)							
**Did not include male caregiver**							
Cassidy 2011[196]	12	85	15	84	12.0%	0.76 (0.33 to 1.73)	
Cicchetti 1999,[200] Toth 2006[199]	5	46	22	54	16.7%	0.18 (0.06 to 0.52)	
Cooper 2009[189]	10	156	16	162	13.6%	0.63 (0.27 to 1.42)	
Moran 2005[218]	28	49	29	50	11.4%	0.97 (0.44 to 2.14)	
Moss 2011[195]	7	35	18	32	14.0%	0.19 (0.07 to 0.57)	
van den Boom 1994,[198] 1995[197]	3	43	4	39	3.6%	0.66 (0.14 to 3.14)	
**Subtotal (95% CI)**		**414**		**421**	**71.4%**	**0.51 (0.35 to 0.75)**	
Total events	65		104				
Heterogeneity: $\chi^2=10.40$, df = 5 ($p=0.06$); $I^2=52\%$							
Test for overall effect: $z=3.46$ ($p=0.0005$)							
**Total (95% CI)**		**505**		**514**	**100.0%**	**0.47 (0.34 to 0.65)**	
Total events	88		147				
Heterogeneity: $\chi^2=11.36$, df = 7 ($p=0.12$); $I^2=38\%$							
Test for overall effect: $z=4.53$ ($p<0.00001$)							
Test for subgroup differences: $\chi^2=0.81$, df = 1 ($p=0.37$); $I^2=0\%$							

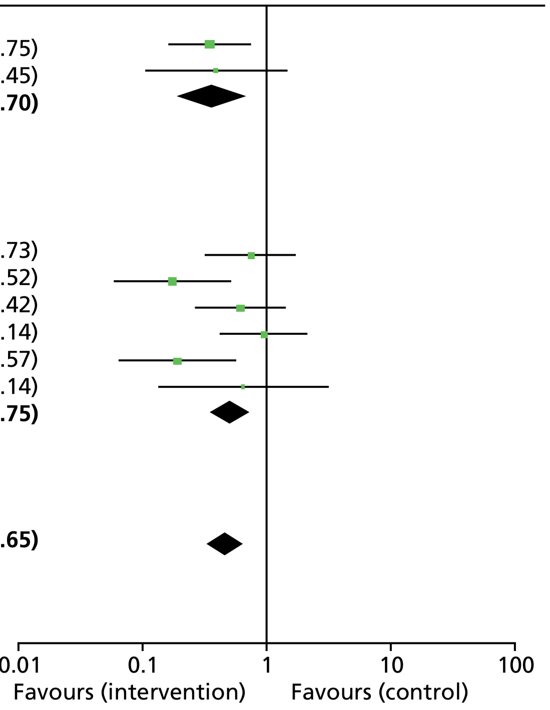

FIGURE 15 A meta-analysis of changes in disorganised attachment patterns, comparing inclusion of male caregiver alongside female caregiver with exclusion of male caregiver. df, degrees of freedom.

Study or subgroup	Intervention Events	Intervention Total	Control Events	Control Total	Weight	OR M–H, fixed, 95% CI
**Used video feedback**						
Cassidy 2011[196]	12	85	15	84	12.0%	0.76 (0.33 to 1.73)
Dozier 2009,[194] Bernard 2012[193]	19	60	34	60	21.6%	0.35 (0.17 to 0.75)
Moran 2005[218]	28	49	29	50	11.4%	0.97 (0.44 to 2.14)
Moss 2011[195]	7	35	18	32	14.0%	0.19 (0.07 to 0.57)
**Subtotal (95% CI)**		**229**		**226**	**59.0%**	**0.52 (0.34 to 0.78)**
Total events	66		96			
Heterogeneity: $\chi^2=7.28$, df=3 ($p=0.06$); $I^2=59\%$						
Test for overall effect: $z=3.15$ ($p=0.002$)						
**Did not use video feedback**						
Cicchetti 1999,[200] Toth 2006[199]	5	46	22	54	16.7%	0.18 (0.06 to 0.52)
Cooper 2009[189]	10	156	16	162	13.6%	0.63 (0.27 to 1.42)
Heinicke 1999,[191] 2001[190]	4	31	9	33	7.0%	0.40 (0.11 to 1.45)
van den Boom 1994,[198] 1995[197]	3	43	4	39	3.6%	0.66 (0.14 to 3.14)
**Subtotal (95% CI)**		**276**		**288**	**41.0%**	**0.41 (0.24 to 0.69)**
Total events	22		51			
Heterogeneity: $\chi^2=3.70$, df=3 ($p=0.30$); $I^2=19\%$						
Test for overall effect: $z=3.30$ ($p=0.0010$)						
**Total (95% CI)**		**505**		**514**	**100.0%**	**0.47 (0.34 to 0.65)**
Total events	88		147			
Heterogeneity: $\chi^2=11.36$, df=7 ($p=0.12$); $I^2=38\%$						
Test for overall effect: $z=4.53$ ($p<0.00001$)						
Test for subgroup differences: $\chi^2=0.49$, df=1 ($p=0.48$); $I^2=0\%$						

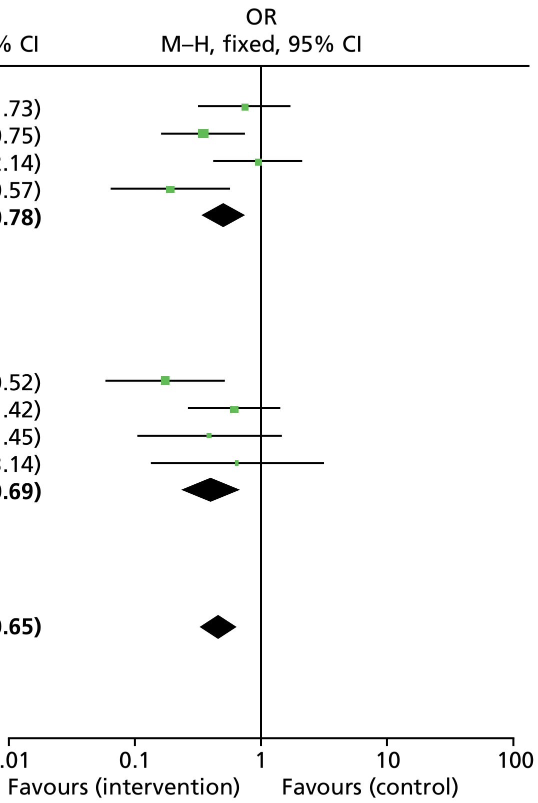

FIGURE 16 A meta-analysis of changes to disorganised outcomes, comparing interventions that provided video feedback with those that did not. df, degrees of freedom.

© Queen's Printer and Controller of HMSO 2015. This work was produced by Wright *et al.* under the terms of a commissioning contract issued by the Secretary of State for Health. This issue may be freely reproduced for the purposes of private research and study and extracts (or indeed, the full report) may be included in professional journals provided that suitable acknowledgement is made and the reproduction is not associated with any form of advertising. Applications for commercial reproduction should be addressed to: NIHR Journals Library, National Institute for Health Research, Evaluation, Trials and Studies Coordinating Centre, Alpha House, University of Southampton Science Park, Southampton SO16 7NS, UK.

Study or subgroup	Intervention Events	Intervention Total	Control Events	Control Total	Weight	OR M–H, fixed, 95% CI
**Yes**						
Cassidy 2011[196]	12	85	15	84	12.0%	0.76 (0.33 to 1.73)
Cicchetti 1999,[200] Toth 2006[199]	5	46	22	54	16.7%	0.18 (0.06 to 0.52)
Cooper 2009[189]	10	156	16	162	13.6%	0.63 (0.27 to 1.42)
Dozier 2009,[194] Bernard 2012[193]	19	60	34	60	21.6%	0.35 (0.17 to 0.75)
Moran 2005[218]	28	49	29	50	11.4%	0.97 (0.44 to 2.14)
Moss 2011[195]	7	35	18	32	14.0%	0.19 (0.07 to 0.57)
van den Boom 1994,[198] 1995[197]	3	43	4	39	3.6%	0.66 (0.14 to 3.14)
**Subtotal (95% CI)**		**474**		**481**	**93.0%**	**0.48 (0.34 to 0.67)**
Total events	84		138			
Heterogeneity: $\chi^2=11.27$, df=6 ($p=0.08$); $I^2=47\%$						
Test for overall effect: $z=4.32$ ($p<0.0001$)						
**No**						
Heinicke 1999,[191] 2001[190]	4	31	9	33	7.0%	0.40 (0.11 to 1.45)
**Subtotal (95% CI)**		**31**		**33**	**7.0%**	**0.40 (0.11 to 1.45)**
Total events	4		9			
Heterogeneity: not applicable						
Test for overall effect: $z=1.40$ ($p=0.16$)						
**Total (95% CI)**		**505**		**514**	**100.0%**	**0.47 (0.34 to 0.65)**
Total events	88		147			
Heterogeneity: $\chi^2=11.36$, df=7 ($p=0.12$); $I^2=38\%$						
Test for overall effect: $z=4.53$ ($p<0.00001$)						
Test for subgroup differences: $\chi^2=0.08$, df=1 ($p=0.78$); $I^2=0\%$						

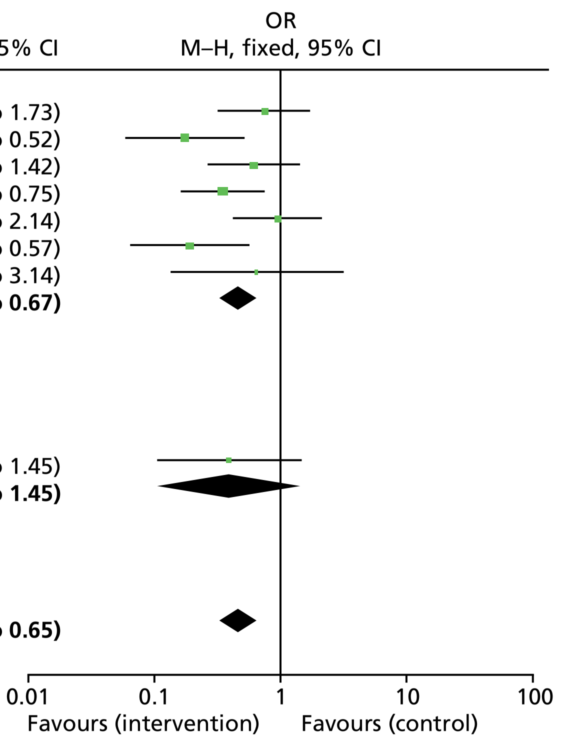

FIGURE 17 A meta-analysis of changes to disorganised outcomes, looking at whether or not the intervention attempts to enhance maternal sensitivity. df, degrees of freedom.

Study or subgroup	Intervention Events	Intervention Total	Control Events	Control Total	Weight	OR M–H, fixed, 95% CI
**Caregiver and child**						
Cassidy 2011[196]	12	85	15	84	13.9%	0.76 (0.33 to 1.73)
Cicchetti 1999,[200] Toth 2006[199]	5	46	22	54	19.4%	0.18 (0.06 to 0.52)
Heinicke 1999,[191] 2001[190]	4	31	9	33	8.2%	0.40 (0.11 to 1.45)
Moran 2005[218]	28	49	29	50	13.2%	0.97 (0.44 to 2.14)
Moss 2011[195]	7	35	18	32	16.2%	0.19 (0.07 to 0.57)
van den Boom 1994,[198] 1995[197]	3	43	4	39	4.2%	0.66 (0.14 to 3.14)
**Subtotal (95% CI)**		**289**		**292**	**75.0%**	**0.48 (0.32 to 0.71)**
Total events	59		97			
Heterogeneity: $\chi^2=10.32$, df = 5 ($p=0.07$); $I^2=52\%$						
Test for overall effect: $z=3.60$ ($p=0.0003$)						
**Mixed**						
Dozier 2009,[194] Bernard 2012[193]	19	60	34	60	25.0%	0.35 (0.17 to 0.75)
**Subtotal (95% CI)**		**60**		**60**	**25.0%**	**0.35 (0.17 to 0.75)**
Total events	19		34			
Heterogeneity: not applicable						
Test for overall effect: $z=2.73$ ($p=0.06$)						
**Total (95% CI)**		**349**		**352**	**100.0%**	**0.45 (0.31 to 0.64)**
Total events	78		131			
Heterogeneity: $\chi^2=10.88$, df = 6 ($p=0.09$); $I^2=45\%$						
Test for overall effect: $z=4.45$ ($p<0.00001$)						
Test for subgroup differences: $\chi^2=0.48$, df = 1 ($p=0.49$); $I^2=0\%$						

OR M–H, fixed, 95% CI

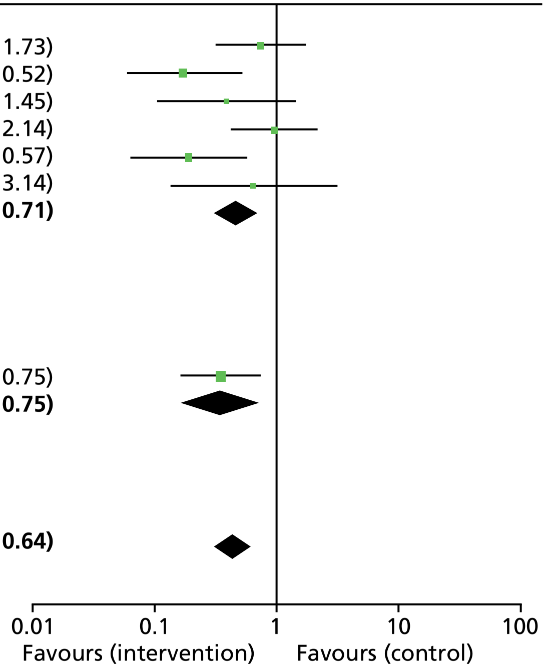

FIGURE 18 A meta-analysis of changes to disorganised outcomes, comparing types of intervention involved (caregiver and child together, caregiver and child separately, caregiver alone or mixed). df, degrees of freedom.

© Queen's Printer and Controller of HMSO 2015. This work was produced by Wright *et al.* under the terms of a commissioning contract issued by the Secretary of State for Health. This issue may be freely reproduced for the purposes of private research and study and extracts (or indeed, the full report) may be included in professional journals provided that suitable acknowledgement is made and the reproduction is not associated with any form of advertising. Applications for commercial reproduction should be addressed to: NIHR Journals Library, National Institute for Health Research, Evaluation, Trials and Studies Coordinating Centre, Alpha House, University of Southampton Science Park, Southampton SO16 7NS, UK.

Discussion of meta-analyses findings of parenting interventions improving disorganised attachment

There was no evidence of publication bias for the papers included in this study.

Disorganised attachment does not change when the number of sessions is between 0 and 4. By contrast, it does change for numbers of sessions above this. The effect size is no greater when session numbers are > 16 than when they are between 5 and 16. It is unlikely that much can be drawn from this, given the small numbers of studies exploring session numbers between 0 and 4 and > 16. It is promising that statistically significant findings emerge for studies of > 4 sessions, particularly in the 5- to 16-session group. This finding informs how resources in busy services can be best utilised to benefit families without long-term therapy and where there might be cost-effectiveness benefits. This remains to be explored further.

The largest effect size was in the group in which the child was > 6 months old at the start of the intervention. This may be because of the small number of studies under investigation. Different effect sizes may be related to selection bias in the subgroups being studied. This could be related to the fact that disorganised attachment often presents in children in institutional care or maltreated children,[218] and such very high-risk children are much less likely to be identified either prenatally or within the first 6 months of life. Moreover, it is not possible to measure disorganised attachment below the age of 6 months, or indeed 9 months, although studies were included in which disorganised attachment was measured at outcome. It is reassuring that interventions can work after 6 months of age, given that many interventions have been focusing on improving parental sensitivity.

It is notable that interventions carried out at home which reported on reducing disorganised attachment had a high degree of significance ($p = 0.0006$). Only one study involved an intervention not in the home.[199]

Studies including a male carer achieved a good effect size and statistical significance, as did studies without, although only two studies involved a male caregiver.[191,194]

There seemed to be a number of interventions that used video feedback and a number that did not. Both achieved good effect sizes and statistical significance. The four studies that did not include video feedback used the following interventions:

i. TPP.[199,200]
ii. The UCLA FDP intervention. This sought to promote mothers' sense of self-efficacy using therapists' home visits and a mother–child group.[190–192]
iii. Home visiting, used by two interventions to develop maternal responsiveness/sensitivity.[189–192,197,198]

Researchers using an intervention to improve disorganised attachment patterns have focused on improving maternal sensitivity and attunement. This seems to work well, in that the overall effect size is good. Only one study did not focus on this area[191] and no study made direct comparisons in a RCT. However, our PPI and expert group believed that the finding that improving maternal sensitivity had a large effect size and statistical significance in meta-analysis is an important one.

All interventions involved caregiver and child, with the exception of Dozier and colleagues[194] and Bernard and colleagues.[193]

Chapter 7 Economic evaluation of parenting interventions for severe attachment problems

Introduction

Severe attachment problems is an umbrella term agreed by the review steering group to define the scope of variations in attachment most commonly associated with negative long-term outcomes (see *Chapter 1*). The term covers both the diagnosis of attachment disorders and the identification of disorganised attachment patterns. It provides a marker of those children who have not formed attachments to primary caregiving figures in early childhood, associated with healthy development. However, evidence remains unclear in terms of which identification strategies provide optimal assessment accuracy, what health benefits subsequent interventions can actually provide and whether or not treatment for various severe attachment problems represents value for money. From the decision-makers' perspective, this raises questions about the potential health benefits of interventions [commonly expressed in generic terms such as quality-adjusted life-years (QALYs)] and the wider societal implications (using a perspective beyond that of the QALY) are accrued for the investment of scarce health-care resources.

This chapter has three aims, (i) to systematically review the existing cost-effectiveness evidence on identification and/or interventions for severe attachment problems; (ii) to evaluate the feasibility of developing a de novo decision model informed by the systematic reviews (presented in the previous chapters) of evidence on the effectiveness of screening and intervention strategies in terms of short- and long-term health resource utilisation and associated outcomes (and, if feasible, other wider societal costs and benefits); and (iii) to discuss the value of information to inform future research priorities.

Section 1: systematic review of existing cost-effectiveness evidence

This phase reviewed the available literature to answer the question of whether or not treatment for severe attachment problems is cost-effective.

Methods

Search strategy

A comprehensive description of the search strategy undertaken in January 2012 has been provided in *Chapter 3*. Specific economic databases were included, namely HTA database, NHS EED, the Campbell Library and HEED.

For the cost-effectiveness systematic review, the PICOS remain congruent with those targeted by the wider review (for further details, see the PRISMA diagram in *Figure 5*).

Inclusion criteria

For inclusion in the review, a study or paper needed to meet the following criteria:

- It must have studied children with a mean age of 13 years or under.
- It must have studied children who currently had, or were at risk of developing, severe attachment problems.
- It must have examined the impact of either (a) screening, assessment and/or diagnostic tools evaluating attachment patterns or disorders, or (b) treatment using psychosocial interventions, psychotherapies or pharmacotherapies aiming to treat or prevent disorganised attachment patterns or attachment disorders.

© Queen's Printer and Controller of HMSO 2015. This work was produced by Wright *et al.* under the terms of a commissioning contract issued by the Secretary of State for Health. This issue may be freely reproduced for the purposes of private research and study and extracts (or indeed, the full report) may be included in professional journals provided that suitable acknowledgement is made and the reproduction is not associated with any form of advertising. Applications for commercial reproduction should be addressed to: NIHR Journals Library, National Institute for Health Research, Evaluation, Trials and Studies Coordinating Centre, Alpha House, University of Southampton Science Park, Southampton SO16 7NS, UK.

Only full economic evaluations that compared two or more options, and considered both costs and consequences (including cost-effectiveness, cost–utility or cost–benefit analyses), were included. Explicit guidelines laid down by the CRD in the preparation of the NHS EED were applied for this purpose.[224]

Exclusion criteria

Studies were excluded if they:

- did not fulfil the specified criteria
- did not explicitly meet criteria of a *full economic evaluation* (e.g. cost–benefit, cost-effectiveness or cost–utility analysis).

Two reviewers screened titles and abstracts to identify potentially eligible studies meeting PICOS criteria. Full papers of potentially eligible studies were obtained and assessed for inclusion independently by two health economists. The quality and relevance of any available economic data were judged from the perspective of the UK NHS according to criteria laid down by Drummond.[225]

Results

From the initial systematic searches of attachment literature, searches of the economics databases for economic evaluations yielded an additional 461 potential articles. On the basis of the titles and abstracts, only two studies[175,187] were identified as meeting PICOS as well as reporting costs alongside outcomes (*Table 18*).

For further information on how these studies were identified, see *Figure 5*.

Minnis and colleagues[187] carried out a RCT of 182 children (and their foster care families), examining the effect of extra training in attachment and communication for foster carers, compared with standard services. The additional training was based on *Communicating With Children. Helping Children in Distress*[226] (a manual used internationally by Save the Children) and was delivered by an experienced social worker or trainer with the overall aim of improving communication skills and the ability to form better attachments.

The main outcomes were child psychopathology, measures of attachment, self-esteem and costs of foster care. No formal attempt was made to map any of these outcomes on to generic health outcomes. Immediately after treatment, extra training showed no effect on attachment, and a non-significant change was observed at 9-month follow-up. At 9-month follow-up, a non-significant decrease in attachment disorders and psychopathology (around 5%) was reported. The median cost of foster care was £3792 in

TABLE 18 Identified studies of attachment containing cost–consequence analysis

Treatment (intervention vs. control)	Study design	Sample population	Population and location	Perspective adopted	Outcomes measured	Resource utilisation	Reference
(Routine care + extra training) vs. (routine care alone)	RCT	182 looked-after children	Foster care families in Scotland	Not stated	Psychopathology<br>Attachment measure<br>Self-esteem<br>Cost of foster care	Cost of treatment<br>Foster care	Minnis *et al.* (2001)[187]
RFTS (group sessions) vs. (supportive home visits)	RCT	76 mothers	General population from Canada	Not stated	Infant attachment<br>Maternal behaviour	Cost of treatment	Niccols (2008)[175]

RFTS, Right from the Start.

the intervention group and £3271 in the control, supporting the conclusion that the difference in costs of foster care associated with the intervention was also not significant. No attempt to extrapolate future costs or benefits was attempted; however, the paper does conclude that 'the cost must be offset against probable savings in later years'. These were not assessed or quantified.

Niccols[175] carried out a trial-based economic analysis comparing the intervention Right from the Start with home visits (treatment as usual). Right from the Start is a 'Coping Modelling Problem Solving Approach' which includes the delivery of eight parent group sessions held in a 'convenient location', designed to equip caregivers with the skills to read infant cues and respond sensitively. Treatment as usual was delivered by a public health professional to identify family needs and empower parents to meet the child's needs. The primary outcome was infant attachment security, assessed using the infant AQS. The maternal behaviour Q-sort also assessed levels of maternal sensitivity. Using intention-to-treat analyses, there was no significant difference in the clinical outcomes between Right from the Start and home visits (both showing small improvements in infant attachments). The assessment of costs included group facilitator time recorded in preparing, leading and supervising the session, as well as their time spent on administration. The incremental cost-effectiveness was assessed at two time points: post intervention and at 6-month follow-up – which provided a cost per unit change in attachment measures. Nichols concludes that Right from the Start is cost-effective over home visits, arguing that the study illustrates an economic advantage by avoiding more costly home visits through centrally run group sessions; Right from the Start offers a favourable return on investment in achieving the observed improvement in infant attachment. No formal attempt was made to map how the change in attachment measure maps on to generic health outcomes, or to extrapolate outcomes beyond the trial.

The studies by Minnis and colleagues[187] and Niccols[175] meet criteria of full economic evaluations by informing decision-makers of the financial consequences. However, the reported observed economic benefits of treatments are generally limited to their ability to reduce costs. The available research remains inconclusive in terms of whether or not any specific screening or treatment strategy may be cost-effective over another (or, more importantly for the NHS, over practice as usual in the UK).

Discussion

An extensive systematic search of the attachment literature suggests that only two studies[175,187] in the research to date meet criteria of a full economic evaluation; the majority of studies identified through searches of economic databases were primarily rejected for not satisfying PICOS criteria.

Two studies met explicit criteria of full economic evaluations, presenting cost consequences of interventions. Minnis and colleagues[187] conclude that there is no difference in clinical outcome nor any significant cost offset in foster care by the addition of training. Niccols[175] finds a small change in clinical outcomes (with no significant difference between comparative groups) but suggests that a favourable return on investment is possible through group sessions, as these reduce costs compared with treatment as usual.

Two major limitations were identified in these currently available economic studies of attachment: extrapolating findings beyond the study and mapping clinical outcomes on to generic measures of health. Benefits of attachment interventions may be accrued in later years; thus, extrapolation beyond the study (based on robust evidence) is likely to be more informative to the decision problem. Furthermore, clinical outcomes are reported as changes on attachment scales; such information has limited use in informing health-care decision-makers, as it is unclear how these measured changes on attachment scales map on to mediating factors, generic and mental health outcomes or other measures of health-related quality of life.

Future research is needed to address this gap in the current literature, and specific focus is required to better understand the causal relationship between changes in attachment and future generic health outcomes.

© Queen's Printer and Controller of HMSO 2015. This work was produced by Wright *et al.* under the terms of a commissioning contract issued by the Secretary of State for Health. This issue may be freely reproduced for the purposes of private research and study and extracts (or indeed, the full report) may be included in professional journals provided that suitable acknowledgement is made and the reproduction is not associated with any form of advertising. Applications for commercial reproduction should be addressed to: NIHR Journals Library, National Institute for Health Research, Evaluation, Trials and Studies Coordinating Centre, Alpha House, University of Southampton Science Park, Southampton SO16 7NS, UK.

Section 2: economic evaluation of severe attachment problems

There are a limited number of existing studies with relevance to the UK, and difficulties in interpreting findings of existing economic evaluation in terms of overall cost-effectiveness (particularly given the absence of long-term consideration of costs and benefits and of attempts to map changes in measures of attachment generic outcomes). This chapter outlines the approach taken to evaluate the feasibility of developing a decision-analytic model from the information obtained through systematic review to inform that economic case.

The conventional perspective taken in HTAs is a narrow *health-care perspective*, which examines cost and benefits directly relevant to a health-care decision-maker. This would include costs of interventions under evaluation, the related cost consequences of that intervention (e.g. how the intervention changes the need for other forms of health care) and the health benefits of the intervention.

However, this conventional HTA framework may be overly health-centric to assess treatment of severe attachment problems, as cost and benefits may be further reaching than those generally observable solely within the health-care system. For example, outcomes related to education, the employment market and the criminal justice system might be important for a decision-maker taking a wider *societal perspective*. Such perspectives can therefore be important to consider costs and benefits occurring outside the health system.

Figure 19 illustrates how health-care and non-health-mediated pathways result in health and non-health outcomes which may be considered under varying health-care or wider societal perspectives. This shows how clinical concerns (i.e. problems presenting to health services) have mediating pathways (i.e. implications of the original concern if unaddressed), and that by placing varying levels of demand on services, are policy-relevant outcomes. To simplify dynamics of the real world into a parsimonious model, this illustration omits interactions between health and non-health pathways. These omissions may be relevant to control for causation; however, it is assumed that these may be adequately captured in the interactions between policy-relevant outcomes.

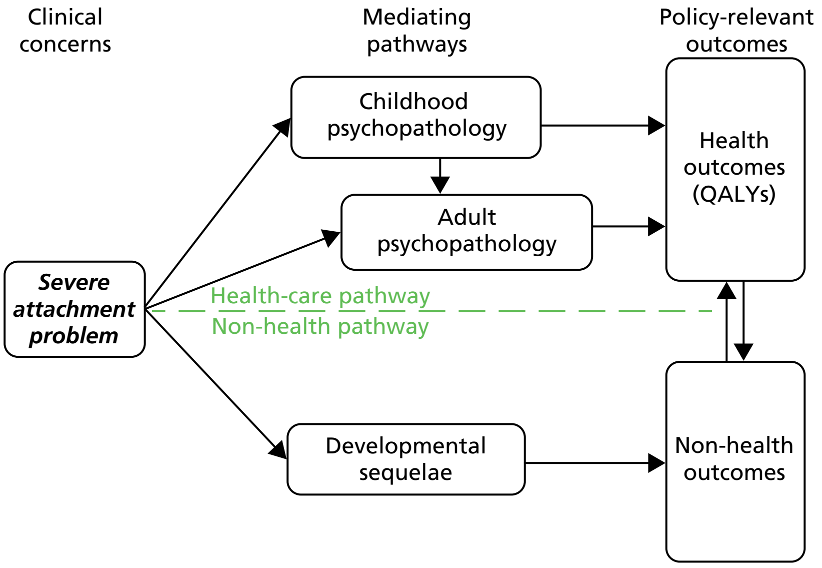

FIGURE 19 How the health-care and non-health pathways to cost and outcomes indicate health-care and wider perspectives.

A conventional HTA is primarily a means to assess how health-care resources directed at conditions (e.g. severe attachment problems) might improve health-related quality of life relative to the expected health-care expenditure. Thus, the perspective of an analysis provides a decision-maker with a tangible method to contrast the return on investment compared with other potential health-care investments. For this specific case, the aim is to provide an objective basis for comparing the relative value for money in deploying resources for the detection and treatment of severe attachment problems. As a decision rule, NICE defines a willingness-to-pay threshold of £20,000–30,000 per QALY as a decision threshold for funding health services, which provides objective valuation that severe attachment problems must demonstrate to justify funding.

Extending beyond simply health production, severe attachment problems may also have fundamental links in the lifelong development of a child, so an evaluation should consider wider implications than those summarised by the cost per QALY of the conventional HTA. A wider perspective should consider the developmental sequelae caused by severe attachment problems and the resulting resource implications for the broader public sectors (e.g. education, social services or criminal justice), implications for individuals' future productivity and the impact of enduring problems on informal carers. This provides a more comprehensive framework, capturing important non-health developmental implications of severe attachment problems and the associated implications for sectors outside health care. To build our understanding of these future trajectories for the purpose of decision analysis, the next section presents the natural history of severe attachment problems, illustrating health and developmental implications.

Natural history of severe attachment problems

Severe attachment problems are broadly divided into patterns of attachment displayed in infancy and attachment disorders presenting in older children. The scope of this definition is defined more completely in *Figure 1* (presented and discussed in *Chapter 1*). Before considering the potential costs and benefits of interventions, the decision-maker must first consider the natural history of these problems (in the short and long term) to comprehend the baseline from which an intervention has its effect. *Figure 19* illustrated how the pathways to potential outcomes of severe attachment problems may be broadly divided into those observable through health care and non-health pathways associated with developmental outcomes of the child.

Severe attachment problems are commonly cited as having implications for future health. For example, disorganised patterns of attachment are associated with heightened levels of childhood psychopathology[45] and have been linked to dissociative experiences in adulthood.[45] The association with adult borderline personality symptoms has also been researched.[45] Such research may provide the basis for future expectations as a result of attachment problems, and when parameterised in a model, can inform the expected long-term gains of treatment.

Likewise, commonly cited non-health implications of severe attachment problems have been previously summarised as *developmental sequelae* of attachment.[87] These non-health implications of attachment problems include language delays,[23] relationship problems,[27,37] antisocial behaviour, child education, and future productivity and criminality,[227] with the caveat that there are complex associations, not always linear, that interact with other risk and resilience factors.[115]

Such sequelae provide important information for decision-making when the values which society apportions to mitigating poor developmental outcomes are considered. Given that prior evidence suggests robust causal links from attachment to specific negative sequelae, the value of averting such outcomes may be informed by previous economic analyses, such as costs of averting crime,[228] poor educational attainment[229] and poor relationships.[230] Including these as parameters in a model may inform the expected future non-health costs and benefits to society and the wider economy of treatments for severe attachment problems.

© Queen's Printer and Controller of HMSO 2015. This work was produced by Wright *et al.* under the terms of a commissioning contract issued by the Secretary of State for Health. This issue may be freely reproduced for the purposes of private research and study and extracts (or indeed, the full report) may be included in professional journals provided that suitable acknowledgement is made and the reproduction is not associated with any form of advertising. Applications for commercial reproduction should be addressed to: NIHR Journals Library, National Institute for Health Research, Evaluation, Trials and Studies Coordinating Centre, Alpha House, University of Southampton Science Park, Southampton SO16 7NS, UK.

Although non-health implications merit consideration within a wider societal perspective, poor developmental outcome may also indirectly alter demands for health care. For example, methods of mapping such non-health outcomes on to predictable health status include links from educational status,[231] poor relationships[78] and criminality,[79] all of which have been demonstrated to directly affect life expectancy.

This may imply that, even within the health-care perspective adopted in the conventional HTA, developmental sequelae could demonstrate relevance to the health-care decision-maker in evaluating long-term implications for health services where direct evidence on the impact on health services utilisation is not available. Where it may be feasible for a model to robustly establish pathways to non-health outcomes, additional model parameters may seek to create tangible causal links of how non-health outcomes would indirectly influence future health-care utilisation.

The natural history of severe attachment problems suggests that economic analysis could either summarise the effect of treatment on non-health outcomes[187] or, alternatively, attempt to infer the relevance of these non-health outcomes through mapping their expected effects as cost and benefits to the health-care system (e.g. implications of educational attainment for QALYs[232] or the societal perspective).

In light of our current understanding of the natural history of severe attachment problems, the theoretical modelling framework required to address the health-care decision problem is presented. This framework is then contrasted against the data available from the systematic review. Finally, based on available parameters for the modelling framework, feasible components of the model are specified to indicate the expected budget impact of treating attachment and how many QALYs would need to be produced to justify that budgetary investment.

Methods for developing a theoretical modelling framework

Within the various classifications of attachment, our evaluation of severe attachment problems includes both disorganised patterns of attachment and attachment disorders. Each unique definition of severe attachment problems will have associated health-care decisions, such as how to identify and how to treat, and should be reinforced by how the decisions change based on the expectations for short- and long-term outcomes.

A decision model is a mathematical framework that brings together all relevant information in an attempt to reduce the decision uncertainty regarding such health-care decisions, in an attempt to efficiently allocate resources to meet demands for health care. For severe attachment problems, models should address how health-care resources are best deployed in order to (1) identify and (2) treat the problems. To elaborate further, identification and treatment components of the model are presented separately.

Identification model

The detection model precedes the intervention strategy, but this stage will cause additional resource use that needs to be considered when assessing the cost-effectiveness of intervention. Furthermore, the effectiveness and cost-effectiveness of intervention will also be related to the identification procedure (i.e. the prevalence of the problem and the accuracy of identification procedures will indicate numbers treated appropriately for a given procedure). By defining key components of the identification strategy, assessment of the cost-effectiveness of identification and intervention must ultimately be considered together. The outcome of this first model is that intervention will only be offered to individuals who screen positive, and this is subject to varying levels of assessment tool/diagnostic accuracy.

Prior to implementing an identification procedure, the population to be screened must be defined. For severe attachment problems, two types of population can be screened. The first is the general population and the second targets specified higher-risk groups. Implementation of a general population screening strategy for severe attachment problems would require that every child born within the general population within a particular age bracket would undergo screening or assessment. However, within a budget-constrained environment, screening a general population for severe attachment problems may not be economically feasible or desirable, or indeed useful.

As with all screening, there are negative as well as positive consequences,[233] such as unnecessary assessment and/or treatment in false positives, unnecessary reassurance in false negatives, stigma, health anxiety, cost and so on.

Screening populations considered to have a higher prevalence of severe attachment problems (owing to risk factors) would potentially reduce the number of individuals screened and reduce false positives and negatives. However, this would require that evidence on what constitutes an *at-risk population* has been established.

The attachment literature commonly cites populations in which prevalence of problems may be elevated. These include children with alternative caregivers[234] [such as situations where the child is adopted or placed in care (looked-after children)], including those institutionalised or in foster care; children born to lower socioeconomic groups;[235] and maltreated children. Within each target population, variation in prevalence rates would have overall implications for the cost-effectiveness of identification and subsequent management (however, the feasibility and cost of targeting screening at selective subpopulations would require further investigation before making inferences about relative value for money).

Once the target population for screening is defined, specific evidence would inform the prevalence rates and thus indicate varying numbers of children by problem type and population (previously discussed in *Chapter 4*). In the identification model specified (*Figure 20*), this provides the probability that an individual is likely to exhibit severe attachment problem types within defined populations to be screened (denoted as *P[A]*).

Diagnostic test accuracy studies compare the performance of screening strategies against a gold standard, indicating the accuracy of identification procedure in the form of *sensitivity* and *specificity*. *Figure 20* illustrates how a model, by including the reported diagnostic test accuracy of a procedure (i.e. sensitivity denoted as the probability $P[D = 1|A = 1]$ and specificity as the probability $P[D = 0|A = 0]$), predicts the expected level of the four diagnostic outcomes, namely true positive, false positive, true negative and false negative.

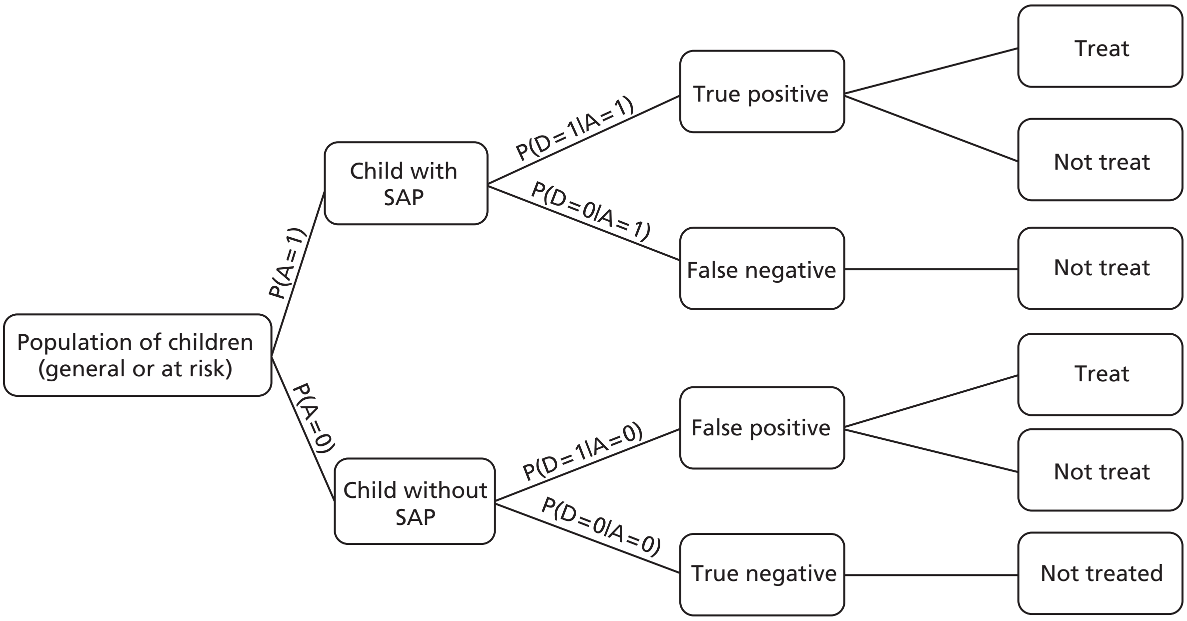

FIGURE 20 Theoretical framework of an identification model for severe attachment problems in specific population of children [A = 1/0 (child with/without severe attachment problem); D = 1/0 (screen positive/screen negative)]. SAP, severe attachment problem.

© Queen's Printer and Controller of HMSO 2015. This work was produced by Wright *et al.* under the terms of a commissioning contract issued by the Secretary of State for Health. This issue may be freely reproduced for the purposes of private research and study and extracts (or indeed, the full report) may be included in professional journals provided that suitable acknowledgement is made and the reproduction is not associated with any form of advertising. Applications for commercial reproduction should be addressed to: NIHR Journals Library, National Institute for Health Research, Evaluation, Trials and Studies Coordinating Centre, Alpha House, University of Southampton Science Park, Southampton SO16 7NS, UK.

Variations in these four diagnostic outcomes infer different decisions on intervention and, therefore, cause variation in potential costs and benefits of subsequent related intervention. Screen-negative children are not conventionally treated, and, therefore, the proportion receiving false-negative outcomes are expected to forgo potential benefits of any subsequent intervention decisions. Screen-positive outcomes are a trigger to health care generally returning for intervention, and in the absence of any further assessment information, a false-positive outcome would be likely to result in unnecessary intervention, leading to additional health service costs with no expected health benefits (and potential harms of exposure to intervention).

Intervention model

Once it has been established that a child is exhibiting a specific type of severe attachment problem (i.e. screen positive), an intervention decision will be required. For modelling purposes, *Figure 21* illustrates scenarios which should be considered at this point which, for simplicity, are described three comparators: (1) an intervention strategy based on the evidence base (e.g. as informed by information such as that identified in *Chapter 6*); (2) care or treatment as usual, which is routine care which may or may not be informed by the available evidence base; or (3) a passive approach equating to an observation of the natural history as previously illustrated (i.e. doing nothing).

Figure 21 highlights that each group will infer conditional probabilities (each contingent on the specific intervention choice) indicating the likelihood that the severe attachment problems-related outcomes can be expected to change or not.

This schematic simplifies the time horizon between the short-term gain (as observed from intervention studies) and the long-term implications of reducing the attachment problem (more often observed in longitudinal studies). In reality, studies of the causal relationship (such as those discussed in *Natural history of severe attachment problems*) are subject to analytical complexity and need to be reviewed to ascertain their relative merit in indicating potential benefits of intervention and, thus, robustness to feature within a cost-effectiveness analysis. Therefore, the time horizon of any model needs to be sufficiently long to ensure that differences between intervention strategies are adequately reflected; for this purpose only, data from epidemiological studies that follow individuals for up to 10 years from the initial indication of attachment were included in the review (see *Chapter 5*).

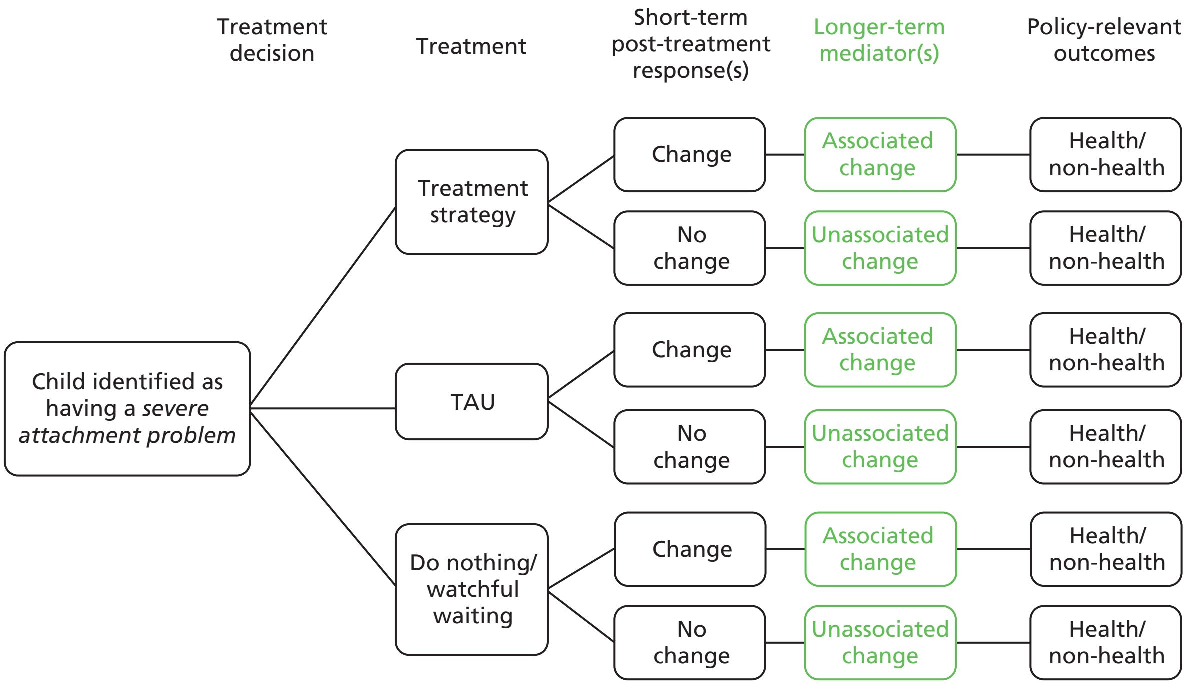

FIGURE 21 Theoretical framework of the decision model of interventions/treatments for severe attachment problems. (The term 'treatment' is used in the figure for simplicity.) TAU, treatment as usual.

Meta-analysis provides methods to inform an expected value of the probability of short-term specific outcomes (conditional on intervention or no intervention). Outcomes of the meta-analysis reported in *Chapter 6* include (a) likelihood of a secure outcome and (b) likelihood of a disorganised outcome. Point estimates of this short-term likelihood are a standard mean difference of each specific outcome between the intervention and control, where these are established using a standardised measure of attachment and will be observed at short-term per-protocol time points of the study design. Cost-effectiveness analysis may make inferences based on these outcomes, which would (at best) provide the ratio of costs per case of the stated change in attachment problem within a given time period. The majority of included studies which reported only a post-intervention effect offer little evidence that any change has been sustained. Therefore, longer-term follow-ups (e.g. at 6 or 12 months) provide a tenuous basis for extrapolation. The natural history would suggest that severe attachment problems have lifetime consequences and hence potentially both the short- and the longer-term outcomes need to be considered.

However, this raises empirical issues about the limited existing evidence base on the effectiveness of interventions themselves over a longer-term horizon. With the existing evidence base on intervention focusing on short-term intermediate outcomes, projections over a longer term (as well as translating short-term and longer-term effects into QALYs) would require several additional steps and assumptions. The validity of these additional steps in informing cost-effectiveness will depend on the robustness of existing evidence and particularly the links from epidemiological studies (see *Chapter 5*) that are inevitably required to translate the short-term intermediate outcomes into longer-term impacts.

As discussed earlier, the aim of the incremental effectiveness should be to comprehensively evaluate the longer-term benefits accrued over time as a result of each intervention. To forecast expected health benefits of treatment, the attachment problems need to be associated with longer-term implications of specific health conditions. Examples cited in the literature (see *Chapter 5*) predominantly focus on increased risk of future psychopathology (and developmental sequelae) associated with attachment problems.

Assuming that short-term treatment outcome is sustained from observed post-treatment effect and may be causally linked to the longer-term health benefits, allocation decisions are best informed when all benefits of investment are expressed by the ratio of cost per QALY. How outcomes of attachment problems map onto QALYs (either directly in the short term or indirectly through mediated pathways in the longer term) has not been established to date, and requires further research.

The array of analytic approaches presented so far is wide ranging and complex, and it is important to emphasise that within each potential pathway exists a set of highly pertinent resource implications. For example, the presence of a specific severe attachment problem may have short- and long-term implications for the intensity of health and non-health service use, such as specific teaching arrangements, or may increase the costs of foster care, and so on. Each of these stated examples would increase the demand for the scarce resource placed on the public sector and, in addition, on the individual health benefits of averting cases of attachment problems, and will also have implications for the cost of services. As such, all included studies across the main and supplementary systematic reviews (see *Chapters 4–6*) sought to extract relevant data on resource implications, and this information informed a budget impact assessment of attachment problems on the public purse.

The next section presents the key information required to populate this theoretical framework of a decision model and reviews the relevant data identified in the systematic reviews.

Key information required to populate the decision model

Any cost-effectiveness analysis must clearly define the disorder to be evaluated, population, scope and resources to be evaluated so as to form an appropriate model and address decision uncertainty surrounding the resource allocation.

© Queen's Printer and Controller of HMSO 2015. This work was produced by Wright *et al.* under the terms of a commissioning contract issued by the Secretary of State for Health. This issue may be freely reproduced for the purposes of private research and study and extracts (or indeed, the full report) may be included in professional journals provided that suitable acknowledgement is made and the reproduction is not associated with any form of advertising. Applications for commercial reproduction should be addressed to: NIHR Journals Library, National Institute for Health Research, Evaluation, Trials and Studies Coordinating Centre, Alpha House, University of Southampton Science Park, Southampton SO16 7NS, UK.

To inform the theoretical framework outlined above, specific forms of information were identified as required within the decision model. Some of this information is fundamentally related to theoretical agreement (or lack of) across the field of attachment research, and other information is more specifically required as model parameters. This section discusses eight key forms of information required to populate a decision model, describing each type of information required (and why). It also discusses how this information would be incorporated into a model and the data that were available (and, if absent, what this precludes for modelling). We present tables of information subsequently used to build the economic case.

These eight key forms of information comprise (1) definition indicators of the severe attachment problem; (2) cohort population studied; (3) prevalence estimates by definition and population type; (4) diagnostic or assessment accuracy information (sensitivity and specificity vs. reference standards); (5) treatment effectiveness by definition of attachment; (6) long-term benefits associable with the treatment effect on risk of psychopathology or (7) developmental sequelae; and (8) resource implications data related to all stages of identification, treatment and outcomes.

To ensure that any additional information was captured which might be relevant across these eight themes identified for the economic analysis, the total pool of studies identified as directly relating to attachment problems (in the main systematic review and both supplementary systematic reviews) was assessed. During full-paper reviewing, the systematic review team flagged where information might have been relevant, and any paper indicated as containing relevant information was subsequently assessed by the team of economists.

With regard to the resource implication data, two broad categories of information were assessed to provide this additional relevant information, namely information relating to direct costs and information relating to resource implications of attachment. The first form of information flagged as potentially relevant aimed to provide the basis to estimate the direct costs of health-care procedures (for both identification and treatment). The review team primarily noted where information indicated (a) duration of procedures (in units of time), (b) the types of personnel required and (c) any specific overhead costs (e.g. where a specific type of venue was required). These were subsequently utilised to inform resource implications and, associated with unit costs, expected costs of associated procedures.

The second form of information flagged during the full-paper review aimed to assess whether or not any studies contained relevant information on the resource consequence from both the health-care and the wider societal perspectives (for both identification and treatment). Potential information on resource consequences was divided into two broad categories: outcomes directly relevant to health-care use and non-health implications of attachment.

Information flagged as specifically relevant to health-care use included where papers would quantify any impacts of attachment on health-care services, resources or costs.

Studies were also flagged where information was relevant to non-health implications of attachment. Reviewers were asked to indicate where a study had quantified resource implication in contexts other than health. These included (1) the education sector, (2) alternative childcare, (3) social care, (4) informal carers (including hours of care required, changes in carers' productivity or attachment-associated impacts on carer health and health-care use) and (5) longer-term outcomes relevant to attachment (e.g. productivity in later life, social service, engagement with social, criminal justice or substance misuse services).

All studies flagged as having potentially relevant information were independently assessed for relevance by a health economist to decide (a) if the study included one of the severe attachment problems under evaluation (as flagging preceded quality assessment and final paper inclusion); (b) whether or not information flagged was relevant to the economic case; and (c) where information satisfied these criteria, whether or not any quantified incremental change associated with a severe attachment problem was sufficiently methodologically robust to inform a parameter in the intended model.

In addition to this, we separately searched for all studies published from longitudinal cohorts (such as the Minnesota study) as well as those identified by the systematic review to inform long-term outcomes of attachment.

The remainder of this section will detail the features of the required information and the questions we would address in an economic analysis, and will discuss the data that have been identified.

Defining the target: severe attachment problems

Research question What forms of attachment should be addressed by the health services?

Key information Multiple categorisations exist for both of pattern of attachment and attachment diagnosis (see *Figure 1* and *Box 1*).

Prior to treatment, formal evaluation aims to identify attachment problems requiring treatment. To define the scope covered by severe attachment problems, this research constrains the scope to disorganised attachment patterns and the attachment disorders (see *Figure 1*). For evaluation, the model considers associated data where only one of these two classifications is referenced in research.

Data available Various classifications and diagnoses were identified in the available literature, namely for patterns of attachment disorganised and organised, and within the organised category various subgroups are identified (e.g. secure, insecure avoidant, insecure ambivalent etc.) and for attachment disorders (e.g. reactive, disorganised, inhibited and disinhibited) – an extensive discussion of definitions is available in *Chapter 4*. Of these definitions, this evaluation considers only disorganised patterns and attachment disorder as severe attachment problems (although, as discussed in previous chapters, it is recognised that some attachment theorists may have selected other groups to identify in terms of intervention). The selection of these definitions for severe attachment problems is primarily based on expert opinion that disorganised patterns of attachment and the attachment disorders are most related to poor outcomes for the individual.

Target populations

Research question To which specific populations should resources be directed?

Key information The attachment literature broadly divides studies within the general population and studies that define their sample as exhibiting greater risk of attachment problems (e.g. the Minnesota study of a cohort of children born into poverty). The target population of the samples will have a direct effect on the estimates of identification, prevalence, long-term outcomes and, potentially, treatment. For example, the expected prevalence is found to vary contingent on whether or not the child is randomly sampled from the general population, is born into poverty, has alternative caregivers (e.g. adopted or fostered) or has experienced maltreatment. This form of information may support the notion of 'risk' and is also important to examine where resources are most appropriately directed.

Data available Studies define samples either within a general population or within defined subpopulations. Information on these sample characteristics are recorded as are variables for subsequent general or subgroup analysis.

© Queen's Printer and Controller of HMSO 2015. This work was produced by Wright *et al.* under the terms of a commissioning contract issued by the Secretary of State for Health. This issue may be freely reproduced for the purposes of private research and study and extracts (or indeed, the full report) may be included in professional journals provided that suitable acknowledgement is made and the reproduction is not associated with any form of advertising. Applications for commercial reproduction should be addressed to: NIHR Journals Library, National Institute for Health Research, Evaluation, Trials and Studies Coordinating Centre, Alpha House, University of Southampton Science Park, Southampton SO16 7NS, UK.

Prevalence of severe attachment problem

Research question What is the prevalence of the various severe attachment problems?

Key information Statistics on prevalence provide the basis to indicate the level of demand within populations and this parameter information may express the percentage of individuals exhibiting the specific severe attachment problem within a given cohort sample (see *Chapter 5*).

Data available Constraining the classification of severe attachment problems to disorganised patterns of attachment and the attachment disorders, average prevalence studies of similar target populations were obtained. Eight studies in supplementary systematic review 2 extracted figures on prevalence to the specific classification of severe attachment problems.[23,45,151,152,155,157,236] Additional studies were scoped on advice from content experts. *Table 19* provides the prevalence of both disorganised attachment and attachment disorder. Prevalence data are considered here in four identified potential target populations.

Consultation with content experts on parameter inputs suggest that *risk* is potentially highest in situations of maltreatment and, for this reason, Van IJzendoorn and colleagues,[23] a group that has performed several previous systematic reviews in the field of attachment, was utilised to inform prevalence in this population.

While the literature showed prevalence data within our systematic review for disorganised attachment patterns, no studies were identified in the supplementary systematic review of outcomes of 10 years or more to inform the rates of attachment disorders. Consultation with experts on the advisory panel recommend that this model parameter be provisionally explored using recent research.[237] This research finds that the prevalence of RAD in the general population to be 1.4%. (This paper was published in 2013 after the cut-off point for our systematic review.)

Overall, limited information is available by target population and pooling figures without acknowledging the differences in underlying sample would ignore heterogeneity in the parameter input.

TABLE 19 Type of severe attachment problem, sample population in which prevalence was measured and the measures of prevalence (mean, minimum and maximum) from the available literature

Type of severe attachment problem	Sample population	Prevalence ,%			Related references
		Mean[a]	Min.	Max.	
RAD	General	1.4	–	–	Minnis *et al.*[236] (2013)[b]
Disorganised pattern of attachment	General	3	–	–	Dan *et al.* (2011)[155]
Disorganised pattern of attachment	Middle class	13.0	8	20	Aikins *et al.* (2009),[151] Steele *et al.* (2002)[157]
Disorganised pattern of attachment	Adopted children[c]	16	–	–	Jaffari-Bimmel *et al.* (2006)[156]
Disorganised pattern of attachment	Born into poverty[c]	37.5	35	40	Weinfield *et al.* (2004),[152] Carlson (1998)[45]
Disorganised pattern of attachment	Maltreatment[c]	48	–	–	Van IJzendoorn *et al.*[23] (1999)[c]

Max., maximum; min., minimum.
a Calculation of mean prevalence does not account for heterogeneity between and within studies and caution is advised in generalising these estimates to the UK context and any results are considered illustrative of potential variance underlying *severe attachment problem*.
b Van IJzendoorn and Bakermans-Kranenburg[23] is a meta-analysis and did not meet the criteria for our review. Minnis *et al.*[236] was not included in the systematic review because it was past the cut-off date for inclusion. Both of these papers were suggested by the content experts to provide useful comparative data for health economic analysis.
c Mean *at-risk* groups presented above fall into two aggregated definitions: (1) prevalence estimates from Minnesota studies define *risk* as children born into poverty and (2) the study by Jaffari-Bimmel *et al.*[156] studies internationally adopted children.

Identification strategies

Research question How are severe attachment problems best identified?

Key information Prior to treatment, a formal identification procedure (e.g. screening) must take place and this process has additional resource implications that need to be considered. To inform the relative merits of various identification strategies, studies reporting sensitivity and specificity data should be incorporated into a decision model to provide outcomes of each approach to identification.

Data available As discussed in *Chapter 4*, while a gold standard identification method for disorganised patterns of attachment is implied in much of the literature, this has not been clearly agreed, and the economic analysis does not have comparative diagnostic or assessment accuracy studies to inform this parameter.

In *Chapter 4*, reviewers refer to a 'reference standard'; however, comparisons with other screening tools (through calculated accuracy data) do not consider the conventional economic trade-offs that are obtained using established 'gold standard' methods (which assume perfect diagnostic precision usually at a larger cost, thus potentially justifying the reduced accuracy of screening). Many other proposed instruments in the literature are used but have not been compared with the available reference standards (as identified in our first supplementary systematic review of assessment tools). It is, therefore, difficult to use the sensitivity and specificity of any specific instruments (compared with a gold standard) as a basis to determine their accuracy given their relative costs.

The reviewers identified two studies that concurrently compared the SSP with an alternative attachment pattern measure and where data were available to calculate sensitivity and specificity of the procedures versus this reference standard (see *Chapter 4*). However, this analysis only allowed accuracy data to be calculated for secure versus insecure categorisations and does not provide information on identification of *disorganised patterns of attachment,* which is the category used by our review; therefore, these data are not applicable within the scope of this definition of severe attachment problems.

For attachment disorder, no diagnostic studies were identified comparing identification procedures for attachment disorder with DSM-IV[56] or ICD-10[55] classification, with the exception of a study comparing the DAI with the SSP.[95]

In the absence of diagnostic accuracy data, policy implications of identification strategies were informed by collecting information on the resource implications of procedures (further detail is provided later; see *Resource data*).

Intervention/treatment effect

Research question Once a *severe attachment problem* is identified, how effective is an intervention?

Key information The effectiveness of an intervention has been informed by meta-analysis of studies (see *Chapter 6*).

Data available Meta-analysis providing the likelihood of treatment leading to (a) 'secure outcome' and (b) improving 'disorganised attachment'; given the defined scope of this evaluation of *severe attachment problems*, the parameter input is constrained to the latter.

Meta-analysis of eight studies of treatments of disorganised attachment provides an OR of 0.47.[189,191,194–196,198,200,218] This indicates that individuals in the intervention arm are significantly less likely than control to exhibit disorganised patterns of attachment at follow-up. Cooper[189] is the only identified study that has a follow-up of 12 months or longer; this study did not find any significant differences in outcomes at this follow-up.

© Queen's Printer and Controller of HMSO 2015. This work was produced by Wright *et al.* under the terms of a commissioning contract issued by the Secretary of State for Health. This issue may be freely reproduced for the purposes of private research and study and extracts (or indeed, the full report) may be included in professional journals provided that suitable acknowledgement is made and the reproduction is not associated with any form of advertising. Applications for commercial reproduction should be addressed to: NIHR Journals Library, National Institute for Health Research, Evaluation, Trials and Studies Coordinating Centre, Alpha House, University of Southampton Science Park, Southampton SO16 7NS, UK.

The average intervention effects may be combined for cost information to indicate a cost per case of severe attachment problem averted. Although this would indicate cost-effectiveness, this would only be relevant within the study time frame (generally less than 12 months) and could only (in the absence of reported generic health outcomes) relate to changes in rating scales of attachment (using the reference standard). Therefore, meta-analysis can only inform the presence or absence of severe attachment problem. Additional analysis is required (a) to forecast longer-term outcomes of changes in these short-term health states and (b) to map short- and long-term outcomes onto policy relevant outcomes, QALYs or other relevant non-health outcomes (e.g. foster care, education and criminality).

Health outcomes

Research question Given the short-term changes in health state (i.e. presence or absence of severe attachment problems), what are the long-term generic health outcomes associable to the short-term change from treatment?

Key information To attempt to show how the intervention effect might inform allocation of scarce resources, clinical outcomes should be expressed in a generic health outcome. Ideally, economic evaluations aim to present outcomes in QALYs. However, there exists no defined method for mapping clinical outcomes of using interventions to improve attachment onto generic health outcomes or QALYs. In the absence of such methods, the economic review of the available literature focused on the studies identified in the main systematic review and the second supplementary review of outcomes of 10 years or more, examining the feasibility of inferring long-term implications of attachment via mediated health pathways.

To inform the direct health benefits of treating either attachment disorder or intervening to reduce disorganised attachment patterns, longitudinal studies were examined with at least 10-year follow-up to inform risk of psychopathology related to attachment problems (see *Chapter 5*) and any treatment studies that indicated relevant secondary health outcomes (see *Chapter 6*).

Figure 22 illustrates information potentially useful to inform longer-term outcomes associated with severe attachment problems. These modelling frameworks are divided into two causal pathways related to psychopathology. The primary pathways relate to policy-relevant health outcomes (predominantly the literature regarding the risk of psychopathology given a severe attachment problem). The second is how resulting psychopathology caused by severe attachment problems cause non-health policy-relevant outcomes [e.g. intellectual quotient (IQ), educational attainment or criminality].

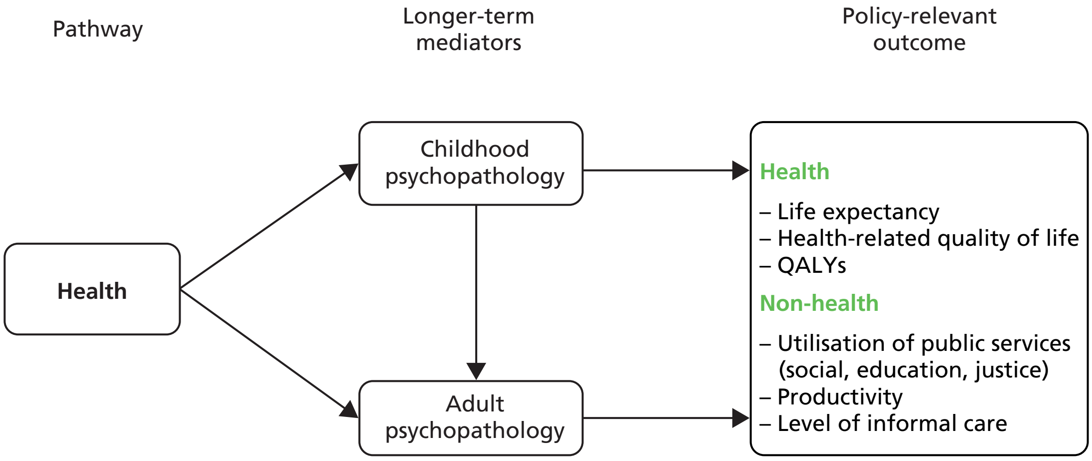

FIGURE 22 Health mediated pathway from severe attachment problem post-treatment effects via longer-term mediators (such as child and adult levels of psychopathology) onto health and non-health outcomes.

Data available Despite the common hypothesis that severe attachment problems (particularly a disorganised attachment pattern) are associated with poor future psychopathology, only a limited number of studies with follow-ups of over 10 years were identified that provide any evidence to support this notion. As was shown in *Chapter 5*, only analysis of the Minnesota study cohort potentially provided any potentially meaningful information from three studies. Specific studies of disorganised attachment examine long-term outcomes such as an overall history of psychopathology at age 17 years,[45] dissociative experiences at age 19 years[45] and borderline personality symptoms at age 28 years.[153]

Carlson[45] utilises the Minnesota study to examine how disorganised attachment explains (a) the overall history of psychopathology at age 17 years and (b) dissociative experiences at age 19 years. 'Disorganised/Disoriented Attachment' is found to have a correlation of 0.34 with psychopathological rating at age 17.5 years and correlation of 0.36 with dissociation at age 19 years. Carlson then utilises hierarchical regression to examine how 'Disorganised/Disoriented Attachment' predicts psychopathology rating and levels of dissociation. Controlling for avoidant attachment, Carlson finds that 'Disorganised/Disoriented Attachment' increases the hierarchical regression analysis explain an additional 5% of the variance in future psychopathology through including 'Disorganisation rating (12–18 months)'. Similarly, 'Disorganised/Disoriented Attachment' is found to explain 12% of the variance in dissociation score at age 19 years. These findings have limited use for modeling, as they only show small proportional predictive power of disorganised attachment on future events and do not provide a causal link. Until there is future replication and more in-depth research to examine the nature of the associations with specific psychopathologies, they are of limited value for modelling.

Carlson and colleagues[153] again utilise data from the Minnesota study to examine the relationship between borderline personality disorder at age 28 years and 'attachment disorganisation' between 12 and 18 months. The research shows a weak correlation (0.20) between disorganised attachment at 12–18 months and borderline personality disorder at age 28 years. When placed into a binomial regression alongside other variables, attachment pattern is not significant, although maternal hostility and maternal life stress at 42 months are significant.

As the research did not provide any strong causal link from early severe attachment problems to future psychopathology, no further analysis was feasible to identify potential means to map these health states onto generic health outcomes.

Developmental mediators and indirect health outcomes

Research question Given the short-term changes in health state (i.e. presence or absence of severe attachment problems), what are the long-term developmental sequelae associable to the short-term change from intervention and can these be used to map to long-term health outcomes?

Key information In addition to associations with risks of psychopathology, the attachment literature commonly cites an increased risk of poor developmental outcomes. The common examples are development of personality, intellectual ability, educational attainment and ability to sustain romantic relationships. For modelling purposes, a plausible hypothesis is that an impaired development of such traits could indirectly mediate poor mental and physical health (e.g. education attainment has been associated with health). Again, to inform parameters to forecast such long-term outcomes, studies from both the main systematic review and the second supplementary review were assessed.

Figure 23 highlights information on three developmental sequelae considered, namely IQ (child or adult outcomes), educational outcomes and criminality. To inform parameters in the model, these three sources of information require significant scrutiny for statistical rigor and ability to inform causality in the model. Satisfying these prerequisites, further modelling strategies seek to causally link the longer-term mediators with health and non-health outcomes.

© Queen's Printer and Controller of HMSO 2015. This work was produced by Wright *et al.* under the terms of a commissioning contract issued by the Secretary of State for Health. This issue may be freely reproduced for the purposes of private research and study and extracts (or indeed, the full report) may be included in professional journals provided that suitable acknowledgement is made and the reproduction is not associated with any form of advertising. Applications for commercial reproduction should be addressed to: NIHR Journals Library, National Institute for Health Research, Evaluation, Trials and Studies Coordinating Centre, Alpha House, University of Southampton Science Park, Southampton SO16 7NS, UK.

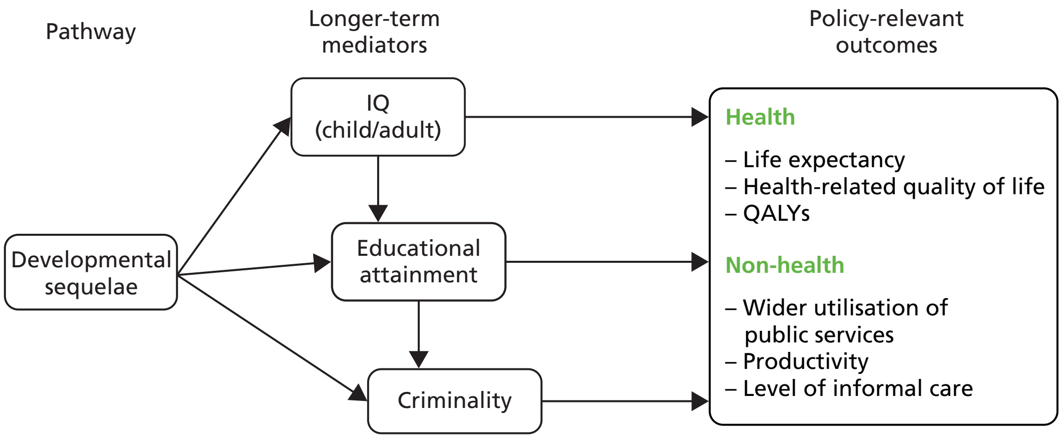

FIGURE 23 Mediating pathway of developmental sequelae from the post-treatment effects via long-term mediators (such as IQ, educational attainment and criminality) onto health and non-health outcomes.

In anticipation that the literature would provide evidence on severe attachment problems having an association with developmental sequelae, a series of further informal searches were undertaken by the health economists to identify any mediating outcomes that provide a basis for quantifying costs or longer-term outcomes. These searches provisionally identified linkages with various cited sequelae to crime,[228] poor educational attainment[229] and poor relationships.[230]

Data available Despite claims that severe attachment problems have associations with developmental sequelae, the review uncovers limited good-quality evidence to support this preposition. This is to say not that there is no association, but that the evidence to support it is sparse. Specifically, one study[184] attempts to report the effect of attachment problems on IQ as a secondary outcome (but the study reports no significant difference in IQ). No studies were identified to indicate that severe attachment problems have any effect on educational outcomes or are linked to risk of criminality.

The lack of evidence on any direct or indirect (intermediate) link between attachment (and related interventions) and final outcomes in the published literature precludes estimation of longer-term cost and outcomes within a decision-analytic framework. In the absence of evidence causally linking severe attachment problems with developmental sequelae, no further attempts are made to model these outcomes.

Resource data

Research questions What is the demand placed on resources (and associated costs) of providing assessment/diagnostic and intervention services for severe attachment problems? Does providing services for severe attachment problems offset costs elsewhere?

Key information Information on resources was sought across all three systematic reviews. Resource-use data are categorised into three sections: identification, intervention and outcomes.

Identification To inform budget impact of identifying cases of severe attachment problems, the prevalence data that we had available were combined with the cost of each specific assessment/diagnostic test to calculate an average cost of case detected. In the absence of measures of sensitivity and specificity, it is not feasible to indicate the proportion of cases accurately detected. For this reason, provisional calculations primarily assume that each strategy would perfectly diagnose all cases (and will have no false positives) and this assumption is later subject to simple deterministic sensitivity analysis to illustrate the effect of variation in identification accuracy.

Treatment To indicate whether or not any specific intervention is cost-effective, the resource intensity of providing each intervention must be extracted and appropriate unit costs applied. Parameter information detailing the protocol of intervention was extracted from each treatment study in the systematic review (see *Chapter 6*).

Outcomes To indicate cost implications of severe attachment problems, two forms of longer-term resource data were sought, namely health-care resource use where severe attachment problems occur and wider societal costs resulting in altered consumption of other public goods (e.g. education, social services, criminal justice) as well as indication of altered productivity (either during education or in later life workforce participation).

Data available *Identification* Data were available in most assessment/diagnostic studies to estimate costs of conducting screening. Parameter information extracted to associate cost was the duration of the identification procedure, the personnel required and the location.

Tables 20 and *21* estimate costs associated with the various detection strategies identified by the first supplementary systematic review for disorganised attachment patterns and attachment disorders, respectively. A cost of the procedures could be estimated only where sufficient detail was provided about the assessment/diagnostic procedure to quantify resource use. These tables indicates the reported personnel time

TABLE 20 Identification strategy, resources required and the estimated cost to the NHS for identifying disorganised patterns of attachment

	Resources			
Identification procedure	Personnel	Face to face (minutes)	Cost, £[a]	Related reference
SSP (Ainsworth *et al.* 1978)[8]	CAMHS team worker[b]	20	29	Crittenden *et al.* (2007)[100]
SSP (Ainsworth *et al.* 1978)[8] and AQS	Clinical psychologist	120	272	Boris *et al.* (2004)[98]
AQS v3.0	Clinical psychologist	120	272	Smeekens *et al.* (2009)[117]
MCDC scales	Clinical psychologist	65	147	Bureau *et al.* (2009)[99]
CAI	Clinical psychologist	50	113	Shmueli *et al.* (2008)[52]
CMCAST	Child psychiatrist	22	117	Minnis *et al.* (2010)[107]

a Unit costs of personnel are the values for 2011–12 including face-to-face and non-contact time at a ratio of (a) 1 : 1.06 for generic single-disciplinary CAMHS team – total staff cost of £86 per hour; and (b) 1 : 1.25 for clinical psychologist – total staff cost of £136 per hour (Personal Social Services Research Unit 2012).[237]
b CAMHS team worker for identification relates to the various individuals who may perform a SSP, including a clinical psychologist, a mental health nurse, a social worker and an occupational therapist.

TABLE 21 Identification strategy, resources required and the estimated cost to the NHS for identifying an attachment disorder

	Resources required			
Identification procedure	Personnel	Time (minutes)	Cost, £[a]	Related reference
RAD children (screened with ICD-10)	Child psychiatrist	17	90	Minnis *et al.* (2010) UK[107]
ICD-10	Child psychiatrist	210	1117	Equit *et al.* (2011) Germany[101]
CAPA-RAD	Child psychiatrist	22.5	120	Minnis *et al.* (2009) UK;[108] McLaughlin *et al.* (2010) UK[109]

CAPA-RAD, Child and Adolescent Psychiatric Assessment – reactive attachment disorder.
a Unit costs are the values for 2011–12 of personnel including face-to-face and non-contact time at a ratio of 1 : 1.58. Total cost £319 per hour (Personal Social Services Research Unit 2012).[237]

© Queen's Printer and Controller of HMSO 2015. This work was produced by Wright *et al.* under the terms of a commissioning contract issued by the Secretary of State for Health. This issue may be freely reproduced for the purposes of private research and study and extracts (or indeed, the full report) may be included in professional journals provided that suitable acknowledgement is made and the reproduction is not associated with any form of advertising. Applications for commercial reproduction should be addressed to: NIHR Journals Library, National Institute for Health Research, Evaluation, Trials and Studies Coordinating Centre, Alpha House, University of Southampton Science Park, Southampton SO16 7NS, UK.

taken to administer an assessment or diagnostic procedure and a unit cost of that time related the personnel from Personal Social Services Research Unit (PSSRU) total staff costs (accounting for the staff wage and overhead costs which reflect the costs of service facilities).[238] The figures indicate an approximation of cost to the NHS of each procedure, and take the average of all screening procedures indicating an average cost per screen. This does not take into account the sensitivity and specificity of procedures and so does not indicate cost per case accurately detected (also bearing in mind that assessment/diagnostic property would vary for severe attachment problem classification and with the type of population to be screened).

Although five studies are referenced as providing sufficient information to inform the cost of identifying disorganised attachment patterns, there are important differences in the above procedure that should be reiterated. First, the SSP is appropriate for use in infants, while MCDC[99] and CAI[52] are designed to identify patterns in later childhood (ages 7–9 and 8–13 years, respectively). Strategies including the Q-sort are measuring other constructs such as maternal sensitivity and are not specific to disorganised patterns; therefore, they are excluded from the mean cost. As such, only information on the SSP is subsequently used to indicate a cost of identifying disorganised patterns of attachment.

Equit and colleagues[101] reference the use of ICD-10[55] as the method for detecting attachment disorders; however, as this is a full assessment (reflected in the cost) and not specific to attachment, this outlying cost is excluded within the mean cost of identification.

Intervention Information was available in most assessment tool studies to estimate costs of delivering various interventions included in the systematic review. Parameter information on the duration of the intervention, the personnel, the location required for intervention and required travel are utilised to indicate the resource intensity of each strategy. The absence of robust information on the sensitivity and specificity (for either disorganised patterns of attachment or attachment disorders) is an important limitation for estimation of resource implications.

Tables 22 and *23* calculate the costs of intervention for disorganised attachment patterns and attachment disorders, respectively. This uses specific information from each study on the total number of sessions, the reported duration of each session, the staffing required (and the unit cost of their time), and calculates a cost per session and as well as the total cost of treatment. PSSRU (2012) unit costs data provide a total staff cost which incorporates the costs of required staff salary as well as covering overhead costs required to provide specific services (e.g. travel, non-face-to-face time).

TABLE 22 Intervention for improving or preventing disorganised attachment, required resource inputs (type of personnel performing the intervention, duration of each session and total number of sessions) and the estimated cost to the NHS for the treatment

	Resources required for treatment				
Treatment for disorganised pattern	Personnel	Duration of session (minutes)	Number of sessions	Cost, £[a]	Related reference
The COS-4 intervention	Clinical psychologist	60	4	544	Cassidy *et al.* (2011)[196]
Treatment name was not reported	Nurse (mental health)/social worker (children's services)	60	16	1072	Cooper *et al.* (2009)[189]
ABC	Social worker (children's services)	60	10	1500	Bernard *et al.* (2012);[193] Dozier *et al.* (2009)[194]
UCLA FDP	Nurse (mental health)	60	78	5226	Heinicke *et al.* (1999)[191]
UCLA FDP	Nurse (mental health)	60	54	3618	Heinicke *et al.* (2001)[190]

a Where more than one different type of staff is stated, the mean average total staff cost is calculated per hour. Unit costs are 2011–12 values (PSSRU 2012).[238]

TABLE 23 Treatment of RADs, required resource inputs (type of personnel performing the intervention, duration of each session and total number of sessions) and the estimated cost to the NHS for the treatment

	Resources required for treatment				
Treatment for disorganised pattern	Personnel	Duration of session (minutes)	Number of sessions	Cost, £	Related reference
Intervention name was not reported (extra training above routine care)	Social worker (children's services)	360	3	2700	Minnis *et al.* (2001)[187]

Unit cost is the values for 2011–12 of personnel including face-to-face and non-contact time at a ratio of 1 : 1.58. Total £319 per hour (PSSRU 2012).[238]

All studies in *Tables 22* and *23* above were included in the meta-analysis of intervention effect. Variation exists in the resource intensity of each intervention which may partly explain between-study heterogeneity.

Outcomes Aside from the direct costs of providing the intervention, very little information is available to inform the cost consequences of severe attachment problems. Associated consequences of resources were flagged in the systematic review for health service use and for wider related costs. The two exceptions are information provided in the studies including economic analysis.[175,187] However, as was discussed earlier, both studies have limitations in their usefulness to inform long-term cost implications of treating severe attachment problems.

Cost of identification and intervention are combined, at which stage the assumption of 'a perfect prediction of severe attachment problem' is subject to sensitivity analysis to simulate the potential budget implication of variation in diagnostic precision.

Feasibility of developing an economic model given available evidence

As has been discussed, developing a full economic model for severe attachment problems (such as disorganised attachment or attachment disorders) requires specific data informed by systematic review of the research literature. The systematic review developed a search strategy that has identified all available evidence on identification, intervention and long-term (longitudinal) outcomes. From this extensive and comprehensive review, evidence synthesis attempted to populate the theoretical decision model requiring eight specific sets of parameters (as described above).

Table 24 reviews these eight sets of parameters required to develop an appropriate decision model, a rating of the quality of the information to inform model parameters (an explanation of how this was rated is provided below the table) and specific details relating to each category of information. This table provides an important summary of the strengths and weaknesses of the current literature to inform a full economic model. This table helps to illustrate how future research might develop an evidence base equipped to better inform the use of scarce health care resources in the identification and treatment of severe attachment problems.

Three specific gaps exist in the literature imposing data limitation on whether or not a full decision model for severe attachment problem is currently feasible, namely assessment tool diagnostic accuracy studies (reporting sensitivity and specificity relative to an agreed gold standard), treatment effectiveness studies and evidence on the longer-term outcomes of health and development.

A major limitation for modelling extrapolating is how associated treatment effects on severe attachment problems (as measured by the associated scales) influence long-term outcomes. Having considered mediating pathways (to form the basis of projecting outcomes beyond those intervention outcomes studied), both health and wider development outcomes measured in the identified longitudinal studies provide limited robust evidence of causal links from early attachment.

© Queen's Printer and Controller of HMSO 2015. This work was produced by Wright *et al.* under the terms of a commissioning contract issued by the Secretary of State for Health. This issue may be freely reproduced for the purposes of private research and study and extracts (or indeed, the full report) may be included in professional journals provided that suitable acknowledgement is made and the reproduction is not associated with any form of advertising. Applications for commercial reproduction should be addressed to: NIHR Journals Library, National Institute for Health Research, Evaluation, Trials and Studies Coordinating Centre, Alpha House, University of Southampton Science Park, Southampton SO16 7NS, UK.

TABLE 24 Required information to inform a decision model, the quality of evidence currently identified and specific details of each category

	Quality of information from:			
Information for decision modelling	Identification accuracy studies	Epidemiological studies	Intervention studies	Summary
Definitions of attachment	1	2	2	Severe attachment problems are constrained to disorganised attachment patterns and the disorders. Limited studies by included definitions were found across the three systematic reviews
Within specific subpopulations	1	2	2	Disorganised: sampling from subpopulation is common across the three reviews; however, minimal numbers of studies for each specific subpopulation<br><br>Disorder: one 'general population'; no studies in specific subgroups (unlike in disorganised attachment)
Prevalence	N/A	2	N/A	Disorganised: eight (longitudinal) studies reporting prevalence (from varying sample types) from supplementary review 2<br><br>Disorder: no prevalence study identified
Identification strategies	0	0	N/A	No identification studies identified
Intervention effect	N/A	0	2	Disorganised: meta-analysis of nine studies with primary outcomes of 'disorganised attachment'. $I^2 = 31\%$ indicates low to medium levels of heterogeneity<br><br>Disorder: one good-quality study identified incorporating economic analysis alongside clinical trial
Long-term health outcomes	N/A	1	0	No evidence demonstrating causal link of early severe attachment problem with long-term health outcomes
Developmental sequelae	N/A	0	0	No evidence demonstrating causal link of early severe attachment problem with non-health outcomes
Economic analysis	1	0	2	Limited number of studies in each phases were identified to estimate economic analysis or resource implication

N/A, not applicable.
Scores involve only studies that contain either attachment disorders or disorganised attachment patterns.
0: No studies containing this information.
1: 0–9 studies but some limits in scope or quantity or quality and no clarity or agreement established.
2: 0–9 studies with good quality and high levels of agreement between them.

Second, limited evidence is currently available to indicate the assessment tool/diagnostic test accuracy of available forms of identification. In the absence of definitive information (or an established gold standard for attachment patterns), the economic model cannot form a link between the identification and intervention model.

In summary, comparing the theoretical framework required to model severe attachment problems with the parameter information available through systematic review suggests that a full economic model of cost-effectiveness is not currently feasible. However, given the limited evidence, the economic analysis can inform specific important components of the economic case for severe attachment problems; the next section presents the budget impact analysis based on constrained information available to provisionally inform the economic case for severe attachment problems.

Informing the economic case for severe attachment problems

In the absence of sufficient information to inform a full economic model of cost-effectiveness, this section utilises discrete components of the available information to inform the economic case for severe attachment problems by assessing the budget impact of detection and subsequent intervention.

The remainder of this chapter will present the available information to assess the budget impact of (1) detection and (2) intervention, and then this information will be combined to indicate the budget impact assessment of parenting interventions for severe attachment problems.

Budget impact assessment of providing identification strategies

As discussed, there are four key parameters providing information required to assess the budget impact of seeking to detect severe attachment problem. These are (1) classification of severe attachment problem, (2) the type of population studied and its size, (3) the prevalence (contingent on cohort profile) and (4) information on the assessment tool test (limited in this case to only resource information given the lack of a gold standard test to indicate sensitivity and specificity).

This budget impact assessment is limited to costs incurred and cannot consider cost consequences of the identification outcomes (i.e. the cost of false positives unnecessarily receiving treatment or the resource implications of false negatives going untreated).

The expected budget impact of screening strategies was assessed within the context of a Clinical Commissioning Group (CCG). Assuming that all children born in a CCG were to be screened at a certain age after birth (identified studies generally report prevalence of disorganised patterns of attachment after approximately 12 months), the number of screens per year would be equal to the number of births. Using an estimated birth rate of 12.26 births per 1000 population,[239] this suggests that, for the UK population (63.2 million in 2013), the number of births in 2013 was 774,832. Assuming the average CCG in the UK covers 264,039 individuals,[240] the expected cohort that could be screened in the general population would be 3237 newborn children within the average CCG.

Table 25 presents budget impact assessment of detection strategies aiming to identify disorganised patterns of attachment through screening of target population. This table presents the percentage of the general population targeted for screening, the expected number to screen (in our hypothetical average CCG), the total cost across this CCG, prevalence and number of children showing disorganised patterns of attachment expected to be detected assuming perfect accuracy (minimum–maximum) and, in combination, the expected cost per case detected.

In this review, the SSP is the most commonly cited procedure to assess patterns of attachment in infancy and information from the systematic review indicates an average cost of £29 to conduct this procedure. The total cost of running the screening strategy across the general population of a CCG would be £93,873. Assuming perfect assessment tool/diagnostic accuracy and for illustrative purposes that 3% of

© Queen's Printer and Controller of HMSO 2015. This work was produced by Wright *et al.* under the terms of a commissioning contract issued by the Secretary of State for Health. This issue may be freely reproduced for the purposes of private research and study and extracts (or indeed, the full report) may be included in professional journals provided that suitable acknowledgement is made and the reproduction is not associated with any form of advertising. Applications for commercial reproduction should be addressed to: NIHR Journals Library, National Institute for Health Research, Evaluation, Trials and Studies Coordinating Centre, Alpha House, University of Southampton Science Park, Southampton SO16 7NS, UK.

TABLE 25 Budget impact assessment of identification strategies to detect disorganised patterns of attachment

Severe attachment problem	Target population (percentage of general population)	Expected number to screen (per average CCG)	Total cost to CCG, £	Prevalence of pattern by target (min.–max.)	Expected number of children showing problems detected (min.–max.)[a]	Mean cost per case detected (min.–max.), £
Disorganised attachment	General (100%)	3237	93,873	3%	97	968
Disorganised attachment	Middle class (25%)	809	23,461	13.0% (8–20%)	105 (65–162)	223 (77–363)
Disorganised attachment	Born into poverty (16%)[b]	518	15,022	37.5% (35–40%)	194 (181–207)	77 (73–83)
Disorganised attachment	Alternative caregiver (0.59%)	19	551	16%	3	184
Disorganised attachment	Maltreated (0.42%)[c]	14	406	48%	7	58

Max., maximum; min., minimum.

a In the absence of sensitivity and specificity, the number of cases detected is based on the assumption that the identification strategy is perfectly accurate.

b World Development Indicators.[241]

c Maltreatment figures were not identified in the systematic review but are included purely for illustrative purposes. The population to be screened is defined as a subset of children with 'alternative caregivers', as 72% of children in alternative care had experienced neglect.[242] This may omit other children identified by services as maltreated and a retrospective survey would find a figure as high as 7%;[243] however, the majority go undetected by any formal service.

children born in the general population will exhibit a disorganised pattern of attachment, this strategy would detect approximately 97 individuals with disorganised patterns of attachment. The cost per case (assumed correctly) detected would be £968.

Subpopulations would seem to exhibit variation in prevalence rates and this could potentially form the basis for targeted screening strategies (i.e. target screening where prevalence is known to be elevated). Such strategies might reduce the overall cost by decreasing the total number of individuals requiring resources needed to perform a SSP. Although this would reduce the overall budget impact, there is no evidence to suggest that one subpopulation encompasses all the cases of disorganised attachment and, therefore, such strategies might not identify all potential cases present in the general population and this trade-off must also explicitly be considered. Further research may be required to specifically evaluate the accuracy of screening by subpopulation type.

Accepting these caveats, and based on the prevalence identified in the systematic review, the lowest estimated budget impact would be through targeting screening of children born into poverty (indicated prevalence associated with maltreatment indicates lower budget impact; however, this prevalence did not meet the inclusion criteria of the systematic review and should be treated with caution). Assuming that the percentage of most people in the low socioeconomic groups in the population correctly indicates the proportion of children born in poor families, this form of strategy could be expected to screen 518 individuals at a total cost of £15,022. With reported prevalence averaging 37.5% within this subpopulation, 194 individuals would be identified. This implies that the cost per case (assumed accurately) detected is £77. However, the estimated numbers of cases detected by screening the subpopulation 'born into poverty' yields a higher number than the total estimated using available general population prevalence data and, therefore, may indicate the level of uncertainty surrounding currently available prevalence figures. To validate whether or not such strategies are favourable, future studies may examine prevalence of disorganised attachment through general population studies and examine the marginal effect of poverty status on predicting cases of disorganised attachment (discussed further in *Chapter 8*). *Table 26* presents budget impact assessment of detection strategies to identify attachment disorders through general population screening.

Based on the four studies of identification strategies, three studies were used to estimate the average cost of identification procedure for a RAD as £109 (omitting Equit and colleagues[101] given the length of procedure reported indicates a full psychiatric assessment and not a specific assessment of an attachment disorder). Given that prevalence is found to be 1.4% in the general population, this would suggest that screening a general population sample of an average CCG would identify 45 cases. This would suggest that the mean cost per case (assumed accurately) detected is £7841.

Several limitations of this budget impact assessment need to be acknowledged and considered in further research priorities so that future estimates of cost-effectiveness can be estimated more accurately. Specifically, the assumption that:

i. All tests are equal and have perfect precision is unlikely to hold and requires (a) more accurate definition of severe attachment problem classification defining a gold standard test, and (b) by severe attachment problem classification, that diagnostic performance be assessed versus the gold standard.
ii. Underlying estimates of prevalence would be expected to be variable given unobservable factors (e.g. comprehensive risk profile, family size, temporal factors) and future research should make better use of advanced statistical methods to explain prevalence controlling for these various potential significant variables.
iii. In real-world settings, not everyone who is at risk may be eligible for screening.
iv. Overall, the quantity and quality of data are limited and therefore estimations of budget impact are primarily illustrative to provide an iterative basis to update from the currently available information.

© Queen's Printer and Controller of HMSO 2015. This work was produced by Wright *et al.* under the terms of a commissioning contract issued by the Secretary of State for Health. This issue may be freely reproduced for the purposes of private research and study and extracts (or indeed, the full report) may be included in professional journals provided that suitable acknowledgement is made and the reproduction is not associated with any form of advertising. Applications for commercial reproduction should be addressed to: NIHR Journals Library, National Institute for Health Research, Evaluation, Trials and Studies Coordinating Centre, Alpha House, University of Southampton Science Park, Southampton SO16 7NS, UK.

TABLE 26 Budget impact assessment of identification strategies to detect attachment disorders

Disorder type	Target population (percentage of general population)	Expected number to screen (per average CCG)	Mean cost of assessment/diagnostic procedure (min.–max.), £	Total cost to CCG, £	Prevalence of disorder	Number of expected diagnosed	Mean cost per diagnoses (min.–max.), £
RAD	General (100%)	3237	109 (90–120)	352,833	1.4%	45	7841 (6429–8571)

Max., maximum; min., minimum.

Budget impact assessment of providing treatment strategies

The interventions for disorganised patterns of attachment vary in their different resource intensities by interventions types from four sessions of less than 1 hour with a mental health nurse, to weekly sessions with a psychologist for 1 year. Treatment programme costs range from £544 to £5226 and, on average, the cost is £2265 for a full course of treatment. Based on the assumption that the SSP perfectly predicts all cases of disorganised attachment, the estimated total cost to the average sized CCG is calculated.

With the number of cases of disorganised attachment patterns expected to range from three (screening alternative caregiver) to 207 (screening those born into poverty), the total costs of treatment strategies vary between £6887 and £469,377. In the absence of reported sensitivity or specificity of any assessment tool used in place of the SSP for disorganised patterns of attachment, these estimates do not account for the potential additional costs of treating individuals who screen false positive and the cost reduction due to not treating false negatives.

Only one study was identified aiming to treat RAD.[187] Given that 45 cases of RAD are expected across a CCG, the expected budget implication of this treatment strategy is £102,660 (again not taking into account costs associated with false positives and negatives).

Budget impact assessment of implementing severe attachment problem programmes with clinical commissioning groups

Rolling out an interventions programme for severe attachment problems can be implemented either at a general population level or in more specific target groups. *Table 27* brings together the expected costs of identification and intervention of disorganised patterns of attachment.

For a general population programme for disorganised patterns of attachment in the average CCG (with a population size of 264,039 individuals), the cost of identification is £93,873 per year and subsequent treatment would cost, on average, £219,987, implying that the total cost to screen the general population and change disorganised patterns of attachment would approximate to £313,860 per year.

To justify this level of expenditure to satisfy the explicit decision rules of cost-effectiveness specified by NICE, screening and treatment of disorganised patterns of attachment would need to demonstrate an incremental cost per QALY threshold of between £20,000 and £30,000 (accepting these unlikely assumptions regarding treatment as usual) and identification plus treatment would need to produce between 0.1995 and 0.2993 QALYs over the lifetime of the child.

A budget impact assessment of CCGs strategically screening to subsequently treat RAD (the only available example for attachment disorders) indicates a total cost £455,493 to the CCG budget (*Table 28*).

TABLE 27 Budget impact assessment of the cost of treating disorganised patterns of attachment by target populations

		Budget implication required for treatment		
**Disorder type**	**Target population**	**Cost of identification, £**	**Cost of subsequent treatment, £**	**Total cost, £**
Disorganised pattern of attachment	General	93,873	219,987	313,860
Disorganised pattern of attachment	Middle class	23,461	238,245	261,706
Disorganised pattern of attachment	Born into poverty	15,022	469,377	484,399
Disorganised pattern of attachment	Alternative caregiver	551	6887	7438
Disorganised pattern of attachment	Maltreatment	406	15,223	15,629

© Queen's Printer and Controller of HMSO 2015. This work was produced by Wright *et al.* under the terms of a commissioning contract issued by the Secretary of State for Health. This issue may be freely reproduced for the purposes of private research and study and extracts (or indeed, the full report) may be included in professional journals provided that suitable acknowledgement is made and the reproduction is not associated with any form of advertising. Applications for commercial reproduction should be addressed to: NIHR Journals Library, National Institute for Health Research, Evaluation, Trials and Studies Coordinating Centre, Alpha House, University of Southampton Science Park, Southampton SO16 7NS, UK.

TABLE 28 Budget impact assessment of the cost of treating RAD in general populations

		Budget implication required for treatment		
Disorder type	Target population	Cost of identification, £	Cost of treatment, £	Total cost, £
RAD	General	352,833	102,660	455,493

The findings of this budget impact analysis should be interpreted with caution given the unrealistic assumptions made in the absence of good-quality evidence. There exist substantial heterogeneity issues underlying estimates of prevalence, intervention effect and the number of cases accurately detected through screening. The following points should be considered in light of this assessment and in consideration of future research:

i. The QALY gain, while substantial, may be accrued over a lifetime (given that the programme would aim to potentially target infants). However, to make fair comparisons of the costs and benefits of different health-care programmes, conventionally these will be discounted over time at a rate of between 3% and 6%. *Time preference* is the economic theory underlying this procedure and is done to reflect individuals' preference to spend money on goods they will receive now as opposed to in the future. In applications to future benefits of intervention for attachment, the conventional discount rate will imply that benefit becomes negligible if extrapolated too far into the future (e.g. over 17 years if the applied time discount rate were 3%).
ii. The estimated budget impact of the identification strategy presented here is based on the (optimistic and unrealistic) best-case scenario in the absence of assessment tool/diagnostic test accuracy and effectiveness data. To highlight the potential implications, consider the hypothetical (optimistic) notion that the test had a specificity of 0.95 with sensitivity of 1; this would imply that 5% of the 3140 newborns unlikely to have disorganised patterns of attachment would receive treatment unnecessarily. For the general population assessment previously presented, treating an additional 5% of the screened population inappropriately as a result of the rate of false positives would increase the budget impact to a CCG by £355,644, thereby increasing the cost per disorganised pattern averted to £12,767 and requiring further health gains to justify the investment.
iii. The figures utilised to assess the budget impact do not currently include any potential cost offset as a result of an improved future prognosis and reduced service use. By averting future health-care needs, cost-consequences may be offset against the initial programme costs.
iv. Adopting a wider societal perspective, wider cost offset may have budget relevance to the national exchequer. This may include impact on education, criminal justice and productivity within the wider economy. One important example found in the evidence base showed that treatment of RAD (as opposed to disorganised pattern, discussed above), has a positive cost consequence by reducing demand for foster care by appropriately treating the attachment disorder;[187] although this observed change was not found to be statistically significant, it is an example of a good methodological approach in this context.

Conclusions

A decision model provides health service decision-makers with a tool aggregating relevant data to assess how resources directed at identification and intervention in severe attachment problems improve health-related quality of life. The systematic review of severe attachment problems reveals a vast research literature; however, applying the inclusion criteria to address the decision uncertainty reveals a paucity of relevant literature to reliably inform policy. Various fundamental issues remain to be addressed to ensure limited health-care resources are efficiently utilised to address severe attachment problems within the context of child mental health services, other early intervention services and also the wider health-care budget.

A clear definition of attachment problems is required to ensure that appropriate individuals are targeted. At the present time, it is unclear whether this should focus on attachment disorders and/or attachment patterns (e.g. the disorganised attachment pattern). In the absence of more robust literature at the current time, this health-care market cannot be properly assessed.

The population within which to seek out relevant cases of severe attachment problems requires further consideration. Our provisional budget impact analysis would suggest that screening of the general population followed by intervention must be highly effective in producing health gains to justify the required expenditure. Screening at-risk cohorts may produce a more favourable budget impact; however, substantial numbers of cases may inadvertently be missed where 'risk' remains unclearly defined (further research is required).

The prevalence of severe attachment problems varies widely and identified prevalence studies are from a variety of countries. Further, country-specific research is required and future research should aim to control for confounding factors, which may be influential over prevalence estimates.

The attachment literature has an absence of research discussing assessment tool/diagnostic test accuracy as a result of the lack of consensus on gold standard measures (in the patterns research literature) or impending current change (for attachment disorders). Diagnostic/assessment practice in severe attachment problems can, therefore, be considered broadly unregulated, as the relative benefits of various procedures are unknown. Further research and consensus building is required.

The literature would indicate that the majority of the research efforts have focused on developing interventions and, as such, a variety of parental interventions exist. The resource intensity of parental interventions also varies widely and relatively little evidence would suggest whether or not variance in this intensity has a relationship to outcomes. Meta-analysis conducted in this review indicates that, on aggregate, those receiving intervention for disorganised attachment have 54% reduction in odds of displaying a disorganised pattern of attachment post treatment (short term) than those not treated. Only one study[189] had a follow-up of 12 months or longer (non-significant in the subgroup meta-analysis). However, if we can assume that the post-intervention effect observed in the overall meta-analysis might be sustained, the budget impact assessment presented here suggests that this change needs to equate to between 0.1995 and 0.2993 QALYs to justify the required expenditure.

To achieve this expected QALY health gain, the benefits of programmes for severe attachment problems would most likely have to be realised over a number of years. Research to date that has been reviewed to forecast these benefits is largely inconclusive on the causal links between severe attachment problems and future risk of psychopathology and/or developmental sequelae. Despite this deficit in empirical evidence, it cannot be concluded that the absence of evidence to inform cost-effectiveness is an evidence of absence of cost-effectiveness.

© Queen's Printer and Controller of HMSO 2015. This work was produced by Wright *et al.* under the terms of a commissioning contract issued by the Secretary of State for Health. This issue may be freely reproduced for the purposes of private research and study and extracts (or indeed, the full report) may be included in professional journals provided that suitable acknowledgement is made and the reproduction is not associated with any form of advertising. Applications for commercial reproduction should be addressed to: NIHR Journals Library, National Institute for Health Research, Evaluation, Trials and Studies Coordinating Centre, Alpha House, University of Southampton Science Park, Southampton SO16 7NS, UK.

Chapter 8 Research priorities and value of information analysis

In this chapter we draw on the evidence from our systematic reviews exploring the various clinical aspects of severe attachment problems, including our main clinical effectiveness and cost-effectiveness review and our supplementary reviews exploring early assessment tools and outcomes of 10 years or more. We discuss the gaps or limitations highlighted in the review and also discuss attempts to develop a decision model. The available literature was not sufficient to populate a decision model framework (see *Table 24*). However, by summarising the identified gaps to inform the decision model framework, specific gaps in the evidence base are discussed in conjunction with the broader findings of the systematic reviews. The following sections highlight the evidence gaps identified in these ways. This, with PPI and expert input, then informs the recommendations for future research priorities relevant to both clinicians and policy-makers.

Despite a vast literature in the field of attachment, there are some important gaps when we examine the literature systematically. There are a large number of short-term and uncontrolled studies and a wealth of qualitative and theoretical papers. There is a relative lack of clinical trials and long-term follow-up studies concerning severe attachment problems. The following sections outline the gaps and limitations that were discovered through the course of this work.

Gaps and limitations identified

Consensus on meaning of severe attachment problems

In order to be able to detect severe attachment problems it is necessary to be clear about what severe attachment problems are and how they are defined. There is no one entity which can be meaningfully called severe attachment problems. For the purposes of this review, it was decided to include disorganised attachment patterns and attachment disorders under this overall term. The systematic review showed that the literature includes many different coding systems and assessments. *Box 1* shows that when methods of assessing attachment patterns were compared, we found a number of papers using 30 different sets of nomenclature.

Furthermore, attachment disorder diagnostic criteria have recently been changed by the APA[56] and are currently being changed by the WHO.[55]

Most studies that we found in our review that sought to compare attachment instruments did not include raw data. There is also a sparse literature on the relationship between the presence of disorganised patterns of attachment in infancy and the later incidence of attachment disorders. Indeed, it is not clear if a progression from one to the other is to be expected.

The PPI group suggested that difficulties in achieving consensus may be hampering more generalised helpful research into prevalence and subsequent developmental sequelae and long-term outcomes.

The use of disorganised attachment patterns and attachment disorders was agreed to be a helpful way of identifying severe attachment problems for practice and further study at the current time.

Measures and identification of children with severe attachment problems

Once we have clarification of definitions, validated assessment tools are necessary for use in clinical practice.

© Queen's Printer and Controller of HMSO 2015. This work was produced by Wright *et al.* under the terms of a commissioning contract issued by the Secretary of State for Health. This issue may be freely reproduced for the purposes of private research and study and extracts (or indeed, the full report) may be included in professional journals provided that suitable acknowledgement is made and the reproduction is not associated with any form of advertising. Applications for commercial reproduction should be addressed to: NIHR Journals Library, National Institute for Health Research, Evaluation, Trials and Studies Coordinating Centre, Alpha House, University of Southampton Science Park, Southampton SO16 7NS, UK.

For developmental reasons, different assessment tools are necessary at different ages during childhood. When conducting our reviews many of the different mechanisms for naming or assessing attachment patterns had not been validated against the reference standard (the SSP) or against other instruments. There is very limited good-quality validation research and there were only two concurrent validity studies for measures with a disorganised attachment pattern comparing with any other instrument.

There was one concurrent validity study for DAD, but this was in a Romanian population who had lived in institutions.[95]

Only one study[95] concurrently compared disorganised attachment patterns with attachment disorders, suggesting little correlation.

The QUADAS-2 was used to evaluate the quality of the assessment tools for patterns and disorders, and the results of this are shown in *Table 5*. The risk of bias for the included studies was rated as unclear or high for many of the studies across most of the bias domains, with the exception of the reference standard domain.

The frequency with which an unclear rating was used suggests that future studies in the area should more clearly report key methodological features that are likely sources of bias. Gaps included a lack of clarity about patient selection (e.g. being consecutively recruited) and a lack of blinded ratings. In addition, there was often limited clarity in statements about whether or not the index test and the reference standard were interpreted blind to each other, the length of time between the administration of the index and reference test, and a clear description of the flow of participants through the study.

Lack of consensus in bodies of literature for both patterns of attachment (measured in infancy) and the diagnosis of attachment disorder (measured in childhood) means that the gold standard approach is not standardised for either attachment patterns or attachment disorders. The task for both clinicians trying to make sense of the literature, and decision-makers considering allocation of scarce resources, would be rendered easier if the broadly expressed notion of 'attachment difficulties' is not used. Instead, disorganised attachment patterns and attachment disorders should be considered separately until such time that any clear link between them is found through further research evidence relating to each of these defined constructs.

Information on severe attachment problems within specific subpopulations

The existing attachment literature commonly studies phenomena in samples taken from subpopulations (e.g. the Minnesota study was based on children born into poverty[244]). There is a range of literature in this field. There is evidence from meta-analyses both that attachment behaviours are universally identifiable cross-culturally and that there are culturally determined influences,[46,245] such as, for example, differences 'in the expression of maternal sensitivity and the manifestations of secure-base behaviour' (p. 81).[87]

Some subpopulations were under-represented in this review (e.g. maltreated children). UK-specific research needs to be clearly defined for these reasons and also in informing UK policy.

These different subpopulations with differing rates of disorganised patterns of attachment may have implications in numerous ways, both clinically and for decision-makers, for example in areas such as cost of identification, clinical workloads, training needs, attrition rates, ethics, resource utilisation, clinical effectiveness and cost-effectiveness.

Prevalence of severe attachment problems

Our supplementary review of outcomes of 10 years or more identified rates of severe attachment problems and we were able to draw from previous meta-analyses and research for wider prevalence figures. More work on the UK populations and subpopulations (both culturally and in at-risk groups) would generate more evidence.

Long-term health outcomes

The relationship between early development of severe attachment problems – either disorganised attachment patterns or attachment disorders – and poor developmental outcomes is commonly cited in the attachment literature.[246] However, research exploring outcomes of disorganised attachment patterns or attachment disorders of 10 years or over is very limited. Although we found studies exploring these attachment patterns, we found no studies with 10-year follow-up or longer that diagnosed an attachment disorder at baseline.

Although eight studies demonstrated long-term outcomes of disorganised attachment patterns in infancy that found associations with psychopathology in adolescence and young adulthood (e.g. the Minnesota study[244]), the study exploring psychopathology used a global measure of psychopathology and published no results looking at this in more detail or looking at specific disorders. With only the Minnesota study including psychopathology, no meta-analysis was possible. There were no high-quality long-term studies looking at other important outcomes such as educational attainment, criminality and productivity.

Having found limited evidence for 10-year outcomes, a supplementary scope has been carried out, exploring 5- to 10-year follow-up of children under 13 years of age who had a diagnosis of attachment disorder or a disorganised attachment pattern at baseline. This can be found in *Appendix 4*. Although this is not part of the systematic review, it shows mixed findings. For example, infant disorganised attachment predicts behaviour problems in preschool but not in school-aged children over several school years.[45] Some studies find an association with infant disorganised attachment and teacher-rated behaviour problems at aged 7 years for boys but not girls.[247] These findings are mirrored in Fearon and colleagues' meta-analysis of attachment patterns at infancy and of subsequent externalising behaviour measured anything up to the age of 12 years.[248] These show, for example, a combined effect size of $d = 0.34$ ($p < 0.05$) that is recomputed as $d = 0.18$ (95% CI 0.01 to 0.34) when 8 of 34 studies have data trimmed and filled to account for publication bias. As with the study by Hazen and colleagues,[247] when genders are examined separately there is a negative association ($d = -0.20$) for girls between infant externalising problems and subsequent behaviour problems.

Intervention effects

Although there are numerous interventions that are described in the literature seeking to improve attachment patterns (see *Chapter 6*), only one study included in our review examined the clinical effectiveness of interventions for children with attachment disorders.[187,188] This demonstrates that there are very few RCTs of parenting interventions for attachment disorders. The PPI group noted that many widely used therapies in the UK (e.g. Theraplay®) did not have RCTs that met the quality criteria for our review. Given that attachment disorders may be defined as extending to relationships with non-caregiving attachment figures, children with attachment disorders might benefit from direct work to help them in their wider social relationships, as well as work with caregivers; but this review not look for these.

Several studies demonstrated that parental interventions can improve attachment outcomes for children with disorganised attachment patterns. Eight intervention studies of disorganised attachment patterns were included in a meta-analysis indicating an OR of a post-treatment effect of 0.46 (95% CI 0.33 to 0.64; $p < 0.0001$).[189,190,193–196,198,200,218] These studies were mainly short-term outcome studies for reducing rates of disorganised patterns of attachment seeking to improve attachment, with limited reporting of mental health outcomes. Of the pool of eight studies, only one study[189] had a follow-up of 12 months or longer, and that one study showed no significant difference at follow-up. The existing literature has not established whether or not current evidenced treatment for disorganised patterns of attachment have any sustained effect over time.

The Cochrane risk of bias tool[174] was used to assess the quality of the clinical effectiveness studies. This revealed that a majority of items were rated as either unclear or high risk of bias. For example, it was common to have selective and incomplete outcome reporting and no Consolidated Standards of Reporting Trials (CONSORT) statement.

© Queen's Printer and Controller of HMSO 2015. This work was produced by Wright *et al.* under the terms of a commissioning contract issued by the Secretary of State for Health. This issue may be freely reproduced for the purposes of private research and study and extracts (or indeed, the full report) may be included in professional journals provided that suitable acknowledgement is made and the reproduction is not associated with any form of advertising. Applications for commercial reproduction should be addressed to: NIHR Journals Library, National Institute for Health Research, Evaluation, Trials and Studies Coordinating Centre, Alpha House, University of Southampton Science Park, Southampton SO16 7NS, UK.

Given that most of the included studies focused on maternal sensitivity, there is a gap in trials exploring interventions for children with high levels of complexity and comorbidity.

Resource and cost-effectiveness information

Despite many potential interventions, only two papers included an economic evaluation of interventions: one assessed an intervention for foster parents of children with attachment disorders[187,188] and the other assessed a parent group intervention called Right from the Start.[175] These did not include long-term health outcomes or developmental sequelae. Both studies deliver an economic analysis describing the cost-effectiveness of the intervention. Neither study attempted to extrapolate the potential benefits beyond their study or attempted to indicate the generic health gains from treatment of their respective problems (e.g. QALYs).

In describing the gaps in the research when considering the decision model, it is pertinent to consider a value of information analysis.

Value of information analysis and informing future research priorities

This review would have ideally liked to have undertaken a formal value of information analysis. Value of information[249] analysis attempts to evaluate the opportunity cost that arises from making an incorrect decision (such as choosing a suboptimal intervention strategy) because of inadequate current evidence. This would be useful in this field because many clinical units use interventions that have no RCTs to support their use at the current time. The value of information analysis informs whether or not future research is worthwhile (i.e. what is the potential 'payback' of expenditure on research?).

However, as discussed in previous chapters, the current evidence base to inform health economic decision-making relating to service provision for severe attachment problems is very limited. Therefore, the current evidence has not allowed for the development of a comprehensive decision model or the probabilistic framework to carry out a value of information analysis.

Description of future research priorities

The combination of the results of the systematic reviews, and particularly the gaps identified by the reviews and the attempt to construct a decision model, were considered by our PPI group and steering group experts. These led to a series of suggestions about research priorities.

Priority 1: recommendations for clarifying the meaning of 'severe attachment problems' and developing consensus on assessment tools to be used

Attachment patterns are different from attachment disorders. Within attachment patterns, disorganised attachment can be regarded as a severe attachment problem, as insecure categorisation has not been found to be useful predictively to identify with which children to intervene.[250,251] In order for clinicians to know which groups will benefit from intervention, there needs to be a reliable and valid way of identifying those groups. A consensus needs to be established about a limited number of validated assessment tools. Without this fundamental agreement, it is not possible to establish long-term outcomes, clinical effectiveness and cost-effectiveness studies or subsequent potential screening programmes.

We have reviewed studies that screen for and attempt to treat attachment disorders such as RAD/DAD. Our review has indicated that there is limited evidence around attachment disorders, indicating that this is a neglected area of research. Changes in RDC by DSM-V[58] and ICD-11[88] complicate this by revising definitions. As with disorganised attachment, a consensus needs to be established about a limited number

of validated assessment tools for RAD and the newly coined disinhibited social engagement disorder in DSM-V. At the current time, the DSM-V guidelines specify that the disturbance must be evident before the age of 5 years, which may limit research in older children. However, once new RDC are defined and established, they will form a good starting point for future identification and follow-up research. One example of a way to approach this is to use an international Delphi expert consensus.

Future studies should have sufficient information collected and reported to ensure that each item of the QUADAS-2[92] (or equivalent) can be assessed. They should report the performance of the screening measure at pre-determined time points to prevent the post-hoc selection of cut-off points, and so that future modelling can examine the effect of different balances between sensitivity and specificity for a particular assessment tool. Raw data should be included in reports so that future systematic reviews and meta-analyses can adequately and objectively assess and compare the literature. In addition, studies should report sufficient data to allow calculation of test accuracy data. Finally, in terms of reporting, studies should provide information such as the typical duration of administration, and the level of training required to deliver the test. This information would prove useful for the cost-effectiveness analysis [*see Priority 3: randomised controlled trials of interventions (clinical effectiveness and cost-effectiveness)*].

A progression has been noted from an early disorganised attachment to a compulsive caregiving or coercive controlling behavioural pattern.[147] Future trials should more closely examine the relationship between early attachment measures and subsequent attachment measures, as these need to be different at different ages.

Priority 2: recommendation for prevalence and long-term outcome studies

Only four studies that met our review criteria reported on outcomes of 10 years or more for infants with identified disorganised attachment patterns at inception[45,151–153] but with limited high-quality reporting of outcome data. There were no papers reporting on the outcomes of attachment disorders over this time course.

The research literature has not as yet elucidated whether disorganised attachment is a causal factor of later psychosocial difficulties or, as is more likely, a probabilistic indicator among a number of factors. Although the attachment literature commonly makes reference to a range of potential adverse associations with attachment disorders or disorganised attachment patterns, including entry into care, psychosocial development, educational attainment, adolescent psychopathology, adult psychopathology and adult criminality, we found little robust evidence to populate a decision model. There needs to be more scientific research to corroborate or refute the hypothesised associations against agreed measures of attachment at baseline.

There is a need for a thorough long-term cohort study to identify children with severe attachment problems in adequately powered samples (ideally using a gold standard method) that provides the basis to follow them up in the long term. The Minnesota study[245] and the National Institute for Child Health and Development (NICHD) study[252] are two good examples of cohorts that yield sequentially useful research. Long-term implications may be best observed through incorporating standardised measures into established longitudinal samples, such as birth cohort studies.

As we have shown, prevalence of disorganised attachment patterns goes up when children face adverse experiences. Any cohort research needs to carefully explore predisposing factors and genetic and environmental influences (e.g. from a systematic review that considers them) as they map onto attachment patterns over time.

Research of this nature will also add to the prevalence literature. The notion that the prevalence may be increased owing to features within subpopulations will require further research. The provisional budget impact analysis (see *Chapter 7*) suggests that strategies targeted at specific subpopulations may be of benefit. The prevalence parameter is important in evaluating the incremental costs and benefits of

© Queen's Printer and Controller of HMSO 2015. This work was produced by Wright *et al.* under the terms of a commissioning contract issued by the Secretary of State for Health. This issue may be freely reproduced for the purposes of private research and study and extracts (or indeed, the full report) may be included in professional journals provided that suitable acknowledgement is made and the reproduction is not associated with any form of advertising. Applications for commercial reproduction should be addressed to: NIHR Journals Library, National Institute for Health Research, Evaluation, Trials and Studies Coordinating Centre, Alpha House, University of Southampton Science Park, Southampton SO16 7NS, UK.

screening and intervention against usual care. The potential impact of uncertainty in this parameter was illustrated examining the implications of variation in the prevalence within the various subpopulations. As current prevalence of disorganised attachment estimates vary from 3%[155] (in the general population) to 37.5% (as indicated in studies of 'children born into poverty'), underlying prevalence is shown to affect the potential budget impact of related potential identification and treatment strategies. For a UK decision model to be better informed, future research needs to ensure that (1) identified prevalence is country specific (in this case, relevant to the UK); (2) population sampling relates to potential types of service demand; and (3) prevalence is estimated (ideally using a gold-standard tool) in sufficiently powered samples.

The integration of natural history findings into a large longer-term cohort in the UK would be of benefit. For example, it would allow consideration to be given to continuity and maintenance of attachment security, subsequent maternal sensitivity and later child development, as well as risk factors, mediators and moderators such as self perception,[253] emotion regulation,[254] parental well-being,[255] family social support,[256] parental conflict,[257] maltreatment[258] and other relevant factors.

Priority 3: randomised controlled trials of interventions (clinical effectiveness and cost-effectiveness)

Before consideration can be given to screening, we need to know whether or not interventions can alter outcomes. Screening is to be of value only if those identified as at risk can be offered an intervention that will reduce the risk of problematic outcomes.

Of the 30 studies[129,175–178,180–186,188,189,191,194–196,198,200–203,205–208,211,212,218] identified in *Chapter 6*, 17 interventions from 13 studies[129,133,180,189–191,193–200,202–204,208,209,218] were included in a meta-analysis to assess the intervention effect on secure attachment. Of these 17 interventions, eight studies[189,191,194–196,198,200] were included in a meta-analysis to assess the intervention effect on disorganised attachment. We found only one study that examined the clinical effectiveness of treatments for attachment disorders[187,188] and were unable to perform a meta-analysis as a result. Definitive RCTs would be required to demonstrate clinical effectiveness and cost-effectiveness in this group and provide a potential basis for extrapolating longer-term benefits of treatment.

When considering disorganised attachment patterns, there is interesting evidence of treatment effects when studies are targeting maternal sensitivity and its expression in mother–child interaction to change attachment patterns. While the paucity of alternative interventions might point to the need for research in this area, our PPI groups believe maternal sensitivity and mother–child interaction to be a fruitful area for continued research. Most parental intervention research has been conducted with the female caregiver and the most promising research in our meta-analysis is focused around the mother–child dyad. The primary caregiver may be male or female and, therefore, further work with male caregivers should be conducted. Although work with the male caregiver is sparse, it is also a complex area given the variety of different at-risk groups associated with severe attachment problems, ranging from supportive but vulnerable caregivers to abusive caregivers. There is not enough research in these different areas with female and/or male carers to comment further on this. Interpretation of the meta-analysis results suggests that prenatal interventions may be able to reduce disorganised attachment, possibly as a result of identifying at-risk families early. Further research here would be productive. Video-feedback research was promising, as was research that did not use video feedback. The PPI group was very positive about the use of constructive video feedback as a helpful tool. Further research could directly compare an intervention with or without video feedback using mixed methods including qualitative analysis and cost-effectiveness analysis. Some experts and PPI members also pointed out that, if early intervention had not been possible, or had not happened for any reason, we need to have treatment trials with older children. As some children with disorganised attachment patterns in infancy may progress on to compulsive caregiving and coercive controlling behaviours,[21,24] there needs to be more research, not only in understanding this progression, but also in the clinical effectiveness and cost-effectiveness of the interventions for these groups. In such studies there would need to be better understanding of aetiologies, including the relationship to earlier patterns of attachment and life experiences that would be helpful in the

testing of the effectiveness and cost-effectiveness of interventions for these groups of older children. Furthermore, the wealth of evidence on the effectiveness of maternal sensitivity work is encouraging, but is it likely to be a useful intervention in children with severe attachment problems with high levels of past traumas and comorbid behavioural and developmental problems? Additional therapeutic options directed at children with more complex problems and comorbidities should also be tested in RCTs.

To avoid bias, adequately powered RCTs using appropriate measures would be needed. The quality of trial design identified by our quality assessment tool demonstrates the need for improved quality of methodological design. This includes clear identification of children at baseline, a series of high-quality baseline measures, and taking steps to reduce bias, with clear prospective identification of outcome measures in health, mental health, education and social outcomes. There need to be multiple studies in order to reduce decision uncertainty. Future studies should also include clear CONSORT statements.

As far as cost-effectiveness is concerned, future research of severe attachment problems needs to acknowledge the scarcity of resources in the health system and that an opportunity cost will always exist when deciding where to allocate a limited health budget. All future research in the field needs to have good resource information identified in published work. There should, for example, be clear descriptions of intervention procedures detailing any personnel time required to perform a procedure and (where applicable) provide a comparison to resources required within routine care. Furthermore, as various procedures will have cost consequences for the health system, for related sectors or within the wider economy, future research should also include tools to measure changes in (1) health service utilisation, (2) non-health service use and (3) wider societal impact (e.g. criminality, levels of informal care or productivity). This information will be informative to improve future evaluation of cost-effectiveness (of identification or interventions). Through the inclusion of these forms of information in future research, emerging evidence will be equipped to inform both the clinical effectiveness and the cost-effectiveness of interventions for severe attachment problems.

The expert group pointed out that many CAMHS are stretched with limited resources, and that effective short-term interventions will be attractive compared with many of the long-term or intensive options currently being used. It is only by including resource usage and cost-effectiveness elements that we will be able to answer important resource allocation questions in order to plan service provision.

One key finding is that despite very large numbers of papers in the field of attachment there are very few that allow economic evaluation and none that have attempted to measure QALYs. This is a large gap that requires attention in future research. This means that studies that use instruments such as the European Quality of Life-5 Dimensions-Youth version (EQ-5D-Y),[259] the Health Utilities Index (HUI-2)[260] or instruments designed for use in child mental health settings, which enable the calculation of QALYs, would be helpful. There needs to be work that begins to better understand what any meaningful clinical gain means in terms of a QALY for this group.

To summarise, as there is a powerful need to develop clarity around interventions that can be used in robust clinical effectiveness and cost-effectiveness research, the focus on parental sensitivity within the dyad of the primary caregiver and child (for disorganised attachment patterns) would appear to be the most promising focus (with statistically significant improvements on meta-analysis) at the current time. There is a need for more good-quality RCTs of interventions treating children diagnosed with attachment disorders or those at high risk such as those in adopted or foster care.

© Queen's Printer and Controller of HMSO 2015. This work was produced by Wright *et al.* under the terms of a commissioning contract issued by the Secretary of State for Health. This issue may be freely reproduced for the purposes of private research and study and extracts (or indeed, the full report) may be included in professional journals provided that suitable acknowledgement is made and the reproduction is not associated with any form of advertising. Applications for commercial reproduction should be addressed to: NIHR Journals Library, National Institute for Health Research, Evaluation, Trials and Studies Coordinating Centre, Alpha House, University of Southampton Science Park, Southampton SO16 7NS, UK.

Summary

Our main systematic review and supplementary reviews provide insights that suggest a number of research priorities. A cohort study would address these gaps by allowing good methodological research with sequential attachment measures alongside gold standards and good baseline epidemiological and risk factor information. This would enable UK-based outcome research with outcomes across health, developmental, educational and social domains. It would also allow for embedded RCTs with robust elements of resource utilisation and cost-effectiveness to allow for the calculation of QALYs.

Alongside the important area of clinical effectiveness and cost-effectiveness (priority 3), we need to know which children require interventions and when. For this to be clearer, future research could usefully focus on the natural history in terms of long-term outcomes (priority 2).

Research communities and clinical networks need to be clear about what is being measured and find a common way of measuring it. There is a need to adopt the most methodologically robust assessment tools (from a large pool of available and unvalidated instruments) to identify previously unidentified severe attachment problems. As noted by our PPI and expert groups, current attachment disorders classification systems are undergoing change, and the existing coding identified for attachment patterns (see *Box 1*) shows multiple different approaches to nomenclature and subcategorisation. Once we have clarity of nomenclature, definition and assessment, it will be a more straightforward task to follow up different at-risk populations and better understand the mid- to long-term consequences. At the current time there is very little evidence for long-term outcomes (10 years or more) of attachment disorders. Once we understand outcomes, we can carry out methodologically robust trials of interventions that permit high-quality clinical effectiveness and cost-effectiveness analysis alongside the calculation of QALYs. It will be appropriate to answer these more fundamental research questions before trials of screening are undertaken. Carrying out high-quality research in these fundamental areas will allow for the development of decision modelling, which in turn will lead to improved policy decisions.

Chapter 9 Discussion and conclusion

Statement of principal findings

The principal findings are as follows.

Objective 1

To identify the range of intervention programmes that are designed for parents of children with severe attachment problems.

Thirty-nine papers documenting 30 intervention studies were found in our main systematic review. Only one study included children with an attachment disorder.[187,188] These interventions included a variety of techniques for enhancing a secure attachment pattern or changing a pattern of disorganised attachment and are discussed in *Chapter 6*.

Objective 2

To examine the clinical effectiveness of intervention programmes designed for parents of children with severe attachment problems.

In a meta-analysis of eight interventions (12 papers),[189–191,193–200,218] we found an overall statistically significant effect size for reducing disorganised attachment patterns. The main focus of this work was improving maternal sensitivity. A meta-analysis of 17 interventions across 13 studies (19 papers)[129,133,180,189–191,193–200,202–204,208,209,218] showed a statistically significant overall effect size in secure attachment (see *Appendix 6*). All of these studies were measuring attachment patterns over the short term (mainly less than 1 year). There were limited data describing secondary outcomes using validated instruments, and a meta-analysis examining these outcomes was not possible.

Objective 3

To examine the cost-effectiveness of intervention programmes designed for parents of children with severe attachment problems.

Only two clinical trials included an economic evaluation of the interventions. One assessed an intervention for foster parents of children with attachment disorders[187,188] and one assessed a parent group intervention called Right from the Start.[175] Both studies delivered an economic analysis describing the cost-effectiveness of the intervention. Neither study attempted to extrapolate the potential benefits of treatment beyond their study. No attempts were made to indicate the generic health gains or health utilities from treatment of their respective problems.

Objective 4

To identify research priorities for developing future intervention programmes for children with severe attachment problems, from the perspective of the UK NHS.

From researching these various and vast areas of attachment literature we have suggested some future research priorities based on the gaps found by the current review (see *Chapter 8*).

Objective 5 (supplementary review 1)

To review the methods of assessment and/or diagnosis of attachment problems and/or disorders.

The review identified 33 studies[24,25,47,50,52,93–122] that examined the development of an assessment tool for attachment patterns ($n = 27$) or a diagnostic tool for attachment disorders ($n = 8$).

© Queen's Printer and Controller of HMSO 2015. This work was produced by Wright *et al.* under the terms of a commissioning contract issued by the Secretary of State for Health. This issue may be freely reproduced for the purposes of private research and study and extracts (or indeed, the full report) may be included in professional journals provided that suitable acknowledgement is made and the reproduction is not associated with any form of advertising. Applications for commercial reproduction should be addressed to: NIHR Journals Library, National Institute for Health Research, Evaluation, Trials and Studies Coordinating Centre, Alpha House, University of Southampton Science Park, Southampton SO16 7NS, UK.

There was one concurrent validity study for DAD, in a Romanian population,[95] showing good concurrent validity for the DAI.

We examined the relationship between the best known and most widely used reference standard, the SSP, and numerous other instruments. We found a diverse nomenclature for categories of attachment patterns. Several different assessment tools for disorganised attachment patterns were identified. Two studies that carried out concurrent validity for the strange situation procedure found good sensitivity but poor specificity.

Objective 6 (supplementary review 2)

To examine the 10 years or more outcomes among children with severe attachment problems and collect prevalence information from these studies.

Eight papers were found that measured a disorganised pattern of attachment at baseline (no studies diagnosed an attachment disorder at baseline).[189,191,194–196,198,200,218] These were linked to outcomes at a follow-up of 10 or more years later to inform health economics analysis and to supplement existing information from systematic review about short-term outcomes.[248] Given that our main review contained short-term outcomes, we also chose to widen the focus to long-term outcome information to help inform health economics models. Our expert/PPI group agreed a 10-year follow-up to ensure that infants reached the age of 10 years or older. To supplement the previous meta-analysis findings that include some studies with short-term outcomes of attachment,[23,248] we limited this review to studies that had conducted a measure of attachment containing a disorganised attachment pattern or a diagnosis of attachment disorders at baseline.

We found one study suggesting an association between disorganised attachment patterns at baseline and overall psychopathology rating using a validated semistructured questionnaire at the age of 17 years.[45] This used a global rating scale and did not specify types of psychopathology. There was also a weak association with borderline personality disorder in young adults,[153] which lost its significance when included with maternal hostility and other factors in a regression analysis.

Limitations

The results of the current review should be interpreted in the light of its limitations.

Any systematic review is limited by its own boundaries. The vast literature in this area means that we needed to put in place clear, high-quality criteria. This is a strength and a weakness. It allows only high-quality research to be included but excludes research that may have a contribution to make in a narrative of the wider literature. However, this latter literature is so vast that it makes high-quality review standards essential to ensure that the conclusions of the smaller number of high-quality studies are not lost among a much larger, but potentially more limited, literature.

Our review, and particularly elements of the first supplementary review (see *Chapter 4*), was limited by differences in the classification systems used which created problems in consolidating the results, performing meta-analyses and choosing, with any degree of certainty, which baseline characteristics were worthy of follow-up. We chose disorganised attachment patterns and attachment disorders (defined in *Chapter 1*), as most closely approximating the notion of severe attachment problems, in discussion with our experts and PPI groups, and as having the best evidence on which to base a systematic review at that point. As this evidence is limited, it remains to be seen in future work whether or not other options emerge as being more promising.

It is important to bear in mind that examination of attachment across the age span must take particular account of three important aspects. First, the type of assessment which is appropriate to assess attachment patterns varies with age: in infancy and young children the assessment focuses on the behaviour; in middle

childhood the focus changes to assessments of representations/internal working models; and in adolescence and adulthood the focus of the assessment is coherence. These different types of assessment require different assessments tools and with them, to some extent, different classifications (although the established secure, insecure avoidant, insecure resistant/ambivalent and disorganised patterns underlie many of the classifications). Second, consistency or stability of attachment pattern over time is not always expected, as a change in the child's caregiving experience may bring about a change in attachment pattern. Systematic reviews will have great difficulty in discerning the relative influences of developmental age and experiences (e.g. a change in the caregiver) from variation produced by definition or measurement. Third, disorganisation may continue and be identified by assessing representations/internal working models. However, the behavioural manifestations in older children may progress to other patterns (e.g. the proposed coercive controlling or compulsive caregiving patterns).[21,24] We coped with this by focusing on concurrent validity when two measures were conducted at the same time and looking for good-quality predictive validity studies, and this represents a limitation. Considerably more work in this area is needed, and some of this may allow for pathways of continuity to be better understood by means of such comparative work. Longitudinal studies with multiple measures may also help.

Choosing a 10-year follow-up limited the number of studies regarding attachment disorder outcomes in our second supplementary review. The choice of 10 years allows for a good understanding of longer-term outcomes, but may have restricted an understanding of important outcomes only discernible across shorter time scales. Other reviewers have explored these shorter-term outcomes,[147,248] and this was not the main focus of our work. It is our opinion that the very large natural history literature warrants a systematic review of its own given the time and financial resource limitations to any given piece of work.

There are some long-term follow-up studies that have produced a number of papers that do not meet our inclusion criteria for the supplementary review for a number of reasons. Some of these are listed in *Appendix 5*. Reasons include the fact that the paper might have described measuring disorganised attachment at inception but not reported it when exploring outcomes, or that the researchers either did not include a disorganised attachment assessment at baseline or reported any relevant outcomes of 10 years or more.

There is some evidence from the research that attachment patterns may influence whether or not children go on to have behaviour disorders.[153,248] There is a vast literature on attention deficit hyperactivity disorder,[259] oppositional defiant disorder and conduct disorder[262] that includes a number of parental intervention studies.[263,264] These would have been included in our systematic review had attachment measures been used. The vast majority of these studies do not include attachment measures. They measure behaviours of conduct or emotions, or other factors/variables.

We found that ethnic minority groups are under-represented in reviewed papers, with most studies having taken place in the USA. Van IJzendoorn and colleagues[46] found that attachment is a cross-cultural concept with 'cross cultural validity',[265] and so we have included attachment work from around the world. There are, however, some cultural differences,[46] and so this review needs to be treated with caution when we are analysing results for a multicultural UK population. Furthermore, policy decisions in different parts of the world may have a large impact on attachment; for example, fostering and adoption policies vary greatly between countries.[98]

The main review was limited by the available evidence and the boundaries of the review. There are likely to be a wealth of interventions that have never been tested in a RCT. Most of the interventions that we identified in our review related to maternal sensitivity delivered in the home or in a clinic. Some institutionalised[219] children with highly complex presentations are likely to need considerably more complex interventions. These will need to be the subject of RCTs. The fact that we are exploring parenting interventions naturally excludes organisational or systemic interventions. While this is a limitation, it does provide a clear focus for this review.

© Queen's Printer and Controller of HMSO 2015. This work was produced by Wright *et al.* under the terms of a commissioning contract issued by the Secretary of State for Health. This issue may be freely reproduced for the purposes of private research and study and extracts (or indeed, the full report) may be included in professional journals provided that suitable acknowledgement is made and the reproduction is not associated with any form of advertising. Applications for commercial reproduction should be addressed to: NIHR Journals Library, National Institute for Health Research, Evaluation, Trials and Studies Coordinating Centre, Alpha House, University of Southampton Science Park, Southampton SO16 7NS, UK.

The commissioned review focused on 'severe attachment problems'. What constituted this group was discussed extensively at our steering group meetings. We originally intended to review the insecure attachment group, but recent comprehensive evidence shows that insecure attachment occurs at about 35% in the general population.[87] The steering committee took the view that this could not be considered at the severe end of the attachment problems scale. Disorganised attachment, which occurs at much lower rates, and attachment disorders both had some evidence of subsequent psychopathology, and the steering group, including experts and PPI representatives, agreed that severe attachment problems should be defined as an umbrella term encompassing both attachment disorders (from either WHO or APA) and disorganised attachment patterns on testing. This would not be considered a very small number, as disorganised patterns of attachment occur at a level of approximately 3%[155] in the general population and 37%[152] in an at-risk population (e.g. high levels of poverty) which would be likely to be typical of a target group for interventions.

Systematic reviews with meta-analyses are often employed in the context of disorders or diseases. Attachment disorder is one such categorically defined disorder. Attachment patterns, however, are not diseases or disorders, and there is a question about whether or not this methodology is an appropriate one. On the one hand, conducting a systematic review leaves to one side some important qualitative studies, but, on the other hand, it allows us to explore studies with clearly defined methodological parameters.

Implications for practice

This review shows good evidence for interventions in infants within high-risk groups who have or may be vulnerable to severe attachment problems. These groups include parents with low SES, homelessness, adolescent mothers, mothers with mental health problems, children with low birthweight, children in foster placements, adopted infants and children with high levels of reported infant irritability. It suggests that interventions focusing on maternal sensitivity are able to achieve significant change and work well if applied early in the infant's life. Several different programmes that include maternal sensitivity work were identified by our review and included the COS-4 programme,[196] the ABC programme[193,194] and other research that has used video feedback work.[195,218] Maternal sensitivity programmes not using video feedback were also identified, such as TPP,[200] the UCLA FDP intervention[190,191] and other research carrying out maternal sensitivity work without video feedback during home visits.[189,197,198]

Parenting work with the foster carers of children with attachment disorders has also been fruitful.[187,188]

The review suggests that having a disorganised attachment pattern as identified by Main and Solomon[44] in these high-risk groups of infants represents a useful predictor of later psychopathology.[147]

The Department of Health's HCP (previously named the Child Health Promotion Programme or CHPP)[71,266] seeks to adopt an integrated approach to support for children and families[61] and states that effective implementation of the HCP should lead to 'strong parent–child attachment and positive parenting, resulting in better social and emotional well-being among children'. The goal to 'enhance the life chances for young children growing up in disadvantaged neighbourhoods'[228] holds healthy attachments as important in this endeavour.[229] Another implication for practice, therefore, is to find better ways of bringing attachment into the evaluation process,[230] as while attachment is a valued concept that is cited frequently in policy documents,[69,82] it is not always identified specifically in lists of 'strong predictors' of children's life chances.[82] Integrated working between practitioners, policy-makers and researchers is likely to be productive.

Recent funding for the children and young people's IAPT initiative includes attachment theory in the clinical training curriculum and parenting work for parents of 3- to 10-year-old children with oppositional defiant disorder and conduct disorder.[77] However, the management of behaviour problems has a different

focus from increasing caregiver sensitivity. Evaluation of these elements will prove fruitful from an attachment perspective. The independent PPI group discussed these initiatives in the light of our attachment review with a strongly held collective view about the importance of continuing to develop the policy and practice of early intervention work. There was some discussion about how better to identify and support vulnerable families prenatally or shortly after birth, and a belief that interventions early in childhood should continue to receive robust support.

Implications for research

The limited evidence we have identified suggests that there are a number of areas of uncertainty and a need for future research to reduce this uncertainty. Full details of the recommended research priorities were given in *Chapter 8*; the main points are summarised here.

Recent changes in definitions of attachment disorders need to be understood by the clinical and research community. There are a limited number of well-validated assessment tools for disorganised patterns of attachment and attachment disorders, and further work is needed to improve this situation. Better agreement of existing tools and nomenclature is required, possibly using a Delphi consensus. This fundamental work on consistency and validity of nomenclature, identification and assessment will become a bedrock on which good-quality outcome and intervention research can thrive.

More good-quality long-term studies are needed to look at children with severe attachment problems and explore outcomes including child and adult psychopathology, educational outcomes, criminal outcomes and future health and social care usage. This should include continued research to identify populations at greatest risk of poor outcomes. Further clarity is required regarding the relationship between early attachment problems and later psychosocial difficulties, specifically where the relationship is causal, or an association is based on common causal factors. A large cohort would allow this to happen in a UK context with multiple and sequential assessment and outcome measures.

Randomised controlled trials of intervention research, including cost-effectiveness, are required for attachment disorders. Further research using RCTs to reduce disorganised attachment patterns needs to take place to build on the existing literature. This needs to be of higher quality (including clarity of identification and high-quality follow-up, with better and more broadly based outcome measurement and with cost-effectiveness). Intervention RCTs that seek to prevent poor outcomes should include economic evaluation to permit the calculation of QALYs.

© Queen's Printer and Controller of HMSO 2015. This work was produced by Wright *et al.* under the terms of a commissioning contract issued by the Secretary of State for Health. This issue may be freely reproduced for the purposes of private research and study and extracts (or indeed, the full report) may be included in professional journals provided that suitable acknowledgement is made and the reproduction is not associated with any form of advertising. Applications for commercial reproduction should be addressed to: NIHR Journals Library, National Institute for Health Research, Evaluation, Trials and Studies Coordinating Centre, Alpha House, University of Southampton Science Park, Southampton SO16 7NS, UK.

Acknowledgements

Contributions of authors

Barry Wright had overall responsibility for the project, chaired the steering group meetings, supervised the trial co-ordinators and took primary responsibility for the drafting of the report.

Dominic Trépel, **Shehzad Ali** and **Stephen Palmer** contributed to all aspects of the economics sections. **Dominic Trépel** took primary responsibility for the drafting of the economic chapters.

Victoria Allgar contributed to all aspects of the statistical analysis, with support on *Chapter 4* from **Laura Manea**.

Lucy Cottrill, **Melissa Barry** and **Ellen Hughes** were sequentially trial co-ordinators for this study.

Steven Duffy (information specialist) and **Julie Glanville** (information specialist) contributed to all aspects of the search strategy. **Steven Duffy** carried out the literature searches.

Jenny Fell and **Lisa Hackney** contributed to the screening, data extraction and drafting of all systematic reviews.

Danya Glaser and **Vivien Prior** provided expert attachment advice throughout the project and contributed to drafts of the report.

Clare Whitton additionally contributed to *Chapter 4*.

Amanda Perry and **Dean McMillan** provided advice throughout the project on systematic review methods and contributed to the writing of the report; they also supervised junior review staff.

Simon Gilbody helped to structure the report, particulary the sections on methodology and quality assessments.

All of the authors contributed to and commented on the report, with particular support from **Ellen Hughes**, **Danya Glaser**, **Vivien Prior**, **Dominic Trépel**, **Melissa Barry** and **Amanda Perry**.

Other contributors

We would like to thank the expert advisory group and the PPI groups for their contribution at all stages of this report. We would particularly like to thank Sharon McNeil and Amanda Boorman for taking the lead on the PPI work, attending steering group meetings and commenting on report drafts at different stages. Helen Minnis provided additional expert advice at the steering groups and focus group meetings.

We would also like to thank David Marshall and Catherine Arthurson for helping in the production of this report and primary study authors for responding for our requests for information.

Lucy Cottrill, Melissa Barry, Amanda Perry, Jenny Fell, Ellen Hughes, Lisa Hackney, Lauren Bridges and Christopher Thain were responsible for study selection, data extraction and validity assessment. Isobel Barlow and Lisa Hackney contributed to additional searches and data extraction.

© Queen's Printer and Controller of HMSO 2015. This work was produced by Wright *et al.* under the terms of a commissioning contract issued by the Secretary of State for Health. This issue may be freely reproduced for the purposes of private research and study and extracts (or indeed, the full report) may be included in professional journals provided that suitable acknowledgement is made and the reproduction is not associated with any form of advertising. Applications for commercial reproduction should be addressed to: NIHR Journals Library, National Institute for Health Research, Evaluation, Trials and Studies Coordinating Centre, Alpha House, University of Southampton Science Park, Southampton SO16 7NS, UK.

Liz Littlewood, Stephen Palmer, Rachel Richardson, Sophie Bennett and Danielle Moore provided support in the development of the protocol and membership of the steering group alongside all of the other members mentioned above.

Data sharing statement

Full information of all aspects of the trial is available on request from the corresponding author.

References

1. Bowlby J. *Attachment: Attachment and Loss Volume 1*. London: Hogarth Press; 1969.
2. Ainsworth M, Wittig B. Attachment and Exploratory Behavior of One-Year-Olds in a Strange Situation. In Foss BM, editor. *Determinants of Infant Behavior*. London: Methuen; 1969. pp. 111–36.
3. Sroufe LA. Attachment and development: a prospective, longitudinal study from birth to adulthood. *Attach Hum Dev* 2005;**7**:349–67. http://dx.doi.org/10.1080/14616730500365928
4. Cozolino L. *The Neuroscience of Human Relationships: Attachment and the Developing Social Brain*. New York, NY: W. W. Norton & Co.; 2006.
5. Goldberg S. *Attachment and Development*. London: Arnold; 2000.
6. Main M, Solomon J. Procedures for Identifying Infants as Disorganized/Disoriented During the Ainsworth Strange Situation. In Greenberg MT, Cicchetti D, Cummings EM, editors. *Attachment During the Preschool Years: Theory, Research and Intervention*. Chicago, IL: University of Chicago Press; 1990. pp. 121–60.
7. Crittenden P. A dynamic-maturational model of attachment. *Aust N Z J Fam Ther* 2006;**27**:105–15. http://dx.doi.org/10.1002/j.1467-8438.2006.tb00704.x
8. Ainsworth M, Blehar M, Waters E, Wall S. *Patterns of Attachment: A Psychological Study of the Strange Situation*. Hillsdale, NJ: Lawrence Erlbaum Associates; 1978.
9. De Wolff M, Van IJzendoorn M. Sensitivity and attachment: a meta-analysis on parental antecedents of infant attachment. *Child Dev* 1997;**68**:571–91. http://dx.doi.org/10.1111/j.1467-8624.1997.tb04218.x
10. Fonagy P, Target M, Steele H, Steele M. *Reflective Functioning Manual, Version 5.0, for Application to Adult Attachment Interviews*. London: University College London; 1998.
11. Slade A. Parental reflective functioning: an introduction. *Attach Hum Dev* 2005;**7**:269–81. http://dx.doi.org/10.1080/14616730500245906
12. Meins E, Fernyhough C, Fradley E, Tuckley M. Rethinking maternal sensitivity: mother's comments on infants' mental processes predict security of attachment at 12 months. *J Child Psychol Psychiatry* 2001;**42**:637–48. http://dx.doi.org/10.1111/1469-7610.00759
13. Steele H, Steele M, Fonagy P. Associations among attachment classifications of mothers, fathers, and their infants. *Child Dev* 1996;**67**:541–55. http://dx.doi.org/10.2307/1131831
14. Roisman G, Booth-Laforce C, Belsky J. Molecular-genetic correlates of infant attachment: a cautionary tale. *Attach Hum Dev* 2013;**15**:384–406. http://dx.doi.org/10.1080/14616734.2013.768790
15. Bakermans-Kranenburg MJ, Van IJzendoorn MH. Research review: genetic vulnerability or differential susceptibility in child development: the case of attachment. *J Child Psychol Psychiatry* 2007;**48**:1160–73. http://dx.doi.org/10.1111/j.1469-7610.2007.01801.x
16. Bokhorst CL, Bakermans-Kranenburg MJ, Fearon R, Van IJzendoorn MH, Fonagy P, Schuengel C. The importance of shared environment in mother-infant attachment security: a behavioral genetic study. *Child Dev* 2003;**74**:1769–82. http://dx.doi.org/10.1046/j.1467-8624.2003.00637.x
17. Bouvette-Turcot A, Bernier A, Meaney M. Intergenerational transmission of psychosocial risk: maternal childhood adversity, mother-child attachment, and child temperament. *Psychol Belg* 2013;**53**:65–83. http://dx.doi.org/10.5334/pb-53-3-65

© Queen's Printer and Controller of HMSO 2015. This work was produced by Wright *et al.* under the terms of a commissioning contract issued by the Secretary of State for Health. This issue may be freely reproduced for the purposes of private research and study and extracts (or indeed, the full report) may be included in professional journals provided that suitable acknowledgement is made and the reproduction is not associated with any form of advertising. Applications for commercial reproduction should be addressed to: NIHR Journals Library, National Institute for Health Research, Evaluation, Trials and Studies Coordinating Centre, Alpha House, University of Southampton Science Park, Southampton SO16 7NS, UK.

18. Waters E, Merrick S, Treboux D, Crowell J, Albersheim L. Attachment security in infancy and early adulthood: a twenty-year longitudinal study. *Child Dev* 2000;**71**:684–9. http://dx.doi.org/10.1111/1467-8624.00176

19. Hodges J, Steele M, Hillman S, Henderson K, Kaniuk J. Change and Continuity in Mental Representations of Attachment after Adoption. In Brodzinsky D, Palacios J, editors. *Psychological Issues in Adoption: Research and Practice*. Westport, CT: Praeger; 2005.

20. Bowlby J. *A Secure Base*. New York, NY: Basic Books; 1988.

21. Jacobvitz D, Hazen N. Developmental Pathways from Infant Disorganization to Childhood Peer Relationships. In Solomon J, Carol G, editors. *Attachment Disorganization*. New York, NY: Guilford Press; 1999. pp. 127–59.

22. Main M, Cassidy J. Categories of response to reunion with the parent at age 6: predictable from infant attachment classifications and stable over a 1-month period. *Dev Psychol* 1988;**24**:415.

23. Van IJzendoorn MHS, Bakermans-Kranenburg MJ. Disorganized attachment in early childhood: meta-analysis of precursors, concomitants, and sequelae. *Dev Psychopathol* 1999;**1**:225–49. http://dx.doi.org/10.1017/s0954579499002035

24. Solomon J, George C, De Jong A. Children classified as controlling at age six: evidence of disorganized representational strategies and aggression at home and at school. *Dev Psychopathol* 1995;**7**:447–63. http://dx.doi.org/10.1017/S0954579400006623

25. Madigan S, Ladd M, Goldberg S. A picture is worth a thousand words: children's representations of family as indicators of early attachment. *Attach Hum Dev* 2003;**5**:19–37. http://dx.doi.org/10.1080/1461673031000078652

26. Main M, Hesse E, Kaplan N. Predictability of Attachment Behavior and Representational Processes at 1, 6, and 19 Years of Age. In Grossmann K, Grossmann K, Waters E, editors. *Attachment from Infancy to Adulthood: The Major Longitudinal Studies*. New York, NY: Guilford Press; 2005. pp. 245–304.

27. Weinfield NS, Sroufe L, Egeland B, Carlson EA. The Nature of Individual Differences in Infant-Caregiver Attachment. In Cassidy J, Shaver PR, editors. *Handbook of Attachment: Theory, Research, and Clinical Applications*. New York, NY: Guilford Press; 1999. pp. 68–88.

28. Cowan P, Cohn D, Cowan C, Pearson J. Parent's attachment histories and children's externalizing and internalizing behaviors: exploring family systems models of linkage. *J Consult Clin Psychol* 1996;**64**:53–63. http://dx.doi.org/10.1037/0022-006X.64.1.53

29. Deater-Deckard K, Petrill S. Parent-child dyadic mutuality and child behavior problems: gene-environment processes. *J Child Psychol Psychiatry* 2004;**45**:1171–9. http://dx.doi.org/10.1111/j.1469-7610.2004.00309.x

30. Marchand J, Schedler S, Wagstaff D. Parents' adult attachment representations, depressive symptoms, and conflict resolution strategies: predictors of children's behavior problems. *Early Child Res Q* 2004;**19**:449–62. http://dx.doi.org/10.1016/j.ecresq.2004.07.003

31. Speltz M, Greenberg M, DeKlyen M. Attachment in preschoolers with disruptive behaviour: a comparison of clinic-referred and non-problem children. *Dev Psychopathol* 1990;**2**:31–46. http://dx.doi.org/10.1017/S0954579400000572

32. Futh A, O'Connor T, Matias C, Green J, Stephen S. Attachment narratives and behavior and emotional symptoms in an ethnically diverse, at risk sample. *J Am Acad Child Adolesc* 2008;**47**:709–18. http://dx.doi.org/10.1097/CHI.0b013e31816bff65

33. Leschied AW, Chiodo D, Whitehead PC, Hurley D. The relationship between maternal depression and child outcomes in a child welfare sample: implications for treatment and policy. *Child Fam Soc Work* 2005;**10**:281–91. http://dx.doi.org/10.1111/j.1365-2206.2005.00365.x

34. Van IJzendoorn M, Feldbrugge J, Derks F, de Ruiter C, Verhages M, Philipe M. Attachment representations of personality-disordered criminal offenders. *Am J Orthospsychiatry* 1997;**67**:449–59. http://dx.doi.org/10.1037/h0080246

35. Loman MM, Wiik KL, Frenn KA, Pollak SD, Gunnar MR. Postinstitutionalized children's development: growth, cognitive, and language outcomes. *Dev Behav Pediatr* 2009;**30**:426–34. http://dx.doi.org/10.1097/DBP.0b013e3181b1fd08

36. Modinos G, Aleman A, Ormel J. Cortisol levels in childhood and psychosis risk in late adolescence. *J Am Acad Child Adolesc* 2009;**48**:765–6. http://dx.doi.org/10.1097/CHI.0b013e3181a5e3ff

37. Greenberg M. Attachment and Psychopathology in Childhood. In Cassidy J, Shaver PR, editors. *Handbook of Attachment: Theory, Research and Clinical Applications*. New York, NY: Guilford Press; 1999. pp. 469–96.

38. Boris NW, Zeanah CH, Work Group on Quality Issues. Practice parameter for the assessment and treatment of children and adolescents with reactive attachment disorder of infancy and early childhood. *J Am Acad Child Adolesc Psychiatry* 2005;**44**:1206–19. http://dx.doi.org/10.1097/01.chi.0000177056.41655.ce

39. Rutter M, Kreppner J, Sonuga-Barke E. Attachment insecurity, disinhibited attachment, and attachment disorders: where do research findings leave the concepts? *J Child Psychol Psychiatry* 2009;**50**:529–43. http://dx.doi.org/10.1111/j.1469-7610.2009.02042.x

40. Grossman K, Grossman K, Kindler H. Early Care and the Roots of Attachment and Partnership Representations. In Grossman KE, Grossman K, Waters E, editors. *Attachment from Infancy to Adulthood: The Major Longitudinal Studies*. New York, NY: Guilford Press; 2005. pp. 98–136.

41. Viding E, Blair J, Moffitt T, Plomin R. Evidence for substantial genetic risk for psychopathy in 7-year-olds. *J Child Psychol Psychiatry* 2005;**46**:592–7. http://dx.doi.org/10.1111/j.1469-7610.2004.00393.x

42. Viding E, Frick P, Plomin R. Aetiology of the relationship between callous-unemotional traits and conduct problems in childhood. *Br J Psychiatry* 2007;**190**:S33–8. http://dx.doi.org/10.1192/bjp.190.5.s33

43. O'Connor T, Byrne J. Attachment measures for research and practice. *Child Adolesc Ment Health* 2007;**12**:187–92. http://dx.doi.org/10.1111/j.1475-3588.2007.00444.x

44. Main M, Solomon J. Discovery of Disorganised/Disorientated Attachment Pattern. In Brazelton TB, Yogman MW, editors. *Affective Development in Infancy*. Norwood, NJ: Ablex; 1986. pp. 95–124.

45. Carlson EA. A prospective longitudinal study of attachment disorganization/disorientation. *Child Dev* 1998;**69**:1107–28. http://dx.doi.org/10.1111/j.1467-8624.1998.tb06163.x

46. Van IJzendoorn MH, Kroonenberg PM. Cross-cultural patterns of attachment: a meta-analysis of the strange situation. *Child Dev* 1988;**59**:147–56.

47. Cassidy J, Marvin R, the MacArthur Consortium on Attachment in the Preschool Years 1989–1992. *Attachment Organization in Three- and Four-Year-Olds: Coding Guidelines*. University of Virginia; 1992.

© Queen's Printer and Controller of HMSO 2015. This work was produced by Wright *et al.* under the terms of a commissioning contract issued by the Secretary of State for Health. This issue may be freely reproduced for the purposes of private research and study and extracts (or indeed, the full report) may be included in professional journals provided that suitable acknowledgement is made and the reproduction is not associated with any form of advertising. Applications for commercial reproduction should be addressed to: NIHR Journals Library, National Institute for Health Research, Evaluation, Trials and Studies Coordinating Centre, Alpha House, University of Southampton Science Park, Southampton SO16 7NS, UK.

48. Waters E, Deane K. Defining and Assessing Individual Differences in Attachment Relationships: Q-Methodology and the Organization of Behavior in Infancy and Early Childhood. In Bretherton I, Waters E, editors. *Growing Points of Attachment Theory and Research: Monographs of the Society for Research in Child Development*. Chicago, IL: University of Chicago; 1985. http://dx.doi.org/10.2307/3333826

49. Bretherton I, Oppenheim D. The Macarthur Story Stem Battery: Development, Administration, Reliability, Validity, and Reflections about Meaning. In Emde RN, Wolf DP, Oppenheim D, editors. *Revealing the Inner Worlds of Young Children: The Macarthur Story Stem Battery and Parent–Child Narratives*. New York, NY: Oxford University Press; 2003. pp. 55–80.

50. Goldwyn R, Stanley C, Smith V, Green J. The Manchester child attachment story task: relationship with parental AAI, SAT and child behaviour. *Attach Hum Dev* 2000;**2**:71–84. http://dx.doi.org/10.1080/146167300361327

51. Crittenden P, Kozlowska K, Landini A. Assessing attachment in school-age children. *Clin Child Psychol Psychiatry* 2010;**15**:185–208. http://dx.doi.org/10.1177/1359104509356741

52. Shmueli-Goetz Y, Target M, Fonagy P, Datta A. The Child Attachment Interview: a psychometric study of reliability and discriminant validity. *Dev Psychol* 2008;**4**:939–56. http://dx.doi.org/10.1037/0012-1649.44.4.939

53. Steele M, Murphy A, Steele H. Identifying therapeutic action in an attachment-centered intervention with high risk families. *Clin Soc Work J* 2010;**38**:61–72. http://dx.doi.org/10.1007/S10615-009-0257-6

54. Muris P, Merckelbach H, Gadet B, Moulaert V. Fears, worries, and scary dreams in 4- to 12-year-old children: their content, developmental pattern, and origins. *J Clin Child Psychol* 2000;**29**:43–52.

55. World Health Organization. *The ICD-10 Classification of Mental and Behavioural Disorders*. Geneva: World Health Organization; 1992.

56. American Psychiatric Association. *Diagnostic and Statistical Manual of Mental Disorders*. Washington, DC: APA; 2000.

57. Boris N, Zeanah C. Disturbances and disorders of attachment in infancy: an overview. *Infant Ment Health J* 1999;**20**:1–9. http://dx.doi.org/10.1002/(sici)1097-0355(199921)20:1<1::aid-imhj1>3.0.co;2-v

58. American Psychiatric Association. *Diagnostic and Statistical Manual of Mental Disorders*. Washington, DC: APA; 2013. pp. 5–25.

59. Zero to Three. *Diagnostic Classification of Mental Health and Developmental Disorders of Infancy and Early Childhood, Revised (DC: 0–3R)*. Washington, DC: Zero to Three Press; 2005.

60. Buchsbaum HK. The use of a narrative story stem technique with maltreated children: implications for theory and practice. *Dev Psychopathol* 1992;**4**:603–25. http://dx.doi.org/10.1017/S0954579400004892

61. Mikulincer M, Phillip RS. *Attachment in Adulthood: Structure, Dynamics, and Change*. New York, NY: Guilford Press; 2010.

62. Juffer F, Bakermans-Kranenburg M, Van IJzendoorn M. Attachment-based interventions: heading for evidenced-based ways to support families. *Occas Pap Assoc Child Adolesc Ment Health* 2009;**29**:47–57.

63. Zeanah CH, Berlin LJ, Boris N. Practitioner review: clinical applications of attachment theory and research for infants and young children. *J Child Psychol Psychiatry* 2011;**52**:819–33. http://dx.doi.org/10.1111/j.1469-7610.2011.02399.x

64. Department for Education and Skills. *Every Child Matters: Change for Children*. London: Department for Education and Skills; 2004.

65. Great Britain. *Children Act 2004*. London: The Stationery Office; 2004.

66. HM Treasury. *Choice for Parents, the Best Start for Children: A Ten Year Strategy for Childcare*. Norwich: The Stationery Office; 2004.

67. Department for Children, Schools and Families. *The Children's Plan: Building Brighter Futures*. London: Department for Children, Schools and Families; 2007.

68. Department for Children, Schools and Families. *Statutory Framework for the Early Years Foundation Stage*. London: Department for Children, Schools and Families; 2008.

69. Centre for Social Justice. *Breakthrough Britain: The Next Generation. A Policy Report for the Early Years Commission*. London: Centre for Social Justice; 2008.

70. Webster-Stratton C. *The Incredible Years*. 1992. URL: www.incredibleyears.com (accessed 17 June 2013).

71. Shribman S, Billingham K. *Healthy Child Programme: Pregnancy and the First Five Years of Life*. London: Department of Health; 2009.

72. Department of Health. *The Child Health Promotion Programme: Pregnancy and the First Five Years Of Life*. London: Department of Health; 2008.

73. Department of Health. *National Service Framework for Children*. *Young People and Maternity Services*. London: Department of Health; 2004.

74. Department of Health. *Delivering Health Services through Sure Start Children's Centres*. London: Department of Health; 2007.

75. Prevention Action. *National Academy for Parenting Practitioners*. URL: www.preventionaction.org/reference/national-academy-parenting-practitioners (accessed 17 June 2013).

76. Department of Education. *Parenting Programmes*. URL: www.education.gov.uk/commissioning-toolkit/Programme/CommissionersSearch (accessed 17 June 2013).

77. NHS. *Improving Access to Psychological Therapies*. URL: www.iapt.nhs.uk/cyp-iapt/children-and-young-peoples-project-faqs/ (accessed 17 June 2013).

78. Riessman C, Gerstel N. Marital dissolution and health: do males or females have greater risk? *Soc Sci Med* 1985;**6**:627–35. http://dx.doi.org/10.1016/0277-9536(85)90401-0

79. Schnittker J, John A. Enduring stigma: the long term effects of incarceration on health. *J Health Soc Behav* 2007;**2**:115–30. http://dx.doi.org/10.1177/002214650704800202

80. Marmot M. *Fair Society, Healthy Lives: The Marmot Review*. London: The Marmot Review; 2010.

81. Munro E. *The Munro Review of Child Protection: Final Report – A Child-Centred System*. London: Department for Education; 2011.

82. Field F. *The Foundation Years: Preventing Poor Children Becoming Poor Adults*. London: HM Government; 2010.

83. Allen G. *Early Intervention: The Next Steps*. London: HM Government; 2011.

84. Tickell C. *The Early Years: Foundations for Life, Health and Learning – Tickell Review*. London: Department for Education; 2011.

85. Department for Education and Department of Health. *Supporting Families in the Foundation Years*. Department for Education and Department of Health; 2011.

© Queen's Printer and Controller of HMSO 2015. This work was produced by Wright *et al.* under the terms of a commissioning contract issued by the Secretary of State for Health. This issue may be freely reproduced for the purposes of private research and study and extracts (or indeed, the full report) may be included in professional journals provided that suitable acknowledgement is made and the reproduction is not associated with any form of advertising. Applications for commercial reproduction should be addressed to: NIHR Journals Library, National Institute for Health Research, Evaluation, Trials and Studies Coordinating Centre, Alpha House, University of Southampton Science Park, Southampton SO16 7NS, UK.

86. Juffer F, Bakermans-Kranenburg M. Attachment-based interventions: heading for evidenced-based ways to support families. *Occas Pap Assoc Child Adolesc Ment Health* 2009;**29**:47–57.

87. Prior V, Glaser D. *Understanding Attachment and Attachment Disorders: Theory, Evidence and Practice*. London: Jessica Kingsley Publishers; 2006.

88. World Health Organization. *The ICD-11 Classification of Mental and Behavioural Disorders*. Geneva: World Health Organization; 2010.

89. Centre for Reviews and Dissemination. *Systematic Reviews: CRD's Guidance for Undertaking Systematic Reviews in Health Care*. York: University of York; 2009.

90. Moher D, Liberati A, Tetzlaff J, Altman DG, The PRISMA Group. Preferred Reporting Items for Systematic Reviews and Meta-Analyses: the PRISMA statement. *BMJ* 2009;**339**:b2535. http://dx.doi.org/10.1136/bmj.b2535

91. Higgins JPT, Green S, editors. *Cochrane Handbook for Systematic Reviews of Interventions Version 5.1.0 (updated March 2011)*. Cochrane; 2011.

92. Whiting P, Rutjes A, Westwood M, Mallett S, Deeks J, Reitsma J, *et al.* QUADAS-2: a revised tool for the quality assessment of diagnostic accuracy studies. *Ann Intern Med* 2011;**15**:529–36. http://dx.doi.org/10.7326/0003-4819-155-8-201110180-00009

93. Clarke-Stewart K, Goossens FA, Allhusen VD. Measuring infant-mother attachment: is the Strange Situation enough? *Soc Dev* 2001;**10**:143–69. http://dx.doi.org/10.1111/1467-9507.00156

94. Finkel D, Wille DE, Matheny AP. Preliminary results from a twin study of infant-caregiver attachment. *Behav Genet* 1998;**28**:1–8. http://dx.doi.org/10.1023/A:1021448429653

95. Gleason MM, Fox NA, Drury S, Smyke A, Egger HL, Nelson CA, *et al.* Validity of evidence-derived criteria for reactive attachment disorder: indiscriminately social/disinhibited and emotionally withdrawn/inhibited types. *J Am Acad Child Adolesc Psychiatry* 2011;**50**:216–31. http://dx.doi.org/10.1016/j.jaac.2010.12.012

96. Aber J, Baker A. Security of attachment in toddlerhood: modifying assessment procedures for joint clinical and research purposes, in attachment in the preschool years: theory, research, and intervention. In Greenberg M, Cicchetti D, Cummings M, editors. Preschool Years: Theory, Research, and Intervention. Chicago, IL: University of Chicago Press; 1990. pp. 427–60.

97. Backman TL. *Examining Parent–Child Relationships in Forensic Attachment Assessments: A Construct Validity Investigation of the Marschak Interaction Method Rating System*. PhD thesis. Fresno, CA: Alliant International University; 2002.

98. Boris NW, Hinshaw-Fuselier SS, Smyke AT, Scheeringa MS, Heller SS, Zeanah CH. Comparing criteria for attachment disorders: establishing reliability and validity in high-risk samples. *J Am Acad Child Adolesc Psychiatry* 2004;**43**:568–77. http://dx.doi.org/10.1097/00004583-200405000-00010

99. Bureau JF, Easlerbrooks M, Lyons-Ruth K. Attachment disorganization and controlling behavior in middle childhood: maternal and child precursors and correlates. *Attach Hum Dev* 2009;**1**:265–84. http://dx.doi.org/10.1080/14616730902814788

100. Crittenden PM, Claussen AH, Kozlowska K. Choosing a valid assessment for attachment for a clinical use: a comparative study. *Aust N Z J Fam Ther* 2007;**28**:78–87. http://dx.doi.org/10.1375/anft.28.2.78

101. Equit M, Paulus F, Fuhrmann P, Niemczyk J, von Gontard A. Comparison of ICD-10 and DC: 0–3R diagnoses in infants, toddlers and preschoolers. *Child Psychiatry Hum Dev* 2011;**42**:623–33. http://dx.doi.org/10.1007/s10578-011-0237-2

102. Fagot BI, Pears KC. Changes in attachment during the third year: consequences and predictions. *Dev Psychopathol* 1996;**8**:325–44. http://dx.doi.org/10.1017/S0954579400007124

103. Fury G, Carlson EA, Sroufe L. Children's representations of attachment relationships in family drawings. *Child Dev* 1997;**68**:1154–64. http://dx.doi.org/10.2307/1132298

104. Gurganus SL. *A Reliability and Validity Study of the May-Nichols Child Behavior Rating Scale*. PhD thesis. Eagan, MI: Minnesota School of Professional Psychology, Argosy University; 2002.

105. Head TL. *Children's Conceptualizations of Attachment and Caregiving*. PhD thesis. Vancouver, BC: University of British Columbia; 1997.

106. Mangelsdorf SC, Plunkett JW, Dedrick CF, Berlin M, Meisels SJ, McHale JL, *et al.* Attachment security in very low birth weight infants. *Dev Psychol* 1996;**32**:914–20. http://dx.doi.org/10.1037/0012-1649.32.5.914

107. Minnis H, Read W, Connolly B, Burston A, Schumm T-S, Putter-Lareman S, *et al.* The Computerised Manchester Child Attachment Story Task: a novel medium for assessing attachment patterns. *Int J Methods Psychiatr Res* 2010;**19**:233–42. http://dx.doi.org/10.1002/mpr.324

108. Minnis H, Green J, O'Connor TG, Liew A, Glaser D, Taylor E, *et al.* An exploratory study of the association between reactive attachment disorder and attachment narratives in early school-age children. *J Child Psychol Psychiatry* 2009;**50**:931–42. http://dx.doi.org/10.1111/j.1469-7610.2009.02075.x

109. McLaughlin A, Espie C, Minnis H. Development of a brief waiting room observation for behaviours typical of reactive attachment disorder. *Child Adolesc Ment Health* 2010;**15**:73–9. http://dx.doi.org/10.1111/j.1475-3588.2009.00549.x

110. Ogilvie AM. *The Assessment of Children with Attachment Disorder: The Randolph Attachment Disorder Questionnaire, the Behavioral and Emotional Rating Scale, and the Biopsychosocial Attachment Types Framework*. PhD thesis. Portland, OR: Portland State University; 2000.

111. Oppenheim D. *Assessing the Validity of a Doll Play Interview for Measuring Attachment in Pre-Schoolers*. PhD thesis. Salt Lake City, UT: University of Utah; 1990.

112. Oppenheim D. The attachment doll-play interview for preschoolers. *Int J Behav Dev* 1997;**20**:681–97. http://dx.doi.org/10.1080/016502597385126

113. Posada G. Assessing attachment security at age three: Q-sort home observations and the MacArthur Strange Situation adaptation. *Soc Dev* 2006;**15**:644–58. http://dx.doi.org/10.1111/j.1467-9507.2006.00362.x

114. Roman M. *Attachment in Adopted Children. Attachment Representations, Behavior and Disturbances*. PhD thesis. Seville: University of Seville; 2010.

115. Silver BL. *A Study of the Convergent and Discriminant Validity of School-Aged Children's Family Drawings as a Measure of Attachment Security*. PhD thesis. Pittsburgh, PA: University of Pittsburgh; 2005.

116. Sirl KS. *Validity of the Attachment Story Completion Task among African-American Six-Year-Olds*. Detroit, MI: Wayne State University; 1999.

117. Smeekens S, Riksen-Walraven J, Van Bakel HJ. The predictive value of different infant attachment measures for socioemotional development at age 5 years. *Infant Ment Health J* 2009;**30**:366–83. http://dx.doi.org/10.1002/imhj.20219

118. Spieker S, Crittenden PM. Comparing two attachment classification methods applied to preschool strange situations. *Clin Child Psychol Psychiatry* 2010;**15**:97–120. http://dx.doi.org/10.1177/1359104509345878

© Queen's Printer and Controller of HMSO 2015. This work was produced by Wright *et al.* under the terms of a commissioning contract issued by the Secretary of State for Health. This issue may be freely reproduced for the purposes of private research and study and extracts (or indeed, the full report) may be included in professional journals provided that suitable acknowledgement is made and the reproduction is not associated with any form of advertising. Applications for commercial reproduction should be addressed to: NIHR Journals Library, National Institute for Health Research, Evaluation, Trials and Studies Coordinating Centre, Alpha House, University of Southampton Science Park, Southampton SO16 7NS, UK.

119. Tarabulsy GM, Moran G. Similarities and differences in mothers' and observers' descriptions of attachment behaviors. *Int J Behav Dev* 1997;**21**:599–619. http://dx.doi.org/10.1080/016502597384802

120. van Dam M, Van IJzendoorn MH. Measuring attachment security: concurrent and predictive validity of the Parental Attachment Q-set. *J Genet Psychol* 1988;**149**:447–57. http://dx.doi.org/10.1080/00221325.1988.10532172

121. Vaughn BE, Waters E. Attachment behavior at home and in the laboratory: Q-sort observations and strange situation classifications of one-year-olds. *Child Dev* 1990;**61**:1965–73. http://dx.doi.org/10.2307/1130850

122. Ziegenhein U, Jacobsen T. Assessing children's representational attachment models: links to mother-child attachment quality in infancy and childhood. *J Genet Psychol* 1999;**160**:22–30. http://dx.doi.org/10.1080/00221329909595377

123. Waters E. *Attachment Behavior Q-Set (Revision 3.0)*. New York, NY: Department of Psychology, State University of New York at Stony Brook; 1987.

124. Crittenden P. Social networks, quality of child rearing, and child development. *Child Dev* 1985:1299–313. http://dx.doi.org/10.2307/1130245

125. Waters E. The Attachment Q-Set (Version 3.0). In Waters E, Vaughn BE, Posada G, Kondo-Ikemura K, editors. *Caregiving Culture, and Cognitive Perspectives on Secure Base Behaviour and Working Models: New Growing Points of Attachment Theory and Research (Monographs of the Society for Research in Child Development)*. Chicago, IL: University of Chicago Press; 1995.

126. Chisholm J. Attachment and time preference. *Human Nat* 1999;**10**:51–83. http://dx.doi.org/10.1007/s12110-999-1001-1

127. Crittenden P. Maltreated infants: vulnerability and resilience. *J Child Psychol Psychiatry* 2006;**26**:85–96. http://dx.doi.org/10.1111/j.1469-7610.1985.tb01630.x

128. Main M, Weston DR. The quality of the toddler's relationship to mother and to father: related to conflict behaviour and the readiness to establish new relationships. *Child Dev* 1981;**52**:932–40. http://dx.doi.org/10.2307/1129097

129. Barnett B, Blignault I, Holmes S, Payne A. Quality of attachment in a sample of 1-year-old Australian children. *J Am Acad Child Adolesc Psychiatry* 1987;**26**:303–7. http://dx.doi.org/10.1097/00004583-198705000-00003

130. Van IJzendoorn M, Vereijken C, Bakermans-Kranenburg M, Marianne Riksen-Walraven J. Assessing attachment security with the attachment Q sort: meta-analytic evidence for the validity of the observer AQS. *Child Dev* 2004;**75**:1188–213. http://dx.doi.org/10.1111/j.1467-8624.2004.00733.x

131. Allen J, McElhaney K, Land D, Kuperminc G, Moore C, O'Beirne-Kelly H. A secure base in adolescence: markers of attachment security in the mother–adolescent relationship. *Child Dev* 2003;**74**:292–307. http://dx.doi.org/10.1111/1467-8624.t01-1-00536

132. Target M, Fonagy P, Schneider T, Ensink K, Janes K. *Raters' Manual for the Hampstead Child Adaptation Measure (HCAM)*. London: University College London and the Anna Freud Centre; 2000.

133. Barnett B, Parker G. Professional and non-professional intervention for highly anxious primiparous mothers. *Br J Psychiatry* 1985;**146**:287–93. http://dx.doi.org/10.1192/bjp.146.3.287

134. Chisholm K. A three year follow-up of attachment and indiscriminate friendliness in children adopted from Romanian orphanages. *Child Dev* 1998;**69**:1092–106. http://dx.doi.org/10.1111/j.1467-8624.1998.tb06162.x

135. Chisholm K, Carter M, Ames E, Morison S. Attachment security and indiscriminately friendly behavior in children adopted from Romanian orphanages. *Dev Psychopathol* 1995;**7**:283–94. http://dx.doi.org/10.1017/S0954579400006507

136. Hansburg H. *Adolescent Separation Anxiety: A Method for the Study of Adolescent Separation Problems*. Springfield, IL: Charles C. Thomas; 1972.

137. Klagsbrun M, Bowlby J. Responses to separation from parents: a clinical test for young children. *Br J Project Psychol* 1976;**21**:7–21.

138. Slough N, Greenberg M. Five-year-olds' representations of separations from parents: responses from the perspective of self and other. *New Dir Child Dev* 1990;**48**:67–84. http://dx.doi.org/10.1002/cd.23219904806

139. Burns RC, Kaufman SH. *Actions, Styles and Symbols in Kinetic Family Drawings (KF-D): An Interpretive Manual*. New York, NY: Bruner/Mazel; 1972.

140. Emde R, Wolf D, Oppenheim D. *Revealing the Inner Worlds of Young Children: The MacArthur Story Stem Battery and Parent–Child Narratives*. New York, NY: Oxford University Press; 2003.

141. Woolgar M. Children's play narrative responses to hypothetical dilemmas and their awareness of moral emotions. *Br J Dev Psychol* 2001;**19**:115–28. http://dx.doi.org/10.1348/026151001165994

142. Macfie J. Effect of maltreatment on preschoolers' narrative representations of responses to relieve distress and of role reversal. *Dev Psychol* 1999;**35**:460. http://dx.doi.org/10.1037/0012-1649.35.2.460

143. Grych JH, Wachsmuth-Schlaefer T, Klockow LL. Interparental aggression and young children's representations of family relationships. *J Fam Psychol* 2002;**16**:259. http://dx.doi.org/10.1037/0893-3200.16.3.259

144. von Klitzing K, Kelsay K, Emde RN, Robinson J, Schmitz S. Gender-specific characteristics of 5-year-olds' play narratives and associations with behavior ratings. *J Am Acad Child Adolesc Psychiatry* 2000;**39**:1017–23. http://dx.doi.org/10.1097/00004583-200008000-00017

145. Warren S. *Revealing the Inner World of Young Children*. New York, NY: Oxford University Press; 2003.

146. Hitchcock D. *Construct Validity Study of the Marschak Interaction Rating System with Adolescent Mother–Child Dyads*. PhD thesis. Fresno, CA: California School of Professional Psychology; 2002.

147. Green J, Goldwyn R. Annotation: attachment disorganisation and psychopathology: new findings in attachment research and their potential implications for developmental psychopathology in childhood. *J Child Psychol Psychiatry* 2002;**43**:835–46. http://dx.doi.org/10.1111/1469-7610.00102

148. Sanderson S, Tatt ID, Higgins JPT. Tools for assessing quality and susceptibility to bias in observational studies in epidemiology: a systematic review and annotated bibliography. *Int J Epidemiol* 2007;**36**:666–76. http://dx.doi.org/10.1093/ije/dym018

149. Wells G, Shea B, O'Connell D, Peterson J, Welch V, Losos M. *The Newcastle-Ottawa Scale (NOS) for Assessing the Quality of Nonrandomized Studies in Meta-Analyses*. OHR Institute; 2009. URL: www.ohri.ca/programs/clinical_epidemiology/oxford.asp (accessed 2 May 2013).

150. Scottish Intercollegiate Guidelines Network (SIGN). *Methodology Checklist 3: Cohort Studies*. Edinburgh; SIGN; 2012.

© Queen's Printer and Controller of HMSO 2015. This work was produced by Wright *et al.* under the terms of a commissioning contract issued by the Secretary of State for Health. This issue may be freely reproduced for the purposes of private research and study and extracts (or indeed, the full report) may be included in professional journals provided that suitable acknowledgement is made and the reproduction is not associated with any form of advertising. Applications for commercial reproduction should be addressed to: NIHR Journals Library, National Institute for Health Research, Evaluation, Trials and Studies Coordinating Centre, Alpha House, University of Southampton Science Park, Southampton SO16 7NS, UK.

151. Aikins JW, Howes C, Hamilton C. Attachment stability and the emergence of unresolved representations during adolescence. *Attach Hum Dev* 2009;**1**:491–512. http://dx.doi.org/10.1080/14616730903017019

152. Weinfield N, Whaley G, Egeland B. Continuity, discontinuity, and coherence in attachment from infancy to late adolescence: sequelae of organization and disorganization. *Attach Hum Dev* 2004;**6**:73–97. http://dx.doi.org/10.1080/14616730310001659566

153. Carlson EA, Egeland B, Sroufe L. A prospective investigation of the development of borderline personality symptoms. *Dev Psychopathol* 2009;**21**:1311–34. http://dx.doi.org/10.1017/S0954579409990174

154. Ammaniti M, Speranza AM, Fedele S. Attachment in Infancy and in Early and Late Childhood: A Longitudinal Study. In Kerns KA, Richardson RA, editors. *Attachment in Middle Childhood*. New York, NY: Guilford Press; 2005. pp. 115–36.

155. Dan O, Sagi-Schwartz A, Bar-haim Y, Eshel Y. Effects of early relationships on children's perceived control: a longitudinal study. *Int J Behav Dev* 2011;**35**:449–56. http://dx.doi.org/10.1177/0165025411406862

156. Jaffari-Bimmel N, Juffer F, Van IJzendoorn MH, Bakermans-Kranenburg MJ, Mooijaart A. Social development from infancy to adolescence: longitudinal and concurrent factors in an adoption sample. *Dev Psychol* 2006;**42**:1143–53. http://dx.doi.org/10.1037/0012-1649.42.6.1143

157. Steele M, Steele H, Johansson M. Maternal predictors of children's social cognition: an attachment perspective. *J Child Psychol Psychiatry* 2002;**43**:861–72. http://dx.doi.org/10.1111/1469-7610.00096

158. University of Minnesota. *Minnesota Longitudinal Study of Risk and Adaptation: Publications*. URL: www.cehd.umn.edu/icd/research/parent-child/publications/attachment.html (accessed 21 November 2013).

159. Hesse E. The Adult Attachment Interview: Historical and Current Perspectives. In Cassidy J, Shaver PR, editors. *Handbook of Attachment: Theory, Research, and Clinical Applications*. New York, NY: Guildford Press; 1999; pp. 395–433.

160. George C, West M, Pettem O. *The Adult Attachment Projective: Disorganization of Adult Attachment at the Level of Representation*. 1999.

161. Carlson E, Putnam F. An update on the Dissociative Experiences Scale. *Dissociation* 1993;**7**:16–27.

162. First MB, Spitzer RL, Gibbon M, Williams JBW. *Structured Clinical Interview for DSM-IV Personality Disorders (SCID-II)*. Washington, DC: American Psychiatric Press; 1997.

163. Hubbs-Tait L, Hughes KP, Culp AM, Osofsky JD, Hahn DM, Eberhartwright A, *et al.* Children of adolescent mothers: attachment representation, materal depression, and later behavior problems. *Am J Orthopsychiatry* 1996;**66**:416–26. http://dx.doi.org/10.1037/h0080192

164. Goldberg S, Gotowiec A, Simmons RJ. Infant–mother attachment and behavior problems in healthy and critically ill preschoolers. *Dev Psychopathol* 1995;**7**:267–82. http://dx.doi.org/10.1017/S0954579400006490

165. Lyons-Ruth K, Easterbrooks MA, Cibelli CD. Infant attachment strategies, infant mental lag, and maternal depressive symptoms: predictors of internalizing and externalizing problems at age 7. *Dev Psychopathol* 1997;**33**:681–92. http://dx.doi.org/10.1037/0012-1649.33.4.681

166. Radke-Yarrow M, McCann K, Demulder E, Belmont B, Martinez P, Richardson DT. Attachment in the context of high-risk conditions. *Dev Psychopathol* 1995;**7**:247–65. http://dx.doi.org/10.1017/S0954579400006489

167. Shaw DS, Owens EB, Vondra JI, Keenan K, Winslow EB. Early risk factors and pathways in the development of early disruptive behavior problems. *Dev Psychopathol* 1996;**8**:679–99. http://dx.doi.org/10.1017/S0954579400007367

168. Greenberg MT, Speltz ML, DeKlyen M, Endriga MC. Attachment security in pre-schoolers with and without externalizing behavior problems: a replication. *Dev Psychopathol* 1991;**3**:413–30. http://dx.doi.org/10.1017/S0954579400007604

169. Marcovitch S, Goldberg SA, Gold A, Washington J, Wasson C, Krekewich K, *et al.* Determinants of behavioural problems in Romanian children adopted in Ontario. *Int J Behav Dev* 1997;**20**:17–31. http://dx.doi.org/10.1080/016502597385414

170. Moss E, Parent S, Gosselin C, Rousseau D, St-Laurent D. Attachment and teacher reported behavior problems during the preschool and early school age period. *Dev Psychopathol* 1996;**8**:511–25. http://dx.doi.org/10.1017/S0954579400007240

171. Wartner U, Grossmann K, Fremmer-Bombik E, Suess G. Attachment patterns at age six in south Germany: predictability from infancy and implications for preschool behavior. *Child Dev* 1994;**65**:1014–27. http://dx.doi.org/10.2307/1131301

172. Verschueren K, Marcoen A. Representation of self and socioemotional competence in kindergartners: differential and combined effects of attachment to mother and to father. *Child Dev* 1999;**70**:183–201. http://dx.doi.org/10.1111/1467-8624.00014

173. Sagi A, Van IJzendoorn M, Scharf M, Koren-Karie N, Joels T, Mayseless O. Stability and discriminant validity of the Adult Attachment Interview: a psychometric study in young Israeli adults. *Dev Psychol* 1994;**30**:771. http://dx.doi.org/10.1037/0012-1649.30.5.771

174. Higgins J, Green S, editors. *Cochrane Handbook for Systematic Reviews of Interventions Version 5.0.2*. Chichester: John Wiley & Sons, Ltd; 2009.

175. Niccols A. 'Right from the Start': randomized trial comparing an attachment group intervention to supportive home visiting. *J Child Psychol Psychiatry* 2008;**49**:754–64. http://dx.doi.org/10.1111/j.1469-7610.2008.01888.x

176. O'Connor T, Matias C, Futh A, Tantam G, Scott S. Social learning theory parenting intervention promotes attachment-based caregiving in young children: randomized clinical trial. *J Clin Child Adolesc Psychol* 2013;**42**:358–70. http://dx.doi.org/10.1080/15374416.2012.723262

177. Spieker SJ, Oxford ML, Kelly JF, Nelson EM, Fleming CB. Promoting first relationships: randomized trial of a relationship-based intervention for toddlers in child welfare. *Child Maltreat* 2012;**17**:271–86. http://dx.doi.org/10.1177/1077559512458176

178. van Doesum KT, Riksen-Walraven J, Hosman CM, Hoefnagels C. A randomized controlled trial of a home-visiting intervention aimed at preventing relationship problems in depressed mothers and their infants. *Child Dev* 2008;**79**:547–61. http://dx.doi.org/10.1111/j.1467-8624.2008.01142.x

179. Kersten-Alvarez LE, Hosman CM, Riksen-Walraven J, van Doesum KT, Hoefnagels C. Long-term effects of a home-visiting intervention for depressed mothers and their infants. *J Child Psychol Psychiatry* 2010;**51**:1160–70. http://dx.doi.org/10.1111/j.1469-7610.2010.02268.x

180. Anisfeld E, Casper V, Nozyce M, Cunningham N. Does infant carrying promote attachment? An experimental study of the effects of increased physical contact on the development of attachment. *Child Dev* 1990;**61**:1617–27. http://dx.doi.org/10.2307/1130769

181. Fisher PA, Kim HK. Intervention effects on foster preschoolers' attachment-related behaviors from a randomized trial. *Prev Sci* 2007;**8**:161–70. http://dx.doi.org/10.1007/s11121-007-0066-5

© Queen's Printer and Controller of HMSO 2015. This work was produced by Wright *et al.* under the terms of a commissioning contract issued by the Secretary of State for Health. This issue may be freely reproduced for the purposes of private research and study and extracts (or indeed, the full report) may be included in professional journals provided that suitable acknowledgement is made and the reproduction is not associated with any form of advertising. Applications for commercial reproduction should be addressed to: NIHR Journals Library, National Institute for Health Research, Evaluation, Trials and Studies Coordinating Centre, Alpha House, University of Southampton Science Park, Southampton SO16 7NS, UK.

182. Forman DR, O'Hara MW, Stuart S, Gorman LL, Larsen KE, Coy KC. Effective treatment for postpartum depression is not sufficient to improve the developing mother–child relationship. *Dev Psychopathol* 2007;**19**:585–602. http://dx.doi.org/10.1017/S0954579407070289

183. Kalinauskiene L, Cekuoliene D, Van IJzendoorn M, Bakermans-Kranenburg M, Juffer F, Kusakovskaja I. Supporting insensitive mothers: the Vilnius randomized control trial of video-feedback intervention to promote maternal sensitivity and infant attachment security. *Child Care Health Dev* 2009;**35**:613–23. http://dx.doi.org/10.1111/j.1365-2214.2009.00962.x

184. Toth SL, Maughan A, Manly JT, Spagnola M, Cicchetti D. The relative efficacy of two interventions in altering maltreated preschool children's representational models: implications for attachment theory. *Dev Psychopathol* 2002;**14**:877–908. http://dx.doi.org/10.1017/S095457940200411X

185. Hansen R, Ulrey G. Motorically impaired infants: impact of a massage procedure on caregiver–infant interactions. *J Multihandicap Pers* 1988;**1**:61–8.

186. Jacobson SW, Frye KF. Effect of maternal social support on attachment: experimental evidence. *Child Dev* 1991;**62**:572–82. http://dx.doi.org/10.2307/1131132

187. Minnis H, Pelosi A, Knapp M, Dunn J. Mental health and foster carer training. *Arch Dis Child* 2001;**84**:302–6. http://dx.doi.org/10.1136/adc.84.4.302

188. Minnis HJ. *Evaluation of a Training Programme for Foster Carers*. London: Institute of Psychiatry; 1999.

189. Cooper PJ, Tomlinson M, Swartz L, Landman M, Molteno C, Stein A, *et al.* Improving quality of mother–infant relationship and infant attachment in socioeconomically deprived community in South Africa: randomised controlled trial. *BMJ* 2009;**338**:1–11. http://dx.doi.org/10.1136/bmj.b974

190. Heinicke CM, Fineman NR, Ponce VA, Guthrie D. Relation-based intervention with at-risk mothers: outcome in the second year of life. *Infant Ment Health J* 2001;**2**:431–62. http://dx.doi.org/10.1002/imhj.1010

191. Heinicke CM, Fineman NR, Ruth G, Recchia SL, Guthrie D, Rodning C. Relationship-based intervention with at-risk mothers: outcome in the first year of life. *Infant Ment Health J* 19;**20**:349–74. http://dx.doi.org/10.1002/(sici)1097-0355(199924)20:4<349::aid-imhj1>3.0.co;2-x

192. Heinicke CM, Goorsky M, Moscov S, Dudley K, Gordon J, Schneider C, *et al.* Relationship-based intervention with at-risk mothers: factors affecting variations in outcome. *Infant Ment Health J* 2000;**21**:133–55.

193. Bernard K, Dozier M, Bick J, Lewis-Morrarty E, Lindhiem O, Carlson E. Enhancing attachment organization among maltreated children: results of a randomized clinical trial. *Child Dev* 2012;**83**:623–36. http://dx.doi.org/10.1111/j.1467-8624.2011.01712.x

194. Dozier M, Lindhiem O, Lewis E, Bick J, Bernard K, Peloso E. Effects of a foster parent training program on young children's attachment behaviors: preliminary evidence from a randomized clinical trial. *Child Adolesc Soc Work J* 2009;**26**:321–32. http://dx.doi.org/10.1007/s10560-009-0165-1

195. Moss E, Dubois-Comtois K, Cyr C, Tarabulsy GM, St-Laurent D, Bernier A. Efficacy of a home-visiting intervention aimed at improving maternal sensitivity, child attachment, and behavioral outcomes for maltreated children: a randomized control trial. *Dev Psychopathol* 2011;**23**:195–210. http://dx.doi.org/10.1017/s0954579410000738

196. Cassidy J, Woodhouse SS, Sherman LJ, Stupica B, Lejuez C. Enhancing infant attachment security: an examination of treatment efficacy and differential susceptibility. *Dev Psychopathol* 2011;**23**:131–48. http://dx.doi.org/10.1017/s0954579410000696

197. van den Boom DC. Do first-year intervention effects endure? Follow-up during toddlerhood of a sample of Dutch irritable infants. *Child Dev* 1995;**6**:1798–816. http://dx.doi.org/10.2307/1131911

198. van den Boom DC. The influence of temperament and mothering on attachment and exploration: an experimental manipulation of sensitive responsiveness among lower-class mothers with irritable infants. *Child Dev* 1994;**65**:1457–77. http://dx.doi.org/10.2307/1131511

199. Toth SL, Rogosch FA, Manly JT, Cicchetti D. The efficacy of toddler-parent psychotherapy to reorganize attachment in the young offspring of mothers with major depressive disorder: a randomized preventive trial. *J Consult Clin Psychol* 2006;**74**:1006–16. http://dx.doi.org/10.1037/0022-006X.74.6.1006

200. Cicchetti D, Toth SL, Rogosch FA. The efficacy of toddler-parent psychotherapy to increase attachment security in offspring of depressed mothers. *Attach Hum Dev* 1999;**1**:34–66. http://dx.doi.org/10.1080/14616739900134021

201. Beckwith L. Intervention with disadvantaged parents of sick preterm infants. *Psychiatry* 1988;**51**:242–7.

202. Brisch KH, Bechinger D, Betzler S, Heinemann H. Early preventive attachment-oriented psychotherapeutic intervention program with parents of a very low birthweight premature infant: results of attachment and neurological development. *Attach Hum Dev* 2003;**5**:120–35. http://dx.doi.org/10.1080/1461673031000108504

203. Klein-Velderman M, Bakermans-Kranenburg MJ, Juffer F, Van IJzendoorn MH. Effects of attachment-based interventions on maternal sensitivity and infant attachment: differential susceptibility of highly reactive infants. *J Fam Psychol* 2006;**20**:266–74. http://dx.doi.org/10.1037/0893-3200.20.2.266

204. Klein-Velderman M, Bakermans-Kranenburg MJ, Juffer F, Van IJzendoorn MH, Mangelsdorf SC, Zevalkink J. Preventing preschool externalizing behavior problems through video-feedback intervention in infancy. *Infant Ment Health J* 2006;**27**:466–93. http://dx.doi.org/10.1002/imhj.20104

205. Roggman LA, Boyce LK, Cook GA. Keeping kids on track: impacts of a parenting-focused Early Head Start program on attachment security and cognitive development. *Early Educ Dev* 2009;**20**:920–41. http://dx.doi.org/10.1080/10409280903118416

206. Ammaniti M, Speranza A, Tambelli R, Muscetta S, Lucarelli L, Vismara L, *et al.* A prevention and promotion intervention program in the field of mother–infant relationship. *Infant Ment Health J* 2006;**27**:70–90. http://dx.doi.org/10.1002/imhj.20081

207. Briskman J, Scott S. *Randomised Controlled Trial of the Fostering Changes Programme: Report for the Department for Education*. The National Academy for Parenting Research; 2012.

208. Murray L, Cooper PJ, Wilson A, Romaniuk H. Controlled trial of the short- and long-term effect of psychological treatment of post-partum depression. II. Impact on the mother–child relationship and child outcome. *Br J Psychiatry* 2003;**182**:420–7. http://dx.doi.org/10.1192/bjp.182.5.420

209. Cooper P, Murray L, Wilson A, Romaniuk H. Controlled trial of the short- and long-term effect of psychological treatment of post-partum depression. I. Impact on maternal mood. *Br J Psychiatry* 2003;**182**:412–19. http://dx.doi.org/10.1192/bjp.182.5.412

210. Stovall–McClough K, Dozier M. Forming attachments in foster care: infant attachment behaviors during the first 2 months of placement. *Dev Psychopathol* 2004;**16**:253–71. http://dx.doi.org/10.1017/S0954579404044505

211. Lieberman AF, Weston DR, Pawl JH. Preventive intervention and outcome with anxiously attached dyads. *Child Dev* 1991;**62**:199–209. http://dx.doi.org/10.2307/1130715

© Queen's Printer and Controller of HMSO 2015. This work was produced by Wright *et al.* under the terms of a commissioning contract issued by the Secretary of State for Health. This issue may be freely reproduced for the purposes of private research and study and extracts (or indeed, the full report) may be included in professional journals provided that suitable acknowledgement is made and the reproduction is not associated with any form of advertising. Applications for commercial reproduction should be addressed to: NIHR Journals Library, National Institute for Health Research, Evaluation, Trials and Studies Coordinating Centre, Alpha House, University of Southampton Science Park, Southampton SO16 7NS, UK.

212. Sajaniemi N, Makela J, Saolkorpi T, von Wendt L, Hamalainen T, Hakamies-Blomqvist L. Cognitive performance and attachment patterns at four years of age in extremely low birth weight infants after early intervention. *Eur Child Adolesc Psychiatry* 2001;**10**:122–9. http://dx.doi.org/10.1007/s007870170035

213. Foley G, Hobin M. *The Attachment-Separation-Individuation (ASI) Scale (Revised)*. Pennsylvania: Family Centered Resource Project; 1982.

214. Green J, Stanley C, Smith V, Goldwyn R. A new method of evaluating attachment representations in the young school-age children: The Manchester Attachment Story Task (MCAST). *Attach Hum Dev* 2000;**2**:48–70. http://dx.doi.org/10.1080/146167300361318

215. Bretherton I, Ridgeway D, Cassidy J. Assessing Internal Working Models of the Attachment Relationship: An Attachment Story Completion Task for 3-Year-Olds. In Greenberg M, Cicchetti D, Cummings EM, editors. *Attachment in the Preschool Years*. Chicago, IL: University of Chicago Press; 1990. pp. 273–308.

216. Bayley N. *Manual for the Bayley Scales of Infant Development*. 2nd edn. New York, NY: Psychological Corp.; 1993.

217. Howes C, Ritchie S. Attachment organization in children with difficult life circumstances. *Dev Psychopathol* 1999;**11**:251–68. http://dx.doi.org/10.1017/S0954579499002047

218. Moran G, Pederson DR, Krupka A. Maternal unresolved attachment status impedes the effectiveness of interventions with adolescent mothers. *Infant Ment Health J* 2005;**26**:231–49. http://dx.doi.org/10.1002/imhj.20045

219. Zeanah C, Smyke A, Koga S, Carlson E. Attachment in institutionalized and non institutionalized Romanian Children. *Child Dev* 2005;**76**:1015–28. http://dx.doi.org/10.1111/j.1467-8624.2005.00894.x

220. O'Connor T, Bredenkamp D, Rutter M. The English and Romanian Adoptees (ERA) Study Team. Attachment disturbances and disorders in children exposed to early severe deprivation. *Infant Ment Health J* 1999;**20**:10–29.

221. Booth P, Jernberg A. *Theraplay: Helping Parents and Children Build Better Relationships Through Attachment-Based Play*. San Francisco, CA: Jossey-Bass; 2009.

222. Becker-Weidman. *The Dyadic Developmental Psychotherapy Casebook*. Lanham: MJA; 2011.

223. Cohen N, Muir E, Parker C, Brown M, Lojkasek M, Muir R, *et al.* Watch, wait, and wonder: testing the effectiveness of a new approach to mother-infant psychotherapy. *Infant Ment Health J* 1999;**20**:429–51. http://dx.doi.org/10.1002/(sici)1097-0355(199924)20:4<429::aid-imhj5>3.0.co;2-q

224. Centre for Reviews and Dissemination. *Making Cost-Effectiveness Information Available: The NHS Economic Evaluation Database Project*. York: University of York; 1996.

225. Drummond M. *Methods for the Economic Evaluation of Health Care Programmes*. New York, NY: Oxford University Press; 2005.

226. Richman N. *Communicating with Children. Helping Children in Distress*. London: Save the Children; 1993.

227. Psacharopoulos G, Patrinos HA. Returns to investment in education: a further update. *Educ Econ* 2004;**2**:111–34. http://dx.doi.org/10.1080/0964529042000239140

228. Brookes N. *Unit Costs in Criminal Justice (UCCJ): PSSRU Discussion Paper*. Canterbury: PSSRU, University of Kent; 2013.

229. Cooper D, McCausland W, Theodossiou I. The health hazards of unemployment and poor education: the socieoeconomic determinants of health duration in the European Union. *Econ Hum Biol* 2006;**3**:273–97. http://dx.doi.org/10.1016/j.ehb.2006.06.001

230. Liu H. Marital dissolution and self-rated health: age trajectories and birth cohort variations. *Soc Sci Med* 2012;**7**:1107–16. http://dx.doi.org/10.1016/j.socscimed.2011.11.037

231. Guralnik J. Educational status and active life expectancy among older blacks and whites. *N Engl J Med* 1993;**2**:110–16. http://dx.doi.org/10.1056/NEJM199307083290208

232. Groot W, Maassen van den Brink H. The health effects of education. *Econ Educ Rev* 2007;**26**:186–200. http://dx.doi.org/10.1016/j.econedurev.2005.09.002

233. Petticrew M, Sowden A, Lister-Sharp D, Wright K. False-negative results in screening programmes: systematic review of impact and implications. *Health Technol Assess* 2000;**4**(5).

234. Dozier M, Stovall K, Albus K, Bates B. Attachment for infants in foster care: the role of caregiver state of mind. *Child Dev* 2001;**72**:1467–77. http://dx.doi.org/10.1111/1467-8624.00360

235. Bakermans-Kranenburg M, Van IJzendoorn M, Kroonenberg P. Differences in attachment security between African-American and white children: ethnicity or socio-economic status? *Infant Behav Dev* 2004;**27**:417–33. http://dx.doi.org/10.1016/j.infbeh.2004.02.002

236. Minnis H, Macmillan S, Pritchett R, Young D, Wallace B, Butcher J, *et al.* Prevalence of reactive attachment disorder in a deprived population. *Br J Psychiatry* 2013;**202**:342–6. http://dx.doi.org/10.1192/bjp.bp.112.114074

237. Prichett R, Pritchett J, Marshall E, Davidson C, Minnis H. Reactive disorder in the general population: a Hidden ESSENCE Disorder. *Sci World J* 2013;**2013**:6. http://dx.doi.org/10.1155/2013/818157

238. Curtis L. *Unit Costs of Health and Social Care 2012*. Canterbury: PSSRU, University of Kent; 2012.

239. Central Intelligence Agency (CIA). *The World Factbook*. URL: www.cia.gov/library/publications/the-world-factbook/fields/2054.html (accessed 11 March 2013).

240. NHS England. *Proposed CCG Configuration and Member Practices Published*. NHS England; 2012. URL: www.england.nhs.uk/2012/05/24/ccg-configuration (accessed 8 April 2013).

241. The World Bank. *World Development Indicators*. URL: http://data.worldbank.org/data-catalog/world-development-indicators (accessed 14 January 2013).

242. Adoption UK. *Facts and Figures*. URL: www.adoptionuk.org.uk/information/103152/e_factsandfigs (accessed 14 January 2013).

243. World Health Organization. *Preventing Child Maltreatment: A Guide to Taking Action and Generating Evidence*. Geneva: World Health Organization; 2006.

244. Sroufe AL, Collins WA. *The Development of the Person: The Minnesota Study of Risk and Adaptation from Birth to Adulthood*. New York, NY: Guilford Press; 2009.

245. Rothbaum F, Weisz J, Pott M, Miyake K, Morelli G. Attachment and culture: security in the United States and Japan. *Am Psychol* 2000;**55**:1093.

246. Rutter M, Sroufe L. Developmental psychopathologyconcepts and challenges. *Dev Psychopathol* 2000;**12**:265–96. http://dx.doi.org/10.1017/S0954579400003023

247. Hazen N, Jacobvitz D, Higgins K, Allen S, Jin M. Pathways from Disorganized Attachment to later Social-Emotional Problems: The Role of Gender and Parentchild Interaction Patterns. In Solomon J, George C, editors. *Disorganized Attachment and Caregiving*. New York, NY: Guilford Press; 2011.

© Queen's Printer and Controller of HMSO 2015. This work was produced by Wright *et al.* under the terms of a commissioning contract issued by the Secretary of State for Health. This issue may be freely reproduced for the purposes of private research and study and extracts (or indeed, the full report) may be included in professional journals provided that suitable acknowledgement is made and the reproduction is not associated with any form of advertising. Applications for commercial reproduction should be addressed to: NIHR Journals Library, National Institute for Health Research, Evaluation, Trials and Studies Coordinating Centre, Alpha House, University of Southampton Science Park, Southampton SO16 7NS, UK.

248. Fearon R, Bakermans-Kranenburg M, Van IJzendoorn M, Lapsley A, Roisman G. The significance of insecure attachment and disorganization in the development of children's externalizing behavior: a meta-analytic study. *Child Dev* 2010;**81**:435–56. http://dx.doi.org/10.1111/j.1467-8624.2009.01405.x

249. Claxton K, Eggington S, Ginnelly L, Griffin S, McCabe C, Philips Z. *A Pilot Study of Value of Information Analysis to Support Research Recommendations for the National Institute for Health and Clinical Excellence*. York: Centre for Health Economics; University of York: 2005.

250. Belsky J, Fearon R. Infant-mother attachment security, contextual risk, and early development: a moderational analysis. *Dev Psychopathol* 2002;**14**:293–310. http://dx.doi.org/10.1017/S0954579402002067

251. Deklyen M, Greenberg M. Attachment and Psychopathology in Childhood. In Cassidy J, Shaver PR, editors. *Handbook of Attachment. Theory Research and Clinical Applications*. New York, NY: Guilford Press; 2008.

252. National Institute for Child Health and Development Early Child Care Research Network. The effects of infant child care on infant-mother attachment security: results of the NICHD Study of Early Child Care. *Child Dev* 1997;**68**:860–79.

253. Cassidy J, Ziv Y, Mehta, Feeney B. Feedback seeking in children and adolescent: association with self- perceptions, attachment representation and depression *Child Dev* 2003;**74**:612–28. http://dx.doi.org/10.1111/1467-8624.7402019

254. Vondra J, Shaw D, Surearinger L, Cohen M, Owens E. Attachment stability and emotional and behavioural regulation from infancy to preschool age. *Dev Psychopathol* 2001;**13**:13–33. http://dx.doi.org/10.1017/S095457940100102X

255. Lyons-Ruth K, Alpern L, Repacholi B. Disorganized infant attachment classification and maternal psychosocial problems as predictors of hostile-aggressive behavior in the preschool classroom. *Child Dev* 1993;**64**:572–85. http://dx.doi.org/10.2307/1131270

256. Spieker S, Bensley L. Roles of living arrangement and grandmother social support in adolescent mothering and infant attachment. *Dev Psychol* 1994;**30**:102–11. http://dx.doi.org/10.1037/0012-1649.30.1.102

257. El-Sheikh M, Elmore–Staton L. The link between marital conflict and child adjustment: parent-child conflict and perceived attachments as mediators, potentiators, and mitigators of risk. *Dev Psychopathol* 2004;**16**:631–48. http://dx.doi.org/10.1017/S0954579404004705

258. Cicchetti D, Carlson V. *Child Maltreatment: Theory and Research on the Causes and Consequences of Child Abuse and Neglect*. Cambridge: Cambridge University Press; 1989. http://dx.doi.org/10.1017/CBO9780511665707

259. Wille N, Badia X, Bonsel G, Burstro K, Cavrini G, Devlin N. Development of the EQ-5D-Y: a child friendly version. *Qual Life Res* 2010;**19**:875–86. http://dx.doi.org/10.1007/s11136-010-9648-y

260. Horsman J, Furlong W, Feeny D, Torrance G. The Health Utilities Index (HUI®): concepts, measurement properties and applications. *Health Qual Life Outcomes* 2003;**1**:54. http://dx.doi.org/10.1186/1477-7525-1-54

261. Shaw M, Hodgkins P, Caci H, Young S, Kahle J, Woods AG, *et al.* A systematic review and analysis of long-term outcomes in attention deficit hyperactivity disorder: effects of treatment and non-treatment. *BMC Med* 2012;**10**:99. http://dx.doi.org/10.1186/1741-7015-10-99

262. Michelson D, Davenport C, Dretzke J, Barlow J, Day C. Do evidence-based interventions work when tested in the 'real world?' a systematic review and meta-analysis of parent management training for the treatment of child disruptive behavior. *Clin Child Fam Psychol Rev* 2013;**16**:1–17. http://dx.doi.org/10.1007/s10567-013-0128-0

263. Waller R, Gardner F, Hyde L. What are the associations between parenting, callous-unemotional traits, and antisocial behavior in youth? A systematic review of evidence. *Clin Psychol Rev* 2013;**33**:593–608. http://dx.doi.org/10.1016/j.cpr.2013.03.001

264. Knerr W, Gardner F, Cluver L. Improving positive parenting skills and reducing harsh and abusive parenting in low-and middle-income countries: a systematic review. *Prev Sci* 2013:**14**;352–63. http://dx.doi.org/10.1007/s11121-012-0314-1

265. Van IJzendoorn M, Sagi-Schwartz A. Cross Cultural Patterns of Attachments. Universal and Contextual Dimensions. In Cassidy J, Shaver PR, editors. *Handbook of Attachment. Theory Research and Clinical Applications*. New York, NY: Guilford Press; 2008.

266. Department of Health. *Healthy Child Programme – Pregnancy and the First Five Years*. London: Department of Health; 2009.

267. Gini M, Oppenheim D, Sagi-Schwartz A. Negotiation styles in mother-child narrative co-construction in middle childhood: associations with early attachment. *Int J Behav Dev* 2007;**31**:149–60. http://dx.doi.org/10.1177/0165025407074626

268. Oppenheim D, Wamboldt F. Associations between 3-year-olds' narrative co-constructions with mothers and fathers and their story completions about affective themes. *Early Dev Parent* 1996;**5**:149–60. http://dx.doi.org/10.1002/(SICI)1099-0917(199609)5:3<149::AID-EDP127>3.0.CO;2-J

269. Achenbach TM. *Manual for the Child Behaviour Checklist: 4–18 and 1991 Profile*. Burlington, VT: University of Vermont; 1991.

270. Munson J, McMahon R, Spieker S. Structure and variability in the developmental trajectory of children's externalizing problems: impact of infant attachment, maternal depressive symptomatology, and child sex. *Dev Psychopathol* 2001;**13**:277–96. http://dx.doi.org/10.1017/S095457940100205X

271. Stams G, Juffer F, Van IJzendoorn M. Maternal sensitivity, infant attachment, and temperament in early childhood predict adjustment in middle childhood: the case of adopted children and their biologically unrelated parents. *Dev Psychol* 2002;**38**:806–21. http://dx.doi.org/10.1037/0012-1649.38.5.806

272. Ziv Y, Oppenheim D, Sagi-Schwartz A. Social information processing in middle childhood: relations to infant-mother attachment. *Attach Hum Dev* 2004;**6**:327–48. http://dx.doi.org/10.1080/14616730412331281511

273. Dodge KA, Price JM. On the relation between social information processing and socially competent behaviour in early school-aged children. *Child Dev* 1994;**65**:1385–97. http://dx.doi.org/10.2307/1131505

274. Santelices MP, Guzmán M, Aracena GM, Farkas C, Armijo I, Perés-Salas CP, *et al.* Promoting secure attachment: evaluation of the effectiveness of an early intervention pilot programme with mother–infant dyads in Santiago, Chile. *Child Care Health Dev* 2010;**37**:203–10. http://dx.doi.org/10.1111/j.1365-2214.2010.01161.x

© Queen's Printer and Controller of HMSO 2015. This work was produced by Wright *et al.* under the terms of a commissioning contract issued by the Secretary of State for Health. This issue may be freely reproduced for the purposes of private research and study and extracts (or indeed, the full report) may be included in professional journals provided that suitable acknowledgement is made and the reproduction is not associated with any form of advertising. Applications for commercial reproduction should be addressed to: NIHR Journals Library, National Institute for Health Research, Evaluation, Trials and Studies Coordinating Centre, Alpha House, University of Southampton Science Park, Southampton SO16 7NS, UK.

Appendix 1 Full search strategy

Assessment/diagnosis search strategies

PsycINFO (via OvidSP)

Date searched: 1806 to week 1, January 2012.

Date of search: 6 January 2012.

A total of 3776 records were retrieved.

Search strategy

1. attachment behavior/ (13,469)
2. attachment disorders/ (370)
3. attachment theory/ (885)
4. (attachment adj2 (disorder$1 or problem$1 or style$1 or pattern$1)).ti,ab. (4327)
5. (attachment adj2 (behavio?r$ or ambivalen$ or avoidant or diffuse or organi$ or disorgani$ or disrupt$ or abnormal$ or disinhib$ or inhib$)).ti,ab. (2562)
6. (attachment adj2 (disorienta$ or reactive or anxious$ or disturb$ or relation$)).ti,ab. (3582)
7. (attachment adj2 (interven$ or insecure$ or secure or security or early or theory or theories)).ti,ab. (7456)
8. or/1-7 (17,172)
9. exp measurement/ (233,204)
10. (sensitivity or specificity or predictive value$ or accurac$ or measurement$ or assess$ or diagnos$).ti,ab. (677,817)
11. 9 or 10 (793,368)
12. 8 and 11 (5273)
13. (comment reply or editorial or letter or reprint or "review book" or "review media" or "review software other").dt. (221,270)
14. (animal or animals or rat or rats or mouse or mice or hamster or hamsters or dog or dogs or cat or cats or bovine or sheep or ovine or pig or pigs).ab,ti,id,de. (232,200)
15. 12 not (13 or 14) (4926)
16. (infancy 2 23 mo or neonatal birth 1 mo or preschool age 2 5 yrs).ag. (113,808)
17. (adolescence 13 17 yrs or childhood birth 12 yrs or school age 6 12 yrs).ag. (524,966)
18. (child$ or infant$ or infancy or preschool$ or pre school$ or baby or babies or pediat$ or paediat$).ti,ab. (504,891)
19. (boy or boys or girl or girls).ti,ab. (69,790)
20. (schoolchild$ or adolescen$ or juvenile$ or youth$ or teenage$ or youngster$).ti,ab. (190,092)
21. (young people or young person or young persons or young adult$ or early adult$).ti,ab. (38,372)
22. exp Parents/ (62,079)
23. exp Parenting/ (64,945)
24. (parent$ or mother$ or maternal$ or mum$ or father$ or paternal$ or dad$).ti,ab. (242,087)
25. Dyads/ (3998)
26. dyad$.ti,ab. (18,706)
27. (attunement or (representation$ adj2 model$)).ti,ab. (1587)
28. exp Child Neglect/ or exp Child Abuse/ (21,046)
29. exp Foster Children/ or exp Foster Care/ or exp Foster Parents/ (4034)

© Queen's Printer and Controller of HMSO 2015. This work was produced by Wright *et al.* under the terms of a commissioning contract issued by the Secretary of State for Health. This issue may be freely reproduced for the purposes of private research and study and extracts (or indeed, the full report) may be included in professional journals provided that suitable acknowledgement is made and the reproduction is not associated with any form of advertising. Applications for commercial reproduction should be addressed to: NIHR Journals Library, National Institute for Health Research, Evaluation, Trials and Studies Coordinating Centre, Alpha House, University of Southampton Science Park, Southampton SO16 7NS, UK.

30. exp “Adoption (Child)”/ or exp Adoptive Parents/ (2891)
31. (neglect$ or abuse or abused or abusive or maltreat$ or mistreat$).ti,ab. (104,195)
32. (foster$ or adopt$).ti,ab. (69,814)
33. or/16-32 (955,446)
34. 15 and 33 (3776)

MEDLINE and MEDLINE In-Process & Other Non-Indexed Citations (via OvidSP)

Date searched: 1946 to week 4, December 2011.

Date of search: 9 January 2012.

A total of 699 records were retrieved in MEDLINE, and 28 in MEDLINE In-Process.

Search strategy

1. Reactive Attachment Disorder/ (296)
2. (attachment adj2 (disorder$1 or problem$1 or style$1 or pattern$1)).ti,ab. (1100)
3. (attachment adj2 (behavio?r$ or ambivalen$ or avoidant or diffuse or organi$ or disorgani$ or disrupt$ or abnormal$ or disinhib$ or inhib$)).ti,ab. (2250)
4. (attachment adj2 (disorienta$ or reactive or anxious$ or disturb$ or relation$)).ti,ab. (842)
5. (attachment adj2 (interven$ or insecure$ or secure or security or early or theory or theories)).ti,ab. (1795)
6. or/1-5 (4718)
7. (sensitiv$ or diagnos$).mp. or di.fs. [HEDGES diagnostic filter] (3,362,246)
8. 6 and 7 (981)
9. animals/ not (animals/ and humans/) (3,548,684)
10. (letter or editorial or comment or news or newspaper article).pt. (1,231,519)
11. 8 not (9 or 10) (925)
12. exp Child/ (1,40,0869)
13. exp Infant/ (854,319)
14. Adolescent/ (1,434,825)
15. (child$ or infant$ or infancy or preschool$ or pre school$ or baby or babies or pediat$ or paediat$).ti,ab. (1,125,683)
16. (boy or boys or girl or girls).ti,ab. (136,911)
17. (schoolchild$ or adolescen$ or juvenile$ or youth$ or teenage$ or youngster$).ti,ab. (214,069)
18. (young people or young person or young persons or young adult$ or early adult$).ti,ab. (60,337)
19. exp Parents/ (60,696)
20. exp Parent-Child Relations/ or Parenting/ (45,480)
21. (parent$ or mother$ or maternal$ or mum$ or father$ or paternal$ or dad$).ti,ab. (520,407)
22. dyad$.ti,ab. (7450)
23. (attunement or (representation$ adj2 model$)).ti,ab. (692)
24. Child Abuse/ (15,437)
25. Foster Home Care/ (2730)
26. Adoption/ (3984)
27. (neglect$ or abuse or abused or abusive or maltreat$ or mistreat$).ti,ab. (101,116)
28. (foster$ or adopt$).ti,ab. (116,855)
29. or/12-28 (3,282,233)
30. 11 and 29 (699)

EMBASE (via OvidSP)

Date searched: 1974 to week 1, 2012.

Date of search: 10 January 2012.

A total of 902 records were retrieved.

Search strategy

1. (attachment adj2 (disorder$1 or problem$1 or style$1 or pattern$1)).ti,ab. (1565)
2. (attachment adj2 (behavio?r$ or ambivalen$ or avoidant or diffuse or organi$ or disorgani$ or disrupt$ or abnormal$ or disinhib$ or inhib$)).ti,ab. (2670)
3. (attachment adj2 (disorienta$ or reactive or anxious$ or disturb$ or relation$)).ti,ab. (1148)
4. (attachment adj2 (interven$ or insecure$ or secure or security or early or theory or theories)).ti,ab. (2513)
5. or/1-4 (5956)
6. (predict$ or specificity).tw. or di.fs. [HEDGES diagnostic filter] (3,318,278)
7. 5 and 6 (1283)
8. Animal/ or Animal Experiment/ or Nonhuman/ (5,761,726)
9. (rat or rats or mouse or mice or murine or rodent or rodents or hamster or hamsters or pig or pigs or porcine or rabbit or rabbits or animal or animals or dogs or dog or cats or cow or bovine or sheep or ovine or monkey or monkeys).ti,ab,sh. (4,749,774)
10. 8 or 9 (6,446,779)
11. exp Human/ or Human Experiment/ (12,937,340)
12. 10 not (10 and 11) (5,116,251)
13. (editorial or letter or note).pt. (1,613,483)
14. 7 not (12 or 13) (1219)
15. child/ (1,135,530)
16. infant/ (476,014)
17. adolescent/ (1,127,803)
18. (child$ or infant$ or infancy or preschool$ or pre school$ or baby or babies or pediat$ or paediat$).ti,ab. (1,413,823)
19. (boy or boys or girl or girls).ti,ab. (177,580)
20. (schoolchild$ or adolescen$ or juvenile$ or youth$ or teenage$ or youngster$).ti,ab. (272,836)
21. (young people or young person or young persons or young adult$ or early adult$).ti,ab. (76,703)
22. exp parent/ (114,307)
23. exp child parent relation/ (58,704)
24. (parent$ or mother$ or maternal$ or mum$ or father$ or paternal$ or dad$).ti,ab. (911,848)
25. dyad$.ti,ab. (9350)
26. (attunement or (representation$ adj2 model$)).ti,ab. (894)
27. child abuse/ or child neglect/ (21,051)
28. foster care/ (3077)
29. adoption/ or adopted child/ (4815)
30. (neglect$ or abuse or abused or abusive or maltreat$ or mistreat$).ti,ab. (131,980)
31. (foster$ or adopt$).ti,ab. (150,939)
32. or/15-31 (3,567,803)
33. 14 and 32 (902)

© Queen's Printer and Controller of HMSO 2015. This work was produced by Wright *et al.* under the terms of a commissioning contract issued by the Secretary of State for Health. This issue may be freely reproduced for the purposes of private research and study and extracts (or indeed, the full report) may be included in professional journals provided that suitable acknowledgement is made and the reproduction is not associated with any form of advertising. Applications for commercial reproduction should be addressed to: NIHR Journals Library, National Institute for Health Research, Evaluation, Trials and Studies Coordinating Centre, Alpha House, University of Southampton Science Park, Southampton SO16 7NS, UK.

Social Policy & Practice (via OvidSP)

Date searched: inception to 2012.

Date of search: 10 January 2012.

A total of 343 records were retrieved.

Search strategy

1. attachment disorder.de. (232)
2. (attachment adj2 (disorder$1 or problem$1 or style$1 or pattern$1)).ti,ab. (390)
3. (attachment adj2 (behavio?r$ or ambivalen$ or avoidant or diffuse or organi$ or disorgani$ or disrupt$ or abnormal$ or disinhib$ or inhib$)).ti,ab. (196)
4. (attachment adj2 (disorienta$ or reactive or anxious$ or disturb$ or relation$)).ti,ab. (265)
5. (attachment adj2 (interven$ or insecure$ or secure or security or early or theory or theories)).ti,ab. (824)
6. or/1-5 (1309)
7. (diagnosis or diagnostic or assessment or measurement).de. (17,861)
8. (sensitivity or specificity or predictive value$ or accurac$ or measurement$ or assess$ or diagnos$).ti,ab. (51,582)
9. 7 or 8 (58,179)
10. and 9 (388)
11. (child$ or infant$ or infancy or preschool$ or pre school$ or baby or babies or pediat$ or paediat$).ti, ab,de. (125,901)
12. (boy or boys or girl or girls).ti,ab,de. (5692)
13. (schoolchild$ or adolescen$ or juvenile$ or youth$ or teenage$ or youngster$).ti,ab,de. (32,056)
14. (young people or young person or young persons or young adult$ or early adult$).ti,ab,de. (42,212)
15. (parent$ or mother$ or maternal$ or mum$ or father$ or paternal$ or dad$).ti,ab,de. (47,034)
16. dyad$.ti,ab,de. (480)
17. (attunement or (representation$ adj2 model$)).ti,ab,de. (66)
18. (neglect$ or abuse or abused or abusive or maltreat$ or mistreat$).ti,ab,de. (23,203)
19. (foster$ or adopt$).ti,ab,de. (19,994)
20. or/11-19 (172,573)
21. 10 and 20 (343)

Science Citation Index (SCI; via ISI Web of Science)

Date searched: 1899 to 6 January 2012.

Date of search: 10 January 2012.

A total of 404 records were retrieved.

Databases=SCI-EXPANDED Timespan=All Years.

Lemmatization=Off.

Search strategy

20 #18 NOT #19 (404)

19 TS=(rat or rats or mouse or mice or murine or hamster or hamsters or animal or animals or dogs or dog or pig or pigs or cats or bovine or cow or sheep or ovine or porcine or monkey) (3,408,525)

18 #7 AND #17 (429)

17 #8 OR #9 OR #10 OR #11 OR #12 OR #13 OR #14 OR #15 OR #16 (1,972,974)

16 TS=(foster* or adopt*) (207,538)

15 TS=(neglect* or abuse or abused or abusive or maltreat* or mistreat*) (117,173)

14 TS=(attunement or (representation* NEAR/2 model*)) (6475)

13 TS=dyad* (10,857)

12 TS=(parent* or mother* or maternal* or mum* or father* or paternal* or dad*) (574,227)

11 TS=("young people" or "young person" or "young persons" or "young adult*" or "early adult*") (55,963)

10 TS=(schoolchild* or adolescen* or juvenile* or youth* or teenage* or youngster*) (249,910)

9 TS=(boy or boys or girl or girls) (96,380)

8 TS=(child* or infant* or infancy or preschool* or "pre school*" or baby or babies or pediat* or paediat*) (1,021,056)

7 #6 AND #5 (917)

6 TS=(sensitivity or specificity or "predictive value*" or accuracy* or measurement* or assess* or diagnos*) (4,444,337)

5 #1 OR #2 OR #3 OR #4 (4095)

4 TS=(attachment NEAR/2 (intervene* or insecure* or secure or security or early or theory or theories)) (1054)

3 TS=(attachment NEAR/2 (disorienta* or reactive or anxious* or disturb* or relation*)) (593)

2 TS=(attachment NEAR/2 (behavior$r* or ambivalen* or avoidant or diffuse or organi* or disorgani* or disrupt* or abnormal* or disinhib* or inhib*)) (2203)

1 TS=(attachment NEAR/2 (disorder$ or problem$ or style$ or pattern$)) (924)

Social Science Citation Index (SSCI; via ISI Web of Science)

Date searched: 1956 to 6 January 2012.

Date of search: 10 January 2012.

A total of 1734 records were retrieved.

Databases=SSCI Timespan=All Years.

Lemmatization = Off.

© Queen's Printer and Controller of HMSO 2015. This work was produced by Wright *et al.* under the terms of a commissioning contract issued by the Secretary of State for Health. This issue may be freely reproduced for the purposes of private research and study and extracts (or indeed, the full report) may be included in professional journals provided that suitable acknowledgement is made and the reproduction is not associated with any form of advertising. Applications for commercial reproduction should be addressed to: NIHR Journals Library, National Institute for Health Research, Evaluation, Trials and Studies Coordinating Centre, Alpha House, University of Southampton Science Park, Southampton SO16 7NS, UK.

Search strategy

20 #18 NOT #19 (1734)

19 TS=(rat or rats or mouse or mice or murine or hamster or hamsters or animal or animals or dogs or dog or pig or pigs or cats or bovine or cow or sheep or ovine or porcine or monkey) (91,781)

18 #7 AND #17 (1752)

17 #8 OR #9 OR #10 OR #11 OR #12 OR #13 OR #14 OR #15 OR #16 (682,942)

16 TS=(foster* or adopt*) (75,821)

15 TS=(neglect* or abuse or abused or abusive or maltreat* or mistreat*) (91,084)

14 TS=(attunement or (representation* NEAR/2 model*)) (2062)

13 TS=dyad* (10,250)

12 TS=(parent* or mother* or maternal* or mum* or father* or paternal* or dad*) (160,559)

11 TS=("young people" or "young person" or "young persons" or "young adult*" or "early adult*") (35,541)

10 TS=(schoolchild* or adolescen* or juvenile* or youth* or teenage* or youngster*) (165,662)

9 TS=(boy or boys or girl or girls) (42,755)

8 TS=(child* or infant* or infancy or preschool* or "pre school*" or baby or babies or pediat* or paediat*) (392,420)

7 #5 AND #6 (2179)

6 TS=(sensitivity or specificity or "predictive value*" or accuracy* or measurement* or assess* or diagnos*) (503,069)

5 #1 OR #2 OR #3 OR #4 (6315)

4 TS=(attachment NEAR/2 (intervene* or insecure* or secure or security or early or theory or theories)) (3614)

3 TS=(attachment NEAR/2 (disorienta* or reactive or anxious* or disturb* or relation*)) (2147)

2 TS=(attachment NEAR/2 (behavior$r* or ambivalen* or avoidant or diffuse or organi* or disorgani* or disrupt* or abnormal* or disinhib* or inhib*)) (1017)

1 TS=(attachment NEAR/2 (disorder$ or problem$ or style$ or pattern$)) (2646)

Conference Proceedings Citation Index – Science (CPCI-S; via ISI Web of Science)

Date searched: 1990 to 6 January 2012.

Date of search: 10 January 2012.

A total of 15 records were retrieved.

Databases=CPCI-S Timespan=All Years.

Lemmatization = Off.

Search strategy

20 #18 NOT #19 (15)

19 TS=(rat or rats or mouse or mice or murine or hamster or hamsters or animal or animals or dogs or dog or pig or pigs or cats or bovine or cow or sheep or ovine or porcine or monkey) (282,876)

18 #7 AND #17 (17)

17 #8 OR #9 OR #10 OR #11 OR #12 OR #13 OR #14 OR #15 OR #16 (291,238)

16 TS=(foster* or adopt*) (87,891)

15 TS=(neglect* or abuse or abused or abusive or maltreat* or mistreat*) (17,504)

14 TS=(attunement or (representation* NEAR/2 model*)) (3792)

13 TS=dyad* (1817)

12 TS=(parent* or mother* or maternal* or mum* or father* or paternal* or dad*) (70,471)

11 TS=("young people" or "young person" or "young persons" or "young adult*" or "early adult*") (5160)

10 TS=(schoolchild* or adolescen* or juvenile* or youth* or teenage* or youngster*) (24,476)

9 TS=(boy or boys or girl or girls) (5857)

8 TS=(child* or infant* or infancy or preschool* or "pre school*" or baby or babies or pediat* or paediat*) (104,538)

7 #5 AND #6 (54)

6 TS=(sensitivity or specificity or "predictive value*" or accuracy* or measurement* or assess* or diagnos*) (922,906)

5 #1 OR #2 OR #3 OR #4 (343)

4 TS=(attachment NEAR/2 (intervene* or insecure* or secure or security or early or theory or theories)) (93)

© Queen's Printer and Controller of HMSO 2015. This work was produced by Wright *et al.* under the terms of a commissioning contract issued by the Secretary of State for Health. This issue may be freely reproduced for the purposes of private research and study and extracts (or indeed, the full report) may be included in professional journals provided that suitable acknowledgement is made and the reproduction is not associated with any form of advertising. Applications for commercial reproduction should be addressed to: NIHR Journals Library, National Institute for Health Research, Evaluation, Trials and Studies Coordinating Centre, Alpha House, University of Southampton Science Park, Southampton SO16 7NS, UK.

3 TS=(attachment NEAR/2 (disorienta* or reactive or anxious* or disturb* or relation*)) (54)

2 TS=(attachment NEAR/2 (behavior$r* or ambivalen* or avoidant or diffuse or organi* or disorgani* or disrupt* or abnormal* or disinhib* or inhib*)) (141)

1 TS=(attachment NEAR/2 (disorder$ or problem$ or style$ or pattern$)) (95)

Conference Proceedings Citation Index – Social Science & Humanities (CPCI-SSH; via ISI Web of Science)

Date searched: 1990 to 6 January 2012.

Date of search: 10 January 2012.

A total of 91 records were retrieved.

Databases=CPCI-SSH Timespan=All Years.

Lemmatization=Off.

Search strategy

20 #18 NOT #19 (91)

19 TS=(rat or rats or mouse or mice or murine or hamster or hamsters or animal or animals or dogs or dog or pig or pigs or cats or bovine or cow or sheep or ovine or porcine or monkey) (4408)

18 #7 AND #17 (91)

17 #8 OR #9 OR #10 OR #11 OR #12 OR #13 OR #14 OR #15 OR #16 (45,707)

16 TS=(foster* or adopt*) (13,580)

15 TS=(neglect* or abuse or abused or abusive or maltreat* or mistreat*) (5468)

14 TS=(attunement or (representation* NEAR/2 model*)) (356)

13 TS=dyad* (832)

12 TS=(parent* or mother* or maternal* or mum* or father* or paternal* or dad*) (8462)

11 TS=("young people" or "young person" or "young persons" or "young adult*" or "early adult*") (1874)

10 TS=(schoolchild* or adolescen* or juvenile* or youth* or teenage* or youngster*) (7388)

9 TS=(boy or boys or girl or girls) (2090)

8 TS=(child* or infant* or infancy or preschool* or "pre school*" or baby or babies or pediat* or paediat*) (19,667)

7 #5 AND #6 (113)

6 TS=(sensitivity or specificity or "predictive value*" or accuracy* or measurement* or assess* or diagnos*) (43,437)

5 #1 OR #2 OR #3 OR #4 (423)

4 TS=(attachment NEAR/2 (intervene* or insecure* or secure or security or early or theory or theories)) (251)

3 TS=(attachment NEAR/2 (disorienta* or reactive or anxious* or disturb* or relation*)) (170)

2 TS=(attachment NEAR/2 (behavior$r* or ambivalen* or avoidant or diffuse or organi* or disorgani* or disrupt* or abnormal* or disinhib* or inhib*)) (83)

1 TS=(attachment NEAR/2 (disorder$ or problem$ or style$ or pattern$)) (143)

Education Resources Information Center (ERIC; via ProQuest)

Date searched: 1966 to December 2011.

Date of search: 11 January 2012.

A total of 717 records were retrieved.

Search strategy

S1 (su((“Attachment Behavior”)) OR TI,AB(attachment NEAR/2 (disorder[*1] OR problem[*1] OR style[*1] OR pattern[*1]))) OR TI,AB(attachment NEAR/2 (behavio*r* or ambivalen* or avoidant or diffuse or organi* or disorgani* or disrupt* or abnormal* or disinhib* or inhib*)) OR TI,AB(attachment NEAR/2 (disorienta* or reactive or anxious* or disturb* or relation* or interven* or insecure* or secure or security or early or theory or theories))) 2909*

S2 su((“Measurement” OR “Predictive Measurement”)) OR TI,AB(sensitivity or specificity or “predictive value*” or accurac* or measurement* or assess* or diagnos*) 206,218*

S3 S1 and S2 717*

Social Services Abstracts (via CSA Illumina)

Date searched: 1979 to December 2011.

Date of search: 11 January 2012.

A total of 141 records were retrieved.

Search strategy

((KW=((attachment within 2 (disorder* or problem* or style* or pattern*)) or (attachment within 2 (behavior* or behavior* or ambivalen* or avoidant or diffuse or organi* or disorgani* or disrupt* or abnormal* or disinhib* or inhib*)) or (attachment within 2 (disorienta* or reactive or anxious* or disturb* or relation*))) or KW=(attachment within 2 (interven* or insecure* or secure or security or early or theory or theories))) and(DE=(diagnosis or measurement) or KW=(sensitivity or specificity or predictive value* or accurac* or measurement* or assess* or diagnos*))) and((DE=(“adolescents” or “children” or “infants”)) or(KW=((child* or infant* or infancy or preschool* or “pre school*” or baby or babies or pediat* or paediat*) or (boy or boys or girl or girls) or (schoolchild* or adolescen* or juvenile* or youth* or teenage* or youngster*)) or KW=(“young people” or “young person” or “young persons” or “young adult*” or “early adult*”)) or(DE=(Dyads or Child Neglect or Child Abuse or Foster Care or Foster Children or Adoption or Adopted Children) or KW=((parent* or mother* or maternal* or mum* or father* or paternal* or dad*) or (dyad* or attunement) or (representation* within 2 model*)) or KW=((neglect* or abuse or abused or abusive or maltreat* or mistreat*) or (foster* or adopt*))))

© Queen’s Printer and Controller of HMSO 2015. This work was produced by Wright *et al.* under the terms of a commissioning contract issued by the Secretary of State for Health. This issue may be freely reproduced for the purposes of private research and study and extracts (or indeed, the full report) may be included in professional journals provided that suitable acknowledgement is made and the reproduction is not associated with any form of advertising. Applications for commercial reproduction should be addressed to: NIHR Journals Library, National Institute for Health Research, Evaluation, Trials and Studies Coordinating Centre, Alpha House, University of Southampton Science Park, Southampton SO16 7NS, UK.

Applied Social Sciences Index and Abstracts (ASSIA; via CSA Illumina)

Date searched: 1987 to December 2011.

Date of search: 11 January 2012.

A total of 469 records were retrieved.

Search strategy

((KW=((attachment within 2 (disorder* or problem* or style* or pattern*)) or (attachment within 2 (behavior* or behavior* or ambivalen* or avoidant or diffuse or organi* or disorgani* or disrupt* or abnormal* or disinhib* or inhib*)) or (attachment within 2 (disorienta* or reactive or anxious* or disturb* or relation*))) or KW=(attachment within 2 (interven* or insecure* or secure or security or early or theory or theories))) or(DE="attachment disorders")) and(DE=((Children or Infants or Adolescents) or (Parents or Dyads) or (Child neglect or Child abuse or Foster Care or Foster children or Adoption or Adopted children or Adoptive parents)) or KW=((child* or infant* or infancy or preschool* or "pre school*" or baby or babies or pediat* or paediat*) or (boy or boys or girl or girls) or (schoolchild* or adolescen* or juvenile* or youth* or teenage* or youngster* or "young people" or "young person" or "young persons" or "young adult*" or "early adult*")) or KW=((parent* or mother* or maternal* or mum* or father* or paternal* or dad* or dyad* or attunement) or (representation* within 2 model*) or (neglect* or abuse or abused or abusive or maltreat* or mistreat* or foster* or adopt*))) and(DE=(Assessment or Measurement or Diagnosis) or KW=(sensitivity or specificity or "predictive value*" or accurac* or measurement* or assess* or diagnos*))

Social Care Online (via SCIE)

Date searched: inception to 2012.

Date of search: 12 January 2012.

A total of 285 records were retrieved.

Advanced search option

(topic"attachment" or freetext="attachment") AND (topic="children" or topic="babies" or topic="young people" or topic="child abuse" or topic="child neglect" or topic="adoption" or topic="adoptive parents" or topic="adoptive children" or topic="foster care" or topic="foster children" or freetext="child*" or freetext="infant*" or freetext="infancy" or freetext="preschool*" or freetext="pre school*" or freetext="baby" or freetext="babies" or freetext="pediat*" or freetext="paediat*" or freetext="juvenile*" or freetext="youth*" or freetext="teenage*" or freetext="youngster*" or freetext="young people" or freetext="young person" or freetext="young persons" or freetext="young adult*" or freetext="early adult") AND (topic="assessment" or topic="diagnosis" or topic="performance measurement" or freetext="sensitivity" or freetext="specificity" or freetext="predictive value*" or freetext="accuracy*" or freetext="measurement*" or freetext="assess*" or freetext="diagnos*")

Epidemiology/natural history search strategies

PsycINFO (via OvidSP)

Date searched: 1806 to week 1, January 2012.

Date of search: 6 January 2012.

A total of 2450 records were retrieved.

Search strategy

1. attachment behavior/ (13,469)
2. attachment disorders/ (370)
3. attachment theory/ (885)
4. (attachment adj2 (disorder$1 or problem$1 or style$1 or pattern$1)).ti,ab. (4327)
5. (attachment adj2 (behavio?r$ or ambivalen$ or avoidant or diffuse or organi$ or disorgani$ or disrupt$ or abnormal$ or disinhib$ or inhib$)).ti,ab. (2562)
6. (attachment adj2 (disorienta$ or reactive or anxious$ or disturb$ or relation$)).ti,ab. (3582)
7. (attachment adj2 (interven$ or insecure$ or secure or security or early or theory or theories)).ti,ab. (7456)
8. or/1-7 (17,172)
9. exp Epidemiology/ (31,806)
10. Patient History/ or Family History/ (5654)
11. risk factors/ (30,662)
12. (epidemiol$ or incidence or prevalence or history or risk$1 or long term).ti,ab. (419,210)
13. or/9-12 (426,083)
14. 8 and 13 (3150)
15. (comment reply or editorial or letter or reprint or "review book" or "review media" or "review software other").dt. (221,270)
16. (animal or animals or rat or rats or mouse or mice or hamster or hamsters or dog or dogs or cat or cats or bovine or sheep or ovine or pig or pigs).ab,ti,id,de. (232,200)
17. 14 not (15 or 16) (2897)
18. (infancy 2 23 mo or neonatal birth 1 mo or preschool age 2 5 yrs).ag. (113,808)
19. (adolescence 13 17 yrs or childhood birth 12 yrs or school age 6 12 yrs).ag. (524,966)
20. (child$ or infant$ or infancy or preschool$ or pre school$ or baby or babies or pediat$ or paediat$).ti,ab. (504,891)
21. (boy or boys or girl or girls).ti,ab. (69,790)
22. (schoolchild$ or adolescen$ or juvenile$ or youth$ or teenage$ or youngster$).ti,ab. (190,092)
23. (young people or young person or young persons or young adult$ or early adult$).ti,ab. (38,372)
24. exp Parents/ (62,079)
25. exp Parenting/ (64,945)
26. (parent$ or mother$ or maternal$ or mum$ or father$ or paternal$ or dad$).ti,ab. (242,087)
27. Dyads/ (3998)
28. dyad$.ti,ab. (18,706)
29. (attunement or (representation$ adj2 model$)).ti,ab. (1587)
30. exp Child Neglect/ or exp Child Abuse/ (21,046)
31. exp Foster Children/ or exp Foster Care/ or exp Foster Parents/ (4034)
32. exp "Adoption (Child)"/ or exp Adoptive Parents/ (2891)
33. (neglect$ or abuse or abused or abusive or maltreat$ or mistreat$).ti,ab. (104,195)
34. (foster$ or adopt$).ti,ab. (69,814)
35. or/18-34 (955,446)
36. 17 and 35 (2450)

© Queen's Printer and Controller of HMSO 2015. This work was produced by Wright *et al.* under the terms of a commissioning contract issued by the Secretary of State for Health. This issue may be freely reproduced for the purposes of private research and study and extracts (or indeed, the full report) may be included in professional journals provided that suitable acknowledgement is made and the reproduction is not associated with any form of advertising. Applications for commercial reproduction should be addressed to: NIHR Journals Library, National Institute for Health Research, Evaluation, Trials and Studies Coordinating Centre, Alpha House, University of Southampton Science Park, Southampton SO16 7NS, UK.

MEDLINE and MEDLINE In-Process & Other Non-Indexed Citations (via OvidSP)

Date searched: 1946 to week 4, December 2011.

Date of search: 9 January 2012.

A total of 710 records were retrieved in MEDLINE, and 45 in MEDLINE In-Process.

Search strategy

1. Reactive Attachment Disorder/ (296)
2. (attachment adj2 (disorder$1 or problem$1 or style$1 or pattern$1)).ti,ab. (1100)
3. (attachment adj2 (behavio?r$ or ambivalen$ or avoidant or diffuse or organi$ or disorgani$ or disrupt$ or abnormal$ or disinhib$ or inhib$)).ti,ab. (2250)
4. (attachment adj2 (disorienta$ or reactive or anxious$ or disturb$ or relation$)).ti,ab. (842)
5. (attachment adj2 (interven$ or insecure$ or secure or security or early or theory or theories)).ti,ab. (1795)
6. or/1-5 (4718)
7. Epidemiology/ (10,988)
8. Incidence/ (147,388)
9. Prevalence/ (152,723)
10. Medical History Taking/ (15,586)
11. Risk Factors/ (463,665)
12. (epidemiol$ or incidence or prevalence or history or risk$1 or long term).ti,ab. (2,138,567)
13. or/7-12 (2,338,046)
14. 6 and 13 (889)
15. animals/ not (animals/ and humans/) (3,548,684)
16. (letter or editorial or comment or news or newspaper article).pt. (1,231,519)
17. 14 not (15 or 16) (862)
18. exp Child/ (1,40,0869)
19. exp Infant/ (854,319)
20. Adolescent/ (1,434,825)
21. (child$ or infant$ or infancy or preschool$ or pre school$ or baby or babies or pediat$ or paediat$).ti,ab. (1,125,683)
22. (boy or boys or girl or girls).ti,ab. (136,911)
23. (schoolchild$ or adolescen$ or juvenile$ or youth$ or teenage$ or youngster$).ti,ab. (214,069)
24. (young people or young person or young persons or young adult$ or early adult$).ti,ab. (60,337)
25. exp Parents/ (60,696)
26. exp Parent-Child Relations/ or Parenting/ (45,480)
27. (parent$ or mother$ or maternal$ or mum$ or father$ or paternal$ or dad$).ti,ab. (520,407)
28. dyad$.ti,ab. (7450)
29. (attunement or (representation$ adj2 model$)).ti,ab. (692)
30. Child Abuse/ (15,437)
31. Foster Home Care/ (2730)
32. Adoption/ (3984)
33. (neglect$ or abuse or abused or abusive or maltreat$ or mistreat$).ti,ab. (101,116)
34. (foster$ or adopt$).ti,ab. (116,855)
35. or/18-34 (3,282,233)
36. 17 and 35 (710)

EMBASE (via OvidSP)

Date searched: 1974 to week 1, 2012.

Date of search: 10 January 2012.

A total of 912 records were retrieved.

Search strategy

1. (attachment adj2 (disorder$1 or problem$1 or style$1 or pattern$1)).ti,ab. (1565)
2. (attachment adj2 (behavio?r$ or ambivalen$ or avoidant or diffuse or organi$ or disorgani$ or disrupt$ or abnormal$ or disinhib$ or inhib$)).ti,ab. (2670)
3. (attachment adj2 (disorienta$ or reactive or anxious$ or disturb$ or relation$)).ti,ab. (1148)
4. (attachment adj2 (interven$ or insecure$ or secure or security or early or theory or theories)).ti,ab. (2513)
5. or/1-4 (5956)
6. epidemiology/ (157,086)
7. incidence/ (175,251)
8. prevalence/ (273,754)
9. medical history/ (9434)
10. risk factor/ (480,128)
11. (epidemiol$ or incidence or prevalence or history or risk$1 or long term).ti,ab. (2,789,649)
12. or/6-11 (3,097,650)
13. 5 and 12 (1180)
14. Animal/ or Animal Experiment/ or Nonhuman/ (5,761,726)
15. (rat or rats or mouse or mice or murine or rodent or rodents or hamster or hamsters or pig or pigs or porcine or rabbit or rabbits or animal or animals or dogs or dog or cats or cow or bovine or sheep or ovine or monkey or monkeys).ti,ab,sh. (4,749,774)
16. 14 or 15 (6,446,779)
17. exp Human/ or Human Experiment/ (12,937,340)
18. 16 not (16 and 17) (5,116,251)
19. (editorial or letter or note).pt. (1,613,483)
20. 13 not (18 or 19) (1138)
21. child/ (1,135,530)
22. infant/ (476,014)
23. adolescent/ (1,127,803)
24. (child$ or infant$ or infancy or preschool$ or pre school$ or baby or babies or pediat$ or paediat$).ti,ab. (1,413,823)
25. (boy or boys or girl or girls).ti,ab. (177,580)
26. (schoolchild$ or adolescen$ or juvenile$ or youth$ or teenage$ or youngster$).ti,ab. (272,836)
27. (young people or young person or young persons or young adult$ or early adult$).ti,ab. (76,703)
28. exp parent/ (114,307)
29. exp child parent relation/ (58,704)
30. (parent$ or mother$ or maternal$ or mum$ or father$ or paternal$ or dad$).ti,ab. (911,848)
31. dyad$.ti,ab. (9350)
32. (attunement or (representation$ adj2 model$)).ti,ab. (894)
33. child abuse/ or child neglect/ (21,051)
34. foster care/ (3077)
35. adoption/ or adopted child/ (4815)
36. (neglect$ or abuse or abused or abusive or maltreat$ or mistreat$).ti,ab. (131,980)
37. (foster$ or adopt$).ti,ab. (150,939)
38. or/21-37 (3,567,803)
39. 20 and 38 (912)

© Queen's Printer and Controller of HMSO 2015. This work was produced by Wright *et al.* under the terms of a commissioning contract issued by the Secretary of State for Health. This issue may be freely reproduced for the purposes of private research and study and extracts (or indeed, the full report) may be included in professional journals provided that suitable acknowledgement is made and the reproduction is not associated with any form of advertising. Applications for commercial reproduction should be addressed to: NIHR Journals Library, National Institute for Health Research, Evaluation, Trials and Studies Coordinating Centre, Alpha House, University of Southampton Science Park, Southampton SO16 7NS, UK.

Social Policy & Practice (via OvidSP)

Date searched: inception to 2012.

Date of search: 10 January 2012.

A total of 281 records were retrieved.

Search strategy

1. attachment disorder.de. (232)
2. (attachment adj2 (disorder$1 or problem$1 or style$1 or pattern$1)).ti,ab. (390)
3. (attachment adj2 (behavio?r$ or ambivalen$ or avoidant or diffuse or organi$ or disorgani$ or disrupt$ or abnormal$ or disinhib$ or inhib$)).ti,ab. (196)
4. (attachment adj2 (disorienta$ or reactive or anxious$ or disturb$ or relation$)).ti,ab. (265)
5. (attachment adj2 (interven$ or insecure$ or secure or security or early or theory or theories)).ti,ab. (824)
6. or/1-5 (1309)
7. (epidemiol$ or incidence or prevalence or history or risk$1 or long term).ti,ab,de. (51,779)
8. 6 and 7 (298)
9. (child$ or infant$ or infancy or preschool$ or pre school$ or baby or babies or pediat$ or paediat$).ti,ab,de. (125,901)
10. (boy or boys or girl or girls).ti,ab,de. (5692)
11. (schoolchild$ or adolescen$ or juvenile$ or youth$ or teenage$ or youngster$).ti,ab,de. (32,056)
12. (young people or young person or young persons or young adult$ or early adult$).ti,ab,de. (42,212)
13. (parent$ or mother$ or maternal$ or mum$ or father$ or paternal$ or dad$).ti,ab,de. (47,034)
14. dyad$.ti,ab,de. (480)
15. (attunement or (representation$ adj2 model$)).ti,ab,de. (66)
16. (neglect$ or abuse or abused or abusive or maltreat$ or mistreat$).ti,ab,de. (23,203)
17. (foster$ or adopt$).ti,ab,de. (19,994)
18. or/9-17 (172,573)
19. 8 and 18 (281)

Science Citation Index (SCI; via ISI Web of Science)

Date searched: 1899 to 6 January 2012.

Date of search: 10 January 2012.

A total of 352 records were retrieved.

Databases=SCI-EXPANDED Timespan=All Years.

Lemmatization=Off.

Search strategy

20 #18 NOT #19 (352)

19 TS=(rat or rats or mouse or mice or murine or hamster or hamsters or animal or animals or dogs or dog or pig or pigs or cats or bovine or cow or sheep or ovine or porcine or monkey) (3,408,525)

18 #7 AND #17 (380)

17 #8 OR #9 OR #10 OR #11 OR #12 OR #13 OR #14 OR #15 OR #16 (1,972,974)

16 TS=(foster* or adopt*) (207,538)

15 TS=(neglect* or abuse or abused or abusive or maltreat* or mistreat*) (117,173)

14 TS=(attunement or (representation* NEAR/2 model*)) (6475)

13 TS=dyad* (10,857)

12 TS=(parent* or mother* or maternal* or mum* or father* or paternal* or dad*) (574,227)

11 TS=("young people" or "young person" or "young persons" or "young adult*" or "early adult*") (55,963)

10 TS=(schoolchild* or adolescen* or juvenile* or youth* or teenage* or youngster*) (249,910)

9 TS=(boy or boys or girl or girls) (96,380)

8 TS=(child* or infant* or infancy or preschool* or "pre school*" or baby or babies or pediat* or paediat*) (1,021,056)

7 #5 AND #6 (575)

6 TS=(epidemiol* or incidence or prevalence or history or risk$ or long term) (2,405,624)

5 #1 OR #2 OR #3 OR #4 (4095)

4 TS=(attachment NEAR/2 (intervene* or insecure* or secure or security or early or theory or theories)) (1054)

3 TS=(attachment NEAR/2 (disorienta* or reactive or anxious* or disturb* or relation*)) (593)

2 TS=(attachment NEAR/2 (behavior$r* or ambivalen* or avoidant or diffuse or organi* or disorgani* or disrupt* or abnormal* or disinhib* or inhib*)) (2203)

1 TS=(attachment NEAR/2 (disorder$ or problem$ or style$ or pattern$)) (924)

Social Science Citation Index (SSCI; via ISI Web of Science)

Date searched: 1956 to 6 January 2012.

Date of search: 10 January 2012.

A total of 1226 records were retrieved.

Databases=SSCI Timespan=All Years.

Lemmatization=Off.

Search strategy

20 #18 NOT #19 (1226)

19 TS=(rat or rats or mouse or mice or murine or hamster or hamsters or animal or animals or dogs or dog or pig or pigs or cats or bovine or cow or sheep or ovine or porcine or monkey) (91,781)

18 #7 AND #17 (1250)

© Queen's Printer and Controller of HMSO 2015. This work was produced by Wright *et al.* under the terms of a commissioning contract issued by the Secretary of State for Health. This issue may be freely reproduced for the purposes of private research and study and extracts (or indeed, the full report) may be included in professional journals provided that suitable acknowledgement is made and the reproduction is not associated with any form of advertising. Applications for commercial reproduction should be addressed to: NIHR Journals Library, National Institute for Health Research, Evaluation, Trials and Studies Coordinating Centre, Alpha House, University of Southampton Science Park, Southampton SO16 7NS, UK.

17 #8 OR #9 OR #10 OR #11 OR #12 OR #13 OR #14 OR #15 OR #16 (682,942)

16 TS=(foster* or adopt*) (75,821)

15 TS=(neglect* or abuse or abused or abusive or maltreat* or mistreat*) (91,084)

14 TS=(attunement or (representation* NEAR/2 model*)) (2062)

13 TS=dyad* (10,250)

12 TS=(parent* or mother* or maternal* or mum* or father* or paternal* or dad*) (160,559)

11 TS=("young people" or "young person" or "young persons" or "young adult*" or "early adult*") (35,541)

10 TS=(schoolchild* or adolescen* or juvenile* or youth* or teenage* or youngster*) (165,662)

9 TS=(boy or boys or girl or girls) (42,755)

8 TS=(child* or infant* or infancy or preschool* or "pre school*" or baby or babies or pediat* or paediat*) (392,420)

7 #5 AND #6 (1437)

6 TS=(epidemiol* or incidence or prevalence or history or risk$ or long term) (536,531)

5 #1 OR #2 OR #3 OR #4 (6315)

4 TS=(attachment NEAR/2 (intervene* or insecure* or secure or security or early or theory or theories)) (3614)

3 TS=(attachment NEAR/2 (disorienta* or reactive or anxious* or disturb* or relation*)) (2147)

2 TS=(attachment NEAR/2 (behavior$r* or ambivalen* or avoidant or diffuse or organi* or disorgani* or disrupt* or abnormal* or disinhib* or inhib*)) (1017)

1 TS=(attachment NEAR/2 (disorder$ or problem$ or style$ or pattern$)) (2646)

Conference Proceedings Citation Index – Science (CPCI-S; via ISI Web of Science)

Date searched: 1990 to 6 January 2012.

Date of search: 10 January 2012.

A total of 18 records were retrieved.

Databases=CPCI-S Timespan=All Years.

Lemmatization=Off.

Search strategy

20 #18 NOT #19 (18)

19 TS=(rat or rats or mouse or mice or murine or hamster or hamsters or animal or animals or dogs or dog or pig or pigs or cats or bovine or cow or sheep or ovine or porcine or monkey) (282,876)

18 #7 AND #17 (22)

17 #8 OR #9 OR #10 OR #11 OR #12 OR #13 OR #14 OR #15 OR #16 (291,238)

16 TS=(foster* or adopt*) (87,891)

15 TS=(neglect* or abuse or abused or abusive or maltreat* or mistreat*) (17,504)

14 TS=(attunement or (representation* NEAR/2 model*)) (3792)

13 TS=dyad* (1817)

12 TS=(parent* or mother* or maternal* or mum* or father* or paternal* or dad*) (70,471)

11 TS=("young people" or "young person" or "young persons" or "young adult*" or "early adult*") (5160)

10 TS=(schoolchild* or adolescen* or juvenile* or youth* or teenage* or youngster*) (24,476)

9 TS=(boy or boys or girl or girls) (5857)

8 TS=(child* or infant* or infancy or preschool* or "pre school*" or baby or babies or pediat* or paediat*) (104,538)

7 #5 AND #6 (45)

6 TS=(epidemiol* or incidence or prevalence or history or risk$ or long term) (362,031)

5 #1 OR #2 OR #3 OR #4 (343)

4 TS=(attachment NEAR/2 (intervene* or insecure* or secure or security or early or theory or theories)) (93)

3 TS=(attachment NEAR/2 (disorienta* or reactive or anxious* or disturb* or relation*)) (54)

2 TS=(attachment NEAR/2 (behavior$r* or ambivalen* or avoidant or diffuse or organi* or disorgani* or disrupt* or abnormal* or disinhib* or inhib*)) (141)

1 TS=(attachment NEAR/2 (disorder$ or problem$ or style$ or pattern$)) (95)

© Queen's Printer and Controller of HMSO 2015. This work was produced by Wright *et al.* under the terms of a commissioning contract issued by the Secretary of State for Health. This issue may be freely reproduced for the purposes of private research and study and extracts (or indeed, the full report) may be included in professional journals provided that suitable acknowledgement is made and the reproduction is not associated with any form of advertising. Applications for commercial reproduction should be addressed to: NIHR Journals Library, National Institute for Health Research, Evaluation, Trials and Studies Coordinating Centre, Alpha House, University of Southampton Science Park, Southampton SO16 7NS, UK.

Conference Proceedings Citation Index – Social Science & Humanities (CPCI-SSH; via ISI Web of Science)

Date searched: 1990 to 6 January 2012.

Date of search: 10 January 2012.

A total of 73 records were retrieved.

Databases=CPCI-SSH Timespan=All Years.

Lemmatization=Off.

Search strategy

20 #18 NOT #19 (73)

19 TS=(rat or rats or mouse or mice or murine or hamster or hamsters or animal or animals or dogs or dog or pig or pigs or cats or bovine or cow or sheep or ovine or porcine or monkey) (4408)

18 #7 AND #17 (74)

17 #8 OR #9 OR #10 OR #11 OR #12 OR #13 OR #14 OR #15 OR #16 (45,707)

16 TS=(foster* or adopt*) (13,580)

15 TS=(neglect* or abuse or abused or abusive or maltreat* or mistreat*) (5468)

14 TS=(attunement or (representation* NEAR/2 model*)) (356)

13 TS=dyad* (832)

12 TS=(parent* or mother* or maternal* or mum* or father* or paternal* or dad*) (8462)

11 TS=("young people" or "young person" or "young persons" or "young adult*" or "early adult*") (1874)

10 TS=(schoolchild* or adolescen* or juvenile* or youth* or teenage* or youngster*) (7388)

9 TS=(boy or boys or girl or girls) (2090)

8 TS=(child* or infant* or infancy or preschool* or "pre school*" or baby or babies or pediat* or paediat*) (19,667)

7 #5 AND #6 (88)

6 TS=(epidemiol* or incidence or prevalence or history or risk$ or long term) (41,725)

5 #1 OR #2 OR #3 OR #4 (423)

4 TS=(attachment NEAR/2 (intervene* or insecure* or secure or security or early or theory or theories)) (251)

3 TS=(attachment NEAR/2 (disorienta* or reactive or anxious* or disturb* or relation*)) (170)

2 TS=(attachment NEAR/2 (behavior$r* or ambivalen* or avoidant or diffuse or organi* or disorgani* or disrupt* or abnormal* or disinhib* or inhib*)) (83)

1 TS=(attachment NEAR/2 (disorder$ or problem$ or style$ or pattern$)) (143)

Education Resources Information Center (ERIC; via ProQuest)

Date searched: 1966 to December 2011.

Date of search: 11 January 2012.

A total of 378 records were retrieved.

Search strategy

S1 (su((“Attachment Behavior”)) OR TI,AB(attachment NEAR/2 (disorder[*1] OR problem[*1] OR style[*1] OR pattern[*1]))) OR TI,AB(attachment NEAR/2 (behavio*r* or ambivalen* or avoidant or diffuse or organi* or disorgani* or disrupt* or abnormal* or disinhib* or inhib*)) OR TI,AB(attachment NEAR/2 (disorienta* or reactive or anxious* or disturb* or relation* or interven* or insecure* or secure or security or early or theory or theories))) 2909*

S2 SU(“Epidemiology” or “Incidence”) OR TI,AB(epidemiol* or incidence or prevalence or “patient history” or “family history” or risk[*1] or long term) 59,334*

S3 S1 and S2 378*

Social Services Abstracts (via CSA Illumina)

Date searched: 1979 to December 2011.

Date of search: 11 January 2012.

A total of 173 records were retrieved.

Search strategy

(KW=((attachment within 2 (disorder* or problem* or style* or pattern*)) or (attachment within 2 (behavior* or behavior* or ambivalen* or avoidant or diffuse or organi* or disorgani* or disrupt* or abnormal* or disinhib* or inhib*)) or (attachment within 2 (disorienta* or reactive or anxious* or disturb* or relation*))) or KW=(attachment within 2 (interven* or insecure* or secure or security or early or theory or theories))) and((DE=(“adolescents” or “children” or “infants”)) or(KW=((child* or infant* or infancy or preschool* or “pre school*” or baby or babies or pediat* or paediat*) or (boy or boys or girl or girls) or (schoolchild* or adolescen* or juvenile* or youth* or teenage* or youngster*)) or KW=(“young people” or “young person” or “young persons” or “young adult*” or “early adult*”)) or(DE=(Dyads or Child Neglect or Child Abuse or Foster Care or Foster Children or Adoption or Adopted Children) or KW=((parent* or mother* or maternal* or mum* or father* or paternal* or dad*) or (dyad* or attunement) or (representation* within 2 model*)) or KW=((neglect* or abuse or abused or abusive or maltreat* or mistreat*) or (foster* or adopt*)))) and(DE=(Epidemiology or (Risk Factors)) or KW=(epidemiol* or incidence or prevalence or history or risk or risks or “long term”))

© Queen’s Printer and Controller of HMSO 2015. This work was produced by Wright *et al.* under the terms of a commissioning contract issued by the Secretary of State for Health. This issue may be freely reproduced for the purposes of private research and study and extracts (or indeed, the full report) may be included in professional journals provided that suitable acknowledgement is made and the reproduction is not associated with any form of advertising. Applications for commercial reproduction should be addressed to: NIHR Journals Library, National Institute for Health Research, Evaluation, Trials and Studies Coordinating Centre, Alpha House, University of Southampton Science Park, Southampton SO16 7NS, UK.

Applied Social Sciences Index and Abstracts (ASSIA; via CSA Illumina)

Date searched: 1987 to December 2011.

Date of search: 11 January 2012.

A total of 284 records were retrieved.

Search strategy

((KW=((attachment within 2 (disorder* or problem* or style* or pattern*)) or (attachment within 2 (behavior* or behavior* or ambivalen* or avoidant or diffuse or organi* or disorgani* or disrupt* or abnormal* or disinhib* or inhib*)) or (attachment within 2 (disorienta* or reactive or anxious* or disturb* or relation*))) or KW=(attachment within 2 (interven* or insecure* or secure or security or early or theory or theories))) or(DE="attachment disorders")) and(DE=((Children or Infants or Adolescents) or (Parents or Dyads) or (Child neglect or Child abuse or Foster Care or Foster children or Adoption or Adopted children or Adoptive parents)) or KW=((child* or infant* or infancy or preschool* or "pre school*" or baby or babies or pediat* or paediat*) or (boy or boys or girl or girls) or (schoolchild* or adolescen* or juvenile* or youth* or teenage* or youngster* or "young people" or "young person" or "young persons" or "young adult*" or "early adult*")) or KW=((parent* or mother* or maternal* or mum* or father* or paternal* or dad* or dyad* or attunement) or (representation* within 2 model*) or (neglect* or abuse or abused or abusive or maltreat* or mistreat* or foster* or adopt*))) and(DE=(Epidemiology or (Risk Factors)) or KW=(epidemiol* or incidence or prevalence or history or risk or risks or "long term"))

Social Care Online (via SCIE)

Date searched: December 2011.

Date of search: 12 January 2012.

A total of 186 records were retrieved.

Advanced search option

(topic"attachment" or freetext="attachment") AND (topic="children" or topic="babies" or topic="young people" or topic="child abuse" or topic="child neglect" or topic="adoption" or topic="adoptive parents" or topic="adoptive children" or topic="foster care" or topic="foster children" or freetext="child*" or freetext="infant*" or freetext="infancy" or freetext="preschool*" or freetext="pre school*" or freetext="baby" or freetext="babies" or freetext="pediat*" or freetext="paediat*" or freetext="juvenile*" or freetext="youth*" or freetext="teenage*" or freetext="youngster*" or freetext="young people" or freetext="young person" or freetext="young persons" or freetext="young adult*" or freetext="early adult") AND (topic="risk" or freetext="epidemiol*" or freetext="incidence" or freetext="prevalence" or freetext="patient history" or freetext="family history" or freetext="risk*")

Named intervention programmes search strategies

PsycINFO (via OvidSP)

Date searched: 1806 to week 1, January 2012

Date of search: 6 January 2012.

A total of 1212 records were retrieved.

Search strategy

1. attachment behavior/ (13,469)
2. attachment disorders/ (370)
3. attachment theory/ (885)
4. (attachment adj2 (disorder$1 or problem$1 or style$1 or pattern$1)).ti,ab. (4327)
5. (attachment adj2 (behavio?r$ or ambivalen$ or avoidant or diffuse or organi$ or disorgani$ or disrupt$ or abnormal$ or disinhib$ or inhib$)).ti,ab. (2562)
6. (attachment adj2 (disorienta$ or reactive or anxious$ or disturb$ or relation$)).ti,ab. (3582)
7. (attachment adj2 (interven$ or insecure$ or secure or security or early or theory or theories)).ti,ab. (7456)
8. or/1-7 (17,172)
9. exp intervention/ (42,251)
10. play therapy/ (2443)
11. (theraplay or (play adj3 (therap$ or program or intervention$))).ti,ab. (3299)
12. (circle adj3 security).ti,ab. (12)
13. ((preschool$ or pre school$ or child$ or infant$) adj3 (psychotherap$ or psycho therap$)).ti,ab. (2909)
14. (watch adj2 wait adj2 wonder).ti,ab. (15)
15. ((interaction or interactive) adj3 guidance).ti,ab. (94)
16. (biobehavio$ or bio behavio$).ti,ab. (1142)
17. ((New Orleans adj3 (intervention$ or program$ or therap$)) or (tulane adj3 (team$ or program$ or intervention$ or therap$))).ti,ab. (20)
18. ((parent$ or mother$ or father$ or dyad$) adj3 (psychotherap$ or psycho therap$)).ti,ab. (889)
19. (((parent$ or child$) adj2 game$) or PCG).ti,ab. (564)
20. (floortime or (floor adj2 time)).ti,ab. (39)
21. ((manipulat$ adj3 respons$) or (Leiden adj3 (program$ or intervention$ or therap$))).ti,ab. (867)
22. (modif$ adj3 guidance).ti,ab. (36)
23. (video$ or VIPP or VIG).ti,ab. (33,944)
24. ((clinician$ adj3 exposure$) or CAVES).ti,ab. (115)
25. (Tamars adj3 Children$).ti,ab. (1)
26. (Florida adj3 (program$ or intervention$ or therap$)).ti,ab. (150)
27. exp Psychodynamic Psychotherapy/ (1711)
28. (psychodynamic adj3 psychotherap$).ti,ab. (1937)
29. ((story or stories) adj3 stem$).ti,ab. (136)
30. ((home or hospital or family) adj3 visit$).ti,ab. (3554)
31. Project CARE.ti,ab. (21)
32. Orion Project.ti,ab. (1)
33. ((violent adj3 resistan$) or (nonviolent adj3 resistan$) or NVR).ti,ab. (75)
34. (cues adj3 clues).ti,ab. (15)
35. (mellow adj3 (baby or babies or parent$)).ti,ab. (6)
36. solihull.ti,ab. (17)
37. ((self adj2 regulat$) or ARC).ti,ab. (12,505)
38. (personal adj3 contact$).ti,ab. (868)
39. ((baby or babies or infant$) adj2 (carrier$ or carry$)).ti,ab. (157)
40. (bath or bathe or bathing or massag$ or tickl$).ti,ab. (2849)

© Queen's Printer and Controller of HMSO 2015. This work was produced by Wright *et al.* under the terms of a commissioning contract issued by the Secretary of State for Health. This issue may be freely reproduced for the purposes of private research and study and extracts (or indeed, the full report) may be included in professional journals provided that suitable acknowledgement is made and the reproduction is not associated with any form of advertising. Applications for commercial reproduction should be addressed to: NIHR Journals Library, National Institute for Health Research, Evaluation, Trials and Studies Coordinating Centre, Alpha House, University of Southampton Science Park, Southampton SO16 7NS, UK.

41. (holding or restrain$ or rage reduc$ or rebirth$).ti,ab. (17,614)
42. ((feed$ or food or water) adj3 (therap$ or program or intervention$)).ti,ab. (1614)
43. or/9-42 (124,196)
44. 8 and 43 (1567)
45. (comment reply or editorial or letter or reprint or "review book" or "review media" or "review software other").dt. (221,270)
46. (animal or animals or rat or rats or mouse or mice or hamster or hamsters or dog or dogs or cat or cats or bovine or sheep or ovine or pig or pigs).ab,ti,id,de. (232,200)
47. 44 not (45 or 46) (1410)
48. (infancy 2 23 mo or neonatal birth 1 mo or preschool age 2 5 yrs).ag. (113,808)
49. (adolescence 13 17 yrs or childhood birth 12 yrs or school age 6 12 yrs).ag. (524,966)
50. (child$ or infant$ or infancy or preschool$ or pre school$ or baby or babies or pediat$ or paediat$).ti,ab. (504,891)
51. (boy or boys or girl or girls).ti,ab. (69,790)
52. (schoolchild$ or adolescen$ or juvenile$ or youth$ or teenage$ or youngster$).ti,ab. (190,092)
53. (young people or young person or young persons or young adult$ or early adult$).ti,ab. (38,372)
54. exp Parents/ (62,079)
55. exp Parenting/ (64,945)
56. (parent$ or mother$ or maternal$ or mum$ or father$ or paternal$ or dad$).ti,ab. (242,087)
57. Dyads/ (3998)
58. dyad$.ti,ab. (18,706)
59. (attunement or (representation$ adj2 model$)).ti,ab. (1587)
60. exp Child Neglect/ or exp Child Abuse/ (21,046)
61. exp Foster Children/ or exp Foster Care/ or exp Foster Parents/ (4034)
62. exp "Adoption (Child)"/ or exp Adoptive Parents/ (2891)
63. (neglect$ or abuse or abused or abusive or maltreat$ or mistreat$).ti,ab. (104,195)
64. (foster$ or adopt$).ti,ab. (69,814)
65. or/48-64 (955,446)
66. 47 and 65 (1212)

MEDLINE and MEDLINE In-Process & Other Non-Indexed Citations (via OvidSP)

Date searched: 1946 to week 4, December 2011.

Date of search: 9 January 2012.

A total of 211 records were retrieved in MEDLINE, and nine in MEDLINE In-Process.

Search strategy

1. Reactive Attachment Disorder/ (296)
2. (attachment adj2 (disorder$1 or problem$1 or style$1 or pattern$1)).ti,ab. (1100)
3. (attachment adj2 (behavio?r$ or ambivalen$ or avoidant or diffuse or organi$ or disorgani$ or disrupt$ or abnormal$ or disinhib$ or inhib$)).ti,ab. (2250)
4. (attachment adj2 (disorienta$ or reactive or anxious$ or disturb$ or relation$)).ti,ab. (842)
5. (attachment adj2 (interven$ or insecure$ or secure or security or early or theory or theories)).ti,ab. (1795)
6. or/1-5 (4718)
7. Intervention Studies/ (4992)
8. Play Therapy/ (866)
9. (theraplay or (play adj3 (therap$ or program or intervention$))).ti,ab. (1550)
10. (circle adj3 security).ti,ab. (6)
11. ((preschool$ or pre school$ or child$ or infant$) adj3 (psychotherap$ or psycho therap$)).ti,ab. (707)
12. (watch adj2 wait adj2 wonder).ti,ab. (1)
13. ((interaction or interactive) adj3 guidance).ti,ab. (103)
14. (biobehavio$ or bio behavio$).ti,ab. (907)

15. ((New Orleans adj3 (intervention$ or program$ or therap$)) or (tulane adj3 (team$ or program$ or intervention$ or therap$))).ti,ab. (40)
16. ((parent$ or mother$ or father$ or dyad$) adj3 (psychotherap$ or psycho therap$)).ti,ab. (210)
17. (((parent$ or child$) adj2 game$) or PCG).ti,ab. (1565)
18. (floortime or (floor adj2 time)).ti,ab. (27)
19. ((manipulat$ adj3 respons$) or (Leiden adj3 (program$ or intervention$ or therap$))).ti,ab. (1536)
20. Videotape Recording/ (9638)
21. (video$ or VIPP or VIG).ti,ab. (57,232)
22. (modif$ adj3 guidance).ti,ab. (60)
23. ((clinician$ adj3 exposure$) or CAVES).ti,ab. (555)
24. (Tamars adj3 Children$).ti,ab. (1)
25. (Florida adj3 (program$ or intervention$ or therap$)).ti,ab. (199)
26. (psychodynamic adj3 psychotherap$).ti,ab. (583)
27. ((story or stories) adj3 stem$).ti,ab. (58)
28. ((home or hospital or family) adj3 visit$).ti,ab. (10,384)
29. Project CARE.ti,ab. (14)
30. Orion Project.ti,ab. (4)
31. ((violent adj3 resistan$) or (nonviolent adj3 resistan$) or NVR).ti,ab. (68)
32. (cues adj3 clues).ti,ab. (11)
33. (mellow adj3 (baby or babies or parent$)).ti,ab. (2)
34. solihull.ti,ab. (41)
35. ((self adj2 regulat$) or ARC).ti,ab. (16,851)
36. (personal adj3 contact$).ti,ab. (1010)
37. ((baby or babies or infant$) adj2 (carrier$ or carry$)).ti,ab. (306)
38. (bath or bathe or bathing or massag$ or tickl$).ti,ab. (31,989)
39. (holding or restrain$ or rage reduc$ or rebirth$).ti,ab. (41,277)
40. ((feed$ or food or water) adj3 (therap$ or program or intervention$)).ti,ab. (4198)
41. or/7-40 (177,793)
42. 6 and 41 (257)
43. animals/ not (animals/ and humans/) (3,548,684)
44. (letter or editorial or comment or news or newspaper article).pt. (1,231,519)
45. 42 not (43 or 44) (243)
46. exp Child/ (1,400,869)
47. exp Infant/ (854,319)
48. Adolescent/ (1,434,825)
49. (child$ or infant$ or infancy or preschool$ or pre school$ or baby or babies or pediat$ or paediat$).ti,ab. (1,125,683)
50. (boy or boys or girl or girls).ti,ab. (136,911)
51. (schoolchild$ or adolescen$ or juvenile$ or youth$ or teenage$ or youngster$).ti,ab. (214,069)
52. (young people or young person or young persons or young adult$ or early adult$).ti,ab. (60,337)
53. exp Parents/ (60,696)
54. exp Parent-Child Relations/ or Parenting/ (45,480)
55. (parent$ or mother$ or maternal$ or mum$ or father$ or paternal$ or dad$).ti,ab. (520,407)
56. dyad$.ti,ab. (7450)
57. (attunement or (representation$ adj2 model$)).ti,ab. (692)
58. Child Abuse/ (15,437)
59. Foster Home Care/ (2730)
60. Adoption/ (3984)
61. (neglect$ or abuse or abused or abusive or maltreat$ or mistreat$).ti,ab. (101,116)
62. (foster$ or adopt$).ti,ab. (116,855)
63. or/46-62 (3,282,233)
64. 45 and 63 (211)

© Queen's Printer and Controller of HMSO 2015. This work was produced by Wright *et al.* under the terms of a commissioning contract issued by the Secretary of State for Health. This issue may be freely reproduced for the purposes of private research and study and extracts (or indeed, the full report) may be included in professional journals provided that suitable acknowledgement is made and the reproduction is not associated with any form of advertising. Applications for commercial reproduction should be addressed to: NIHR Journals Library, National Institute for Health Research, Evaluation, Trials and Studies Coordinating Centre, Alpha House, University of Southampton Science Park, Southampton SO16 7NS, UK.

EMBASE (via OvidSP)

Date searched: 1974 to week 1, 2012.

Date of search: 10 January 2012.

A total of 291 records were retrieved.

Search strategy

1. (attachment adj2 (disorder$1 or problem$1 or style$1 or pattern$1)).ti,ab. (1565)
2. (attachment adj2 (behavio?r$ or ambivalen$ or avoidant or diffuse or organi$ or disorgani$ or disrupt$ or abnormal$ or disinhib$ or inhib$)).ti,ab. (2670)
3. (attachment adj2 (disorienta$ or reactive or anxious$ or disturb$ or relation$)).ti,ab. (1148)
4. (attachment adj2 (interven$ or insecure$ or secure or security or early or theory or theories)).ti,ab. (2513)
5. or/1-4 (5956)
6. intervention study/ (12,529)
7. play therapy/ (1258)
8. (theraplay or (play adj3 (therap$ or program or intervention$))).ti,ab. (2276)
9. (circle adj3 security).ti,ab. (10)
10. ((preschool$ or pre school$ or child$ or infant$) adj3 (psychotherap$ or psycho therap$)).ti,ab. (1294)
11. (watch adj2 wait adj2 wonder).ti,ab. (5)
12. ((interaction or interactive) adj3 guidance).ti,ab. (141)
13. (biobehavio$ or bio behavio$).ti,ab. (1121)
14. ((New Orleans adj3 (intervention$ or program$ or therap$)) or (tulane adj3 (team$ or program$ or intervention$ or therap$))).ti,ab. (44)
15. ((parent$ or mother$ or father$ or dyad$) adj3 (psychotherap$ or psycho therap$)).ti,ab. (369)
16. (((parent$ or child$) adj2 game$) or PCG).ti,ab. (2017)
17. (floortime or (floor adj2 time)).ti,ab. (36)
18. ((manipulat$ adj3 respons$) or (Leiden adj3 (program$ or intervention$ or therap$))).ti,ab. (1844)
19. videorecording/ (29,800)
20. (video$ or VIPP or VIG).ti,ab. (73,821)
21. (modif$ adj3 guidance).ti,ab. (86)
22. ((clinician$ adj3 exposure$) or CAVES).ti,ab. (723)
23. (Tamars adj3 Children$).ti,ab. (1)
24. (Florida adj3 (program$ or intervention$ or therap$)).ti,ab. (238)
25. (psychodynamic adj3 psychotherap$).ti,ab. (941)
26. ((story or stories) adj3 stem$).ti,ab. (67)
27. ((home or hospital or family) adj3 visit$).ti,ab. (13,559)
28. Project CARE.ti,ab. (21)
29. Orion Project.ti,ab. (4)
30. ((violent adj3 resistan$) or (nonviolent adj3 resistan$) or NVR).ti,ab. (111)
31. (cues adj3 clues).ti,ab. (13)
32. (mellow adj3 (baby or babies or parent$)).ti,ab. (4)
33. solihull.ti,ab. (58)
34. ((self adj2 regulat$) or ARC).ti,ab. (21,865)
35. (personal adj3 contact$).ti,ab. (1299)
36. ((baby or babies or infant$) adj2 (carrier$ or carry$)).ti,ab. (376)
37. (bath or bathe or bathing or massag$ or tickl$).ti,ab. (41,112)
38. (holding or restrain$ or rage reduc$ or rebirth$).ti,ab. (50,303)
39. ((feed$ or food or water) adj3 (therap$ or program or intervention$)).ti,ab. (5536)

40. or/6-39 (235,474)
41. 5 and 40 (354)
42. Animal/ or Animal Experiment/ or Nonhuman/ (5,761,726)
43. (rat or rats or mouse or mice or murine or rodent or rodents or hamster or hamsters or pig or pigs or porcine or rabbit or rabbits or animal or animals or dogs or dog or cats or cow or bovine or sheep or ovine or monkey or monkeys).ti,ab,sh. (4,749,774)
44. 42 or 43 (6,446,779)
45. exp Human/ or Human Experiment/ (12,937,340)
46. 44 not (44 and 45) (5,116,251)
47. (editorial or letter or note).pt. (1,613,483)
48. 41 not (46 or 47) (335)
49. child/ (1,135,530)
50. infant/ (476,014)
51. adolescent/ (1,127,803)
52. (child$ or infant$ or infancy or preschool$ or pre school$ or baby or babies or pediat$ or paediat$).ti,ab. (1,413,823)
53. (boy or boys or girl or girls).ti,ab. (177,580)
54. (schoolchild$ or adolescen$ or juvenile$ or youth$ or teenage$ or youngster$).ti,ab. (272,836)
55. (young people or young person or young persons or young adult$ or early adult$).ti,ab. (76,703)
56. exp parent/ (114,307)
57. exp child parent relation/ (58,704)
58. (parent$ or mother$ or maternal$ or mum$ or father$ or paternal$ or dad$).ti,ab. (911,848)
59. dyad$.ti,ab. (9350)
60. (attunement or (representation$ adj2 model$)).ti,ab. (894)
61. child abuse/ or child neglect/ (21,051)
62. foster care/ (3077)
63. adoption/ or adopted child/ (4815)
64. (neglect$ or abuse or abused or abusive or maltreat$ or mistreat$).ti,ab. (131,980)
65. (foster$ or adopt$).ti,ab. (150,939)
66. or/49-65 (3,567,803)
67. 48 and 66 (291)

Social Policy & Practice (via OvidSP)

Date searched: inception to 2012.

Date of search: 10 January 2012.

A total of 162 records were retrieved.

Search strategy

1. attachment disorder.de. (232)
2. (attachment adj2 (disorder$1 or problem$1 or style$1 or pattern$1)).ti,ab. (390)
3. (attachment adj2 (behavio?r$ or ambivalen$ or avoidant or diffuse or organi$ or disorgani$ or disrupt$ or abnormal$ or disinhib$ or inhib$)).ti,ab. (196)
4. (attachment adj2 (disorienta$ or reactive or anxious$ or disturb$ or relation$)).ti,ab. (265)
5. (attachment adj2 (interven$ or insecure$ or secure or security or early or theory or theories)).ti,ab. (824)
6. or/1-5 (1309)
7. intervention programmes.de. (6409)
8. play therapy.de. (528)

© Queen's Printer and Controller of HMSO 2015. This work was produced by Wright *et al.* under the terms of a commissioning contract issued by the Secretary of State for Health. This issue may be freely reproduced for the purposes of private research and study and extracts (or indeed, the full report) may be included in professional journals provided that suitable acknowledgement is made and the reproduction is not associated with any form of advertising. Applications for commercial reproduction should be addressed to: NIHR Journals Library, National Institute for Health Research, Evaluation, Trials and Studies Coordinating Centre, Alpha House, University of Southampton Science Park, Southampton SO16 7NS, UK.

9. (theraplay or (play adj3 (therap$ or program or intervention$))).ti,ab. (378)
10. (circle adj3 security).ti,ab. (4)
11. ((preschool$ or pre school$ or child$ or infant$) adj3 (psychotherap$ or psycho therap$)).ti,ab. (260)
12. (watch adj2 wait adj2 wonder).ti,ab. (1)
13. ((interaction or interactive) adj3 guidance).ti,ab. (12)
14. (biobehavio$ or bio behavio$).ti,ab. (17)
15. ((New Orleans adj3 (intervention$ or program$ or therap$)) or (tulane adj3 (team$ or program$ or intervention$ or therap$))).ti,ab. (2)
16. ((parent$ or mother$ or father$ or dyad$) adj3 (psychotherap$ or psycho therap$)).ti,ab. (72)
17. (((parent$ or child$) adj2 game$) or PCG).ti,ab. (164)
18. (floortime or (floor adj2 time)).ti,ab. (5)
19. ((manipulat$ adj3 respons$) or (Leiden adj3 (program$ or intervention$ or therap$))).ti,ab. (4)
20. (modif$ adj3 guidance).ti,ab. (0)
21. (video$ or VIPP or VIG).ti,ab. (1859)
22. ((clinician$ adj3 exposure$) or CAVES).ti,ab. (5)
23. (Tamars adj3 Children$).ti,ab. (1)
24. (Florida adj3 (program$ or intervention$ or therap$)).ti,ab. (18)
25. (psychodynamic adj3 psychotherap$).ti,ab. (52)
26. ((story or stories) adj3 stem$).ti,ab. (18)
27. ((home or hospital or family) adj3 visit$).ti,ab. (918)
28. Project CARE.ti,ab. (19)
29. Orion Project.ti,ab. (0)
30. ((violent adj3 resistan$) or (nonviolent adj3 resistan$) or NVR).ti,ab. (20)
31. (cues adj3 clues).ti,ab. (1)
32. (mellow adj3 (baby or babies or parent$)).ti,ab. (5)
33. solihull.ti,ab. (146)
34. ((self adj2 regulat$) or ARC).ti,ab. (347)
35. (personal adj3 contact$).ti,ab. (72)
36. ((baby or babies or infant$) adj2 (carrier$ or carry$)).ti,ab. (6)
37. (bath or bathe or bathing or massag$ or tickl$).ti,ab. (503)
38. (holding or restrain$ or rage reduc$ or rebirth$).ti,ab. (1591)
39. ((feed$ or food or water) adj3 (therap$ or program or intervention$)).ti,ab. (82)
40. or/7-39 (12,821)
41. 6 and 40 (168)
42. (child$ or infant$ or infancy or preschool$ or pre school$ or baby or babies or pediat$ or paediat$).ti,ab,de. (125,901)
43. (boy or boys or girl or girls).ti,ab,de. (5692)
44. (schoolchild$ or adolescen$ or juvenile$ or youth$ or teenage$ or youngster$).ti,ab,de. (32,056)
45. (young people or young person or young persons or young adult$ or early adult$).ti,ab,de. (42,212)
46. (parent$ or mother$ or maternal$ or mum$ or father$ or paternal$ or dad$).ti,ab,de. (47,034)
47. dyad$.ti,ab,de. (480)
48. (attunement or (representation$ adj2 model$)).ti,ab,de. (66)
49. (neglect$ or abuse or abused or abusive or maltreat$ or mistreat$).ti,ab,de. (23,203)
50. (foster$ or adopt$).ti,ab,de. (19,994)
51. or/42-50 (172,573)
52. 41 and 51 (162)

Science Citation Index (SCI; via ISI Web of Science)

Date searched: 1899 to 6 January 2012.

Date of search: 10 January 2012.

A total of 88 records were retrieved.

Databases=SCI-EXPANDED Timespan=All Years.

Lemmatization=Off.

Search strategy

52 #50 not #51 (88)

51 TS=(rat or rats or mouse or mice or murine or hamster or hamsters or animal or animals or dogs or dog or pig or pigs or cats or bovine or cow or sheep or ovine or porcine or monkey) (3,408,525)

50 #39 and #49 (96)

49 #48 OR #47 OR #46 OR #45 OR #44 OR #43 OR #42 OR #41 OR #40 (1,972,974)

48 TS=(foster* or adopt*) (207,538)

47 TS=(neglect* or abuse or abused or abusive or maltreat* or mistreat*) (117,173)

46 TS=(attunement or (representation* NEAR/2 model*)) (6475)

45 TS=dyad* (10,857)

44 TS=(parent* or mother* or maternal* or mum* or father* or paternal* or dad*) (574,227)

43 TS=("young people" or "young person" or "young persons" or "young adult*" or "early adult*") (55,963)

42 TS=(schoolchild* or adolescen* or juvenile* or youth* or teenage* or youngster*) (249,910)

41 TS=(boy or boys or girl or girls) (96,380)

40 TS=(child* or infant* or infancy or preschool* or "pre school*" or baby or babies or pediat* or paediat*) (1,021,056)

39 #5 and #38 (128)

38 #37 OR #36 OR #35 OR #34 OR #33 OR #32 OR #31 OR #30 OR #29 OR #28 OR #27 OR #26 OR #25 OR #24 OR #23 OR #22 OR #21 OR #20 OR #19 OR #18 OR #17 OR #16 OR #15 OR #14 OR #13 OR #12 OR #11 OR #10 OR #9 OR #8 OR #7 OR #6 (239,830)

37 TS=((feed* or food or water) NEAR/3 (therap* or program or intervention*)) (7948)

36 TS=(holding or restrain* or "rage reduc*" or rebirth*) (62,561)

© Queen's Printer and Controller of HMSO 2015. This work was produced by Wright *et al.* under the terms of a commissioning contract issued by the Secretary of State for Health. This issue may be freely reproduced for the purposes of private research and study and extracts (or indeed, the full report) may be included in professional journals provided that suitable acknowledgement is made and the reproduction is not associated with any form of advertising. Applications for commercial reproduction should be addressed to: NIHR Journals Library, National Institute for Health Research, Evaluation, Trials and Studies Coordinating Centre, Alpha House, University of Southampton Science Park, Southampton SO16 7NS, UK.

35 TS=(bath or bathe or bathing or massag* or tickl*) (49,406)

34 TS=((baby or babies or infant*) NEAR/2 (carrier* or carry*)) (424)

33 TS=(personal NEAR/3 contact*) (659)

32 TS=(self NEAR/2 regulat*) (6655)

31 TS=solihull (33)

30 TS=(mellow NEAR/3 (baby or babies or parent*)) (1)

29 TS=(cues NEAR/3 clues) (14)

28 TS=(violent NEAR/3 resistan*) or TS=(nonviolent NEAR/3 resistan*) or TS=(NVR) (76)

27 TS="Orion Project" (5)

26 TS="Project CARE" (7)

25 TS=((home or hospital or family) NEAR/3 visit*) (6921)

24 TS=((story or stories) NEAR/3 stem*) (26)

23 TS=(psychodynamic NEAR/3 psychotherap*) (422)

22 TS=(Florida NEAR/3 (program* or intervention* or therap*)) (321)

21 TS=Tamars (0)

20 TS=(clinician* NEAR/3 exposure*) or TS=(CAVES) (3528)

19 TS=(video* or VIPP or VIG) (92,063)

18 TS=(modif* NEAR/3 guidance) (123)

17 TS=(manipulate* NEAR/3 respons*) or TS=(Leiden NEAR/3 (program* or intervention* or therap*)) (635)

16 TS=(floortime) or TS=(floor NEAR/2 time) (194)

15 TS=((parent* or child*) NEAR/2 game*) (441)

14 TS=((parent* or mother* or father* or dyad*) NEAR/3 (psychotherapy* or "psycho therap*")) (115)

13 TS=("New Orleans" NEAR/3 (intervention* or program* or therap*)) or TS=(tulane NEAR/3 (team* or program* or intervention* or therap*)) (35)

12 TS=(biobehavio* or "bio behavio*") (758)

11 TS=((interaction or interactive) NEAR/3 guidance) (160)

10 TS=(watch NEAR/2 wait) (269)

9 TS=((preschool* or "pre school*" or child* or infant*) NEAR/3 (psychotherap* or "psycho therap*")) (556)

8 TS=(circle NEAR/3 security) (5)

7 TS=(theraplay) or TS=(play NEAR/3 (therap* or program* or intervention*)) (1522)

6 TS=(intervention NEAR/2 program*) (6490)

5 #1 OR #2 OR #3 OR #4 (4095)

4 TS=(attachment NEAR/2 (intervene* or insecure* or secure or security or early or theory or theories)) (1054)

3 TS=(attachment NEAR/2 (disorienta* or reactive or anxious* or disturb* or relation*)) (593)

2 TS=(attachment NEAR/2 (behavior$r* or ambivalen* or avoidant or diffuse or organi* or disorgani* or disrupt* or abnormal* or disinhib* or inhib*)) (2203)

1 TS=(attachment NEAR/2 (disorder$ or problem$ or style$ or pattern$)) (924)

Social Science Citation Index (SSCI; via ISI Web of Science)

Date searched: 1956 to 6 January 2012.

Date of search: 10 January 2012.

A total of 426 records were retrieved.

Databases=SSCI Timespan=All Years.

Lemmatization=Off.

Search strategy

52 #50 not #51 (426)

51 TS=(rat or rats or mouse or mice or murine or hamster or hamsters or animal or animals or dogs or dog or pig or pigs or cats or bovine or cow or sheep or ovine or porcine or monkey) (91,781)

50 #39 and #49 (431)

49 #48 OR #47 OR #46 OR #45 OR #44 OR #43 OR #42 OR #41 OR #40 (682,942)

48 TS=(foster* or adopt*) (75,821)

47 TS=(neglect* or abuse or abused or abusive or maltreat* or mistreat*) (91,084)

46 TS=(attunement or (representation* NEAR/2 model*)) (2062)

45 TS=dyad* (10,250)

© Queen's Printer and Controller of HMSO 2015. This work was produced by Wright *et al.* under the terms of a commissioning contract issued by the Secretary of State for Health. This issue may be freely reproduced for the purposes of private research and study and extracts (or indeed, the full report) may be included in professional journals provided that suitable acknowledgement is made and the reproduction is not associated with any form of advertising. Applications for commercial reproduction should be addressed to: NIHR Journals Library, National Institute for Health Research, Evaluation, Trials and Studies Coordinating Centre, Alpha House, University of Southampton Science Park, Southampton SO16 7NS, UK.

44 TS=(parent* or mother* or maternal* or mum* or father* or paternal* or dad*) (160,559)

43 TS=("young people" or "young person" or "young persons" or "young adult*" or "early adult*") (35,541)

42 TS=(schoolchild* or adolescen* or juvenile* or youth* or teenage* or youngster*) (165,662)

41 TS=(boy or boys or girl or girls) (42,755)

40 TS=(child* or infant* or infancy or preschool* or "pre school*" or baby or babies or pediat* or paediat*) (392,420)

39 #5 and #38 (507)

38 #37 OR #36 OR #35 OR #34 OR #33 OR #32 OR #31 OR #30 OR #29 OR #28 OR #27 OR #26 OR #25 OR #24 OR #23 OR #22 OR #21 OR #20 OR #19 OR #18 OR #17 OR #16 OR #15 OR #14 OR #13 OR #12 OR #11 OR #10 OR #9 OR #8 OR #7 OR #6 (69,998)

37 TS=((feed* or food or water) NEAR/3 (therap* or program or intervention*)) (2609)

36 TS=(holding or restrain* or "rage reduc*" or rebirth*) (17,063)

35 TS=(bath or bathe or bathing or massag* or tickl*) (2861)

34 TS=((baby or babies or infant*) NEAR/2 (carrier* or carry*)) (89)

33 TS=(personal NEAR/3 contact*) (605)

32 TS=(self NEAR/2 regulat*) (8392)

31 TS=solihull (16)

30 TS=(mellow NEAR/3 (baby or babies or parent*)) (2)

29 TS=(cues NEAR/3 clues) (11)

28 TS=(violent NEAR/3 resistan*) or TS=(nonviolent NEAR/3 resistan*) or TS=(NVR) (88)

27 TS="Orion Project" (3)

26 TS="Project CARE" (14)

25 TS=((home or hospital or family) NEAR/3 visit*) (4069)

24 TS=((story or stories) NEAR/3 stem*) (71)

23 TS=(psychodynamic NEAR/3 psychotherap*) (1031)

22 TS=(Florida NEAR/3 (program* or intervention* or therap*)) (208)

21 TS=Tamars (0)

20 TS=(clinician* NEAR/3 exposure*) or TS=(CAVES) (580)

19 TS=(video* or VIPP or VIG) (21,932)

18 TS=(modif* NEAR/3 guidance) (15)

17 TS=(manipulate* NEAR/3 respons*) or TS=(Leiden NEAR/3 (program* or intervention* or therap*)) (210)

16 TS=(floortime) or TS=(floor NEAR/2 time) (30)

15 TS=((parent* or child*) NEAR/2 game*) (495)

14 TS=((parent* or mother* or father* or dyad*) NEAR/3 (psychotherapy* or "psycho therap*")) (404)

13 TS=("New Orleans" NEAR/3 (intervention* or program* or therap*)) or TS=(tulane NEAR/3 (team* or program* or intervention* or therap*)) (21)

12 TS=(biobehavio* or "bio behavio*") (771)

11 TS=((interaction or interactive) NEAR/3 guidance) (48)

10 TS=(watch NEAR/2 wait) (12)

9 TS=((preschool* or "pre school*" or child* or infant*) NEAR/3 (psychotherap* or "psycho therap*")) (1902)

8 TS=(circle NEAR/3 security) (4)

7 TS=(theraplay) or TS=(play NEAR/3 (therap* or program* or intervention*)) (1113)

6 TS=(intervention NEAR/2 program*) (7397)

5 #1 OR #2 OR #3 OR #4 (6315)

4 TS=(attachment NEAR/2 (intervene* or insecure* or secure or security or early or theory or theories)) (3614)

3 TS=(attachment NEAR/2 (disorienta* or reactive or anxious* or disturb* or relation*)) (2147)

2 TS=(attachment NEAR/2 (behavior$r* or ambivalen* or avoidant or diffuse or organi* or disorgani* or disrupt* or abnormal* or disinhib* or inhib*)) (1017)

1 TS=(attachment NEAR/2 (disorder$ or problem$ or style$ or pattern$)) (2646)

© Queen's Printer and Controller of HMSO 2015. This work was produced by Wright *et al.* under the terms of a commissioning contract issued by the Secretary of State for Health. This issue may be freely reproduced for the purposes of private research and study and extracts (or indeed, the full report) may be included in professional journals provided that suitable acknowledgement is made and the reproduction is not associated with any form of advertising. Applications for commercial reproduction should be addressed to: NIHR Journals Library, National Institute for Health Research, Evaluation, Trials and Studies Coordinating Centre, Alpha House, University of Southampton Science Park, Southampton SO16 7NS, UK.

Conference Proceedings Citation Index – Science (CPCI-S; via ISI Web of Science)

Date searched: 1990 to 6 January 2012.

Date of search: 10 January 2012.

A total of three records were retrieved.

Databases=CPCI-S Timespan=All Years.

Lemmatization=Off.

Search strategy

52 #50 not #51 (3)

51 TS=(rat or rats or mouse or mice or murine or hamster or hamsters or animal or animals or dogs or dog or pig or pigs or cats or bovine or cow or sheep or ovine or porcine or monkey) (282,876)

50 #39 and #49 (4)

49 #48 OR #47 OR #46 OR #45 OR #44 OR #43 OR #42 OR #41 OR #40 (291,238)

48 TS=(foster* or adopt*) (87,891)

47 TS=(neglect* or abuse or abused or abusive or maltreat* or mistreat*) (17,504)

46 TS=(attunement or (representation* NEAR/2 model*)) (3792)

45 TS=dyad* (1817)

44 TS=(parent* or mother* or maternal* or mum* or father* or paternal* or dad*) (70,471)

43 TS=("young people" or "young person" or "young persons" or "young adult*" or "early adult*") (5160)

42 TS=(schoolchild* or adolescen* or juvenile* or youth* or teenage* or youngster*) (24,476)

41 TS=(boy or boys or girl or girls) (5857)

40 TS=(child* or infant* or infancy or preschool* or "pre school*" or baby or babies or pediat* or paediat*) (104,538)

39 #5 and #38 (9)

38 #37 OR #36 OR #35 OR #34 OR #33 OR #32 OR #31 OR #30 OR #29 OR #28 OR #27 OR #26 OR #25 OR #24 OR #23 OR #22 OR #21 OR #20 OR #19 OR #18 OR #17 OR #16 OR #15 OR #14 OR #13 OR #12 OR #11 OR #10 OR #9 OR #8 OR #7 OR #6 (91,491)

37 TS=((feed* or food or water) NEAR/3 (therap* or program or intervention*)) (1518)

36 TS=(holding or restrain* or "rage reduc*" or rebirth*) (13,037)

35 TS=(bath or bathe or bathing or massag* or tickl*) (7541)

34 TS=((baby or babies or infant*) NEAR/2 (carrier* or carry*)) (33)

33 TS=(personal NEAR/3 contact*) (91)

32 TS=(self NEAR/2 regulat*) (1493)

31 TS=solihull (6)

30 TS=(mellow NEAR/3 (baby or babies or parent*)) (0)

29 TS=(cues NEAR/3 clues) (3)

28 TS=(violent NEAR/3 resistan*) or TS=(nonviolent NEAR/3 resistan*) or TS=(NVR) (49)

27 TS="Orion Project" (4)

26 TS="Project CARE" (8)

25 TS=((home or hospital or family) NEAR/3 visit*) (575)

24 TS=((story or stories) NEAR/3 stem*) (1)

23 TS=(psychodynamic NEAR/3 psychotherap*) (24)

22 TS=(Florida NEAR/3 (program* or intervention* or therap*)) (83)

21 TS=Tamars (0)

20 TS=(clinician* NEAR/3 exposure*) or TS=(CAVES) (640)

19 TS=(video* or VIPP or VIG) (65,316)

18 TS=(modif* NEAR/3 guidance) (52)

17 TS=(manipulate* NEAR/3 respons*) or TS=(Leiden NEAR/3 (program* or intervention* or therap*)) (67)

16 TS=(floortime) or TS=(floor NEAR/2 time) (90)

15 TS=((parent* or child*) NEAR/2 game*) (146)

14 TS=((parent* or mother* or father* or dyad*) NEAR/3 (psychotherapy* or "psycho therap*")) (4)

13 TS=("New Orleans" NEAR/3 (intervention* or program* or therap*)) or TS=(tulane NEAR/3 (team* or program* or intervention* or therap*)) (4)

12 TS=(biobehavio* or "bio behavio*") (106)

11 TS=((interaction or interactive) NEAR/3 guidance) (81)

© Queen's Printer and Controller of HMSO 2015. This work was produced by Wright *et al.* under the terms of a commissioning contract issued by the Secretary of State for Health. This issue may be freely reproduced for the purposes of private research and study and extracts (or indeed, the full report) may be included in professional journals provided that suitable acknowledgement is made and the reproduction is not associated with any form of advertising. Applications for commercial reproduction should be addressed to: NIHR Journals Library, National Institute for Health Research, Evaluation, Trials and Studies Coordinating Centre, Alpha House, University of Southampton Science Park, Southampton SO16 7NS, UK.

10 TS=(watch NEAR/2 wait) (51)

9 TS=((preschool* or "pre school*" or child* or infant*) NEAR/3 (psychotherap* or "psycho therap*")) (27)

8 TS=(circle NEAR/3 security) (2)

7 TS=(theraplay) or TS=(play NEAR/3 (therap* or program* or intervention*)) (234)

6 TS=(intervention NEAR/2 program*) (628)

5 #1 OR #2 OR #3 OR #4 (343)

4 TS=(attachment NEAR/2 (intervene* or insecure* or secure or security or early or theory or theories)) (93)

3 TS=(attachment NEAR/2 (disorienta* or reactive or anxious* or disturb* or relation*)) (54)

2 TS=(attachment NEAR/2 (behavior$r* or ambivalen* or avoidant or diffuse or organi* or disorgani* or disrupt* or abnormal* or disinhib* or inhib*)) (141)

1 TS=(attachment NEAR/2 (disorder$ or problem$ or style$ or pattern$)) (95)

Databases=CPCI-S Timespan=All Years

Conference Proceedings Citation Index – Social Science & Humanities (CPCI-SSH; via ISI Web of Science)

Date searched: 1990 to 6 January 2012.

Date of search: 10 January 2012.

A total of 31 records were retrieved.

Databases=CPCI-SSH Timespan=All Years.

Lemmatization=Off.

Search strategy

52 #50 not #51 (31)

51 TS=(rat or rats or mouse or mice or murine or hamster or hamsters or animal or animals or dogs or dog or pig or pigs or cats or bovine or cow or sheep or ovine or porcine or monkey) (4408)

50 #39 and #49 (31)

49 #48 OR #47 OR #46 OR #45 OR #44 OR #43 OR #42 OR #41 OR #40 (45,707)

48 TS=(foster* or adopt*) (13,580)

47 TS=(neglect* or abuse or abused or abusive or maltreat* or mistreat*) (5468)

46 TS=(attunement or (representation* NEAR/2 model*)) (356)

45 TS=dyad* (832)

44 TS=(parent* or mother* or maternal* or mum* or father* or paternal* or dad*) (8462)

43 TS=("young people" or "young person" or "young persons" or "young adult*" or "early adult*") (1874)

42 TS=(schoolchild* or adolescen* or juvenile* or youth* or teenage* or youngster*) (7388)

41 TS=(boy or boys or girl or girls) (2090)

40 TS=(child* or infant* or infancy or preschool* or "pre school*" or baby or babies or pediat* or paediat*) (19,667)

39 #5 and #38 (38)

38 #37 OR #36 OR #35 OR #34 OR #33 OR #32 OR #31 OR #30 OR #29 OR #28 OR #27 OR #26 OR #25 OR #24 OR #23 OR #22 OR #21 OR #20 OR #19 OR #18 OR #17 OR #16 OR #15 OR #14 OR #13 OR #12 OR #11 OR #10 OR #9 OR #8 OR #7 OR #6 (7,242)

37 TS=((feed* or food or water) NEAR/3 (therap* or program or intervention*)) (172)

36 TS=(holding or restrain* or "rage reduc*" or rebirth*) (1844)

35 TS=(bath or bathe or bathing or massag* or tickl*) (185)

34 TS=((baby or babies or infant*) NEAR/2 (carrier* or carry*)) (2)

33 TS=(personal NEAR/3 contact*) (63)

32 TS=(self NEAR/2 regulat*) (645)

31 TS=solihull (0)

30 TS=(mellow NEAR/3 (baby or babies or parent*)) (0)

29 TS=(cues NEAR/3 clues) (2)

28 TS=(violent NEAR/3 resistan*) or TS=(nonviolent NEAR/3 resistan*) or TS=(NVR) (8)

27 TS="Orion Project" (0)

26 TS="Project CARE" (1)

25 TS=((home or hospital or family) NEAR/3 visit*) (175)

24 TS=((story or stories) NEAR/3 stem*) (4)

23 TS=(psychodynamic NEAR/3 psychotherap*) (55)

© Queen's Printer and Controller of HMSO 2015. This work was produced by Wright *et al.* under the terms of a commissioning contract issued by the Secretary of State for Health. This issue may be freely reproduced for the purposes of private research and study and extracts (or indeed, the full report) may be included in professional journals provided that suitable acknowledgement is made and the reproduction is not associated with any form of advertising. Applications for commercial reproduction should be addressed to: NIHR Journals Library, National Institute for Health Research, Evaluation, Trials and Studies Coordinating Centre, Alpha House, University of Southampton Science Park, Southampton SO16 7NS, UK.

22 TS=(Florida NEAR/3 (program* or intervention* or therap*)) (17)

21 TS=Tamars (0)

20 TS=(clinician* NEAR/3 exposure*) or TS=(CAVES) (72)

19 TS=(video* or VIPP or VIG) (3479)

18 TS=(modif* NEAR/3 guidance) (2)

17 TS=(manipulate* NEAR/3 respons*) or TS=(Leiden NEAR/3 (program* or intervention* or therap*)) (13)

16 TS=(floortime) or TS=(floor NEAR/2 time) (3)

15 TS=((parent* or child*) NEAR/2 game*) (78)

14 TS=((parent* or mother* or father* or dyad*) NEAR/3 (psychotherapy* or "psycho therap*")) (25)

13 TS=("New Orleans" NEAR/3 (intervention* or program* or therap*)) or TS=(tulane NEAR/3 (team* or program* or intervention* or therap*)) (4)

12 TS=(biobehavio* or "bio behavio*") (35)

11 TS=((interaction or interactive) NEAR/3 guidance) (10)

10 TS=(watch NEAR/2 wait) (2)

9 TS=((preschool* or "pre school*" or child* or infant*) NEAR/3 (psychotherap* or "psycho therap*")) (81)

8 TS=(circle NEAR/3 security) (1)

7 TS=(theraplay) or TS=(play NEAR/3 (therap* or program* or intervention*)) (56)

6 TS=(intervention NEAR/2 program*) (327)

5 #1 OR #2 OR #3 OR #4 (425)

4 TS=(attachment NEAR/2 (interven* or insecure* or secure or security or early or theory or theories)) (254)

3 TS=(attachment NEAR/2 (disorienta* or reactive or anxious* or disturb* or relation*)) (170)

2 TS=(attachment NEAR/2 (behavior$r* or ambivalen* or avoidant or diffuse or organi* or disorgani* or disrupt* or abnormal* or disinhib* or inhib*)) (83)

1 TS=(attachment NEAR/2 (disorder$ or problem$ or style$ or pattern$)) (143)

Education Resources Information Center (ERIC; via ProQuest)

Date searched: 1966 to December 2011.

Date of search: 11 January 2012.

A total of 372 records were retrieved.

Search strategy

S10 S1 and S9 (372*)

S9 S2 or S3 or S4 or S5 or S6 or S7 or S8 (69,842*)

S8 TI,AB((baby or babies or infant*) NEAR/2 (carrier* or carry*)) OR TI,AB(bath or bathe or bathing or massag*$ or tickl*) OR TI,AB(holding or restrain* or rage reduc* or rebirth*) OR TI,AB((feed* or food or water) NEAR/3 (therap* or program or intervention*) (8820*)

S7 TI,AB(cues NEAR/3 clues) OR TI,AB(mellow NEAR/3 (baby or babies or parent*)) OR TI,AB(solihull) OR TI,AB(self NEAR/2 regulat*) OR TI,AB(personal NEAR/3 contact*) (3423*)

S6 TI,AB((story or stories) NEAR/3 stem*) OR TI,AB((home or hospital or family) NEAR/3 visit*) OR TI,AB ("Project CARE") OR TI,AB("Orion Project") AND TI,AB((violent NEAR/3 resistan*) or (nonviolent NEAR/3 resistan*) or NVR) (2027*)

S5 TI,AB(video* or VIPP or VIG) OR TI,AB((clinician* NEAR/3 exposure*) or CAVES) OR TI,AB(Tamars NEAR/3 Children*) OR TI,AB(Florida NEAR/3 (program* or intervention* or therap*)) AND TI,AB (psychodynamic NEAR/3 psychotherap*) (23,895*)

S4 TI,AB((parent* or mother* or father* or dyad*) NEAR/3 (psychotherap* or "psycho therap*")) OR TI, AB((parent* or child*) NEAR/2 game*) OR TI,AB(floortime or (floor NEAR/2 time)) OR TI,AB((manipulat* NEAR/3 respons*) or (Leiden NEAR/3 (program* or intervention* or therap*))) AND TI,AB(modif* NEAR/3 guidance) (760*)

S3 TI,AB((preschool* or "pre school*" or child* or infant*) NEAR/3 (psychotherap* or "psycho therap*")) OR TI,AB(watch NEAR/2 wait) OR TI,AB((interaction or interactive) NEAR/3 guidance) OR TI,AB (biobehavio* or "bio behavio*") AND TI,AB((New Orleans NEAR/3 (intervention* or program* or therap*)) or (tulane NEAR/3 (team* or program* or intervention* or therap*))) (311*)

S2 SU("Intervention" or "Play Therapy") OR TI,AB(theraplay or (play NEAR/3 (therap* or program or intervention*))) OR TI,AB(circle NEAR/3 security) (32,903*)

S1 ((su(("Attachment Behavior")) OR TI,AB(attachment NEAR/2 (disorder[*1] OR problem[*1] OR style[*1] OR pattern[*1]))) OR TI,AB(attachment NEAR/2 (behavio*r* OR ambivalen* OR avoidant OR diffuse OR organi* OR disorgani* OR disrupt* OR abnormal* OR disinhib* OR inhib*)) OR TI,AB(attachment NEAR/2 (disorienta* OR reactive OR anxious* OR disturb* OR relation* OR interven* OR insecure* OR secure OR security OR early OR theory OR theories))) (2909*)

© Queen's Printer and Controller of HMSO 2015. This work was produced by Wright *et al.* under the terms of a commissioning contract issued by the Secretary of State for Health. This issue may be freely reproduced for the purposes of private research and study and extracts (or indeed, the full report) may be included in professional journals provided that suitable acknowledgement is made and the reproduction is not associated with any form of advertising. Applications for commercial reproduction should be addressed to: NIHR Journals Library, National Institute for Health Research, Evaluation, Trials and Studies Coordinating Centre, Alpha House, University of Southampton Science Park, Southampton SO16 7NS, UK.

Social Services Abstracts (via CSA Illumina)

Date searched: 1979 to December 2011.

Date of search: 11 January 2012.

A total of 99 records were retrieved.

Search strategy

((KW=((attachment within 2 (disorder* or problem* or style* or pattern*)) or (attachment within 2 (behavior* or behavior* or ambivalen* or avoidant or diffuse or organi* or disorgani* or disrupt* or abnormal* or disinhib* or inhib*)) or (attachment within 2 (disorienta* or reactive or anxious* or disturb* or relation*))) or KW=(attachment within 2 (interven* or insecure* or secure or security or early or theory or theories))) and((DE=("adolescents" or "children" or "infants")) or(KW=((child* or infant* or infancy or preschool* or "pre school*" or baby or babies or pediat* or paediat*) or (boy or boys or girl or girls) or (schoolchild* or adolescen* or juvenile* or youth* or teenage* or youngster*)) or KW=("young people" or "young person" or "young persons" or "young adult*" or "early adult*")) or(DE=(Dyads or Child Neglect or Child Abuse or Foster Care or Foster Children or Adoption or Adopted Children) or KW=((parent* or mother* or maternal* or mum* or father* or paternal* or dad*) or (dyad* or attunement) or (representation* within 2 model*)) or KW=((neglect* or abuse or abused or abusive or maltreat* or mistreat*) or (foster* or adopt*))))) and((DE=(Intervention or Psychodynamics) or KW=((theraplay or (play within 3 (therap* or program or intervention*))) or ((preschool* or "pre school*" or child* or infant*) within 3 (psychotherap* or "psycho therap*")) or (watch within 2 wait)) or KW=(((interaction or interactive) within 3 guidance) or (biobehavio* or "bio behavio*") or (("New Orleans" within 3 (intervention* or program* or therap*)) or (tulane within 3 (team* or program* or intervention* or therap*))))) or(KW=(((parent* or mother* or father* or dyad*) within 3 (psychotherap* or "psycho therap*")) or ((parent* or child*) within 2 game*) or (floortime or (floor within 2 time))) or KW=(((manipulat* within 3 respons*) or (Leiden within 3 (program* or intervention* or therap*))) or ((preschool* or "pre school*" or child* or infant*) within 3 (psychotherap* or "psycho therap*")) or (watch within 2 wait)) or KW=(((interaction or interactive) within 3 guidance) or (modif* within 3 guidance) or (video* or VIPP or VIG))) or(KW=(((clinician* within 3 exposure*) or CAVES) or (Tamars within 3 Children*) or (Florida within 3 (program* or intervention* or therap*))) or KW=((psychodynamic within 3 psychotherap*) or ((story or stories) within 3 stem*) or ((home or hospital or family) within 3 visit*)) or KW=("Project CARE" or "Orion Project")) or(KW=(((violent within 3 resistan*) or (nonviolent within 3 resistan*) or NVR) or (cues within 3 clues) or (mellow within 3 (baby or babies or parent*))) or KW=((solihull or bath or bathe or bathing or massag* or tickl*) or (self within 2 regulat*) or (personal within 3 contact*)) or KW=(((baby or babies or infant*) within 2 (carrier* or carry*)) or ((feed* or food or water) within 3 (therap* or program or intervention*)) or (holding or restrain* or "rage reduc*" or rebirth*))))

Applied Social Sciences Index and Abstracts (ASSIA; via CSA Illumina)

Date searched: 1987 to December 2011.

Date of search: 11 January 2012.

A total of 109 records were retrieved.

Search strategy

((KW=((attachment within 2 (disorder* or problem* or style* or pattern*)) or (attachment within 2 (behavior* or behavior* or ambivalen* or avoidant or diffuse or organi* or disorgani* or disrupt* or abnormal* or disinhib* or inhib*)) or (attachment within 2 (disorienta* or reactive or anxious* or disturb* or relation*))) or KW=(attachment within 2 (interven* or insecure* or secure or security or early or theory or theories))) or(DE="attachment disorders")) and(DE=((Children or Infants or Adolescents) or (Parents or

Dyads) or (Child neglect or Child abuse or Foster Care or Foster children or Adoption or Adopted children or Adoptive parents)) or KW=((child* or infant* or infancy or preschool* or "pre school*" or baby or babies or pediat* or paediat*) or (boy or boys or girl or girls) or (schoolchild* or adolescen* or juvenile* or youth* or teenage* or youngster* or "young people" or "young person" or "young persons" or "young adult*" or "early adult*")) or KW=((parent* or mother* or maternal* or mum* or father* or paternal* or dad* or dyad* or attunement) or (representation* within 2 model*) or (neglect* or abuse or abused or abusive or maltreat* or mistreat* or foster* or adopt*))) and((DE=(Interventions or Psychodynamics or Play therapy) or KW=((theraplay or (play within 3 (therap* or program or intervention*))) or ((preschool* or "pre school*" or child* or infant*) within 3 (psychotherap* or "psycho therap*")) or (watch within 2 wait)) or KW=(((interaction or interactive) within 3 guidance) or (biobehavio* or "bio behavio*") or (("New Orleans" within 3 (intervention* or program* or therap*)) or (tulane within 3 (team* or program* or intervention* or therap*))))) or(KW=(((parent* or mother* or father* or dyad*) within 3 (psychotherap* or "psycho therap*")) or ((parent* or child*) within 2 game*) or (floortime or (floor within 2 time))) or KW=(((manipulat* within 3 respons*) or (Leiden within 3 (program* or intervention* or therap*))) or ((preschool* or "pre school*" or child* or infant*) within 3 (psychotherap* or "psycho therap*")) or (watch within 2 wait)) or KW=(((interaction or interactive) within 3 guidance) or (modif* within 3 guidance) or (video* or VIPP or VIG))) or(KW=(((clinician* within 3 exposure*) or CAVES) or (Tamars within 3 Children*) or (Florida within 3 (program* or intervention* or therap*))) or KW=((psychodynamic within 3 psychotherap*) or ((story or stories) within 3 stem*) or ((home or hospital or family) within 3 visit*)) or KW=("Project CARE" or "Orion Project")) or(KW=(((violent within 3 resistan*) or (nonviolent within 3 resistan*) or NVR) or (cues within 3 clues) or (mellow within 3 (baby or babies or parent*))) or KW=((solihull or bath or bathe or bathing or massag* or tickl*) or (self within 2 regulat*) or (personal within 3 contact*)) or KW=(((baby or babies or infant*) within 2 (carrier* or carry*)) or ((feed* or food or water) within 3 (therap* or program or intervention*)) or (holding or restrain* or "rage reduc*" or rebirth*))))

Social Care Online (via SCIE)

Date searched: inception to 2012.

Date of search: 12 January 2012.

A total of 196 records were retrieved.

Advanced search option

(topic"attachment" or freetext="attachment") AND (topic="children" or topic="babies" or topic="young people" or topic="child abuse" or topic="child neglect" or topic="adoption" or topic="adoptive parents" or topic="adoptive children" or topic="foster care" or topic="foster children" or freetext="child*" or freetext="infant*" or freetext="infancy" or freetext="preschool*" or freetext="pre school*" or freetext="baby" or freetext="babies" or freetext="pediat*" or freetext="paediat*" or freetext="juvenile*" or freetext="youth*" or freetext="teenage*" or freetext="youngster*" or freetext="young people" or freetext="young person" or freetext="young persons" or freetext="young adult*" or freetext="early adult") AND (topic="Intervention" or topic="play therapy" or freetext=" theraplay" or freetext="play therapy" or freetext="circle of security" or freetext=" psychotherap*" or freetext="biobehavio*" or freetext="new orleans" or freetext="floortime" or freetext="floor time" or freetext="leiden" or freetext="solihull" or freetext="video*" or freetext="psychodynamic psychotherapy*or freetext="floortime" or freetext="cues" or freetext="mellow")

© Queen's Printer and Controller of HMSO 2015. This work was produced by Wright *et al.* under the terms of a commissioning contract issued by the Secretary of State for Health. This issue may be freely reproduced for the purposes of private research and study and extracts (or indeed, the full report) may be included in professional journals provided that suitable acknowledgement is made and the reproduction is not associated with any form of advertising. Applications for commercial reproduction should be addressed to: NIHR Journals Library, National Institute for Health Research, Evaluation, Trials and Studies Coordinating Centre, Alpha House, University of Southampton Science Park, Southampton SO16 7NS, UK.

Controlled trials search strategies

PsycINFO (via OvidSP)

Date searched: 1806 to week 1, January 2012.

Date of search: 6 January 2012.

A total of 858 records were retrieved.

Search strategy

1. attachment behavior/ (13,469)
2. attachment disorders/ (370)
3. attachment theory/ (885)
4. (attachment adj2 (disorder$1 or problem$1 or style$1 or pattern$1)).ti,ab. (4327)
5. (attachment adj2 (behavio?r$ or ambivalen$ or avoidant or diffuse or organi$ or disorgani$ or disrupt$ or abnormal$ or disinhib$ or inhib$)).ti,ab. (2562)
6. (attachment adj2 (disorienta$ or reactive or anxious$ or disturb$ or relation$)).ti,ab. (3582)
7. (attachment adj2 (interven$ or insecure$ or secure or security or early or theory or theories)).ti,ab. (7456)
8. or/1-7 (17,172)
9. (double-blind or random$ assigned or control).tw. [HEDGES Best Balance] (291,583)
10. 8 and 9 (1187)
11. (comment reply or editorial or letter or reprint or "review book" or "review media" or "review software other").dt. (221,270)
12. (animal or animals or rat or rats or mouse or mice or hamster or hamsters or dog or dogs or cat or cats or bovine or sheep or ovine or pig or pigs).ab,ti,id,de. (232,200)
13. 10 not (11 or 12) (1120)
14. (infancy 2 23 mo or neonatal birth 1 mo or preschool age 2 5 yrs).ag. (113,808)
15. (adolescence 13 17 yrs or childhood birth 12 yrs or school age 6 12 yrs).ag. (524,966)
16. (child$ or infant$ or infancy or preschool$ or pre school$ or baby or babies or pediat$ or paediat$).ti,ab. (504,891)
17. (boy or boys or girl or girls).ti,ab. (69,790)
18. (schoolchild$ or adolescen$ or juvenile$ or youth$ or teenage$ or youngster$).ti,ab. (190,092)
19. (young people or young person or young persons or young adult$ or early adult$).ti,ab. (38,372)
20. exp Parents/ (62,079)
21. exp Parenting/ (64,945)
22. (parent$ or mother$ or maternal$ or mum$ or father$ or paternal$ or dad$).ti,ab. (242,087)
23. Dyads/ (3998)
24. dyad$.ti,ab. (18,706)
25. (attunement or (representation$ adj2 model$)).ti,ab. (1587)
26. exp Child Neglect/ or exp Child Abuse/ (21,046)
27. exp Foster Children/ or exp Foster Care/ or exp Foster Parents/ (4034)
28. exp "Adoption (Child)"/ or exp Adoptive Parents/ (2891)
29. (neglect$ or abuse or abused or abusive or maltreat$ or mistreat$).ti,ab. (104,195)
30. (foster$ or adopt$).ti,ab. (69,814)
31. or/14-30 (955,446)
32. 13 and 31 (858)

MEDLINE and MEDLINE In-Process & Other Non-Indexed Citations (via OvidSP)

Date searched: 1946 to week 4, December 2011.

Date of search: 9 January 2012.

A total of 327 records were retrieved in MEDLINE, and 17 in MEDLINE In-Process.

Search strategy

1. Reactive Attachment Disorder/ (296)
2. (attachment adj2 (disorder$1 or problem$1 or style$1 or pattern$1)).ti,ab. (1100)
3. (attachment adj2 (behavio?r$ or ambivalen$ or avoidant or diffuse or organi$ or disorgani$ or disrupt$ or abnormal$ or disinhib$ or inhib$)).ti,ab. (2250)
4. (attachment adj2 (disorienta$ or reactive or anxious$ or disturb$ or relation$)).ti,ab. (842)
5. (attachment adj2 (interven$ or insecure$ or secure or security or early or theory or theories)).ti,ab. (1795)
6. or/1-5 (4718)
7. randomized controlled trial.pt. (315,877)
8. controlled clinical trial.pt. (83,182)
9. randomized.ab. (221,432)
10. placebo.ab. (127,183)
11. drug therapy.fs. (1,488,786)
12. randomly.ab. (160,369)
13. trial.ab. (228,368)
14. groups.ab. (1,061,229)
15. or/7-14 (2,757,907)
16. 6 and 15 (587)
17. animals/ not (animals/ and humans/) (3,548,684)
18. (letter or editorial or comment or news or newspaper article).pt. (1,231,519)
19. 16 not (17 or 18) (524)
20. exp Child/ (1,400,869)
21. exp Infant/ (854,319)
22. Adolescent/ (1,434,825)
23. (child$ or infant$ or infancy or preschool$ or pre school$ or baby or babies or pediat$ or paediat$).ti,ab. (1,125,683)
24. (boy or boys or girl or girls).ti,ab. (136,911)
25. (schoolchild$ or adolescen$ or juvenile$ or youth$ or teenage$ or youngster$).ti,ab. (214,069)
26. (young people or young person or young persons or young adult$ or early adult$).ti,ab. (60,337)
27. exp Parents/ (60,696)
28. exp Parent-Child Relations/ or Parenting/ (45,480)
29. (parent$ or mother$ or maternal$ or mum$ or father$ or paternal$ or dad$).ti,ab. (520,407)
30. dyad$.ti,ab. (7450)
31. (attunement or (representation$ adj2 model$)).ti,ab. (692)
32. Child Abuse/ (15,437)
33. Foster Home Care/ (2730)
34. Adoption/ (3984)
35. (neglect$ or abuse or abused or abusive or maltreat$ or mistreat$).ti,ab. (101,116)
36. (foster$ or adopt$).ti,ab. (116,855)
37. or/20-36 (3,282,233)
38. 19 and 37 (327)

© Queen's Printer and Controller of HMSO 2015. This work was produced by Wright *et al.* under the terms of a commissioning contract issued by the Secretary of State for Health. This issue may be freely reproduced for the purposes of private research and study and extracts (or indeed, the full report) may be included in professional journals provided that suitable acknowledgement is made and the reproduction is not associated with any form of advertising. Applications for commercial reproduction should be addressed to: NIHR Journals Library, National Institute for Health Research, Evaluation, Trials and Studies Coordinating Centre, Alpha House, University of Southampton Science Park, Southampton SO16 7NS, UK.

EMBASE (via OvidSP)

Date searched: 1974 to week 1, 2012.

Date of search: 10 January 2012.

A total of 306 records were retrieved.

Search strategy

1. (attachment adj2 (disorder$1 or problem$1 or style$1 or pattern$1)).ti,ab. (1565)
2. (attachment adj2 (behavio?r$ or ambivalen$ or avoidant or diffuse or organi$ or disorgani$ or disrupt$ or abnormal$ or disinhib$ or inhib$)).ti,ab. (2670)
3. (attachment adj2 (disorienta$ or reactive or anxious$ or disturb$ or relation$)).ti,ab. (1148)
4. (attachment adj2 (interven$ or insecure$ or secure or security or early or theory or theories)).ti,ab. (2513)
5. or/1-4 (5956)
6. random.tw. (154,661)
7. clinical trial.mp. (891,809)
8. exp Health Care Quality/ (1,569,346)
9. or/6-8 [HEDGES trials filter] (2,354,119)
10. 5 and 9 (493)
11. Animal/ or Animal Experiment/ or Nonhuman/ (5,761,726)
12. (rat or rats or mouse or mice or murine or rodent or rodents or hamster or hamsters or pig or pigs or porcine or rabbit or rabbits or animal or animals or dogs or dog or cats or cow or bovine or sheep or ovine or monkey or monkeys).ti,ab,sh. (4,749,774)
13. 11 or 12 (6,446,779)
14. exp Human/ or Human Experiment/ (12,937,340)
15. 13 not (13 and 14) (5,116,251)
16. (editorial or letter or note).pt. (1,613,483)
17. 10 not (15 or 16) (474)
18. child/ (1,135,530)
19. infant/ (476,014)
20. adolescent/ (1,127,803)
21. (child$ or infant$ or infancy or preschool$ or pre school$ or baby or babies or pediat$ or paediat$).ti,ab. (1,413,823)
22. (boy or boys or girl or girls).ti,ab. (177,580)
23. (schoolchild$ or adolescen$ or juvenile$ or youth$ or teenage$ or youngster$).ti,ab. (272,836)
24. (young people or young person or young persons or young adult$ or early adult$).ti,ab. (76,703)
25. exp parent/ (114,307)
26. exp child parent relation/ (58,704)
27. (parent$ or mother$ or maternal$ or mum$ or father$ or paternal$ or dad$).ti,ab. (911,848)
28. dyad$.ti,ab. (9350)
29. (attunement or (representation$ adj2 model$)).ti,ab. (894)
30. child abuse/ or child neglect/ (21,051)
31. foster care/ (3077)
32. adoption/ or adopted child/ (4815)
33. (neglect$ or abuse or abused or abusive or maltreat$ or mistreat$).ti,ab. (131,980)
34. (foster$ or adopt$).ti,ab. (150,939)
35. or/18-34 (3,567,803)
36. 17 and 35 (306)

Social Policy & Practice (via OvidSP)

Date searched: inception to 2012.

Date of search: 10 January 2012.

A total of 166 records were retrieved.

Search strategy

1. attachment disorder.de. (232)
2. (attachment adj2 (disorder$1 or problem$1 or style$1 or pattern$1)).ti,ab. (390)
3. (attachment adj2 (behavio?r$ or ambivalen$ or avoidant or diffuse or organi$ or disorgani$ or disrupt$ or abnormal$ or disinhib$ or inhib$)).ti,ab. (196)
4. (attachment adj2 (disorienta$ or reactive or anxious$ or disturb$ or relation$)).ti,ab. (265)
5. (attachment adj2 (interven$ or insecure$ or secure or security or early or theory or theories)).ti,ab. (824)
6. or/1-5 (1309)
7. (random$ or clin$ trial$ or control$ or prospectiv$ or placebo$).ti,ab,de. (20,123)
8. ((singl$ or doubl$ or tripl$ or trebl$) adj3 (blind$ or mask$)).ti,ab,de. (83)
9. ((case control$ or cohort$ or prospectiv$ or quantitativ$ or longitudinal or comparator or comparison or comparative or control$ or evaluation or followup or follow up or intervention or multicenter$ or multi center$ or multicentre$ or multi centre$ or family or open) adj3 (study or studies or trial$ or group or groups or series)).ti,ab,de. (13,403)
10. or/7-9 (28,407)
11. 6 and 10 (189)
12. (child$ or infant$ or infancy or preschool$ or pre school$ or baby or babies or pediat$ or paediat$).ti,ab,de. (125,901)
13. (boy or boys or girl or girls).ti,ab,de. (5692)
14. (schoolchild$ or adolescen$ or juvenile$ or youth$ or teenage$ or youngster$).ti,ab,de. (32,056)
15. (young people or young person or young persons or young adult$ or early adult$).ti,ab,de. (42,212)
16. (parent$ or mother$ or maternal$ or mum$ or father$ or paternal$ or dad$).ti,ab,de. (47,034)
17. dyad$.ti,ab,de. (480)
18. (attunement or (representation$ adj2 model$)).ti,ab,de. (66)
19. (neglect$ or abuse or abused or abusive or maltreat$ or mistreat$).ti,ab,de. (23,203)
20. (foster$ or adopt$).ti,ab,de. (19,994)
21. or/12-20 (172,573)
22. 11 and 21 (166)

Science Citation Index (SCI; via ISI Web of Science)

Date searched: 1899 to 6 January 2012.

Date of search: 10 January 2012.

A total of 362 records were retrieved.

Databases=SCI-EXPANDED Timespan=All Years.

Lemmatization=Off.

© Queen's Printer and Controller of HMSO 2015. This work was produced by Wright *et al.* under the terms of a commissioning contract issued by the Secretary of State for Health. This issue may be freely reproduced for the purposes of private research and study and extracts (or indeed, the full report) may be included in professional journals provided that suitable acknowledgement is made and the reproduction is not associated with any form of advertising. Applications for commercial reproduction should be addressed to: NIHR Journals Library, National Institute for Health Research, Evaluation, Trials and Studies Coordinating Centre, Alpha House, University of Southampton Science Park, Southampton SO16 7NS, UK.

Search strategy

23 #21 not #22 (362)

22 TS=(rat or rats or mouse or mice or murine or hamster or hamsters or animal or animals or dogs or dog or pig or pigs or cats or bovine or cow or sheep or ovine or porcine or monkey) (3,408,525)

21 #10 and #20 (389)

20 #11 OR #12 OR #13 OR #14 OR #15 OR #16 OR #17 OR #18 OR #19 (1,972,974)

19 TS=(foster* or adopt*) (207,538)

18 TS=(neglect* or abuse or abused or abusive or maltreat* or mistreat*) (117,173)

17 TS=(attunement or (representation* NEAR/2 model*)) (6475)

16 TS=dyad* (10,857)

15 TS=(parent* or mother* or maternal* or mum* or father* or paternal* or dad*) (574,227)

14 TS=("young people" or "young person" or "young persons" or "young adult*" or "early adult*") (55,963)

13 TS=(schoolchild* or adolescen* or juvenile* or youth* or teenage* or youngster*) (249,910)

12 TS=(boy or boys or girl or girls) (96,380)

11 TS=(child* or infant* or infancy or preschool* or "pre school*" or baby or babies or pediat* or paediat*) (1,021,056)

10 #5 and #9 (867)

9 #6 or #7 or #8 (4,236,177)

8 TS=((singl* or doubl* or tripl* or trebl*) NEAR/2 (blind* or mask*)) (152,011)

7 TS=(random* or "clin* trial*" or "controlled study" or "controlled studies" or "controlled trial*" or "control* group" or "control* groups" or "control* series" or prospective) (1,293,016)

6 TS=("case control*" or cohort* or quantitative* or longitudinal or comparat* or comparison or evaluation or followup or "follow up" or intervention or multicenter* or "multi center*" or multicentre* or "multi centre*") (3,357,932)

5 #1 OR #2 OR #3 OR #4 (4128)

4 TS=(attachment NEAR/2 (interven* or insecure* or secure or security or early or theory or theories)) (1090)

3 TS=(attachment NEAR/2 (disorienta* or reactive or anxious* or disturb* or relation*)) (593)

2 TS=(attachment NEAR/2 (behavior$r* or ambivalen* or avoidant or diffuse or organi* or disorgani* or disrupt* or abnormal* or disinhib* or inhib*)) (2203)

1 TS=(attachment NEAR/2 (disorder$ or problem$ or style$ or pattern$)) (924)

Social Science Citation Index (SSCI; via ISI Web of Science)

Date searched: 1956 to 6 January 2012.

Date of search: 10 January 2012.

A total 1318 records were retrieved.

Databases=SSCI-EXPANDED Timespan=All Years.

Lemmatization=Off.

Search strategy

23 #21 not #22 (1318)

22 TS=(rat or rats or mouse or mice or murine or hamster or hamsters or animal or animals or dogs or dog or pig or pigs or cats or bovine or cow or sheep or ovine or porcine or monkey) (91,781)

21 #10 and #20 (1336)

20 #11 OR #12 OR #13 OR #14 OR #15 OR #16 or #17 or #18 or #19 (682,942)

19 TS=(foster* or adopt*) (75,821)

18 TS=(neglect* or abuse or abused or abusive or maltreat* or mistreat*) (91,084)

17 TS=(attunement or (representation* NEAR/2 model*)) (2062)

16 TS=dyad* (10,250)

15 TS=(parent* or mother* or maternal* or mum* or father* or paternal* or dad*) (160,559)

14 TS=("young people" or "young person" or "young persons" or "young adult*" or "early adult*") (35,541)

13 TS=(schoolchild* or adolescen* or juvenile* or youth* or teenage* or youngster*) (165,662)

12 TS=(boy or boys or girl or girls) (42,755)

11 TS=(child* or infant* or infancy or preschool* or "pre school*" or baby or babies or pediat* or paediat*) (392,420)

10 #5 and #9 (1635)

9 #6 or #7 or #8 (580,190)

8 TS=((singl* or doubl* or tripl* or trebl*) NEAR/2 (blind* or mask*)) (16,949)

7 TS=(random* or "clin* trial*" or "controlled study" or "controlled studies" or "controlled trial*" or "control* group" or "control* groups" or "control* series" or prospective) (171,684)

6 TS=("case control*" or cohort* or quantitative* or longitudinal or comparat* or comparison or evaluation or followup or "follow up" or intervention or multicenter* or "multi center*" or multicentre* or "multi centre*") (480,117)

© Queen's Printer and Controller of HMSO 2015. This work was produced by Wright *et al.* under the terms of a commissioning contract issued by the Secretary of State for Health. This issue may be freely reproduced for the purposes of private research and study and extracts (or indeed, the full report) may be included in professional journals provided that suitable acknowledgement is made and the reproduction is not associated with any form of advertising. Applications for commercial reproduction should be addressed to: NIHR Journals Library, National Institute for Health Research, Evaluation, Trials and Studies Coordinating Centre, Alpha House, University of Southampton Science Park, Southampton SO16 7NS, UK.

5 #1 OR #2 OR #3 OR #4 (6395)

4 TS=(attachment NEAR/2 (interven* or insecure* or secure or security or early or theory or theories)) (3717)

3 TS=(attachment NEAR/2 (disorienta* or reactive or anxious* or disturb* or relation*)) (2147)

2 TS=(attachment NEAR/2 (behavior$r* or ambivalen* or avoidant or diffuse or organi* or disorgani* or disrupt* or abnormal* or disinhib* or inhib*)) (1017)

1 TS=(attachment NEAR/2 (disorder$ or problem$ or style$ or pattern$)) (2646)

Conference Proceedings Citation Index - Science (CPCI-S; via ISI Web of Science)

Date searched: 1990 to 6 January 2012.

Date of search: 10 January 2012.

A total of 19 records were retrieved.

Databases=CPCI-S-EXPANDED Timespan=All Years.

Lemmatization=Off.

Search strategy

23 #21 not #22 (19)

22 TS=(rat or rats or mouse or mice or murine or hamster or hamsters or animal or animals or dogs or dog or pig or pigs or cats or bovine or cow or sheep or ovine or porcine or monkey) (282,876)

21 #10 and #20 (20)

20 #11 OR #12 OR #13 OR #14 OR #15 OR #16 OR #17 OR #18 OR #19 (291,238)

19 TS=(foster* or adopt*) (87,891)

18 TS=(neglect* or abuse or abused or abusive or maltreat* or mistreat*) (17,504)

17 TS=(attunement or (representation* NEAR/2 model*)) (3792)

16 TS=dyad* (1817)

15 TS=(parent* or mother* or maternal* or mum* or father* or paternal* or dad*) (70,471)

14 TS=("young people" or "young person" or "young persons" or "young adult*" or "early adult*") (5160)

13 TS=(schoolchild* or adolescen* or juvenile* or youth* or teenage* or youngster*) (24,476)

12 TS=(boy or boys or girl or girls) (5857)

11 TS=(child* or infant* or infancy or preschool* or "pre school*" or baby or babies or pediat* or paediat*) (104,538)

10 #5 and #9 (51)

9 #6 or #7 or #8 (788,345)

8 TS=((singl* or doubl* or tripl* or trebl*) NEAR/2 (blind* or mask*)) (15,441)

7 TS=(random* or "clin* trial*" or "controlled study" or "controlled studies" or "controlled trial*" or "control* group" or "control* groups" or "control* series" or prospective) (196,209)

6 TS=("case control*" or cohort* or quantitative* or longitudinal or comparat* or comparison or evaluation or followup or "follow up" or intervention or multicenter* or "multi center*" or multicentre* or "multi centre*") (640,059)

5 #1 OR #2 OR #3 OR #4 (344)

4 TS=(attachment NEAR/2 (interven* or insecure* or secure or security or early or theory or theories)) (94)

3 TS=(attachment NEAR/2 (disorienta* or reactive or anxious* or disturb* or relation*)) (54)

2 TS=(attachment NEAR/2 (behavior$r* or ambivalen* or avoidant or diffuse or organi* or disorgani* or disrupt* or abnormal* or disinhib* or inhib*)) (141)

1 TS=(attachment NEAR/2 (disorder$ or problem$ or style$ or pattern$)) (95)

Conference Proceedings Citation Index – Social Science & Humanities (CPCI-SSH; via ISI Web of Science)

Date searched: 1990 to 6 January 2012.

Date of search: 10 January 2012.

A total of 71 records were retrieved.

Databases=CPCI-SSH Timespan=All Years.

Lemmatization=Off.

Search strategy

23 #21 not #22 (71)

22 TS=(rat or rats or mouse or mice or murine or hamster or hamsters or animal or animals or dogs or dog or pig or pigs or cats or bovine or cow or sheep or ovine or porcine or monkey) (4408)

21 #10 and #20 (71)

20 #11 OR #12 OR #13 OR #14 OR #15 OR #16 OR #17 OR #18 OR #19 (45,707)

19 TS=(foster* or adopt*) (13,580)

18 TS=(neglect* or abuse or abused or abusive or maltreat* or mistreat*) (5468)

17 TS=(attunement or (representation* NEAR/2 model*)) (356)

© Queen's Printer and Controller of HMSO 2015. This work was produced by Wright *et al.* under the terms of a commissioning contract issued by the Secretary of State for Health. This issue may be freely reproduced for the purposes of private research and study and extracts (or indeed, the full report) may be included in professional journals provided that suitable acknowledgement is made and the reproduction is not associated with any form of advertising. Applications for commercial reproduction should be addressed to: NIHR Journals Library, National Institute for Health Research, Evaluation, Trials and Studies Coordinating Centre, Alpha House, University of Southampton Science Park, Southampton SO16 7NS, UK.

16 TS=dyad* (832)

15 TS=(parent* or mother* or maternal* or mum* or father* or paternal* or dad*) (8462)

14 TS=("young people" or "young person" or "young persons" or "young adult*" or "early adult*") (1874)

13 TS=(schoolchild* or adolescen* or juvenile* or youth* or teenage* or youngster*) (7388)

12 TS=(boy or boys or girl or girls) (2090)

11 TS=(child* or infant* or infancy or preschool* or "pre school*" or baby or babies or pediat* or paediat*) (19,667)

10 #5 and #9 (90)

9 #6 or #7 or #8 (53,782)

8 TS=((singl* or doubl* or tripl* or trebl*) NEAR/2 (blind* or mask*)) (1128)

7 TS=(random* or "clin* trial*" or "controlled study" or "controlled studies" or "controlled trial*" or "control* group" or "control* groups" or "control* series" or prospective) (9834)

6 TS=("case control*" or cohort* or quantitative* or longitudinal or comparat* or comparison or evaluation or followup or "follow up" or intervention or multicenter* or "multi center*" or multicentre* or "multi centre*") (47,091)

5 #1 OR #2 OR #3 OR #4 (425)

4 TS=(attachment NEAR/2 (interven* or insecure* or secure or security or early or theory or theories)) (254)

3 TS=(attachment NEAR/2 (disorienta* or reactive or anxious* or disturb* or relation*)) (170)

2 TS=(attachment NEAR/2 (behavior$r* or ambivalen* or avoidant or diffuse or organi* or disorgani* or disrupt* or abnormal* or disinhib* or inhib*)) (83)

1 TS=(attachment NEAR/2 (disorder$ or problem$ or style$ or pattern$)) (143)

Education Resources Information Center (ERIC; via ProQuest)

Date searched: 1966 to December 2011.

Date of search: 11 January 2012.

A total of 450 records were retrieved.

Search strategy

S1 ((su(("Attachment Behavior")) OR TI,AB(attachment NEAR/2 (disorder[*1] OR problem[*1] OR style[*1] OR pattern[*1]))) OR TI,AB(attachment NEAR/2 (behavio*r* OR ambivalen* OR avoidant OR diffuse OR organi* OR disorgani* OR disrupt* OR abnormal* OR disinhib* OR inhib*)) OR TI,AB(attachment NEAR/2 (disorienta* OR reactive OR anxious* OR disturb* OR relation* OR interven* OR insecure* OR secure OR security OR early OR theory OR theories))) 2909*

S2 SU((“Case Studies” OR “Followup Studies” OR “Longitudinal Studies”)) OR TI,AB(“case control*” or cohort* or longitudinal or followup or “follow up” or multicenter* or “multi center*” or multicentre* or “multi centre*”) OR TI,AB(random* or “clin* trial*” or “controlled study” or “controlled studies” or “controlled trial*” or “control* group” or “control* groups” or “control* series” or “prospective study” or “prospective studies” or “prospective trial[*1]”) OR TI,AB((singl* or doubl* or tripl* or trebl*) NEAR/2 (blind* or mask*))108,306*

S3 S1 and S2 450*

Social Services Abstracts (via CSA Illumina)

Date searched: 1979 to December 2011.

Date of search: 11 January 2012.

A total of 125 records were retrieved.

Search strategy

(KW=((attachment within 2 (disorder* or problem* or style* or pattern*)) or (attachment within 2 (behavior* or behavior* or ambivalen* or avoidant or diffuse or organi* or disorgani* or disrupt* or abnormal* or disinhib* or inhib*)) or (attachment within 2 (disorienta* or reactive or anxious* or disturb* or relation*))) or KW=(attachment within 2 (interven* or insecure* or secure or security or early or theory or theories))) and((DE=(“adolescents” or “children” or “infants”)) or(KW=((child* or infant* or infancy or preschool* or “pre school*” or baby or babies or pediat* or paediat*) or (boy or boys or girl or girls) or (schoolchild* or adolescen* or juvenile* or youth* or teenage* or youngster*)) or KW=(“young people” or “young person” or “young persons” or “young adult*” or “early adult*”)) or(DE=(Dyads or Child Neglect or Child Abuse or Foster Care or Foster Children or Adoption or Adopted Children) or KW=((parent* or mother* or maternal* or mum* or father* or paternal* or dad*) or (dyad* or attunement) or (representation* within 2 model*)) or KW=((neglect* or abuse or abused or abusive or maltreat* or mistreat*) or (foster* or adopt*)))) and(DE=((Longitudinal Studies) or (Case Studies) or (Cohort Analysis)) or KW=(((case control* or cohort* or longitudinal or followup or “follow up” or multicenter* or “multi center*” or multicentre* or “multi centre*” or family or open) within 3 (study or studies or trial* or group or groups or series)) or (random* or “clin* trial*” or control* or prospectiv* or placebo*) or ((singl* or doubl* or tripl* or trebl*) within 3 (blind* or mask*))))

Applied Social Sciences Index and Abstracts (ASSIA; via CSA Illumina)

Date searched: 1987 to December 2011.

Date of search: 11 January 2012.

A total of 312 records were retrieved.

Search strategy

(((KW=((attachment within 2 (disorder* or problem* or style* or pattern*)) or (attachment within 2 (behavior* or behavior* or ambivalen* or avoidant or diffuse or organi* or disorgani* or disrupt* or abnormal* or disinhib* or inhib*)) or (attachment within 2 (disorienta* or reactive or anxious* or disturb* or relation*))) or KW=(attachment within 2 (interven* or insecure* or secure or security or early or theory or theories))) or(DE=“attachment disorders”)) and(DE=((Children or Infants or Adolescents) or (Parents or Dyads) or (Child neglect or Child abuse or Foster Care or Foster children or Adoption or Adopted children or Adoptive parents)) or KW=((child* or infant* or infancy or preschool* or “pre school*” or baby or babies or pediat* or paediat*) or (boy or boys or girl or girls) or (schoolchild* or adolescen* or juvenile* or youth* or teenage* or youngster* or “young people” or “young person” or “young persons” or “young adult*” or “early adult*”)) or KW=((parent* or mother* or maternal* or mum* or father* or paternal* or dad* or dyad* or attunement) or (representation* within 2 model*) or (neglect* or abuse or abused or

© Queen’s Printer and Controller of HMSO 2015. This work was produced by Wright et al. under the terms of a commissioning contract issued by the Secretary of State for Health. This issue may be freely reproduced for the purposes of private research and study and extracts (or indeed, the full report) may be included in professional journals provided that suitable acknowledgement is made and the reproduction is not associated with any form of advertising. Applications for commercial reproduction should be addressed to: NIHR Journals Library, National Institute for Health Research, Evaluation, Trials and Studies Coordinating Centre, Alpha House, University of Southampton Science Park, Southampton SO16 7NS, UK.

abusive or maltreat* or mistreat* or foster* or adopt*)))) and(((“case control*” or cohort* or quantitativ* or longitudinal or comparator or comparison or comparative or evaluation or followup or “follow up” or intervention or multicenter* or “multi center*” or multicentre* or “multi centre*” or family or open) within 3 (study or studies or trial* or group or groups or series)) or (DE=(Case controlled studies or Cohort analysis or Quantitative methods or Quantitative analysis or Longitudinal studies or Comparative research or Comparative studies or Comparative approaches or Evaluation designs or Evaluation or Followup studies or Followup)) or (random* or “clin* trial*” or control* or prospectiv* or placebo*) or ((singl* or doubl* or tripl* or trebl*) within 3 (blind* or mask*)) or (DE=(Randomization or Randomized consent design or Randomized controlled trials or Clinical randomized controlled trials or Cluster randomized controlled trials or Double blind randomized controlled trials or Single blind randomized controlled trials or Urn randomization or Clinical trials or Double blind randomized trials or Placebos or Placebo effect or Control groups or Prospective controlled trials or Prospective studies)))

Social Care Online (via SCIE)

Date searched: inception to 2012.

Date of search: 12 January 2012.

A total of 119 records were retrieved.

Advanced search option

(topic“attachment” or freetext=“attachment”) AND (topic=“children” or topic=“babies” or topic=“young people” or topic=“child abuse” or topic=“child neglect” or topic=“adoption” or topic=“adoptive parents” or topic=“adoptive children” or topic=“foster care” or topic=“foster children” or freetext=“child*” or freetext=“infant*” or freetext=“infancy” or freetext=“preschool*” or freetext=“pre school*” or freetext=“baby” or freetext=“babies” or freetext=“pediat*” or freetext=“paediat*” or freetext=“juvenile*” or freetext=“youth*” or freetext=“teenage*” or freetext=“youngster*” or freetext=“young people” or freetext=“young person” or freetext=“young persons” or freetext=“young adult*” or freetext=“early adult”) AND (topic=“randomised controlled trials” or topic=“case studies” or topic=“longitudinal studies” or freetext=” case control*” or freetext=“cohort stud*” or freetext=“longitudinal stud*” or freetext=“follow up stud*” or freetext=“multicent* stud*” or freetext=“multi cent* stud*” or freetext=“random*” or freetext=“clin* trial*” or freetext=“prospective*”)

Cochrane Central Register of Controlled Trials (via The Cochrane Library)

Issue 4, 2011.

Date searched: inception to 2012.

Date of search: 12 January 2012.

A total of 193 records were retrieved.

Search strategy

#1 MeSH descriptor Reactive Attachment Disorder explode all trees 9

#2 (attachment NEAR/3 (disorder* or problem* or style* or pattern*)):ti,ab,kw 59

#3 (attachment NEAR/3 (behavio?r* or ambivalen* or avoidant or diffuse or organi* or disorgani* or disrupt* or abnormal* or disinhib* or inhib*)):ti,ab,kw 31

#4 (attachment NEAR/3 (disorienta* or reactive or anxious* or disturb* or relation*)):ti,ab,kw 108

#5 (attachment NEAR/3 (interven* or insecure* or secure or security or early or theory or theories)):ti,ab,kw 81

#6 (#1 OR #2 OR #3 OR #4 OR #5) 199

[Line #6 includes the results from all The Cochrane Library databases: CDSR 3, DARE 1, CENTRAL 193, HTA 1, and NHS EED 1]

Economics/costs search strategies

PsycINFO (via OvidSP)

Date searched: 1806 to week 1, January 2012.

Date of search: 6 January 2012.

A total of 282 records were retrieved.

Search strategy

1. attachment behavior/ (13,469)
2. attachment disorders/ (370)
3. attachment theory/ (885)
4. (attachment adj2 (disorder$1 or problem$1 or style$1 or pattern$1)).ti,ab. (4327)
5. (attachment adj2 (behavio?r$ or ambivalen$ or avoidant or diffuse or organi$ or disorgani$ or disrupt$ or abnormal$ or disinhib$ or inhib$)).ti,ab. (2562)
6. (attachment adj2 (disorienta$ or reactive or anxious$ or disturb$ or relation$)).ti,ab. (3582)
7. (attachment adj2 (interven$ or insecure$ or secure or security or early or theory or theories)).ti,ab. (7456)
8. or/1-7 (17,172)
9. exp "Costs and Cost Analysis"/ (15,039)
10. health care costs/ (5358)
11. "cost containment"/ (429)
12. (econom$ or cost or costs or costly or costing or price or prices or pricing or pharmacoeconomic$).ti,ab,id. (122,816)
13. (expenditure$ not energy).ti,ab,id. (4131)
14. (value adj2 money).ti,ab,id. (238)
15. budget$.ti,ab,id. (4568)
16. (willingness adj2 pay).ti,ab,id. (731)
17. or/9-16 (129,872)
18. (task adj2 cost$).ti,ab,id. (267)
19. (switch$ adj2 cost$).ti,ab,id. (585)
20. (metabolic adj cost).ti,ab,id. (45)
21. ((energy or oxygen) adj cost).ti,ab,id. (163)
22. ((energy or oxygen) adj expenditure).ti,ab,id. (1485)
23. or/18-22 (2390)
24. (animal or animals or rat or rats or mouse or mice or hamster or hamsters or dog or dogs or cat or cats or bovine or sheep or ovine or pig or pigs).ab,ti,id,de. (232,200)
25. 17 not (23 or 24) (125,660)
26. 8 and 25 (298)
27. (comment reply or editorial or letter or reprint or "review book" or "review media" or "review software other").dt. (221,270)
28. 26 not 27 (282)

© Queen's Printer and Controller of HMSO 2015. This work was produced by Wright *et al.* under the terms of a commissioning contract issued by the Secretary of State for Health. This issue may be freely reproduced for the purposes of private research and study and extracts (or indeed, the full report) may be included in professional journals provided that suitable acknowledgement is made and the reproduction is not associated with any form of advertising. Applications for commercial reproduction should be addressed to: NIHR Journals Library, National Institute for Health Research, Evaluation, Trials and Studies Coordinating Centre, Alpha House, University of Southampton Science Park, Southampton SO16 7NS, UK.

MEDLINE and MEDLINE In-Process & Other Non-Indexed Citations (via OvidSP)

Date searched: 1946 to week 4, December 2011.

Date of search: 9 January 2012

A total of 47 records were retrieved in MEDLINE, and one in MEDLINE In-Process.

Search strategy

1. Reactive Attachment Disorder/ (296)
2. (attachment adj2 (disorder$1 or problem$1 or style$1 or pattern$1)).ti,ab. (1100)
3. (attachment adj2 (behavio?r$ or ambivalen$ or avoidant or diffuse or organi$ or disorgani$ or disrupt$ or abnormal$ or disinhib$ or inhib$)).ti,ab. (2250)
4. (attachment adj2 (disorienta$ or reactive or anxious$ or disturb$ or relation$)).ti,ab. (842)
5. (attachment adj2 (interven$ or insecure$ or secure or security or early or theory or theories)).ti,ab. (1795)
6. or/1-5 (4718)
7. economics/ (26,133)
8. exp "costs and cost analysis"/ (159,926)
9. economics, dental/ (1833)
10. exp "economics, hospital"/ (17,522)
11. economics, medical/ (8420)
12. economics, nursing/ (3852)
13. economics, pharmaceutical/ (2276)
14. (economic$ or cost or costs or costly or costing or price or prices or pricing or pharmacoeconomic$).ti,ab. (346,791)
15. (expenditure$ not energy).ti,ab. (14,586)
16. (value adj1 money).ti,ab. (17)
17. budget$.ti,ab. (14,854)
18. or/7-17 (460,918)
19. ((energy or oxygen) adj cost).ti,ab. (2355)
20. (metabolic adj cost).ti,ab. (617)
21. ((energy or oxygen) adj expenditure).ti,ab. (13,435)
22. or/19-21 (15,780)
23. 18 not 22 (457,312)
24. 6 and 23 (73)
25. animals/ not (animals/ and humans/) (3,548,684)
26. (letter or editorial or comment or news or newspaper article).pt. (1,231,519)
27. 24 not (25 or 26) (66)
28. exp Child/ (1,400,869)
29. exp Infant/ (854,319)
30. Adolescent/ (1,434,825)
31. (child$ or infant$ or infancy or preschool$ or pre school$ or baby or babies or pediat$ or paediat$).ti,ab. (1,125,683)
32. (boy or boys or girl or girls).ti,ab. (136,911)
33. (schoolchild$ or adolescen$ or juvenile$ or youth$ or teenage$ or youngster$).ti,ab. (214,069)
34. (young people or young person or young persons or young adult$ or early adult$).ti,ab. (60,337)
35. exp Parents/ (60,696)
36. exp Parent-Child Relations/ or Parenting/ (45,480)
37. (parent$ or mother$ or maternal$ or mum$ or father$ or paternal$ or dad$).ti,ab. (520,407)
38. dyad$.ti,ab. (7450)
39. (attunement or (representation$ adj2 model$)).ti,ab. (692)
40. Child Abuse/ (15,437)
41. Foster Home Care/ (2730)

42. Adoption/ (3984)
43. (neglect$ or abuse or abused or abusive or maltreat$ or mistreat$).ti,ab. (101,116)
44. (foster$ or adopt$).ti,ab. (116,855)
45. or/28-44 (3,282,233)
46. 27 and 45 (47)

EMBASE (via OvidSP)

Date searched: 1974 to week 1, 2012.

Date of search: 10 January 2012.

A total of 67 records were retrieved.

Search strategy

1. (attachment adj2 (disorder$1 or problem$1 or style$1 or pattern$1)).ti,ab. (1565)
2. (attachment adj2 (behavio?r$ or ambivalen$ or avoidant or diffuse or organi$ or disorgani$ or disrupt$ or abnormal$ or disinhib$ or inhib$)).ti,ab. (2670)
3. (attachment adj2 (disorienta$ or reactive or anxious$ or disturb$ or relation$)).ti,ab. (1148)
4. (attachment adj2 (interven$ or insecure$ or secure or security or early or theory or theories)).ti,ab. (2513)
5. or/1-4 (5956)
6. Health Economics/ (31,517)
7. exp Economic Evaluation/ (176,759)
8. exp Health Care Cost/ (170,263)
9. exp PHARMACOECONOMICS/ (142,949)
10. or/6-9 (404,965)
11. (econom$ or cost or costs or costly or costing or price or prices or pricing or pharmacoeconomic$).ti,ab. (479,601)
12. (expenditure$ not energy).ti,ab. (19,251)
13. (value adj2 money).ti,ab. (1033)
14. budget$.ti,ab. (20,159)
15. or/11-14 (499,971)
16. 10 or 15 (737,326)
17. (metabolic adj cost).ti,ab. (712)
18. ((energy or oxygen) adj cost).ti,ab. (2782)
19. ((energy or oxygen) adj expenditure).ti,ab. (16,168)
20. or/17-19 (18,942)
21. 16 not 20 (732,958)
22. Animal/ or Animal Experiment/ or Nonhuman/ (5,761,726)
23. (rat or rats or mouse or mice or murine or rodent or rodents or hamster or hamsters or pig or pigs or porcine or rabbit or rabbits or animal or animals or dogs or dog or cats or cow or bovine or sheep or ovine or monkey or monkeys).ti,ab,sh. (4,749,774)
24. 22 or 23 (6,446,779)
25. exp Human/ or Human Experiment/ (12,937,340)
26. 24 not (24 and 25) (5,116,251)
27. (editorial or letter or note).pt. (1,613,483)
28. 5 and 21 (105)
29. 28 not (26 or 27) (98)
30. child/ (1,135,530)
31. infant/ (476,014)

© Queen's Printer and Controller of HMSO 2015. This work was produced by Wright *et al.* under the terms of a commissioning contract issued by the Secretary of State for Health. This issue may be freely reproduced for the purposes of private research and study and extracts (or indeed, the full report) may be included in professional journals provided that suitable acknowledgement is made and the reproduction is not associated with any form of advertising. Applications for commercial reproduction should be addressed to: NIHR Journals Library, National Institute for Health Research, Evaluation, Trials and Studies Coordinating Centre, Alpha House, University of Southampton Science Park, Southampton SO16 7NS, UK.

32. adolescent/ (1,127,803)
33. (child$ or infant$ or infancy or preschool$ or pre school$ or baby or babies or pediat$ or paediat$).ti,ab. (1,413,823)
34. (boy or boys or girl or girls).ti,ab. (177,580)
35. (schoolchild$ or adolescen$ or juvenile$ or youth$ or teenage$ or youngster$).ti,ab. (272,836)
36. (young people or young person or young persons or young adult$ or early adult$).ti,ab. (76,703)
37. exp parent/ (114,307)
38. exp child parent relation/ (58,704)
39. (parent$ or mother$ or maternal$ or mum$ or father$ or paternal$ or dad$).ti,ab. (911,848)
40. dyad$.ti,ab. (9350)
41. (attunement or (representation$ adj2 model$)).ti,ab. (894)
42. child abuse/ or child neglect/ (21,051)
43. foster care/ (3077)
44. adoption/ or adopted child/ (4815)
45. (neglect$ or abuse or abused or abusive or maltreat$ or mistreat$).ti,ab. (131,980)
46. (foster$ or adopt$).ti,ab. (150,939)
47. or/30-46 (3,567,803)
48. 29 and 47 (67)

Social Policy & Practice (via OvidSP)

Date searched: inception to 2012.

Date of search: 10 January 2012.

A total of 36 records were retrieved.

Search strategy

1. attachment disorder.de. (232)
2. (attachment adj2 (disorder$1 or problem$1 or style$1 or pattern$1)).ti,ab. (390)
3. (attachment adj2 (behavio?r$ or ambivalen$ or avoidant or diffuse or organi$ or disorgani$ or disrupt$ or abnormal$ or disinhib$ or inhib$)).ti,ab. (196)
4. (attachment adj2 (disorienta$ or reactive or anxious$ or disturb$ or relation$)).ti,ab. (265)
5. (attachment adj2 (interven$ or insecure$ or secure or security or early or theory or theories)).ti,ab. (824)
6. or/1-5 (1309)
7. (econom$ or cost or costs or costly or costing or price or prices or pricing or pharmacoeconomic$).ti,ab,de. (38,824)
8. 6 and 7 (36)

Science Citation Index (SCI; were ISI Web of Science)

Date searched: 1899 to 6 January 2012.

Date of search: 10 January 2012.

A total of 22 records were retrieved.

Databases=SCI-EXPANDED Timespan=All Years.

Lemmatization=Off.

Search strategy

20 #18 NOT #19 (22)

19 TS=(rat or rats or mouse or mice or murine or hamster or hamsters or animal or animals or dogs or dog or pig or pigs or cats or bovine or cow or sheep or ovine or porcine or monkey) (3,408,525)

18 #7 and #17 (24)

17 #8 OR #9 OR #10 OR #11 OR #12 OR #13 OR #14 OR #15 OR #16 (1,972,974)

16 TS=(foster* or adopt*) (207,538)

15 TS=(neglect* or abuse or abused or abusive or maltreat* or mistreat*) (117,173)

14 TS=(attunement or (representation* NEAR/2 model*)) (6475)

13 TS=dyad* (10,857)

12 TS=(parent* or mother* or maternal* or mum* or father* or paternal* or dad*) (574,227)

11 TS=("young people" or "young person" or "young persons" or "young adult*" or "early adult*") (55,963)

10 TS=(schoolchild* or adolescen* or juvenile* or youth* or teenage* or youngster*) (249,910)

9 TS=(boy or boys or girl or girls) (96,380)

8 TS=(child* or infant* or infancy or preschool* or "pre school*" or baby or babies or pediat* or paediat*) (1,021,056)

7 #5 and #6 (61)

6 TS=(econom* or cost or costs or costly or costing or price or prices or pricing or pharmacoeconomic*) (684,586)

5 #1 OR #2 OR #3 OR #4 (4128)

4 TS=(attachment NEAR/2 (interven* or insecure* or secure or security or early or theory or theories)) (1090)

3 TS=(attachment NEAR/2 (disorienta* or reactive or anxious* or disturb* or relation*)) (593)

2 TS=(attachment NEAR/2 (behavior$r* or ambivalen* or avoidant or diffuse or organi* or disorgani* or disrupt* or abnormal* or disinhib* or inhib*)) (2203)

1 TS=(attachment NEAR/2 (disorder$ or problem$ or style$ or pattern$)) (924)

© Queen's Printer and Controller of HMSO 2015. This work was produced by Wright *et al.* under the terms of a commissioning contract issued by the Secretary of State for Health. This issue may be freely reproduced for the purposes of private research and study and extracts (or indeed, the full report) may be included in professional journals provided that suitable acknowledgement is made and the reproduction is not associated with any form of advertising. Applications for commercial reproduction should be addressed to: NIHR Journals Library, National Institute for Health Research, Evaluation, Trials and Studies Coordinating Centre, Alpha House, University of Southampton Science Park, Southampton SO16 7NS, UK.

Social Science Citation Index (SSCI; via ISI Web of Science)

Date searched: 1956 to 6 January 2012.

Date of search: 10 January 2012

A total of 80 records were retrieved.

Databases=SSCI-EXPANDED Timespan=All Years.

Lemmatization=Off.

Search strategy

20 #18 NOT #19 (80)

19 TS=(rat or rats or mouse or mice or murine or hamster or hamsters or animal or animals or dogs or dog or pig or pigs or cats or bovine or cow or sheep or ovine or porcine or monkey) (91,781)

18 #7 AND #17 (80)

17 #8 OR #9 OR #10 OR #11 OR #12 OR #13 OR #14 OR #15 OR #16 (682,942)

16 TS=(foster* or adopt*) (75,821)

15 TS=(neglect* or abuse or abused or abusive or maltreat* or mistreat*) (91,084)

14 TS=(attunement or (representation* NEAR/2 model*)) (2062)

13 TS=dyad* (10,250)

12 TS=(parent* or mother* or maternal* or mum* or father* or paternal* or dad*) (160,559)

11 TS=("young people" or "young person" or "young persons" or "young adult*" or "early adult*") (35,541)

10 TS=(schoolchild* or adolescen* or juvenile* or youth* or teenage* or youngster*) (165,662)

9 TS=(boy or boys or girl or girls) (42,755)

8 TS=(child* or infant* or infancy or preschool* or "pre school*" or baby or babies or pediat* or paediat*) (392,420)

7 #5 and #6 (128)

6 TS=(econom* or cost or costs or costly or costing or price or prices or pricing or pharmacoeconomic*) (499,554)

5 #1 OR #2 OR #3 OR #4 (6395)

4 TS=(attachment NEAR/2 (interven* or insecure* or secure or security or early or theory or theories)) (3,717)

3 TS=(attachment NEAR/2 (disorienta* or reactive or anxious* or disturb* or relation*)) (2147)

2 TS=(attachment NEAR/2 (behavior$r* or ambivalen* or avoidant or diffuse or organi* or disorgani* or disrupt* or abnormal* or disinhib* or inhib*)) (1017)

1 TS=(attachment NEAR/2 (disorder$ or problem$ or style$ or pattern$)) (2646)

Conference Proceedings Citation Index – Science (CPCI-S; via ISI Web of Science)

Date searched: 1990 to 6 January 2012.

Date of search: 10 January 2012.

One record was retrieved.

Databases=CPCI-S Timespan=All Years.

Lemmatization=Off.

Search strategy

20 #18 NOT #19 (1)

19 TS=(rat or rats or mouse or mice or murine or hamster or hamsters or animal or animals or dogs or dog or pig or pigs or cats or bovine or cow or sheep or ovine or porcine or monkey) (282,876)

18 #7 AND #17 (1)

17 #8 OR #9 OR #10 OR #11 OR #12 OR #13 OR #14 OR #15 OR #16 (291,238)

16 TS=(foster* or adopt*) (87,891)

15 TS=(neglect* or abuse or abused or abusive or maltreat* or mistreat*) (17,504)

14 TS=(attunement or (representation* NEAR/2 model*)) (3792)

13 TS=dyad* (1817)

12 TS=(parent* or mother* or maternal* or mum* or father* or paternal* or dad*) (70,471)

11 TS=("young people" or "young person" or "young persons" or "young adult*" or "early adult*") (5160)

10 TS=(schoolchild* or adolescen* or juvenile* or youth* or teenage* or youngster*) (24,476)

9 TS=(boy or boys or girl or girls) (5857)

8 TS=(child* or infant* or infancy or preschool* or "pre school*" or baby or babies or pediat* or paediat*) (104,538)

7 #5 and #6 (9)

© Queen's Printer and Controller of HMSO 2015. This work was produced by Wright *et al.* under the terms of a commissioning contract issued by the Secretary of State for Health. This issue may be freely reproduced for the purposes of private research and study and extracts (or indeed, the full report) may be included in professional journals provided that suitable acknowledgement is made and the reproduction is not associated with any form of advertising. Applications for commercial reproduction should be addressed to: NIHR Journals Library, National Institute for Health Research, Evaluation, Trials and Studies Coordinating Centre, Alpha House, University of Southampton Science Park, Southampton SO16 7NS, UK.

6 TS=(econom* or cost or costs or costly or costing or price or prices or pricing or pharmacoeconomic*) (290,497)

Databases=CPCI-S Timespan=All Years.

5 #1 OR #2 OR #3 OR #4 (344)

4 TS=(attachment NEAR/2 (interven* or insecure* or secure or security or early or theory or theories)) (94)

3 TS=(attachment NEAR/2 (disorienta* or reactive or anxious* or disturb* or relation*)) (54)

2 TS=(attachment NEAR/2 (behavior$r* or ambivalen* or avoidant or diffuse or organi* or disorgani* or disrupt* or abnormal* or disinhib* or inhib*)) (141)

1 TS=(attachment NEAR/2 (disorder$ or problem$ or style$ or pattern$)) (95)

Conference Proceedings Citation Index – Social Science & Humanities (CPCI-SSH; via ISI Web of Science)

Date searched: 1990 to 6 January 2012.

Date of search: 10 January 2012.

A total of eight records were retrieved.

Databases=CPCI-SSH Timespan=All Years.

Lemmatization=Off.

Search strategy

20 #18 NOT #19 (8)

19 TS=(rat or rats or mouse or mice or murine or hamster or hamsters or animal or animals or dogs or dog or pig or pigs or cats or bovine or cow or sheep or ovine or porcine or monkey) (4408)

18 #7 AND #17 (8)

17 #8 OR #9 OR #10 OR #11 OR #12 OR #13 OR #14 OR #15 OR #16 (45,707)

16 TS=(foster* or adopt*) (13,580)

15 TS=(neglect* or abuse or abused or abusive or maltreat* or mistreat*) (5468)

14 TS=(attunement or (representation* NEAR/2 model*)) (356)

13 TS=dyad* (832)

12 TS=(parent* or mother* or maternal* or mum* or father* or paternal* or dad*) (8462)

11 TS=("young people" or "young person" or "young persons" or "young adult*" or "early adult*") (1874)

10 TS=(schoolchild* or adolescen* or juvenile* or youth* or teenage* or youngster*) (7388)

9 TS=(boy or boys or girl or girls) (2090)

8 TS=(child* or infant* or infancy or preschool* or "pre school*" or baby or babies or pediat* or paediat*) (19,667)

7 #5 and #6 (14)

6 TS=(econom* or cost or costs or costly or costing or price or prices or pricing or pharmacoeconomic*) (65,628)

5 #1 OR #2 OR #3 OR #4 (425)

4 TS=(attachment NEAR/2 (interven* or insecure* or secure or security or early or theory or theories)) (254)

3 TS=(attachment NEAR/2 (disorienta* or reactive or anxious* or disturb* or relation*)) (170)

2 TS=(attachment NEAR/2 (behavior$r* or ambivalen* or avoidant or diffuse or organi* or disorgani* or disrupt* or abnormal* or disinhib* or inhib*)) (83)

1 TS=(attachment NEAR/2 (disorder$ or problem$ or style$ or pattern$)) (143)

Education Resources Information Center (ERIC; via ProQuest)

Date searched: 1966 to December 2011.

Date of search: 11 January 2012.

A total of 72 records were retrieved.

Search strategy

S1 ((su(("Attachment Behavior")) OR TI,AB(attachment NEAR/2 (disorder[*1] OR problem[*1] OR style[*1] OR pattern[*1]))) OR TI,AB(attachment NEAR/2 (behavio*r* OR ambivalen* OR avoidant OR diffuse OR organi* OR disorgani* OR disrupt* OR abnormal* OR disinhib* OR inhib*)) OR TI,AB(attachment NEAR/2 (disorienta* OR reactive OR anxious* OR disturb* OR relation* OR interven* OR insecure* OR secure OR security OR early OR theory OR theories))) 2909*

S2 SU("Cost Effectiveness" or "Economic Research" or "Health Care Costs") OR TI,AB(econom* or cost or costs or costly or costing or price or prices or pricing or pharmacoeconomic*) 113,357*

S3 S1 and S2 72*

© Queen's Printer and Controller of HMSO 2015. This work was produced by Wright *et al.* under the terms of a commissioning contract issued by the Secretary of State for Health. This issue may be freely reproduced for the purposes of private research and study and extracts (or indeed, the full report) may be included in professional journals provided that suitable acknowledgement is made and the reproduction is not associated with any form of advertising. Applications for commercial reproduction should be addressed to: NIHR Journals Library, National Institute for Health Research, Evaluation, Trials and Studies Coordinating Centre, Alpha House, University of Southampton Science Park, Southampton SO16 7NS, UK.

Social Services Abstracts (via CSA Illumina)

Date searched: 1979 to December 2011.

Date of search: 11 January 2012.

A total of 19 records were retrieved.

Search strategy

(KW=((attachment within 2 (disorder* or problem* or style* or pattern*)) or (attachment within 2 (behavior* or behavior* or ambivalen* or avoidant or diffuse or organi* or disorgani* or disrupt* or abnormal* or disinhib* or inhib*)) or (attachment within 2 (disorienta* or reactive or anxious* or disturb* or relation*))) or KW=(attachment within 2 (interven* or insecure* or secure or security or early or theory or theories))) and((DE=("adolescents" or "children" or "infants")) or(KW=((child* or infant* or infancy or preschool* or "pre school*" or baby or babies or pediat* or paediat*) or (boy or boys or girl or girls) or (schoolchild* or adolescen* or juvenile* or youth* or teenage* or youngster*)) or KW=("young people" or "young person" or "young persons" or "young adult*" or "early adult*")) or(DE=(Dyads or Child Neglect or Child Abuse or Foster Care or Foster Children or Adoption or Adopted Children) or KW= ((parent* or mother* or maternal* or mum* or father* or paternal* or dad*) or (dyad* or attunement) or (representation* within 2 model*)) or KW=((neglect* or abuse or abused or abusive or maltreat* or mistreat*) or (foster* or adopt*)))) and(DE=((Health Care Costs) or (Cost-Benefit Analysis) or (Cost Containment)) or KW=(econom* or cost or costs or costly or costing or price or prices or pricing or pharmacoeconomic*))

Applied Social Sciences Index and Abstracts (ASSIA; via CSA Illumina)

Date searched: 1987 to December 2011.

Date of search: 11 January 2012.

A total of 27 records were retrieved.

Search strategy

(((KW=((attachment within 2 (disorder* or problem* or style* or pattern*)) or (attachment within 2 (behavior* or behavior* or ambivalen* or avoidant or diffuse or organi* or disorgani* or disrupt* or abnormal* or disinhib* or inhib*)) or (attachment within 2 (disorienta* or reactive or anxious* or disturb* or relation*))) or KW=(attachment within 2 (interven* or insecure* or secure or security or early or theory or theories))) or(DE="attachment disorders")) and(DE=((Children or Infants or Adolescents) or (Parents or Dyads) or (Child neglect or Child abuse or Foster Care or Foster children or Adoption or Adopted children or Adoptive parents)) or KW=((child* or infant* or infancy or preschool* or "pre school*" or baby or babies or pediat* or paediat*) or (boy or boys or girl or girls) or (schoolchild* or adolescen* or juvenile* or youth* or teenage* or youngster* or "young people" or "young person" or "young persons" or "young adult*" or "early adult*")) or KW=((parent* or mother* or maternal* or mum* or father* or paternal* or dad* or dyad* or attunement) or (representation* within 2 model*) or (neglect* or abuse or abused or abusive or maltreat* or mistreat* or foster* or adopt*)))) and(DE=(Economic analysis or Cost benefit analysis or Fiscal impact analysis or Cost effectiveness or Cost effective analysis or Cost containment or Health costs) or KW=(econom* or cost or costs or costly or costing or price or prices or pricing or pharmacoeconomic*))

Social Care Online (via SCIE)

Date searched: inception to 2012.

Date of search: 12 January 2012.

A total of 27 records were retrieved.

Advanced search option

(topic"attachment" or freetext="attachment") AND (topic="children" or topic="babies" or topic="young people" or topic="child abuse" or topic="child neglect" or topic="adoption" or topic="adoptive parents" or topic="adoptive children" or topic="foster care" or topic="foster children" or freetext="child*" or freetext="infant*" or freetext="infancy" or freetext="preschool*" or freetext="pre school*" or freetext="baby" or freetext="babies" or freetext="pediat*" or freetext="paediat*" or freetext="juvenile*" or freetext="youth*" or freetext="teenage*" or freetext="youngster*" or freetext="young people" or freetext="young person" or freetext="young persons" or freetext="young adult*" or freetext="early adult") AND (topic="cost effectiveness" or freetext="economic*" or freetext="cost" or freetext="costs" or freetext="costly" or freetext="costing" or freetext="price" or freetext="prices" or freetext="pricing" or freetext="pharmacoeconomic*")

NHS Economic Evaluation Database (via The Cochrane Library)

Issue 4, 2011.

Date searched: inception to 2012.

Date of search: 12 January 2012.

One record was retrieved.

Search strategy

#1 MeSH descriptor Reactive Attachment Disorder explode all trees 9

#2 (attachment NEAR/3 (disorder* or problem* or style* or pattern*)):ti,ab,kw 59

#3 (attachment NEAR/3 (behavio?r* or ambivalen* or avoidant or diffuse or organi* or disorgani* or disrupt* or abnormal* or disinhib* or inhib*)):ti,ab,kw 31

#4 (attachment NEAR/3 (disorienta* or reactive or anxious* or disturb* or relation*)):ti,ab,kw 108

#5 (attachment NEAR/3 (interven* or insecure* or secure or security or early or theory or theories)):ti,ab, kw 81

#6 (#1 OR #2 OR #3 OR #4 OR #5) 199

[Line #6 includes the results from all The Cochrane Library databases: CDSR 3, DARE 1, CENTRAL 193, HTA 1, and NHS EED 1.]

© Queen's Printer and Controller of HMSO 2015. This work was produced by Wright *et al.* under the terms of a commissioning contract issued by the Secretary of State for Health. This issue may be freely reproduced for the purposes of private research and study and extracts (or indeed, the full report) may be included in professional journals provided that suitable acknowledgement is made and the reproduction is not associated with any form of advertising. Applications for commercial reproduction should be addressed to: NIHR Journals Library, National Institute for Health Research, Evaluation, Trials and Studies Coordinating Centre, Alpha House, University of Southampton Science Park, Southampton SO16 7NS, UK.

Health Economic Evaluations Database (via Wiley InterScience)

Date searched: inception to 2012.

Date of search: 12 January 2012.

No records were retrieved.

Search strategy

AX='attachment disorder' within 3 or 'attachment disorders' within 3 or 'attachment problem' within 3 or 'attachment problems' within 3 or 'attachment style' within 3 or 'attachment styles' within 3 or 'attachment pattern' within 3 or 'attachment patterns' within 3 (0)

AX='attachment behaviour' within 3 or 'attachment behaviour' within 3 or 'attachment avoidant' within 3 or 'attachment diffuse' within 3 or 'attachment organised' within 3 or 'attachment organized' within 3 or 'attachment disorganised' within 3 or 'attachment disorganized' within 3 (0)

AX='attachment disruption' within 3 or 'attachment abnormal' within 3 or 'attachment disinhibited' within 3 or 'attachment inhibited' within 3 or 'attachment disoriented' within 3 or 'attachment reactive' within 3 or 'attachment anxious' within 3 or 'attachment disturbed' within 3 (0)

AX='attachment relationship' within 3 or 'attachment intervention' within 3 or 'attachment insecure' within 3 or 'attachment secure' within 3 or 'attachment security' within 3 or 'attachment early' within 3 or 'attachment theory' within 3 or 'attachment theories' within 3 (0)

CS=1 or 2 or 3 or 4 (0)

Generic searches

Cochrane Database of Systematic Reviews, Database of Abstracts of Reviews of Effects and Health Technology Assessment (via The Cochrane Library)

Issue 12/4, 2011.

Date searched: inception to 2012.

Date of search: 12 Januray 2012.

Three records were retrieved in CDSR, one in DARE, and one in HTA.

Search strategy

#1 MeSH descriptor Reactive Attachment Disorder explode all trees 9

#2 (attachment NEAR/3 (disorder* or problem* or style* or pattern*)):ti,ab,kw 59

#3 (attachment NEAR/3 (behavio?r* or ambivalen* or avoidant or diffuse or organi* or disorgani* or disrupt* or abnormal* or disinhib* or inhib*)):ti,ab,kw 31

#4 (attachment NEAR/3 (disorienta* or reactive or anxious* or disturb* or relation*)):ti,ab,kw 108

#5 (attachment NEAR/3 (interven* or insecure* or secure or security or early or theory or theories)):ti,ab, kw 81

#6 (#1 OR #2 OR #3 OR #4 OR #5) 199

[Line #6 includes the results from all The Cochrane Library databases: CDSR 3, DARE 1, CENTRAL 193, HTA 1, and NHS EED 1.]

Campbell Library (Campbell Collaboration)

Date searched: inception to 2012.

Date of search: 12 January 2012.

Eight records were retrieved.

Search strategy

1. attachment disorder* in all text or attachment problem* in all text or attachment style* in all text or attachment pattern* in all text (6)

2. attachment behavior* in all text or attachment behaviour* in all text or attachment ambivalen* in all text or attachment avoidant in all text or attachment diffuse in all text or attachment organi* in all text or attachment disorgani* in all text or attachment disrupt* in all text or attachment abnormal* in all text or attachment disinhib* in all text or attachment inhib* in all text (3)

3. attachment disorienta* in all text or attachment reactive in all text or attachment anxious* in all text or attachment disturb* in all text or attachment relation* in all text (1)

4. attachment interven* in all text or attachment insecure* in all text or attachment secure in all text or attachment security in all text or attachment early in all text or attachment theory in all text or attachment theories in all text (1)

5. 1 OR 2 OR 3 OR 4 8

Research Register for Social Care

URL: www.researchregister.org.uk/

Date searched: inception to 2012.

Date of search: 12 January 2012.

Six records were retrieved.

Search strategy

attachment

Index to Theses

URL: www.theses.com/

Date searched: inception to 2012.

Date of search: 12 January 2012.

A total of 24 records were retrieved.

Standard search.

© Queen's Printer and Controller of HMSO 2015. This work was produced by Wright *et al.* under the terms of a commissioning contract issued by the Secretary of State for Health. This issue may be freely reproduced for the purposes of private research and study and extracts (or indeed, the full report) may be included in professional journals provided that suitable acknowledgement is made and the reproduction is not associated with any form of advertising. Applications for commercial reproduction should be addressed to: NIHR Journals Library, National Institute for Health Research, Evaluation, Trials and Studies Coordinating Centre, Alpha House, University of Southampton Science Park, Southampton SO16 7NS, UK.

Any field

"attachment disorder"

"attachment disorders"

"attachment problem"

"attachment problems"

"attachment behaviour"

"attachment behaviours"

"attachment behavior"

OAIster

URL: http://oaister.worldcat.org/

Date searched: inception to 2012.

Date of search: 12 January 2012.

A total of 68 records were retrieved.

Search strategy

kw:"attachment disorder" OR "attachment disorders"

OpenGrey

URL: www.opengrey.eu/

Date searched: inception to 2012.

Date of search: 12 January 2012.

A total of 31 records were retrieved.

Search strategy

attachment NEAR/2 disorder* OR attachment NEAR/2 problem* OR attachment NEAR/2 style* OR attachment NEAR/2 pattern* OR attachment NEAR/2 behaviour* OR attachment NEAR/2 behavior* OR attachment NEAR/2 avoidant OR attachment NEAR/2 diffuse OR attachment NEAR/2 organi* OR attachment NEAR/2 disorgani* OR attachment NEAR/2 disrupt* OR attachment NEAR/2 abnormal* OR attachment NEAR/2 disinhib* OR attachment NEAR/2 inhib* OR attachment NEAR/2 disorienta* OR attachment NEAR/2 insecure OR attachment NEAR/2 secure OR attachment NEAR/2 reactive OR attachment NEAR/2 theor* OR attachment NEAR/2 anxious* OR attachment NEAR/2 disturb* OR attachment NEAR/2 relation*

Zetoc

URL: http://zetoc.mimas.ac.uk/

Date searched: inception to 2012.

Date of search: 12 January 2012.

A total of 610 records were retrieved.

Each line searched separately.

General search

All fields

"attachment disorder*"

"attachment problem*"

"attachment theor*" child*

"attachment theor*" parent*

"attachment theor*" infan*

"attachment theor*" adoles*

"attachment theor*" adopt*

"attachment theor*" foster*

"attachment behaviour*"

"attachment reactive*"

"attachment interven*"

"attachment insecure*"

"attachment secure"

ClinicalTrials.gov

URL: http://clinicaltrials.gov/

Date searched: inception to 2012.

Date of search: 12 January 2012.

Two records were retrieved.

Search strategy

"attachment disorder" OR "attachment disorders"

© Queen's Printer and Controller of HMSO 2015. This work was produced by Wright *et al.* under the terms of a commissioning contract issued by the Secretary of State for Health. This issue may be freely reproduced for the purposes of private research and study and extracts (or indeed, the full report) may be included in professional journals provided that suitable acknowledgement is made and the reproduction is not associated with any form of advertising. Applications for commercial reproduction should be addressed to: NIHR Journals Library, National Institute for Health Research, Evaluation, Trials and Studies Coordinating Centre, Alpha House, University of Southampton Science Park, Southampton SO16 7NS, UK.

metaRegister of Controlled Trials (mRCT)

URL: www.controlled-trials.com/mrct/

Date searched: inception to 2012.

Date of search: 12 January 2012.

Three records were retrieved.

Search strategy

"attachment disorder" OR "attachment disorders"

World Health Organization International Clinical Trials Registry Platform (ICTRP)

URL: www.who.int/ictrp

Date searched: inception to 2012.

Date of search: 12 January 2012.

A total of 13 records were retrieved.

Search strategy

Condition: attachment disorder OR attachment disorders

UK Clinical Research Network Study Portfolio

URL: http://public.ukcrn.org.uk/

Date searched: inception to 2012.

Date of search: 12 January 2012.

Nine records were retrieved.

Search strategy

Title/acronym: attachment

Research Summary: attachment

HSRProj (Health Services Research Projects in Progress)

URL: www.cf.nlm.nih.gov/hsr_project/home_proj.cfm

Date searched: inception to 2012.

Date of search: 12 January 2012.

Ten records were retrieved.

Search strategy

attachment

Internet sites searched

Organisation websites were browsed (publications and/or research) and searched for publications relating to attachment disorder.

Searches were undertaken on 18 January 2012.

APA: www.psych.org/.

Association Child and Adolescent Mental Health: www.acamh.org.uk/.

Mental Health Foundation: www.mentalhealth.org.uk/.

MIND: www.mind.org.uk/.

Royal College of Psychiatrists: www.rcpsych.ac.uk/.

National Collaborating Centre for Mental Health (NCCMH): www.nccmh.org.uk/.

National Institute of Mental Health (NIMH): www.nimh.nih.gov/index.shtml.

Institute for Attachment & Child Development: www.instituteforattachment.org/.

Association for Treatment and Training in the Attachment of Children: www.attach.org/theorational.htm.

Young Minds: www.youngminds.org.uk/.

British Association for Adoption and Fostering: www.baaf.org.uk/.

© Queen's Printer and Controller of HMSO 2015. This work was produced by Wright *et al.* under the terms of a commissioning contract issued by the Secretary of State for Health. This issue may be freely reproduced for the purposes of private research and study and extracts (or indeed, the full report) may be included in professional journals provided that suitable acknowledgement is made and the reproduction is not associated with any form of advertising. Applications for commercial reproduction should be addressed to: NIHR Journals Library, National Institute for Health Research, Evaluation, Trials and Studies Coordinating Centre, Alpha House, University of Southampton Science Park, Southampton SO16 7NS, UK.

Appendix 2 Stakeholder involvement

Stakeholder and advisory group members

Amanda Boorman	Service user
Amy Darwin	Advanced practitioner, Looked After and Adopted Children's Health team (LAACH)
Carol Myers	Service user
Danya Glaser	Consultant Child and Adolescent Psychiatrist
Elizabeth Edginton	Research and Development Lead, Northern School of Child & Adolescent Psychotherapy
Geraldine Casswell	CAMHS consultant psychologist lead for adoption services
Helen Minnis	Clinical Senior Lecturer in Child and Adolescent Psychiatry, University of Glasgow
Marie Hawes	Service user
Mary McKelvy	Service user
Sarah Bryan	Attachment therapist
Sharon McNeil	Service user
Tony Myers	Service user
Vivian Prior	Senior research associate

© Queen's Printer and Controller of HMSO 2015. This work was produced by Wright *et al.* under the terms of a commissioning contract issued by the Secretary of State for Health. This issue may be freely reproduced for the purposes of private research and study and extracts (or indeed, the full report) may be included in professional journals provided that suitable acknowledgement is made and the reproduction is not associated with any form of advertising. Applications for commercial reproduction should be addressed to: NIHR Journals Library, National Institute for Health Research, Evaluation, Trials and Studies Coordinating Centre, Alpha House, University of Southampton Science Park, Southampton SO16 7NS, UK.

Appendix 3 Quality assessment tool for cohort studies

(NB: in most instances exposed means those with a disorganised 'D' classification/or RAD/DAD diagnosis; unexposed means those with an organised classification or non-RAD/DAD population.)

Was the cohort drawn from the same community/source?

High bias: drawn from different samples/sources; selected group; no description of the derivation. Low bias: drawn from the same community/source. Unclear bias: not enough information to permit judgement.

Are the groups assembled/recruited at the same age (i.e. the measurement period)?

High bias: different recruitment period. Low bias: same recruitment period. Unclear bias: not enough information to permit judgement.

Ascertainment of exposure: was the same measurement of attachment organisation/disorders used across the sample?

High bias: different validated measures used to classify attachment organisation/presence of RAD/DAD. Low bias: same validated measurement used for all of the sample. Unclear bias: insufficient information to permit judgement.

Were the coders of the exposure blind to risk factors/predictive variables related to the exposure status?

High bias: coders not blinded to risk factors or predictor variables. Low bias: coders blind to risk factors and predictor variables. Unclear bias: insufficient information to warrant a decision.

Is there demonstration that outcome(s) of interest are not present at start of the study?

High bias: outcomes of interest are present at the start of the study. Low bias: demonstration that outcome(s) of interest are not present at start of the study. Unclear bias: insufficient information to warrant judgement.

Is there a description of attachment classification across the entire sample at baseline?

High bias: attachment patterns/diagnosis of RAD/DAD was not reported for the full original sample. Low bias: attachment pattern/diagnosis of RAD/DAD was reported for full original sample. Unclear bias: insufficient information to make a judgement.

Were subsequent measures rated by blind coders who were not aware of the exposed/unexposed status?

High bias: coders not blinded to exposed/non-exposed group. Low bias: coders blind to status. Unclear bias: insufficient information to warrant a decision.

Were there any significant differences at baseline between those lost at follow-up?

High bias: significant ($p < 0.05$) baseline differences between groups. Low bias: no significant differences between groups. Unclear bias: insufficient information reported.

© Queen's Printer and Controller of HMSO 2015. This work was produced by Wright *et al.* under the terms of a commissioning contract issued by the Secretary of State for Health. This issue may be freely reproduced for the purposes of private research and study and extracts (or indeed, the full report) may be included in professional journals provided that suitable acknowledgement is made and the reproduction is not associated with any form of advertising. Applications for commercial reproduction should be addressed to: NIHR Journals Library, National Institute for Health Research, Evaluation, Trials and Studies Coordinating Centre, Alpha House, University of Southampton Science Park, Southampton SO16 7NS, UK.

If significant differences at baseline are found did they do any analysis to compensate?

High bias: no analysis to compensate. Low bias: statistical analysis to compensate. Unclear bias: insufficient information reported.

Adequacy of follow-up: were the dropout rates/attrition adequately reported?

High bias: more than 20% attrition rate and no description of those lost to follow-up, or no statement. Low bias: complete follow-up (all data accounted for); subjects lost unlikely to contribute to bias (< 20% follow-up, or a description of those who were lost provided). Unclear bias: insufficient information to permit a judgement.

Were dropout rates and reasons for dropout similar across the exposed/unexposed?

High bias: dissimilar dropout rates or reasons across for exposed/unexposed. Low bias: similar rates/reasons for attrition. Unclear bias: insufficient information to permit judgement.

Did the study declare conflicts of interest or identification of funding resources?

High bias: declared conflict of interest or funding sources that may cause bias. Low bias: no conflicts of interest declared or funding resources that may cause bias. Unclear: insufficient information to warrant a decision.

Any other bias?

High bias: there is at least one source of important bias not covered by above criteria. Low bias: no other sources of bias. Unclear bias: insufficient information to permit judgement of whether an important risk of bias exists; or insufficient rationale or evidence that an identified problem will introduce bias.

Appendix 4 Additional searches for 5- to 10-year outcomes for children with disorganised attachment at baseline

Although it was not part of the main or supplementary reviews plan, we carried out a limited scoping review of 5- to 10-year outcomes including attachment outcome measures and any mental health, psychological, cognitive, social or developmental outcomes (this was not a systematic review but is included here for information). These are presented in two tables below (*Table 29* and *30*).

TABLE 29 Summary of findings of attachment stability (or other attachment-related outcomes) between 5 and 10 years

Author, year	Age assessed by SSP	Name of outcome and measurement tool	Age at follow-up (years)	Summary of findings
Gini *et al*. 2007[267]	12–16 months	Mother-child affective negotiation and communication<br><br>Joint Story-telling Task[268]	7.5	Multinomial logistic regression model was employed. Wald chi-squared results showed that infants classified as disorganised were significantly more likely to be classified as overwhelming in middle childhood than mutual-balanced<br><br>Wald χ^2 (1, $n = 110$) = 5.32, $p < 0.05$
Wartner *et al*. 1994[171]	12 or 18 months	Attachment patterns at age 6 years<br><br>Play and reunion session[22]	6	Stability of attachment results for disorganised attachment not specified

© Queen's Printer and Controller of HMSO 2015. This work was produced by Wright *et al.* under the terms of a commissioning contract issued by the Secretary of State for Health. This issue may be freely reproduced for the purposes of private research and study and extracts (or indeed, the full report) may be included in professional journals provided that suitable acknowledgement is made and the reproduction is not associated with any form of advertising. Applications for commercial reproduction should be addressed to: NIHR Journals Library, National Institute for Health Research, Evaluation, Trials and Studies Coordinating Centre, Alpha House, University of Southampton Science Park, Southampton SO16 7NS, UK.

TABLE 30 Summary of findings for psychosocial and developmental 5- to 10-year outcomes

Author, year	Name of outcome and measurement tool	Age at follow-up (years)	Summary of findings
Gini *et al.* 2007[267]	Children's behaviour problems/maladjustment<br><br>Child Behaviour Checklist[269]	7.5	ANOVA and chi-squared analyses were conducted to examine relationship between attachment styles and scores on Child Behaviour Checklist<br><br>There were no significant associations<br><br>Overwhelming children are more likely to have externalising problems
Munson *et al.* 2001[270]	Child externalising problems<br><br>Child Behaviour Checklist[269]	4.5, 5.5, 7, 8 and 9	Disorganised children has significantly higher externalising scores at 9 years than secure children but not avoidant children: $t = 2.05$; $p < 0.05$<br><br>Disorganised classification did not significantly predict trajectory of externalising scores $t = 0.05$; $p > 0.10$
Stams *et al.* 2002[271]	Internalising and externalising behaviours<br><br>Child Behaviour Checklist/Teacher Report Form[269]	7	A hieracrchical multiple regression analysis found that disorganised attachment did not significantly predict externalising $\beta = 0.08$ or internalising behaviour $\beta = -0.05$
Ziv *et al.* 2004[272]	Social information processing<br><br>Revised/adapted version of Social Information Processing Interview[273]	6–7	One-way ANCOVAs were conducted to determine whether or not SIPI discriminated between different SSP classifications<br><br>No significant difference found between C and D groups. *F*-value not reported; $p > 0.10$

ANCOVA, analysis of covariance; ANOVA, analysis of variance; SIPI, Social Information Processing Interview.

Appendix 5 List of excluded studies, with reasons

Supplementary systematic review 1 excluded papers with reasons

Reasons	Key
The study does not focus on the development of a screening/assessment tool	1
The study does not compare the screening tool to another gold standard	2
The aim of the screening tool is not focused on the measurement of attachment (child to primary caregiver)	3
The instrument is not a total scale as opposed to individual attachment items	4
The study does not include a sample of parents and or children under the age of 13 years	5
Papers not found	NF
Papers found post cut-off date	PCO

Supplementary systematic review 1 excluded reference list

Number	Reference	Reason
1	Abela JR, Hankin BL, Haigh EA, Adams P, Vinokuroff T, Trayhern L. Interpersonal vulnerability to depression in High-risk children: the role of insecure attachment and reassurance seeking. *J Clin Child Adolesc Psychol* 2005;**34**:182–92	1
2	Abela JR, Zinck S, Kryger S, Zilber I, Hankin BL. Contagious depression: negative attachment cognitions as a moderator of the temporal association between parental depression and child depression. *J Clin Child Adolesc Psychol* 2009;**38**:16–26	1
3	Abrams KY. *Pathways to Disorganization: A Study Concerning Varying Types of Parental Frightened and Frightening Behaviors as Related to Infant Disorganized Attachment*. PhD thesis. Berkeley, CA: University of California, Berkeley; 2001	1
4	Abrams KY, Rifkin A, Hesse E. Examining the role of parental frightened/frightening subtypes in predicting disorganized attachment within a brief observational procedure. *Dev Psychopathol* 2006;**18**:345–61	1
5	Ackerman JP, Dozier M. The influence of foster parent investment on children's representations of self and attachment figures. *J Applied Dev Psychol* 2005;**26**:507–20	1
6	Adams BL. *An Investigation of the Interrelationships among Security of Attachment, Parenting Attitudes, and the Development of Competence.* PhD thesis. Norfolk, VA: Virginia Consortium For Professional Psychology Old Dominion University; 1995	1
7	Ades LAF. *Maternal Employment, Attachment, and Breastfeeding: Pathways to Early Childhood Problem Behaviors*. PhD thesis. Lincoln, NE: University of Nebraska – Lincoln; 2010	1
8	Ahern NR, Ruland JP. Maternal–fetal attachment in African-American and Hispanic-American women. *J Perinat Educ* 2003;**12**:27–35	3
9	Ainsworth MD. Patterns of attachment behavior shown by the infant in interaction with his mother. *Merrill Palmer Q* 1964;**10**:51–8	2
10	Ainsworth MDS, Blehar MC, Waters E, Wall S. *Patterns of Attachment: A Psychological Study of the Strange Situation.* Hillsdale, NJ: Lawrence Erlbaum Associates; 1978	1

© Queen's Printer and Controller of HMSO 2015. This work was produced by Wright *et al.* under the terms of a commissioning contract issued by the Secretary of State for Health. This issue may be freely reproduced for the purposes of private research and study and extracts (or indeed, the full report) may be included in professional journals provided that suitable acknowledgement is made and the reproduction is not associated with any form of advertising. Applications for commercial reproduction should be addressed to: NIHR Journals Library, National Institute for Health Research, Evaluation, Trials and Studies Coordinating Centre, Alpha House, University of Southampton Science Park, Southampton SO16 7NS, UK.

Number	Reference	Reason
11	Guilón-Rivera ÁL. Puerto Rican kindergartners' self-worth as coded from the Attachment Story Completion Task: correlated with other self-evaluation measures and ratings of child behavior toward mothers and peers. *Attach Hum Dev* 2013;**15**:1–23	2
12	Alers V. Treating severely traumatised children and adolescents using sensory integration, attachment theory and clinical reasoning. *J Child Adolesc Ment Health* 2005;**17**:vi–vii	1
13	An J, Zhang J, Wang L. The adolescent attachment inventory. *Chinese Ment Health J* 2004;**18**:760–2	5
14	Anderson GC, Radjenovic D, Chiu S-H, Conlon M, Lane AE. Development of an observational instrument to measure mother-infant separation post birth. *J Nurs Measure* 2004;**12**:215–34	3
15	Andreassen C, Fletcher P. *Early Childhood Longitudinal Study, Birth Cohort (ECLS-B): Psychometric Report for the 2-Year Data Collection. NCES 2007–084.* Washington, DC: US Department of Education, National Center for Education Statistics; 2007	2
16	Andreassen C, West J. Measuring socioemotional functioning in a national birth cohort study. *Infant Ment Health J* 2007;**28**:627–46	2
17	Ang RP. Dysfunctional parenting behaviors and parenting stress among mothers of aggressive boys. *Child Fam Behav Therapy* 2008;**30**:319–36	3
18	Aoki Y, Zeanah CH, Heller SS, Bakshi S. Parent–infant relationship global assessment scale: a study of its predictive validity. *Psychiatry Clin Neurosci* 2002;**56**:493–7	2
19	Arace A. The attachment relationship in early infancy: universal and cultural dimensions. *Eta Evolutiva* 2006;**83**:102–14	1
20	Atwood GC. *Adult Attachment Disorganization: A New Classification and Scoring Scheme for the Adult Attachment Interview*. PhD thesis. Cambridge, MA: Harvard University; 1996	3
21	Aviezer O, Sagi A, Resnick G, Gini M. School competence in young adolescence: links to early attachment relationships beyond concurrent self-perceived competence and representations of relationships. *Int J Behav Dev* 2002;**26**:397–409	3
22	Bacro F. French validation of the child-father and child-mother attachment perceptions security scale (Kerns, Klepac, & Cole, 1996). *Rev Eur Psychol Appl* 2011;**61**:213–21	2
23	Barber R. *The Amae Construct: An Empirical Investigation*. PhD thesis. New York, NY: New School University; 2004	3
24	Barnett D, Butler CM, Vondra JI. Atypical attachment in infancy and early childhood among children at development risk. VIII. Atypical patterns of early attachment: discussion and future directions. *Monogr Soc Res Child Dev* 1999;**64**:172–209	1
25	Barsky S. *Development of a Scale that Measures Attachment Styles of Latency-Aged Children.* PhD thesis. Miami, FL: Carlos Albizu University; 2006	2
26	Bayer JK, Sanson AV, Hemphill SA. Children's moods, fears, and worries: development of an early childhood parent questionnaire. *J Emot Behav Disord* 2006;**14**:41–9	3
27	Becker A. Two Cribs: Bad for Baby? *Psychology Today;* 2003	1
28	Behar LB, Stringfield S. A behavior rating scale for the preschool child. *Dev Psychol* 1974;**10**:601–10	1
29	Behrens K, Kaplan N. Japanese children's family drawings and their link to attachment. *Attach Hum Dev* 2011;**13**:437–50	2
30	Behrens KY, Parker AC, Haltigan JD. Maternal sensitivity assessed during the Strange Situation Procedure predicts child's attachment quality and reunion behaviors. *Infant Behav Dev* 2011;**34**:378–81	3
31	Belden AC, Sullivan J, Luby JL. Depressed and healthy preschoolers' internal representations of their mothers' caregiving: associations with observed caregiving behaviors one year later. *Attach Hum Dev* 2007;**9**:239–54	3
32	Bell M. Bell Object Relations Inventory for adolescents and children: reliability, validity, and factorial invariance. *J Personality Assess* 2003;**8**:19–25	4
33	Belsky J, Rovine M. Q-Sort security and first-year nonmaternal care. *New Directions Child Dev* 1990;**1990**:7–22	1

Number	Reference	Reason
34	Belsky J, Rovine M. Temperament and attachment security in the strange situation: an empirical rapprochement. *Child Dev* 1987;**58**:787–95	1
35	Benoit D, Parker KC, Zeanah CH. Mothers' representations of their infants assessed prenatally: stability and association with infants' attachment classifications. *J Child Psychol Psychiatry* 1997;**38**:307–13	3
36	Beresford C, Robinson JL, Holmberg J, Ross RG. Story stem responses of preschoolers with mood disturbances. *Attach Hum Dev* 2007;**9**:255–70	3
37	Bernstein VJ, Hans SL, Percansky C. Advocating for the young-child in need through strengthening the parent–child relationship. *J Clin Child Psychol* 1991;**20**:28–41	1
38	Bienfait M, Maury M, Haquet A, Faillie J-L, Franc N, Combes C, *et al.* Pertinence of the self-report Mother-to-Infant Bonding Scale in the neonatal unit of a maternity ward. *Early Hum Dev* 2011;**87**:281–7	3
39	Bifulco A, Figueiredo B, Guedeney N, Gorman L, Hayes S, Muzik M, *et al.* Maternal attachment style and depression associated with childbirth: preliminary results from a European and US cross-cultural study. *Br J Psychiatry* 2004;**184**(Suppl. 46):s31–7	3
40	Biringen Z, Brown D, Donaldson L, Green S, Krcmarik S, Lovas G. Adult Attachment Interview: linkages with dimensions of emotional availability for mothers and their pre-kindergarteners. *Attach Hum Dev* 2000;**2**:188–202	3
41	Blokland K. *Maternal Attachment and Response to Infant Affect.* PhD thesis. Toronto, ON: Univeristy of Toronto; 2000	3
42	Bojanowski JJ, Ammen S. Discriminating between pre- versus post-theraplay treatment Marschak Interaction Methods using the Marschak Interaction Method Rating System. *Int J Play Ther* 2011;**20**:1–11	1
43	Boris NW, Zeanah CH, Larrieu JA, Scheeringa MS, Heller SS. Attachment disorders in infancy and early childhood: a preliminary investigation of diagnostic criteria. *Am J Psychiatry* 1998;**155**:295–7	2
44	Bretherton I G-RÁ, Page TF, Oettel BJ, Corey JM, Golby BJ. Children's attachment-related self-worth: a multi-method investigation of postdivorce preschoolers' relationships with their mothers and peers. *Attach Hum Dev* 2013;**15**:25–49	2
45	Britner PA, Marvin RS, Pianta RC. Development and preliminary validation of the caregiving behavior system: association with child attachment classification in the preschool Strange Situation. *Attach Hum Dev* 2005;**7**:83–102	3
46	Britner PAI. *Maternal Caregiving Behavior and Child Attachment Classifications in the Preschool Strange Situation.* PhD thesis. Charlottesville, VA: University of Virginia; 1997	3
47	Brock DM, Sarason IG, Sanghvi H, Gurung RAR. The Perceived Acceptance Scale: development and validation. *J Soc Pers Relatsh* 1998;**15**:5–21	5
48	Brockington I, Fraser C, Wilson D. The Postpartum Bonding Questionnaire: a validation. *Arch Womens Ment Health* 2006;**9**:233–42	3
49	Brockington IF, Oates J, George S, Turner D, Vostanis P, Sullivan M, *et al.* A screening questionnaire for mother–infant bonding disorders. *Arch Womens Ment Health* 2001;**3**:133–40	3
50	Brookings JB, Zembar MJ, Hochstetler GM. An interpersonal circumplex/five-factor analysis of the Rejection Sensitivity Questionnaire. *Pers Indiv Differ* 2003;**34**:449–61	3
51	Brown LS, Wright J. Attachment theory in adolescence and its relevance to developmental psychopathology. *Clin Psychol Psychother* 2001;**8**:15–32	1
52	Bruscato WL, Iacoponi E. Validity and reliability of the Brazilian version of an inventory for the evaluation of object relations. *Revista Brasileira de Psiquiatria* 2000;**22**:172–7	5
53	Buchheim A. Clinical attachment research: measures and results. *Nervenheilkunde* 2007;**26**:291–8	1
54	Buchsbaum HK, Toth SL, Clyman RB, Cicchetti D, Emde RN. The use of a narrative story stem technique with maltreated children: implications for theory and practice. *Dev Psychopathol* 1992;**4**:603–25	1
55	Busch LE. *Development of the Attachment Relationship Questionnaire*. PhD thesis. Bowling Green, OH: Bowling Green State University; 1993	2

© Queen's Printer and Controller of HMSO 2015. This work was produced by Wright *et al.* under the terms of a commissioning contract issued by the Secretary of State for Health. This issue may be freely reproduced for the purposes of private research and study and extracts (or indeed, the full report) may be included in professional journals provided that suitable acknowledgement is made and the reproduction is not associated with any form of advertising. Applications for commercial reproduction should be addressed to: NIHR Journals Library, National Institute for Health Research, Evaluation, Trials and Studies Coordinating Centre, Alpha House, University of Southampton Science Park, Southampton SO16 7NS, UK.

Number	Reference	Reason
56	Busch-Rossnagel NA, Fracasso MP, Vargas M. Reliability and validity of a Q-sort measure of attachment security in Hispanic infants. *Hispanic J Behav Sci* 1994;**16**:240–54	2
57	Cappelletty GG, Brown MM, Shumate SE. Correlates of the Randolph Attachment Disorder Questionnaire (RADQ) in a sample of children in foster placement. *Child Adolesc Soc Work J* 2005;**22**:71–84	2
58	Carlson V, Cicchetti D, Barnett D, Braunwald K. Disorganized/disoriented attachment relationships in maltreated infants. *Dev Psychol* 1989;**25**:525–31	1
59	Carter AS, Briggs-Gowan MJ, Jones SM, Little TD. The Infant-Toddler Social and Emotional Assessment (ITSEA): factor structure, reliability, and validity. *J Abnorm Child Psychol* 2003;**31**:495–514	3
60	Carter AS, Little C, Briggs-Gowan MJ, Kogan N. The infant-toddler social and emotional assessment (ITSEA): comparing parent ratings to laboratory observations of task mastery, emotion regulation, coping behaviors and attachment status. *Infant Ment Health J* 1999;**20**:375–92	3
61	Cassibba R, Coppola G, Bruno S. Preliminary analysis for the validazione of the atttachment Story Completion Task and of the Attachment Q-Sort. *Psicologia Clinica dello Sviluppo* 2003;**7**:407–55	1
62	Cassibba R, van IJzendoorn MH, D'Odorico L. Attachment and play in child care centres: reliability and validity of the attachment Q-sort for mothers and professional caregivers in Italy. *Int J Behav Dev* 2000;**24**:241–55	2
63	Cassidy J, Poehlmann J, Shaver P. An attachment perspective on incarcerated parents and their children. *Attach Hum Dev* 2010;**12**:285–8	1
64	Cassidy J, Shaver PR. *Handbook of Attachment: Theory, Research, and Clinical Applications*. New York, NY: Guilford Publications Inc; 2008	1
65	Cavalieri CE. *Assessing Mothers' Emotion-Centered Engagement in Attachment–Caregiving Relationships.* PhD thesis. Madison, WI: University of Wisconsin–Madison; 2006	3
66	Cawthorpe D. A new empirical method for assessing human attachment relationship quality. *Cyberpsychol Behav* 2000;**3**:969–79	5
67	Caya ML. *Sibling Support and Sibling Attachment: Promoting the Adjustment of Children in High-Conflict Families*. PhD thesis. Boston, MA: University of Massachusetts Boston; 2002	3
68	Cerezo M, Pons-Salvador G, Trenado RM. Mother–infant interaction and children's socio-emotional development with high- and low-risk mothers. *Infant Behav Dev* 2008;**31**:578–89	3
69	Chaimongkol NN, Flick LH. Maternal sensitivity and attachment security in Thailand: cross-cultural validation of western measures. *J Nurs Measure* 2006;**14**:5–17	2
70	Cicchetti DV. On a model for assessing the security of infantile attachment – issues of observer reliability and validity. *Behav Brain Sci* 1984;**7**:149–50	1
71	Clements M, Barnett D. Parenting and attachment among toddlers with congenital anomalies: examining the strange situation and attachment Q-sort. *Infant Ment Health J* 2002;**23**:625–42	3
72	Condon JT, Corkindale CJ, Boyce P. Assessment of postnatal paternal-infant attachment: development of a questionnaire instrument. *J Reprod Infant Psychol* 2008;**26**:195–210	3
73	Condon JT, Corkindale CJ. The assessment of parent-to-infant attachment: development of a self-report questionnaire instrument. *J Reprod Infant Psychol* 1998;**16**:57–76	3
74	Condon JT. The assessment of antenatal emotional attachment: development of a questionnaire instrument. *Br J Med Psychol* 1993;**66**:167–83	5
75	Condon M-C, Spieker S. It's more than a measure: reflections on a university-early head start partnership. *Infants Young Children* 2008;**21**:70–81	1
76	Coppola G, Vaughn BE, Cassibba R, Costantini A. The attachment script representation procedure in an Italian sample: associations with Adult Attachment Interview scales and with maternal sensitivity. *Attach Hum Dev* 2006;**8**:209–19	3
77	Counts JM, Buffington ES, Chang-Rios K, Rasmussen HN, Preacher KJ. The development and validation of the protective factors survey: a self-report measure of protective factors against child maltreatment. *Child Abuse Neglect* 2010;**34**:762–72	3

Number	Reference	Reason
78	Cox B, Enns M, Clara I. The Parental Bonding Instrument: confirmatory evidence for a three-factor model in a psychiatric clinical sample and in the National Comorbidity Survey. *Soc Psychiatry Psychiatr Epidemiol* 2000;**35**:353–7	5
79	Crittenden P, Kozlowska K, Landini A. Assessing attachment in school-age children. *Clin Child Psychol Psychiatry* 2010;**15**:185–208	2
80	Cummings EM, El-Sheikh M. *An Organizational Scheme for the Classification of Attachments on a Continuum of Felt-Security*. Report number ED288653. Washington, DC: Education Resources Information Center; 1986	2
81	Cyr C, Dubois-Comtois K, Moss E. Mother-child conversations and the attachment of children in the pre-school period. *Can J Behav Sci-Rev Can Sci Comport* 2008;**40**:140–52	1
82	Dabrassi F, Imbasciati A, Della Vedova AM. The social support in pregnancy: Italian validation and assessment of the instrument. *Giornale di Psicologia* 2009;**3**:141–51	3
83	Daniel B, Taylor J, Scott J, Derbyshire D, Neilson D. *Recognizing and Helping the Neglected Child: Evidence-Based Practice for Assessment and Intervention*. London: Jessica Kingsley Publishers; 2011	1
84	Dashiff CJ, Weaver M. Development and testing of a scale to measure separation anxiety of parents of adolescents. *J Nurs Measure* 2008;**16**:61–80	1
85	David Oppenheim RNE, Frederick S. Wamboldt. Associations between 3-year-olds' narrative co-constructions with mothers and fathers and their story completions about affective themes. *Early Dev Parenting* 1996;**5**:149–60	2
86	Davidson CE. *The Intergenerational Transmission of Resilience and Risk: Maternal Depression, Maternal Adult Attachment Status, and the Competence of School-Aged Children*. PhD thesis. Medford, MA: Tufts University; 1993	3
87	de Haas MA, Bakermans-Kranenburg MJ, van IJzendoorn MH. The Adult Attachment Interview and questionnaires for attachment style, temperament, and memories of parental behavior. *J Genetic Psychol* 1994;**155**:471–86	3
88	Declercq S, Nicolis H. The attachment theory to help adolescents hospitalized limits. *Neuropsychiatrie de l Enfance et de l Adolescence* 2010;**58**:107–11	5
89	Dekker-van der Sande V, Janssen CGC. Using the AACAP practice parameter for diagnosis of reactive attachment disorder in children with visual and/or ID. *J Intell Disabil Res* 2008;**52**:726	1
90	Diana MS. The Relationship between Observations of Affiliative Behavior Patterns for Parents and Toddlers and Parental Reports of Caregiving, Play, and Support-Control Behaviors. Annual Meeting of the Midwest Association for the Education of Young Children, 17–20 April, 1985, Des Moines, IA, USA	3
91	Dias P, Soares I, Freire T. A scale for assessing the maternal perception of the attachment behavior in 6-years old children. *Psicologia* 2002;**7**:331–43	1
92	Doan HM, Cox NL, Zimerman A. The maternal fetal attachment scale: some methodological ponderings. *J Prenat Perinat Psychol Health* 2003;**18**:167–88	1
93	Doughty C. Effective strategies for promoting attachment between young children and their parents. *NZHTA* 2007;**6**:1–67	1
94	Duffy B, Fell M. Patterns of attachment: further use of the Separation Anxiety Test. *Ir J Psychol* 1999;**20**:159–71	1
95	Dugan AD. *The Relation between Attachment Representations and Emotion Regulation in Preschool-Aged Children with Externalizing Behavior Disorders*. PhD thesis. Newton, MA: Massachusetts School of Professional Psychology; 2007	4
96	Dugravier R. Understanding consequences of hospitalization within attachment theory. *Arch Pediatr* 2010;**17**:723–4	1
97	Dwyer KM. The meaning and measurement of attachment in middle and late childhood. *Hum Dev* 2005;**48**:155–82	1
98	Easterbrooks M, Biringen Z. The Emotional Availability Scales: methodological refinements of the construct and clinical implications related to gender and at-risk interactions. *Infant Ment Health J* 2005;**26**:291–4	1

© Queen's Printer and Controller of HMSO 2015. This work was produced by Wright *et al.* under the terms of a commissioning contract issued by the Secretary of State for Health. This issue may be freely reproduced for the purposes of private research and study and extracts (or indeed, the full report) may be included in professional journals provided that suitable acknowledgement is made and the reproduction is not associated with any form of advertising. Applications for commercial reproduction should be addressed to: NIHR Journals Library, National Institute for Health Research, Evaluation, Trials and Studies Coordinating Centre, Alpha House, University of Southampton Science Park, Southampton SO16 7NS, UK.

Number	Reference	Reason
99	Edelstein RS, Alexander KW, Shaver PR, Schaaf JM, Quas JA, Lovas GS, *et al.* Adult attachment style and parental responsiveness during a stressful event. *Attach Hum Dev* 2004;**6**:31–52	3
100	Edwards J. The use of music therapy to promote attachment between parents and infants. *Arts Psychother* 2011;**38**:190–5	1
101	Egblomasse SC. *The Development of the Mother-Infant-Toddler Attachment Scale (MITAS) (Attachment, Early Intervention)*. PhD thesis. Boston, MA; Boston University; 1999	3
102	Egger HL, Erkanli A, Keeler G, Potts E, Walter B, Angold A. Test-retest reliability of the Preschool Age Psychiatric Assessment (PAPA). *J Am Acad Child Adolesc Psychiatry* 2006;**45**:538–49	1
103	Emde RN, Wolf D, Oppenheim D. *Revealing the Inner Worlds of Young Children: The MacArthur Story Stem Battery and Parent-Child Narratives*. New York, NY: Oxford University Press; 2003	1
104	Erickson ME. *The Relationships among Need Satisfaction, Support, and Maternal Attachment in the Adolescent Mother.* PhD thesis. Austin, TX: Univeristy of Texas at Austin; 1996	3
105	Ewig ML. *The Effect of Child Protective Services Home Removal on the Development of Attachment.* PhD thesis. San Francisco, CA: California School of Professional Psychology – San Francisco Bay Campus; 2006	5
106	Fairchild SR. Attachment representations and parental memories of incarcerated fathers. *Child Adolesc Soc Work J* 2009;**26**:361–77	5
107	Fairchild SR. Understanding attachment: reliability and validity of selected attachment measures for preschoolers and children. *Child Adolesc Soc Work J* 2006;**23**:235–61	1
108	Fairchild-Kienlen SR. *The Clinical Assessment of Attachment Disorder in Children 3–13: An Evaluation of the Attachment Disorder Assessment Scale (ADAS).* PhD thesis. Arlington, TX: Univeristy of Texas at Arlington; 2002	2
109	Fava Vizziello G, Simonelli A, Petena I. Maternal representations and intergenerational transmission of risk and protective factors in children of drug-addicted mothers. *Adicciones* 2000;**12**:413–24	1
110	Feldman S, Ingham ME. Attachment behavior: a validation study in two age groups. *Child Dev* 1975;**46**:319–30	2
111	Feldstein S, Hane A, Morrison B, Huang K. Relation of the Postnatal Attachment Questionnaire to the attachment Q-set. *J Reprod Infant Psychol* 2004;**22**:111–21	3
112	Field T. *Leavetakings and Reunions of Infants, Toddlers, Preschoolers and Their Parents*. Report number ED219161. Washington, DC: Education Resources Information Center; 1978	3
113	Figueiredo B, Marques A, Costa R, Pacheco A, Pais A. Bonding: scale to evaluate parents' emotional involvement with their infant. *Psychologica* 2005;**40**:133–54	3
114	Finzi R, Har-Even D, Weizman A, Tyano S, Shnit D. The adaptation of the Attachment Styles Questionnaire for latency-aged children. *Isr J Psychol* 1996;**5**:167–77	1
115	Floyd L. A model for assisting high-risk families in neonatal nurturing. *Child Welfare* 1981;**60**:637–43	3
116	Follan M, Anderson S, Huline-Dickens S, Lidstone E, Young D, Brown G, *et al.* Discrimination between attention deficit hyperactivity disorder and reactive attachment disorder in school aged children. *Res Dev Disabil* 2011;**32**:520–6	2
117	Foster D, Davies S, Steele H. The evacuation of British children during World War II: a preliminary investigation into the long term psychological effects. *Aging Ment Health* 2003;**7**:398–408	3
118	Fouladi RT, Moller NP, McCarthy CJ. Examination of internal consistency and construct validity of scores on the Parental Attachment Scale: preliminary psychometric results. *Meas Eval Couns Dev* 2006;**39**:2–30	5
119	Francis, DA Attachment disorder: a decline in the performance of the bravo pH system. *Am J Gastroenterol* 2008;**103**:1168–72	3
120	Frankel KA, Boyum LA, Harmen RJ. Diagnoses and presenting symptoms in an infant psychiatry clinic: comparison of two diagnostic systems. *J Am Acad Child Adolesc Psychiatry* 2004;**43**:578–87	NP

Number	Reference	Reason
121	Gaensbauer TJ, Harmon RJ, Culp AM, Schultz LA, van Doorninick WJ, Dawson P. Relationships between attachment behavior in the laboratory and the caretaking environment. *Infant Behav Dev* 1985;**8**:355–69	2
122	Gaensbauer TJ, Harmon RJ, Cytryn L, McKnew DH. Social and affective development in infants with a manic-depressive parent. *Am J Psychiatry* 1984:**141**:223–9	2
123	Gaffney KF. Maternal-fetal attachment in relation to self-concept and anxiety. *Matern Child Nurs J* 1986;**15**:91–101	1
124	Gaffney M, Greene SM, Wieczorek-Deering D, Nugent J. The concordance between mother-infant attachment at 18 months and maternal attachment 10 years later among married and single mothers. *Ir J Psychol* 2000;**21**:154–70	3
125	Gainey RR, Catalano RF, Haggerty KP, Hoppe MJ. Deviance among the children of heroin addicts in treatment: Impact of parents and peers. *Deviant Behav* 1997;**18**:143–59	1
126	Ganiban J, Barnett D, Cicchetti D. Negative reactivity and attachment: Down syndrome's contribution to the attachment-temperament debate. *Dev Psychopathol* 2000;**12**:1–21	1
127	Gao Y, Raine A, Chan F, Venables PH, Mednick SA. Early maternal and paternal bonding, childhood physical abuse and adult psychopathic personality. *Psychol Med* 2010;**40**:1007–16	1
128	Gardner RA. Assessment for the stronger, healthier psychological bond in child-custody evaluations. *J Divorce Remarriage* 1999;**31**:1–14	1
129	Gardner W, Lamb ME, Thompson RA, Sagi A. On individual differences in Strange Situation behavior: categorical and continuous measurements systems in a cross-cultural data set. *Infant Behav Dev* 1986;**9**:355–75	2
130	Garmon LC. *Relations between Attachment Representations and Moral Judgment.* PhD thesis. Columbus, OH: Ohio State University; 2000	5
131	Garwood MM. *Parental Sensitivity, Parenting Beliefs, and Child Temperament: Modeling Effects on Mother– and Father–Infant Attachment*. South Bend, IN: University of Notre Dame; 1998	1
132	Gathwala G, Singh B, Balhara B. KMC facilitates mother baby attachment in low birth weight infants. *Indian J Pediatr* 2008;**75**:43–7	3
133	Gaudet C. Pregnancy after perinatal loss: association of grief, anxiety and attachment. *J Reprod Infant Psychol* 2010;**28**:240–51	5
134	Geddes H. *Attachment in the Classroom. The Links Between Children's Early Experience, Emotional Well-Being and Performance in School.* London: Worth Publishing Limited; 2006	1
135	George M. Can babies get depressed? *Children Now* 2004;20–21	1
136	George MR, Cummings EM, Davies PT. Positive aspects of fathering and mothering, and children's attachment in kindergarten. *Early Child Dev Care* 2010;**180**:107–19	1
137	Germo GR. *Effects of Early Adversity and Current Stressors on Adolescents' and Young Adults' Morning Cortisol Levels: The Moderating Role of Attachment Style.* PhD thesis. Irvine, CA: University of California; 2008	5
138	Gerner L. *Exploring Prenatal Attachment: Factors that Facilitate Paternal Attachment during Pregnancy*. PhD thesis. Fresno, CA: California School of Professional Psychology, Alliant International University; 2005	3
139	Gervai J, Novak A, Lakatos K, Toth I, Danis I, Ronai Z, *et al.* Infant genotype may moderate sensitivity to maternal affective communications: attachment disorganization, quality of care, and the DRD4 polymorphism. *Soc Neurosci* 2007;**2**:307–19	1
140	Geserick B, Spangler G. The influence of early attachment experiences and concurrent maternal support on task-oriented behavior of six-year-olds during cognitive a cognitively challenging situation. *Psychologie Erziehung Unterricht* 2007;**54**:86–102	3
141	Giannini M, Gori A, De Sanctis E, Schuldberg D. Attachment in psychotherapy: psychometric properties of the Psychological Treatment Inventory Attachment Styles Scale (PTI-ASS). *J Psychother Integration* 2011;**21**:363–81	5

© Queen's Printer and Controller of HMSO 2015. This work was produced by Wright *et al.* under the terms of a commissioning contract issued by the Secretary of State for Health. This issue may be freely reproduced for the purposes of private research and study and extracts (or indeed, the full report) may be included in professional journals provided that suitable acknowledgement is made and the reproduction is not associated with any form of advertising. Applications for commercial reproduction should be addressed to: NIHR Journals Library, National Institute for Health Research, Evaluation, Trials and Studies Coordinating Centre, Alpha House, University of Southampton Science Park, Southampton SO16 7NS, UK.

Number	Reference	Reason
142	Gibson LA. *Adult Attachment and Maternal Representations of Gender during Pregnancy: Their Impact on the Child's Subsequent Gender-Role Development*. PhD thesis. New York, NY: City University New York; 1998	1
143	Gil A, Escosteguy NU, Picon P, Zanel AP, Luz C, Litvin E, *et al.* Adrenocortical responses in children to a stress-situation (relation with observation of behavior and interaction with caregivers, considering variables 'temperament and attachment'). *Revista de Psiquiatria do Rio Grande do Sul* 2002;**24**:135–42	1
144	Gilbride KE. *Adolescent Maternal Behavior: Assessment over Time and its Relationship to Infant–Mother Attachment*. PhD thesis. Salt Lake City, UT: University of Utah; 1989	1
145	Glowinski AL. Reactive attachment disorder: an evolving entity. *J Am Acad Child Adolesc Psychiatr* 2011;**50**:210–2	1
146	Goldberg S, Benoit D, Blokland K, Madigan S. Atypical maternal behavior, maternal representations, and infant disorganized attachment. *Dev Psychopathol* 2003;**15**:239–57	3
147	Goldwyn R, Hugh-Jones S. Using the Adult Attachment Interview to understand reactive attachment disorder: findings from a 10-case adolescent sample. *Attach Hum Dev* 2011;**13**:169–91	5
148	Goldwyn R. *What Can the Adult Attachment Interview Contribute to the Assessment of Attachment Disorders and Difficulties in Adolescence? A Pilot Study*. PhD thesis. Leeds: Leeds University; 2005	5
149	Goodfriend MS. Treatment of attachment disorder of infancy in a neonatal intensive care unit. *Pediatrics* 1993;**91**:139–42	1
150	Goodman F, Ford T, Richards H, Gatward R, Meltzer H. The Development and Well-Being Assessment: description and initial validation of an integrated assessment of child and adolescent psychopathology. *J Child Psychol Psychiatry* 2000;**41**:645–56	1
151	Goodman G. Empirical evidence supporting the conceptual relatedness of object representations and internal working models. *J Am Psychoanalytic Assoc* 2005;**53**:597–617	3
152	Goodrich TA. *Healthy Blame.* PhD thesis. New Ypork, NY: Adelphi University; 2002	1
153	Granger CEG. *The Role of the Reflective Function of Foster Carers in the Quality of Long Term Foster Placements: An Exploratory Study.* D.Clin.Psych thesis. Oxford: Oxford University; 2008.	3
154	Green J, Stanley C, Smith V, Goldwyn R. A new method of evaluating attachment representations in the young school-age children: the Manchester Child Attachment Story Task (MCAST). *Attach Hum Dev* 2000;**2**:48–70	2
155	Greenspan SI, Lourie RS. Developmental structuralist approach to the classification of adaptive and pathologic personality organizations: infancy and early childhood. *Am J Psychiatry* 1981;**38**:725–35	1
156	Grienenberger JF. *The Impact of Maternal Reflective Functioning on Mother-Infant Affective Communication: Exploring the Link between Mental States and Observed Caregiving Behavior.* PhD thesis. Wright Institute Los Angeles; 2003	3
157	Grossmann KE, Grossmann K. *Preliminary Observations on Japanese Infants' Behavior in Ainsworth's Strange Situation*. Report number 4. Research & Clinical Center for Child Development Annual Report; 1989	2
158	Grossmann KE, Grossmann K. The development of psychological security in attachment–results and conclusions for therapy. *Z Psychosomatische Med Psych* 2007;**53**:9–28	1
159	Grych JH, Wachsmuth-Schlaefer T, Klockow LL. Interparental aggression and young children's representations of family relationships. *J Fam Psychol* 2002;**16**:259–72	2
160	Guarnieri S, Ponti L, Tani F. The Inventory of Parent and Peer Attachment (IPPA): a study on the validity of styles of adolescent attachment to parents and peers in an Italian sample. *TPM-Testing, Psychometrics, Methodol Appl Psychol* 2010;**17**:103–30	5
161	Guedeney A. Kwashiorkor, depression, and attachment disorders. *Lancet* 1995;**346**:1293	1
162	Guedeney N, Guedeney A, Rabouam C, Mintz AS, Danon G, Huet MM, *et al.* The zero-to-three diagnostic classification: a contribution to the validation of this classification from a sample of 85 under-threes. *Infant Ment Health J* 2003;**24**:313–36	2
163	Guedeney N, Lamas C, Bekhechi V, Mintz AS, Guedeney A. Development of the process of attachment between a baby and its mother. *Arch Pediatr* 2008;**15**(Suppl. 1):12–19	1

Number	Reference	Reason
164	Guile JM. Home part in therapeutic foster care: the contribution of theories of attachment. *Neuropsychiatrie de l Enfance et de l Adolescence* 2003;**51**:450–4	1
165	Gullone E, Robinson K. The Inventory of Parent and Peer Attachment–Revised (IPPA-R) for children: a psychometric investigation. *Clin Psychol Psychother* 2005;**12**:67–79	5
166	Gullon-Rivera AL. *Puerto Rican Kindergarteners' Representation of the Self within Family Relationships: Are they Related to their Self-Worth, Child–Mother Relationship and Behavioral Adjustment?* PhD thesis. Madison, WI: University of Wisconsin – Madison; 2009	3
167	Gunaydin G, Selcuk E, Sumer N, Uysal A. Psychometric evaluation of the Short Form of Inventory of Parent and Peer Attachment. *Turk Psikoloji Yazilari* 2005;**8**:13–23	2
168	Guzder J, Bond S, Rabiau M, Zelkowitz P, Rohar S. The relationship between alliance, attachment and outcome in a child multi-modal treatment population: pilot study. *J Can Acad Child Adolesc Psychiatry* 2011;**20**:196–202	1
169	Hall SEK, Geher G, Brackett MA. *The Measurement of Emotional Intelligence in Children: The Case of Reactive Attachment Disorder.* Hauppaug, Nova Science Publishers; 2004	3
170	Harris JR. Attachment theory underestimates the child. *Behav Brain Sci* 2009;**32**:30	1
171	Hartmann M. Attachment disorder and aurum muriaticum natronatum. *Homoeopathic Links* 2009;**22**:194–6	1
172	Haubrich KA. *The Development of an Instrument to Measure Maternal Pperceptions of the Physical Appearance of Their Infants at Birth.* PhD thesis. Cincinnati, OH: University of Cincinnati; 1996	3
173	Hebebrand J, Mohler E. Children of impaired parents. *Z Kinder Jugendpsychiatr Psychother* 2009;**37**:307–8	1
174	Heider D, Matschinger H, Bernert S, Vilagut G, Martinez-Alonso M, Dietrich S, *et al.* Empirical evidence for an invariant three-factor structure of the Parental Bonding Instrument in six European countries. *Psychiatry Res* 2005;**135**:237–47	3
175	Heiss GE, Berman WH, Sperling MB. Five scales in search of a construct: exploring continued attachment to parents in college students. *J Personality Assess* 1996;**67**:102–15	5
176	Hesse E. The Adult Attachment Interview: Protocol, Method of Analysis, and Empirical Studies. In Cassidy J, Shaver PR, editors. *Handbook of Attachment: Theory, Research, and Clinical Application.* 2nd edn. New York, NY: Guilford Press; 2008. pp. 552–98	1
177	Hexel M. Validation of the German Version of the Attachment Style Questionnaire (ASQ) in participants with and without psychiatric diagnosis. *Zeitschrift Klinische Psychologie Psychotherapie: Forschung und Praxis* 2004;**33**:79–90	1
178	Hock E. Maternal separation anxiety: mother-infant separation from the maternal perspective. *Child Dev* 1989;**60**:793–802	3
179	Hoeksma J, Alliet M, Koomen H. Attachment behaviour observed by fathers and mothers. *Kind en Adolescent* 1998;**19**:93–8	1
180	Honda J. Development of the scale 'Attachment Toward Mother' in middle childhood. *Jpn J Couns Sci* 2002;**35**:246–55	1
181	Honig AS. Research in review: risk factors in infants and young children. *Young Child* 1984;**39**:60–73	1
182	Hornstein JF. *Emotional Health among Young Children with Special Needs and Typical Children: A Psychometric Study of Measures of Attachment, Interaction, Mastery and Social Support using Confirmatory Factor Analysis*. PhD thesis. Cambridge, MA: Harvard University; 1999	4
183	Howard AR. *An Evaluation of the Psychometric Properties of the Beech Brook Attachment Disorder Checklist*. PhD thesis. Fort Worth, TX: Texas Christian University; 2009	2
184	Howard JM. *Early Childhood Coping: Measure Development and Relationships with Attachment Security and Psychological Functioning*. PhD thesis. Evanston, IL: Northwestern University; 2010	3
185	Howe D. Attachment disorders: disinhibited attachment behaviours and secure base distortions with special reference to adopted children. *Attach Hum Dev* 2003;**5**:265–70	1

© Queen's Printer and Controller of HMSO 2015. This work was produced by Wright *et al.* under the terms of a commissioning contract issued by the Secretary of State for Health. This issue may be freely reproduced for the purposes of private research and study and extracts (or indeed, the full report) may be included in professional journals provided that suitable acknowledgement is made and the reproduction is not associated with any form of advertising. Applications for commercial reproduction should be addressed to: NIHR Journals Library, National Institute for Health Research, Evaluation, Trials and Studies Coordinating Centre, Alpha House, University of Southampton Science Park, Southampton SO16 7NS, UK.

Number	Reference	Reason
186	Howes C, Hamilton CE. Children's relationships with caregivers: mothers and child care teachers. *Child Dev* 1992;**63**:859–66	3
187	Howes C, Ritchie S. Attachment organizations in children with difficult life circumstances. *Dev Psychopathol* 1999;**11**:251–68	3
188	Hu CC. *Construction and Validation of Attachment to Parent/Caregiver Inventory (APCI).* PhD thesis. West Lafayette, IN: Purdue University; 2010	3
189	Hubbard FOA, van Ijzendoorn M. Attachment in Social Networks. Maternal Unresponsiveness, Infant Crying. A Critical Replication of the Bell and Ainsworth Study. In Tavecchio LWC, van IJzendoorn M, editors. *Attachment in Social Networks.Contributions to the Bowlby-Ainsworth Attachment Theory*. Amsterdam and New York, NY: Elsevier Science Publishers; 1987. pp. 339–78	3
190	Hubbard FHO, van IJzendoorn MI. Maternal unresponsiveness and infant crying across the first 9 months: a naturalistic study. *Infant Behav Dev* 1991;**14**:299–312	3
191	Hugger LE. *The Emotional Assessment of Infants, Toddlers and Preschoolers.* PhD thesis. Garden City, NY: Adelphi University, Institute Advanced Psychological Studies; 2000	2
192	Hughes DA. *Attachment Focused Family Therapy.* New York, NY: W.W. Norton; 2007	1
193	Hughes DA. Psychological interventions for the spectrum of attachment disorders and intrafamilial trauma. *Attach Hum Dev* 2003;**5**:271–8	1
194	Humber N, Moss E. The relationship of preschool and early school age attachment to mother–child interaction. *Am J Orthopsychiatry* 2005;**75**:128–41	2
195	Huston J, Hoffman J. Children bewildering parents – reactive attachment disorder and oppositional defiant disorder. *California Pharmacist* 2011;**58**:39–43	1
196	Huth-Bocks AC, Levendosky AA, Theran SA, Bogat G. The impact of domestic violence on mothers' prenatal representations of their infants. *Infant Ment Health J* 2004;**25**:79–98	5
197	Jacobson S, Yumoto C. *Attachment Representation in Inner-City African American Adolescents: A Validation of the Child Attachment Interview.* Paper presented at Attachment in Middle Childhood: New Research on Risk and Resilience, Anna Freud Centre, 2009, London, UK	5
198	Jin MK, Jacobvitz D, Hazen N, Jung SH. Maternal sensitivity and infant attachment security in Korea: cross-cultural validation of the Strange Situation. *Attach Hum Dev* 2012;**14**:33–44	3
199	John A, Halliburton AL. Q methodology to assess child-father attachment. *Early Child Dev Care* 2010;**180**:71–85	3
200	Johnson LN, Ketring SA, Abshire C. The revised inventory of parent attachment: measuring attachment in families. *Contemp Fam Ther* 2003;**25**:333–49	5
201	Johnson SC, Dweck CS, Chen FS. Evidence for infants' internal working models of attachment. *Psychological Sci* 2007;**18**:501–2	1
202	Jurgens M, Levy-Rueff M, Goffinet F, Golse B, Beauquier-Macotta B. Psychometric properties of the French version of the Prenatal Attachment Inventory in 112 pregnant women. *L'Encephale: revue de psychiatrie clinique biologique et therapeutique* 2010;**36**:219–25	3
203	Kappenberg ES, Halpern DF. Kinship Center Attachment Questionnaire: Development of a Caregiver-Completed Attachment Measure for Children Younger than 6 Years. *Educ Psychol Meas* 2006;**66**:852–73	2
204	Karantzas GC, Feeney JA, Wilkinson R. Is less more? Confirmatory factor analysis of the Attachment Style Questionnaires. *J Soc Pers Relatsh* 2010;**27**:749–80	5
205	Kashwer CD. *Assessing the Relationship between a Mother and her Unborn Child: The Prenatal Marschak Interaction Method Rating System.* PhD thesis. Fresno, CA: California School of Professional Psychology, Alliant International University; 2004	3
206	Kayoko Y. Assessing attachment representations in early childhood: validation of the attachment doll play. *Jpn J Educ Psychol* 2006;**54**:476–86	1
207	Kelly KM. *A Modification of the AMBIANCE Scale for Use with Four-Month-Old Infants and their Caregivers: Development, Stability, and Predictive Validity*. PhD thesis. New York, NY: City University; 2004	3

Number	Reference	Reason
208	Kennedy JH. Is maternal behavior in the strange situation related to infant attachment? *J Early Child Infant Psychol* 2008;**4**:83–92	4
209	Kerns KA, Abraham MM, Schlegelmilch A, Morgan TA. Mother-child attachment in later middle childhood: assessment approaches and associations with mood and emotion regulation. *Attach Hum Dev* 2007;**9**:33–53	4
210	Kerns KA, Brumariu LE, Seibert A. Multi-method assessment of mother-child attachment: links to parenting and child depressive symptoms in middle childhood. *Attach Hum Dev* 2011;**13**:315–33	NP
211	Kerns KA, Tomich PL, Aspelmeier JE, Contreras JM. Attachment-based assessments of parent–child relationships in middle childhood. *Dev Psychol* 2000;**36**:614–26	4
212	King P. A 'rebirth' brings death. A controversial therapy for children diagnosed with attachment disorder draws intense scrutiny. *Newsweek* 2005;**135**:65	1
213	Kissgen R. Early intervention – use of attachment and childhood development research in education, counseling, treatment and prevention. *Psychologie in Erziehung Und Unterricht* 2003;**50**:325–7	1
214	Kokcu F, Kesebir S, Dereboy F. The relationship attachment style, personality, and temperament in patients with bipolar disorder and their children: a controlled study. *Bipolar Disord* 2010;**12**:32–3	5
215	Kraemer S, Loader P. Passing Through Life: Alexithymia and Attachment Disorders. *Occasional papers- association for child psychology and psychiatry: Psychosomatic Problems in Children: Clinical Research Perspectives.* 1996:17–20	1
216	Kroonenberg P, van Dam M, van IJzendoorn M, Mooijaart A. Dynamics of behaviour in the strange situation: a structural equation approach. *Br J Psychol* 1997;**88**:311–32	1
217	Kroonenberg PM, Basford KE, van Dam M. Classifying infants in the Strange Situation with three–way mixture method of clustering. *Br J Psychol* 1995;**86**:397–418	1
218	Kunster AK, Fegert JM, Ziegenhain U. Assessing parent–child interaction in the preschool years: a pilot study on the psychometric properties of the Toddler CARE-Index. *Clin Child Psycho Psychiatry* 2010;**15**:379–89	3
219	Kusube T. Disinhibited attachment disorder of childhood. *Ryoikibetsu Shokogun Shirizu* 2003;**40**:57–8	1
220	Kusube T. Reactive attachment disorder of infancy or early childhood. *Ryoikibetsu Shokogun Shirizu* 2003;**40**:54–6	1
221	Lacharite C, Ethier L, Piche C. Parental stress in mothers of preschoolers: validity and standards of the Parenting Stress Index in Quebec. *Sante mentale au Quebec* 1992;**17**:183–203	3
222	Lakatos K, Toth I, Ney K, Gervai J, Nemoda Z, Ronai Z, *et al.* Genetic risk for attachment disorganisation in infancy: the role of the D4 dopamine receptor (DRD4). *J Reprod Infant Psychol* 2001;**19**:269	1
223	Lamb ME, Thompson RM, Gardner W, Charnov EL, Estes D. Security of infantile attachment as assessed in the Strange Situation – Its study and biological interpretation. *Behav Brain Sci* 1984;**7**:127–47	1
224	Lamb ME. Attachments, social networks, and developmental contexts. *Hum Dev* 2005;**48**:108–12	1
225	Lauriola M, Panno A, Riccardi C, Taglialatela D. A psychometric comparative study of three self-report scales assessing individual differences in maternal prenatal attachment. *Infanzia e Adolescenza* 2010;**9**:135–50	3
226	Lee KS, Jeong SJ, Shin YJ. A comparative study of reactive attachment disorder with autistic child in social cognition – focused on social referencing and joint attention. *Korean Jf Clin Psychol* 2000;**19**:793–806	1
227	Lee Y, Park KJ, Rah YM. A study on the development of criterion scores for the Attachment Q-set in Korea. *Korean J Child Studies* 1997;**18**:131–48	2
228	Lehman EB, Denham SA, Moser MH, Reeves SL. Soft object and pacifier attachments in young children – the role of security of attachment to the mother. *J Child Psychol Psychiatry Allied Discip* 1992;**33**:1205–15	1
229	Lester SD. *The Development of a Scale for Measuring Primary Bonding Patterns in Middle-Class Black and White Families.* PhD thesis. Vanderbilt University, George Peabody College for Teachers; 1986	3

© Queen's Printer and Controller of HMSO 2015. This work was produced by Wright *et al.* under the terms of a commissioning contract issued by the Secretary of State for Health. This issue may be freely reproduced for the purposes of private research and study and extracts (or indeed, the full report) may be included in professional journals provided that suitable acknowledgement is made and the reproduction is not associated with any form of advertising. Applications for commercial reproduction should be addressed to: NIHR Journals Library, National Institute for Health Research, Evaluation, Trials and Studies Coordinating Centre, Alpha House, University of Southampton Science Park, Southampton SO16 7NS, UK.

Number	Reference	Reason
230	Levitt MJ. Social relations in childhood and adolescence: the convoy model perspective. *Human Development* 2005;**48**:28–47	1
231	Levy TM, Orlans M. Attachment, trauma, and healing: understanding and treating attachment disorder in children and families. *Adolescence* 2000;**35**:821	1
232	Leyendecker B, Lamb ME, Scholmerich A. Studying mother–infant interaction: the effects of context and length of observation in two subcultural groups. *Infant Behav Dev* 1997;**20**:325–37	3
233	Li F, Fu G. A preliminary study on the Adult Attachment Questionnaire (AAQ 3.1). *Chin J Clin Psychol* 2001;**9**:190–2	5
234	Lim K, Corlett L, Thompson L, Law J, Wilson P, Gillberg C, *et al.* Measuring attachment in large populations: a systematic review. *Educ Child Psychol* 2010;**27**:22–32	1
235	Lindberg MA, Thomas SW. The Attachment and Clinical Issues Questionnaire (ACIQ): scale development. *J Genet Psychol* 2011;**172**:329–52	5
236	Livianos-Aldana L, Rojo-Moreno L. On the convergent validity of two Parental Rearing Behaviour Scales: EMBU and PBI. *Acta Psychiatrica Scandinavica* 1999;**100**:263–9	5
237	Lizardi H, Klein DN. Evidence of increased sensitivity using a three-factor version of the Parental Bonding Instrument. *J Nerv Ment Dis* 2002;**190**:619–23	5
238	Lizardi H, Klein DN. Long term stability of parental representations in depressed outpatients utilizing the Parental Bonding Instrument. *J Nerv Ment Dis* 2005;**193**:183–8	3
239	Lounds JJ, Borkowski JG, Whitman TL, Centers for the Prevention of Child Neglect. Reliability and validity of the mother-child neglect scale. *Child Maltreat* 2004;**9**:371–81	3
240	Lovejoy M, Verda MR, Hays CE. Convergent and discriminant validity of measures of parenting efficacy and control. *J Clin Child Psychol* 1997;**26**:366–76	3
241	Lyons-Ruth K, Yellin C, Melnick S, Atwood G. Expanding the concept of unresolved mental states: Hostile/Helpless states of mind on the Adult Attachment Interview are associated with disrupted mother-infant communication and infant disorganization. *Dev Psychopathol* 2005;**17**:1–23	3
242	Ma CQ, Huebner ES. Attachment relationships and adolescents' life satisfaction: some relationships matter more to girls than boys. *Psychol Schools* 2008;**45**:177–90	3
243	Madigan S, Bakermans-Kranenburg MJ, Van IJzendoorn MH, Moran G, Pederson DR, Benoit D. Unresolved states of mind, anomalous parental behavior, and disorganized attachment: a review and meta-analysis of a transmission gap. *Attach Hum Dev* 2006;**8**:89–111	1
244	Madigan S, Moran G, Pederson DR. Unresolved states of mind, disorganized attachment relationships, and disrupted interactions of adolescent mothers and their infants. *Dev Psychol* 2006;**42**:293–304	3
245	Maier MA, Bernier A, Perkrun R, Zimmermann P, Grossmann KE. Attachment working models as unconscious structures: an experimental test. *Int J Behav Dev* 2004;**28**:180–9	5
246	Manassis K, Bradley S, Goldberg S, Hood J, Swinson RP. Behavioural inhibition, attachment and anxiety in children of mothers with anxiety disorders. *Can J Psychiatry* 1995;**40**:87–92	1
247	Manassis K, Owens M, Adam KS, West M, Sheldon-Keller AE. Assessing attachment: Convergent validity of the Adult Attachment Interview and the Parental Bonding Instrument. *Aust N Z J Psychiatry* 1999;**33**:559–67	5
248	Mangelsdorf SC, McHale JL, Diener M, Goldstein LH, Lehn L. Infant attachment: contributions of infant temperament and maternal characteristics. *Infant Behav Dev* 2000;**23**:175–96	1
249	Marcus RF, Kramer C. Reactive and proactive aggression: attachment and social competence predictors. *J Genet Psychol* 2001;**162**:260–75	3
250	Marcus RF. Concordance between parent inventory and directly observed measures of attachment. *Early Child Dev Care* 1997;**135**:109–17	1
251	Marcus RF. The attachments of children in foster care. *Genetic, Social, and General Psychology Monographs* 1991;**117**:365–94	1
252	Marcus RF. The Parent/Child Reunion Inventory: a measure of attachment for children beyond the infancy years. *Psychological Reports* 1990;**67**:1329–30	3

Number	Reference	Reason
253	Marecki M, Wooldridge P, Dow A, Thompson J, Lechner-Hyman C. Early sibling attachment. *J Obstet Gynecol Neonatal Nurs* 1985;**14**:418–23	3
254	Maslin-Cole C, Spieker SJ. Attachment as a Basis for Independent Motivation: A View from Risk and Nonrisk Samples. In Greenberg M, Cicchetti D, Cummings M, editors. *Attachment in the Preschool Years: Theory, Research, and Intervention*. Chicago, IL: University of Chicago Press; 1990. pp. 245–72	3
255	McCall RB, Groark CJ, Fish L. A caregiver-child socioemotional and relationship rating scale. *Infant Ment Health J* 2010;**31**:201–19	3
256	McCarthy GDE. *Doing Well and Doing Poorly in Care: Caregivers' Attachment Status and Other Risk and Resilience Predictors of Children's Outcomes in Kinship, Foster, and Adoptive Placements*. PhD thesis. York: Smith College School of Social Work; 2007	4
257	McCormack D, Scott-Heyes G, McCusker CG. The impact of hyperemesis gravidarum on maternal mental health and maternal-fetal attachment. *J Psychosom Obstet Gynecol* 2011;**32**:79–87	3
258	McFarland J, Salisbury AL, Battle CL, Hawes K, Halloran K, Lester BM. Major depressive disorder during pregnancy and emotional attachment to the fetus. *Arch Womens Ment Health* 2011;**14**:425–34	3
259	McHale JL. *Infant-Father Attachment: Infant and Father Antecedents.* PhD thesis. Urbana–Champaign, IL: University of Illinois at Urbana–Champaign; 1997	4
260	McKenzie LB. *Development of an Adult Attachment Scale: Preliminary Study.* PhD thesis. Fort Worth, TX: Texas Christian University; 2011	3
261	Mercer RT, Ferketich S, May K, DeJoseph J, Sollid D. Further exploration of maternal and paternal fetal attachment. *Res Nurs Health* 1988;**11**:83–95	3
262	Mikhail MS, Freda MC, Merkatz RB, Polizzotto R, Mazloom E, Merkatz IR. The effect of fetal movement counting on maternal attachment to fetus. *Am J Obstet Gynecol* 1991;**165**:988–91	3
263	Mikulincer M, Florian V. Maternal–fetal bonding, coping strategies, and mental health during pregnancy – the contribution of attachment style. *J Soc Clin Psychol* 1999;**18**:255–76	3
264	Miller WB, Sable MR, Csizmadia A. Pregnancy wantedness and child attachment security: is there a relationship? *Matern Child Health J* 2008;**12**:478–87	1
265	Millione C, Corsano P, Cassibba R. The evaluation of child-teacher attachment: a preliminary study for the validation of the Attachment Q-sort in preschool age. *Eta Evolutiva* 2005;**80**:29–40	3
266	Minde K, Minde R, Vogel W. Culturally sensitive assessment of attachment in children aged 18–40 months in a South African township. *Infant Ment Health J* 2006;**27**:544–58	2
267	Minnis H, Millward R, Sinclair C, Kennedy E, Greig A, Towlson K, *et al.* The Computerized MacArthur Story Stem Battery – a pilot study of a novel medium for assessing children's representations of relationships. *Int J Methods Psychiatr Res* 2006;**15**:207–14	2
268	Minnis H, Rabe-Hesketh S, Wolkind S. Development of a brief, clinically relevant, scale for measuring attachment disorders. *Int J Methods Psychiatr Res* 2002;**11**:90–8	2
269	Minnis H, Ramsay R, Campbell L. Reactive attachment disorder: usefulness of a new clinical category. *J Nerv Ment Dis* 1996;**184**:440	1
270	Minnis H. Reactive attachment disorder. *J Am Acad Child Adolesc Psychiatry* 2001;**40**:129–33	5
271	Mizukami K, Kobayashi N, Iwata H, Ishii T. Telethermography in Measurement of Infants Early Attachment. In Von Euler C, Forssberg H, Lagercrantz H. *Neurobiology of Early Infant Behaviour. Wenner-Gren Center International Symposium Series. 55*. New York, NY: Stockton Press; 1989. pp. 249–59	2
272	Mohr S, Preisig M, Fenton B, Ferrero F. Validation of the French version of the Parental Bonding Instrument in adults. *Pers Individ Diff* 1999;**26**:1065–74	3
273	Morgan FT. *Development of a Measure to Screen for Reactive Attachment Disorder in Children 0–5 Years*. PhD thesis. Fresno, CA: Alliant International University, California School of Professional Psychology; 2005	2
274	Moss E, Bureau J-F, Cyr C, Dubois-Comtois K. Is the maternal Q-Set a valid measure of preschool child attachment behavior? *Int J Behav Dev* 2006;**30**:488–97	1

© Queen's Printer and Controller of HMSO 2015. This work was produced by Wright *et al.* under the terms of a commissioning contract issued by the Secretary of State for Health. This issue may be freely reproduced for the purposes of private research and study and extracts (or indeed, the full report) may be included in professional journals provided that suitable acknowledgement is made and the reproduction is not associated with any form of advertising. Applications for commercial reproduction should be addressed to: NIHR Journals Library, National Institute for Health Research, Evaluation, Trials and Studies Coordinating Centre, Alpha House, University of Southampton Science Park, Southampton SO16 7NS, UK.

Number	Reference	Reason
275	Moss E, Bureau J-F, Cyr C, Mongeau C, St-Laurent D. Correlates of attachment at age 3: construct validity of the preschool attachment classification system. *Dev Psychol* 2004;**40**:323–34	4
276	Muller ME, Ferketich S. Assessing the validity of the dimensions of prenatal attachment. *Maternal-Child Nursing Journal* 1992;**20**:1–10	3
277	Muller ME, Ferketich S. Factor analysis of the Maternal Fetal Attachment Scale. *Nurs Res* 1993;**42**:144–7	3
278	Muller ME. A questionnaire to measure mother-to-infant attachment. *J Nurs Measure* 1994;**2**:129–41	3
279	Muller ME. *The Development and Testing of the Muller Prenatal Attachment Inventory.* San Francisco, CA: University of California, San Francisco; 1990	3
280	Murray L, Cooper PJ. The clinical application of ethology and attachment theory. In Richer J, editor. *Clinical Applications of Attachment Theory and Research: Change in Infant Attachment with Brief Psychotherapy.* Oxford: ACPP, 1994. pp. vi–64.	1
281	Murray L, Woolgar M, Briers M, Hipwell A. Children's social representations in dolls' house play and theory of mind tasks, and their relation to family adversity and child disturbance. *Soc Dev* 1999;**8**:179–200	1
282	Murray MP. *Attachment Relationships and Empathic Response in Young People who Sexually Abuse*. PhD thesis. Belfast: Queen's University Belfast; 2002	5
283	Music G. *Nurturing Natures: Attachment and Children's Emotional, Sociocultural and Brain Development*. Hove: Psychology Press; 2011	1
284	Narita S, Maehara S. The development of maternal-fetal attachment during pregnancy. *Nihon Kango Kagakkaishi* 1993;**13**:1–9	3
285	National Institute of Child Health and Human Development Early Child Care Research Network. *NICHD Study of Early Child Care. Phase I [CD-ROM]* 1999. URL: http://public.rti.org/secc/ (accessed 17 June 2013)	1
286	Neisworth JT, Bagnato SJ, Salvia J, Hunt FM. *TABS Manual for the Temperament and Atypical Behavior Scale: Early Childhood Indicators of Developmental Dysfunction*. Baltimore, MD: Paul H. Brookes Publishing Co.; 1999	3
287	Niederhofer H, Reiter A. About the association between attachment and temperament at the age of 6 months. *Psychiatria Danubina* 2003;**15**:7–12	3
288	Niederhofer H, Reiter A. Does the maternal appraisal of attachment to the child remain stable from the prenatal phase to the postnatal period, and can it be objectively assessed from intrauterine activity? *Psychiatria Danubina* 2002;**14**:35–40	1
289	Niederhofer H. Attachment as a component of attention-deficit hyperactivity disorder. *Psychological Reports* 2009;**104**:645–8	4
290	Niederhofer H. [Long term effects of prepartal mother-child-attachment.] *Z Geburtshilfe Neonatol* 2007;**211**:82–6	1
291	Nievar MA, Becker BJ. Sensitivity as a privileged predictor of attachment: a second perspective on De Wolff and Van IJzendoorn's meta-analysis. *Soc Dev* 2008;**17**:102–14	1
292	O'Connor TG, Bredenkamp B, Rutter M. Attachment disturbances and disorders in children exposed to early severe deprivation. *Infant Ment Health J* 1999;**20**:10–29	1
293	O'Connor MJ, Masten AS. Use of the strange-situation procedure in the diagnosis of attachment disorder. *Child Psychiatry Hum Dev* 1984;**15**:64–71	1
294	Ortiz JA, Borre A, Carrillo S, Gutierrez G. The effect of attachment in adolescent mothers and Kangaroo program babies. *Revista Latinoamericana de Psicologia* 2006;**38**:71–86	1
295	Out D, Bakermans-Kranenburg MJ, Van IJzendoorn MH. The role of disconnected and extremely insensitive parenting in the development of disorganized attachment: validation of a new measure. *Attach Hum Dev* 2009;**11**:419–43	3
296	Oxford ML, Harachi TW, Catalano RF, Haggerty KP, Abbott RD. Early elementary school-aged child attachment to parents: a test of theory and implications for intervention. *Prevent Sci* 2000;**1**:61–9	3

Number	Reference	Reason
297	Padron E. *The Disorganized Dyad: The Roles of Mother and Child in the Development of Disorganized Attachment*. PhD thesis. Minneapolis, MN: University of Minnesota; 2004	1
298	Palacios J. Family context for emotional recovery in internationally adopted children. *Int Soc Work* 2009;**52**:609–20	1
299	Paquette D, Bigras M. The risky situation: a procedure for assessing the father-child activation relationship. *Early Child Dev Care* 2010;**180**:33–50	3
300	Parent S, Gosselin C, Moss E. From mother-regulated to child-regulated joint planning activity: A look at familial adversity and attachment. *J Appl Dev Psychol* 2000;**21**:447–70	1
301	Parent S, Moss E. The influence of mother-child attachment and preschool child's verbal skills on maternal scaffolding in a simple planning task. *Enfance* 1995;**3**:317–35	1
302	Parez T, Di Gallo A, Schmeck K, Schmid M. Interpersonal trauma and the correlation with attachment difficulties and psychopathology among children in foster care. *Kindheit und Entwicklung* 2011;**20**:72–82	1
303	Parisi D, Cecconi F, Cerini A. Kin-Directed Altruism and Attachment Behaviour in an Evolving Population of Neural Networks. In Gilbert N, Conte R, editors. *Artificial Societies: Computer Simulation of Social Life*. Siena: UCL Press; 1995	1
304	Patino FL. *Mothers' Prenatal Attachment History, Attachment Status, and Social Support Satisfaction as Predictors of Postnatal Maternal Sensitivity*. PhD thesis. San Diego: California School of Professional Psychology; 1993	3
305	Pearson RM, Lightman SL, Evans J. Attentional processing of infant emotion during late pregnancy and mother–infant relations after birth. *Arch Womens Ment Health* 2011;**14**:23–31	3
306	Pederson DR, Gleason KE, Moran G, Bento S. Maternal attachment representations, maternal sensitivity, and the infant-mother attachment relationship. *Dev Psychol* 1998;**34**:925–33	3
307	Pederson DR, Moran G, Sitko C, Campbell K, *et al.* Maternal sensitivity and the security of infant-mother attachment: a Q-sort study. *Child Dev* 1990;**61**:1974–83	3
308	Pederson DR, Moran G. A categorical description of infant–mother relationships in the home and its relation to Q-sort measures of infant–mother interaction. *Monogr Soc Res Child Dev* 1995;**60**:111–32	1
309	Pederson DR, Moran G. Expressions of the attachment relationship outside of the Strange Situation. *Child Dev* 1996;**67**:915–27	3
310	Pederson DR. *Maternal Sensitivity and Attachment Security: Concordance of Home- and Lab-Based Measures*. Report number ED349101. Toronto: Ontario Mental Health Foundation; 1992	3
311	Philipp DA, Herve MJ, Keren M. Does the portal of entry determine our view? Interfaces between dyadic and three-way assessment of a clinical family transitioning to parenthood. *Infant Ment Health J* 2008;**29**:175–295	1
312	Pianta RC, Longmaid K, Ferguson JE. Attachment-based classifications of children's family drawings: psychometric properties and relations with children's adjustment in kindergarten. *J Clin Child Psychol* 1999;**28**:244–55	2
313	Pierrehumbert B, Muhlemann I, Antonietti J-P, Sieye A. Validation of a French version of Waters and Deane's Attachment Q-Sort. *Enfance* 1995;**3**:293–315	1
314	Pierrehumbert B, Santelices MP, Ibanez M, Alberdi M, Ongari B, Roskam I, *et al.* Gender and attachment representations in the preschool years: comparisons between five countries. *J Cross Cult Psychol* 2009;**40**:543–66	1
315	Pinel-Jacquemin S, Zaouche-Gaudron C, Troupel-Cremel O. Father-child attachment and quality of the sibling relationships. *Psychologie Francaise* 2009;**54**:307–22	1
316	Pisciella AE. *Estimating Effects of Participation in Parental Leave on Children's and Mothers' Well-Being.* PhD thesis. Bronx, NY: Fordham University; 2008	1
317	Pollock PH, Percy A. Maternal antenatal attachment style and potential fetal abuse. *Child Abuse Negl* 1999;**23**:1345–57	3
318	Pool MM, Bijleveld CC, Tavecchio LW. The effect of same-age and mixed-age grouping in day care on parent–child attachment security. *Soc Behav Pers* 2000;**28**:595–602	1

© Queen's Printer and Controller of HMSO 2015. This work was produced by Wright *et al.* under the terms of a commissioning contract issued by the Secretary of State for Health. This issue may be freely reproduced for the purposes of private research and study and extracts (or indeed, the full report) may be included in professional journals provided that suitable acknowledgement is made and the reproduction is not associated with any form of advertising. Applications for commercial reproduction should be addressed to: NIHR Journals Library, National Institute for Health Research, Evaluation, Trials and Studies Coordinating Centre, Alpha House, University of Southampton Science Park, Southampton SO16 7NS, UK.

Number	Reference	Reason
319	Popper SD. *Social and Object Mastery Play in 12-Month Olds with Depressed and Non-Depressed Mothers: Developmental Changes and Correlates*. Report number ED357879. Bethesda, MD: National Institute of Mental Health (DHHS); 1993	1
320	Portnoy FC, Simmons CH. Day care and attachment. *Child Dev* 1978;**49**:239–42	1
321	Posada G, Carbonell OA, Alzate G, Plata SJ. Through Colombian lenses: ethnographic and conventional analyses of maternal care and their associations with secure base behavior. *Dev Psychol* 2004;**40**:508–18	2
322	Posada G, Jacobs A, Carbonell OA, Alzate G, Bustamante MR, Arenas A. Maternal care and attachment security in ordinary and emergency contexts. *Dev Psychol* 1999;**35**:1379–88	3
323	Posada G, Jacobs A, Richmond MK, Carbonell OA, Alzate G, Bustamante MR, Quiceno J. Maternal caregiving and infant security in two cultures. *Dev Psychol* 2002;**38**:67–78	1
324	Posada G, Jacobs A. Child-mother attachment relationships and culture. *Am Psychol* 2001;**56**:821–2	1
325	Priel B, Besser A. Bridging the gap between attachment and object relations theories: a study of the transition to motherhood. *Br J Med Psychol* 2001;**74**:85–100	3
326	Propper C, Willoughby M, Halpern CT, Carbone MA, Cox M. Parenting quality, DRD4, and the prediction of externalizing and internalizing behaviors in early childhood. *Dev Psychobiol* 2007;**49**:619–32	1
327	Propper CB. *Infant Behavioral and Physiological Regulation during the Strange Situation Procedure at 12-months: Contributions of Genotype and Maternal Behavior.* PhD thesis. Chapel Hill, NC: University of North Carolina; 2007	1
328	Puentes-Neuman G. Toddlers' social coordination with an unfamiliar peer: Patternings of attachment, temperament, and coping during dyadic exchange. Montréal: Université de Montréal. *Dissertation Abstracts International: Section B: The Sciences and Engineering*; 2000	1
329	Pure K. *Instructional Antecedents to Children's Compliant Behaviour.* Fredericton, NB: University of New Brunswick; 2002	1
330	Raaska H, Sinkkonen J, Lapinleimu H, Makipaa S, Elovainio M. Attachment problems among internationally adopted toddlers in Finland; the FINADO-study. *Eur Child Adolesc Psychiatry* 2011. 14th International Congress of ESCAP European Society for Child and Adolescent Psychiatry, 2011, Helsinki, Finland	1
331	Rabung S, Ubbelohde A, Kiefer E, Schauenburg H. Attachment security and quality of life in atopic dermatitis. *Psychother Psychosom Med Psychol* 2004;**54**:330–8	1
332	RadojeviA M. Prenatal paternal representations of attachment predict of infant-father attachment at 15 months: an Australian study. *Psihijatrija Danas* 2005;**37**:257–87	3
333	Radtke LT. *Attachment, Parenting Behaviors, and Negative Affect: Are They Related to Effortful Control at Age 4?* PhD thesis. Chicago, IL: Illinois Institute of Technology; 2009	1
334	Ramos-Marcuse F, Arsenio WF. Young children's emotionally-charged moral narratives: Relations with attachment and behavior problems. *Early Educ Dev* 2001;**12**:165–84	1
335	Ramos-Marcuse FM. *Children's Moral Narratives: Links to Aggression and Early Relationships: Ramos-Marcuse*. PhD thesis. New York, NY: Yeshiva University; 2002	1
336	Reading AE, Cox DN, Sledmere CM, Campbell S. Psychological changes over the course of pregnancy: a study of attitudes toward the fetus/neonate. *Health Psychol* 1984;**3**:211–21	3
337	Reardon PA. *A Primary Care Setting Screening for Risk Factors to Attachment Security.* PhD thesis. Antioch University/New England Graduate School; 2001	3
338	Reck C, Klier C, Pabst K, Stehle E, Steffenelli U, Struben K, *et al.* The German version of the Postpartum Bonding Instrument: Psychometric properties and association with postpartum depression. *Arch Womens Ment Health* 2006;**9**:265–71	3
339	Rellinger EA. *Precursors and Consequences of Insecure Attachment in Children of Adolescent Mothers*. PhD thesis. University of Notre Dame; 1995	1
340	Rembert C. *Female Adolescent Ego Development and How it is Related to Attachment and Depression: A Longitudinal Study*. New York, NY: Pace University. *Dissertation Abstracts International: Section B: The Sciences and Engineering*; 2007	3

Number	Reference	Reason
341	Ren Z, Wang D, Yang A, Li M, Higgins LT. Implicit and explicit measures of adult attachment to mothers in a Chinese context. *Soc Behav Pers* 2011;**39**:701–12	1
342	Richters MM, Volkmar FR. Reactive attachment disorder of infancy or early childhood. *J Am Acad Child Adolesc Psychiatry* 1994;**33**:328–32	1
343	Ridenour TA, Greenberg MT, Cook ET. Structure and validity of people in my life: a self-report measure of attachment in late childhood. *J Youth Adolesc* 2006;**35**:1037–53	2
344	Righetti P, Dell'Avanzo M, Grigio M, Nicolini U. Maternal/paternal antenatal attachment and fourth-dimensional ultrasound technique: a preliminary report. *Br J Psychol* 2005;**96**:129–37	1
345	Rimashevskaia NV, Kremneva LF. Mental development of young children in maternal behavior disorders. *Zh Nevrol Psikhiatr Im S S Korsakova* 2003;**103**:19–24	1
346	Roberto A, Carlyle K, Goodall C, Castle J. The relationship between parents' verbal aggressiveness and responsiveness and young adult children's attachment style and relational satisfaction with parents. *J Fam Commun* 2009;**9**:90–106	5
347	Robinson LL. *Caregivers' Internal Working Models: The Circle of Security Interview Classification System*. Charlottesville, VA: University of Virginia. *Dissertation Abstracts International: Section B: The Sciences and Engineering*; 2003	3
348	Roccato M, Tartaglia S. An Italian adaptation of the Carver's Measure of attachment qualities. *Testing Psicometria Metodologia* 2003;**10**:65–78	5
349	Rodning C, Beckwith L, Howard J. Prenatal exposure to drugs and its influence on attachment. *Ann N Y Acad Sci* 1989;**562**:352–4	3
350	Rodrigues AR, Perez-Lopez J, de la Nuez AG. Prenatal attachment and anxiety during the final trimester of pregnancy in early parenthood: A preliminary study. *Anales de Psicologia* 2004;20(1):95–102	3
351	Roehrig C, Genet C, Cyrulnik B. An ethological observation: styles of attachment and evocation of pregnancy during adolescence. *Sexologies* 2006;**15**:134–41	5
352	Roelofs J, Meesters C, ter Huurne M, Bamelis L, Muris P. On the links between attachment style, parental rearing behaviors, and internalizing and externalizing problems in non-clinical children. *J Child Family Stud* 2006;**15**:331–44	1
353	Roisman GI, Fraley R, Belsky J. A taxometric study of the Adult Attachment Interview. *Dev Psychol* 2007;**43**:675–86	5
354	Roopnarine JL, Lamb ME. The effects of day care on attachment and exploratory behavior in a strange situation. *Merrill-Palmer Q* 1978;**24**:85–95	3
355	Royal College of Psychiatrists. *Attachment Disorders. Part 2 The Evidence*. London: Royal College of Psychiatrists; 2002. pp. 153–6	1
356	Sazvar S, Ezabadi Z, Omani Samani R, Eshrati B. Validation of Iranian version of the prenatal attachment inventory. *J Reproduktionsmedizin Endokrinologie* 2010. 20th World Congress on Fertility and Sterility, 2010, Munich, Germany	3
357	Scharfe E. Cause or consequence?: Exploring causal links between attachment and depression. *J Soc Clin Psychol* 2007;**26**:1048–64	3
358	Schiller M. *Maternal Interaction Style, the Family and Attachment Outcomes.* PhD thesis. Kingston, RI: University of Rhode Island; 1995	1
359	Schwark B, Schmidt S, Strauss B. A study of the relationship between attachment patterns and problem perception in a sample of 9–11-year-old children with behavioral disorders. *Praxis Kinderpsychol Kinderpsychiatrie* 2000;**49**:340–50	1
360	Schwerdtfeger KL, Goff BS. Intergenerational transmission of trauma: exploring mother–infant prenatal attachment. *J Trauma Stress* 2007;**20**:39–51	5
361	Scopesi A, Viterbori P, Sponza S, Zucchinetti P. Assessing mother-to-infant attachment: the Italian adaptation of a self-report questionnaire. *J Reprod Infant Psychol* 2004;**22**:99–109	3
362	Seifer R, Schiller M, Sameroff AJ, Resnick S, Riordan K. Attachment, maternal sensitivity, and infant temperament during the first year of life. *Dev Psychol* 1996;**32**:12–25	3
363	Seimyr L, Sjogren B, Welles-Nystrom B, Nissen E. Antenatal maternal depressive mood and parental-fetal attachment at the end of pregnancy. *Arch Womens Ment Health* 2009;**12**:269–79	5

© Queen's Printer and Controller of HMSO 2015. This work was produced by Wright *et al.* under the terms of a commissioning contract issued by the Secretary of State for Health. This issue may be freely reproduced for the purposes of private research and study and extracts (or indeed, the full report) may be included in professional journals provided that suitable acknowledgement is made and the reproduction is not associated with any form of advertising. Applications for commercial reproduction should be addressed to: NIHR Journals Library, National Institute for Health Research, Evaluation, Trials and Studies Coordinating Centre, Alpha House, University of Southampton Science Park, Southampton SO16 7NS, UK.

Number	Reference	Reason
364	Shaw HE. *The COURT Diagram: Validation of a Self-Report Instrument of Relationship Functioning.* PhD thesis. University of Northern Colorado; 2005	4
365	Sher-Censor E, Oppenheim D. Children attachment narratives and early attachment behaviors. *Enfance* 2008;**60**:31–41	2
366	Shieh C, Kravitz M. Severity of drug use, initiation of prenatal care, and maternal-fetal attachment in pregnant marijuana and cocaine/heroin users. *J Obstetr Gynecol Neonatal Nurs* 2006;**35**:499–508	3
367	Shieh W-H. *Maternal-Fetal Attachment in Illicit-Drug-Using Pregnant Women.* PhD thesis. Kokomo, IN: School of Nursing, Indiana University; 1999	3
368	Shields A, Ryan RM, Cicchetti D. Narrative representations of caregivers and emotion dysregulation as predictors of maltreated children's rejection by peers. *Dev Psychol* 2001;**37**:321–37	1
369	Shin H, Kim YH. Maternal Attachment Inventory: psychometric evaluation of the Korean version. *J Adv Nurs* 2007;**59**:299–307	3
370	Shouldice AE, Stevenson-Hinde J. Coping with security distress: the Separation Anxiety Test and attachment classification at 4.5 years. *J Child Psychol Psychiatry* 1992;**33**:331–48	1
371	Siddiqui A, Eisemann M, Hagglof B. The stability of maternal interpretation of infant's facial expressions during pre- and postnatal period and its relation to prenatal attachment. *Early Child Dev Care* 2000;**162**:41–50	3
372	Siddiqui A, Hagglof B, Eisemann M. An exploration of prenatal attachment in Swedish expectant women. *J Reprod Infant Psychol* 1999;**17**:369–80	3
373	Siddiqui A, Hagglof B. Does maternal prenatal attachment predict postnatal mother–infant interaction? *Early Hum Dev* 2000;**59**:13–25	3
374	Sideridis GD, Kafetsios K. Perceived parental bonding, fear of failure and stress during class presentations. *Int J Behav Dev* 2008;**32**:119–30	1
375	Silven M, Vienola M. From mother–infant interaction to attachment security. *Psykologia* 1995;**30**:445–57	2
376	Slade P, Laxton-Kane M, Spiby H. Smoking in pregnancy: the role of the transtheoretical model and the mother's attachment to the fetus. *Addict Behav* 2006;**31**:743–57	3
377	Smeekens S, Riksen-Walraven J, van Bakel HJ. Multiple determinants of externalizing behavior in 5-year-olds: a longitudinal model. *J Abnorm Child Psychol* 2007;**35**:347–61	1
378	Smith KE, Landry SH, Swank PR. The influence of decreased parental resources on the efficacy of a responsive parenting intervention. *J Consult Clin Psychol* 2005;**73**:711–20	1
379	Smith PB. *Infant-Mother Attachment as a Function of Maternal Sensitivity, Cognition, Personality, and Social Support.* PhD thesis. University of Western Ontario; 1988	1
380	Smolen AG. Boys only! No mothers allowed. *Int J Psychoanal* 2009;**90**:1–11	1
381	Smyke AT, Dumitrescu A, Zeanah CH. Attachment disturbances in young children. I: the continuum of caretaking casualty. *J Am Acad Child Adolesc Psychiatry* 2002;**41**:972–82	1
382	Sneddon H, Ferriter M, Macdonald G. CBT interventions for young people aged 10–18 who sexually offend. *Cochrane Database Syst Rev* 2012;**5**:CD009829	1
383	Sokol-Katz JS. *The Relationships between Parental Attachment/Family Structure and Deviant Behavior: A Test of the Social Control Theory*. Miami, FL: University of Miami; 1993	1
384	Solomon J, George C. The Measurement of Attachment Security and Related Constructs in Infancy and Early Childhood. In Cassidy J, Shaver PR, editors. *Handbook of Attachment: Theory, Research, and Clinical Applications*. 2nd edn. New York, NY: Guilford Press; 2008. pp. 383–416	1
385	Spangler G, Grossmann K. Biobehavioral organization in securely and insecurely attached infants. *Child Dev* 1993;**64**:1439–50	1
386	Spangler G, Grossmann KE, Schieche M. Biobehavioral organization of the attachment system in infancy. *Psychologie Erziehung Unterricht* 2002;**49**:102–20	1
387	Spangler G, Schieche M. Biobehavioral organization in one-year-olds: quality of mother-infant attachment and immunological and adrenocortical regulation. *Psychologische Beitrage* 1994;**36**:30–5	1

Number	Reference	Reason
388	Spangler G, Schieche M. Emotional and adrenocortical responses of infants to the strange situation: the differential function of emotional expression. *Int J Behav Dev* 1998;**22**:681–706	4
389	Speltz ML, Greenberg MT, Deklyn M. Attachment in preschoolers with disruptive behaviour: a comparison of clinic-referred and non-problem children. *Dev Psychopathol* 1990;**2**:31–46	2
390	Speltz ML, DeKlyen M, Greenberg MT, Dryden M. Clinic referral for oppositional defiant disorder: relative significance of attachment and behavioral variables. *J Abnorm Child Psychol* 1995;**23**:487–507	1
391	Speltz ML, DeKlyen M, Greenberg MT. Attachment in boys with early onset conduct problems. *Dev Psychopathol* 1999;**11**:269–85	1
392	Spieker S, Nelson EM, Condon M-C. Validity of the TAS-45 as a measure of toddler-parent attachment: preliminary evidence from Early Head Start families. *Attach Hum Dev* 2011;**13**:69–90	3
393	Spieker SJ, Bensley L. Roles of living arrangements and grandmother social support in adolescent mothering and infant attachment. *Dev Psychol* 1994;**30**:102–11	1
394	Spitzer S. *Maternal Distress Regulation and Dyadic Repair: Contributions to Infant Socio-Emotional Functioning*. PhD thesis. New York, NY: City University; 2001	3
395	Spletzer PU. *Mutual Regulation: The Relationship between Maternal-Fetal Attachment, Maternal Prenatal Sleep, and Postnatal Infant Sleep Patterns.* PhD thesis. Carpinteria, CA: Pacifica Graduate Institute; 2006	3
396	Stainton MC. *Origins of Attachment: Culture and Cue Sensitivity.* PhD thesis. San Francisco, CA: University of California; 1986	3
397	Stanford DD. *The Effect of Fetal Ultrasound Imaging on Parental-Fetal Attachment and on Dyadic Attachment of the Expectant Couple*. California School Of Professional Psychology – Berkeley/Alameda; 2002	3
398	Stauffer S. Trauma and disorganized attachment in refugee children: integrating theories and exploring treatment options. *Refugee Survey Q* 2008;**27**:150–63	1
399	Stayton DJ, Salter Ainsworth MD. Individual differences in infant responses to brief, everyday separations as related to other infant and maternal behaviors. *Dev Psychol* 1973;**9**:226–35	3
400	Steele H, Steele M, Croft C. Early attachment predicts emotion recognition at 6 and 11 years old. *Attach Hum Dev* 2008;**10**:379–93	1
401	Steele H, Steele M. The Construct of Coherence as an Indicator of Attachment Security in Middle Childhood: The Friends and Family Interview. In Kerns KA, Richardson RA, editors. *Attachment in Middle Childhood*. New York, NY: Guilford Press; 2005. pp. 137–60	3
402	Stefankova Z. Attachment in early adolescence. *Ceskoslovenska Psychologie* 2007;**51**:503–16	5
403	Sternberg KJ, Lamb ME. Evaluations of attachment relationships by Jewish Israeli day-care providers. *J Cross Cult Psychol* 1992;**23**:285–99	5
404	Stevenson-Hinde J, Marshall PJ. Behavioral inhibition, heart period, and respiratory sinus arrhythmia: an attachment perspective. *Child Dev* 1999;**70**:805–16	3
405	Stevenson-Hinde J, Shouldice A. Fear and attachment in 2.5-year-olds. *Br J Dev Psychol* 1990;**8**:319–33	3
406	Stewart RB, Marvin RS. Sibling relations: the role of conceptual perspective-taking in the ontogeny of sibling caregiving. *Child Dev* 1984;**55**:1322–32	3
407	Sumpter JM. Reactive Attachment Disorder: Developing a Developmental Perspective. 13th Annual CECP Research Exchange Conference Marquette University, 2011, Milwaukee, WI, USA	1
408	Svanberg P, Mennet L, Spieker S. Promoting a secure attachment: a primary prevention practice model. *Clin Child Psychol Psychiatry* 2010;**15**:363–78	1
409	Szajnberg NM, Skrinjaric J, Moore A. Affect attunement, attachment, temperament, and zygosity: a twin study. *J Am Acad Child Adolesc Psychiatry* 1989;**28**:249–53	1
410	Takahashi K. Examining the strange-situation procedure with Japanese mothers and 12-month-old infants. *Dev Psychol* 1986;**22**:265–70	2

© Queen's Printer and Controller of HMSO 2015. This work was produced by Wright *et al.* under the terms of a commissioning contract issued by the Secretary of State for Health. This issue may be freely reproduced for the purposes of private research and study and extracts (or indeed, the full report) may be included in professional journals provided that suitable acknowledgement is made and the reproduction is not associated with any form of advertising. Applications for commercial reproduction should be addressed to: NIHR Journals Library, National Institute for Health Research, Evaluation, Trials and Studies Coordinating Centre, Alpha House, University of Southampton Science Park, Southampton SO16 7NS, UK.

Number	Reference	Reason
411	Tarabulsy GM, Provost MA, Bordeleau S, Trudel-Fitzgerald C, Moran G, Pederson DR, *et al.* Validation of a short version of the maternal behavior Q-set applied to a brief video record of mother–infant interaction. *Infant Behav Dev* 2009;**32**:132–6	3
412	Target M, Fonagy P, Shmueli-Goetz Y. Attachment representations in school-age children: the development of the Child Attachment Interview (CAI). *J Child Psychother* 2003;**29**:171–86	1
413	Tarren-Sweeney M. The Assessment Checklist for Children – ACC: a behavioral rating scale for children in foster, kinship and residential care. *Child Youth Serv Rev* 2007;**29**:672–91	3
414	Tavecchio L, Van IJzendoorn M. Discriminant validity of scales for temperament and perceived security. *Gedrag: Tijdschrift voor Psychologie* 1985;**13**:79–95	3
415	Taylor A, Atkins R, Kumar R, Adams D, Glover V. A new Mother-to-Infant Bonding Scale: links with early maternal mood. *Arch Womens Ment Health* 2005;**8**:45–51	3
416	Teti DM, Gelfand DM. The Preschool Assessment of Attachment: construct validity in a sample of depressed and nondepressed families. *Dev Psychopathol* 1997;**9**:517–36	2
417	Teti DM, Killeen LA, Candelaria M, Miller W, Hess CR, O'Connell M. Adult Attachment, Parental Commitment to Early Intervention, and Developmental Outcomes in an African American Sample. In Steele H, Steele M, editors. *Clinical Applications of the Adult Attachment Interview.* New York, NY: Guilford Press; US; 2008. pp. 126–53	4
418	Teti DM, McGourty S. Using mothers versus trained observers in assessing children's secure base behavior: theoretical and methodological considerations. *Child Dev* 1996;**67**:597–605	2
419	Teti DM. *Maternal Depression and Child-Mother Attachment in the First Three Years: A View from the Intermountain West.* Crittenden PM, Claussen AH, editors. *The Organization of Attachment Relationships: Maturation, Culture, and Context*. New York, NY: Cambridge University Press; 2000. pp. 190–213	1
420	Thomas J, Clar R. Disruptive behavior in the very young child: diagnostic Classification: 0–3 guides identification of risk factors and relational interventions. *Infant Ment Health J* 1998;**19**:229–44	2
421	Thompson RA, Connell JP, Bridges LJ. Temperament, emotion, and social interactive behavior in the Strange Situation: a component process analysis of attachment system functioning. *Child Dev* 1988;**59**:1102–10	1
422	Tilbrook DMW. *Attachment, Conduct Disorder and Perspective Taking in 7–9 Year Old Boys.* PhD thesis. Open University; 2000	3
423	Toth SL, Maughan A, Manly JT, Spagnola M, Cicchetti D. The relative efficacy of two interventions in altering maltreated preschool children's representational models: implications for attachment theory. *Dev Psychopathol* 2002;**14**:877–908	1
424	Toth SL, Rogosch FA, Sturge-Apple M, Cicchetti D. Maternal depression, children's attachment security, and representational development: an organizational perspective. *Child Dev* 2009;**80**:192–208	1
425	Tracy RL, Salter Ainsworth MD. maternal affectionate behavior and infant-mother attachment patterns. *Child Dev* 1981;**52**:1341–3	3
426	Trapolini T, Ungerer J, McMahon C. Maternal depression and children's attachment representation during the preschool years. *Br J Dev Psychol* 2007;**25**:247–61	3
427	Tsartsara E, Johnson MP. The impact of miscarriage on women's pregnancy-specific anxiety and feelings of prenatal maternal-fetal attachment during the course of a subsequent pregnancy: an exploratory follow-up study. *J Psychosom Obstetr Gynecol* 2006;**27**:173–82	3
428	Turmes L. Attachment disorders in the postpartum period. *Psychiatr Prax* 2010;**37**:310–11	1
429	van Bakel HJA, Riksen-Walraven JM. Parenting and development of one-year-olds: links with parental, contextual, and child characteristics. *Child Dev* 2002;**73**:256–73	2
430	van Bussel JC, Spitz B, Demyttenaere K. Reliability and validity of the Dutch version of the Maternal Antenatal Attachment Scale. *Arch Womens Ment Health* 2010;**13**:267–77	3
431	van Bussel JC, Spitz B, Demyttenaere K. Three self-report questionnaires of the early mother-to-infant bond: reliability and validity of the Dutch version of the MPAS, PBQ and MIBS. *Arch Womens Ment Health* 2010;**13**:373–84	3

Number	Reference	Reason
432	van der Mark IL, Bakermans-Kranenburg MJ, Van IJzendoorn MH. The role of parenting, attachment, and temperamental fearfulness in the prediction of compliance in toddler girls. *Br J Dev Psychol* 2002;**20**:361–78	4
433	Van IJzendoorn M, Tavecchio LWC, Goosens FA, Verger MM, Swaan J. How B is B4? Attachment and security of Dutch children in Ainsworth's Strange Situation and at home. *Psychol Rep* 1983;**52**:683–91	1
434	Van IJzendoorn MH, Bakermans-Kranenburg MJ, Juffer F. *Why Less Is More: From the Dodo Bird Verdict to Evidence-Based Interventions on Sensitivity and Early Attachments.* Berlin LJ, Ziv Y, Amaya-Jackson L, Greenberg T, editors. *Enhancing Early Attachments: Theory, Research, Intervention, and Policy.* New York, NY: Guilford Press; 2005. pp. 297–312	1
435	Van IJzendoorn MH, de Wolff MS. In search of the absent father – meta-analyses of infant-father attachment: a rejoinder to our discussants. *Child Dev* 1997;**68**:604–9	1
436	Van IJzendoorn MH, Kranenburg MJ, Zwart-Woudstra HA, Van Busschbach AM, *et al.* Parental attachment and children's socio-emotional development: some findings on the validity of the Adult Attachment Interview in the Netherlands. *Int J Behav Dev* 1991;**14**:375–94	3
437	Van IJzendoorn MH, Kroonenberg PM. Cross-cultural consistency of coding the strange situation. *Infant Behav Dev* 1990;**13**:469–85	1
438	Van IJzendoorn MH, M. KP. Cross-cultural patterns of attachment: a meta-analysis of the strange situation. *Child Dev* 1988;**59**:147–56	1
439	Van IJzendoorn MH, Rutgers AH, Bakermans-Kranenburg MJ, van Daalen E, Dietz C, Buitelaar JK, *et al.* Parental sensitivity and attachment in children with autism spectrum disorder: comparison with children with mental retardation, with language delays, and with typical development. *Child Dev* 2007;**78**:597–608	1
440	Van IJzendoorn MH. Adult attachment representations, parental responsiveness, and infant attachment: a meta-analysis on the predictive validity of the Adult Attachment Interview. *Psychol Bull* 1995;**117**:387–403	1
441	Van IJzendoorn, Hubbard F. Are infant crying and maternal responsiveness during the first year related to infant-mother attachment at 15 months? *Attach Hum Dev* 2000;**2**:371–91	2
442	van Oudenhoven JP, Hofstra J, Bakker W. Development and evaluation of the Attachment Styles Questionnaire (ASQ). *Nederlands Tijdschrift voor de Psychologie en haar Grensgebieden* 2003;**58**:95–102	5
443	Varra EM, Pearlman LA, Brock KJ, Hodgson ST. Factor analysis of the trauma and attachment belief scale: A measure of cognitive schema disruption related to traumatic stress. *J Psychol Trauma* 2008;**7**:185–96	1
444	Vereijken CM, Hanta S, Van Lieshout CF. Validity of attachment Q-sort descriptions by mothers: the Japanese case. *Jpn Psychol Res* 1997;**39**:291–9	1
445	Vereijken CMJL, Riksen-Walraven JM, Van Lieshout CFM. Mother-infant relationships in Japan: attachment, dependency, and amae. *J Cross Cult Psychol* 1997;**28**:442–62	1
446	Vizziello GF, Antonioli ME, Invernizzi R, Zancato P. Attachment and psychopathology during the first 3 years of life. *G Neuropsichiatr Evol* 1995;**15**:45–61	1
447	Wachter MPK. *Psychological Distress and Dyadic Satisfaction as Predictors of Maternal-Fetal Attachment*. PhD thesis. Illinois Institute Technology; 2002	3
448	Wagner PE. *Development of a Measure of Attachment for Children Ages 8 to 12.* PhD thesis. George Mason University; 1992	2
449	Walsh AP. *Representations of Attachment and Caregiving: The Disruptive Effects of Loss and Trauma*. PhD thesis. University of Virginia; 2003	3
450	Wan MW, Green J. Negative and atypical story content themes depicted by children with behaviour problems. *J Child Psychol Psychiatry* 2010;**51**:1125–31	1
451	Wartner UG. Attachment patterns at age six in south germany: predictability from infancy and implications for preschool behavior. *Child Dev* 1994;**65**:1014–27	1

© Queen's Printer and Controller of HMSO 2015. This work was produced by Wright *et al.* under the terms of a commissioning contract issued by the Secretary of State for Health. This issue may be freely reproduced for the purposes of private research and study and extracts (or indeed, the full report) may be included in professional journals provided that suitable acknowledgement is made and the reproduction is not associated with any form of advertising. Applications for commercial reproduction should be addressed to: NIHR Journals Library, National Institute for Health Research, Evaluation, Trials and Studies Coordinating Centre, Alpha House, University of Southampton Science Park, Southampton SO16 7NS, UK.

Number	Reference	Reason
452	Waters E, Deane KE. Defining and assessing individual differences in attachment relationships: Q-methodology and the organization of behavior in infancy and early childhood. In Bretherton I, Waters E, editors. *Growing Points of Attachment Theory and Research. Monogr Soc Res Child Dev*, 50 (1–2, Serial No. 209), 41–65	1
453	Waters E, Deane KE. Defining and assessing individual differences in attachment relationships: Q-methodology and the organization of behavior in infancy and early childhood. *Monogr Soc Res Child Dev* 1985;**50**:41–65	1
454	Waters E, Wippman J, Sroufe L. Attachment, positive affect, and competence in the peer group: two studies in construct validation. *Child Dev* 1979;**50**:821–9	3
455	Waters E. The reliability and stability of individual differences in infant-mother attachment. *Child Dev* 1978;**49**:483–94	1
456	Waters HS, Rodrigues LM, Ridgeway D. Cognitive underpinnings of narrative attachment assessment. *J Exp Child Psychol* 1998;**71**:211–34	1
457	Weinberg HA. Improved functioning in children diagnosed with reactive attachment disorder after SSRI therapy. *J Can Acad Child Adolesc Psychiatry* 2010;**19**:48–50	1
458	Wheeler DJ. *Fantasy Proneness and Attachment Style: A Mediation Model of the Development of Psychopathology.* PhD thesis. Fairfax, VA: George Mason University; 1999	3
459	White O, McCorry NK, Scott-Heyes G, Dempster M, Manderson J. Maternal appraisals of risk, coping and prenatal attachment among women hospitalised with pregnancy complications. *J Reprod Infant Psychol* 2008;**26**:74–85	3
460	Widera-Wysoczanska A. Complex childhood abuse, attachment disorders and health problems. *J Psychosom Res* 2004;**56**:670	1
461	Willcox E. Reactive attachment disorder in children. *Paediatr Nurs* 1995;**7**:14–16	1
462	Williams TM. *The Development of Maternal Attachment: A Longitudinal Study.* Report number ED172956. Biennial Meeting of the Society for Research in Child Development, San Francisco, CA, 1979	3
463	Wise S, Grossman FK. Adolescent mothers and their infants: psychological factors in early attachment and interaction. *Am J Orthopsychiatry* 1980;**50**:454–68	2
464	Wittkowski A, Williams J, Wieck A. An examination of the psychometric properties and factor structure of the Post-partum Bonding Questionnaire in a clinical inpatient sample. *Br J Clin Psychol* 2010;**49**:163–72	3
465	Wright JC, Binney V, Smith PK. Security of attachment in 8–12-year-olds: a revised version of the Separation Anxiety Test, its psychometric properties and clinical interpretation. *J Child Psychol Psychiatry* 1995;**36**:757–74	2
466	Youngblade LM, Park KA, Belsky J. Measurement of young children's close friendship: a comparison of two independent assessment systems and their associations with attachment security. *Int J Behav Dev* 1993;**16**:563–87	3
467	Yunger JL, Corby BC, Perry DG. Dimensions of attachment in middle childhood. In Kerns KA, Richardson RA, editors. *Attachment in Middle Childhood*. New York, NY: Guilford Press; 2005. pp. 89–114.	1
468	Zeanah CH, Scheeringa M, Boris NW, Heller SS, Smyke AT, Trapani J. Reactive attachment disorder in maltreated toddlers. *Child Abuse Negl* 2004;**28**:877–88	1
469	Zeanah CH, Smyke AT, Koga SFM, Carlson E & the BEIP Core Group. Attachment in institutionalized and non institutionalized Romanian Children. *Child Dev* 2005;**76**:1015–28	3
470	Zhang Y-L, Zhang Y-L, Zhang Y-X, Wang J-L, Hung C-Y. Reliability and validity of Chinese version of Revised Inventory of Parent and Peer Attachment in junior students. *Chin Ment Health J* 2011;**25**:66–70	1
471	Ziegenhain U, Jacobsen T. Assessing children's representational attachment models: Links to mother-child attachment quality in infancy and childhood. *J Genet Psychol* 1999;**160**:22–30	1

Supplementary systematic review 2 excluded papers with reasons

Reasons	Key
The study is not a longitudinal prospective cohort study of 10 years or more	1
The study does not contain relevant epidemiological data (prevalence or long-term outcome data)	2
The study does not attempt to use one of the approved 'gold standard' measurements of attachment	3
The study does not attempt to identify 'severe attachment problems' by capturing 'disorganised attachment style' AND/OR attachment disorder	4

Supplementary systematic review 2 excluded reference list

Number	Reference	Reason
1	Ahnert L, Lamb ME. Infant-care provider attachments in contrasting child care settings II. Individual-oriented care before German reunification. *Infant Behav Dev* 2000;**23**:211–22	1
2	Albus KE. *The Significance of Maternal Sensitivity in Infant Distress and Play Contexts.* PhD thesis. University of Delaware; 2001	1
3	Alink LR, Egeland B. The roles of antisocial history and emerging adulthood developmental adaptation in predicting adult antisocial behavior. *Aggressive Behav* 2013;**39**:131–40	2
4	Ammaniti M, Speranza AM. [Maternal representations and disorganized patterns of attachment in children.] *G Neuropsichiatr Evol* 1995;**15**:27–34	1
5	Anderson SE, Gooze RA, Lemeshow S, Whitaker RC. Quality of early maternal–child relationship and risk of adolescent obesity. *Pediatrics* 2012;**129**:132–40	3
6	Ang RP. Dysfunctional parenting behaviors and parenting stress among mothers of aggressive boys. *Child Fam Behav Ther* 2008;**30**:319–36	1
7	Arnoni GO. *A Comparison of Mother-Fetal Attachment in Medically Low-Risk and High-Risk Primagravidae Women.* PhD thesis. Indiana State University; 1990	1
8	Asendorpf JB, Wilpers S. Attachment security and available support: closely linked relationship qualities. *J Soc Pers Relat* 2000;**17**:115–38	1
9	Audet K, Le Mare L. Mitigating effects of the adoptive caregiving environment on inattention/ overactivity in children adopted from romanian orphanages. *Int J Behav Dev* 2011;**35**:107–15	2
10	Barber JS, Axinn WG, Thornton A. Unwanted childbearing, health, and mother–child relationships. *J Health Soc Behav* 1999;**40**:231–57	2
11	Barbiero C. [Child reading and attachment.] *Quaderni ACP* 2005;**12**:206–9	1
12	Bar-Haim Y, Aviezer O, Berson Y, Sagi A. Attachment in infancy and personal space regulation in early adolescence. *Attach Hum Dev* 2002;**4**:68–83	3
13	Bar-Haim Y, Dan O, Eshel Y, Sagi-Schwartz A. Predicting children's anxiety from early attachment relationships. *J Anxiety Dis* 2007;**21**:1061–8	3
14	Becker-Stoll F, Fremmer-Bombik E, Wartner U, Zimmermann P, Grossmann KE. Is attachment at ages 1, 6 and 16 related to autonomy and relatedness behavior of adolescents in interaction towards their mothers? *Int J Behav Dev* 2008;**32**:372–80	3
15	Beckwith L, Cohen SE, Hamilton CE. Maternal sensitivity during infancy and subsequent life events relate to attachment representation at early adulthood. *Dev Psychol* 1999;**35**:693–700	2

© Queen's Printer and Controller of HMSO 2015. This work was produced by Wright *et al.* under the terms of a commissioning contract issued by the Secretary of State for Health. This issue may be freely reproduced for the purposes of private research and study and extracts (or indeed, the full report) may be included in professional journals provided that suitable acknowledgement is made and the reproduction is not associated with any form of advertising. Applications for commercial reproduction should be addressed to: NIHR Journals Library, National Institute for Health Research, Evaluation, Trials and Studies Coordinating Centre, Alpha House, University of Southampton Science Park, Southampton SO16 7NS, UK.

Number	Reference	Reason
16	Beckwith L, Crawford S, Moore JA, Howard J. Attentional and Social Functioning of Preschool-Age Children Exposed to PCP and Cocaine in Utero. In Lewis M, Bendersky M, editors. *Mothers, Babies, and Cocaine: The Role of Toxins in Development.* Hillsdale, NJ, England: Lawrence Erlbaum Associates Inc.; 1995. pp. 287–303	1
17	Belsky J, Houts RM, Fearon R. Infant attachment security and the timing of puberty: testing an evolutionary hypothesis. *Psychol Sci* 2010;**21**:1195–201	3
18	Berlin LJ, Cassidy J, Belsky J. Loneliness in young-children and infant-mother attachment – a longitudinal-study. *Merrill-Palmer Q-J Dev Psychol* 1995;**41**:91–103	1
19	Bohlin G, Hagekull B. Socio-emotional development: from infancy to young adulthood. *Scand J Psychol* 2009;**50**:592–601	3
20	Bohlin G, Thorell L, Eninger L, Brocki C. Are attachment disorganization and inhibition independent predictors of symptoms of ADHD, externalizing problem behaviors and callous unemotional traits? European Network for Hyperkinetic Disorders Eunethydis 1st International ADHD Conference, 26–28 May 2010, Amsterdam, Netherlands: From Data to Best Clinical Practice. *Eur Child Adolesc Psychiatry* 2010;**19**	1
21	Bombardier CL. *Prenatal Exposure to Cocaine and Other Substances: Its Effect on Newborn Behavior and Subsequent Attachment Behaviour*. PhD thesis. University of Massachusetts; 1997	1
22	Borelli JL. *Emotional Reactivity and Children's Representational Models of Attachment Relationships.* PhD thesis. Yale University; 2008	1
23	Borkowski JG, Farris JR, Whitman TL, Carothers SS, Weed K, Keogh DA. *Risk and Resilience: Adolescent Mothers and their Children Grow Up.* Mahwah, NJ: Lawrence Erlbaum Associates Publishers; 2007	2
24	Bosquet MA. *An Examination of the Development of Anxiety Symptoms from a Developmental Psychopathology Perspective*. PhD thesis. University of Minnesota; 2001	4
25	Carlson KS. *Dismissing Attachment and Narcissism: Examining Two Constructs in Terms of Personality Similarities and Differences over a 20-Year Period*. PhD thesis. Santa Cruz, CA: University of California, Santa Cruz; 2002	2
26	Carlson V, Cicchetti D, Barnett D, Braunwald K. Disorganized/disoriented attachment relationships in maltreated infants. *Dev Psychol* 1989;**25**:525–31	1
27	Castle J, Groothues C, Beckett C, Colvert E, Hawkins A, Kreppner J, *et al.* Parents' evaluation of adoption success: a follow-up study of intercountry and domestic adoptions. *Am J Orthopsychiatry* 2009;**79**:522–31	2
28	Causadias JM, Salvatore JE, Sroufe LA. Early patterns of self-regulation as risk and promotive factors in development: a longitudinal study from childhood to adulthood in a high-risk sample. *Int J Behav Dev* 2012;**36**:293–302	2
29	Cherng CF. *Self and Interpersonal Perceptions in Relation to Child Development and Attachment: A Longitudinal Analysis of the Abused Child as a Mother*. PhD thesis. Newark, NJ: Rutgers University; 1999	1
30	Christopoulou C. *Familial Antecedents of Peer Social Competence in Second Graders.* PhD thesis. University of Virginia, 1989	1
31	Claussen AH, Mundy PC, Mallik SA, Willoughby JC. Joint attention and disorganized attachment status in infants at risk. *Dev Psychopathol* 2002;**14**:279–91	1
32	Coffino B. The role of childhood parent figure loss in the etiology of adult depression: findings from a prospective longitudinal study. *Attach Hum Dev* 2009;**11**:445–70	3
33	Connell DB. *Individual Differences in Attachment Behavior: Long Term Stability and Relationships to Language Development*. PhD thesis. Syracuse, NY: Syracuse University; 1978	1
34	Corriveau KH, Harris PL, Meins E, Fernyhough C, Arnott B, Elliott L, *et al.* Young children's trust in their mother's claims: longitudinal links with attachment security in infancy. *Child Dev* 2009;**80**:750–61	1
35	Cox M. *The Relationships between Episodes of Parental Incarceration and Students' Psycho-Social and Educational Outcomes: An Analysis of Risk Factors.* PhD thesis. Temple University; 2009	2
36	Crittenden PM. The effect of mandatory protective daycare on mutual attachment in maltreating mother-infant dyads. *Int J Child Abuse Negl* 1983;**7**:297–300	1

Number	Reference	Reason
37	Crockenberg SC, Leerkes EM. Parental acceptance, postpartum depression, and maternal sensitivity: mediating and moderating processes. *J Fam Psychol* 2003;**17**:80–93	1
38	Cyr C, Dubois-Comtois K, Moss E. [Mother-child conversations and the attachment of children in the pre-school period.] *Can J Behav Sci* 2008;**40**:140–52	1
39	Dallaire DH, Weinraub M. Predicting children's separation anxiety at age 6: the contributions of infant-mother attachment security, maternal sensitivity, and maternal separation anxiety. *Attach Hum Dev* 2005;**7**:393–408	1
40	Dan O, Sagi-Schwartz A, Bar-haim Y, Eshel Y. Effects of early relationships on children's perceived control: a longitudinal study. *Int J Behav Dev* 2011;**35**:449–56	3
41	Davidson CE. *The Intergenerational Transmission of Resilience and Risk: Maternal Depression, Maternal Adult Attachment Status, and the Competence of School-Aged Children*. PhD thesis. Tufts University; 1993	1
42	Declercq S, Nicolis H. La theorie de l attachement au secours des adolescents limites hospitalises. *Neuropsychiatrie de l Enfance et de l Adolescence* 2010;**58**:107–11	1
43	DeKlyen M, Speltz ML, Greenberg MT. Fathering and early onset conduct problems: Positive and negative parenting, father-son attachment, and the marital context. *Clin Child Fam Psychol Rev* 1998;**1**:3–21	1
44	DiGiaro DA. *Maternal Attachment History Variables as they Relate to Mother's Separation Concerns, Child's Attachment Patterns, and Child's Preschool Behaviour.* PhD thesis. California School of Professional Psychology; 1991	1
45	Dixon SD, LeVine RA, Brazelton T. Malnutrition: a closer look at the problem in an East African village. *Dev Med Child Neurol* 1982;**24**:670–85	1
46	Dugravier R. [Understanding consequences of hospitalization within attachment theory.] *Arch Pediatr* 2010;**17**:723–4	1
47	Dutra L, Bureau JF, Holmes B, Lyubchik A, Lyons-Ruth K. Quality of early care and childhood trauma: a prospective study of developmental pathways to dissociation. *J Nerv Ment Dis* 2009;**197**:383–90	1
48	Dutra L. *Quality of Early Care and Childhood Trauma: Developmental Pathways to Dissociation.* PhD thesis. Boston University; 2008	1
49	Easterbrooks M, Biesecker G, Lyons-Ruth K. Infancy predictors of emotional availability in middle childhood: the roles of attachment security and maternal depressive symptomatology. *Attach Hum Dev* 2000;**2**:170–87	1
50	Egeland B, Vaughn B. Failure of "bond formation" as a cause of abuse, neglect, and maltreatment. *Annual Progress Child Psychiatry Child Dev* 1982;**15**;188–98	1
51	Elmore GM. *The Role of School Satisfaction in Moderating and Mediating the Relationship between Attachment Relationships and Negative School Behavior in Adolescents.* PhD thesis. University of South Carolina; 2007	1
52	Else-Quest NM, Hyde JS, Clark R. Breastfeeding, bonding, and the mother–infant relationship. *Merrill-Palmer Q* 2003;**49**:495–517	1
53	Englund MM, Kuo SIC, Puig J, Collins WA. Early roots of adult competence: the significance of close relationships from infancy to early adulthood. *Int J Behav Dev* 2011;**35**:490–6	3
54	Englund MM, Siebenbruner J. Developmental pathways linking externalizing symptoms, internalizing symptoms, and academic competence to adolescent substance use. *J Adolesc* 2012;**35**:1123–40	2
55	Englund MML, A. K., Hyson DM, Sroufe LA. Adolescent social competence: effectiveness in a group setting. *Child Dev* 2000;**71**:1049–60	3
56	Erez T. Predicting Preschoolers' Social Coping from Early Attachment History. In Shulman S, editor. *Close Relationships and Socioemotional Development*. Westport, CT: Ablex Publishing; 1995. pp. 1–34	1
57	Feldman R. The rational basis of adolescent adjustment: trajectories of mother-child interactive behaviors from infancy to adolescence shape adolescents' adaptation. *Attach Hum Dev* 2010;**12**:173–92	2
58	Fergusson DM, Lynskey MT, Horwood LJ. Comorbidity between depressive disorders and nicotine dependence in a cohort of 16-year-olds. *Arch Gen Psychiatry* 1996;**53**:1043–7	2

© Queen's Printer and Controller of HMSO 2015. This work was produced by Wright *et al.* under the terms of a commissioning contract issued by the Secretary of State for Health. This issue may be freely reproduced for the purposes of private research and study and extracts (or indeed, the full report) may be included in professional journals provided that suitable acknowledgement is made and the reproduction is not associated with any form of advertising. Applications for commercial reproduction should be addressed to: NIHR Journals Library, National Institute for Health Research, Evaluation, Trials and Studies Coordinating Centre, Alpha House, University of Southampton Science Park, Southampton SO16 7NS, UK.

Number	Reference	Reason
59	Fernandez E. How children experience fostering outcomes: participatory research with children. *Child Fam Social Work* 2007;**12**:349–59	1
60	Foley D, Rutter M, Pickles A, Angold A, Maes H, Silberg J, *et al.* Informant disagreement for separation anxiety disorder. *J Am Acad Child Adolesc Psychiatry* 2004;**43**:452–60	1
61	Fonagy P, Steele H, Steele M. A prospective longitudinal-study of the influence of adult attachment patterns on infant attachment and child-development. *Int J Psychol* 1992;**27**:209	1
62	Fonagy P, Steele M, Steele H, Leigh T, Kennedy R, Mattoon G, *et al.* Attachment, the Reflective Self, and Borderline States: The Predictive Specificity of the Adult Attachment Interview and Pathological Emotional Development. In Goldberg S, Muir R, Kerr J, editors. *Attachment Theory: Social, Developmental, and Clinical Perspectives.* Hillsdale, NJ: Analytic Press Inc.; 1995. pp. 233–78	1
63	Fonseca V, da Silva GA, Otta E. The relationship between postpartum depression and maternal emotional availability. *Cad Saude Publica* 2010;**26**:738–46	1
64	Fothergill KE, Ensminger ME. Childhood and adolescent antecedents of drug and alcohol problems: a longitudinal study. *Drug Alcohol Depend* 2006;**82**:61–76	2
65	Freitag MK, Belsky J, Grossmann K, Grossmann KE, *et al.* Continuity in parent–child relationships from infancy to middle childhood and relations with friendship competence. *Child Dev* 1996;**67**:1437–54	1
66	Freitag MK. *Continuity in Parent–Child Relationships from Infancy to Middle Childhood and Relations with Friendship Competence.* PhD thesis. Pennsylvania State University; 1995	3
67	Gaffney M, Greene SM, Wieczorek-Deering D, Nugent J. The concordance between mother-infant attachment at 18 months and maternal attachment 10 years later among married and single mothers. *Ir J Psychol* 2000;**21**:154–70	1
68	Gaik LP, Abdullah MC, Elias H, Uli J. Development of antisocial behaviour. International Conference on Learner Diversity. *Procedia Soc Behav Sci* 2010;7:383–8	1
69	Gainey RR, Catalano RF, Haggerty KP, Hoppe MJ. Deviance among the children of heroin addicts in treatment: impact of parents and peers. *Deviant Behav* 1997;**18**:143–59	1
70	Gao Y, Raine A, Chan F, Venables PH, Mednick SA. Early maternal and paternal bonding, childhood physical abuse and adult psychopathic personality. *Psychol Med* 2010;**40**:1007–16	2
71	Geddes H. *Attachment in the Classroom. The Links between Children's Early Experience, Emotional Well-Being and Performance in School*. London: Worth Publishing Limited; 2006	1
72	George CY. *Individual Differences in Affective Sensitivity: A Study of Five-Year-Olds and Their Parents*. PhD thesis. University of California; 1985	1
73	Gervai J, Novak A, Lakatos K, Toth I, Danis I, Ronai Z, *et al.* Infant genotype may moderate sensitivity to maternal affective communications: attachment disorganization, quality of care, and the DRD4 polymorphism. *Soc Neurosci* 2007;**2**:307–19	1
74	Gilbride KE. *Adolescent Maternal Behavior: Assessment over Time and its Relationship to Infant-Mother Attachment*. PhD thesis. University of Utah; 1989	1
75	Goldfarb W. The effects of early institutional care on adolescent personality. *J Exp Educ* 1944;**12**:106–29	2
76	Goossens FA, Van IJzendoorn MH. Quality of infants' attachments to professional caregivers: Relation to infant-parent attachment and day-care characteristics. *Child Dev* 1990;**61**:832–7	1
77	Grossmann K, Grossmann KE, Fremmer-Bombik E, Kindler H, Scheuerer-Englisch H, Zimmermann P. The uniqueness of the child-father attachment relationship: Fathers' sensitive and challenging play as a pivotal variable in a 16-year longitudinal study. *Soc Dev* 2002;**11**:307–31	3
78	Grossmann K, Grossmann KE. Newborn Behavior, the Quality of Early Parenting and Later Toddler–Parent Relationships in a Group of German Infants. In Nugent JK, Lester BM, Brazelton B, editors. *The Cultural Context of Infancy, Vol 2: Multicultural and Interdisciplinary Approaches to Parent–Infant Relations.* Westport, CT: Ablex Publishing; 1991. pp. 3–38	1
79	Guedeney A. Kwashiorkor, depression, and attachment disorders. *Lancet* 1995;**346**:293	1
80	Guedeney N, Lamas C, Bekhechi V, Mintz AS, Guedeney A. Developpement du processusd'attachement entre un bebe et sa mere. *Arch Pediatr* 2008;**15**(Suppl. 1):12–S9	1

Number	Reference	Reason
81	Hamilton CE. Continuity and discontinuity of attachment from infancy through adolescence. *Child Dev* 2000;**71**:690–4	3
82	Harris SJ. *A Longitudinal Investigation of Attachment and Emotion Regulation: Links with Internalizing and Externalizing Symptoms among Children of Mothers with Mood Spectrum Disorders in Comparison with Children of Well Mothers.* PhD thesis. The Catholic University of America; 2008	2
83	Harrison L, Ungerer JA. Child care predictors of infant-mother attachment security at age 12 months. *Early Child Dev Care* 1997;**137**:31–46	1
84	Haydon KC. *Pathways to Generalized and Partner-Specific Attachment Representations in Adulthood: A Developmental Perspective on the Organization of Romantic Behavior*. PhD thesis. University of Minnesota; 2009	3
85	Heckler SM. *The Effects of Siblings on Attachment Formation in Foster Children*. PhD thesis. Regent University; 2011	1
86	Heywood CV. *Predictors of Favorable Outcomes among Children in Foster Care: An Analysis of Early Childhood Variables and their Relationship to Tthe Development of Assets.* PhD thesis. University of Oregon; 2010	1
87	Hodges J, Tizard B. IQ and behavioural adjustment of ex-institutional adolescents. *J Child Psychol Psychiatry* 1989;**30**:53–75	2
88	Hodges J, Tizard B. Social and family relationships of ex-institutional adolescents. *J Child Psychol Psychiatry* 1989;**30**:77–97	2
89	Hodges J. The Natural History of Early Non-Attachment. In Bernstein B, Brannen J, editors. *Children, Research and Policy: Essays for Barbara Tizard.* Philadelphia: Taylor & Francis; 1996. pp. 63–80	2
90	Hoevet GL. *The Relationship of a Facial Defect and Separation History with Security, Attachment Behavior, Exploration, and Object Permanence in the One Year Old Infant.* PhD thesis. University of California; 1983	1
91	Holmes BM, Lyons-Ruth K. The relationship questionnaire-clinical version (RQ-CV): introducing a profoundly-distrustful attachment style. *Infant Ment Health J* 2006;**27**:310–25	2
92	Honig AS. Infant Care and Dual Career Parents: Ideas and Tips. In Gielen UP, Comunian AL, editors. *The Family and Family Therapy in International Perspective*. Trieste: Edizioni Lint Trieste; 1998. pp. 386–400	1
93	Honig AS. Research in review: risk factors in infants and young children. *Young Child* 1984;**39**:60–73	1
94	Horvath DL. *Parenting over Time: A Developmental Perspective.* PhD thesis. Temple University; 2004	1
95	Howes P, Markman HJ. Marital quality and child functioning: a longitudinal investigation. *Child Dev* 1989;**60**:1044–51	1
96	Hubbstait L, Osofsky JD, Hann DM, Culp AM. Predicting behavior problems and social competence in children of adolescent mothers. *Fam Relat* 1994;**43**:439–46	1
97	Hurst JR. *The Development of Adolescent Autonomy: Contributions of the Mother-Child Attachment Relationship and Maternal Sensitivity*. PhD thesis. Renee: University of Texas at Dallas; 2011	4
98	Ingram JR, Patchin JW, Huebner BM, McCluskey JD, Bynum TS. Parents, friends, and serious delinquency: an examination of direct and indirect effects among at-risk early adolescents. *Criminal Justice Rev* 2007;**32**:380–400	1
99	Ivan Z, Bereczkei T. Parental bonding, risk-taking behavior and life history theory. *J Cult Evol Psychol* 2006;**4**:267–75	1
100	Iwaniec D, Sneddon H. Attachment style in adults who failed to thrive as children: outcomes of a 20 year follow-up study of factors influencing maintenance or change in attachment style. *Br J Soc Work* 2001;**31**:179–95	3
101	Iwaniec D. *Children who Fail to Thrive: A Practice Guide.* Chichester: Wiley; 2004	1
102	Jacobsen T, Edelstein W, Hofmann V. A longitudinal study of the relation between representations of attachment in childhood and cognitive functioning in childhood and adolescence. *Dev Psychol* 1994;**30**:112–24	2

© Queen's Printer and Controller of HMSO 2015. This work was produced by Wright *et al.* under the terms of a commissioning contract issued by the Secretary of State for Health. This issue may be freely reproduced for the purposes of private research and study and extracts (or indeed, the full report) may be included in professional journals provided that suitable acknowledgement is made and the reproduction is not associated with any form of advertising. Applications for commercial reproduction should be addressed to: NIHR Journals Library, National Institute for Health Research, Evaluation, Trials and Studies Coordinating Centre, Alpha House, University of Southampton Science Park, Southampton SO16 7NS, UK.

Number	Reference	Reason
103	Jimerson SR, Egeland B, Sroufe L, Carlson B. A prospective longitudinal study of high school dropouts: examining multiple predictors across development. *J Sch Psychol* 2000;**38**:525–49	3
104	Jones KA, Benda BB. Alcohol use among adolescents with non-residential fathers: a study of assets and deficits. *Alcohol Treat Q* 2004;**22**:3–25	2
105	Jones ME. *An Anthropological Assessment of Bonding: Mother/Infant Attachment Behavior in a Study of Forty Adolescent Black and White Mothers from Delivery through Six Months*. PhD thesis. Southern Methodist University; 1981	1
106	Kadir NBA. Attachment style in relation to depressive-risk among single and married Malaysian mothers. *Int J Psychol* 2008;**43**:566	1
107	Keller TE, Spieker SJ, Gilchrist L. Patterns of risk and trajectories of preschool problem behaviors: a person-oriented analysis of attachment in context. *Dev Psychopathol* 2005;**17**:349–84	1
108	Kelly KP. *Ongoing Substance Abuse and its Effect on the Attachment Quality of Toddlers*. PhD thesis. Fordham University; 2003	1
109	Kerns KA, Siener S, Brumariu LE. Mother–child relationships, family context, and child characteristics as predictors of anxiety symptoms in middle childhood. *Dev Psychopathol* 2011;**23**:593–604	2
110	Kidwell SL. *Attachment and Vagal Tone as Predictors of Self-Regulation among Low-Income, African-American Children: A Multivariate, Longitudinal Approach*. PhD thesis. Wayne State University; 2001	1
111	Kim S. *The Effects of Parent Bonding, School Bonding, Belief on the Structure of Problem Behaviors in Elementary School-Age Children*. PhD thesis. University of Washington; 2000	1
112	Kwako LE, Noll JG, Putnam FW, Trickett PK. Childhood sexual abuse and attachment: an intergenerational perspective. *Clin Child Psychol Psychiatry* 2010;**15**:407–22	1
113	Lakatos K, Toth I, Ney K, Gervai J, Nemoda Z, Ronai Z, *et al.* Genetic risk for attachment disorganisation in infancy: the role of the D4 dopamine receptor (DRD4). *J Reprod Infant Psychol* 2001;**19**:269	1
114	Lee KS, Jeong SJ, Shin YJ. A comparative study of reactive attachment disorder with autistic child in social cognition – focused on social referencing and joint attention. *Korean J Clin Psychol* 2000;**19**:793–806	1
115	Leschied AW, Chiodo D, Whitehead PC, Hurley D. The relationship between maternal depression and child outcomes in a child welfare sample: implications for treatment and policy. *Child Fam Social Work* 2005;**10**:281–91	1
116	Lewis M, Feiring C, Rosenthal S. Attachment over time. *Child Dev* 2000;**71**:707–20	3
117	Lewis M. Contextualism and the issue of continuity. *Infant Behav Dev* 1999;**22**:431–44	1
118	Lichtenberg JD. Procreation – a discussion. *Psychoanalytic Inquiry* 2011;**31**:430–3	2
119	Lieberman AF. Preschoolers' competence with a peer: relations with attachment and peer experience. *Child Dev* 1977;**48**:1277–87	1
120	Lieberman M, Doyle AB, Markiewicz D. Developmental patterns in security of attachment to mother and father in late childhood and early adolescence: associations with peer relations. *Child Dev* 1999;**70**:202–13	1
121	Lis S. *Characteristics of Attachment Behavior in Institution-Reared Children. The Organization of Attachment Relationships: Maturation, Culture, and Context.* New York, NY: Cambridge University Press; 2000. pp. 141–70	1
122	Lorber MF, Egeland B. Infancy parenting and externalizing psychopathology from childhood through adulthood: developmental trends. *Dev Psychol* 2009;**45**:909–12	2
123	Lorber MF, Egeland B. Parenting and infant difficulty: testing a mutual exacerbation hypothesis to predict early onset conduct problems. *Child Dev* 2011;**82**:2006–20	1
124	Lyons-Ruth K, Connell DB, Zoll D. Patterns of Maternal Behavior among Infants at Risk for Abuse: Relations with Infant Attachment Behavior and Infant Development at 12 Months of Age. In Cicchetti D, Carlson V, editors. *Child Maltreatment: Theory and Research on the Causes and Consequences of Child Abuse and Neglect.* New York, NY: Cambridge University Press; 1989. pp. 464–93	1

Number	Reference	Reason
125	Lyons-Ruth K, Dutra L, Schuder MR, Bianchi I. From infant attachment disorganization to adult dissociation: relational adaptations or traumatic experiences? *Psychiatric Clinics N Am* 2006;**29**:63–86	1
126	Lyons-Ruth K. Dissociation and the parent-infant dialogue: a longitudinal perspective from attachment research. *J Am Psychoanalytic Assoc* 2003;**51**:883–911	1
127	MacDonald HZ, Beeghly M, Knight WG, Woods R, Cabral H, Jacobs RR, *et al.* Longitudinal relation between disorganized infant attachment and childhood posttraumatic stress symptoms. *Pediatr Res* 2004;**55**:80A–A	1
128	Main M, Hesse, E., & Kaplan, N. Predictability of Attachment Behavior and Representational Processes at 1, 6, and 19 Years of Age. In Grossman KE, Grossman K, Waters E, editors. *Attachment from Infancy to Adulthood: The Major Longitudinal Studies.* New York, NY: Guilford Publications, Inc.; 2005. pp. 245–304	4
129	Main M. Cross-cultural studies of attachment organization: recent studies, changing methodologies, and the concept of conditional strategies. *Hum Dev* 1990;**33**:787–95	1
130	Mallery CJ. *The Effects of Neighborhood Context and Parenting Factors on Adolescent Mental Health: A Multilevel Investigation*. PhD thesis. The George Washington University; 2011	1
131	Marchand JF, Schedler S, Wagstaff DA. The role of parents' attachment orientations, depressive symptoms, and conflict behaviors in children's externalizing and internalizing behavior problems. *Early Child Res Q* 2004;**19**:449–62	1
132	McBride S, Belsky J. Characteristics, determinants, and consequences of maternal separation anxiety. *Dev Psychol* 1988;**24**:407–14	1
133	McCrone ER. *Attachment and History with Mother as Predictors of Middle-Childhood Representations of Relationships Assessed with Projective Storytelling*. PhD thesis. University of Minnesota; 1995	3
134	McLaren L, Kuh D, Hardy R, Mishra G. Postnatal depression and the original mother–child relationship: a prospective cohort study. *J Affect Dis* 2007;**100**:211–19	2
135	McLeod SK. *Maternal Family History Correlates of Disorganized Attachment in 18-Month-Old High-Risk Infants.* PhD thesis. Boston College; 1989	1
136	Miller L, Warner V, Wickramaratne P, Weissman M. Self-esteem and depression: ten year follow-up of mothers and offspring. *J Affect Dis* 1999;**52**:41–9	2
137	Mintz TM, Hamre BK, Hatfield BE. The role of effortful control in mediating the association between maternal sensitivity and children's social and relational competence and problems in first grade. *Early Educ Dev* 2011;**22**:360–87	1
138	Mizukami K, Kobayashi N, Iwata H, Ishii T. Telethermography in Measurement of Infants' Early Attachment. In von Euler C, Zetterstrom R. *Neurobiology of Early Infant Behaviour: Wenner-Gren Center International Symposium Series.* New York, NY: Stockton Press; 1989. pp. 249–59	1
139	Moore GA, Cohn JF, Belsky J, Campbell SB. A comparison of traditional and quantitative classification of attachment status. *Infant Behav Dev* 1996;**19**:265–8	1
140	Moran G, Forbes L, Evans E, Tarabulsy GM, Madigan S. Both maternal sensitivity and atypical maternal behavior independently predict attachment security and disorganization in adolescent mother–infant relationships. *Infant Behav Dev* 2008;**31**:321–5	1
141	Murphy E, Wickramaratne P, Weissman M. The stability of parental bonding reports: a 20-year follow-up. *J Affect Dis* 2010;**125**:307–15	2
142	Murray J. *The Relationship of Attachment and Coping Strategy Use to Later Adjustment to Starting Secondary School.* PhD thesis. University of Wales; 2000	1
143	Murray L, Arteche A, Fearon P, Halligan S, Goodyer I, Cooper P. Maternal postnatal depression and the development of depression in offspring up to 16 years of age. *J Am Acad Child Adolesc Psychiatry* 2011;**50**:460–70	3
144	Murray L, Cooper PJ, editors. Clinical Applications of Attachment Theory and Research: Change in Infant Attachment with Brief Psychotherapy. In Richer J, editor. *The Clinical Application of Attachment Theory*. Oxford: ACPP; 1994	1

© Queen's Printer and Controller of HMSO 2015. This work was produced by Wright *et al.* under the terms of a commissioning contract issued by the Secretary of State for Health. This issue may be freely reproduced for the purposes of private research and study and extracts (or indeed, the full report) may be included in professional journals provided that suitable acknowledgement is made and the reproduction is not associated with any form of advertising. Applications for commercial reproduction should be addressed to: NIHR Journals Library, National Institute for Health Research, Evaluation, Trials and Studies Coordinating Centre, Alpha House, University of Southampton Science Park, Southampton SO16 7NS, UK.

Number	Reference	Reason
145	Murray L, Cooper PJ. The impact of postpartum depression on child development. *Int Rev Psych* 1996;**8**:55–63	1
146	Murray L, Halligan SL, Adams G, Patterson P, Goodyer IM. Socioemotional development in adolescents at risk for depression: the role of maternal depression and attachment style. *Dev Psychopathol* 2006;**18**:489–516	3
147	Murray MP. *Attachment Relationships and Empathic Response in Young People who Sexually Abuse.* PhD thesis. Queen's University Belfast; 2002	1
148	NICHD Early Child Care Research Network. The effects of infant child care on infant-mother attachment security: results of the NICHD Study of Early Child Care. *Child Dev* 1997;**68**:860–79	1
149	Niederhofer H. [Long term effects of prepartal mother-child-attachment.] *Z Geburtshilfe Neonatol* 2007;**211**:82–6	1
150	O'Connor E, McCartney K. Attachment and cognitive skills: an investigation of mediating mechanisms. *J Appl Dev Psychol* 2007;**28**:458–76	1
151	O'Connor TG, Glover V, Bergman K, Sarkar P. Maternal stress/anxiety on infant stress physiology and development: contrasting programming and plasticity models. *Biol Psychiatry* 2009;**65**(Suppl.):65S	1
152	Padron E. *The Disorganized Dyad: The Roles of Mother and Child in the Development of Disorganized Attachment.* PhD thesis. University of Minnesota; 2004	1
153	Paivastegar M. Predicting risk factors of attachment disorders in school age children with respect to parents attachment styles. *Int J Psychol* 2008;**43**:509	1
154	Palacios J, Roman M, Moreno C, Leon E. Family context for emotional recovery in internationally adopted children. *Int Social Work* 2009;**52**:609–20	1
155	Parisi D, Cecconi F, Cerini A, editors. Kin-Directed Altruism and Attachment Behaviour in an Evolving Population of Neural Networks. In Gilbert N, Conte R, editor. *Artificial Societies: Computer Simulation of Social Life*. Siena: UCL Press, 1995	1
156	Passman RH, Adams RE. Learning-theory and infantile attachment – re-evaluation. *Behav Brain Sci* 1978;**1**:454–5	1
157	Patino FL. *Mothers' Prenatal Attachment History, Attachment Status, and Social Support Satisfaction as Predictors of Postnatal Maternal Sensitivity*. PhD thesis. California School of Professional Psychology, San Diego; 1993	1
158	Pauli-Pott U, Schneider A. Early communication within mother–infant interaction and development in infancy and early childhood. *Fruhforderung Interdisziplinar* 2006;**25**:26–36	1
159	Pierrehumbert B, Muntean A, Tomita M, Ungureanu R, Habersaat S. The significant behavioral aspects of adopted children within successful adoption in Romania. *Rev Cercet Interv Soc* 2011;**32**:107–30	2
160	Powell JL. *Linkages between Childhood Experiences of Family Violence and Intimacy in Close Relationships during Childhood and Adolescence*. PhD thesis. University of Minnesota, 2001	2
161	Powers. *Cognitive Development, Temperament, Attachment, and Maternal Sensitivity as Factors in Emotion Regulation*. PhD thesis. University of Notre Dame; 1999	1
162	Proctor CD. *Behavior Indicators as Attachment Difficulties in Children Adopted from Romania*. PhD thesis. Case Western Reserve University; 2007	1
163	Puig J, Englund MM, Simpson JA, Collins WA. Predicting adult physical illness from infant attachment: a prospective longitudinal study. *Health Psychol* 2012;**32**:409–17	3
164	Radke-Yarrow M. *Attachment Patterns in Children of Depressed Mothers. Attachment across the Life Cycle*. New York, NY: Tavistock/Routledge; 1991	1
165	Reid JA, Sullivan CJ. A model of vulnerability for adult sexual victimization: the impact of attachment, child maltreatment, and scarred sexuality. *Violence Victims* 2009;**24**:485–501	2
166	Rochford LG. *Attending to Attachment: The Relation between Attention-Deficit/Hyperactivity Disorder and Mother-Child Attachment in Early Childhood.* PhD thesis. University of North Carolina; 2005	1
167	Roisman GI, Padron E, Sroufe L, Egeland B. Earned-secure attachment status in retrospect and prospect. *Child Dev* 2002;**73**:1204–19	3

Number	Reference	Reason
168	Rosenblum O, Benony H, Mazet P. [Psycho-affective infant development and maternal depression.] *Eur Rev Appl Psychol* 1997;**47**:231–6	1
169	Rutter M, Colvert E, Kreppner J, Beckett C, Castle J, Groothues C, *et al.* Early adolescent outcomes for institutionally-deprived and non-deprived adoptees. I: Disinhibited attachment. *J Child Psychol Psychiatry* 2007;**48**:17–30	1
170	Sakin JW. *Maternal and Child Correlates of Attachment Quality among Toddler.* PhD thesis. University of Maryland Baltimore County; 1997	1
171	Salo J, Jokela M, Lehtimaki T, Keltikangas-Jarvinen L. Serotonin receptor 2A gene moderates the effect of childhood maternal nurturance on adulthood social attachment. *Genes Brain Behav* 2011;**10**:702–9	2
172	Salo J, Jokela M, Merjonen P, Lehtimaki T, Keltikangas-Jarvinen L. Serotonin receptor 2A gene moderates the effect of childhood nurturing environment on social attachment in adulthood. Twin Research and Human Genetics Conference: 13th International Congress on Twin Studies Seoul, South Korea, 2010	2
173	Sampson MC. *Continuity and Change in Patterns of Attachment between Infancy, Adolescence, and Early Adulthood in a High Risk Sample*. PhD thesis. University of Minnesota; 2006	4
174	Schactman A. *Emotion Regulation and Behaviour Problems in Young Children Exposed to Domestic Violence*. PhD thesis. University of Saskatchewan; 2010	1
175	Scher A, Mayseless O. Mothers of anxious/ambivalent infants: maternal characteristics and child-care context. *Child Dev* 2000;**71**:1629–39	1
176	Schneider-Rosen K. Current perspectives in attachment theory: illustration from the study of maltreated infants. *Monographs Society Res Child Dev* 1985;**50**:194–210	1
177	Shaffer A, Yates TM, Egeland B. The relation of emotional maltreatment to early adolescent competence: develpmental processes in a prospective study. *Child Abuse Negl* 2009;**33**:36–44	2
178	Shechory M, Sommerfeld E. Attachment style, home-leaving age and behavioral problems among residential care children. *Child Psychiatry Hum Dev* 2007;**37**:361–73	1
179	Shulman S, Elicker J, Sroufe L. Stages of friendship growth in preadolescence as related to attachment history. *J Soc Pers Relat* 1994;**11**:341–61	1
180	Silva EM. *Disorganized/Disoriented Attachment and Maternal Behavior in Twelve-Month-Old High-Risk Infants:* PhD thesis. Boston University; 1990	1
181	Simpson JA, Collins WA, Tran S, Haydon KC. Attachment and the experience and expression of emotions in romantic relationships: a developmental perspective. *J Personality Soc Psychol* 2007;**92**:355–67	3
182	Simpson JA, Rholes WS. Attachment, perceived support, and the transition to parenthood: social policy and health implications. *Soc Issues Policy Rev* 2008;**2**:37–63	1
183	Simpson JA, Steven Rholes W, Campbell L, Wilson CL. Changes in attachment orientations across the transition to parenthood. *J Exp Soc Psychol* 2003;**39**:317–31	1
184	Slade A. Quality of attachment and early symbolic play. *Dev Psychol* 1987;**23**:78–85	1
185	Smith PB. *Infant-Mother Attachment as a Function of Maternal Sensitivity, Cognition, Personality, and Social Support.* PhD thesis. University of Western Ontario; 1988	1
186	Sousa C, Herrenkohl TI, Moylan CA, Tajima EA, Klika JB, Herrenkohl RC, *et al.* Longitudinal study on the effects of child abuse and children's exposure to domestic violence, parent–child attachments, and antisocial behavior in adolescence. *J Interpers Violence* 2011;**26**:111–36	1
187	Spangler G, Johann M, Ronai Z, Zimmermann P. Genetic and environmental influence on attachment disorganization. *J Child Psychol Psychiatry* 2009;**50**:952–61	1
188	Sroufe L, Duggal S, Weinfield N, Carlson E. Relationships Development and Psychopathology. In Sameroff AJ, Lewis M, Miller SM, editors. *Handbook of Developmental Psychopathology*. New York, NY: Springer Science and Business Media; 2000	1
189	Sroufe L. Attachment and development: a prospective, longitudinal study from birth to adulthood. *Attach Hum Dev* 2005;b:349–67	1

© Queen's Printer and Controller of HMSO 2015. This work was produced by Wright *et al.* under the terms of a commissioning contract issued by the Secretary of State for Health. This issue may be freely reproduced for the purposes of private research and study and extracts (or indeed, the full report) may be included in professional journals provided that suitable acknowledgement is made and the reproduction is not associated with any form of advertising. Applications for commercial reproduction should be addressed to: NIHR Journals Library, National Institute for Health Research, Evaluation, Trials and Studies Coordinating Centre, Alpha House, University of Southampton Science Park, Southampton SO16 7NS, UK.

Number	Reference	Reason
190	Sroufe LA, Egeland B, Kreutzer T. The fate of early experience following developmental change: longitudinal approaches to individual adaptation in childhood. *Child Dev* 1990;**61**:1363–73	3
191	Sroufe LA. Placing Early Attachment Experiences in Developmental Context. In Grossman KE, Grossman K, Waters E, editors. *Attachment from Infancy to Adulthood: The Major Longitudinal Studies.* New York, NY: Guilford Publications, Inc.; 2005	1
192	Stauffer S. Trauma and disorganized attachment in refugee children: integrating theories and exploring treatment options. *Refugee Survey Q* 2008;**27**:150–63	1
193	Strijker J, Knorth EJ, Knot-Dickscheit J. Placement history of foster children: a study of placement history and outcomes in long term family foster care. *Child Welfare* 2008;**87**:107–24	1
194	Suess GJ, Sroufe J. Clinical implications of the development of the person. *Attach Hum Dev* 2005;**7**:381–92	3
195	Svejda MJ, Campos JJ, Emde RN. Mother infant bonding: failure to generalize. *Child Dev* 1980;**51**:775–9	1
196	Sypeck MF. *Attachment Style and Childhood Sexual Abuse: A Longitudinal Examination*. PhD thesis. American University; 2005	1
197	Tilbrook DMW. *Attachment, Conduct Disorder and Perspective Taking in 7–9 Year Old Boys.* PhD thesis. Open University; 2000	1
198	Toth SL, Cicchetti D. The impact of relatedness with mother on school functioning in maltreated children. *J Sch Psychol* 1996;**34**:247–66	1
199	Turton P, Hughes P, Fonagy P, Fainman D. An investigation into the possible overlap between PTSD and unresolved responses following stillbirth: an absence of linkage with only unresolved status predicting infant disorganization. *Attach Hum Dev* 2004;**6**:241–53	1
200	Urban J, Carlson E, Egeland B, Sroufe L. Patterns of individual adaptation across childhood. *Dev Psychopathol* 1991;**3**:445–60	1
201	Van Dam M, Van IJzendoorn M. Do working mothers have insecure attachment relationships with their infants? A secondary analysis of the relation between maternal employment and the quality of infant-mother attachment. *Kind en Adolescent* 1990;**11**:71–80	1
202	Van Der Zee KI, Ali AJ, Haaksma I. Determinants of effective coping with cultural transition among expatriate children and adolescents. *Anxiety Stress Coping* 2007;**20**:25–45	1
203	Verschueren K, Marcoen A. Representation of self and socioemotional competence in kindergartners: differential and combined effects of attachment to mother and father. *Child Dev* 1999;**70**:183–201	1
204	Warren SL, Huston L, Egeland B, Sroufe L. Child and adolescent anxiety disorders and early attachment. *J Am Acad Child Adolesc Psychiatry* 1997;**36**:637–44	3
205	Waters E, Hamilton CE, Weinfield NS. The stability of attachment security from infancy to adolescence and early adulthood: general introduction. *Child Dev* 2000;**71**:678–83	3
206	Waters E, Merrick S, Treboux D, Crowell J, Albersheim L. Attachment security in infancy and early adulthood: a twenty-year longitudinal study. *Child Dev* 2000;**71**:684–9	3
207	Weinfield NS, Sroufe L, Egeland B. Attachment from infancy to early adulthood in a high-risk sample: continuity, discontinuity, and their correlates. *Child Dev* 2000;**71**:695–702	3
208	Weinfield NS, Whaley GJ, Egeland B. Continuity, discontinuity, and coherence in attachment from infancy to late adolescence: sequelae of organization and disorganization. *Attach Hum Dev* 2004;**6**:73–97	4
209	Willcox E. Reactive attachment disorder in children. *Paediatr Nurs* 1995;**7**:14–16	1
210	Woodward L, Fergusson DM, Belsky J. Timing of parental separation and attachment to parents in adolescence: results of a prospective study from birth to age 16. *J Marriage Fam* 2000;**62**:162–74	2
211	Yumoto C. *Attachment Representation in Inner-City African American Adolescents*. PhD thesis. Wayne State University; 2008	1
212	Zimmermann P, Becker-Stoll F, Grossmann K, Grossmann KE, Scheuerer-Englisch H, Wartner U. [Longitudinal attachment development from infancy through adolescence.] *Psychol Erz Unterr* 2000;**47**:99–117	3

Main systematic review excluded papers with reasons

Reason	Key
Does the study focus on an intervention for parents/caregivers?	1
Does the study evaluate the intervention with a measure of attachment?	2
Does the study have pre and post outcome measures for a population of children recruited into the study under the age of 13 years?	3A
Does the study have post outcome measures of attachment for children recruited into the study at the age of 1 year and under?	3B
Is the study described as a randomised controlled trial?	4

Main systematic review reference list

Number	Reference	Reason
1	Abela JR, Hankin BL, Haigh EA, Adams P, Vinokuroff T, Trayhern L. Interpersonal vulnerability to depression in high-risk children: the role of insecure attachment and reassurance seeking. *J Clinl Child Adolesc Psychol* 2005;**34**:182–92	1
2	Abela JR, Zinck S, Kryger S, Zilber I, Hankin BL. Contagious depression: negative attachment cognitions as a moderator of the temporal association between parental depression and child depression. *J Clin Child Adolesc Psychol* 2009;**38**:16–26	4
3	Abrams KY, Rifkin A, Hesse E. Examining the role of parental frightened/frightening subtypes in predicting disorganized attachment within a brief observational procedure. *Dev Psychopathol* 2006;**18**:345–61	1
4	Abrams KY. *Pathways to Disorganization: A Study Concerning Varying Types of Parental Frightened and Frightening Behaviors as Related to Infant Disorganized Attachment*. PhD thesis. Berkeley, CA: University of California; 2001	1
5	Adams BL. *An Investigation of the Interrelationships among Security of Attachment, Parenting Attitudes, and the Development of Competence.* PhD thesis. Norfolk, VA: Virginia Consortium For Professional Psychology, Old Dominion University; 1995	4
6	Ades LAF. *Maternal Employment, Attachment, and Breastfeeding: Pathways to Early Childhood Problem Behaviors.* PhD thesis. Lincoln, NB: University of Nebraska; 2010	1
7	Ahern NR, Ruland JP. Maternal-fetal attachment in African-American and Hispanic-american women. *J Perinat Educ* 2003;**12**:27–35	1
8	Ainsworth MD. Patterns of attachment behavior shown by the infant in interaction with his mother. *Merrill Palmer Q* 1964;**10**	4
9	Akai CE, Guttentag CL, Baggett KM, Noria CC, Centers ftPoCN. Enhancing parenting practices of at-risk mothers. *J Prim Prev* 2008;**29**:223–2	2
10	Alers V. Treating severely traumatised children and adolescents using sensory integration, attachment theory and clinical reasoning. *J Child Adolesc Ment Health* 2005;17:vi–vii	4
11	Alpermann M, Koch G. Parent-child-interaction based diagnostic in high risk families with infants and toddlers. *Prax Kinderpsychol Kinderpsychiatr* 2007;**56**:836–51	1
12	Ammen SA. A Play-Based Teen Parenting Program to Facilitate Parent–Child Attachment. In Kaduson HG, Schaefer CE. *Short-Term Play Therapy for Children*. New York, NY: Guilford Press; 2000. pp. 345–69	2
13	Ang RP. Dysfunctional parenting behaviors and parenting stress among mothers of aggressive boys. *Child Family Behav Ther* 2008;**30**:319–36	4

© Queen's Printer and Controller of HMSO 2015. This work was produced by Wright *et al.* under the terms of a commissioning contract issued by the Secretary of State for Health. This issue may be freely reproduced for the purposes of private research and study and extracts (or indeed, the full report) may be included in professional journals provided that suitable acknowledgement is made and the reproduction is not associated with any form of advertising. Applications for commercial reproduction should be addressed to: NIHR Journals Library, National Institute for Health Research, Evaluation, Trials and Studies Coordinating Centre, Alpha House, University of Southampton Science Park, Southampton SO16 7NS, UK.

Number	Reference	Reason
14	Armstrong KL, Fraser JA, Dadds MR, Morris J. A randomized, controlled trial of nurse home visiting to vulnerable families with newborns. *J Paediatr Child Health* 1999;**35**:237–44	2
15	Armstrong KL, Fraser JA, Dadds MR, Morris J. Promoting secure attachment, maternal mood and child health in a vulnerable population: a randomized controlled trial. *J Paediatr Child Health* 2000;**36**:555–62	2
16	Francis DL. Attachment disorder: a decline in the performance of the Bravo pH System. *Am J Gastroenterol* 2008;**103**:2663	1
17	Atzaba-Poria N, Meiri G, Millikovsky M, Barkai A, Dunaevsky-Idan M, Yerushalmi B. Father-child and mother-child interaction in families with a child feeding disorder: the role of paternal involvement. *Infant Ment Health J* 2010;**31**:682–98	2
18	B Cramer CRT, DN Stern. Outcome evaluation in brief mother-infant psychotherapy: a preliminary report. *Infant Ment Health J* 1990;**11**:278–300	2
19	Bakermans-Kranenburg MJ, Breddels-van Baardewijk P, Juffer F, Velderman MK, Van IJzendoorn MH. Insecure Mothers with Temperamentally Reactive Infants: A Chance for Intervention. In Juffer F, Bakermans-Kranenburg FJ, Van IJzendoorn MH. *Promoting positive parenting: An attachment-based intervention.* New York, NY: Taylor & Francis Group/Lawrence Erlbaum Associates; 2008. pp. 75–90	2
20	Bakermans-Kranenburg MJ, Van IJzendoorn MH, Juffer F. Less is More: Meta-Analytic Arguments for the use of Sensitivity-Focused Interventions. In Juffer F, Bakermans-Kranenburg FJ, Van IJzendoorn MH. *Promoting positive parenting: An attachment-based intervention.* New York, NY: Taylor & Francis Group/Lawrence Erlbaum Associates; 2008	2
21	Bakermans-Kranenburg MJ, Van IJzendoorn MH, Juffer F. Less is more: meta-analyses of sensitivity and attachment interventions in early childhood. *Psychol Bull* 2003;**129**:195–215	4
22	Barlow J, Coren E, Stewart-Brown S. Parent-training programmes for improving maternal psychosocial health. *Campbell Syst Rev* 2005. URL: http://www.thehealthwell.info/node/97084?&content=resource&member=572160&catalogue=Campbell%20Reviews,Systematic%20Reviews&collection=Mental%20Health%20&tokens_complete=true	4
23	Barlow J, Parsons J. Group-based parent-training programmes for improving emotional and behavioural adjustment in 0–3 year old children. *Campbell Syst Rev* 2005. URL: http://www.campbellcollaboration.org/lib/project/6/	4
24	Barnard KE, Magyary D, Sumner G, Booth CL, Mitchell SK, Spieker S. Prevention of parenting alterations for women with low social support. *Psychiatry: Interpers Biol Processes* 1988;**51**:248–53	4
25	Barnett D, Butler CM, Vondra JI. Atypical attachment in infancy and early childhood among children at development risk. VIII. Atypical patterns of early attachment: discussion and future directions. *Monogr Soc Res Child Dev* 1999;**64**:67–96, discussion 213–20	1
26	Becker A. Two cribs: bad for baby? *Psychology Today*; 2003	1
27	Becker-Weidman A. Dyadic Developmental Psychotherapy: A Multi-Year Follow-Up. In Sturt S. *New Developments in Child Abuse Research*. Hauppauge, NY: Nova Science Publishers; 2006. pp. 43–60	4
28	Becker-Weidman A. Treatment for children with reactive attachment disorder: dyadic developmental psychotherapy. *Child Adolesc Ment Health* 2008;**13**:52–U66	4
29	Belsky J, Rovine M. Temperament and attachment security in the strange situation: an empirical rapprochement. *Child Dev* 1987;**58**:787–95	1
30	Benjamin JL. *Biopsychosocial-Based Versus Behavioral-Based Parenting Model: A Clinical Trial for Adoptive Parents with Attachment-Challenged Children.* San Diego, CA: Alliant International University, California School of Professional Psychology. Dissertation Abstracts International: Section B: The Sciences and Engineering; 2010	4
31	Benoit D, Madigan S, Lecce S, Shea B, Goldberg S. Atypical maternal behavior toward feeding-disordered infants before and after intervention. *Infant Ment Health J* 2000;**22**:611–26	2
32	Berlin LJ, Whiteside-Mansell L, Roggman LA, Green BL, Robinson J, Spieker S. Testing maternal depression and attachment style as moderators of Early Head Start's effects on parenting. *Attach Hum Dev* 2011;**13**:49–67	2
33	Bernstein VJ, Hans SL, Percansky C. Advocating for the young-child in need through strengthening the parent-child relationship. *J Clin Child Psychol* 1991;**20**:28–41	4
34	Bigelow AE, Littlejohn M, Bergman N, McDonald C. The relation between early mother-infant skin-to-skin contact and later maternal sensitivity in South African mothers of low birth weight infants. *Infant Ment Health J* 2010;**31**:358–77	2

Number	Reference	Reason
35	Binney V, McKnight I, Broughton S. Relationship Play Therapy for Attachment Disturbances in Four to Seven Year Old Children. In Richer J, Forrest G. *The Clinical Applications of Ethology and Attachment Theory*. Oxford: ACPP; 1994	4
36	Bos K, Zeanah CH, Fox NA, Drury SS, McLaughlin KA, Nelson CA. Psychiatric outcomes in young children with a history of institutionalization. *Harv Rev Psychiatry* 2011;**19**:15–24	1
37	Bos KJ, Zeanah CH, Jr., Smyke AT, Fox NA, Nelson CA III. Stereotypies in children with a history of early institutional care. *Arch Pediatr Adolesc Med* 2010;**164**:406–11	1
38	Bosquet M, Egeland B. Associations among maternal depressive symptomatology, state of mind and parent and child behaviors: Implications for attachment-based interventions. *Attach Hum Dev* 2001;**3**:173–99	2
39	Bowen SM, Miller BC. Paternal attachment behavior as related to presence at delivery and preparenthood classes: a pilot study. *Nurs Res* 1980;**29**:307–11	4
40	Boyce LK, Boyce GC, King J, Cook GA, D'Zatko K, Akers AL. Developing relationships between very low birthweight infants and their mothers: a look at timing of intervention in relation to infant and maternal characteristics. *Early Child Serv* 2008;**2**:173–93	4
41	Britt GC, Myers BJ. *Brazelton Intervention with Substance Abusing Mothers and Their Infants: An Experimental Intervention*. Report number ED361098. Richmond, VA: Virginia Commonwealth University; Rockville, MD: National Instonstite on Drug Abuse (DHHS), Division of Resource Development; 1993	2
42	Brophy HE. *Adolescent Mothers and their Infants: A Home-Based Crisis Prevention Effort*. PhD thesis. Syracuse, NY: Syracuse University; 1997	4
43	Brophy-Herb HE, Honig AS. Reflectivity: key ingredient in positive adolescent parenting. *J Prim Prev* 1999;**19**:241–50	2
44	Brown LS, Wright J. Attachment theory in adolescence and its relevance to developmental psychopathology. *Clin Psychol Psychother* 2001;**8**:15–32	1
45	Call CD. *Effects of an Attachment Based Intervention on Parent–Child Relational Measures*. PhD thesis. Fort Worth, TX: Texas Christian University; 2011	4
46	Carbonell OA, Plata SJ, Pena PA, Cristo M, Posada G. Quality of maternal care: a comparison of preterm infants in kangaroo mother care and full-term infants in regular care. *Univ Psychol* 2010;**9**:773–85	2
47	Carson K, Virden S. Can prenatal teaching promote maternal attachment? Practising nurses test Carter-Jessop's prenatal attachment intervention. *Health Care Women Int* 1984;**5**:355–69	2
48	Carter-Jessop L. Promoting maternal attachment through prenatal intervention. *Am J Matern Child Nurs* 1981;**6**:107–12	2
49	Cassibba R, Van IJzendoorn MH, Coppola G, Bruno S, Costantini A, Gatto S, *et al.* Supporting Families with Preterm Children and Children Suffering from Dermatitis. In Juffer FM, Bakermans-Kranenburg MJ, Van IJzendoorn MH. *Promoting Positive Parenting: An Attachment-Based Intervention*. New York, NY: Taylor & Francis Group/Lawrence Erlbaum Associates; 2008. pp. 91–110	4
50	Cassidy J, Poehlmann J, Shaver P. An attachment perspective on incarcerated parents and their children. *Attach Hum Dev* 2010;**12**:285–8	1
51	Cassidy J, Ziv Y, Stupica B, Sherman LJ, Butler H, Karfgin A, *et al.* Enhancing attachment security in the infants of women in a jail-diversion program. *Attach Hum Dev* 2010;**12**:333–53	4
52	Cevasco AM. The effects of mothers' singing on full-term and preterm infants and maternal emotional responses. *J Music Ther* 2008;**45**:273–306	2
53	Chamberlain P, Weinrott M. Specialized foster care: treating seriously emotionally disturbed children. *Child Today* 1990;**19**:24	4
54	Chan MM. *Maternal Attachment Behaviors with Adopted and Birth Infants*. Report number ED286588. 1987	2
55	Cheng S, Kondo N, Aoki Y, Kitamura Y, Takeda Y, Yamagata Z. The effectiveness of early intervention and the factors related to child behavioural problems at age 2: a randomized controlled trial. *Early Hum Dev* 2007;**83**:683–91	2

© Queen's Printer and Controller of HMSO 2015. This work was produced by Wright *et al.* under the terms of a commissioning contract issued by the Secretary of State for Health. This issue may be freely reproduced for the purposes of private research and study and extracts (or indeed, the full report) may be included in professional journals provided that suitable acknowledgement is made and the reproduction is not associated with any form of advertising. Applications for commercial reproduction should be addressed to: NIHR Journals Library, National Institute for Health Research, Evaluation, Trials and Studies Coordinating Centre, Alpha House, University of Southampton Science Park, Southampton SO16 7NS, UK.

Number	Reference	Reason
56	Chiu SH, Anderson GC. Effect of early skin-to-skin contact on mother-preterm infant interaction through 18 months: randomized controlled trial. *Int J Nurs Stud* 2009;**46**:1168–80	2
57	Cicchetti D, Rogosch FA, Toth SL. Fostering secure attachment in infants in maltreating families through preventive interventions. *Dev Psychopathol* 2006;**18**:623–49	4
58	Cicchetti D, Rogosch FA, Toth SL. The effects of child maltreatment and polymorphisms of the serotonin transporter and dopamine D4 receptor genes on infant attachment and intervention efficacy. *Dev Psychopathol* 2011;**23**:357–72	4
59	Cicchetti D, Rogosch FA, Toth SL. The efficacy of toddler-parent psychotherapy for fostering cognitive development in offspring of depressed mothers. *J Abnormal Child Psychol* 2000;**28**:135–48	2
60	Clark HB, Prange ME, Lee B, Boyd LA, McDonald BA, Stewart ES. Improving adjustment outcomes for foster children with emotional and behavioral disorders early findings from a controlled study on individualized services. *J Emotional Behav Dis* 1994;**2**:207–18	4
61	Cohen NJ, Lojkasek M, Muir E, Muir R, Parker CJ. Six-month follow-up of two mother-infant psychotherapies: convergence of therapeutic outcomes. *Infant Ment Health J* 2002;**23**:361–80	4
62	Cohen NJ, Muir E, Lojkasek M, Muir R, Parker CJ, Barwick M, *et al.* Watch, wait, and wonder: testing the effectiveness of a new approach to mother-infant psychotherapy. *Infant Ment Health J* 1999;**21**:131	4
63	Cohen SE, Woods WA. The role of epidural morphine in the postcesarean patient: efficacy and effects on bonding. *Anesthesiology* 1983;**58**:500–4	2
64	Constantino JN, Hashemi N, Solis E, Alon T, Haley S, McClure S, *et al.* Supplementation of urban home visitation with a series of group meetings for parents and infants: results of a "real-world" randomized, controlled trial. *Child Abuse Negl* 2001;**25**:1571–81	2
65	Cotton-Cornelius D. Change processes and the behavior of foster children as a function of an increased number of counseling sessions and the therapeutic alliance: A 12–18 month longitudinal analysis. Chicago, IL: The Chicago School of Professional Psychology. *Dissertation Abstracts International: Section B: The Sciences and Engineering*; 2004	4
66	Crockenberg SC, Rutter M, Bakermans-Kranenburg MJ, Van IJzendoorn MH, Juffer F. The effects of early social-emotional and relationship experience on the development of young orphanage children. *Monogr Soc Res Child Dev* 2008;**73**:245–62	4
67	Curry MA. Maternal attachment behavior and the mother's self-concept: the effect of early skin-to-skin contact. *Nurs Res* 1982;**31**:73–8	2
68	Cyr C, Dubois-Comtois K, Moss E. Mother-child conversations and the attachment of children in the pre-school period. *Can J Behav Sci-Rev Can Sci Comport* 2008;**40**:140–52	4
69	Daniel B, Taylor J, Scott J, Derbyshire D, Neilson D. *Recognizing and Helping the Neglected Child: Evidence-Based Practice for Assessment and Intervention.* London: Jessica Kingsley Publications; 2011	4
70	Davidson CE. The intergenerational transmission of resilience and risk: maternal depression, maternal adult attachment status, and the competence of school-aged children. Tufts University, Medford, MA. *Dissertation Abstracts Int*; 1993	4
71	de l'Etoile SK. Infant behavioral responses to infant-directed singing and other maternal interactions. *Infant Behav Dev* 2006;**29**:456–70	4
72	Declercq S, Nicolis H. La theorie de l attachement au secours des adolescents limites hospitalises. *Neuropsychiatrie de l Enfance et de l Adolescence* 2010;**58**:107–11	PCO
73	DeGarmo DS, Patterson GR, MS. F. How do outcomes in a specified parent training intervention maintain or wane over time? *Prev Sci* 2004;**5**:73–89	2
74	Del Castillo-Garcia A. *Nurturing Touch: Facilitating Parenting Confidence and Attachment Relations*. PhD thesis. The Claremont Graduate University; 2011	4
75	Denham SA, Burton R. A social-emotional intervention for at-risk 4-year-olds. *J Sch Psychol* 1996;**34**:225–45	1
76	Diana, Mark S. The *Relationship Between Observations of Affiliative Behavior Patterns for Parents and Toddlers and Parental Reports of Caregiving, Play and Support-Control Behaviors*. Washington, DC: distributed by ERIC Clearinghouse; 1985	1

Number	Reference	Reason
77	Dobbs J. The association between preschool children's socio-emotional functioning and their mathematical skills. *J Appl Dev Psychol* 2006;**27**:97–108	1
78	Dozier M, Bick J, Bernard K. Attachment-Based Treatment for Young, Vulnerable Children. In Osofsky JD. *Clinical Work with Traumatized Young Children*. New York, NY: Guilford Press; 2011. pp. 75–95	4
79	Dozier M, Bick J, Bernard K. Intervening with foster parents to enhance biobehavioral outcomes among infants and toddlers. *Zero to Three* 2011;**31**:17–22	4
80	Dozier M, Peloso E, Lewis E, Laurenceau J-P, Levine S. Effects of an attachment-based intervention of the cortisol production of infants and toddlers in foster care. *Dev Psychopathol* 2008;**20**:845–59	3B
81	Dozier M, Peloso E, Lindhiem O, Gordon M, Manni M, Sepulveda S, *et al.* Developing evidence-based interventions for foster children: an example of a randomized clinical trial with infants and toddlers. *J Soc Issues* 2006;**62**:767–85	2
82	Driscoll TG. Juvenile misbehavior and the school: An educational cohort analysis. Grand Forks, ND: University of North Dakota. *Dissertation Abstracts Int;* 1993	NF
83	Drotar D. *Family Centered Intervention with Infant Failure to Thrive*. Report number ED361098. Paper presented at the Annual Meeting of the American Psychological Association, Los Angeles, CA, 24–28 August 1981. URL: http://eric.ed.gov/?id=ED208960	4
84	Drury SS, Gleason MM, Theall KP, Smyke AT, Nelson CA, Fox NA, *et al.* Genetic sensitivity to the caregiving context: the influence of 5httlpr and BDNF val66met on indiscriminate social behavior. *Physiol Behav* 2011;**106**:728–35	1
85	Duggan AK, Berlin LJ, Cassidy J, Burrell L, Tandon S. Examining maternal depression and attachment insecurity as moderators of the impacts of home visiting for at-risk mothers and infants. *J Consult Clin Psychol* 2009;**77**:788–99	2
86	Dugravier R, Guedeney A, Saias T, Greacen T, Tubach F. CAPEDP: a preventive longitudinal study on infant–mother relationship disorders. *Neuropsychiatrie de l'Enfance et de l'Adolescence* 2009;**57**:482–6	PCO
87	Dugravier R. Understanding consequences of hospitalization within attachment theory. *Arch Pediatr* 2010;**17**:723–4	4
88	Dumaret A, Picchi V. Early intervention: Psychosocial outcome of families and children's development. *Annales Medico-Psychologiques* 2005;**163**:476–85	4
89	Edwards J. The use of music therapy to promote attachment between parents and infants. *Arts Psychother* 2011;**38**:190–5	4
90	Egeland B, Sroufe LA. Developmental Sequelae of Maltreatment in Infancy. In Rizley R, Cichetti D. *Developmental Perspectives in Child Maltreatment*. San Francisco, CA: Jossey-Bass; 1981. pp. 77–92	4
91	Eickhorst A, Schweyer D, Kohler H, Jelen-Mauboussin A, Kunz E, Sidor A, *et al.* [Sensitivity of parents with psychosocial stress.] *Bundesgesundheitsblatt Gesundheitsforschung Gesundheitsschutz* 2010;**53**:1126–33	4
92	Erickson MF, Egeland B. Linking theory and research to practice: the Minnesota longitudinal study of parents and children and the STEEPTM program. *Clin Psychol* 2004;**8**:5–9	4
93	Erickson MF, Korfmacher J, Egeland BR. Attachments past and present: implications for therapeutic intervention with mother-infant dyads. *Dev Psychopathol* 1992;**4**:495–507	2
94	Fairchild SR. Attachment representations and parental memories of incarcerated fathers. *Child Adolesc Soc Work J* 2009;**26**:361–77	1
95	Feinberg ME, Kan ML, Goslin MC. Enhancing coparenting, parenting, and child self-regulation: effects of family foundations 1 year after birth. *Prev Sci* 2009;**10**:276–85	2
96	Feinberg ME, Kan ML. Establishing family foundations: intervention effects on coparenting, parent/infant well-being, and parent–child relations. *J Fam Psychol* 2008;**22**:253–63	2
97	Feinfield KA, Baker BL. Empirical support for a treatment program for families of young children with externalizing problems. *J Clin Child Adolesc Psychol* 2004;**30**:182–95	2
98	Feldman R, Weller A, Sirota L, Eidelman AI. Testing a family intervention hypothesis: the contribution of mother-infant skin-to-skin contact (kangaroo care) to family interaction, proximity, and touch. *J Fam Psychol* 2003;**17**:94–107	2

© Queen's Printer and Controller of HMSO 2015. This work was produced by Wright *et al.* under the terms of a commissioning contract issued by the Secretary of State for Health. This issue may be freely reproduced for the purposes of private research and study and extracts (or indeed, the full report) may be included in professional journals provided that suitable acknowledgement is made and the reproduction is not associated with any form of advertising. Applications for commercial reproduction should be addressed to: NIHR Journals Library, National Institute for Health Research, Evaluation, Trials and Studies Coordinating Centre, Alpha House, University of Southampton Science Park, Southampton SO16 7NS, UK.

Number	Reference	Reason
99	Field T. *Leavetakings and Reunions of Infants, Toddlers, Preschoolers and Their Parents.* Report number ED219161. 1978	1
100	Fisher PA, B. Burraston, Pears. K. The early intervention foster care program: permanent placement outcomes from a randomized trial. *Child Maltreat* 2005;**10**:61–71	2
101	Fisher PA, Gunnar MR, Dozier M, Bruce J, Pears KC. Effects of therapeutic interventions for foster children on behavioral problems, caregiver attachment, and stress regulatory neural systems. *Ann N Y Acad Sci* 2006;**1094**:215–25	4
102	Fisher PA, Van Ryzin MJ, Gunnar MR. Mitigating HPA axis dysregulation associated with placement changes in foster care. *Psychoneuroendocrinology* 2011;**36**:531–9	2
103	Ford JD. Neurobiological and Developmental Research: Clinical Implications. In Courtois CA, Ford JD, van der Kolk BA, Herman JL. *Treating Complex Traumatic Stress Disorders: An Evidence-Based Guide.* New York, NY: Guilford Press; 2009. pp. 31–58	1
104	Foster D, Davies S, Steele H. The evacuation of British children during World War II: a preliminary investigation into the long term psychological effects. *Aging Ment Health* 2003;**7**:398–408	4
105	Fraiberg S, Adelson E, Shapiro V. Ghosts in the nursery: a psychoanalytic approach to impaired infant–mother relationships. *J Am Acad Child Psychiatry* 1975;**14**:387–421	4
106	Frank ML. *Therapeutic Change at Project Pride: Residential Substance Abuse Treatment from an Attachment Perspective.* Palo Alto University; 2010	2
107	Franz M, Weihrauch L, Buddenberg T, Guttgemanns J, Haubold S, Schafer R. Effectiveness of an attachment-oriented parental training program for single mothers and their children: PALME. *Kindheit Entwicklung* 2010;**19**:90–101	4
108	Franz M, Weihrauch L, Buddenberg T, Schafer R. PALME: effectiveness of attachment-based parental training for single mothers and their children. *Psychotherapeut* 2009;**54**:357–69	PCO
109	Franz M, Weihrauch L, Schaefer R. PALME: a preventive parental training program for single mothers with preschool aged children. *J Public Health* 2011;**19**:305–19	2
110	Gaensbauer TJ, *et al.* Relationships between attachment behavior in the laboratory and the caretaking environment. *Infant Behav Dev* 1985;**8**:355–69	1
111	Gaensbauer TJ, Harmon RJ, Cytryn L, McKnew DH. Social and affective development in infants with a manic-depressive parent. *Am J Psychiatry* 1984;**141**:223–9	4
112	Gaffney KF. Maternal-fetal attachment in relation to self-concept and anxiety. *Matern Child Nurs J* 1986;**15**:91–101	4
113	Gaffney M, Greene SM, Wieczorek-Deering D, Nugent J. The concordance between mother-infant attachment at 18 months and maternal attachment 10 years later among married and single mothers. *Ir J Psychol* 2000;**21**:154–70	4
114	Gainey RR, Catalano RF, Haggerty KP, Hoppe MJ. Deviance among the children of heroin addicts in treatment: impact of parents and peers. *Deviant Behav* 1997;**18**:143–59	2
115	Ganiban J, Barnett D, Cicchetti D. Negative reactivity and attachment: Down syndrome's contribution to the attachment-temperament debate. *Dev Psychopathol* 2000;**12**:1–21	1
116	Gao Y, Raine A, Chan F, Venables PH, Mednick SA. Early maternal and paternal bonding, childhood physical abuse and adult psychopathic personality. *Psychol Med* 2010;**40**:1007–16	1
117	Garmon LC. *Relations between Attachment Representations and Moral Judgment.* PhD thesis. Ohio State University; 2000	4
118	Garwood MM. *Parental Sensitivity, Parenting Beliefs, and Child Temperament: Modeling Effects on Mother- and Father-Infant Attachment.* PhD thesis. University of Notre Dame; 1998	4
119	Garzarelli L. The effectiveness of parenting programs on recidivism rates. Naples, FL: Walden University. *Dissertation Abstracts International Section A: Humanities and Social Sciences*; 2011	4
120	Gathwala G, Singh B, Balhara B. KMC facilitates mother baby attachment in low birth weight infants. *Indian J Pediatr* 2008;**75**:43	3B
121	Gaudet C. Pregnancy after perinatal loss: association of grief, anxiety and attachment. *J Reprod Infant Psychol* 2010;**28**:240–51	1

Number	Reference	Reason
122	Gaudin J, M, Wodarski J, S, Arkinson M, Avery L, S. Remedying child neglect: effectiveness of social network interventions. *J Appl Socl Sci* 1990;**15**:97–123	2
123	Geddes H. *Attachment in the Classroom. The Links Between Children's Early Experience, Emotional Well-Being and Performance in School*. London: Worth Publishing Limited; 2006	4
124	Gelfand DM, Teti DM, Seiner SA, Jameson PB. Helping mothers fight depression: evaluation of a home-based intervention program for depressed mothers and their infants. *J Clin Child Psychol* 1996;**25**:406–22	4
125	George M. Can babies get depressed? *Children Now* 2004 pp. 20–1	4
126	George MR, Cummings EM, Davies PT. Positive aspects of fathering and mothering, and children's attachment in kindergarten. *Early Child Dev Care* 2010;**180**:107–19	4
127	Gerner L. Exploring prenatal attachment: factors that facilitate paternal attachment during pregnancy. Fresno, CA: California School of Professional Psychology, Alliant International University. *Dissertation Abstracts International: Section B: The Sciences and Engineering;* 2006	1
128	Gervai J, Novak A, Lakatos K, Toth I, Danis I, Ronai Z, *et al.* Infant genotype may moderate sensitivity to maternal affective communications: attachment disorganization, quality of care, and the DRD4 polymorphism. *Soc Neurosci* 2007;**2**:307–19	4
129	Geserick B, Spangler G. The influence of early attachment experiences and concurrent maternal support on task-oriented behavior of six-year-olds during cognitive a cognitively challenging situation. *Psychol Erzieh Unterr* 2007;**54**:86–102	1
130	Gewirtz JL, Pelaez-Nogueras M. The Attachment Metaphor and the Conditioning of Infant Separation Protests. In Gewirtz JL, Kurtines WM, Lamb JL. *Intersections with Attachment*. Hillsdale, NJ: Lawrence Erlbaum Associates, Inc.; 1991. pp. 123–44	4
131	Giannini M, Gori A, De Sanctis E, Schuldberg D. Attachment in psychotherapy: psychometric properties of the Psychological Treatment Inventory Attachment Styles Scale (PTI-ASS). *J Psychother Integr* 2011;**21**:363–81	4
132	Gibson LA. *Adult Attachment and Maternal Representations of Gender During Pregnancy: Their Impact on the Child's Subsequent Gender-Role Development.* PhD thesis. New York, NY: City University; 1998	1
133	Gil A, Escosteguy NU, Picon P, Zanel AP, Luz C, Litvin E, *et al.* Adrenocortical responses in children to a stress-situation (relation with observation of behavior and interaction with caregivers, considering variables "temperament and attachment"). *Revista de Psiquiatria do Rio Grande do Sul* 2002;**24**:135–42	1
134	Gilbride KE. *Adolescent Maternal Behavior: Assessment over Time and its Relationship to Infant-Mother Attachment*. PhD thesis. University of Utah; 1989	4
135	Glowinski AL. Reactive attachment disorder: an evolving entity. *J Am Acad Child Adolesc Psychiatr* 2011;**50**:210–12	4
136	Gomes-Pedro J. *Early Intervention and Mother-Infant Interaction during the First Three Months of Life.* Report number ED292558. 1987	2
137	Goodman G. Empirical evidence supporting the conceptual relatedness of object representations and internal working models. *J Am Psychoanalytic Assoc* 2005;**53**:597–617	1
138	Gooen JA. The relationship among attachment styles of adult caregiving daughters, caregiver burden, and psychological well-being. Garden City, NY: Adelphi University. *Dissertation Abstracts International: Section B: The Sciences and Engineering;* 2000	1
139	Gordon JE. The psychological and social functioning of adult children of alcoholics: an attachment perspective. Fairfax, VA: George Mason University. *Dissertation Abstracts International: Section B: The Sciences and Engineering;* 1995	1
140	Gottfried DC. Adult sequelae of childhood sexual abuse: dissociation, attachment style and substance use. Garden City, NY: Institute of Advanced Psychological Studies, Adelphi University. *Dissertation Abstracts International: Section B: The Sciences and Engineering;* 2005	1
141	Granger CEG. *The Role of the Reflective Function of Foster Carers in the Quality of Long Term Foster Placements: An Exploratory Study*. D.Clin.Psych thesis. Oxford University; 2008	1

© Queen's Printer and Controller of HMSO 2015. This work was produced by Wright *et al.* under the terms of a commissioning contract issued by the Secretary of State for Health. This issue may be freely reproduced for the purposes of private research and study and extracts (or indeed, the full report) may be included in professional journals provided that suitable acknowledgement is made and the reproduction is not associated with any form of advertising. Applications for commercial reproduction should be addressed to: NIHR Journals Library, National Institute for Health Research, Evaluation, Trials and Studies Coordinating Centre, Alpha House, University of Southampton Science Park, Southampton SO16 7NS, UK.

Number	Reference	Reason
142	Graves T. *Maternal Object Representations and Implications for the Child's Experience of Posttraumatic Stress: A Single Case Phenomenological Study.* PhD thesis. Chestnut Hill College; 2009	1
143	Green BL, Furrer CJ, McAllister CL. Does attachment style influence social support or the other way around? A longitudinal study of Early Head Start mothers. *Attachm Hum Dev* 2011;**13**:27–47	2
144	Grette Moe R. Groupwork as early intervention in high risk families. *Groupwork* 1989:1989–90	4
145	Grienenberger JF. The impact of maternal reflective functioning on mother-infant affective communication: Exploring the link between mental states and observed caregiving behavior. New York, NY: City University of New York. *Dissertation Abstracts International: Section B: The Sciences and Engineering* 2003	1
146	Grossman PB. Attachment style and history of parents of aggressive children: effects on response to intervention. College Station, TX: Texas A&M University. *Dissertation Abstracts International: Section B: The Sciences and Engineering;* 1994.	NF
147	Guedeney A. Kwashiorkor, depression, and attachment disorders. *Lancet* 1995;**34**:1293	4
148	Guedeney N, Lamas C, Bekhechi V, Mintz AS, Guedeney A. Developpement du processus d'attachement entre un bebe et sa mere. *Arch Pediatr* 2008;**15**(Suppl. 1):S12–S9.	2
149	Guile JM. Accueil partiel en placement familial therapeutique: contribution des theories de l'attachement. *Neuropsychiatrie De L Enfance Et De L Adolescence* 2003;**51**:450–4	4
150	Gullon-Rivera AL. *Puerto Rican Kindergarteners' Representation of the Self Within Family Relationships: Are They Related to their Self-Worth, Child–Mother Relationship and Behavioral Adjustment?* PhD thesis. University of Wisconsin – Madison; 2009	1
151	Gunaydin G, Selcuk E, Sumer N, Uysal A. Psychometric evaluation of the short form of inventory of parent and peer attachment. *Turk Psikoloji Yazilari* 2005;**8**:13–23	4
152	Gurney Smith B, *et al.* 'In time and in tune': the Fostering Attachments Group – capturing sustained change in both caregiver and child. *Adoption Fostering* 2010;**34**:50–60	2
153	Guyer B, Hughart N, Strobino D, Jones A, Scharfstein D. Assessing the impact of pediatric-based developmental services on infants, families and clinicians: challenges to evaluating the healthy steps program. *Pediatrics* 2000;**105**:E33	2
154	Guzder J, Bond S, Rabiau M, Zelkowitz P, Rohar S. The relationship between alliance, attachment and outcome in a child multi-modal treatment population: pilot study *J Can Acad Child Adolesc Psychiatry* 2011;**20**:196–202	4
155	Gwynne K, Zilibowitz M. Spilstead Model of Early Intervention for Vulnerable Families. World Congress of Internal Medicine, WCIM 2010 in Conjunction with Physicians Week, Melbourne, VIC, Australia	4
156	Hampson RB, Schulte MA, Ricks CC. Individual vs group training for foster parents: efficacy/ effectiveness evaluations. *Fam Relations* 1983;**32**:191–201	4
157	Harris JR. Attachment theory underestimates the child. *Behav Brain Sci* 2009;**32**:30	4
158	Hartmann M. Attachment disorder and aurum muriaticum natronatum. *Homoeopathic Links* 2009;**22**:194–6	4
159	Havighurst SS, Wilson KR, Harley AE, Prior MR. Tuning in to kids: an emotion-focused parenting program-initial findings from a community trial. *J Community Psychol* 2009;**37**:1008–23	2
160	Hebebrand J, Mohler E. Children of impaired parents. *Jugendpsychiatr Psychother* 2009;**37**:307–8	4
161	Heinicke CM, Levine MS. The AAI Anticipates the Outcome of a Relation-Based Early Intervention. In Steele H, Steele M. *Clinical Applications of the Adult Attachment Interview*. New York, NY: Guilford Press; 2008. pp. 99–125	2
162	Henley D. Attachment disorders in post-institutionalized adopted children: art therapy approaches to reactivity and detachment. *Arts Psychother* 2005;**32**:29–46	4
163	Honig AS, Pfannestiel A. *Clinical Issues in Reaching Low-Income Fathers with a Program of "Information and Insights about Infants" (III)*. Report number ED291474. 1988	2
164	Honig AS. Research in review: risk factors in infants and young children. *Young Child* 1984;**39**:60–73	1

Number	Reference	Reason
165	Hooper LM. the application of attachment theory and family systems theory to the phenomena of parentification. *Fam J* 2007;**15**:217–23	4
166	Hornstein C, Hohm E, Trautmann-Villalba P. Postpartum bonding disorder: a risk constellation for infanticide? *Forensische Psychiatrie, Psychologie, Kriminologie* 2009;**3**:3–10	4
167	Howe D. Attachment disorders: disinhibited attachment behaviours and secure base distortions with special reference to adopted children. *Attach Hum Dev* 2003;**5**:265–70	4
168	Howe D. *Child Abuse and Neglect: Attachment, Development and Intervention.* London: Palgrave Macmillan; 2005	4
169	Howes C, Galinsky E, Kontos S. Child care caregiver sensitivity and attachment. *Soc Dev* 1998;**7**:25–36	4
170	Hughes DA. Psychological interventions for the spectrum of attachment disorders and intrafamilial trauma. *Attach Hum Dev* 2003;**5**:271–8	4
171	Humber N, Moss E. The relationship of preschool and early school age attachment to mother-child interaction. *Am J Orthopsychiatry* 2005;**75**:128–41	4
172	Huth-Bocks AC, Levendosky AA, Theran SA, Bogat G. The impact of domestic violence on mothers' prenatal representations of their infants. *Infant Ment Health J* 2004;**25**:79–98	1
173	Isfort M, Brühl A, Bünte A, Jorch G, Kray A. [Contributions and effects of parental basal stimulation contact nursing (BSK) within the scope of the gentle nursing of premature infants–I.] *Kinderkrankenschwester* 2008;**27**:233–40	2
174	Jacques SL. The effect of a nursing intervention during the third trimester on maternal-fetal attachment and pregnancy outcomes. Austin, TX: University of Texas at Austin. *Dissertation Abstracts International: Section B: The Sciences and Engineering*; 1995	2
175	Johnson DE, Guthrie D, Smyke AT, Koga SF, Fox NA, Zeanah CH, *et al.* Growth and associations between auxology, caregiving environment, and cognition in socially deprived romanian children randomized to foster vs ongoing institutional care. *Arch Pediatr Adolesc Med* 2010;**164**:507–16	2
176	Juffer F, Bakermans-Kranenburg MJ, Van IJzendoorn MH. Promoting Positive Parenting: An Attachment-Based Intervention. In Juffer F, Bakermans-Kranenburg MJ, Van IJzendoorn MH. *Promoting Positive Parenting: An Attachment-Based Intervention.* New York, NY: Taylor & Francis Group/Lawrence Erlbaum Associates; 2008. p. 238	4
177	Juffer F, Bakermans-Kranenburg MJ, Van IJzendoorn MH. The importance of parenting in the development of disorganized attachment: evidence from a preventive intervention study in adoptive families. *J Child Psychol Psychiatry* 2005;**46**:263–74	4
178	Juffer F, Hoksbergen RA, Riksen-Walraven J, Kohnstamm GA. Early intervention in adoptive families: Supporting maternal sensitive responsiveness, infant-mother attachment, and infant competence. *J Child Psychol Psychiatry* 1997;**38**:1039–50	4
179	Juffer F, Van IJzendoorn M, Duyvesteyn M. Parenting support and intergenerational transmission of attachment: a review of intervention studies. *Kind en Adolescent* 1994;**15**:204–21	4
180	Juffer F, Van IJzendoorn MH, Bakermans-Kranenburg MJ. Supporting Adoptive Families with Video-Feedback Intervention. In Juffer F, Bakermans-Kranenburg MJ, Van IJzendoorn MH. *Promoting Positive Parenting: An Attachment-Based Intervention.* New York, NY: Taylor & Francis Group/Lawrence Erlbaum Associates; 2008. pp. 139–53	4
181	Kalinauskiene L, Kusakovskaja I, Cekuoliene D, Kiltanaviciute V. Effects of video-feedback correction of infant–mother interaction on two-years-olds' behaviour. *Psichologija* 2009;**40**:53–65	PCO
182	Kennedy H, Landor M, Todd L. Video Interaction Guidance as a method to promote secure attachment. *Educ Child Psychol* 2010;**27**:59–72	4
183	Kennell JH, Trause MA, Klaus MH. Evidence for a sensitive period in the human mother. *Ciba Found Symp* 1975;**33**:87–101	4
184	Kerhulas IA. The effects of attachment history on altruistic behaviour. Los Angeles, CA: California School of Professional Psychology. *Dissertation Abstracts International*; 1986	NF
185	King P. A 'rebirth' brings death. A controversial therapy for children diagnosed with attachment disorder draws intense scrutiny. *Newsweek* 2000;**135**:65	4

© Queen's Printer and Controller of HMSO 2015. This work was produced by Wright *et al.* under the terms of a commissioning contract issued by the Secretary of State for Health. This issue may be freely reproduced for the purposes of private research and study and extracts (or indeed, the full report) may be included in professional journals provided that suitable acknowledgement is made and the reproduction is not associated with any form of advertising. Applications for commercial reproduction should be addressed to: NIHR Journals Library, National Institute for Health Research, Evaluation, Trials and Studies Coordinating Centre, Alpha House, University of Southampton Science Park, Southampton SO16 7NS, UK.

Number	Reference	Reason
186	Kissgen R. Early intervention – use of attachment and childhood development research in education, counseling, treatment and prevention. *Psychol Erzieh Unterr* 2003;**50**:325–7	4
187	Kokcu F, Kesebir S, Dereboy F. The relationship attachment style, personality, and temperament in patients with bipolar disorder and their children: a controlled study. *Bipolar Disord* 2010;**12**:32–3	1
188	Kontos D. A study of the effects of extended mother-infant contact on maternal behavior at one and three months. *Birth Fam J* 1978;**5**:133–40	4
189	Korfmacher J, Adam E, Ogawa J, Egeland B. Adult attachment: implications for the therapeutic process in a home visitation intervention. *Appl Dev Sci* 1997;**1**:43–52	2
190	Kraemer S, Loader P. Passing through life: alexithymia and attachment disorders. *J Psychosom Res* 1995;**39**:937–41	4
191	Krupka A. *The Quality of Mother–Infant Interactions in Families at Risk for Maladaptive Parenting*. PhD thesis. London, ON: University of Western Ontario; 1996	2
192	Kusube T. Disinhibited attachment disorder of childhood. *Ryoikibetsu Shokogun Shirizu* 2003;**40**:57–8	4
193	Kusube T. Reactive attachment disorder of infancy or early childhood. *Ryoikibetsu Shokogun Shirizu* 2003;**40**:54–6	4
194	Lakatos K, Toth I, Ney K, Gervai J, Nemoda Z, Ronai Z, *et al.* Genetic risk for attachment disorganisation in infancy: the role of the D4 dopamine receptor (DRD4). *J Reprod Infant Psychol* 2001;**19**:269	1
195	Lamb ME, Thompson RM, Gardner W, Charnov EL, Estes D. Security of infantile attachment as assessed in the Strange Situation – its study and biological interpretation. *Behav Brain Sci* 1984;**7**:127–47	4
196	Lamb ME. Attachments, social networks, and developmental contexts. *Hum Dev* 2005;**48**:108–12	1
197	Lampe A, Mitmansgruber H, Gast U, Schussler G, Reddemann L. Treatment outcome of psychodynamic trauma therapy in an inpatient setting. *Neuropsychiatrie* 2008;**22**:189–97	4
198	Landry SH, Smith KE, Swank PR. Responsive parenting: establishing early foundations for social, communication, and independent problem-solving skills. *Dev Psychol* 2006;**42**:627–42	2
199	Lartigue M, Vives Rocabert J. Development of infant–mother bond: a comparative longitudinal study. *Revista Mexicana de Psicologia* 1992;**9**:127–42	4
200	Latifses V. Teaching expectant fathers to massage their partners: an exploration of fetal attachment behaviors, anxiety, and marital adjustment in fathers. *Dissertation Abstracts International: Section B: The Sciences and Engineering;* 2004	2
201	Lecannelier F, Undurraga V, Olivares AM, Rodriguez J, Nunez JC, Hoffmann M, *et al.* Outcome study about two early attachment based interventions in dyads mother-baby from Santiago De Chile. *Rev Argent Clin Psicol* 2009;**18**:143–55	PCO
202	Lee KS, Jeong SJ, Shin YJ. A comparative study of reactive attachment disorder with autistic child in social cognition – focused on social referencing and joint attention. *Korean J Clin Psychol* 2000;**19**:793–806	1
203	Lester BM, Masten A, McEwen B. Part II. Neurobiological Processes. In Lester BM, Masten A, McEwen B. *Resilience in Children.* Malden: Blackwell Publishing; 2006. pp. 164–247	2
204	Lindhiem OJ. *Modeling change: An Attachment-Based Intervention with High-Risk Birth Mothers.* Newark, DE: University of Delaware; 2010	2
205	Liptak GS, Keller BB, Feldman AW, Chamberlin RW. Enhancing infant development and parent–practitioner interaction with the Brazelton Neonatal Assessment Scale. *Pediatrics* 1983;**72**:71–8	2
206	Lyons-Ruth K, Connell DB, Grunebaum HU, Botein S. Infants at social risk: maternal depression and family support services as mediators of infant development and security of attachment. *Child Dev* 1990;**61**:85–98	4
207	Lyons-Ruth K, Zoll D, Connell D, Grunebaum HU. The depressed mother and her one-year-old infant: environment, interaction, attachment, and infant development. *New Directions Child Dev* 1986;**34**:61–82	1

Number	Reference	Reason
208	Ma CQ, Huebner ES. Attachment relationships and adolescents' life satisfaction: some relationships matter more to girls than boys. *Psychol Schools* 2008;**45**:177–90	1
209	Mackay T, Reynolds S, Kearney M. From attachment to attainment: The impact of nurture groups on academic achievement. *Educ Child Psychol* 2010;**27**:100–10	4
210	Madigan S, Bakermans-Kranenburg MJ, Van IJzendoorn MH, Moran G, Pederson DR, Benoit D. Unresolved states of mind, anomalous parental behavior, and disorganized attachment: a review and meta-analysis of a transmission gap. *Attach Hum Dev* 2006;**8**:89–111	4
211	Madigan S, Hawkins E, Goldberg S, Benoit D. Reduction of disrupted caregiver behavior using modified interaction guidance. *Infant Ment Health J* 2006;**27**:509–27	2
212	Mahon JM. A comparison of parent–child attachment to a play therapy program. Boston, MA: Boston University. *Dissertation Abstracts International Section A: Humanities and Social Sciences*; 1994	4
213	Maki PS. *Attachment Disorders: Effectiveness of an Identified Attachment Intervention with Adopted Children.* PhD thesis. Minneapolis, MN: Capella University; 2002	2
214	Manassis K, Bradley S, Goldberg S, Hood J, Swinson RP. Behavioural inhibition, attachment and anxiety in children of mothers with anxiety disorders. *Can J Psychiatry* 1995;**40**:87–92	1
215	Manning-Orenstein G. A birth intervention: The therapeutic effects of doula support versus Lamaze preparation on first-time mothers' working models of caregiving. *Altern Ther Health Med* 1998;**4**:73–81	2
216	Marcovitch S, Goldberg S, Gold A, Washington J, *et al.* Determinants of behavioural problems in Romanian children adopted in Ontario. *Int J Behav Dev* 1997;**20**:17–31	2
217	Marcus RF, Kramer C. Reactive and proactive aggression: attachment and social competence predictors. *J Genet Psychol* 2001;**162**:260–75	4
218	Marcus RF. The attachments of children in foster care. *Genetic Soc Gen Psychol Monogr* 1991;**117**:365–94	4
219	Marecki M, Wooldridge P, Dow A, Thompson J, Lechner-Hyman C. Early sibling attachment. *J Obstet Gynecol Neonatal Nurs* 1985;**14**:418–23	1
220	Marsh LB. *Impact of Attachment Training on Male Adolescents' Responses to Infant Simulator Dolls*. PhD thesis. Antioch University/New England Graduate School; 1999	4
221	Mascolo MF, Basseches M. Psychotherapy with infants and young children: repairing the effects of stress and trauma on early attachment. *J Appl Dev Psychol* 2009;**30**:563–6	4
222	Maslin-Cole C, Spieker SJ. Attachment as a Basis for Independent Motivation: A View from Risk and Nonrisk Samples. In Greenberg MT, Cicchetti D, Cummings EM. *Attachment in the Preschool Years: Theory, Research, and Intervention.* Chicago, IL: University of Chicago Press; 1990. pp. 245–72	4
223	McArthur LE. *Intimate Partner Violence, Attachment, and Coparenting Intervention Outcomes among Latino Teen Parents.* PhD thesis. University of Utah; 2011	2
224	McCarthy GDE. Doing well and doing poorly in care: Caregivers' attachment status and other risk and resilience predictors of children's outcomes in kinship, foster, and adoptive placements. West Whately, MA: Smith College School for Social Work. *Dissertation Abstracts International: Section B: The Sciences and Engineering*; 2007	2
225	McCormack D, Scott-Heyes G, McCusker CG. The impact of hyperemesis gravidarum on maternal mental health and maternal-fetal attachment. *J Psychosom Obstet Gynecol* 2011;**32**:79–87	4
226	McCormick MC, McCarthon C, Brooks-Gunn J, Belt P, Gross RT. The infant health and development program: interim summary. *J Dev Behav Pediatr* 1998;**19**:359–70	2
227	McFarland J, Salisbury AL, Battle CL, Hawes K, Halloran K, Lester BM. Major depressive disorder during pregnancy and emotional attachment to the fetus. *Arch Womens Ment Health* 2011;**14**:425–34	1
228	McLaughlin KA, Zeanah CH, Fox NA, Nelson CA. Attachment security as a mechanism linking foster care placement to improved mental health outcomes in previously institutionalized children. *J Child Psychol Psychiatry* 2012;**53**:46–55	1
229	Meijssen D, Wolf M-J, van Bakel H, Koldewijn K, Kok J, van Baar A. Maternal attachment representations after very preterm birth and the effect of early intervention. *Infant Behav Dev* 2011;**34**:72–80	4

© Queen's Printer and Controller of HMSO 2015. This work was produced by Wright *et al.* under the terms of a commissioning contract issued by the Secretary of State for Health. This issue may be freely reproduced for the purposes of private research and study and extracts (or indeed, the full report) may be included in professional journals provided that suitable acknowledgement is made and the reproduction is not associated with any form of advertising. Applications for commercial reproduction should be addressed to: NIHR Journals Library, National Institute for Health Research, Evaluation, Trials and Studies Coordinating Centre, Alpha House, University of Southampton Science Park, Southampton SO16 7NS, UK.

Number	Reference	Reason
230	Melley AH, Cosgrove K, Norris-Shortle C, Kiser LJ, Levey EB, Coble CA, *et al.* Supporting positive parenting for young children experiencing homelessness: the PACT Therapeutic Nursery. *Zero to Three* 2010;**30**:39–45	4
231	Mentore JL. *The Effectiveness of Early Intervention with Young Children 'at risk': A Decade in Review.* PhD thesis. New York, NY: Fordham University; 2000	4
232	Mercer RT, Ferketich S, May K, DeJoseph J, Sollid D. Further exploration of maternal and paternal fetal attachment. *Res Nursing Health* 1988;**11**:83–95	4
233	Mesman J, Stolk MN, van Zeijl J, Alink LR, Juffer F, Bakermans-Kranenburg MJ, *et al.* Extending the Video-Feedback Intervention to Sensitive Discipline: The Early Prevention of Antisocial Behavior. In Juffer F, Bakermans-Kranenburg MJ, Van IJzendoorn MH. *Promoting Positive Parenting: An Attachment-Based Intervention*. New York, NY: Taylor & Francis Group/Lawrence Erlbaum Associates; 2008. pp. 171–91	2
234	Meyer J. *Maternal Anger, Toddler Attachment, and Maternal Separation Anxiety.* PhD thesis. New York, NY: Yeshiva University; 1992	NF
235	Mikhail MS, Freda MC, Merkatz RB, Polizzotto R, Mazloom E, Merkatz IR. The effect of fetal movement counting on maternal attachment to fetus. *Am J Obstet Gynecol* 1991;**165**:988–91	1
236	Mikulincer M, Florian V. Maternal–fetal bonding, coping strategies, and mental health during pregnancy – the contribution of attachment style. *J Soc Clin Psychol* 1999;**18**:255–76	4
237	Miljkovitch R, Pierrehumbert B. From behavioral strategies of attachment to representational strategies: construction and validity of the coding system for story stems. *Enfance* 2008;**60**:22–30	2
238	Miller WB, Sable MR, Csizmadia A. Pregnancy wantedness and child attachment security: is there a relationship? *Maternal and Child Health Journal* 2008;**12**:478–87	1
239	Minnis H, Bryee G. Maltreated children: finding the right attachment relationship. *Educ Child Psychol* 2010;**27**:51–8	4
240	Minnis H. How can foster carers help children with complex mental health and attachment problems? *Int J Child Fam Welfare* 2004;**7**:162–7	4
241	Minnis H. Reactive attachment disorder. *J Am Acad Child Adolesc Psychiatry* 2001;**40**:129–33	1
242	Mizukami K, Kobayashi N, Iwata H, Ishii T. Telethermography in Measurement of Infants Early Attachment. In von Euler C, Forssberg H, Lagercrantz H. *Neurobiology of Early Infant Behaviour. Wenner-Gren Center International Symposium Series. 55*. New York, NY: Stockton Press; 1989. pp. 49–59	1
243	Moffatt MJ Jr. The affective bond between preschool-aged children and mentors. New York, NY: New School For Social Research. *Dissertation Abstracts International: Section B: The Sciences and Engineering*; 2000	1
244	Morcuende MA, Troutman B. Circle of Security, individual protocol: An intervention for the intergenerational transmission of psychopathology. Clinical and Translational Science. 2010. In Clinical and Translational Research and Education Meeting: ACRT/SCTS Joint Annual Meeting; Washington, DC: Clinical and Translational Science; 2010. p. 60	4
245	Morgan FT. Development of a measure to screen for reactive attachment disorder in children 0–5 years. Fresno, CA: California School of Professional Psychology, Alliant International University. *Dissertation Abstracts International: Section B: The Sciences and Engineering;* 2005	4
246	Morisset CE. Environmental influences on language development of high social-risk toddlers. Seattle, WA: Univeristy of Washington. *Dissertation Abstracts International*; 1991	NF
247	Morris DL. Infant attachment and problem-solving in the toddler: relations to mother's family history. Minneapolis, MN; University of Minnesota. *Dissertation Abstracts International*; 1980	NF
248	Muller ME. *The Development and Testing of the Muller Prenatal Attachment Inventory.* PhD thesis. San Francisco, CA: University of California; 1990	4
249	Murray L, Cooper PJ. Clinical Applications of Attachment Theory and Research: Change in Infant Attachment with Brief Psychotherapy; 1991. In Richer J, editor. *The Clinical Application of Ethology and Attachment Theory*. Oxford: ACPP; 1994	4
250	Murray MP. *Attachment Relationships and Empathic Response in Young People who Sexually Abuse*. PhD thesis. Belfast: Queen's University Belfast; 2002	4

Number	Reference	Reason
251	Myeroff RL. *Comparative Effectiveness of Attachment Therapy with the Special Needs Adoptive Population*. PhD thesis. Brattleboro, VT: Union Institute & University; 1997	4
252	Narita S, Maehara S. The development of maternal-fetal attachment during pregnancy. *Nihon Kango Kagakkaishi* 1993;**13**:1–9	1
253	Neander K, Engstrom I. Parents' assessment of parent–child interaction interventions – a longitudinal study in 101 families. *Child Adolesc Psychiatry Ment Health* 2009;**3**:8	4
254	Neu M, Robinson J. Maternal holding of preterm infants during the early weeks after birth and dyad interaction at six months. *J Obstet Gynecol Neonatal Nurs* 2010;**39**:401–14	2
255	Niccols A, Mohamed S. Parent training in groups: pilot study with parents of infants with developmental delay. *J Early Interv* 2000;**23**:133–43	2
256	Niederhofer H, Reiter A. Does the maternal appraisal of attachment to the child remain stable from the prenatal phase to the postnatal period, and can it be objectively assessed from intrauterine activity? *Psychiatria Danubina* 2002;**14**:35–40	4
257	Nievar MA, Becker BJ. Sensitivity as a privileged predictor of attachment: a second perspective on De Wolff and Van IJzendoorn's meta-analysis. *Social Development* 2008;**17**:102–14	4
258	Nievar MA, Jacobson A, Dier S. *Home Visiting for At-Risk Preschoolers: A Successful Model for Latino Families*. Paper presented at the National Council on Family Relations, Little Rock, AK, 2008	2
259	Norman T. *Treatment of Dysfunctional Adolescents Using Goats to Model Mother-Infant Attachment Behavior*. Palo Alto, CA: Pacific Graduate School of Psychology, Palo Alto University. Dissertation Abstracts International: Section B: The Sciences and Engineering; 2005	4
260	Norr KF, Roberts JE, Freese U. Early postpartum rooming-in and maternal attachment behaviors in a group of medically indigent primiparas. *J Nurs Midwifery* 1989;**34**:85–91	2
261	Norris-Shortle C, Melley AH, Kiser LJ, Levey E, Cosgrove K, Leviton A. Targeted interventions for homeless children at a therapeutic nursery. *Zero to Three J* 2006;**26**:49–55	4
262	Notaro PC, Volling BL. Parental responsiveness and infant-parent attachment: A replication study with fathers and mothers. *Infant Behav Dev* 1999;**22**:345–52	4
263	Olds D. The Prenatal/Early Infancy Project: Fifteen Years Later. In Albee GW, Gullotta TP. *Primary Prevention Works*. Thousand Oaks, CA: Sage Publications, Inc.; 1997. pp. 41–67	4
264	Onyezia NE. *Complex Trauma in the Child Welfare System: Initial Clinical Presentation and Treatment Response*. PhD thesis. Evanston, IL: Northwestern University; 2009	2
265	Oppenheim D, Koren-Karie N, Sagi A. Mothers' empathic understanding of their preschoolers' internal experience: relations with early attachment. *Int J Behav Dev* 2001;**25**:16–26	4
266	Osofsky JD, Kronenberg M, Hammer JH, Lederman JC, Katz L, Adams S, *etal*. The development and evaluation of the intervention model for the Florida Infant Mental Health Pilot Program. *Infant Ment Health J* 2007;**28**:259–80	4
267	Ottaviano CM. *Early Contact and Infant-Mother Attachment at One Year*. Report ED174342. 1979	4
268	Out D, Bakermans-Kranenburg MJ, Van IJzendoorn MH. The role of disconnected and extremely insensitive parenting in the development of disorganized attachment: validation of a new measure. *Attach Hum Dev* 2009;**11**:419–43	4
269	Oxford ML, Harachi TW, Catalano RF, Haggerty KP, Abbott RD. Early elementary school-aged child attachment to parents: a test of theory and implications for intervention. *Prevent Sci* 2000;**1**:61–9	PCO
270	Padron E. *The Disorganized Dyad: The Roles of Mother and Child in the Development of Disorganized Attachment*. Minneapolis, MN; University of Minnesota. Dissertation Abstracts International: Section B: The Sciences and Engineering; 2004	1
271	Pajusco E, editor The role of the attachment theory in understanding 'the need of family' owned by every child and in providing the right answer to this need. Eusarf international conference on assessing the 'evidence-base' of intervention for vulnerable children and their families, 2008, Padua, Italy	4
272	Parisi D, Cecconi F, Cerini A. Kin-Directed Altruism and Attachment Behaviour in an Evolving Population of Neural Networks. In Gilbert N, Conte R, editors. *Artificial Societies: The Computer Simulation of Social Life*. Siena: UCL Press; 1995	4

© Queen's Printer and Controller of HMSO 2015. This work was produced by Wright *et al.* under the terms of a commissioning contract issued by the Secretary of State for Health. This issue may be freely reproduced for the purposes of private research and study and extracts (or indeed, the full report) may be included in professional journals provided that suitable acknowledgement is made and the reproduction is not associated with any form of advertising. Applications for commercial reproduction should be addressed to: NIHR Journals Library, National Institute for Health Research, Evaluation, Trials and Studies Coordinating Centre, Alpha House, University of Southampton Science Park, Southampton SO16 7NS, UK.

Number	Reference	Reason
273	Patino FL. *Mothers' Prenatal Attachment History, Attachment Status, and Social Support Satisfaction as Predictors of Postnatal Maternal Sensitivity.* PhD thesis. San Diego, CA: California School of Professional Psychology; 1993	4
274	Pavill BC. *Father-Infant Attachment in Breastfed versus Bottlefed Infants*. PhD thesis. Scranton, PA: Marywood University; 2002	4
275	Pearson RM, Lightman SL, Evans J. Attentional processing of infant emotion during late pregnancy and mother–infant relations after birth. *Arch Womens Ment Health* 2011;**14**:23–31	4
276	Pechous EA. *Young Children with Autism and Intensive Behavioral Programs: Effects on the Primary Attachment Relationship*. PhD thesis. Fresno, CA: California School of Professional Psychology; 2001	2
277	Perry DF, Ettinger AK, Mendelson T, Le HN. Prenatal depression predicts postpartum maternal attachment in low-income Latina mothers with infants. *Infant Behav Dev* 2011;**34**:339–50	4
278	Philipp DA, Herve MJ, Keren M. Does the portal of entry determine our view? Interfaces between dyadic and three-way assessment of a clinical family transitioning to parenthood. *Infant Ment Health J* 2008;**29**:259–77	2
279	Pierrehumbert B, Muntean A, Tomita M, Ungureanu R, Habersaat S. The significant behavioral aspects of adopted children within successful adoption in Romania. *Rev Cercet Interv Soc* 2011;**32**:107–30	1
280	Pierrehumbert B, Santelices MP, Ibanez M, Alberdi M, Ongari B, Roskam I, *et al.* Gender and attachment representations in the preschool years: comparisons between five countries. *J Cross Cult Psychol* 2009;**40**:543–66	1
281	Pignotti M. Reactive attachment disorder and international adoption: a systematic synthesis. *Sci Rev Ment Health Pract* 2011;**8**:30–49	1
282	Pisciella AE. *Estimating Effects of Participation in Parental Leave on Children's and Mothers' Well-Being*. PhD thesis. New York, NY: Fordham University; 2008	4
283	Pollock PH, Percy A. Maternal antenatal attachment style and potential fetal abuse. *Child Abuse Negl* 1999;**23**:1345–57	4
284	Pool MM, Bijleveld CC, Tavecchio LW. The effect of same-age and mixed-age grouping in day care on parent–child attachment security. *Soc Behav Personality* 2000;**28**:595–602	4
285	Portnoy FC, Simmons CH. Day care and attachment. *Child Dev* 1978;**49**:239–42	1
286	Posada G, Jacobs A. Child-mother attachment relationships and culture. *Am Psychol* 2001;**56**:821–2	1
287	Priel B, Besser A. Bridging the gap between attachment and object relations theories: a study of the transition to motherhood. *Br J Med Psychol* 2001;**74**:85–100	1
288	Propper C, Willoughby M, Halpern CT, Carbone MA, Cox M. Parenting quality, DRD4, and the prediction of externalizing and internalizing behaviors in early childhood. *Dev Psychobiol* 2007;**49**:619–32	1
289	Propper CB. *Infant Behavioral and Physiological Regulation During the Strange Situation Procedure at 12-Months: Contributions of Genotype and Maternal Behavior*. Durham, NC: Duke University. Dissertation Abstracts International: Section B: The Sciences and Engineering; 2007	1
290	Puckering C, Connolly B, Werner C, Toms-Whittle L, Thompson L, Lennox J, et al. Rebuilding relationships: a pilot study of the effectiveness of the Mellow Parenting Programme for children with Reactive Attachment Disorder. *Clin Child Psychol Psychiatry* 2011;**16**:73–87	4
291	Puentes-Neuman G. *Toddlers' Social Coordination with an Unfamiliar Peer: Patternings of Attachment, Temperament, and Coping During Dyadic Exchange.* Montréal: Université de Montréal. Dissertation Abstracts International: Section B: The Sciences and Engineering; 2000	4
292	Pure K. *Instructional Antecedents to Children's Compliant Behaviour*. PhD thesis. Fredericton, NB: University of New Brunswick; 2002	1
293	Purvis KB. *Correlates of Behavioral Change in a Sample of At-Risk Adopted Children: A Preliminary Study*. Fort Worth, TX: Texas Christian University. Dissertation Abstracts International: Section B: The Sciences and Engineering; 2004	2
294	Raaska H, Sinkkonen J, Lapinleimu H, Makipaa S, Elovainio M. Attachment problems among internationally adopted toddlers in Finland; the FINADO-study. 14th International Congress of ESCAP European Society for Child and Adolescent Psychiatry, June 2011, Helsinki, Finland	4

Number	Reference	Reason
295	Radtke LT. *Attachment, Parenting Behaviors, and Negative Affect: Are they Related to Effortful Control at Age 4?* PhD thesis. Chicago, IL: Illinois Institute of Technology; 2009	1
296	Ramos-Marcuse FM. *Children's Moral Narratives: Links to Aggression and Early Relationships.* PhD thesis. Yeshiva University; 2002	4
297	Reading AE, Cox DN, Sledmere CM, Campbell S. Psychological changes over the course of pregnancy: a study of attitudes toward the fetus/neonate. *Health Psychol* 1984;**3**:211–21	1
298	Reardon PA. *A Primary Care Setting Screening for Risk Factors to Attachment Security.* PhD thesis. Antioch University/New England Graduate School; 2001	1
300	Reichert J, Zollner N, Steinhardt A, Gurth H, Burkhardt W, Rudiger M. The "Familienetz"– psychosocial support for parents of preterm infants. 19th European Workshop on Neonatology La Granja de San Ildefonso, Segovia, Spain. *J Neonat Perinat Med* 2011	NF
301	Reid MJ, Webster-Stratton C, Hammond M. Enhancing a classroom social competence and problem-solving curriculum by offering parent training to families of moderate- to high-risk elementary school children. *J Clin Child Adolesc Psychol* 2007;**36**:605–20	4
302	Rellinger EA. *Precursors and Consequences of Insecure Attachment in Children of Adolescent Mothers*. PhD thesis. South Bend, IN: University of Notre Dame; 1995	2
303	Rembert C. *Female adolescent ego development and how it is related to attachment and depression: A longitudinal study*. New York, NY: Pace University. Dissertation Abstracts International: Section B: The Sciences and Engineering; 2007	4
304	Renaud M. Evaluation of a Structured Programme of a Group Intervention with Families Experiencing Parenting Difficulties. 5th World Congress on Innovations in Psychiatry, 19–22 May 1998, London, UK	1
305	Righetti P, Dell'Avanzo M, Grigio M, Nicolini U. Maternal/paternal antenatal attachment and fourth-dimensional ultrasound technique: a preliminary report. *Br J Psychol* 2005;**96**:129–37	4
306	Rimashevskaia NV, Kremneva LF. Mental development of young children in maternal behavior disorders. *Zh Nevrol Psikhiatr Im S S Korsakova* 2003;**103**:19–24	2
307	Rinich E, Drotar D, Brinish P. Security of attachment and outcome of preschoolers with histories of nonorganic failure to thrive. *J Clin Child Psychol* 1989;**18**:142–52	4
308	Roberto A, Carlyle K, Goodall C, Castle J. The relationship between parents' verbal aggressiveness and responsiveness and young adult children's attachment style and relational satisfaction with parents. *J Fam Commun* 2009;**9**:90–106	4
309	Robson K, Tooby A. Play therapy with looked after children: an attachment perspective. *Br J Play Ther* 2004;**1**:16–25	1
310	Rodning C, Beckwith L, Howard J. Prenatal exposure to drugs and its influence on attachment. *Ann N Y Acad Sci* 1989;**562**:352–4	4
311	Rodrigues AR, Perez-Lopez J, de la Nuez AG. Prenatal attachment and anxiety during the final trimester of pregnancy in early parenthood: a preliminary study. *Anales de Psicologia* 2004;**20**:95–102	1
312	Roehrig C, Genet C, Cyrulnik B. An ethological observation: styles of attachment and evocation of pregnancy during adolescence. *Sexologies* 2006;**15**:134–41	4
313	Roelofs J, Meesters C, ter Huurne M, Bamelis L, Muris P. On the links between attachment style, parental rearing behaviors, and Internalizing and Externalizing Problems in Non-Clinical Children. *Journal of Child and Family Studies* 2006;**15**:331–44	3A
314	Roggman LA, Boyce LK, Cook GA, Cook J. Getting dads involved: predictors of father involvement in Early Head Start and with their children. *Infant Ment Health J* 2002;**23**:62–78	PCO
315	Roisman GI, Fraley R, Belsky J. A taxometric study of the Adult Attachment Interview. *Dev Psychol* 2007;**43**:675–86	1
316	Roopnarine JL, Lamb ME. The effects of day care on attachment and exploratory behavior in a strange situation. *Merrill-Palmer Q* 1978;**24**:85–95	4
317	Royal College of Psychiatrists. *Attachment Disorders. Part 2 The Evidence*. London: Royal College of Psychiatrists; 2002. pp. 153–6	1

© Queen's Printer and Controller of HMSO 2015. This work was produced by Wright *et al.* under the terms of a commissioning contract issued by the Secretary of State for Health. This issue may be freely reproduced for the purposes of private research and study and extracts (or indeed, the full report) may be included in professional journals provided that suitable acknowledgement is made and the reproduction is not associated with any form of advertising. Applications for commercial reproduction should be addressed to: NIHR Journals Library, National Institute for Health Research, Evaluation, Trials and Studies Coordinating Centre, Alpha House, University of Southampton Science Park, Southampton SO16 7NS, UK.

Number	Reference	Reason
318	Sadler LS. *Minding the Baby: Home Visiting Program Evaluation*. New Haven, CT: National Institute of Child Health and Human Development (NICHD), School of Nursing Yale University; ongoing	4
319	Sadler LS, Slade A, Mayes LC. Minding the Baby: A Mentalization-Based Parenting Program. In Allen JG, Fonagy P. *The Handbook of Mentalization-Based Treatment*. Hoboken, NJ: John Wiley & Sons Inc.; 2006. pp. 271–88	4
320	Salo S. Does Theraplay increase emotional availability among substance-abusing mothers and their infants? 14th International Congress of ESCAP European Society for Child and Adolescent Psychiatry, 2011, Helsinki Finland	4
321	Sapp MS. Relationship of maternal depression to disorganized attachment in latency-age children. New York, NY: The New School. *Dissertation Abstracts International: Section B: The Sciences and Engineering*; 2004	4
322	Sazvar S, Ezabadi Z, Omani Samani R, Eshrati B. Validation of Iranian version of the prenatal attachment inventory. 20th World Congress on Fertility and Sterility, IFFS, 2010, Munich, Germany. *J Reproduktionsmedizin Endokrinologie* 2010	NF
323	Scharfe E. Cause or consequence?: Exploring causal links between attachment and depression. *J Soc Clin Psychology* 2007;**26**:1048–64	1
324	Schechter DS, Willheim E. When parenting becomes unthinkable: intervening with traumatized parents and their toddlers. *J Am Acad Child Adolesc Psychiatry* 2009;**48**:249–53	1
325	Schiltz-Day SK. *The Impact of Enhancing a Behavioral Program for Children with Severe Behavioral Disorders by Promoting Parents' Coherent Life Story*. PhD thesis. Ames, IA: Iowa State University; 1997	4
326	Schwark B, Schmidt S, Strauss B. A study of the relationship between attachment patterns and problem perception in a sample of 9–11-year-old children with behavioral disorders. *Praxis der Kinderpsychologie und Kinderpsychiatrie* 2000;**49**:340–50	4
327	Schwerdtfeger KL, Goff BS. Intergenerational transmission of trauma: exploring mother–infant prenatal attachment. *J Trauma Stress* 2007;**20**:39–51	2
328	Seimyr L, Sjogren B, Welles-Nystrom B, Nissen E. Antenatal maternal depressive mood and parental-fetal attachment at the end of pregnancy. *Arch Womens Ment Health* 2009;**12**:269–79	4
329	Serafica FC. *Effects of Illumination on Attachment Behaviors in a Novel Environment*. Report number ED089885. 1973; p. 11	1
330	US Department of Health & Human Services. *Building their Futures: How Early Head Start Programs are Enhancing the Lives of Infants and Toddlers in Low-Income Families*. US Department of Health & Human Services; 2001	1
331	Shechtman Z, Dvir V. Attachment style as a predictor of behavior in group counseling with preadolescents. *Group Dyn-Theor Res* 2006;**10**:29–42	1
332	Sheridan SM, Knoche LL, Edwards CP, Bovaird JA, Kupzyk KA. Parent engagement and school readiness: effects of the Getting Ready Intervention on preschool children's social-emotional competencies. *Early Educ Dev* 2010;**21**:125–56	2
333	Shieh C, Kravitz M. Severity of drug use, initiation of prenatal care, and maternal-fetal attachment in pregnant marijuana and cocaine/heroin users. *J Obstet Gynecol Neonatal Nurs* 2006;**35**:499–508	4
334	Shieh W-H. Maternal-fetal attachment in illicit-drug-using pregnant women. New Haven, CT: Yale University. *Dissertation Abstracts International: Section B: The Sciences and Engineering*; 1999	1
335	Siddiqui A, Eisemann M, Hagglof B. The stability of maternal interpretation of infant's facial expressions during pre- and postnatal period and its relation to prenatal attachment. *Early Child Dev Care* 2000;**162**:41–50	4
336	Siddiqui A, Hagglof B, Eisemann M. An exploration of prenatal attachment in Swedish expectant women. *J Reprod Infant Psychol* 1999;**17**:369–80	1
337	Siddiqui A, Hagglof B. Does maternal prenatal attachment predict postnatal mother–infant interaction? *Early Hum Dev* 2000;**59**:13–25	1
338	Sideridis GD, Kafetsios K. Perceived parental bonding, fear of failure and stress during class presentations. *Int J Behavioral Dev* 2008;**32**:119–30	4

Number	Reference	Reason
339	Siegel E, Bauman KE, Schaefer ES, Saunders MM, Ingram DD. Hospital and home support during infancy: impact on maternal attachment, child abuse and neglect, and health care utilization. *Pediatrics* 1980;**66**:183–90	1
340	Siegel E, Gillings D, Campbell S, Guild P. Controlled evaluation of rural regional perinatal care: developmental and neurologic outcomes at 1 year. *Pediatrics* 1986;**77**:187–95	4
341	Slade P, Laxton-Kane M, Spiby H. Smoking in pregnancy: the role of the transtheoretical model and the mother's attachment to the fetus. *Addict Behav* 2006;**31**:743–57	2
342	Smeekens S, Riksen-Walraven J, van Bakel HJ. Multiple determinants of externalizing behavior in 5-year-olds: a longitudinal model. *J Abnorm Child Psychol* 2007;**35**:347–61	2
343	Smith JC, Cumming A, Xeros-Constantinides S. A decade of parent and infant relationship support group therapy programs. *Int J Group Psychother* 2010;**60**:59–90	1
344	Smith KE, Landry SH, Swank PR. The influence of decreased parental resources on the efficacy of a responsive parenting intervention. *J Consult Clin Psychol* 2005;**73**:711–20	1
345	Smith PB. *Infant-Mother Attachment as a Function of Maternal Sensitivity, Cognition, Personality, and Social Support*. PhD thesis. London, ON: University of Western Ontario; 1988	4
346	Smolen AG. Boys only! No mothers allowed. *Int J Psychoanal* 2009;**90**:1–11	2
347	Smyke AT, Dumitrescu A, Zeanah CH. Attachment disturbances in young children. I: The continuum of caretaking casualty. *J Am Acad Child Adolesc Psychiatry* 2002;**41**:972–82	1
348	Smyke AT, Zeanah CH, Fox NA, Nelson CA, Guthrie D. Placement in foster care enhances quality of attachment among young institutionalized children. *Child Dev* 2010;**81**:212–23	4
349	Sneddon H, Ferriter M, Macdonald G. CBT interventions for young people aged 10–18 who sexually offend. *Cochrane Database Syst Rev* 2010;**5**:CD009829	1
350	Sokol-Katz JS. *The Relationships between Parental Attachment/Family Structure and Deviant Behavior: A Test of the Social Control Theory.* Dissertation Abstracts International. 1993	1
351	Spangler G, Grossmann K. Biobehavioral organization in securely and insecurely attached infants. *Child Dev* 1993;**64**:1439–50	2
352	Speltz ML, DeKlyen M, Greenberg MT. Attachment in boys with early onset conduct problems. *Dev Psychopathol* 1999;**11**:269–85	4
353	Spira A, Scippa L, Berthet C, Meuret C, Besozzi R, Cramer B. Hospitalism in the year 2000. The psychological development of children in a Romanian orphanage. *Psychiatrie de l'Enfant* 2000;**43**:587–646	4
354	Spitzer S. *Maternal Distress Regulation and Dyadic Repair: Contributions to Infant Socio-Emotional Functioning*. PhD thesis. New York, NY: City University; 2001	4
355	Spletzer PU. Mutual regulation: the relationship between maternal-fetal attachment, maternal prenatal sleep, and postnatal infant sleep patterns. Carpinteria, CA: Pacifica Graduate Institute. *Dissertation Abstracts International: Section B: The Sciences and Engineering*; 2006	4
356	St. Petersburg USA Orphanage Research Team. The effects of early social-emotional and relationship experience on the development of young orphanage children. *Monogr Soc Res Child Dev* 2008;**73**:vii–295	1
357	Stainton MC. *Origins of Attachment: Culture and Cue Sensitivity*. PhD thesis. San Francisco, CA: University of California; 1986	2
358	Stams G, Juffer F, Rispens J, Hoksbergen RAC. The development and adjustment of 7-year-old children adopted in infancy. *J Child Psychol Psychiatry* 2000;**41**:1025–37	1
359	Stams G-JJ, Juffer F, Van IJzendoorn MH, Hocksbergen RC. Attachment-based intervention in adoptive families in infancy and children's development at age 7: two follow-up studies. *Br J Dev Psychol* 2001;**19**:159–80	4
360	Stanford DD. *The Effect of Fetal Ultrasound Imaging on Parental-Fetal Attachment and on Dyadic Attachment of the Expectant Couple*. PhD thesis. Berkeley/Alameda, CA: California School of Professional Psychology; 2002	2
361	Stayton DJ, Salter Ainsworth MD. *Individual Differences in Infant Responses to Brief, Everyday Separations as Related to Other Infant and Maternal Behaviors.* Report number ED069438. 1973	4

© Queen's Printer and Controller of HMSO 2015. This work was produced by Wright *et al.* under the terms of a commissioning contract issued by the Secretary of State for Health. This issue may be freely reproduced for the purposes of private research and study and extracts (or indeed, the full report) may be included in professional journals provided that suitable acknowledgement is made and the reproduction is not associated with any form of advertising. Applications for commercial reproduction should be addressed to: NIHR Journals Library, National Institute for Health Research, Evaluation, Trials and Studies Coordinating Centre, Alpha House, University of Southampton Science Park, Southampton SO16 7NS, UK.

Number	Reference	Reason
362	Steele M, Murphy A, Steele H. Identifying therapeutic action in an attachment-centered intervention with high risk families. *Clin Soc Work J* 2010;**38**:61–72	4
363	Sterkenburg P, Janssen C, Schuengel C. The effect of an attachment-based behaviour therapy for children with visual and severe intellectual disabilities. *J Appl Res Intellect Dis* 2008;**21**:126–35	4
364	Sterkenburg PS, Schuengel PC, Janssen CGC. Fostering secure attachment during psychotherapy for children with severe ID and visually impairment. *J Intellect Dis Res* 2008;**52**:726	4
365	Storebo OJ, Pedersen J, Skoog M, Thomsen PH, Gluud C, Simonsen E. Social-Skills Training and Parental Training Plus Standard Treatment Versus Standard Treatment of Children with Attention-Deficit Hyperactivity Disorder: The Sostra Randomised Trial Protocol. Eunethydis 1st International ADHD Conference: From Data to Best Clinical Practice, 2012, Amsterdam Netherlands	4
366	Stubenbort K, Cohen MM, Trybalski V. The effectiveness of an attachment-focused treatment model in a therapeutic preschool for abused children. *Clin Soc Work J* 2010;**38**:51–60	NF
367	Suchman N, DeCoste C, Castiglioni N, Legow N, Mayes L. The mothers and toddlers program: preliminary findings from an attachment-based parenting intervention for substance-abusing mothers. *Psychoanal Psychol* 2008;**25**:499–517	2
368	Suchman N, Decoste C, Leigh D, Borelli, J. Reflective functioning in mothers with drug use disorders: implications for dyadic interactions with infants and toddlers. *Attach Hum Dev* 2012;**12**:567–85	2
369	Suchman NE, DeCoste C, Castiglioni N, McMahon TJ, Rounsaville B, Mayes L. The mothers and toddlers program, an attachment-based parenting intervention for substance using women: post-treatment results from a randomized clinical pilot. *Attach Hum Dev* 2010;**12**:483–504	4
370	Suchman NE, Decoste C, McMahon TJ, Rounsaville B, Mayes L. The mothers and toddlers program, an attachment-based parenting intervention for substance-using women: results at 6-week follow-up in a randomized clinical pilot. *Infant Ment Health J* 2011;**32**:427–49	4
371	Suchman NE, DeCoste C, Rosenberger P, McMahon TJ. Attachment-based intervention for substance using mothers: a preliminary test of the proposed mechanisms of change. *Infant Ment Health J* 2012;**33**	2
372	Suess GJ, Bohlen U, Mali A, Maier MF. Preliminary results concerning the effectiveness of early intervention from the STEEP practice research project 'WiEge'. *Bundesgesundheitsblatt Gesundheitsforschung Gesundheitsschutz* 2010;**53**:1143–9	2
373	Sumpter JM. Reactive Attachment Disorder: Developing a Developmental Perspective. In 13th Annual CECP Research Exchange Conference, Marquette University, Milwaukee, 2011	2
374	Svanberg P, Mennet L, Spieker S. Promoting a secure attachment: a primary prevention practice model. *Clin Child Psychol Psychiatry* 2010;**15**:363–78	4
375	Szajnberg N, Wilson ME, Beauchaine TP, Waters E. Mothers of children with inflammatory bowel disease: a controlled study of adult attachment classifications and patterns of psychopathology. *Isr J Psychiatry Relat Sci* 2011;**48**:34–4	4
376	Szajnberg NM, Skrinjaric J, Moore A. Affect attunement, attachment, temperament, and zygosity: a twin study. *J Am Acad Child Adolesc Psychiatry* 1989;**28**:249–53	4
377	Tambelli R, Odorisio F, Notari V. Maternal attachment security and compensative intervention in risk context. *Eta Evolutiva* 2010;**96**:89–103	2
378	Tarabulsy GM, Robitaille J, Lacharite C, Deslandes J, Coderre R. Intervention for young mothers and their infants: an attachment theory perspective. *Criminologie* 1998;**31**:7–23	4
379	Taylor A, Atkins R, Kumar R, Adams D, Glover V. A new Mother-to-Infant Bonding Scale: links with early maternal mood. *Arch WomensMent Health* 2005;**8**:45–51	2
380	Tessier R, Cristo M, Velez S, Giron M, de Calume ZF, Ruiz-Palaez JG, *et al.* Kangaroo mother care and the bonding hypothesis. *Pediatrics* 1998;**102**:e17	4
381	Teti DM, Killeen LA, Candelaria M, Miller W, Hess CR, O'Connell M. Adult Attachment, Parental Commitment to Early Intervention, and Developmental Outcomes in an African American Sample. In Steele H, Steele M. *Clinical Applications of the Adult Attachment Interview*. New York, NY: Guilford Press; 2008. pp. 126–53	4

Number	Reference	Reason
382	Teti DM. Maternal Depression and Child-Mother Attachment in the First Three Years: A View from the Intermountain West. In Crittenden PM, Claussen AH. *The Organization of Attachment Relationships: Maturation, Culture, and Context*. New York, NY: Cambridge University Press; 2000. pp. 190–213	2
383	The St. Petersburg-USA Orphanage Research Team. XI. Intervention effects on caregiver–child interactions (infant affect manual, attachment variables). The effects of early social-emotional and relationship experience on the development of young orphanage children. *Monogr Soc Res Child Dev* 2008;**73**:187–223	4
384	Thomas R, Zimmer-Gembeck MJ. Accumulating evidence for parent–child interaction therapy in the prevention of child maltreatment. *ChildDev* 2011;**82**:177–92	4
385	Tilbrook DMW. *Attachment, Conduct Disorder and Perspective Taking in 7–9 Year Old Boys*. PhD thesis. Open University; 2000	4
386	Torres B, Alonso-Arbiol I, Cantero MJ, Abubakar A. Infant-mother attachment can be improved through group intervention: a preliminary evaluation in Spain in a non-randomized controlled trial. *Span J Psychol* 2011;**14**:630–8	2
387	Toth SL, Rogosch FA, Cicchetti D. Attachment-Theory-Informed Intervention and Reflective Functioning in Depressed Mothers. In Steele H, Steele M. *Clinical Applications of the Adult Attachment Interview*. New York, NY: Guilford Press; 2008. pp. 154–72	4
388	Tsartsara E, Johnson MP. The impact of miscarriage on women's pregnancy-specific anxiety and feelings of prenatal maternal-fetal attachment during the course of a subsequent pregnancy: an exploratory follow-up study. *J Psychosom Obstet Gynecol* 2006;**27**:173–82	4
389	Twemlow SW, Fonagy P, Sacco FC. A developmental approach to mentalizing communities: the Peaceful Schools Experiment. Part 2. *Pszichoterapia* 2009;**18**:338–45	4
390	Van den Boom DC. Intervention with irritable children. *Psycholoog* 1991;**26**:7–13	4
391	van der Mark IL, Bakermans-Kranenburg MJ, Van IJzendoorn MH. The role of parenting, attachment, and temperamental fearfulness in the prediction of compliance in toddler girls. *Br J Dev Psychol* 2002;**20**:361–78	4
392	Van IJzendoorn MH, Bakermans-Kranenburg MJ, Juffer F. Why Less Is More: From the Dodo Bird Verdict to Evidence-Based Interventions on Sensitivity and Early Attachments. In Berlin LJ. *Enhancing Early Attachments: Theory, Research, Intervention, and Policy*. New York, NY: Guilford Press; 2005. pp. 297–312	PCO
393	Van IJzendoorn MH, De Wolff MS. In search of the absent father–meta-analyses of infant-father attachment: a rejoinder to our discussants. *Child Dev* 1997;**68**:604–9	1
394	Van IJzendoorn MH, Juffer F. The Emanuel Miller Memorial Lecture 2006: Adoption as intervention. Meta-analytic evidence for massive catch-up and plasticity in physical, socio-emotional, and cognitive development. *J Child Psychol Psychiatry* 2006;**47**:1228–45	4
395	Van Zeijl J, Mesman J, Van IJzendoorn MH, Bakermans-Kranenburg MJ, Juffer F, Stolk MN, *et al.* Attachment-based intervention for enhancing sensitive discipline in mothers of 1- to 3-year-old children at risk for externalizing behavior problems: a randomized controlled trial. *J Consulting Clin Psychol* 2006;**74**:994–1005	4
396	Venet M, Bureau J-F, Gosselin C, Capuano F. Attachment representations in a sample of neglected preschool-age children. *School Psychol Int* 2007;**28**:264–93	2
397	Vorria P, Papaligoura Z, Sarafidou J, Kopakaki M, Dunn J, Van IJzendoorn MH, et al. The development of adopted children after institutional care: a follow-up study. *J Child Psychol Psychiatry* 2006;**47**:1246–53	2
398	Wachter MPK. *Psychological Distress and Dyadic Satisfaction as Predictors of Maternal-Fetal Attachment*. PhD thesis. Chicago, IL: Illinois Institute of Technology; 2002	4
399	Wadsby M, Arvidsson E. Eight years after – a follow-up study of mothers and children at psychosocial risk who received early treatment: does early intervention leave its mark? *Child Fam Social Work* 2010;**15**:452–60	1
400	Walsh AP. Representations of attachment and caregiving: the disruptive effects of loss and trauma. Charlottesville, VA: University of Virginia. *Dissertation Abstracts International: Section B: The Sciences and Engineering*; 2003	2

© Queen's Printer and Controller of HMSO 2015. This work was produced by Wright *et al.* under the terms of a commissioning contract issued by the Secretary of State for Health. This issue may be freely reproduced for the purposes of private research and study and extracts (or indeed, the full report) may be included in professional journals provided that suitable acknowledgement is made and the reproduction is not associated with any form of advertising. Applications for commercial reproduction should be addressed to: NIHR Journals Library, National Institute for Health Research, Evaluation, Trials and Studies Coordinating Centre, Alpha House, University of Southampton Science Park, Southampton SO16 7NS, UK.

Number	Reference	Reason
401	Wan MW, Warburton AL, Appleby L, Abel KM. Mother and baby unit admissions: feasibility study examining child outcomes 4–6 years on. *Aust N Z J Psychiatry* 2007;**41**:150–6	2
402	Weinberg HA. Improved functioning in children diagnosed with reactive attachment disorder after SSRI therapy. *J Can Acad Child Adolesc Psychiatry* 2010;**19**:48–50	1
403	Wendland-Carro J, Piccinini CA, Millar WS. The role of an early intervention on enhancing the quality of mother–infant interaction. *Child Dev* 1999;**70**:713–21	4
404	Whalen JW. Mothers' attributions of control and children's attachments in a prison sample. Alameda, CA: California School of Professional Psychology-Berkeley. *Dissertation Abstracts International*; 1990	4
405	Wheeler DJ. Fantasy proneness and attachment style: a mediation model of the development of psychopathology. Fairfax, VA: George Mason University; *Dissertation Abstracts International: Section B: The Sciences and Engineering*; 1999	2
406	White O, McCorry NK, Scott-Heyes G, Dempster M, Manderson J. Maternal appraisals of risk, coping and prenatal attachment among women hospitalised with pregnancy complications. *J Reprod Infant Psychol* 2008;**26**:74–85	NF
407	Widera-Wysoczanska A. Complex childhood abuse, attachment disorders and health problems. *J Psychosom Res* 2004;**56**:670	1
408	Wiggins TL, Sofronoff K, Sanders MR. Pathways Triple P-Positive Parenting Program: effects on parent–child relationships and child behavior problems. *Family Process* 2009;**48**:517–30	4
409	Willcox E. Reactive attachment disorder in children. *Paediatr Nurs* 1995;**7**:14–6	1
410	Wimmer JS, Elizabeth Vonk M, Reeves PM. Adoptive mothers' perceptions of reactive attachment disorder therapy and its impact on family functioning. *Clin Soc Work J* 2010;**38**:120–31	2
411	Wise S, Grossman FK. Adolescent mothers and their infants: psychological factors in early attachment and interaction. *Am J Orthopsychiatry* 1980;**50**:454–68	4
412	Zelazo P. *Fathers and Sons: An Experimental Facilitation of Attachment Behaviors*. Report number ED139546. 1977	4
413	Ziegenhain U, Derksen B, Dreisorner R. Young parenthood: adolescent mothers and their children. *Monatsschrift Kingerheilkunde* 2003;**151**:608–12	4
414	Ziegenhain U, Mueller B, Rauh H. *Early Attachment Experiences and Distinctive Behavioral Features of Small Children in a Social and Cognitive Demand Situation.* PhD thesis. Fachinformationszentrum Karlsruhe TIB – Technische Informationsbibliothek; 1995	1

Appendix 6 Meta-analysis of studies seeking to establish secure attachment patterns

Thirteen studies (17 interventions)[129,133,180,189–191,193–200,202–204,208,209,218] were included that reported interventions to promote a 'secure' outcome where this was measured using a validated instrument. Two studies[129,133,203,204] had two interventions, and Murray *et al.*[208] and Cooper *et al.*[209] had three interventions. These have been reported as separate studies.

Barnett[129,133] included the following interventions:

1. home visits with social workers – professional intervention (referred to in meta-analysis as 'Prof')
2. home visits with experienced mothers – non-professional intervention (referred to as 'Non Prof').

Klein-Velderman[203,204] included the following interventions:

1. written information about sensitive parenting information and personal video feedback (referred to as 'VIPP')
2. written information about sensitive parenting information and personal video feedback with additional discussions about early attachment experiences (referred to as 'VIPP-R') labelled as + D for + Discussions.

Murray *et al.*[208] and Cooper *et al.*[209] included the following interventions:

1. cognitive–behavioural therapy (referred to as 'CBT')
2. psychodynamic therapy (referred to as 'Psy')
3. counselling (referred to as 'Cou').

The funnel plot is roughly symmetrical, indicating that publication bias is not likely to be present.

© Queen's Printer and Controller of HMSO 2015. This work was produced by Wright *et al.* under the terms of a commissioning contract issued by the Secretary of State for Health. This issue may be freely reproduced for the purposes of private research and study and extracts (or indeed, the full report) may be included in professional journals provided that suitable acknowledgement is made and the reproduction is not associated with any form of advertising. Applications for commercial reproduction should be addressed to: NIHR Journals Library, National Institute for Health Research, Evaluation, Trials and Studies Coordinating Centre, Alpha House, University of Southampton Science Park, Southampton SO16 7NS, UK.

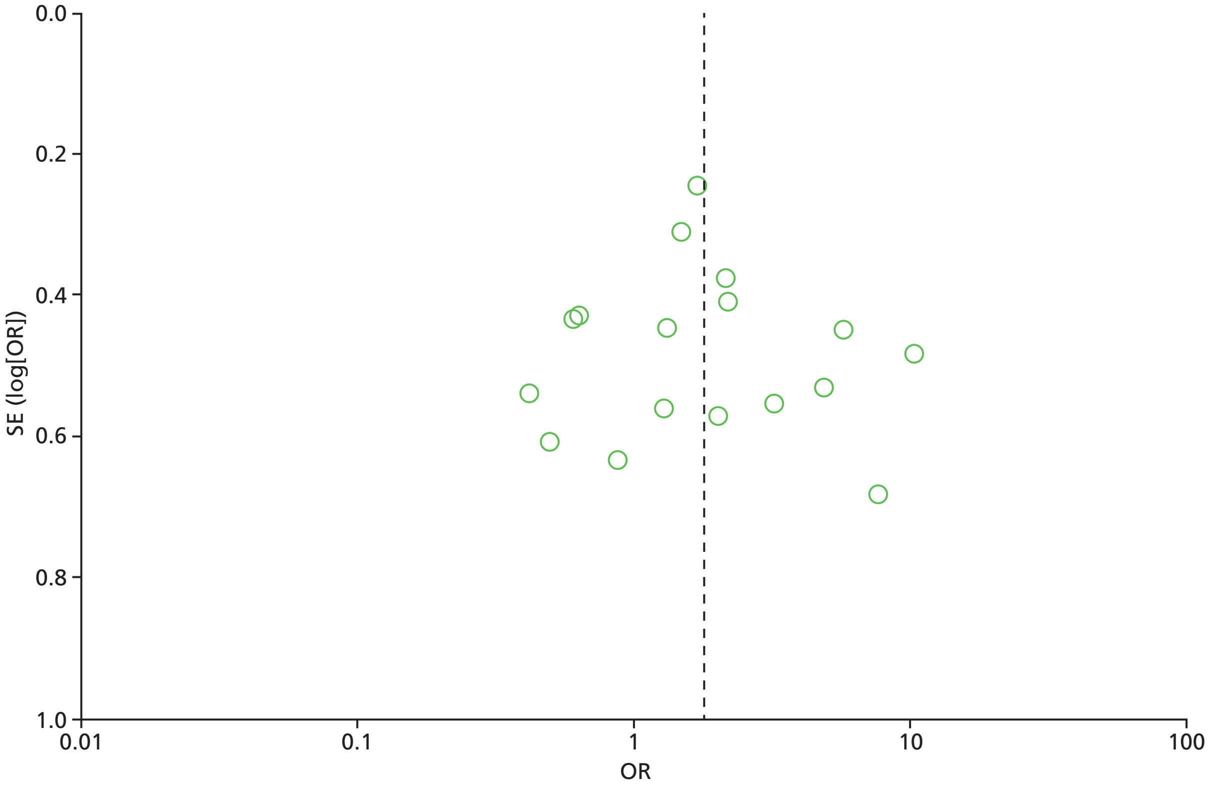

FIGURE 24 Forest plot for secure outcomes for all 17 interventions in the included studies.

A random-effects model was used. Overall, the intervention resulted in increased secure behaviour (OR 1.83, 95% CI 1.26 to 2.66), compared with the control group ($p = 0.0002$).

Study or subgroup	Parental intervention Events	Parental intervention Total	Control Events	Control Total	Weight	OR M–H, random, 95% CI
Anisfeld 1990[180]	19	23	10	26	4.5%	7.60 (2.00 to 28.93)
Barnett (NonP) 1985,[133] 1987[129]	20	28	17	23	4.8%	0.88 (0.26 to 3.05)
Barnett (Prof) 1985,[133] 1987[129]	17	29	17	23	5.0%	0.50 (0.15 to 1.64)
Brisch 2003[202]	19	32	28	36	5.5%	0.42 (0.15 to 1.20)
Cassidy 2011[196]	51	85	42	84	7.3%	1.50 (0.82 to 2.76)
Cicchetti 1999,[200] Toth 2006[199]	31	46	9	54	5.9%	10.33 (4.02 to 26.57)
Cooper 2009[189]	116	156	102	162	7.7%	1.71 (1.06 to 2.76)
Dozier 2009,[194] Bernard 2012[193]	31	60	20	60	6.7%	2.14 (1.02 to 4.47)
Heinicke 1999,[191] 2001[190]	24	31	17	33	5.3%	3.23 (1.09 to 9.54)
Klein-Velderman (+D) 2006[203]	16	26	15	27	5.3%	1.28 (0.43 to 3.83)
Klein-Velderman 2006[204]	20	28	15	27	5.2%	2.00 (0.65 to 6.11)
Moran 2005[218]	28	49	19	50	6.5%	2.18 (0.97 to 4.86)
Moss 2011[195]	23	35	9	32	5.5%	4.90 (1.73 to 13.85)
Murray,[208] Cooper (CBT) 2003[209]	19	41	27	47	6.3%	0.64 (0.28 to 1.49)
Murray,[208] Cooper (Cou) 2003[209]	25	39	27	47	6.2%	1.32 (0.55 to 3.17)
Murray,[208] Cooper (Psy) 2003[209]	18	40	27	47	6.3%	0.61 (0.26 to 1.42)
van den Boom 1994,[198] 1995[197]	31	50	11	50	6.2%	5.78 (2.40 to 13.94)
**Total (95% CI)**		**798**		**828**	**100.0%**	**1.78 (1.19 to 2.67)**
Total events	508		412			

Heterogeneity: $\tau^2=0.49$; $\chi^2=55.85$, df = 16 ($p=0.00001$); $I^2=71\%$

Test for overall effect: $z=2.80$ ($p<0.005$)

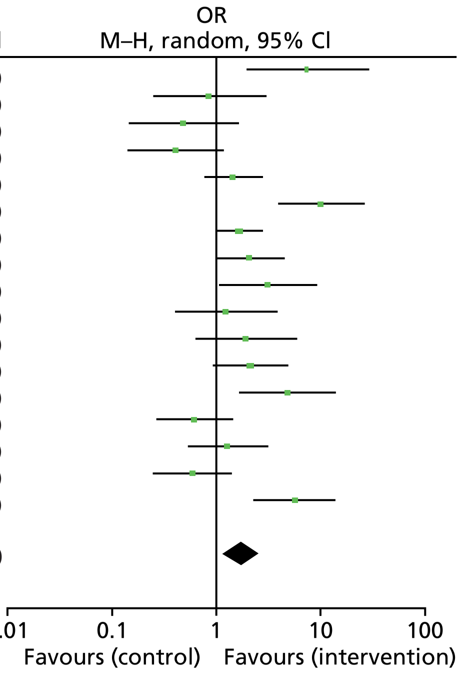

FIGURE 25 A meta-analysis of changes in secure outcomes comparing parental intervention to a control condition.

© Queen's Printer and Controller of HMSO 2015. This work was produced by Wright *et al.* under the terms of a commissioning contract issued by the Secretary of State for Health. This issue may be freely reproduced for the purposes of private research and study and extracts (or indeed, the full report) may be included in professional journals provided that suitable acknowledgement is made and the reproduction is not associated with any form of advertising. Applications for commercial reproduction should be addressed to: NIHR Journals Library, National Institute for Health Research, Evaluation, Trials and Studies Coordinating Centre, Alpha House, University of Southampton Science Park, Southampton SO16 7NS, UK.

The 17 interventions comprised 1762 children. Control interventions included interventions delivered at home ($n = 4$) and in the clinic ($n = 3$). Control interventions varied in content, length and intensity and included a 10-week Developmental Education for Families programme;[194] psycho-educational home visits;[196] a single educational lecture;[274] a single home visit;[218] and care as usual that included a range of interventions (including some of those listed above).[129,190,197,200] The parental interventions for attachment included a wide range of therapies delivered at different times. This included interventions delivered with parents prenatally ($n = 8$), between 0 and 6 months of age ($n = 5$) and with parents of children older than 6 months ($n = 11$). Some of the studies were carried out in at-risk groups, including foster children ($n = 1$), children with a history of maltreatment and children of parents with mental health problems.

As a result of this diversity, a series of meta-analyses were carried out to explore factors that may have influenced study outcome. They include the following:

- duration of intervention (< 12 months/≥ 12 months)
- length of follow-up (< 12 months/≥ 12 months)
- number of sessions (≤ 5, 5–15, ≥ 16)
- age of child at start of the intervention (≤ 6 months/> 6 months)
- middle-class families
- intervention location (home, mixed, other)
- male caregiver included
- video feedback
- attempts to enhance maternal sensitivity
- primary focus to modify child attachment
- caregiver and child (separate, together, mixed).

Study or subgroup	Experimental Events	Experimental Total	Control Events	Control Total	Weight	OR M–H, random, 95% CI
**<12 months**						
Brisch 2003[202]	19	32	28	36	9.1%	0.42 (0.15 to 1.20)
Dozier 2009,[194] Bernard 2012[193]	31	60	20	60	11.3%	2.14 (1.02 to 4.47)
Klein-Velderman (+D) 2006[203]	16	26	15	27	8.9%	1.28 (0.43 to 3.83)
Klein-Velderman 2006[204]	16	26	15	27	8.9%	1.28 (0.43 to 3.83)
Moran 2005[218]	28	49	19	50	10.8%	2.18 (0.97 to 4.86)
Moss 2011[195]	23	35	9	32	9.2%	4.90 (1.73 to 13.85)
Murray,[208] Cooper (CBT) 2003[209]	19	41	27	47	10.6%	0.64 (0.28 to 1.49)
Murray,[208] Cooper (Cou) 2003[209]	25	39	27	47	10.4%	1.32 (0.55 to 3.17)
Murray,[208] Cooper (Psy) 2003[209]	18	40	27	47	10.5%	0.61 (0.26 to 1.42)
van den Boom 1994,[198] 1995[197]	31	50	11	50	10.3%	5.78 (2.40 to 13.94)
**Subtotal (95% CI)**		**398**		**423**	**100.0%**	**1.48 (0.87 to 2.50)**
Total events	226		198			
Heterogeneity: $\tau^2=0.50$; $\chi^2=29.89$, df$=9$ ($p=0.0005$); $I^2=70\%$						
Test for overall effect: $z=1.46$ ($p=0.15$)						
**≥12 months**						
Barnett (NonP) 1985,[133] 1987[129]	20	28	17	23	32.6%	0.88 (0.26 to 3.05)
Barnett (Prof) 1985,[133] 1987[129]	17	29	17	23	32.9%	0.50 (0.15 to 1.64)
Cicchetti 1999,[200] Toth 2006[199]	31	46	9	54	34.5%	10.33 (4.02 to 26.57)
**Subtotal (95% CI)**		**103**		**100**	**100.0%**	**1.71 (0.24 to 12.10)**
Total events	68		43			
Heterogeneity: $\tau^2=2.66$; $\chi^2=18.35$, df$=2$ ($p=0.0001$); $I^2=89\%$						
Test for overall effect: $z=0.54$ ($p=0.59$)						

Test for subgroup differences: $\chi^2=0.02$, df$=1$ ($p=0.89$); $I^2=0\%$

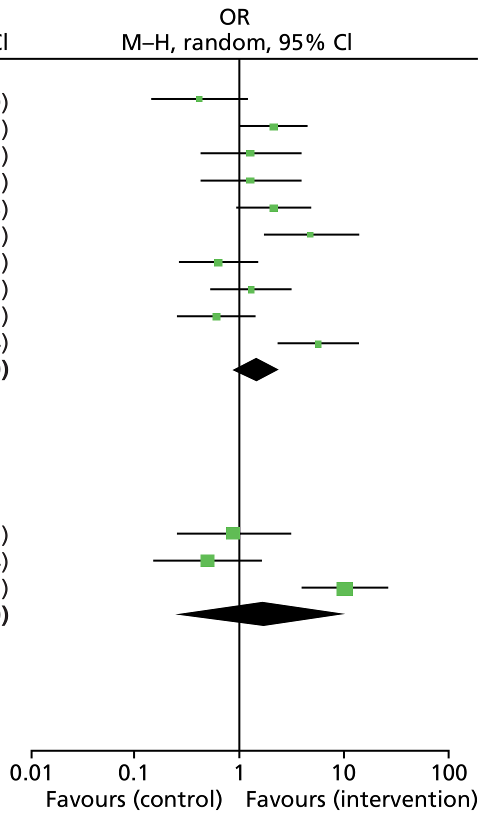

FIGURE 26 A meta-analysis of changes in secure outcomes comparing duration of intervention (< 12 months, ≥ 12 months).

© Queen's Printer and Controller of HMSO 2015. This work was produced by Wright *et al.* under the terms of a commissioning contract issued by the Secretary of State for Health. This issue may be freely reproduced for the purposes of private research and study and extracts (or indeed, the full report) may be included in professional journals provided that suitable acknowledgement is made and the reproduction is not associated with any form of advertising. Applications for commercial reproduction should be addressed to: NIHR Journals Library, National Institute for Health Research, Evaluation, Trials and Studies Coordinating Centre, Alpha House, University of Southampton Science Park, Southampton SO16 7NS, UK.

Only three studies are included in the meta-analysis for intervention that carries on for longer than 12 months. The 95% CI for the OR is large (0.24 to 12.0). The findings show that interventions promoting secure attachment can achieve significant outcomes using interventions of less than 12 months' duration.

Study or subgroup	Experimental Events	Experimental Total	Control Events	Control Total	Weight	OR M–H, random, 95% CI
**<12 months**						
Barnett (NonP) 1985,[133] 1987[129]	20	28	17	23	9.5%	0.88 (0.26 to 3.05)
Barnett (Prof) 1985,[133] 1987[129]	17	29	17	23	9.8%	0.50 (0.15 to 1.64)
Brisch 2003[202]	19	32	28	36	10.8%	0.42 (0.15 to 1.20)
Dozier 2009,[194] Bernard 2012[193]	31	60	20	60	13.2%	2.14 (1.02 to 4.47)
Klein-Velderman (+D) 2006[203]	16	26	15	27	10.5%	1.28 (0.43 to 3.83)
Klein-Velderman 2006[204]	16	26	15	27	10.5%	1.28 (0.43 to 3.83)
Moran 2005[218]	28	49	19	50	12.7%	2.18 (0.97 to 4.86)
Moss 2011[195]	23	35	9	32	10.9%	4.90 (1.73 to 13.85)
van den Boom 1994,[198] 1995[197]	31	50	11	50	12.1%	5.78 (2.40 to 13.94)
**Subtotal (95% CI)**		**335**		**328**	**100.0%**	**1.59 (0.89 to 2.83)**
Total events	201		151			
Heterogeneity: $\tau^2=0.52$; $\chi^2=24.56$, df = 8 ($p=0.002$); $I^2=67\%$						
Test for overall effect: $z=1.57$ ($p=0.12$)						
**≥12 months**						
Cooper 2009[189]	116	156	102	162	34.2%	1.71 (1.06 to 2.76)
Murray,[208] Cooper (CBT) 2003[209]	19	41	27	47	22.3%	0.64 (0.28 to 1.49)
Murray,[208] Cooper (Cou) 2003[209]	25	39	27	47	21.5%	1.32 (0.55 to 3.17)
Murray,[208] Cooper (Psy) 2003[209]	18	40	27	47	22.1%	0.61 (0.26 to 1.42)
**Subtotal (95% CI)**		**276**		**303**	**100.0%**	**1.03 (0.59 to 1.80)**
Total events	178		183			
Heterogeneity: $\tau^2=0.17$; $\chi^2=6.71$, df = 3 ($p=0.08$); $I^2=55\%$						
Test for overall effect: $z=0.11$ ($p=0.91$)						

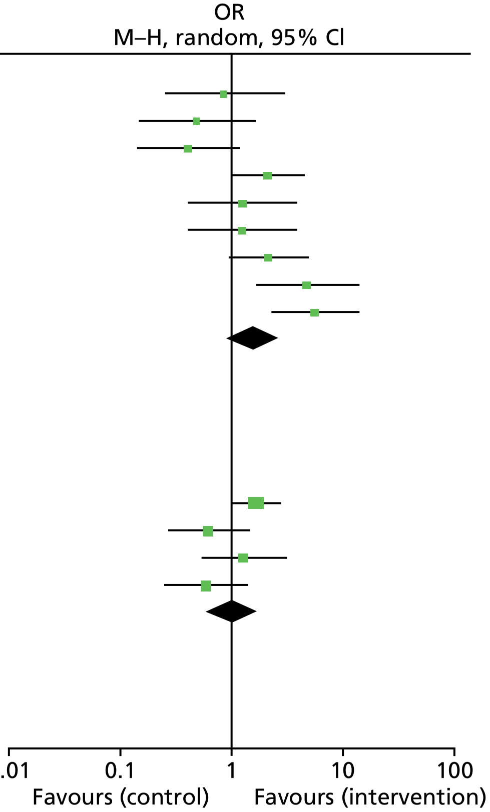

Test for subgroup differences: $\chi^2=1.11$, df = 1 ($p=0.29$); $I^2=9.6\%$

FIGURE 27 A meta-analysis of changes in secure outcomes comparing different lengths of follow-up (< 12 months, ≥ 12 months).

© Queen's Printer and Controller of HMSO 2015. This work was produced by Wright *et al.* under the terms of a commissioning contract issued by the Secretary of State for Health. This issue may be freely reproduced for the purposes of private research and study and extracts (or indeed, the full report) may be included in professional journals provided that suitable acknowledgement is made and the reproduction is not associated with any form of advertising. Applications for commercial reproduction should be addressed to: NIHR Journals Library, National Institute for Health Research, Evaluation, Trials and Studies Coordinating Centre, Alpha House, University of Southampton Science Park, Southampton SO16 7NS, UK.

We carried out an analysis that explored studies where the length of follow-up reported in the paper was less than 12 months and greater than 12 months. This follow-up time is calculated from the end of the intervention to when the first attachment measure was conducted. There is no overall effect in the group being followed up over 12 months. These studies were all by one research team.

Study or subgroup	Experimental Events	Experimental Total	Control Events	Control Total	Weight	OR M–H, random, 95% CI
**< 5 sessions**						
Cassidy 2011[196]	51	85	42	84	32.5%	1.50 (0.82 to 2.76)
Klein-Velderman (+D) 2006[203]	16	26	15	27	21.1%	1.28 (0.43 to 3.83)
Klein-Velderman 2006[204]	16	26	15	27	21.1%	1.28 (0.43 to 3.83)
van den Boom 1994,[198] 1995[197]	31	50	11	50	25.6%	5.78 (2.40 to 13.94)
**Subtotal (95% CI)**		**187**		**188**	**100.0%**	**1.98 (0.97 to 4.06)**
Total events	114		83			
Heterogeneity: $\tau^2=0.32$; $\chi^2=7.72$, df = 3 ($p=0.005$); $I^2=61\%$						
Test for overall effect: $z=1.87$ ($p=0.06$)						
**5–16 sessions**						
Brisch 2003[202]	19	32	28	36	10.2%	0.42 (0.15 to 1.20)
Cooper 2009[189]	116	156	102	162	16.5%	1.71 (1.06 to 2.76)
Dozier 2009,[194] Bernard 2012[193]	31	60	20	60	13.5%	2.14 (1.02 to 4.47)
Moran 2005[218]	28	49	19	50	12.8%	2.18 (0.97 to 4.86)
Moss 2011[195]	23	35	9	32	10.4%	4.90 (1.73 to 13.85)
Murray,[208] Cooper (CBT) 2003[209]	19	41	27	47	12.4%	0.64 (0.28 to 1.49)
Murray,[208] Cooper (Cou) 2003[209]	25	39	27	47	12.0%	1.32 (0.55 to 3.17)
Murray,[208] Cooper (Psy) 2003[209]	18	40	27	47	12.3%	0.61 (0.26 to 1.42)
**Subtotal (95% CI)**		**452**		**481**	**100.0%**	**1.33 (0.81 to 2.16)**
Total events	279		259			
Heterogeneity: $\tau^2=0.32$; $\chi^2=20.74$, df = 7 ($p=0.004$); $I^2=66\%$						
Test for overall effect: $z=1.13$ ($p=0.26$)						
**> 16 sessions**						
Cicchetti 1999,[200] Toth 2006[199]	31	46	9	54	52.7%	10.33 (4.02 to 26.57)
Heinicke 1999,[191] 2001[190]	24	31	17	33	47.3%	3.23 (1.09 to 9.54)
**Subtotal (95% CI)**		**77**		**87**	**100.0%**	**5.96 (1.91 to 18.62)**
Total events	55		26			
Heterogeneity: $\tau^2=0.41$; $\chi^2=2.52$, df = 1 ($p=0.11$); $I^2=60\%$						
Test for overall effect: $z=3.07$ ($p=0.002$)						

Test for subgroup differences: $\chi^2=5.80$, df = 2 ($p=0.05$); $I^2=65.5\%$

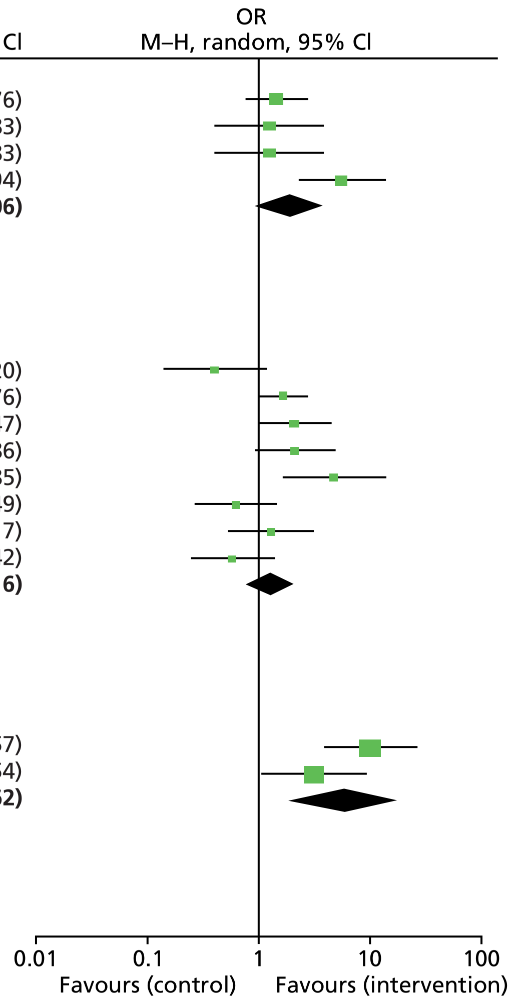

FIGURE 28 A meta-analysis of changes in secure outcomes comparing the number of sessions (< 5, 5–16, > 16).

© Queen's Printer and Controller of HMSO 2015. This work was produced by Wright *et al.* under the terms of a commissioning contract issued by the Secretary of State for Health. This issue may be freely reproduced for the purposes of private research and study and extracts (or indeed, the full report) may be included in professional journals provided that suitable acknowledgement is made and the reproduction is not associated with any form of advertising. Applications for commercial reproduction should be addressed to: NIHR Journals Library, National Institute for Health Research, Evaluation, Trials and Studies Coordinating Centre, Alpha House, University of Southampton Science Park, Southampton SO16 7NS, UK.

Meta-analysis of the number of sessions shows that positive outcomes can be achieved when different researchers have used different numbers of sessions. While interventions with greater than 16 sessions have higher significance and effect sizes, this cannot be taken to assume greater effectiveness as no direct comparison has been made. None of the studies directly compare a small number of sessions with a large number of sessions. The finding that a small number of sessions may be effective suggests that just such a study may be worthwhile as a mechanism to explore cost-effectiveness in the short and longer terms.

Study or subgroup	Experimental Events	Experimental Total	Control Events	Control Total	Weight	OR M–H, random, 95% CI
**Prenatal**						
Brisch 2003[202]	19	32	28	36	29.8%	0.42 (0.15 to 1.20)
Cooper 2009[189]	116	156	102	162	41.0%	1.71 (1.06 to 2.76)
Heinicke 1999,[191] 2001[190]	24	31	17	33	29.2%	3.23 (1.09 to 9.54)
**Subtotal (95% CI)**		**219**		**231**	**100.0%**	**1.35 (0.51 to 3.61)**
Total events	159		147			
Heterogeneity: $\tau^2=0.55$; $\chi^2=7.83$, df = 2 ($p=0.02$); $I^2=74\%$						
Test for overall effect: $z=0.60$ ($p=0.55$)						
**≤6 months**						
Anisfeld 1990[180]	19	23	10	26	22.7%	7.60 (2.00 to 28.93)
Moran 2005[218]	28	49	19	50	40.2%	2.18 (0.97 to 4.86)
van den Boom 1994,[198] 1995[197]	31	50	11	50	37.0%	5.78 (2.40 to 13.94)
**Subtotal (95% CI)**		**122**		**126**	**100.0%**	**4.15 (1.92 to 8.98)**
Total events	78		40			
Heterogeneity: $\tau^2=0.22$; $\chi^2=3.75$, df = 2 ($p=0.15$); $I^2=47\%$						
Test for overall effect: $z=3.62$ ($p=0.0003$)						
**>6 months**						
Cassidy 2011[196]	51	85	42	84	14.6%	1.50 (0.82 to 2.76)
Cicchetti 1999,[200] Toth 2006[199]	31	46	9	54	12.3%	10.33 (4.02 to 26.57)
Klein-Velderman (+D) 2006[203]	16	26	15	27	11.3%	1.28 (0.43 to 3.83)
Klein-Velderman 2006[204]	16	26	15	27	11.3%	1.28 (0.43 to 3.83)
Moss 2011[195]	23	35	9	32	11.7%	4.90 (1.73 to 13.85)
Murray,[208] Cooper (CBT) 2003[209]	19	41	27	47	13.0%	0.64 (0.28 to 1.49)
Murray,[208] Cooper (Cou) 2003[209]	25	39	27	47	12.8%	1.32 (0.55 to 3.17)
Murray,[208] Cooper (Psy) 2003[209]	18	40	27	47	13.0%	0.61 (0.26 to 1.42)
**Subtotal (95% CI)**		**338**		**365**	**100.0%**	**1.65 (0.86 to 3.16)**
Total events	199		171			
Heterogeneity: $\tau^2=0.66$; $\chi^2=29.52$, df = 7 ($p=0.0001$); $I^2=76\%$						
Test for overall effect: $z=1.51$ ($p=0.13$)						

Test for subgroup differences: $\chi^2=4.30$, df = 2 ($p=0.12$); $I^2=53.4\%$

OR M–H, random, 95% CI

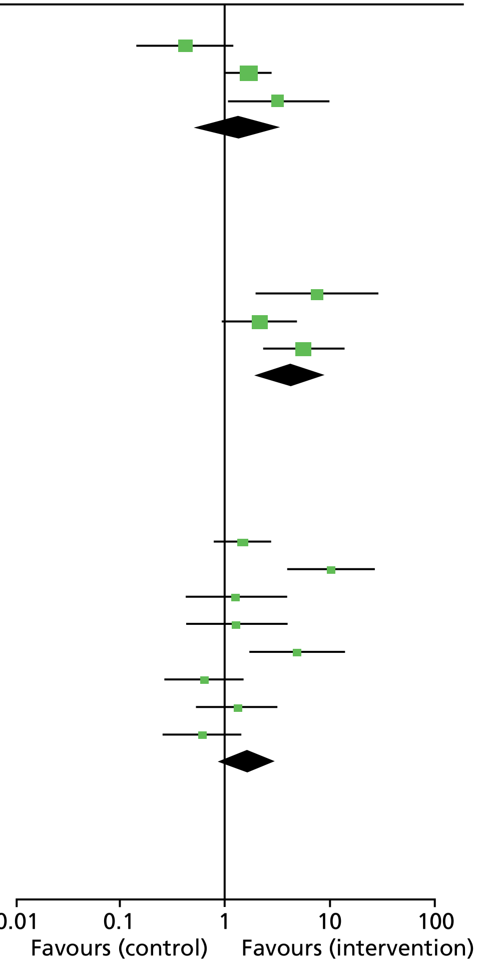

FIGURE 29 A meta-analysis of changes in secure outcomes comparing age of child at start (≤ 6 months, > 6 months).

© Queen's Printer and Controller of HMSO 2015. This work was produced by Wright *et al.* under the terms of a commissioning contract issued by the Secretary of State for Health. This issue may be freely reproduced for the purposes of private research and study and extracts (or indeed, the full report) may be included in professional journals provided that suitable acknowledgement is made and the reproduction is not associated with any form of advertising. Applications for commercial reproduction should be addressed to: NIHR Journals Library, National Institute for Health Research, Evaluation, Trials and Studies Coordinating Centre, Alpha House, University of Southampton Science Park, Southampton SO16 7NS, UK.

When exploring the effect of interventions delivered at different ages there is no significance achieved for interventions delivered prenatally or for those starting older than 6 months of age. Despite relatively large ORs for some interventions after 6 months of age, others showed limited apparent benefit. Interventions that began at the age of between 0 and 6 months show the greatest effect and significance, but were limited to three studies. None of the studies directly compared the timing of the intervention and so it is not possible to make direct comparisons.

Study or subgroup	Control Events	Control Total	Parenting Events	Parenting Total	Weight	OR M–H, random, 95% CI
**Middle class**						
Brisch 2003[202]	19	32	28	36	100.0%	0.42 (0.15 to 1.20)
**Subtotal (95% CI)**		**32**		**36**	**100.0%**	**0.42 (0.15 to 1.20)**
Total events	19		28			
Heterogeneity: not applicable						
Test for overall effect: $z = 1.62$ ($p = 0.11$)						
**Other**						
Anisfeld 1990[180]	19	23	10	26	4.6%	7.60 (2.00 to 28.93)
Barnett (NonP) 1985,[133] 1987[129]	20	28	17	23	5.0%	0.88 (0.26 to 3.05)
Barnett (Prof) 1985,[133] 1987[129]	17	29	17	23	5.2%	0.50 (0.15 to 1.64)
Cassidy 2011[196]	51	85	42	84	7.8%	1.50 (0.82 to 2.76)
Cicchetti 1999,[200] Toth 2006[199]	31	46	9	54	6.2%	10.33 (4.02 to 26.57)
Cooper 2009[189]	116	156	102	162	8.4%	1.71 (1.06 to 2.76)
Dozier 2009,[194] Bernard 2012[193]	31	60	20	60	7.2%	2.14 (1.02 to 4.47)
Heinicke 1999,[191] 2001[190]	24	31	17	33	5.6%	3.23 (1.09 to 9.54)
Klein-Velderman (+D) 2006[203]	16	26	15	27	5.5%	1.28 (0.43 to 3.83)
Klein-Velderman 2006[204]	16	26	15	27	5.5%	1.28 (0.43 to 3.83)
Moran 2005[218]	28	49	19	50	6.9%	2.18 (0.97 to 4.86)
Moss 2011[195]	23	35	9	32	5.8%	4.90 (1.73 to 13.85)
Murray,[208] Cooper (CBT) 2003[209]	19	41	27	47	6.7%	0.64 (0.28 to 1.49)
Murray,[208] Cooper (Cou) 2003[209]	25	39	27	47	6.5%	1.32 (0.55 to 3.17)
Murray,[208] Cooper (Psy) 2003[209]	18	40	27	47	6.7%	0.61 (0.26 to 1.42)
van den Boom 1994,[198] 1995[197]	31	50	11	50	6.5%	5.78 (2.40 to 13.94)
**Subtotal (95% CI)**		**764**		**792**	**100.0%**	**1.89 (1.27 to 2.81)**
Total events	485		384			
Heterogeneity: $\tau^2 = 0.44$; $\chi^2 = 48.90$, df = 15 ($p < 0.0001$); $I^2 = 69\%$						
Test for overall effect: $z = 3.12$ ($p = 0.002$)						

OR M–H, random, 95% CI

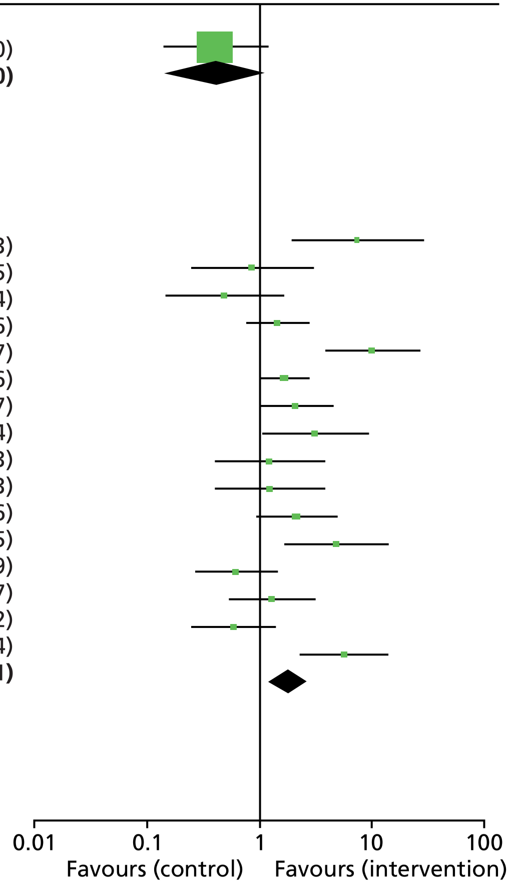

Test for subgroup differences: $\chi^2 = 6.86$, df = 1 ($p = 0.009$); $I^2 = 85.4\%$

FIGURE 30 A meta-analysis of changes in secure outcomes comparing middle-class families and other family types.

© Queen's Printer and Controller of HMSO 2015. This work was produced by Wright *et al.* under the terms of a commissioning contract issued by the Secretary of State for Health. This issue may be freely reproduced for the purposes of private research and study and extracts (or indeed, the full report) may be included in professional journals provided that suitable acknowledgement is made and the reproduction is not associated with any form of advertising. Applications for commercial reproduction should be addressed to: NIHR Journals Library, National Institute for Health Research, Evaluation, Trials and Studies Coordinating Centre, Alpha House, University of Southampton Science Park, Southampton SO16 7NS, UK.

We examined the SES of the intervention populations and found that some studies described their population sample as 'middle class', while the majority targeted interventions at low socioeconomic groups. A much larger effect size was found in studies targeted at lower socioeconomic groups, although only two studies with 'middle-class' populations were available for comparison. There were no studies comparing the same intervention targeted at different socioeconomic groups and so no direct comparisons can be made.

We examined studies that were conducted at home, in mixed locations (i.e home and another setting) and interventions carried out at other locations. Brisch *et al.*[201] conducted their intervention in hospital. Meta-analysis of interventions carried out in the home, or home and elsewhere (mixed) both had significant overall effect sizes.

Anisfeld *et al.*[180] provided the parents with baby carriers that could be used in a variety of locations. Heinicke *et al.*[190,191] carried out their intervention in the home but additionally held parent groups in different locations.

Study or subgroup	Experimental Events	Experimental Total	Control Events	Control Total	Weight	OR M–H, random, 95% CI
**Male carer included**						
Barnett (NonP) 1985,[133] 1987[129]	20	28	17	23	17.6%	0.88 (0.26 to 3.05)
Barnett (Prof) 1985,[133] 1987[129]	17	29	17	23	18.3%	0.50 (0.15 to 1.64)
Brisch 2003[202]	19	32	28	36	20.0%	0.42 (0.15 to 1.20)
Dozier 2009,[194] Bernard 2012[193]	31	60	20	60	24.4%	2.14 (1.02 to 4.47)
Heinicke 1999,[191] 2001[190]	24	31	17	33	19.6%	3.23 (1.09 to 9.54)
**Subtotal (95% CI)**		**180**		**175**	**100.0%**	**1.10 (0.49 to 2.43)**
Total events	111		99			
Heterogeneity: $\tau^2=0.53$; $\chi^2=11.70$, df=4 ($p=0.02$); $I^2=66\%$						
Test for overall effect: $z=0.23$ ($p=0.82$)						
**Male carer not included**						
Anisfeld 1990[180]	19	23	10	26	6.5%	7.60 (2.00 to 28.93)
Cassidy 2011[196]	51	85	42	84	11.0%	1.50 (0.82 to 2.76)
Cicchetti 1999,[200] Toth 2006[199]	31	46	9	54	8.8%	10.33 (4.02 to 26.57)
Cooper 2009[189]	116	156	102	162	11.8%	1.71 (1.06 to 2.76)
Klein-Velderman (+D) 2006[203]	16	26	15	27	7.9%	1.28 (0.43 to 3.83)
Klein-Velderman 2006[204]	16	26	15	27	7.9%	1.28 (0.43 to 3.83)
Moran 2005[218]	28	49	19	50	9.7%	2.18 (0.97 to 4.86)
Moss 2011[195]	23	35	9	32	8.2%	4.90 (1.73 to 13.85)
Murray,[208] Cooper (CBT) 2003[209]	19	41	27	47	9.5%	0.64 (0.28 to 1.49)
Murray,[208] Cooper (Cou) 2003[209]	25	39	27	47	9.3%	1.32 (0.55 to 3.17)
Murray,[208] Cooper (Psy) 2003[209]	18	40	27	47	9.4%	0.61 (0.26 to 1.42)
**Subtotal (95% CI)**		**566**		**603**	**100.0%**	**1.87 (1.16 to 3.02)**
Total events	362		302			
Heterogeneity: $\tau^2=0.44$; $\chi^2=35.00$, df=10 ($p=0.0001$); $I^2=71\%$						
Test for overall effect: $z=0.11$ ($p=0.91$)						

Test for subgroup differences: $\chi^2=1.28$, df=1 ($p=0.26$); $I^2=21.8\%$

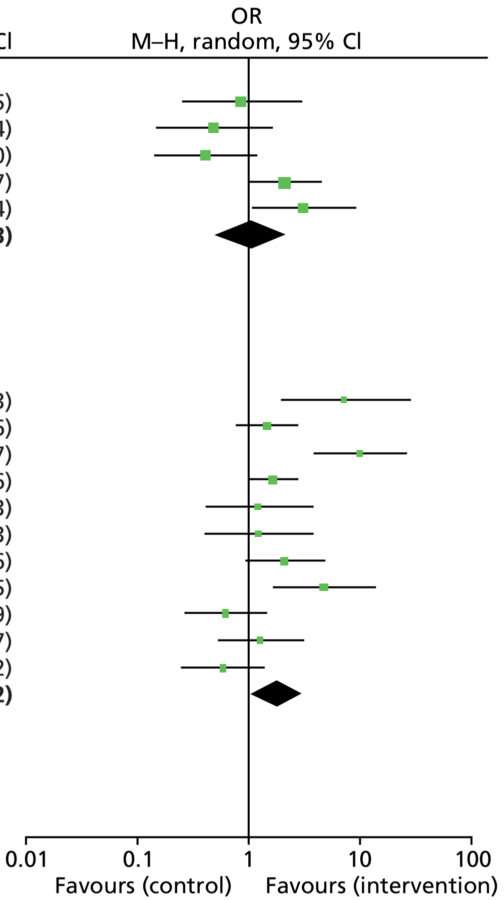

FIGURE 31 A meta-analysis of changes in secure outcomes comparing whether or not the male caregiver was included alongside the female caregiver in the intervention.

© Queen's Printer and Controller of HMSO 2015. This work was produced by Wright *et al.* under the terms of a commissioning contract issued by the Secretary of State for Health. This issue may be freely reproduced for the purposes of private research and study and extracts (or indeed, the full report) may be included in professional journals provided that suitable acknowledgement is made and the reproduction is not associated with any form of advertising. Applications for commercial reproduction should be addressed to: NIHR Journals Library, National Institute for Health Research, Evaluation, Trials and Studies Coordinating Centre, Alpha House, University of Southampton Science Park, Southampton SO16 7NS, UK.

The majority of interventions were targeted at the dyad between the infant and mother or female caregiver–child dyad. However, the interventions where the male caregiver also took part in the intervention alongside, or instead of, the female caregiver were meta-analysed. The effect size when the male caregiver was included was not significant.

Dozier and colleagues[194] and Bernard and colleagues[193] offered the intervention to both mothers and fathers. Four of the participating primary caregivers were male. Brisch and colleagues[202] focused on providing both mothers and fathers with individual and joint psychotherapy as well as allowing for male caregivers to be present in the other aspects of the intervention. Barnett and colleagues,[129] and Barnett and Parker[133] encouraged the male partner to be involved in the intervention and to support the mother. Heinicke and colleagues[190,191] encouraged the male caregiver to be involved in the intervention, and 42% of fathers chose to take part.

Study or subgroup	Experimental Events	Experimental Total	Control Events	Control Total	Weight	OR M–H, random, 95% CI
**No video feedback**						
Anisfeld 1990[180]	19	23	10	26	8.3%	7.60 (2.00 to 28.93)
Barnett (NonP) 1985,[133] 1987[129]	20	28	17	23	8.7%	0.88 (0.26 to 3.05)
Barnett (Prof) 1985,[133] 1987[129]	17	29	17	23	9.0%	0.50 (0.15 to 1.64)
Cicchetti 1999,[200] Toth 2006[199]	31	46	9	54	10.2%	10.33 (4.02 to 26.57)
Cooper 2009[189]	116	156	102	162	12.2%	1.71 (1.06 to 2.76)
Heinicke 1999,[191] 2001[190]	24	31	17	33	9.5%	3.23 (1.09 to 9.54)
Murray,[208] Cooper (CBT) 2003[209]	19	41	27	47	10.6%	0.64 (0.28 to 1.49)
Murray,[208] Cooper (Cou) 2003[209]	25	39	27	47	10.5%	1.32 (0.55 to 3.17)
Murray,[208] Cooper (Psy) 2003[209]	18	40	27	47	10.6%	0.61 (0.26 to 1.42)
van den Boom 1994,[198] 1995[197]	31	50	11	50	10.5%	5.78 (2.40 to 13.94)
**Subtotal (95% CI)**		**483**		**512**	**100.0%**	**1.86 (0.99 to 3.50)**
Total events	320		264			
Heterogeneity: $\tau^2 = 0.79$; $\chi^2 = 43.84$, df = 9 ($p < 0.00001$); $I^2 = 79\%$						
Test for overall effect: $z = 1.92$ ($p = 0.05$)						
**Video feedback**						
Brisch 2003[202]	19	32	28	36	12.0%	0.42 (0.15 to 1.20)
Cassidy 2011[196]	51	85	42	84	19.7%	1.50 (0.82 to 2.76)
Dozier 2009,[194] Bernard 2012[193]	31	60	20	60	17.2%	2.14 (1.02 to 4.47)
Klein-Velderman (+D) 2006[203]	16	26	15	27	11.5%	1.28 (0.43 to 3.83)
Klein-Velderman 2006[204]	16	26	15	27	11.5%	1.28 (0.43 to 3.83)
Moran 2005[218]	28	49	19	50	15.9%	2.18 (0.97 to 4.86)
Moss 2011[195]	23	35	9	32	12.2%	4.90 (1.73 to 13.85)
**Subtotal (95% CI)**		**313**		**316**	**100.0%**	**1.62 (1.00 to 2.60)**
Total events	184		148			
Heterogeneity: $\tau^2 = 0.20$; $\chi^2 = 12.15$, df = 6 ($p = 0.06$); $I^2 = 51\%$						
Test for overall effect: $z = 1.97$ ($p = 0.05$)						

Test for subgroup differences: $\chi^2 = 0.12$, df = 1 ($p = 0.73$); $I^2 = 0\%$

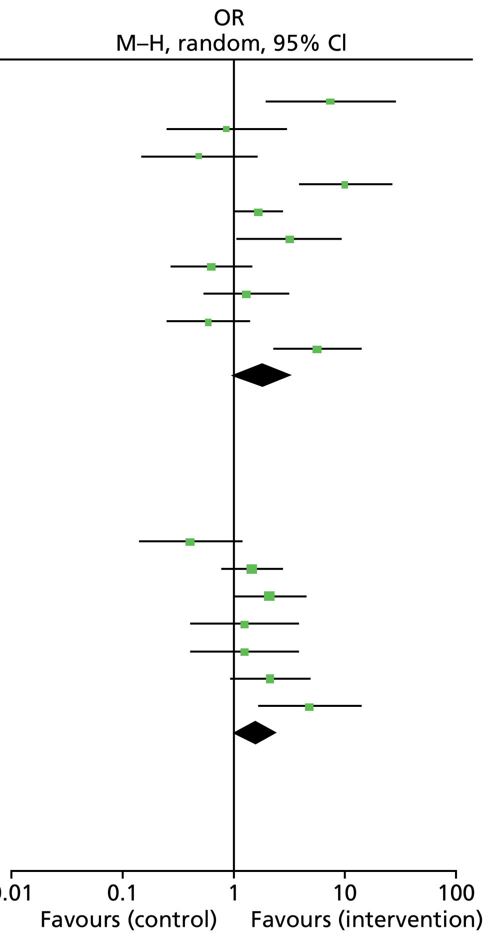

FIGURE 32 A meta-analysis of changes in secure outcomes comparing whether or not video feedback was provided.

© Queen's Printer and Controller of HMSO 2015. This work was produced by Wright *et al.* under the terms of a commissioning contract issued by the Secretary of State for Health. This issue may be freely reproduced for the purposes of private research and study and extracts (or indeed, the full report) may be included in professional journals provided that suitable acknowledgement is made and the reproduction is not associated with any form of advertising. Applications for commercial reproduction should be addressed to: NIHR Journals Library, National Institute for Health Research, Evaluation, Trials and Studies Coordinating Centre, Alpha House, University of Southampton Science Park, Southampton SO16 7NS, UK.

There were seven studies where interventions used video feedback. The OR was 1.62 (95% CI 1.00 to 2.60), which was statistically significant ($p = 0.05$). Meta-analysis found that interventions without video feedback were also effective, which was statistically significant ($p = 0.05$). There were individual interventions in both groups that showed limited effect.

Study or subgroup	Experimental Events	Experimental Total	Control Events	Control Total	Weight	OR M–H, random, 95% CI
**Yes**						
Anisfeld 1990[180]	19	23	10	26	5.8%	7.60 (2.00 to 28.93)
Barnett (NonP) 1985,[133] 1987[129]	20	28	17	23	6.3%	0.88 (0.26 to 3.05)
Barnett (Prof) 1985,[133] 1987[129]	17	29	17	23	6.5%	0.50 (0.15 to 1.64)
Brisch 2003[202]	19	32	28	36	7.2%	0.42 (0.15 to 1.20)
Cassidy 2011[196]	51	85	42	84	9.6%	1.50 (0.82 to 2.76)
Cicchetti 1999,[200] Toth 2006[199]	31	46	9	54	7.8%	10.33 (4.02 to 26.57)
Cooper 2009[189]	116	156	102	162	10.3%	1.71 (1.06 to 2.76)
Dozier 2009,[194] Bernard 2012[193]	31	60	20	60	8.9%	2.14 (1.02 to 4.47)
Klein-Velderman (+D) 2006[203]	16	26	15	27	7.0%	1.28 (0.43 to 3.83)
Klein-Velderman 2006[204]	20	28	15	27	6.9%	2.00 (0.65 to 6.11)
Moran 2005[218]	28	49	19	50	8.5%	2.18 (0.97 to 4.86)
Moss 2011[195]	23	35	9	32	7.3%	4.90 (1.73 to 13.85)
van den Boom 1994,[198] 1995[197]	31	50	11	50	8.1%	5.78 (2.40 to 13.94)
**Subtotal (95% CI)**		**647**		**654**	**100.0%**	**2.08 (1.32 to 3.30)**
Total events	422		314			
Heterogeneity: $\tau^2=0.47$; $\chi^2=41.22$, df=12 ($p<0.0001$); $I^2=71\%$						
Test for overall effect: $z=3.14$ ($p=0.002$)						
**No**						
Heinicke 1999,[191] 2001[190]	24	31	17	33	21.3%	3.23 (1.09 to 9.54)
Murray,[208] Cooper (CBT) 2003[209]	19	41	27	47	26.5%	0.64 (0.28 to 1.49)
Murray,[208] Cooper (Cou) 2003[209]	25	39	27	47	25.8%	1.32 (0.55 to 3.17)
Murray,[208] Cooper (Psy) 2003[209]	18	40	27	47	26.4%	0.61 (0.26 to 1.42)
**Subtotal (95% CI)**		**151**		**174**	**100.0%**	**1.07 (0.53 to 2.18)**
Total events	86		98			
Heterogeneity: $\tau^2=0.30$; $\chi^2=7.30$, df=3 ($p=0.06$); $I^2=59\%$						
Test for overall effect: $z=0.20$ ($p=0.84$)						

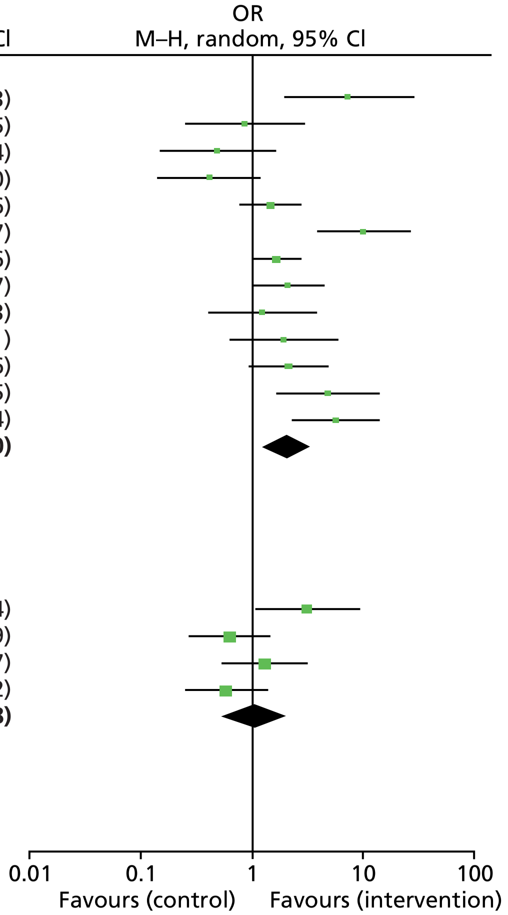

Test for subgroup differences: $\chi^2=2.38$, df=1 ($p=0.12$); $I^2=58.0\%$

FIGURE 33 A meta-analysis of changes in secure outcomes comparing whether or not the intervention attempts to enhance maternal sensitivity.

© Queen's Printer and Controller of HMSO 2015. This work was produced by Wright *et al.* under the terms of a commissioning contract issued by the Secretary of State for Health. This issue may be freely reproduced for the purposes of private research and study and extracts (or indeed, the full report) may be included in professional journals provided that suitable acknowledgement is made and the reproduction is not associated with any form of advertising. Applications for commercial reproduction should be addressed to: NIHR Journals Library, National Institute for Health Research, Evaluation, Trials and Studies Coordinating Centre, Alpha House, University of Southampton Science Park, Southampton SO16 7NS, UK.

Many researchers and clinicians target interventions at maternal sensitivity to the infant when working to improve attachment and relationships. This meta-analysis suggests that this approach improves secure attachment. The studies not using this approach when meta-analysed do not reach significance. The interventions that have not focused on improving maternal sensitivity have focused on improving parents' mental health[208,209] and promoting the caregivers' sense of self efficacy.[190,191] No studies directly compare maternal sensitivity interventions with those that do not, and so it is difficult to make any direct comparisons or definitive statements about this.

Study or subgroup	Experimental Events	Experimental Total	Control Events	Control Total	Weight	OR M–H, random, 95% CI
**Yes**						
Anisfeld 1990[180]	19	23	10	26	6.8%	7.60 (2.00 to 28.93)
Brisch 2003[202]	19	32	28	36	8.3%	0.42 (0.15 to 1.20)
Cassidy 2011[196]	51	85	42	84	11.2%	1.50 (0.82 to 2.76)
Cicchetti 1999,[200] Toth 2006[199]	31	46	9	54	9.0%	10.33 (4.02 to 26.57)
Cooper 2009[189]	116	156	102	162	12.0%	1.71 (1.06 to 2.76)
Dozier 2009,[194] Bernard 2012[193]	31	60	20	60	10.4%	2.14 (1.02 to 4.47)
Heinicke 1999,[191] 2001[190]	24	31	17	33	8.2%	3.23 (1.09 to 9.54)
Klein-Velderman (+D) 2006[203]	16	26	15	27	8.1%	1.28 (0.43 to 3.83)
Klein-Velderman 2006[204]	16	26	15	27	8.1%	1.28 (0.43 to 3.83)
Moss 2011[195]	23	35	9	35	8.4%	4.90 (1.73 to 13.85)
van den Boom 1994,[198] 1995[197]	31	50	11	50	9.5%	5.78 (2.40 to 13.94)
**Subtotal (95% CI)**		**570**		**591**	**100.0%**	**2.46 (1.50 to 4.02)**
Total events	377		278			
Heterogeneity: $\tau^2=0.47$; $\chi^2=34.99$, df = 10 ($p=0.0001$); $I^2=71\%$						
Test for overall effect: $z=3.58$ ($p=0.0003$)						
**No**						
Barnett (NonP) 1985,[133] 1987[129]	20	28	17	23	11.3%	0.88 (0.26 to 3.05)
Barnett (Prof) 1985,[133] 1987[129]	17	29	17	23	12.0%	0.50 (0.15 to 1.64)
Moran 2005[218]	28	49	19	50	20.3%	2.18 (0.97 to 4.86)
Murray,[208] Cooper (CBT) 2003[209]	19	41	27	47	19.1%	0.64 (0.28 to 1.49)
Murray,[208] Cooper (Cou) 2003[209]	25	39	27	47	18.4%	1.32 (0.55 to 3.17)
Murray,[208] Cooper (Psy) 2003[209]	18	40	27	47	19.0%	0.61 (0.26 to 1.42)
**Subtotal (95% CI)**		**226**		**237**	**100.0%**	**0.93 (0.58 to 1.50)**
Total events	127		134			
Heterogeneity: $\tau^2=0.12$; $\chi^2=7.69$, df = 5 ($p=0.17$); $I^2=35\%$						
Test for overall effect: $z=0.28$ ($p=0.78$)						

Test for subgroup differences: $\chi^2=7.69$, df = 1 ($p=0.006$); $I^2=87.0\%$

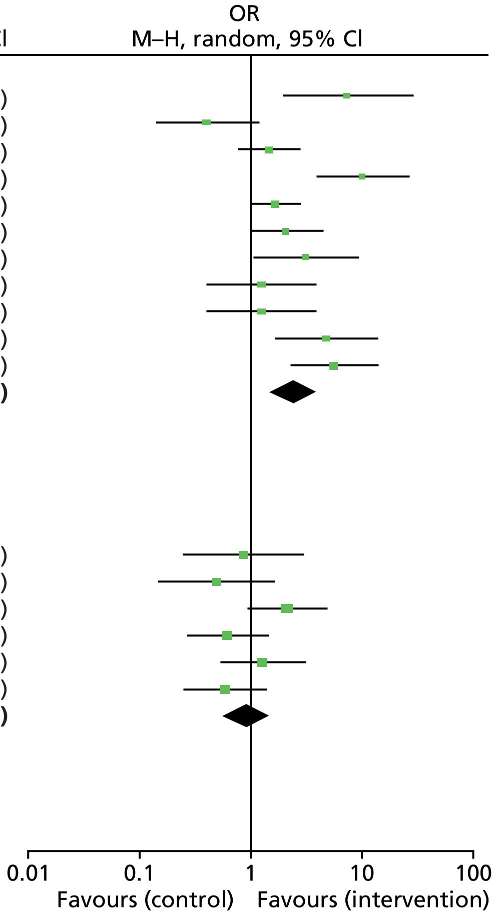

FIGURE 34 A meta-analysis of changes to secure outcomes comparing whether or not the primary focus of the intervention was to modify child attachment.

© Queen's Printer and Controller of HMSO 2015. This work was produced by Wright *et al.* under the terms of a commissioning contract issued by the Secretary of State for Health. This issue may be freely reproduced for the purposes of private research and study and extracts (or indeed, the full report) may be included in professional journals provided that suitable acknowledgement is made and the reproduction is not associated with any form of advertising. Applications for commercial reproduction should be addressed to: NIHR Journals Library, National Institute for Health Research, Evaluation, Trials and Studies Coordinating Centre, Alpha House, University of Southampton Science Park, Southampton SO16 7NS, UK.

Studies that set out explicitly to promote attachment security perform well when meta-analysed. Where the goal was not to modify the child's attachment security, there is no significant effect on meta-analysis. For example, Barnett and colleagues[129] and Barnett and Parker[133] focused on general support and specific antianxiety measures for the caregiver. Murray and colleagues[208] and Cooper and colleagues[209] focused their intervention on addressing the psychological needs of the caregiver, providing the caregiver with counselling, psychotherapy or cognitive–behavioural therapy. Moran and colleagues[218] focused the intervention on supporting the mother's sensitivity towards her infant. No studies directly compared attachment-focused interventions with those focused in other areas.

The meta-analysis compares whether the intervention was conducted within the dyad of mother and child, with the mother separately or with a combination of both dyadic work and individual intervention for the caregiver. Meta-analysis appears to confirm an earlier suggestion that interventions that involve the child and caregiver do well. Interventions that do not involve the primary caregiver or mother do not do well, but there are relatively few of them. No studies directly compared interventions with and without the child.

Interventions promoting secure attachment

Interventions under 12 months in duration demonstrate statistically significant improvements in secure attachment. However, the paucity of interventions that last longer than 12 months means that little can be gleaned from this. Health economics work could usefully explore how many sessions are required for meaningful long-term change and improved outcomes.

Only a few interventions in two papers are included where more than 12 months' follow-up takes place, and this is not a large enough group to make any firm conclusions. The meta-analysis shows that studies with less than 12 months' follow-up can be effective and this may be an important finding from a resource utilisation point of view, given the large pressures on resources in the NHS and allied professional groups interested in attachment (e.g. local authority education departments). Whether or not treatment effects diminish over time has not been explicitly examined in research. Most research only includes follow-up for less than 12 months, which may not be long enough to examine effects of interest from a developmental or psychopathological point of view.

The greater number of sessions (> 16) appears to deliver better effect sizes but there are no studies directly comparing number of sessions in a RCT. Indeed, the effect size for 0–4 sessions (1.87) is very similar to that for 5–16 sessions (1.19), with neither reaching significance in meta-analysis.

Theorists who suggest that attachment interventions need to be delivered early in the child's life will be interested in the finding that interventions delivered between birth and 6 months showed the best effect size and significance in meta-analysis. These interventions involved an intervention where a baby carrier was used, a video feedback home-visit intervention and an intervention involving home visits plus a video-taped session. In contrast, the other two groups where intervention was started prenatally or after 6 months of age showed no statistically significant overall effect size when meta-analysed. This finding could be because of some other factors related to a bias of the included studies and should be treated with caution given that no direct comparison was made and the studies took place at different times in different places. Nevertheless, it is self-evident that attachment work is difficult to do with an infant in utero, and that 6 months after delivery may be leaving potential at-risk dyads without intervention for 6 months of the child's life.

The finding that the effect size is high in low socioeconomic groups is perhaps to be expected in that some researchers consider that many of the at-risk groups may be subsumed within the larger lower socioeconomic sector of the population and may, therefore, contain more vulnerable families where attachment is concerned. The scope for measured change may, therefore, be greater.

None of the studies involved middle-class data and so no meta-analysis was conducted.

There is a large effect size for the mixed location interventions and interventions primarily provided in the home are also significant on meta-analysis, although only three studies were included. Whether or not intervention location is important would need to be explored in future research. For example, it may be more difficult to put structure around interventions delivered in the home because of external factors.[235]

The meta-analysis examining the addition of the male to the female caregiver showed no significance when the male carer was involved in the intervention. It is not clear why this should be. It is possible that targeting a dyad in attachment work is the most effective way of improving attachment, as attachment usually focuses on the child's particular care-seeking from one primary individual, although infants are usually attached to more than one individual. It could be that involving the male carer somehow dilutes effects but no studies randomised between involving or not involving male caregivers, so no clear conclusion can be drawn.

Video-feedback intervention has been hailed as an important tool in generating insight for parents into helpful and unhelpful interactions in terms of the child's developmental needs. As a variety of interventions are being analysed together in both the video-feedback group and the non video-feedback group, findings should be treated with caution. Furthermore, the number of video-feedback sessions varies between interventions. Video feedback appears to be effective, but non-video-feedback interventions are also effective, and it may be that a combination of tools for eliciting insight and change should be considered in future research.

There is interesting evidence of large effect sizes when studies are targeting maternal sensitivity. This is concordant with the view of many clinicians that this is a productive area for interventions.[211]

There are various clinicians who believe that attachment work is more powerful when done in the dyad between the mother and the infant. Our meta-analysis produces an effect size that is highly significant statistically when working with the child and caregiver together.

© Queen's Printer and Controller of HMSO 2015. This work was produced by Wright *et al.* under the terms of a commissioning contract issued by the Secretary of State for Health. This issue may be freely reproduced for the purposes of private research and study and extracts (or indeed, the full report) may be included in professional journals provided that suitable acknowledgement is made and the reproduction is not associated with any form of advertising. Applications for commercial reproduction should be addressed to: NIHR Journals Library, National Institute for Health Research, Evaluation, Trials and Studies Coordinating Centre, Alpha House, University of Southampton Science Park, Southampton SO16 7NS, UK.

Appendix 7 Cochrane risk of bias tool

SEQUENCE GENERATION: was the allocation sequence adequately generated? [Short form: Adequate sequence generation?]	
Criteria for a judgement of 'YES' (i.e. low risk of bias)	The investigators describe a random component in the sequence generation process such as:<br><br>Referring to a random number table<br><br>Using a computer random number generator<br><br>Coin tossing<br><br>Shuffling cards or envelopes<br><br>Throwing dice<br><br>Drawing of lots<br><br>Minimisation*<br><br>*Minimisation may be implemented without a random element, and this is considered to be equivalent to being random
Criteria for the judgement of 'NO' (i.e. high risk of bias)	The investigators describe a non-random component in the sequence generation process. Usually, the description would involve some systematic, non-random approach, e.g.:<br><br>Sequence generated by odd or even date of birth<br><br>Sequence generated by some rule based on date (or day) of admission<br><br>Sequence generated by some rule based on hospital or clinic record number<br><br>Other non-random approaches happen much less frequently than the systematic approaches mentioned above and tend to be obvious. They usually involve judgement or some method of non-random categorisation of participants, e.g.:<br><br>Allocation by judgement of the clinician<br><br>Allocation by preference of the participant<br><br>Allocation based on the results of a laboratory test or a series of tests<br><br>Allocation by availability of the intervention
Criteria for the judgement of 'UNCLEAR' (uncertain risk of bias)	Insufficient information about the sequence generation process to permit judgement of 'Yes' or 'No'

© Queen's Printer and Controller of HMSO 2015. This work was produced by Wright *et al.* under the terms of a commissioning contract issued by the Secretary of State for Health. This issue may be freely reproduced for the purposes of private research and study and extracts (or indeed, the full report) may be included in professional journals provided that suitable acknowledgement is made and the reproduction is not associated with any form of advertising. Applications for commercial reproduction should be addressed to: NIHR Journals Library, National Institute for Health Research, Evaluation, Trials and Studies Coordinating Centre, Alpha House, University of Southampton Science Park, Southampton SO16 7NS, UK.

ALLOCATION CONCEALMENT: was allocation adequately concealed? [Short form: Allocation concealment?]	
Criteria for a judgement of 'YES' (i.e. low risk of bias)	Participants and investigators enrolling participants could not foresee assignment because one of the following, or an equivalent method, was used to conceal allocation:<br><br>Central allocation (including telephone, web-based and pharmacy-controlled randomisation)<br><br>Sequentially numbered drug containers of identical appearance<br><br>Sequentially numbered, opaque, sealed envelopes
Criteria for the judgement of 'NO' (i.e. high risk of bias)	Participants or investigators enrolling participants could possibly foresee assignments and thus introduce selection bias, such as allocation based on:<br><br>Using an open random allocation schedule (e.g. a list of random numbers)<br><br>Assignment envelopes were used without appropriate safeguards (e.g. if envelopes were unsealed or non-opaque or not sequentially numbered)<br><br>Alternation or rotation<br><br>Date of birth<br><br>Case record number<br><br>Any other explicitly unconcealed procedure
Criteria for the judgement of 'UNCLEAR' (uncertain risk of bias)	Insufficient information to permit judgement of 'Yes' or 'No'. This is usually the case if the method of concealment is not described or not described in sufficient detail to allow a definite judgement, for example if the use of assignment envelopes is described, but it remains unclear whether envelopes were sequentially numbered, opaque and sealed
BLINDING OF PARTICIPANTS, PERSONNEL AND OUTCOME ASSESSORS: was knowledge of the allocated interventions adequately prevented during the study? [Short form: Blinding?]	
Criteria for a judgement of 'YES' (i.e. low risk of bias)	Any one of the following:<br><br>No blinding, but the review authors judge that the outcome and the outcome measurement are not likely to be influenced by lack of blinding<br><br>Blinding of participants and key study personnel ensured, and unlikely that the blinding could have been broken<br><br>Either participants or some key study personnel were not blinded, but outcome assessment was blinded and the non-blinding of others unlikely to introduce bias
Criteria for the judgement of 'NO' (i.e. high risk of bias)	Any one of the following:<br><br>No blinding or incomplete blinding, and the outcome or outcome measurement is likely to be influenced by lack of blinding<br><br>Blinding of key study participants and personnel attempted, but likely that the blinding could have been broken<br><br>Either participants or some key study personnel were not blinded, and the non-blinding of others likely to introduce bias
Criteria for the judgement of 'UNCLEAR' (uncertain risk of bias)	Any one of the following:<br><br>Insufficient information to permit judgement of 'Yes' or 'No'<br><br>The study did not address this outcome

INCOMPLETE OUTCOME DATA: were incomplete outcome data adequately addressed? [Short form: Incomplete outcome data addressed?]	
Criteria for a judgement of 'YES' (i.e. low risk of bias)	Any one of the following:<br>No missing outcome data<br>Reasons for missing outcome data unlikely to be related to true outcome (for survival data, censoring unlikely to be introducing bias)<br>Missing outcome data balanced in numbers across intervention groups, with similar reasons for missing data across groups<br>For dichotomous outcome data, the proportion of missing outcomes compared with observed event risk not enough to have a clinically relevant impact on the intervention effect estimate<br>For continuous outcome data, plausible effect size (difference in means or standardised difference in means) among missing outcomes not enough to have a clinically relevant impact on observed effect size<br>Missing data have been imputed using appropriate methods
Criteria for the judgement of 'NO' (i.e. high risk of bias)	Any one of the following:<br>Reason for missing outcome data likely to be related to true outcome, with either imbalance in numbers or reasons for missing data across intervention groups<br>For dichotomous outcome data, the proportion of missing outcomes compared with observed event risk enough to induce clinically relevant bias in intervention effect estimate<br>For continuous outcome data, plausible effect size (difference in means or standardised difference in means) among missing outcomes enough to induce clinically relevant bias in observed effect size<br>'As-treated' analysis done with substantial departure of the intervention received from that assigned at randomisation<br>Potentially inappropriate application of simple imputation
Criteria for the judgement of 'UNCLEAR' (uncertain risk of bias)	Any one of the following:<br>Insufficient reporting of attrition/exclusions to permit judgement of 'Yes' or 'No' (e.g. number randomised not stated, no reasons for missing data provided)<br>The study did not address this outcome

© Queen's Printer and Controller of HMSO 2015. This work was produced by Wright *et al.* under the terms of a commissioning contract issued by the Secretary of State for Health. This issue may be freely reproduced for the purposes of private research and study and extracts (or indeed, the full report) may be included in professional journals provided that suitable acknowledgement is made and the reproduction is not associated with any form of advertising. Applications for commercial reproduction should be addressed to: NIHR Journals Library, National Institute for Health Research, Evaluation, Trials and Studies Coordinating Centre, Alpha House, University of Southampton Science Park, Southampton SO16 7NS, UK.

SELECTIVE OUTCOME REPORTING: are reports of the study free of suggestion of selective outcome reporting? [Short form: Free of selective reporting?]	
Criteria for a judgement of 'YES' (i.e. low risk of bias)	Any of the following:<br><br>The study protocol is available and all of the study's pre-specified (primary and secondary) outcomes that are of interest in the review have been reported in the pre-specified way<br><br>The study protocol is not available but it is clear that the published reports include all expected outcomes, including those that were pre-specified (convincing text of this nature may be uncommon)
Criteria for the judgement of 'NO' (i.e. high risk of bias)	Any one of the following:<br><br>Not all of the study's pre-specified primary outcomes have been reported<br><br>One or more primary outcomes is reported using measurements, analysis methods or subsets of the data (e.g. subscales) that were not pre-specified<br><br>One or more reported primary outcomes were not pre-specified (unless clear justification for their reporting is provided, such as an unexpected adverse effect)<br><br>One or more outcomes of interest in the review are reported incompletely so that they cannot be entered in a meta-analysis<br><br>The study report fails to include results for a key outcome that would be expected to have been reported for such a study
Criteria for the judgement of 'UNCLEAR' (uncertain risk of bias)	Insufficient information to permit judgement of 'Yes' or 'No'. It is likely that the majority of studies will fall into this category
OTHER POTENTIAL THREATS TO VALIDITY: was the study apparently free of other problems that could put it at a risk of bias? [Short form: Free of other bias?]	
Criteria for a judgement of 'YES' (i.e. low risk of bias)	The study appears to be free of other sources of bias
Criteria for the judgement of 'NO' (i.e. high risk of bias)	There is at least one important risk of bias. For example, the study:<br><br>Had a potential source of bias related to the specific study design used; or<br><br>Stopped early due to some data-dependent process (including a formal-stopping rule); or<br><br>Had extreme baseline imbalance; or<br><br>Has been claimed to have been fraudulent; or<br><br>Had some other problem
Criteria for the judgement of 'UNCLEAR' (uncertain risk of bias)	There may be a risk of bias, but there is either:<br><br>Insufficient information to assess whether an important risk of bias exists; or<br><br>Insufficient rationale or evidence that an identified problem will introduce bias

Appendix 8 The Quality assessment of diagnostic accuracy studies – version 2

QUADAS-2

Phase 1: State the review question:

Patients (setting, intended use of index test, presentation, prior testing):

Index test(s):

Reference standard and target condition:

Phase 2: Draw a flow diagram for the primary study

Phase 3: Risk of bias and applicability judgments

QUADAS-2 is structured so that 4 key domains are each rated in terms of the risk of bias and the concern regarding applicability to the research question (as defined above). Each key domain has a set of signalling questions to help reach the judgments regarding bias and applicability.

DOMAIN 1: PATIENT SELECTION

A. Risk of Bias

Describe methods of patient selection:

+ Was a consecutive or random sample of patients enrolled? Yes/No/Unclear

+ Was a case-control design avoided? Yes/No/Unclear

+ Did the study avoid inappropriate exclusions? Yes/No/Unclear

Could the selection of patients have introduced bias? **RISK: LOW/HIGH/UNCLEAR**

Describe included patients (prior testing, presentation, intended use of index test and setting):

Is there concern that the included patients do not match the review question? **CONCERN: LOW/HIGH/UNCLEAR**

© Queen's Printer and Controller of HMSO 2015. This work was produced by Wright *et al.* under the terms of a commissioning contract issued by the Secretary of State for Health. This issue may be freely reproduced for the purposes of private research and study and extracts (or indeed, the full report) may be included in professional journals provided that suitable acknowledgement is made and the reproduction is not associated with any form of advertising. Applications for commercial reproduction should be addressed to: NIHR Journals Library, National Institute for Health Research, Evaluation, Trials and Studies Coordinating Centre, Alpha House, University of Southampton Science Park, Southampton SO16 7NS, UK.

DOMAIN 2: INDEX TEST(S)

If more than one index test was used, please complete for each test.

A. Risk of Bias

Describe the index test and how it was conducted and interpreted:

+ Were the index test results interpreted without knowledge of the results of the reference standard? Yes/No/Unclear

+ If a threshold was used, was it pre-specified? Yes/No/Unclear

Could the conduct or interpretation of the index test have introduced bias? **RISK: LOW/HIGH/UNCLEAR**

B. Concerns regarding applicability

Is there concern that the index test, its conduct, or its interpretation introduced bias? **CONCERN: LOW/HIGH/UNCLEAR**

DOMAIN 3: REFERNCE

If more than one index test was used, please complete for each test.

A. Risk of Bias

Describe the reference standard and how it was conducted and interpreted:

+ Is the reference standard likely to correctly classify the target condition? Yes/No/Unclear

+ Were the reference standard results interpreted without knowledge of the results of the index text? Yes/No/Unclear

Could the reference standard, its conduct, or its interpretation have introduced this? **RISK: LOW/HIGH/UNCLEAR**

B. Concerns regarding applicability

Is there concern that the target condition as defined by the reference standard does not match the review question? **CONCERN: LOW/HIGH/UNCLEAR**

DOMAIN 4: FLOW AND TIMING

A. Risk of Bias

Describe any patients who did not receive the index test(s) and/or reference standard or who were excluded from the 2 x 2 table (refer to flow diagram):

Describe the time interval and any interventions between index test(s) and reference standard:

+ Was the appropriate interval between index test(s) and reference standard?	Yes/No/Unclear
+ Did all patients receive a reference standard?	Yes/No/Unclear
+ Did all patients receive the same reference standard?	Yes/No/Unclear

© Queen's Printer and Controller of HMSO 2015. This work was produced by Wright *et al.* under the terms of a commissioning contract issued by the Secretary of State for Health. This issue may be freely reproduced for the purposes of private research and study and extracts (or indeed, the full report) may be included in professional journals provided that suitable acknowledgement is made and the reproduction is not associated with any form of advertising. Applications for commercial reproduction should be addressed to: NIHR Journals Library, National Institute for Health Research, Evaluation, Trials and Studies Coordinating Centre, Alpha House, University of Southampton Science Park, Southampton SO16 7NS, UK.

EME
HS&DR
HTA
PGfAR
PHR

Part of the NIHR Journals Library
www.journalslibrary.nihr.ac.uk

This report presents independent research funded by the National Institute for Health Research (NIHR). The views expressed are those of the author(s) and not necessarily those of the NHS, the NIHR or the Department of Health

Published by the NIHR Journals Library
